# GIANT: galaxy-based tool for interactive analysis of transcriptomic data

**DOI:** 10.1038/s41598-020-76769-w

**Published:** 2020-11-16

**Authors:** Jimmy Vandel, Céline Gheeraert, Bart Staels, Jérôme Eeckhoute, Philippe Lefebvre, Julie Dubois-Chevalier

**Affiliations:** Univ. Lille, Inserm, CHU Lille, Institut Pasteur de Lille, U1011-EGID, 59000 Lille, France

**Keywords:** Bioinformatics, Data mining, Data processing, Microarrays, Software, Quality control, RNA sequencing

## Abstract

Transcriptomic analyses are broadly used in biomedical research calling for tools allowing biologists to be directly involved in data mining and interpretation. We present here GIANT, a Galaxy-based tool for Interactive ANalysis of Transcriptomic data, which consists of biologist-friendly tools dedicated to analyses of transcriptomic data from microarray or RNA-seq analyses. GIANT is organized into modules allowing researchers to tailor their analyses by choosing the specific set of tool(s) to analyse any type of preprocessed transcriptomic data. It also includes a series of tools dedicated to the handling of raw Affymetrix microarray data. GIANT brings easy-to-use solutions to biologists for transcriptomic data mining and interpretation.

## Introduction

Transcriptomic analyses have become a standard procedure to characterize biological systems and to monitor the molecular consequences of tested experimental conditions. Those analyses can be handled on the one hand by bioinformaticians using tools available essentially as R packages. More user-friendly solutions for biologists consist, on the other hand, of licensed softwares. In this context, we present here a series of freely available Galaxy-based tools dedicated to the analysis of transcriptomic data, which we have called Galaxy-based tool for Interactive ANalysis of Transcriptomic data (GIANT). Galaxy is a web-based platform offering access to tools enabling researchers without informatics expertise to perform computational analyses of large biomedical datasets^1^. The open source and collaborative characteristics of the Galaxy project supported by an active users and developers community constitute an attractive framework for GIANT.

GIANT consists of a series of Galaxy-based tools working as interrelated but independent modules. This allows a customized utilization through which users can choose to perform all or only a subset of the available data processing and analysis steps. GIANT puts together tools by encapsulating independently freely available R packages and programs, offering an easy access to both statistical analyses and interactive visualizations of data.

Nowadays, RNA sequencing (RNA-seq) has become the preferred technology for transcriptomic studies and numerous tools are already available in Galaxy to analyse RNA-seq datasets, especially for quality controls (QC)^2,3^ and differential analyses^4,5^. In this context, microarrays, which have been the reference technology for decades, have been neglected in recent Galaxy tool developments even through microarrays are still commonly used in laboratories and contribute to a great extent to available datasets in public databases. Indeed, surveying the Gene Expression Omnibus (GEO) database indicated that 60,000 transcriptomic studies based on microarrays were available, with 4270 new datasets (24% of transcriptomic studies) submitted between January 1st, 2019 and June 1st, 2020. Despite various tools developed to analyse transcriptomic data in Galaxy, none of them allows deep exploration of preprocessed data through interactive and highly customizable visualisations. GIANT offers the possibility to mine any type of preprocessed transcriptomic data such as RNA-seq normalized counts, microarray normalized expressions and most of differential analysis result files. In addition, to fill the lack of existing Galaxy tools for thorough microarray analyses, we added dedicated tools to handle Affymetrix microarrays raw data and to perform differential analyses with complex contrasts. Altogether, the GIANT suite comprises an unprecedented number of Galaxy-based tools for transcriptomic analyses.

In the next sections, inputs, ouputs and main characteristics of each Galaxy tool available in the GIANT suite are detailed. To demonstrate benefits of proposed tools for transcriptomic data analyses, specific workflows for RNA-seq and microarray data are presented. Finally, each workflow is illustrated in the “Results” section by an application on publicly available datasets.

## Methods

### Overview of the GIANT tool suite

**Figure 1:**
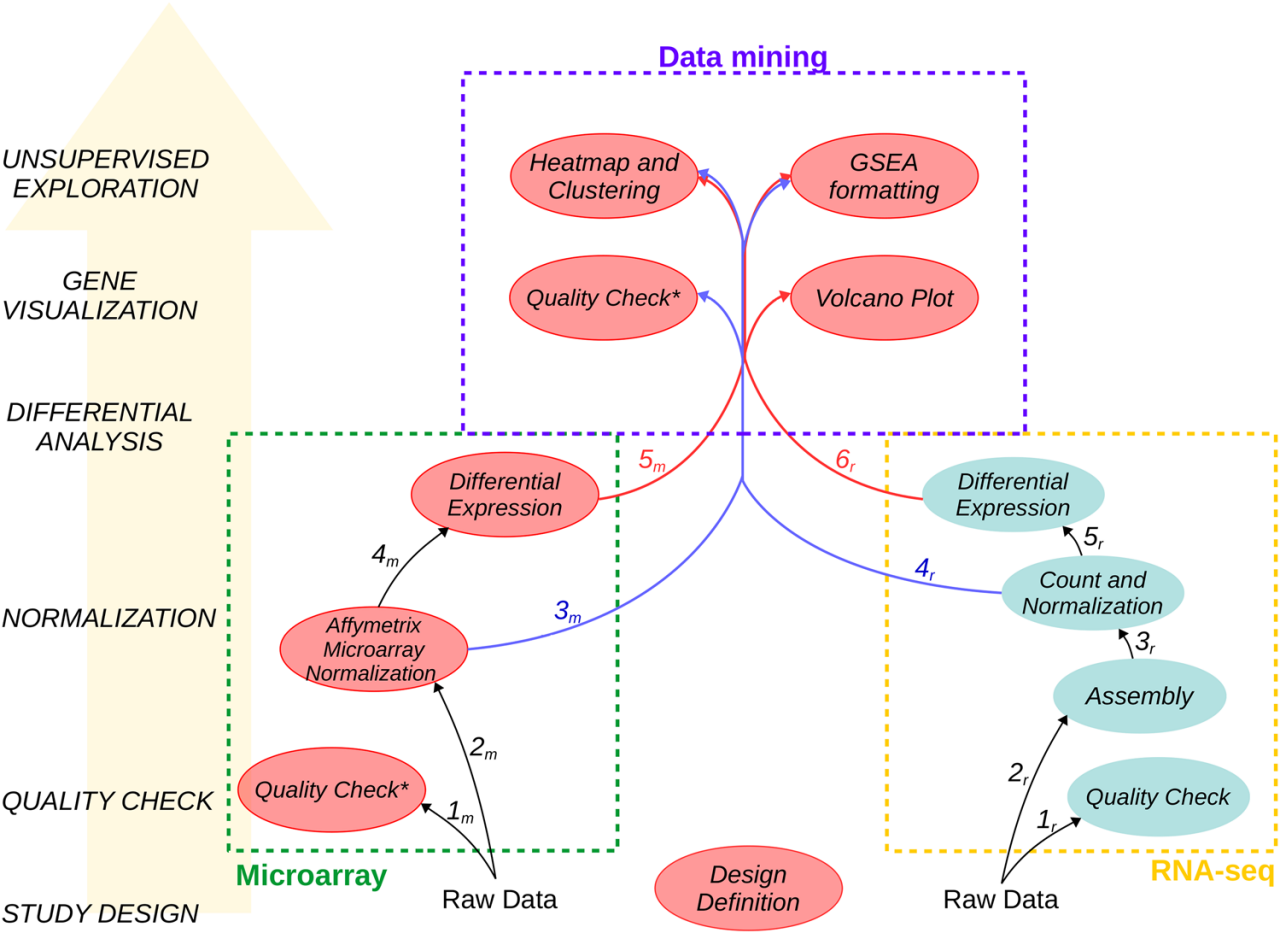
Transcriptomic analysis workflows using GIANT Galaxy tools. The general steps of the workflows are indicated on the left. Two workflows depending on the initial raw data are represented, both starting from the design definition (at the center bottom) to generic data mining analyses (purple dashed area at the top). Specific steps from quality check to differential analysis are indicated for microarray (left, green dashed area) and RNA-seq (right, yellow dashed area). Steps in which GIANT tools can be used are coloured in red, specific RNA-seq steps with available Galaxy tools are coloured in blue. Arrows $1_m$-$5_m$ and $1_r$-$6_r$ indicate the tools which should be used in consecutive steps for microarray and RNA-seq data analysis, respectively. Note that running the *Quality Check tool* both before and after data normalization is recommended (*marked).

The GIANT tool suite is composed of 7 independent Galaxy tools. While each tool can work independently, input and output formats have been standardized to facilitate the creation of integrated analysis workflows. Depending on the nature of transcriptomic data (microarray or RNA-seq), two specific workflows can be followed as shown in Fig. 1. Each workflow is described in the following sections and illustrated in the “Results” section. Beyond the initial processing steps (from QC to differential analysis steps) which are intrinsically specific to each transcriptomic technology, GIANT offers generic tools to mine any normalized data or differential analysis results through highly configurable tools and interactive results and plots (Data mining tool set in Fig. 1).

**Figure 2:**
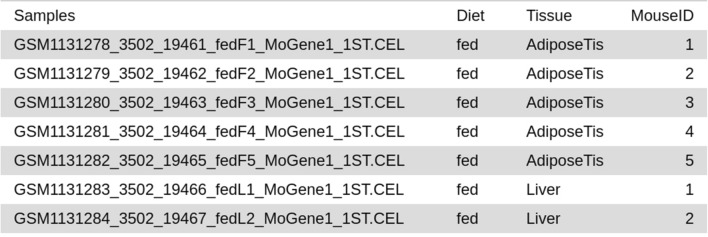
Extract of a factor file describing experimental design (GEO:GSE46495). For each sample listed in the first column, associated values for 3 experimental factors (Diet, Tissue and Mouse ID) are given in the 3 following columns.

**Figure 3:**
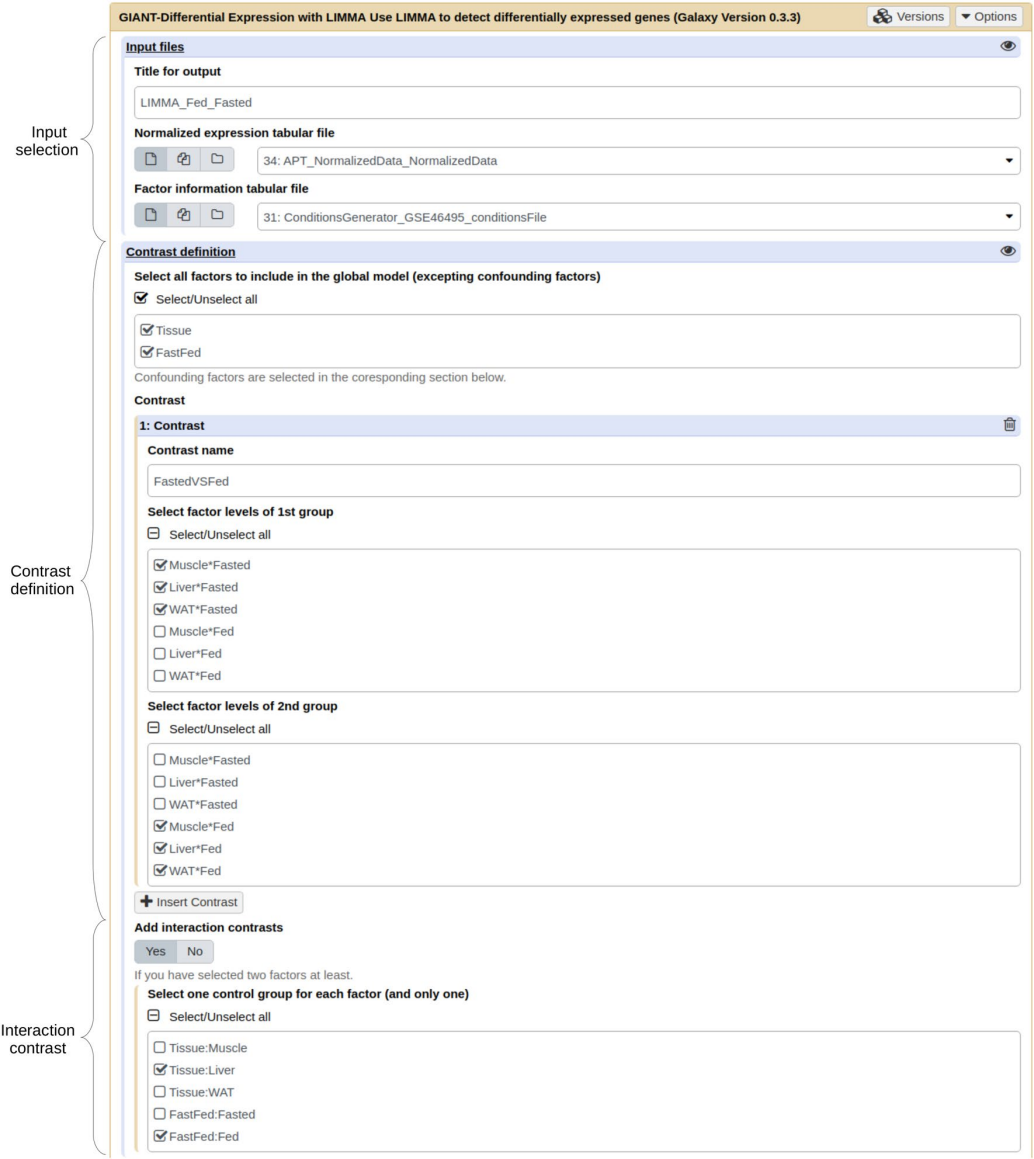
Partial view of the differential expression tool form showing input files selection, definition of contrasts and auto-generation of complex interaction contrasts. Both normalized data and study design files are selected input files. Definition of contrasts requires selection of factors among those automatically extracted from the design file and definition of groups (to compare first group to second group) as a selection of one or several factor value combinations (dynamically generated based on selected factors). Interaction contrasts are automatically defined as a function of the control value selected by the user.

Most GIANT tools have both graphical and non-graphical outputs.

**Non-graphical outputs** are provided as tabular files containing various statistics directly used as input in other GIANT tools allowing to build personalized workflows. The tabulated content of these outputs can also be inspected by hand if needed or exported for further mining as GO term enrichment analyses.

**Graphical outputs** play a major role in data analyses, facilitating results interpretation for users. Several R packages are employed to display clear and interactive plots. The *ggplot2* R package^6^ is the most used graphical package allowing to generate various kinds of plots such as bar plots, volcano plots and histograms. Thanks to its association with the *plotly* R package^7^, generated plots are easily converted to interactive plots. For interactive heatmaps, the *heatmaply* R package^8^ is used which also integrates *plotly* conversion facilities. These interactive graphical results are accessible through a *html* page which summarizes results making them easily accessible through hyperlinks. When necessary, tabular results are also displayed in an interactive *html* frame allowing users to search for specific genes or to reorder dynamically genes according to desired output values. As an example, the tabular output of the *Heatmap and clustering tool* contains cluster information for each gene which can be used to perform GO term enrichment analyses on each cluster. Thanks to the downloading option of the Galaxy interface, users can download all outputs including *html* pages. Thus, downloaded results can be opened and shared on any computer independently of Galaxy while maintaining interactivity, making these files particularly valuable for results sharing. Available *svg* format for snapshot also facilitates the integration of generated graphics in publications, since it offers high definition and the possibility to modify each *svg* element through free software as *Inkscape* (https://inkscape.org). Furthermore, as numerical information is displayed dynamically when the mouse hovers over the graph, only the requested information is displayed ensuring figure clarity.

In addition, each tool generates a text file (log file) where important information is recorded such as the version of the R packages used and warning messages. In case of error during tool execution, this log file may contain additional information to those displayed by the main Galaxy interface to help in error identification and correction.

### Description of transcriptomic workflows

The two depicted workflows in Fig. 1 consist of generic steps starting from the study design step to fill in experimental factor information for each sample using the *Factor table generation tool*. Then, depending on the origin of data (microarrays or RNA-seq), each workflow will require dedicated tools allowing for data processing from data quality check to differential analysis. Finally, both workflows share the visualization and unsupervised exploration tools. The main characteristics, inputs, outputs and options of each tool are described in the following paragraphs.

**Factor table generation tool** helps users to create tabular files containing factor information such as strain, treatment or diet, for each sample in a format appropriate for further use in other suite tools. Sample names are automatically captured from input files, which can be either tabular files containing sample names in the first row (as most of expression data files) or a raw file collection in which each file name is considered as a sample name. Users can create as many factors as needed and have to enumerate possible values for each factor. Then users assign samples to each factor value by selecting them amongst a list automatically generated from input files. The output file contains sample names in the first column and factor information in the following ones with factor names as headers as illustrated in Fig. 2.

#### Specific RNA-seq analysis steps

Unlike for microarrays, numerous tools and workflows to process RNA-seq data have been proposed in Galaxy, especially for alignment to the genome^9–11^, assembling/counting^12,13^ and differential analyses^4,5^. However the lack of flexible and configurable tools allowing to fully exploit the generated results is limiting and forces users to export their results out of Galaxy into graphical and statistical analysis software such as PRISM (graphpad : https://www.graphpad.com). GIANT offers the possibility to mine these results (normalized counts, differential statistics) in Galaxy and to compare information from various studies in a simple way. Amongst proposed data mining tools, the *Quality check tool* can generate 3D Principal Component Analysis (PCA) plots based on count data and the *Volcano plot tool* can be directly applied to output files of common RNA-seq differential Galaxy tools such as Limma-voom^5^ and SARTools^4^ that includes DESeq2^14^ and edgeR^15^, without the need for tedious data formatting steps. Output from the *Factor table generation tool* also fits with the majority of RNA-seq tools requiring a design file such as Limma-voom.

**Figure 4:**
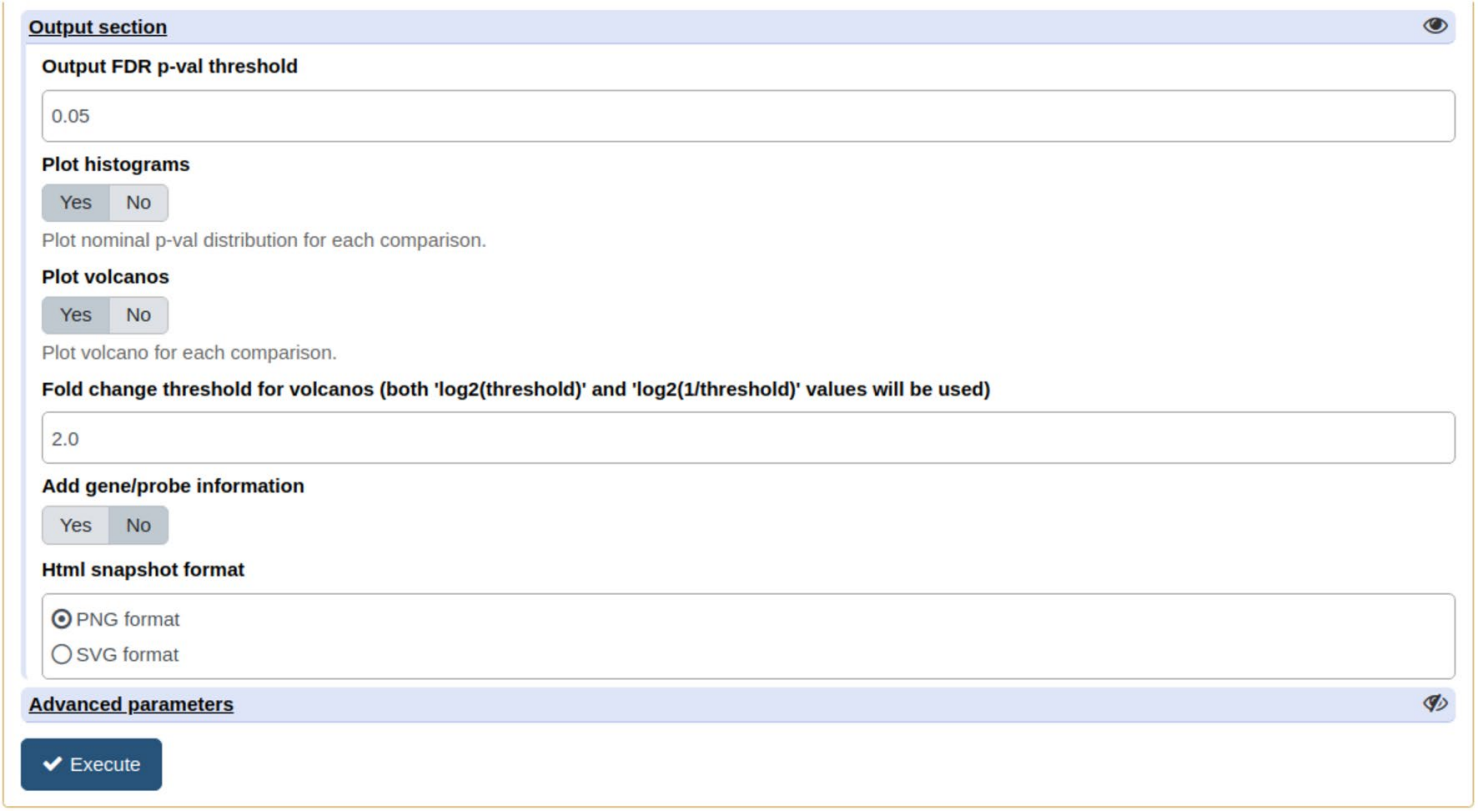
Partial view of the differential expression tool form showing tuning parameters and optional outputs. False Discovery Rate (FDR) and Fold Change cutoffs are tuned to filter out genes/probes from the output file. P-value histograms and volcano plots for each contrast are added to the output upon user request, as well as additional gene information extracted from public databases.

**Figure 5:**
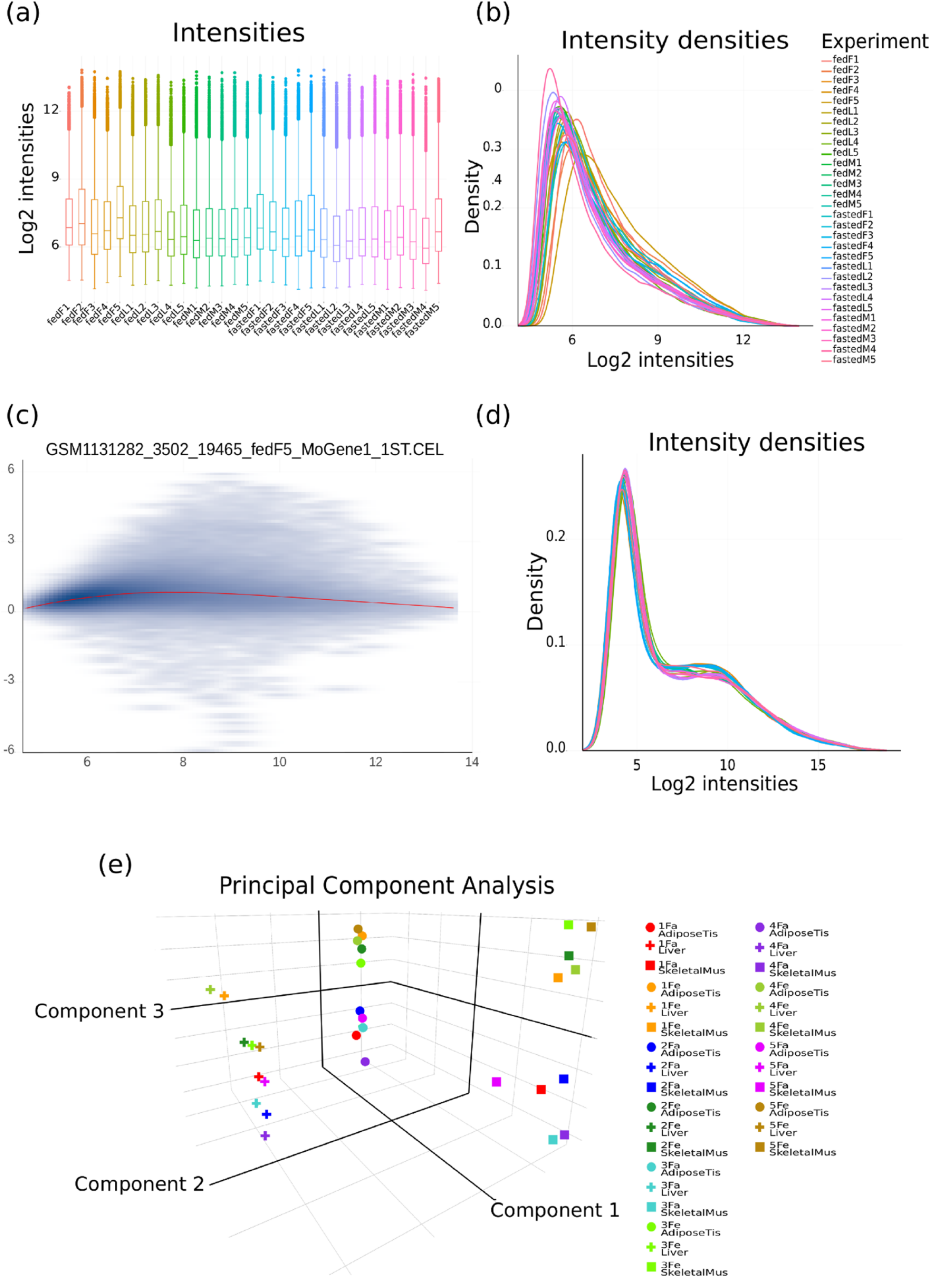
Graphics produced by the *Quality Check tool*. Before normalization: (**a**) boxplots and (**b**) histograms of raw data including all .CEL files and (**c**) MA-plot of a single .CEL file. After normalization: (**d**) histograms and (**e**) 3D PCA of normalized microarray expression data.

**Figure 6:**
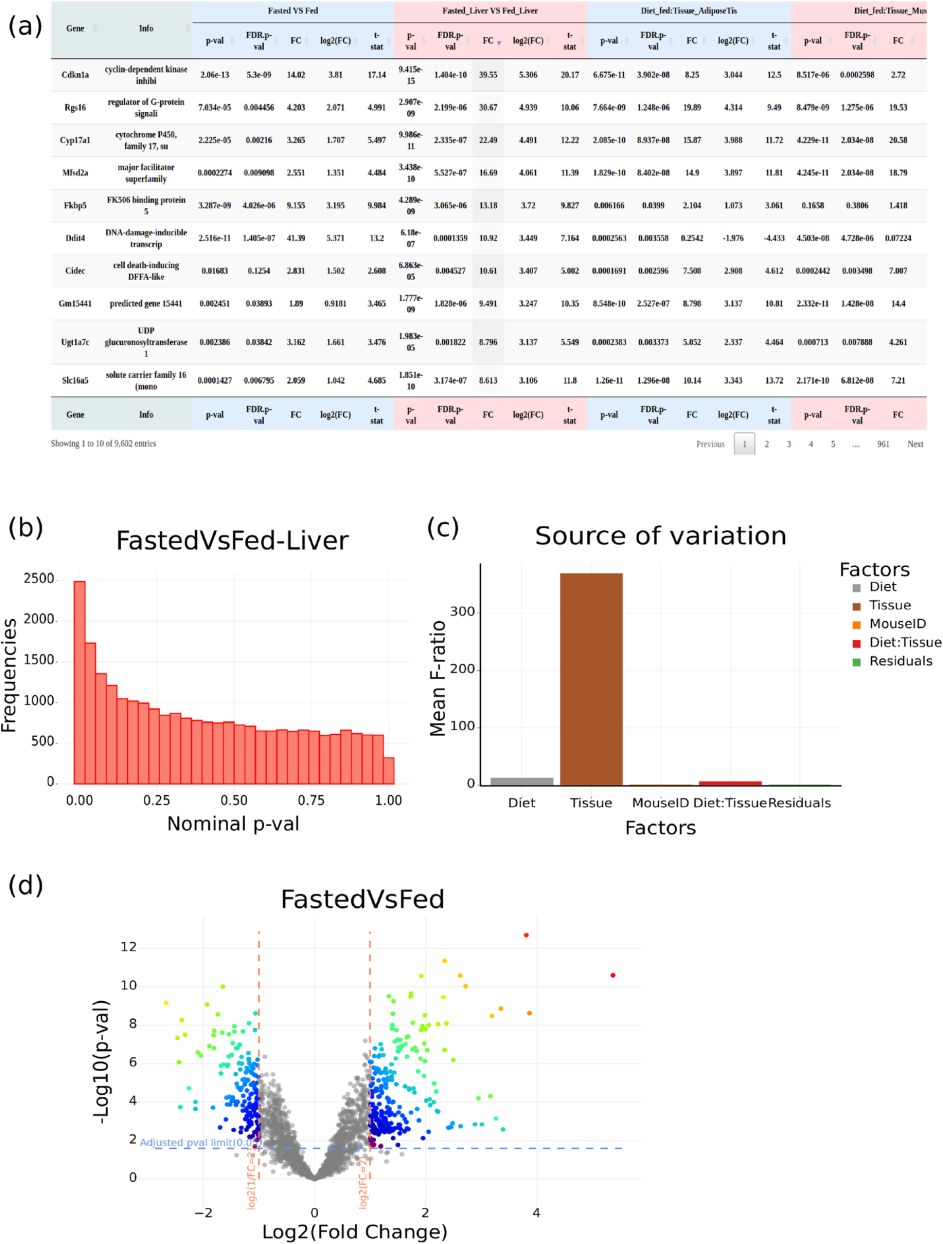
Results issued from the *Differential expression tool*: (**a**) differential statistics, (**b**) p-value distribution for a given contrast and (**c**) F-ratio bar plot for differential model factors; Graphic generated by the *Volcano plot tool*: (**d**) volcano plot generated from statistics computed by the *Differential expression tool*.

#### Specific microarray analysis steps

Despite available tools to analyse transcriptomic data in Galaxy, the limited number of microarray dedicated tools prevent from defining complete workflow for such data especially for unsupervised analysis. GIANT allows to build an end-to-end workflow with dedicated tools to normalize Affymetrix microarray raw data with various normalization options, to check data quality before and after normalization and to define complex contrasts for differential analyses with Limma. The resulting files can then be processed through the generic data mining tools (Fig. 1).

**Quality check tool** It allows to check the quality of transcriptomic data. Input data can be a collection of .CEL files to assess integrity of Affymetrix raw data (in a microarray workflow), or more broadly, any common tabular file containing expression data with samples in columns and transcripts/probes in lines. Upon users request, numerous plots can be generated: histograms and boxplots to display gene expression distribution in each sample, MA plots to compare expression in a sample to the median expression over all samples, and 3D PCA plots. For the latter, users can also load the corresponding factor file (Fig. 2) and select factors to customize dot shape and color based on factor values. This allows the easy identification of potential factors explaining dots coordinates in the 3 first principal components of PCA, thus variability in gene expression. All these plots help the user to visually identify technical bias between samples, thus requiring normalization and possibly sample removal. Additionally, in a microarray workflow with .CEL files as input, microarray images can be displayed for visual inspection. This tool is also used to ensure efficiency of the normalization step by checking uniformity of normalized data, before performing differential gene expression analyses.

**Affymetrix microarray normalization tool** It encapsulates the *apt-probeset-summarize* program from the *Affymetrix Power Tools* package (www.thermofisher.com/fr/fr/home/life-science/microarray-analysis/microarray-analysis-partners-programs/affymetrix-developers-network/affymetrix-power-tools.html) and requires .CEL files as input and array-specific configuration files (*.pgf, .clf, .cdf, .mps, .bgp*). Normalized data are saved in a tabular output file. Additionally, users can select annotation files to annotate probe IDs contained in the output tabular file. Several strategies are proposed for probes that share the same annotation: average expression, duplicate probes, keep probe with the highest/lowest variance.

Available normalization methods for Affymetrix microarrays are the standard RMA^16^ algorithm with or without additional GC correction and scaling, as recommended by Affymetrix (www.affymetrix.com/support/developer/powertools/changelog/VIGNETTE-apt-probeset-summarize-GCCN-SST.html). For more recent arrays (for example : Human/Mouse Transcriptome Arrays, Clariom D arrays), 2 normalization levels are available (probeset/core genes). To improve the user’s experience, the array configuration files necessary for *apt-probeset-summarize* execution can be durably hosted and referenced by the Galaxy instance through Galaxy configuration files, thus avoiding redundant file uploads.

**Differential expression tool** It encapsulates functions from the *Limma* R package^5^ dedicated to differential gene expression analyses in a microarray analysis workflow. The first input required is a generic tabular file containing microarray normalized expression data with sample names as first row and probe IDs/gene names as first column (as provided by *Affymetrix microarray normalization tool*). A tabular file containing the study design information is required as second input (e.g. generated by *Factor table generation tool*).

**Figure 7:**
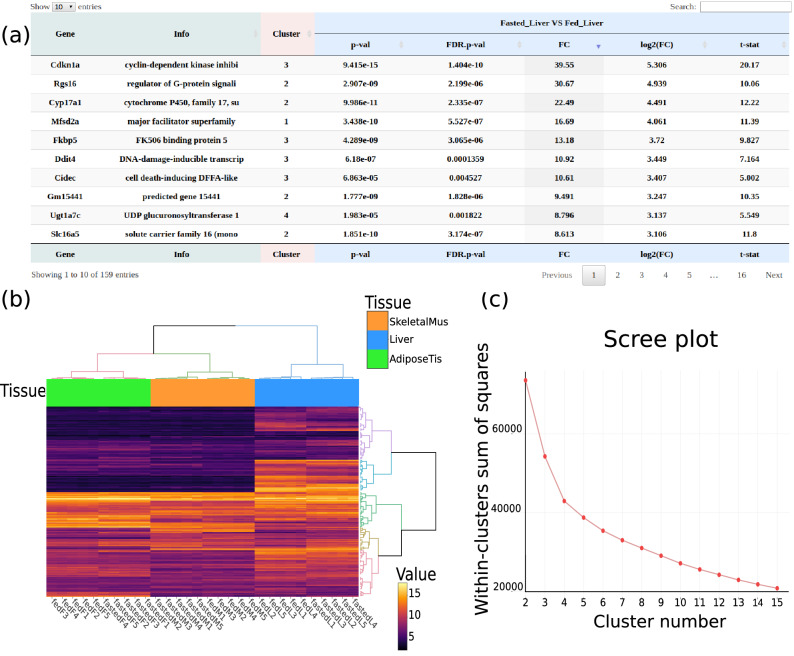
Results issued from the *Heatmap and clustering tool*: (**a**) cluster information added to differential statistics, (**b**) normalized microarray expression heatmap with hierarchical clustering of genes and samples and (**c**) scree plot showing within-clusters variance as a function of cluster number to assist in the cluster number choice.

**Figure 8:**
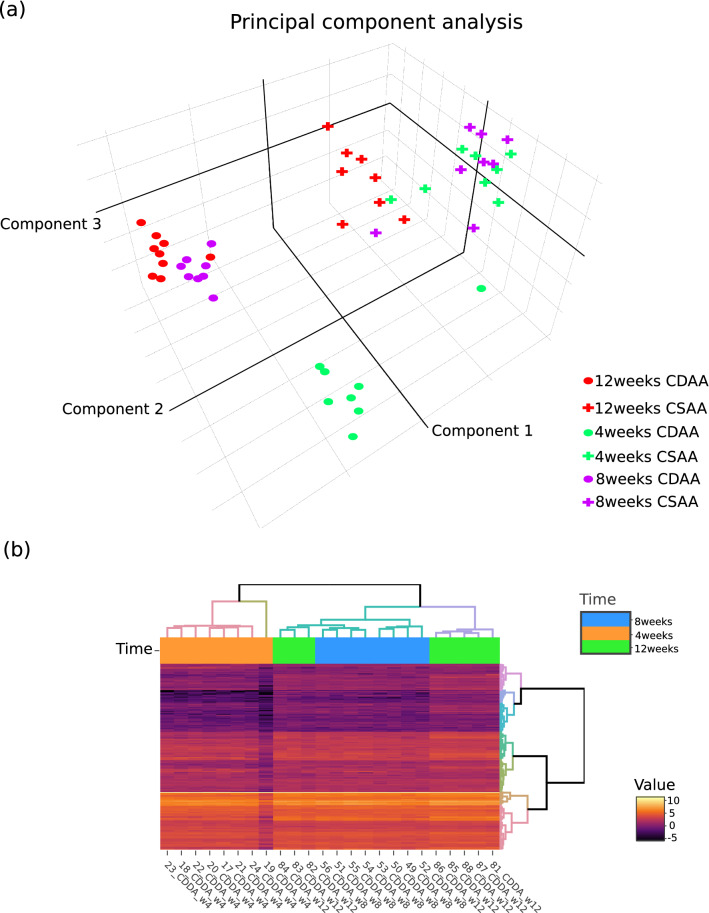
Graphics issued from the *Quality Check tool*: (**a**) 3D PCA of normalized RNA-seq expression data and the *Heatmap and clustering tool*: (**b**) normalized RNA-seq expression heatmap with hierarchical clustering of genes and samples.

Next, users define the Limma linear model according to the study design by selecting factors from which contrasts will be specified^17^. The list of available factors and possible combinations to define contrasts are automatically generated from the factor file provided as input. Users can specify as many contrasts as needed, each contrast being defined through the Galaxy interface without requiring equations, thus lowering the complexity for users. For each contrast (e.g. group A versus group B), two frames (respectively for groupA and group B) contain clickable combinations of factor values and allow users to build simple (one factor value combination per frame) or complex (several factor value combinations per frame) contrasts (Fig. 3). Furthermore, complex contrasts corresponding to factor interactions are automatically generated by the tool as soon as each requested factor value combination has been observed at least once in experimental design. If necessary, the user can add confounding factors to the Limma model. According to user’s specifications, main and confounding factors are included into the linear model as multiplicative and additive effects, respectively. In the case of paired-analysis, the “duplicateCorrelation” Limma function can be applied upon user’s request^18^.

Using user’s defined False Discovery Rate (FDR) and Fold Change (FC) cutoffs (Fig. 4), a filtered tabular file is generated, containing differential statistics for each contrast (FC, $log_2(FC)$, p-value, FDR and t-statistics). Gene information collected through the *biomaRt* R package^19^ can be added to this file. To facilitate contrast analysis, p-value histograms are plotted for each contrast as well as the F-ratio plot to measure the influence of each differential model factor in the expression variance. In addition to a specific tool designed to generate volcano plots (see the GIANT *Volcano plot tool* section below), volcano plots corresponding to defined contrasts can be directly drawn using the *Differential expression tool* after performing the whole differential analysis.

#### Unsupervised exploration of transcriptomic data

Following execution of specific tools to generate normalized expression data and differential analysis statistics, several GIANT tools can be applied to further mine these results. The main feature of these tools is their ability to make use of any tabular data.

**Volcano plot tool** It generates volcano plots from tabular files containing differential expression statistics. Multiple volcano plots can be drawn in a single execution without running again the whole differential analysis (in contrast to the *Differential expression tool*). This is particularly valuable and time-saving when assessing different FDR and FC thresholds for visualization purposes. The input tabular file must contain requested differential statistics (p-value and FC) in columns with the first column containing probe IDs/gene names. In addition to volcano plots, a tabular file containing statistical data (p-value, FDR and FC) is generated. Furthermore, information about genes/probes can be retrieved (using the *biomaRt* R package) and added to the output tabular file. For each volcano plot, users have to select columns containing p-values and FC values. Additional column containing already adjusted p-values can be selected if available. If they are not available, p-values will be adjusted for multiple testing using the FDR approach^20^. Thresholds for FC and adjusted p-values values can be specified for visualization purposes and for selecting genes of interest appearing in the tabular output file. Generated volcano plots exploit *ggplot2* and *plotly* R packages abilities to dynamically display gene information when hovering the mouse cursor over the plots.

**Heatmap and clustering tool** It allows to cluster expression data or statistical data generated by the GIANT *Differential expression tool*, or more broadly any data contained in tabular files with samples as columns, the typical output format of existing Galaxy tools. Depending on input data, all or a subset of columns are used for clustering. All columns are systematically considered for expression data clustering, whereas users have to select specific columns or contrasts of interest in the case of generic or *Differential expression tool* generated data, respectively. In addition to probe/gene clustering, a clustering of samples can be performed. To facilitate sample clustering interpretation, values of a user-selected factor can be displayed directly on the heatmap through a colored sidebar. Clustering results are represented through an interactive heatmap generated by *heatmaply* together with a tabular file containing cluster information for each probe/gene. A circular heatmap can also be plotted thanks to the *circlize* R package^21^. Additionally, a scree plot showing within-clusters variance as a function of cluster number, is generated to help users to choose the best number of probe/gene clusters.

Among numerous available options, a gene filtering function has been implemented. Filtering can be based on a gene list or from a differential statistic file with adjustable FC and FDR thresholds. As hierarchical clustering is applied independently on rows (probes/genes) and columns (samples/contrasts), users can select the specific number of clusters for each dimension. Various distance measures and agglomeration strategies are also available.

**GSEA formatting tool** It helps users to generate properly formatted files for Gene Set Enrichment Analysis (GSEA) designed by the Broad Institute^22^. The GSEA is one of the most commonly used tools to identify molecular pathways or particular Gene Ontology (GO) terms associated with differentially expressed genes. The *GSEA formatting tool* does not perform GSEA analyses on its own but facilitates its use. Required inputs depend on the planned analysis being either native or “pre-ranked” GSEA. For native GSEA analysis, the tool generates the formatted expression (.gct) and phenotype (.cls) files from the tabular normalized expression file and factor file respectively. For GSEA pre-ranked analysis, users have to select the statistic file produced by *Differential expression tool* as input, and choose the desired contrast and statistics to be used for ranking and thus generate the ranked gene list (.rnk).

## Results

This tool suite has been tried and tested in our laboratory to analyse or to reanalyse up to 30 transcriptomic studies. We present here 2 examples for which the GIANT tool suite was used to analyse microarray and RNA-seq data respectively.

### Microarray data analysis: case study

Microarray data from an in vivo mouse experiment (GEO:GSE46495) designed to study the transcriptomic response of white adipose tissue, liver, and skeletal muscle to fasting^23^ were analysed. This dataset is composed of 30 samples with fasted and fed conditions. For each condition, three different tissues and five biological replicates are available.

First, the *Factor table generation tool* was used to generate the factor file in accordance with the experimental design, including diet, tissue and replicate ID information for each sample (Fig. 2).

Then, the *Quality Check tool* was run on the 30 raw files (Affymetrix .CEL files) to detect potential technical issues during data collection. Graphical outputs (expression densities, boxplots, MA plot and chip image) are shown in Fig. 5a-c and in Supp. Fig. 1. Despite natural slight variation in raw expression profiles between samples, generally due to technical noise, all samples followed similar distributions. Thus, all of them were conserved for the normalization step in which technical variation will be removed.

The 30 samples were normalized using the *Affymetrix microarray normalization tool*, generating a single tabular file containing normalized data for all samples. Validation of the normalization procedure was requested before pursuing to differential analyses, thus the *Quality Check tool* was run again on the normalized expression file. The generated plot exhibited homogeneous expression profiles between samples (Fig. 5d) demonstrating efficiency of normalization. PCA was also performed to identify clustering of samples. Furthermore, by using the factor file as input, the link between the observed separation in PCA and any available factor was easily identified through dots of different colors and shapes (Fig. 5e). Thus, 3 sample clusters were clearly identified corresponding to the “*Tissue*” factor. In each of these 3 clusters, another separation was attributed to the “*Diet*” factor, however this separation was sharper in skeletal muscle and adipose tissue samples than in liver samples.

The *Differential expression tool* was then used to analyse the differentially expressed genes (DEGs) from the normalized data. The factor file was also used to define sample groups to be compared (Fig. 3). Differential statistics for all requested contrasts were generated as a tabular formatted file. To evaluate the statistical significance of tested contrasts, corresponding p-value distributions were plotted. The influence of each factor composing the differential model were summarized as an F-ratio bar plot (Fig. 6a–c). In our application, the strong influence of the “*Tissue*” factor was clearly identified using the F-ratio and confirmed by PCA. Volcano plots can be obtained simultaneously to differential gene expression analyses or subsequently using the *Volcano plot tool* with the statistics file (Fig. 6d).

Then, the *Heatmap and clustering tool* was run using the normalized expression data file to cluster statistically DEGs. We used embedded filtering options to restrict clustering to DEGs. The differential statistics file was used as an additional input, and user-defined FC and FDR thresholds were applied to the selected contrast. A second clustering was applied on samples (columns) with the associated sidebar colored based on the “*Tissue*” factor. The resulting clustering is represented by an interactive heatmap, and gene cluster annotation is given by the output tabular file (Fig. 7a,b). Again, influence of the “*Tissue*” factor was confirmed by the sample clustering in which the 3 principal clusters corresponded to this factor. Scree plot was also generated to assist users in choosing the optimal number of gene clusters by looking for the elbow in the curve, which was located at 4 clusters for this clustering (Fig. 7c).

Finally, in order to perform a GSEA for identified DEGs, the *GSEA formatting tool* was run using the differential statistics file as an input. A formatted file was generated containing a list of ranked genes according to differential statistics for a selected contrast, ready to be used as an input for the pre-ranked mode analysis of the GSEA software.

### RNA-seq data analysis: case study

To illustrate how GIANT can facilitate the analysis of RNA-seq data, we present here an example of GIANT-based analysis of RNA-seq data designed to identify new biomarkers in a rat dietary NASH model (GEO:GSE134715)^24^. This dataset is composed of 48 samples with the 2 diet conditions, CSAA (choline-supplemented L-amino acid-defined control diet) and CDAA (choline-deficient L-amino acid-defined NASH diet) and 3 timepoints (4, 8 and 12 weeks) with 8 animals per group. For this analysis, the read count matrix available on the GEO public repository was used.

First, differential analysis was performed on read count matrix using existing Galaxy tools. As the input of GIANT data mining tools can be any tabulated file without specific order of columns, results of most popular differential methods such as DESeq2, edgeR and Limma-voom can be mined with GIANT. In this application, to perform the differential analysis with the Galaxy-*limma-voom tool*^5^ (iuc-limma_voom repository from Galaxy toolshed), a study design file was necessary. This file was generated thanks to the *Factor table generation tool* that automatically extracts sample names from the input read count matrix to facilitate sample assignment to user defined diet and time factors values. Then, the design file was used to define sample groups to be compared in a differential analysis performed by the limma-voom tool. Limma-voom was used for both normalization of read count and differential analysis. After filtering out genes with low expression (less than 2 counts per million in at least 5 samples), the diet contrast “CDAA vs CSAA” was tested. Among generated files, one contained filtered normalized counts and a second differential analysis results with $log_2(FC)$, FDR and p-value statistics.

Then, the *Quality Check tool* was used to assess sample quality and to evaluate factor influence from the normalized counts file. Among generated plots, the 3D PCA (Fig. 8a) highlights a strong influence of the *Diet* factor in all samples and a *Time* factor effect specific to the CDAA diet samples in which the 4 weeks samples were clustered away from the 8 and 12 weeks samples. Interpretation of PCA was facilitated by the interactivity of the plot which permitted to dynamically rotate the graph and to display sample information when the mouse hovers over the dots. Furthermore, customization of dots color and shape based on diet and time factor values improved readability of the plot allowing factor values identification at first glance without need of unnecessary text fields.

Finally, the GIANT tools allowed to mine the limma-voom results and to prepare data for enrichment analyses. To determine biological pathways involved in CDAA diet samples according to the diet duration, the *Heatmap and clustering tool* was used to cluster expression of DEGs resulting from the limma-voom run. The normalized counts file was considered as a “generic file”, columns corresponding to CDAA conditions were selected and the study design file was used to color the sidebar based on time factor. The differential results file from limma-voom was used to filter genes, only those with FDR $< 0.01$ and $log_2(FC) > 1$ were considered for clustering. The generated heatmap (Fig. 8b) helps user to determine the CDAA diet samples sharing similar expression profiles over DEGs. As previously observed in PCA performed over all genes (Fig. 8a), the hierarchical clustering of samples associated to the heatmap identified a 4 weeks specific cluster, whereas 12 weeks samples were separated in 2 distinct clusters. All statistics related to DEGs analysis and their clustering was provided as a tabular output file (Supp. Fig. 2) allowing in-depth data mining such as GO terms/pathways enrichment analyses.

## Conclusion

Despite numerous tools available for transcriptomic data analyses and an active Galaxy community, to our knowledge no Galaxy-based tool suite is available to perform full analyses of transcriptomic data supported by interactive and customizable plots. Compared to existing Galaxy tools, the principal benefits of GIANT are: -interactive plots and tabular results to facilitate navigation and sharing of data; -multiple tunable parameters to improve analysis and visualization of data; -embedded filtering options in tools to cross information from several files and to reduce pre-processing operations; -generic inputs and outputs to use each tool independently or as a part of Galaxy analysis workflows. Tables 1, 2, and 3 summarize the benefits of the *GIANT tool suite*, the *Volcano plot* and the *Heatmap and clustering* tools with regards to existing Galaxy tools.

**Table 1:** Comparison of existing Galaxy tool suites.

Tool suite	Tunable	Modularity	Design definition	QC plots	Interactive ouput	Filter options	Cross studies	Param. clustering
GIANT suite	$\checkmark$	$\checkmark$	$\checkmark$	$\checkmark$	$\checkmark$	$\checkmark$	$\checkmark$	$\checkmark$
SARTools^4^	$\checkmark$	$\sim$	$\checkmark$	$\checkmark$		$\checkmark$		
LIMMA-voom^5^	$\checkmark$		$\checkmark$	$\checkmark$	$\sim$	$\checkmark$		

Compared functionalities from left to right are: tunable tool parameters, specific tool for each analysis step insuring modularity, possibility to build a design file, generation of QC plots, interactivity in generated files, input filtering options, possibility to cross information with another dataset and advanced clustering parameters. $\checkmark$and $\sim$ signs indicate that the corresponding functionality is fully and partially available in the tool suite respectively.

**Table 2:** Comparison of existing Galaxy volcano plot tools.

Volcano plot tool	Tunable	Generic input	Interactive plot	Interactive table	Filter options	Gene labeling
GIANT volcano tool	$\checkmark$	$\checkmark$	$\checkmark$	$\checkmark$	$\checkmark$	$\checkmark$
Volcanoplot	$\checkmark$	$\checkmark$			$\checkmark$	$\sim$
LIMMA-voom^5^	$\checkmark$		$\checkmark$	$\checkmark$	$\checkmark$	$\checkmark$

Compared functionalities from left to right are: tunable tool parameters, generic input, interactivity in generated plots, interactivity in generated tables, input filtering options and labeling of genes in the volcano plot. $\checkmark$and $\sim$ signs indicate that the corresponding functionality is fully and partially available in the volcano tool respectively.(*Volcanoplot* is available on Galaxy-toolshed : https://toolshed.g2.bx.psu.edu/view/iuc/volcanoplot/73b8cb5bddcd).

**Table 3:** Comparison of some existing Galaxy heatmap and clustering tools.

Heatmap and clustering tool	Generic input	Interactive output	Filter options	Cross studies	Param. clustering	Cluster assignation	Side colors	Colors definition
GIANT heatmap tool	$\checkmark$	$\checkmark$	$\checkmark$	$\checkmark$	$\checkmark$	$\checkmark$	$\checkmark$	$\checkmark$
LIMMA-voom^5^			$\checkmark$				$\checkmark$	
heatmap	$\checkmark$							$\checkmark$
heatmap_colormanipulation	$\checkmark$				$\checkmark$			$\checkmark$
plotHeatmap			$\checkmark$	$\checkmark$	$\checkmark$			
ggplot2_heatmap2^6^	$\checkmark$				$\checkmark$			$\sim$

Compared functionalities from left to right are: generic input, interactivity in generated files, input filtering options, possibility to cross information with another dataset, advanced clustering parameters, retrieve cluster assignation, display colored side bar and color personalization. $\checkmark$and $\sim$ signs indicate that the corresponding functionality is fully and partially available in the heatmap and clustering tool respectively. (heatmap available at https://toolshed.g2.bx.psu.edu/view/guru-ananda/heatmap/dbd447fcd3e4 ; heatmap_colormanipulation available at https://toolshed.g2.bx.psu.edu/view/mir-bioinf/heatmap_colormanipulation/58772ebbeb9f ; plotHeatmap available at https://toolshed.g2.bx.psu.edu/view/earlhaminst/plotheatmap/bd8fd161908b).

GIANT is freely available to the community, each tool can be downloaded to any Galaxy instance from the Galaxy Main Tool Shed repository and the full source code is available on GitHub.

## Supplementary information

Supplementary Figure.

Supplementary Information.

## Data Availability

*Availability and versioning*: The GIANT source code is freely available on GitHub (https://github.com/juliechevalier/GIANT) under GNU General Public Licence version 3. The Galaxy tool suite is available on the Galaxy Main Tool Shed (https://toolshed.g2.bx.psu.edu; name:suite_giant; owner:vandelj) and can be installed on any Galaxy instance. GIANT tools have been installed and tested on Galaxy releases v18.09 and v19.09. Tools are versioned according to tool functionalities and intput/output formats. As the Galaxy platform allows the independent selection of different version for each installed tool, compatibility issues may occur if compatibility rules are not respected. Version compatibilities are summarized in the “README.rst” file, available on the GIANT GitHub repository. Tool versions used for this article were : *Quality check tool* (v 0.1.2), *Affymetrix microarray normalization tool* (v 0.1.1), *Factor table generation tool* (v 0.1.1), *Differential expression tool* (v 0.3.7), *Volcano plot tool* (v 0.3.1), *Heatmap and clustering tool* (v 0.5.0) and *GSEA formatting tool* (v 0.2.0). *Tools requirements*: Galaxy tool dependencies are managed through Conda environments. These environments are automatically created during the tool installation and follow requirements listed during tool development to avoid any additional manual installation. However, possible errors due to missing dependencies may occur, during tool execution depending on local computing platform. In such case, the manual installation is needed. Please read the troubleshooting information section in the GIANT documentation for more information. *Availability of supporting data and materials*: The GIANT documentation is available on the GIANT GitHub repository. This documentation contains a troubleshooting section and a step-by-step tutorial. Microarray raw data and RNA-seq read count matrix used in the Application section are available at NCBI (www.ncbi.nlm.nih.gov) GEO:GSE46495 and GEO:GSE134715 respectively. Screenshots of GIANT tool parameters, required input and output files for each step of presented microarray and RNA-seq analyses are available as Supplementary data 1. Several microarray configuration files required by the *APT-Normalization tool* are provided in the GIANT zenodo page https://doi.org/10.5281/zenodo.3908285. This repository contains pgf, clf, bgp, mps, cdf and formatted annotations files for MOE430A 1.0, MOE430B 1.0, MOE430 2.0, Mo/Hugene 1.0, 1.1, 2.0, HTA, MTA, mouse/human Clariom S and mouse/human Clariom D Affymetrix microarrays. Thanks to the available *svg* format for interactive plot screenshots, clarity of figures displayed in this manuscript was directly improved by increasing the size of axis labels, titles and legends using Inkscape software.
