# Supplementary material for: GIANT: galaxy-based tool for interactive analysis of transcriptomic data
Source: Sci Rep. 2020 Nov 16;10:19835. doi: 10.1038/s41598-020-76769-w (PMC7670435; doi:10.1038/s41598-020-76769-w)
Supplement: Supplementary file 1 — Supplementary Figure. [file 41598_2020_76769_MOESM1_ESM.pdf]

GIANT : Galaxy-based tool for Interactive ANalysis of Transcriptomic data

Jimmy Vandel[1,*], **Céline Gheeraert**[1], **Bart Staels**[1], **Jérôme Eeckhoute**[1], **Philippe Lefebvre**[1], **and Julie Dubois-Chevalier**[1,*]

[1]Univ. Lille, Inserm, CHU Lille, Institut Pasteur de Lille, U1011-EGID, F-59000 Lille, France.
[*]jimmy.vandel@inserm.fr; julie.chevalier@inserm.fr

Supplementary Material

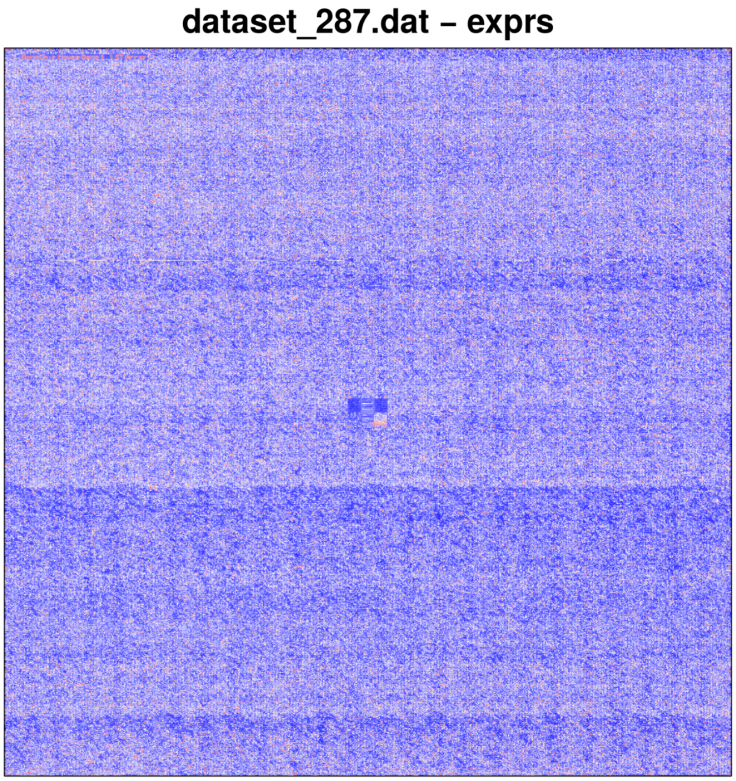

Supp. Figure 1. Graphic issued from the *Quality Check tool*: microarray image of a .CEL file.

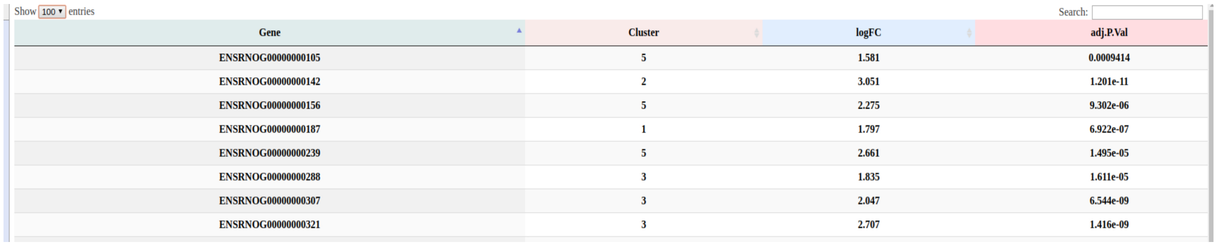

Show 100 entries Search:

Gene	Cluster	logFC	adj.P.Val
ENSRNOG00000000105	5	1.581	0.0009414
ENSRNOG00000000142	2	3.051	1.201e-11
ENSRNOG00000000156	5	2.275	9.302e-06
ENSRNOG00000000187	1	1.797	6.922e-07
ENSRNOG00000000239	5	2.661	1.495e-05
ENSRNOG00000000288	3	1.835	1.611e-05
ENSRNOG00000000307	3	2.047	6.544e-09
ENSRNOG00000000321	3	2.707	1.416e-09

Supp. Figure 2. Result issued from the *Heatmap and clustering tool*: cluster information added to differential statistics.
