# Supplementary material for: GIANT: galaxy-based tool for interactive analysis of transcriptomic data
Source: Sci Rep. 2020 Nov 16;10:19835. doi: 10.1038/s41598-020-76769-w (PMC7670435; doi:10.1038/s41598-020-76769-w)
Supplement: Supplementary file 2 — Supplementary Information. [file 41598_2020_76769_MOESM2_ESM.zip › Tools_InputOutput_Parameters/MicroArray_workflow/Step1-QCraw/Outputs/QCplot_rawData_HTML.html_64.html]

### Histograms

Histograms1

### Boxplots

Boxplots1

### MA plots (show/hide)

MAplot GSM1131298\_3502\_19481\_fastedL1\_MoGene1\_1ST.CEL

MAplot GSM1131297\_3502\_19480\_fastedF5\_MoGene1\_1ST.CEL

MAplot GSM1131296\_3502\_19479\_fastedF4\_MoGene1\_1ST.CEL

MAplot GSM1131295\_3502\_19478\_fastedF3\_MoGene1\_1ST.CEL

MAplot GSM1131294\_3502\_19477\_fastedF2\_MoGene1\_1ST.CEL

MAplot GSM1131293\_3502\_19476\_fastedF1\_MoGene1\_1ST.CEL

MAplot GSM1131292\_3502\_19475\_fedM5\_MoGene1\_1ST.CEL

MAplot GSM1131291\_3502\_19474\_fedM4\_MoGene1\_1ST.CEL

MAplot GSM1131290\_3502\_19473\_fedM3\_MoGene1\_1ST.CEL

MAplot GSM1131289\_3502\_19472\_fedM2\_MoGene1\_1ST.CEL

MAplot GSM1131307\_3502\_19490\_fastedM5\_MoGene1\_1ST.CEL

MAplot GSM1131288\_3502\_19471\_fedM1\_MoGene1\_1ST.CEL

MAplot GSM1131287\_3502\_19470\_fedL5\_MoGene1\_1ST.CEL

MAplot GSM1131286\_3502\_19469\_fedL4\_MoGene1\_1ST.CEL

MAplot GSM1131285\_3502\_19468\_fedL3\_MoGene1\_1ST.CEL

MAplot GSM1131284\_3502\_19467\_fedL2\_MoGene1\_1ST.CEL

MAplot GSM1131283\_3502\_19466\_fedL1\_MoGene1\_1ST.CEL

MAplot GSM1131282\_3502\_19465\_fedF5\_MoGene1\_1ST.CEL

MAplot GSM1131281\_3502\_19464\_fedF4\_MoGene1\_1ST.CEL

MAplot GSM1131280\_3502\_19463\_fedF3\_MoGene1\_1ST.CEL

MAplot GSM1131279\_3502\_19462\_fedF2\_MoGene1\_1ST.CEL

MAplot GSM1131306\_3502\_19489\_fastedM4\_MoGene1\_1ST.CEL

MAplot GSM1131278\_3502\_19461\_fedF1\_MoGene1\_1ST.CEL

MAplot GSM1131305\_3502\_19488\_fastedM3\_MoGene1\_1ST.CEL

MAplot GSM1131304\_3502\_19487\_fastedM2\_MoGene1\_1ST.CEL

MAplot GSM1131303\_3502\_19486\_fastedM1\_MoGene1\_1ST.CEL

MAplot GSM1131302\_3502\_19485\_fastedL5\_MoGene1\_1ST.CEL

MAplot GSM1131301\_3502\_19484\_fastedL4\_MoGene1\_1ST.CEL

MAplot GSM1131300\_3502\_19483\_fastedL3\_MoGene1\_1ST.CEL

MAplot GSM1131299\_3502\_19482\_fastedL2\_MoGene1\_1ST.CEL

### Microarray

Microarray GSM1131298\_3502\_19481\_fastedL1\_MoGene1\_1ST.CEL

Microarray GSM1131297\_3502\_19480\_fastedF5\_MoGene1\_1ST.CEL

Microarray GSM1131296\_3502\_19479\_fastedF4\_MoGene1\_1ST.CEL

Microarray GSM1131295\_3502\_19478\_fastedF3\_MoGene1\_1ST.CEL

Microarray GSM1131294\_3502\_19477\_fastedF2\_MoGene1\_1ST.CEL

Microarray GSM1131293\_3502\_19476\_fastedF1\_MoGene1\_1ST.CEL

Microarray GSM1131292\_3502\_19475\_fedM5\_MoGene1\_1ST.CEL

Microarray GSM1131291\_3502\_19474\_fedM4\_MoGene1\_1ST.CEL

Microarray GSM1131290\_3502\_19473\_fedM3\_MoGene1\_1ST.CEL

Microarray GSM1131289\_3502\_19472\_fedM2\_MoGene1\_1ST.CEL

Microarray GSM1131307\_3502\_19490\_fastedM5\_MoGene1\_1ST.CEL

Microarray GSM1131288\_3502\_19471\_fedM1\_MoGene1\_1ST.CEL

Microarray GSM1131287\_3502\_19470\_fedL5\_MoGene1\_1ST.CEL

Microarray GSM1131286\_3502\_19469\_fedL4\_MoGene1\_1ST.CEL

Microarray GSM1131285\_3502\_19468\_fedL3\_MoGene1\_1ST.CEL

Microarray GSM1131284\_3502\_19467\_fedL2\_MoGene1\_1ST.CEL

Microarray GSM1131283\_3502\_19466\_fedL1\_MoGene1\_1ST.CEL

Microarray GSM1131282\_3502\_19465\_fedF5\_MoGene1\_1ST.CEL

Microarray GSM1131281\_3502\_19464\_fedF4\_MoGene1\_1ST.CEL

Microarray GSM1131280\_3502\_19463\_fedF3\_MoGene1\_1ST.CEL

Microarray GSM1131279\_3502\_19462\_fedF2\_MoGene1\_1ST.CEL

Microarray GSM1131306\_3502\_19489\_fastedM4\_MoGene1\_1ST.CEL

Microarray GSM1131278\_3502\_19461\_fedF1\_MoGene1\_1ST.CEL

Microarray GSM1131305\_3502\_19488\_fastedM3\_MoGene1\_1ST.CEL

Microarray GSM1131304\_3502\_19487\_fastedM2\_MoGene1\_1ST.CEL

Microarray GSM1131303\_3502\_19486\_fastedM1\_MoGene1\_1ST.CEL

Microarray GSM1131302\_3502\_19485\_fastedL5\_MoGene1\_1ST.CEL

Microarray GSM1131301\_3502\_19484\_fastedL4\_MoGene1\_1ST.CEL

Microarray GSM1131300\_3502\_19483\_fastedL3\_MoGene1\_1ST.CEL

Microarray GSM1131299\_3502\_19482\_fastedL2\_MoGene1\_1ST.CEL
