# Supplementary figures and images for: GIANT: galaxy-based tool for interactive analysis of transcriptomic data
Source: Sci Rep. 2020 Nov 16;10:19835. doi: 10.1038/s41598-020-76769-w (PMC7670435; doi:10.1038/s41598-020-76769-w)

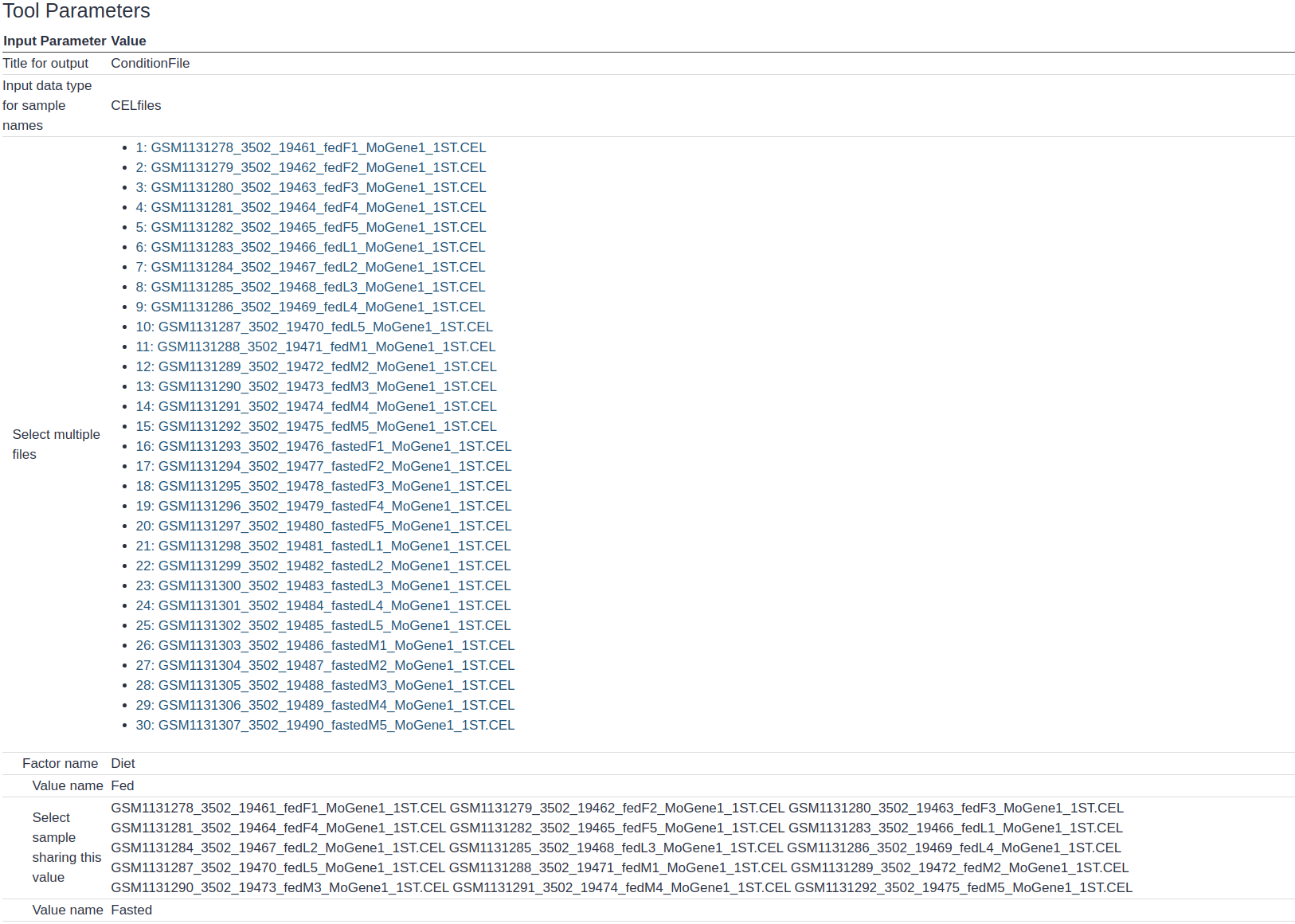

Supplement: Supplementary file 2 — Supplementary Information. [file 41598_2020_76769_MOESM2_ESM.zip › Tools_InputOutput_Parameters/MicroArray_workflow/Step0-FactorFile/Step0-FactorFile_A.toolParameters.png]

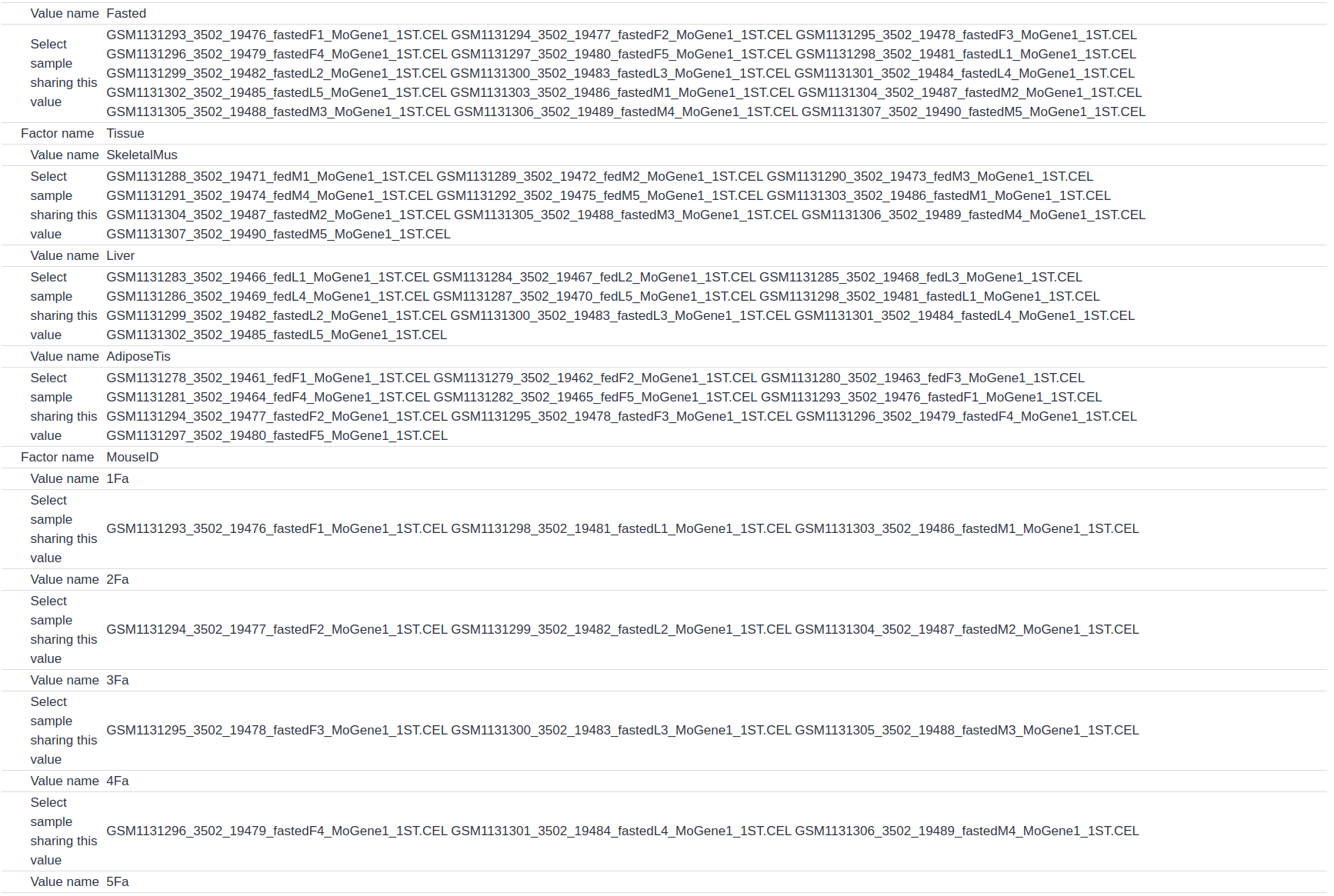

Supplement: Supplementary file 2 — Supplementary Information. [file 41598_2020_76769_MOESM2_ESM.zip › Tools_InputOutput_Parameters/MicroArray_workflow/Step0-FactorFile/Step0-FactorFile_B.toolParameters.png]

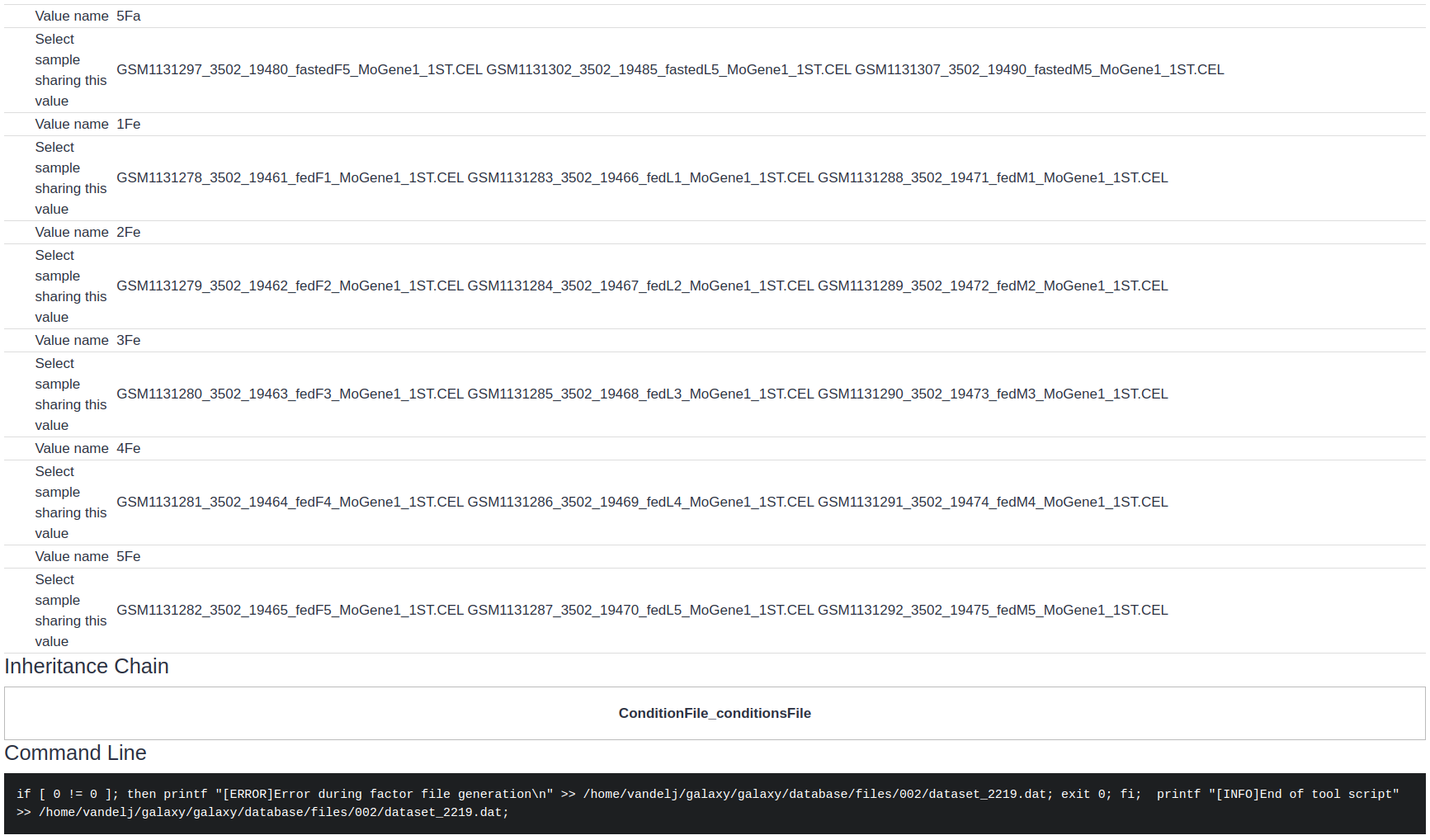

Supplement: Supplementary file 2 — Supplementary Information. [file 41598_2020_76769_MOESM2_ESM.zip › Tools_InputOutput_Parameters/MicroArray_workflow/Step0-FactorFile/Step0-FactorFile_C.toolParameters.png]

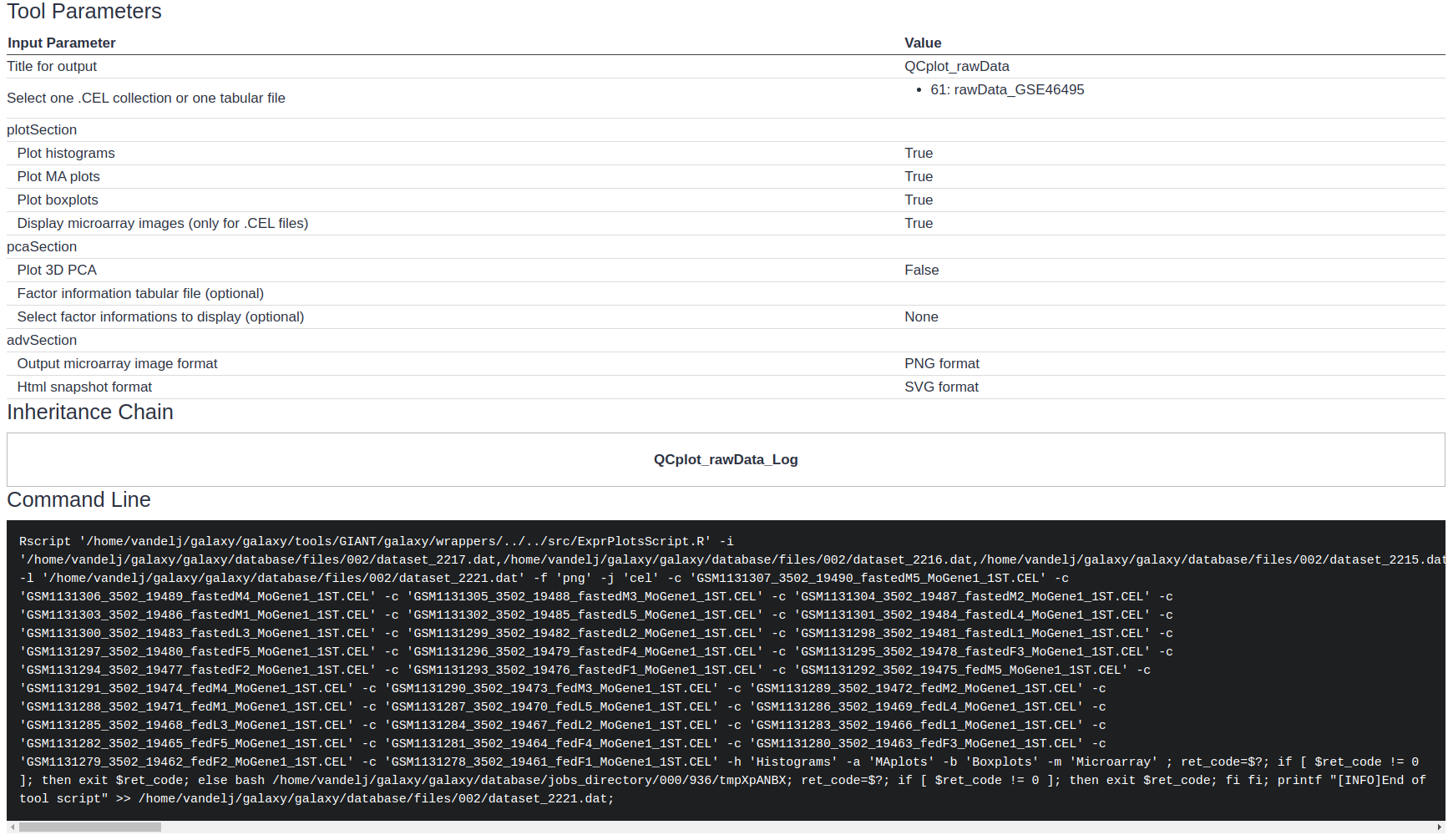

Supplement: Supplementary file 2 — Supplementary Information. [file 41598_2020_76769_MOESM2_ESM.zip › Tools_InputOutput_Parameters/MicroArray_workflow/Step1-QCraw/Step1-QCraw.toolParameters.png]

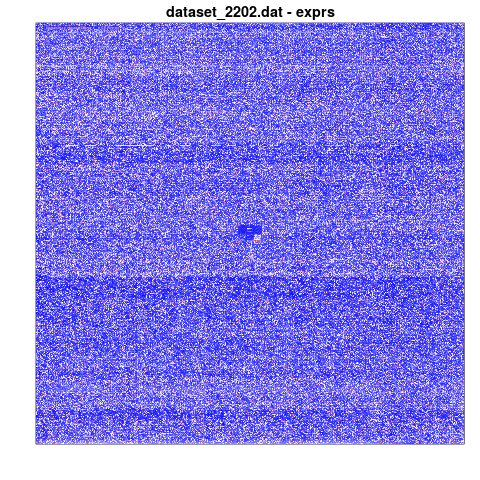

Supplement: Supplementary file 2 — Supplementary Information. [file 41598_2020_76769_MOESM2_ESM.zip › Tools_InputOutput_Parameters/MicroArray_workflow/Step1-QCraw/Outputs/extra_files_path_64/Microarray_Condition16.png]

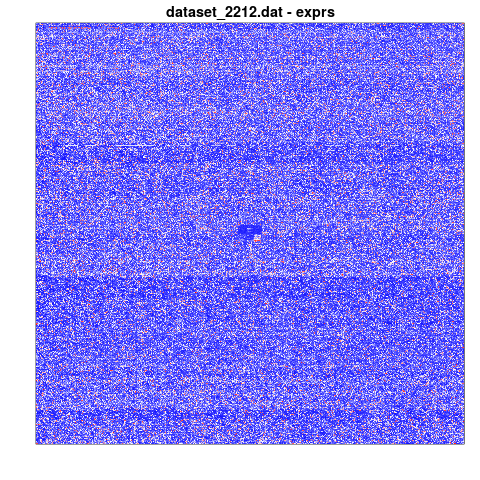

Supplement: Supplementary file 2 — Supplementary Information. [file 41598_2020_76769_MOESM2_ESM.zip › Tools_InputOutput_Parameters/MicroArray_workflow/Step1-QCraw/Outputs/extra_files_path_64/Microarray_Condition6.png]

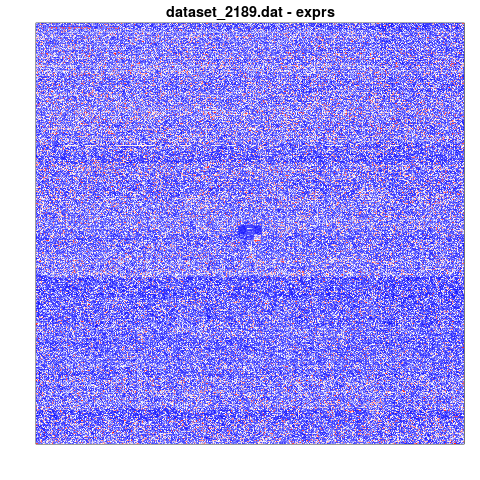

Supplement: Supplementary file 2 — Supplementary Information. [file 41598_2020_76769_MOESM2_ESM.zip › Tools_InputOutput_Parameters/MicroArray_workflow/Step1-QCraw/Outputs/extra_files_path_64/Microarray_Condition29.png]

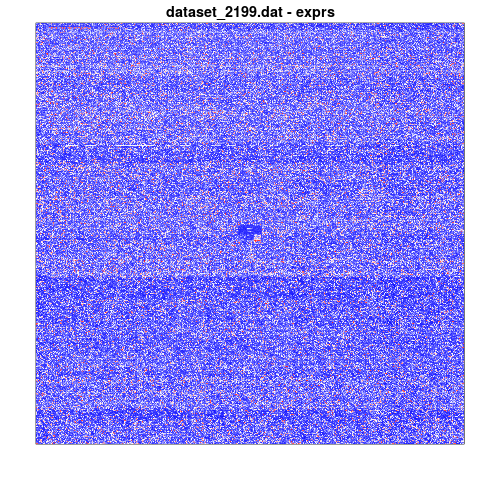

Supplement: Supplementary file 2 — Supplementary Information. [file 41598_2020_76769_MOESM2_ESM.zip › Tools_InputOutput_Parameters/MicroArray_workflow/Step1-QCraw/Outputs/extra_files_path_64/Microarray_Condition19.png]

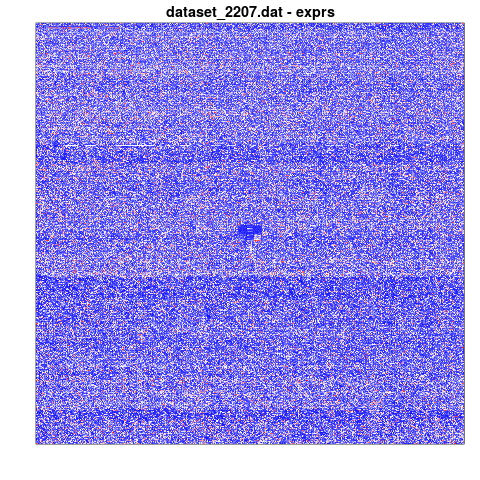

Supplement: Supplementary file 2 — Supplementary Information. [file 41598_2020_76769_MOESM2_ESM.zip › Tools_InputOutput_Parameters/MicroArray_workflow/Step1-QCraw/Outputs/extra_files_path_64/Microarray_Condition11.png]

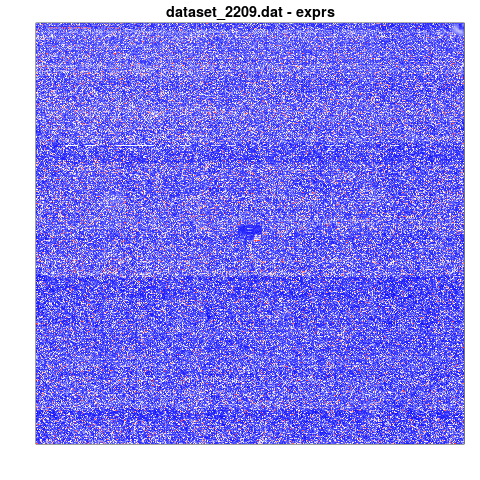

Supplement: Supplementary file 2 — Supplementary Information. [file 41598_2020_76769_MOESM2_ESM.zip › Tools_InputOutput_Parameters/MicroArray_workflow/Step1-QCraw/Outputs/extra_files_path_64/Microarray_Condition9.png]

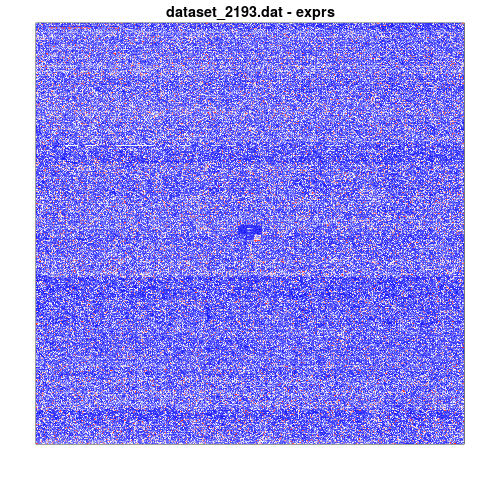

Supplement: Supplementary file 2 — Supplementary Information. [file 41598_2020_76769_MOESM2_ESM.zip › Tools_InputOutput_Parameters/MicroArray_workflow/Step1-QCraw/Outputs/extra_files_path_64/Microarray_Condition25.png]

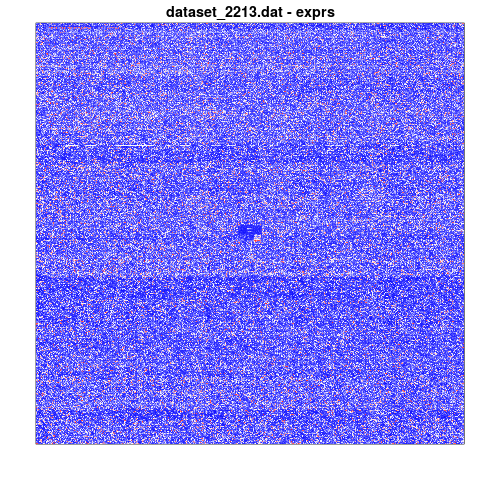

Supplement: Supplementary file 2 — Supplementary Information. [file 41598_2020_76769_MOESM2_ESM.zip › Tools_InputOutput_Parameters/MicroArray_workflow/Step1-QCraw/Outputs/extra_files_path_64/Microarray_Condition5.png]

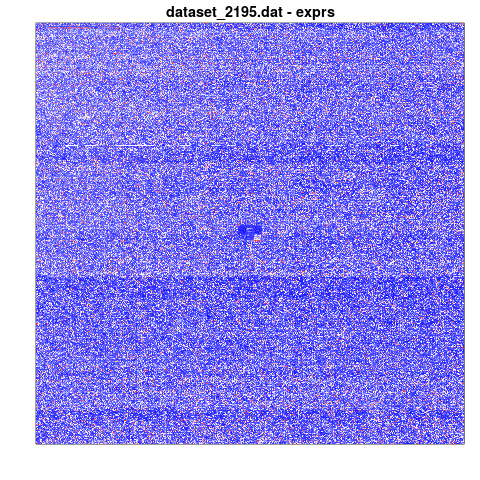

Supplement: Supplementary file 2 — Supplementary Information. [file 41598_2020_76769_MOESM2_ESM.zip › Tools_InputOutput_Parameters/MicroArray_workflow/Step1-QCraw/Outputs/extra_files_path_64/Microarray_Condition23.png]

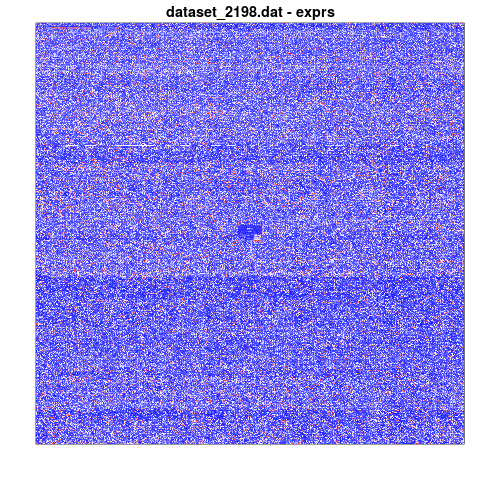

Supplement: Supplementary file 2 — Supplementary Information. [file 41598_2020_76769_MOESM2_ESM.zip › Tools_InputOutput_Parameters/MicroArray_workflow/Step1-QCraw/Outputs/extra_files_path_64/Microarray_Condition20.png]

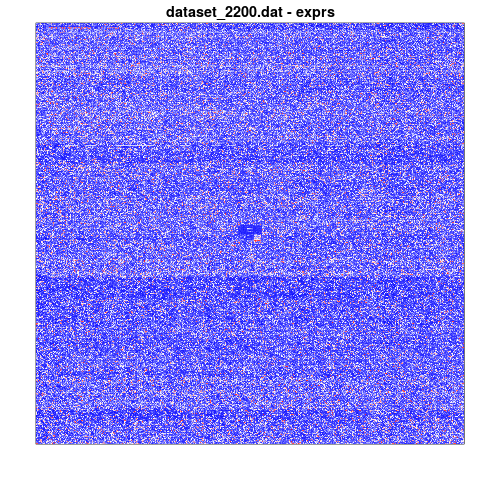

Supplement: Supplementary file 2 — Supplementary Information. [file 41598_2020_76769_MOESM2_ESM.zip › Tools_InputOutput_Parameters/MicroArray_workflow/Step1-QCraw/Outputs/extra_files_path_64/Microarray_Condition18.png]

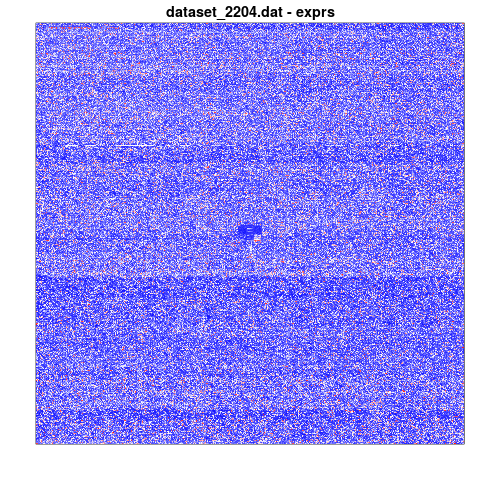

Supplement: Supplementary file 2 — Supplementary Information. [file 41598_2020_76769_MOESM2_ESM.zip › Tools_InputOutput_Parameters/MicroArray_workflow/Step1-QCraw/Outputs/extra_files_path_64/Microarray_Condition14.png]

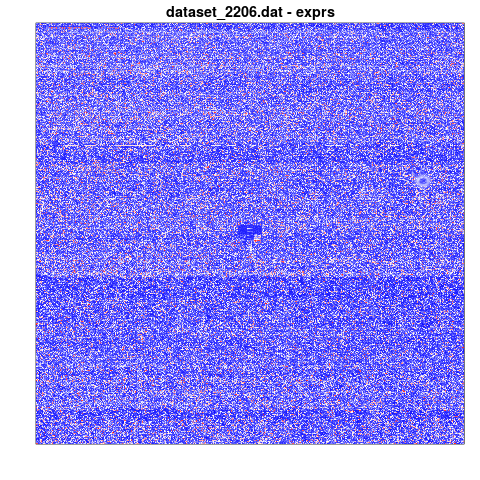

Supplement: Supplementary file 2 — Supplementary Information. [file 41598_2020_76769_MOESM2_ESM.zip › Tools_InputOutput_Parameters/MicroArray_workflow/Step1-QCraw/Outputs/extra_files_path_64/Microarray_Condition12.png]

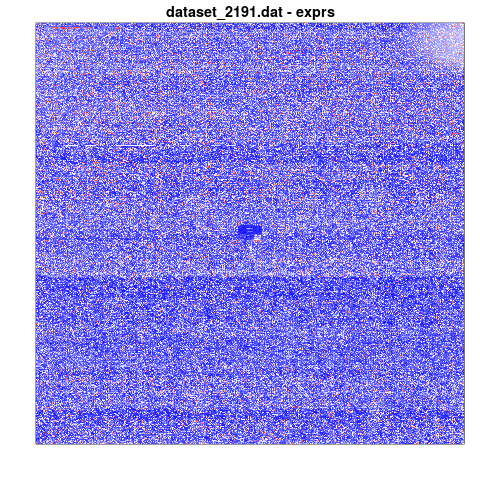

Supplement: Supplementary file 2 — Supplementary Information. [file 41598_2020_76769_MOESM2_ESM.zip › Tools_InputOutput_Parameters/MicroArray_workflow/Step1-QCraw/Outputs/extra_files_path_64/Microarray_Condition27.png]

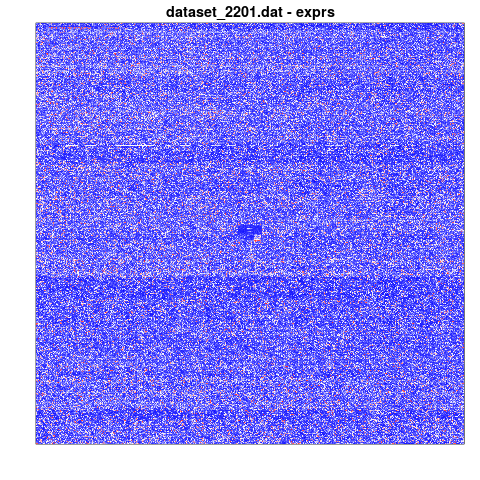

Supplement: Supplementary file 2 — Supplementary Information. [file 41598_2020_76769_MOESM2_ESM.zip › Tools_InputOutput_Parameters/MicroArray_workflow/Step1-QCraw/Outputs/extra_files_path_64/Microarray_Condition17.png]

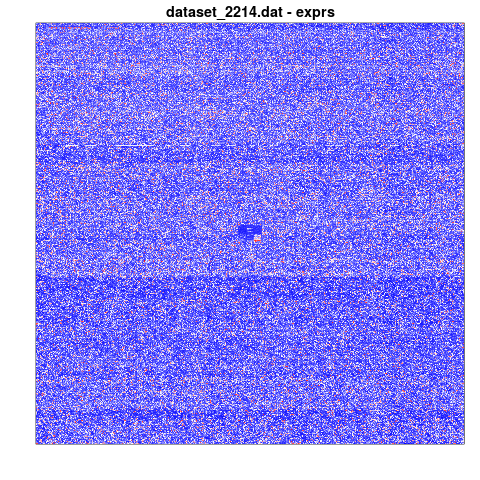

Supplement: Supplementary file 2 — Supplementary Information. [file 41598_2020_76769_MOESM2_ESM.zip › Tools_InputOutput_Parameters/MicroArray_workflow/Step1-QCraw/Outputs/extra_files_path_64/Microarray_Condition4.png]

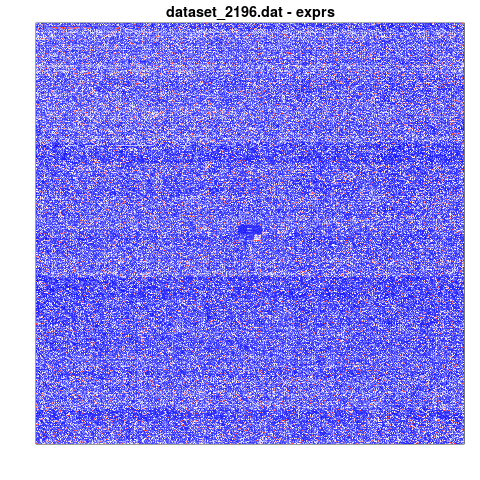

Supplement: Supplementary file 2 — Supplementary Information. [file 41598_2020_76769_MOESM2_ESM.zip › Tools_InputOutput_Parameters/MicroArray_workflow/Step1-QCraw/Outputs/extra_files_path_64/Microarray_Condition22.png]

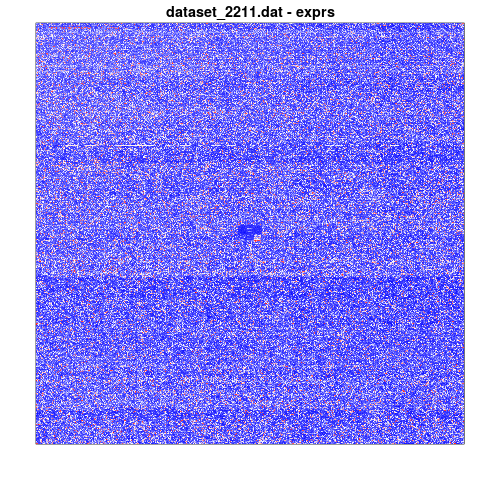

Supplement: Supplementary file 2 — Supplementary Information. [file 41598_2020_76769_MOESM2_ESM.zip › Tools_InputOutput_Parameters/MicroArray_workflow/Step1-QCraw/Outputs/extra_files_path_64/Microarray_Condition7.png]

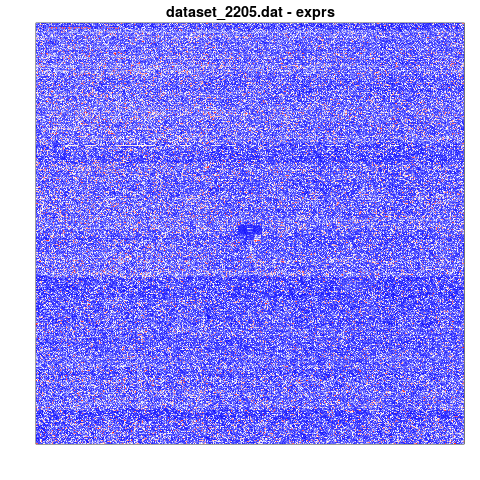

Supplement: Supplementary file 2 — Supplementary Information. [file 41598_2020_76769_MOESM2_ESM.zip › Tools_InputOutput_Parameters/MicroArray_workflow/Step1-QCraw/Outputs/extra_files_path_64/Microarray_Condition13.png]

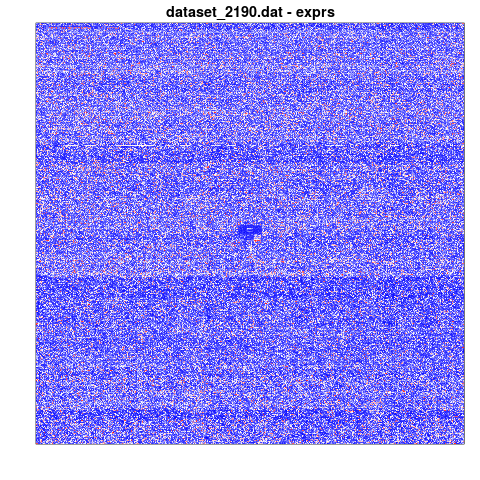

Supplement: Supplementary file 2 — Supplementary Information. [file 41598_2020_76769_MOESM2_ESM.zip › Tools_InputOutput_Parameters/MicroArray_workflow/Step1-QCraw/Outputs/extra_files_path_64/Microarray_Condition28.png]

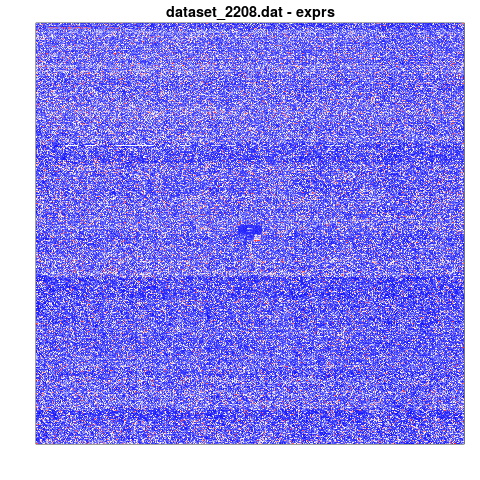

Supplement: Supplementary file 2 — Supplementary Information. [file 41598_2020_76769_MOESM2_ESM.zip › Tools_InputOutput_Parameters/MicroArray_workflow/Step1-QCraw/Outputs/extra_files_path_64/Microarray_Condition10.png]

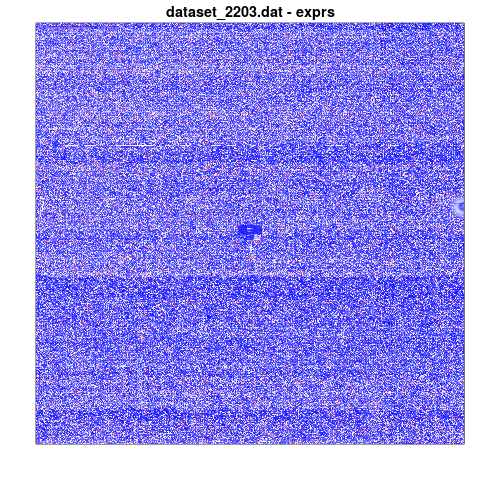

Supplement: Supplementary file 2 — Supplementary Information. [file 41598_2020_76769_MOESM2_ESM.zip › Tools_InputOutput_Parameters/MicroArray_workflow/Step1-QCraw/Outputs/extra_files_path_64/Microarray_Condition15.png]

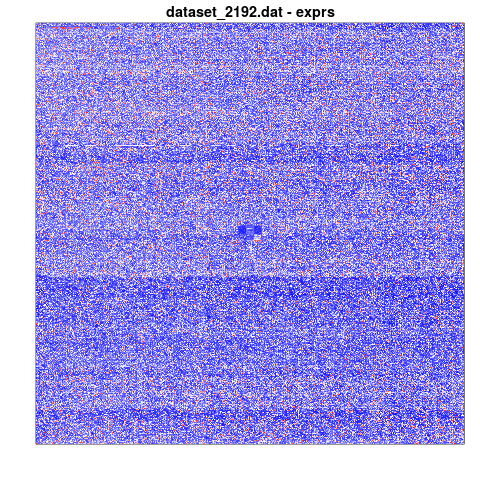

Supplement: Supplementary file 2 — Supplementary Information. [file 41598_2020_76769_MOESM2_ESM.zip › Tools_InputOutput_Parameters/MicroArray_workflow/Step1-QCraw/Outputs/extra_files_path_64/Microarray_Condition26.png]

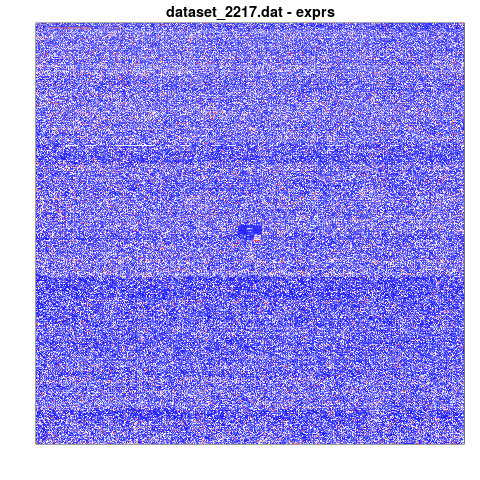

Supplement: Supplementary file 2 — Supplementary Information. [file 41598_2020_76769_MOESM2_ESM.zip › Tools_InputOutput_Parameters/MicroArray_workflow/Step1-QCraw/Outputs/extra_files_path_64/Microarray_Condition1.png]

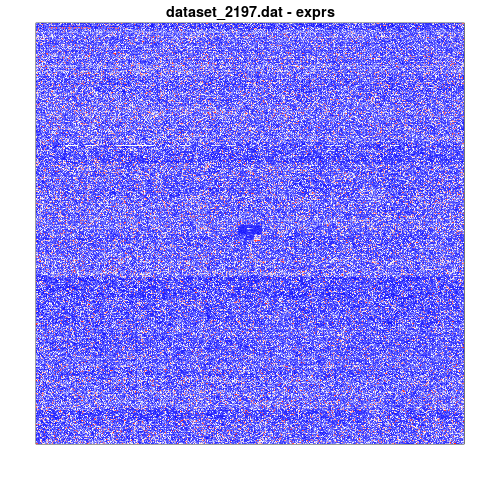

Supplement: Supplementary file 2 — Supplementary Information. [file 41598_2020_76769_MOESM2_ESM.zip › Tools_InputOutput_Parameters/MicroArray_workflow/Step1-QCraw/Outputs/extra_files_path_64/Microarray_Condition21.png]

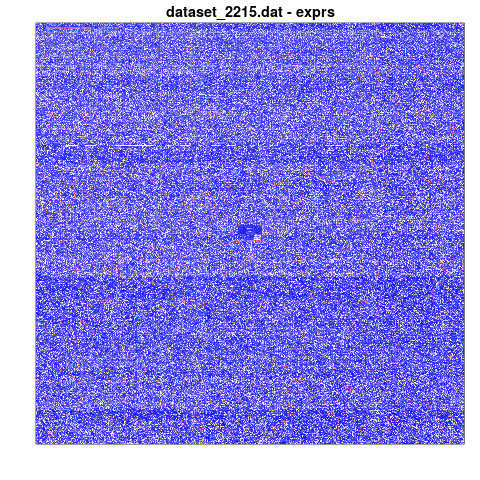

Supplement: Supplementary file 2 — Supplementary Information. [file 41598_2020_76769_MOESM2_ESM.zip › Tools_InputOutput_Parameters/MicroArray_workflow/Step1-QCraw/Outputs/extra_files_path_64/Microarray_Condition3.png]

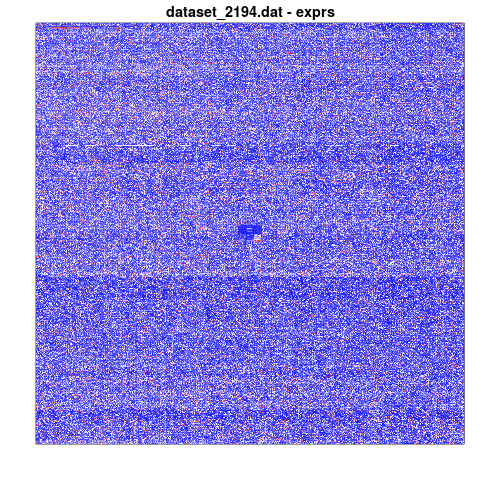

Supplement: Supplementary file 2 — Supplementary Information. [file 41598_2020_76769_MOESM2_ESM.zip › Tools_InputOutput_Parameters/MicroArray_workflow/Step1-QCraw/Outputs/extra_files_path_64/Microarray_Condition24.png]

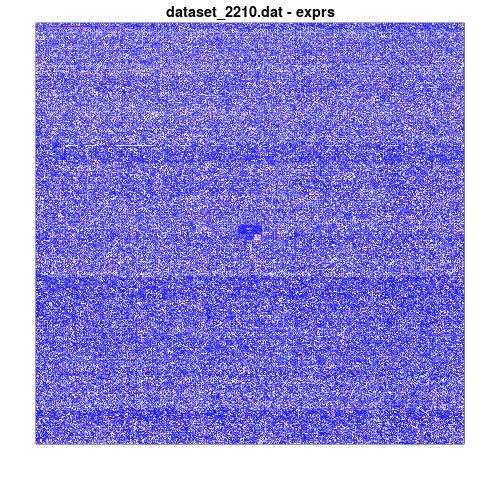

Supplement: Supplementary file 2 — Supplementary Information. [file 41598_2020_76769_MOESM2_ESM.zip › Tools_InputOutput_Parameters/MicroArray_workflow/Step1-QCraw/Outputs/extra_files_path_64/Microarray_Condition8.png]

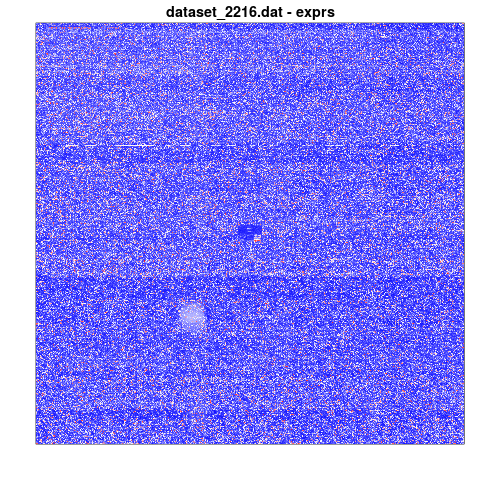

Supplement: Supplementary file 2 — Supplementary Information. [file 41598_2020_76769_MOESM2_ESM.zip › Tools_InputOutput_Parameters/MicroArray_workflow/Step1-QCraw/Outputs/extra_files_path_64/Microarray_Condition2.png]

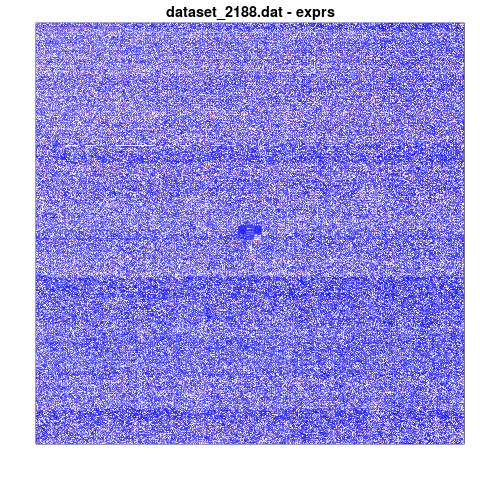

Supplement: Supplementary file 2 — Supplementary Information. [file 41598_2020_76769_MOESM2_ESM.zip › Tools_InputOutput_Parameters/MicroArray_workflow/Step1-QCraw/Outputs/extra_files_path_64/Microarray_Condition30.png]

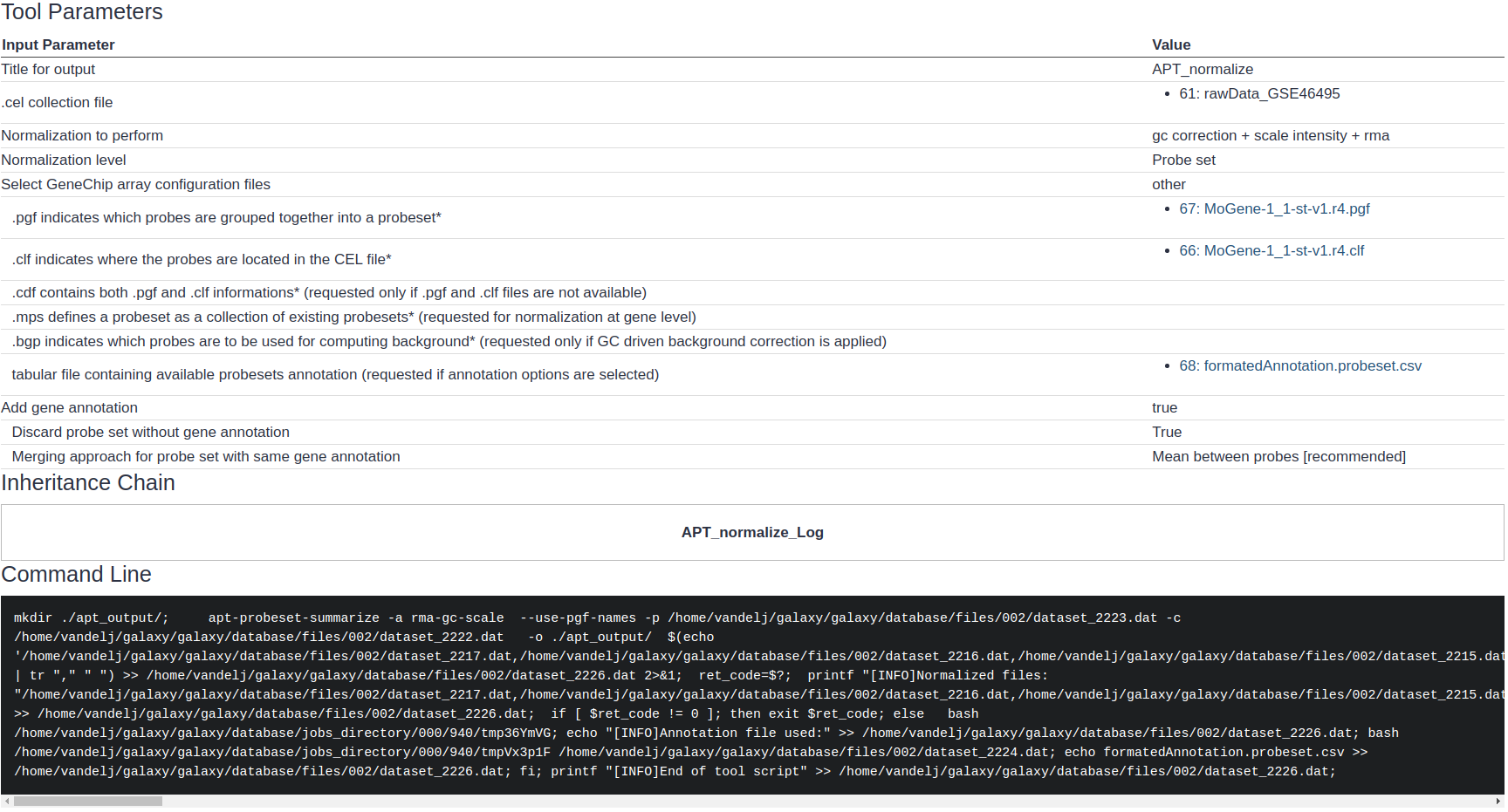

Supplement: Supplementary file 2 — Supplementary Information. [file 41598_2020_76769_MOESM2_ESM.zip › Tools_InputOutput_Parameters/MicroArray_workflow/Step2-Normalization/Step2-Normalization.toolParameters.png]

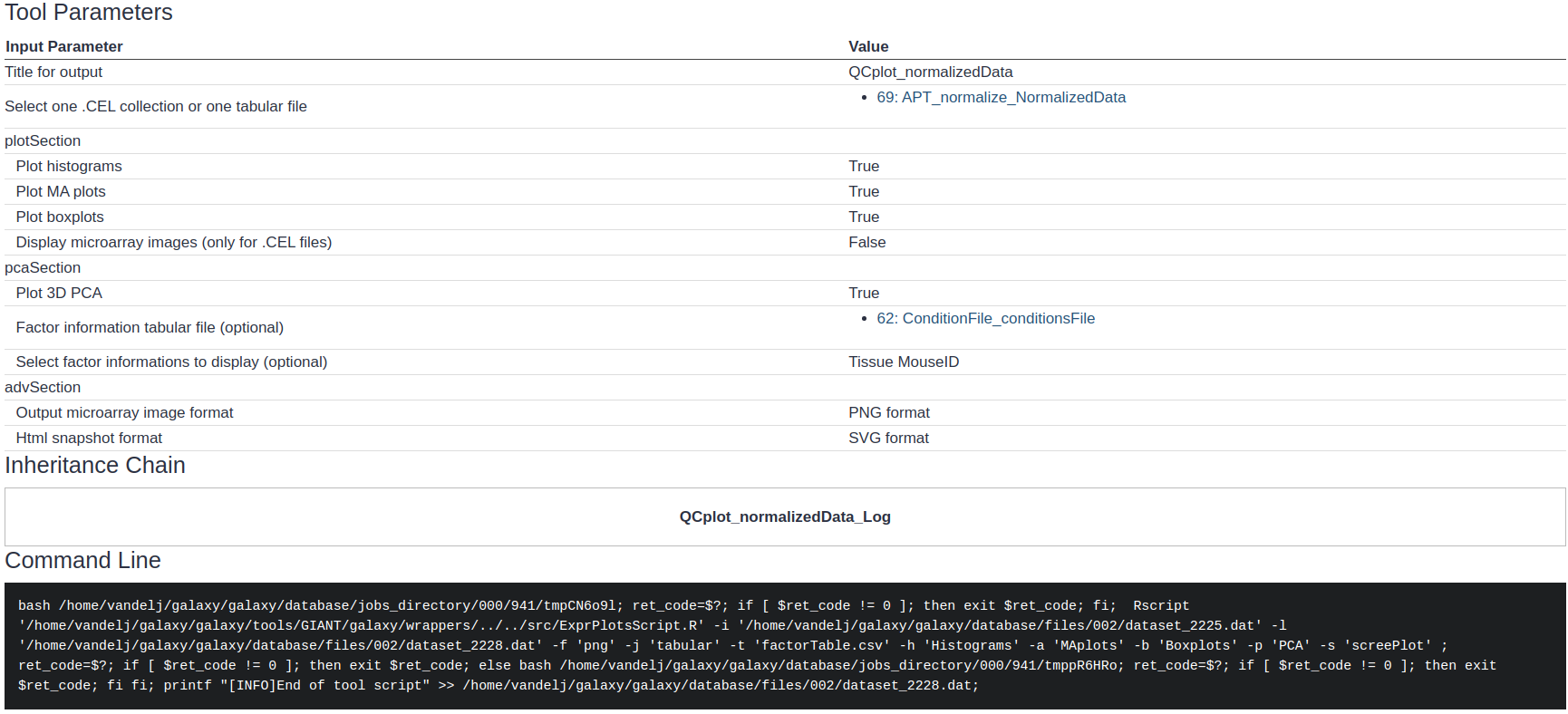

Supplement: Supplementary file 2 — Supplementary Information. [file 41598_2020_76769_MOESM2_ESM.zip › Tools_InputOutput_Parameters/MicroArray_workflow/Step3-QCnormalized/Step3-QCnormalized.toolParameters.png]
