# Supplementary material for: GIANT: galaxy-based tool for interactive analysis of transcriptomic data
Source: Sci Rep. 2020 Nov 16;10:19835. doi: 10.1038/s41598-020-76769-w (PMC7670435; doi:10.1038/s41598-020-76769-w)
Supplement: Supplementary file 2 — Supplementary Information. [file 41598_2020_76769_MOESM2_ESM.zip › Tools_InputOutput_Parameters/MicroArray_workflow/Step4-LIMMA/Outputs/extra_files_path_75/outputLIMMA.html]

| Gene | Info | FastedVsFed | | | | | FastedVsFed-Liver | | | | | Diet\_Fasted:Tissue\_AdiposeTis | | | | | Diet\_Fasted:Tissue\_SkeletalMus | | | | |  |
| --- | --- | --- | --- | --- | --- | --- | --- | --- | --- | --- | --- | --- | --- | --- | --- | --- | --- | --- | --- | --- | --- | --- |
|  |  | p-val | FDR.p-val | FC | log2(FC) | t-stat | p-val | FDR.p-val | FC | log2(FC) | t-stat | p-val | FDR.p-val | FC | log2(FC) | t-stat | p-val | FDR.p-val | FC | log2(FC) | t-stat |  |
| Gene | Info | p-val | FDR.p-val | FC | log2(FC) | t-stat | p-val | FDR.p-val | FC | log2(FC) | t-stat | p-val | FDR.p-val | FC | log2(FC) | t-stat | p-val | FDR.p-val | FC | log2(FC) | t-stat |  |
| --- | --- | --- | --- | --- | --- | --- | --- | --- | --- | --- | --- | --- | --- | --- | --- | --- | --- | --- | --- | --- | --- | --- |
| Serpina7 | serine (or cysteine) peptidase | 1.968e-08 | 1.406e-05 | 0.2867 | -1.802 | -8.957 | 1.092e-14 | 1.404e-10 | 0.03679 | -4.765 | -20.02 | 1.606e-14 | 6.885e-11 | 20.01 | 4.323 | 19.61 | 5.683e-15 | 2.924e-11 | 23.67 | 4.565 | 20.71 |  |
| --- | --- | --- | --- | --- | --- | --- | --- | --- | --- | --- | --- | --- | --- | --- | --- | --- | --- | --- | --- | --- | --- | --- |
| Gm15998 | predicted gene 15998 | 4.259e-07 | 0.0001355 | 0.3433 | -1.542 | -7.348 | 1.943e-14 | 1.666e-10 | 0.03532 | -4.823 | -19.42 | 2.934e-15 | 2.17e-11 | 30.49 | 4.93 | 21.44 | 3.14e-15 | 2.693e-11 | 30.12 | 4.913 | 21.36 |  |
| Pcsk9 | proprotein convertase subtilis | 4.537e-07 | 0.0001389 | 0.4025 | -1.313 | -7.316 | 6.793e-13 | 3.472e-09 | 0.09384 | -3.414 | -16.08 | 2.667e-13 | 6.237e-10 | 10.01 | 3.323 | 16.9 | 2.024e-12 | 2.264e-09 | 7.888 | 2.98 | 15.16 |  |
| Hsd3b5 | hydroxy-delta-5-steroid dehydr | 8.454e-07 | 0.0002219 | 0.1843 | -2.44 | -7.011 | 8.1e-13 | 3.472e-09 | 0.01062 | -6.557 | -15.93 | 3.926e-13 | 7.769e-10 | 79.39 | 6.311 | 16.56 | 8.879e-13 | 1.202e-09 | 65.82 | 6.04 | 15.85 |  |
| Zbtb16 | zinc finger and BTB domain con | 3.152e-10 | 8.109e-07 | 3.317 | 1.73 | 11.45 | 7.096e-13 | 3.472e-09 | 7.299 | 2.868 | 16.04 | 2.137e-10 | 9.014e-08 | 0.2611 | -1.938 | -11.71 | 2.12e-08 | 2.543e-06 | 0.3595 | -1.476 | -8.916 |  |
| Usp2 | ubiquitin specific peptidase 2 | 4.326e-05 | 0.003302 | 1.8 | 0.848 | 5.204 | 1.855e-11 | 6.816e-08 | 6.015 | 2.589 | 13.43 | 1.287e-10 | 6.248e-08 | 0.2252 | -2.15 | -12.05 | 1.907e-13 | 3.773e-10 | 0.1189 | -3.072 | -17.21 |  |
| Cyp17a1 | cytochrome P450, family 17, su | 2.225e-05 | 0.00216 | 3.265 | 1.707 | 5.497 | 9.986e-11 | 2.335e-07 | 22.49 | 4.491 | 12.22 | 2.085e-10 | 8.937e-08 | 0.06303 | -3.988 | -11.72 | 4.229e-11 | 2.034e-08 | 0.04859 | -4.363 | -12.83 |  |
| Rgn | regucalcin | 8.306e-05 | 0.004882 | 0.6653 | -0.588 | -4.919 | 1.336e-10 | 2.741e-07 | 0.3077 | -1.7 | -12.02 | 3.208e-11 | 2.253e-08 | 3.261 | 1.705 | 13.02 | 7.079e-11 | 2.985e-08 | 3.099 | 1.632 | 12.46 |  |
| Slc16a5 | solute carrier family 16 (mono | 0.0001427 | 0.006795 | 2.059 | 1.042 | 4.685 | 1.851e-10 | 3.174e-07 | 8.613 | 3.106 | 11.8 | 1.26e-11 | 1.296e-08 | 0.09858 | -3.343 | -13.72 | 2.171e-10 | 6.812e-08 | 0.1387 | -2.85 | -11.7 |  |
| Pfkfb2 | 6-phosphofructo-2-kinase/fruct | 0.0002808 | 0.01028 | 1.485 | 0.5701 | 4.394 | 1.789e-10 | 3.174e-07 | 3.519 | 1.815 | 11.82 | 1.246e-10 | 6.248e-08 | 0.3045 | -1.715 | -12.07 | 6.596e-12 | 5.355e-09 | 0.2466 | -2.02 | -14.21 |  |
| Mfsd2a | major facilitator superfamily | 0.0002274 | 0.009098 | 2.551 | 1.351 | 4.484 | 3.438e-10 | 5.527e-07 | 16.69 | 4.061 | 11.39 | 1.829e-10 | 8.402e-08 | 0.06711 | -3.897 | -11.81 | 4.245e-11 | 2.034e-08 | 0.05323 | -4.232 | -12.82 |  |
| Hsd3b4 | hydroxy-delta-5-steroid dehydr | 0.0001735 | 0.007748 | 0.4415 | -1.18 | -4.601 | 4.839e-10 | 6.224e-07 | 0.09551 | -3.388 | -11.17 | 2.287e-10 | 9.491e-08 | 9.678 | 3.275 | 11.66 | 1.521e-10 | 5.146e-08 | 10.21 | 3.352 | 11.93 |  |
| Apex2 | apurinic/apyrimidinic endonucl | 0.0002739 | 0.01015 | 1.683 | 0.7514 | 4.405 | 3.876e-10 | 5.538e-07 | 4.869 | 2.284 | 11.31 | 1.726e-11 | 1.587e-08 | 0.1745 | -2.519 | -13.48 | 5.248e-10 | 1.452e-07 | 0.2369 | -2.078 | -11.12 |  |
| Clec2h | C-type lectin domain family 2, | 0.0001321 | 0.006446 | 0.349 | -1.519 | -4.718 | 1.869e-09 | 1.849e-06 | 0.06554 | -3.932 | -10.32 | 1.201e-09 | 3.089e-07 | 13.32 | 3.736 | 10.6 | 3.56e-09 | 6.643e-07 | 11.34 | 3.504 | 9.937 |  |
| Cyp4a31 | cytochrome P450, family 4, sub | 0.0005201 | 0.01508 | 1.9 | 0.9261 | 4.13 | 4.203e-10 | 5.69e-07 | 7.932 | 2.988 | 11.26 | 2.727e-11 | 2.192e-08 | 0.1067 | -3.228 | -13.14 | 1.308e-10 | 4.608e-08 | 0.1288 | -2.957 | -12.04 |  |
| Adgrf1 | adhesion G protein-coupled rec | 0.0001222 | 0.006213 | 0.4462 | -1.164 | -4.752 | 2.503e-09 | 2.038e-06 | 0.1301 | -2.942 | -10.15 | 3.205e-09 | 6.391e-07 | 6.426 | 2.684 | 9.999 | 4.002e-09 | 7.199e-07 | 6.27 | 2.649 | 9.868 |  |
| Rgs16 | regulator of G-protein signali | 7.034e-05 | 0.004456 | 4.203 | 2.071 | 4.991 | 2.907e-09 | 2.199e-06 | 30.67 | 4.939 | 10.06 | 7.664e-09 | 1.248e-06 | 0.05027 | -4.314 | -9.49 | 8.479e-09 | 1.275e-06 | 0.0512 | -4.288 | -9.432 |  |
| Map3k6 | mitogen-activated protein kina | 2.731e-11 | 1.405e-07 | 3.792 | 1.923 | 13.14 | 3.777e-05 | 0.002927 | 1.881 | 0.9113 | 5.263 | 1.105e-07 | 9.121e-06 | 2.441 | 1.288 | 8.032 | 7.358e-10 | 1.931e-07 | 3.358 | 1.748 | 10.9 |  |
| Nudt7 | nudix (nucleoside diphosphate | 0.0004181 | 0.01316 | 0.5145 | -0.9587 | -4.223 | 2.204e-09 | 1.949e-06 | 0.1491 | -2.746 | -10.22 | 2.68e-09 | 5.651e-07 | 5.708 | 2.513 | 10.11 | 3.112e-10 | 9.309e-08 | 7.204 | 2.849 | 11.46 |  |
| Cdkn1a | cyclin-dependent kinase inhibi | 2.06e-13 | 5.3e-09 | 14.02 | 3.81 | 17.14 | 9.415e-15 | 1.404e-10 | 39.55 | 5.306 | 20.17 | 6.675e-11 | 3.902e-08 | 0.1212 | -3.044 | -12.5 | 8.517e-06 | 0.0002598 | 0.3676 | -1.444 | -5.929 |  |
| Mvd | mevalonate (diphospho) decarbo | 6.495e-05 | 0.00429 | 0.4423 | -1.177 | -5.026 | 2.273e-09 | 1.949e-06 | 0.1409 | -2.828 | -10.21 | 2.639e-07 | 1.815e-05 | 3.854 | 1.946 | 7.587 | 2.099e-10 | 6.666e-08 | 8.032 | 3.006 | 11.72 |  |
| Hmgcr | 3-hydroxy-3-methylglutaryl-Coe | 0.001405 | 0.02816 | 0.7108 | -0.4924 | -3.705 | 1.23e-09 | 1.439e-06 | 0.3155 | -1.664 | -10.58 | 9.69e-11 | 5.082e-08 | 3.441 | 1.783 | 12.24 | 1.605e-10 | 5.295e-08 | 3.323 | 1.732 | 11.9 |  |
| Gstm3 | glutathione S-transferase, mu | 0.0003867 | 0.01255 | 0.439 | -1.188 | -4.257 | 6.581e-09 | 4.232e-06 | 0.1117 | -3.162 | -9.577 | 1.142e-08 | 1.685e-06 | 7.115 | 2.831 | 9.262 | 2.645e-09 | 5.154e-07 | 8.523 | 3.091 | 10.11 |  |
| Adh4 | alcohol dehydrogenase 4 (class | 7.392e-05 | 0.004593 | 0.6141 | -0.7035 | -4.97 | 8.693e-09 | 5.324e-06 | 0.3351 | -1.577 | -9.417 | 6.636e-08 | 6.276e-06 | 2.44 | 1.287 | 8.299 | 3.743e-08 | 4.045e-06 | 2.522 | 1.334 | 8.606 |  |
| Keg1 | kidney expressed gene 1 | 0.0005759 | 0.01603 | 0.4924 | -1.022 | -4.087 | 9.273e-09 | 5.327e-06 | 0.146 | -2.776 | -9.38 | 2.078e-08 | 2.646e-06 | 5.449 | 2.446 | 8.927 | 2.019e-09 | 4.155e-07 | 7.041 | 2.816 | 10.28 |  |
| Lss | lanosterol synthase | 1.025e-07 | 4.794e-05 | 0.3933 | -1.346 | -8.071 | 2.245e-11 | 7.219e-08 | 0.1624 | -2.622 | -13.29 | 1.345e-05 | 0.0003668 | 2.064 | 1.046 | 5.722 | 1.87e-12 | 2.186e-09 | 6.877 | 2.782 | 15.22 |  |
| C330021F23Rik | RIKEN cDNA C330021F23 gene | 0.0009011 | 0.02121 | 0.5104 | -0.9704 | -3.895 | 9.319e-09 | 5.327e-06 | 0.1472 | -2.764 | -9.378 | 2.067e-09 | 4.746e-07 | 6.97 | 2.801 | 10.26 | 8.109e-09 | 1.242e-06 | 5.984 | 2.581 | 9.457 |  |
| Car3 | carbonic anhydrase 3 | 8.091e-05 | 0.004801 | 0.5928 | -0.7543 | -4.931 | 9.584e-09 | 5.359e-06 | 0.3089 | -1.695 | -9.362 | 1.058e-08 | 1.593e-06 | 2.947 | 1.559 | 9.305 | 2.974e-07 | 1.848e-05 | 2.397 | 1.261 | 7.527 |  |
| Gm15441 | predicted gene 15441 | 0.002451 | 0.03893 | 1.89 | 0.9181 | 3.465 | 1.777e-09 | 1.828e-06 | 9.491 | 3.247 | 10.35 | 8.548e-10 | 2.527e-07 | 0.1137 | -3.137 | -10.81 | 2.332e-11 | 1.428e-08 | 0.06943 | -3.848 | -13.26 |  |
| Aqp8 | aquaporin 8 | 0.001541 | 0.02972 | 0.5067 | -0.9807 | -3.665 | 1.074e-08 | 5.61e-06 | 0.13 | -2.944 | -9.297 | 2.463e-09 | 5.294e-07 | 7.876 | 2.977 | 10.16 | 3.589e-09 | 6.643e-07 | 7.525 | 2.912 | 9.932 |  |
| Id3 | inhibitor of DNA binding 3 | 0.0004675 | 0.01415 | 0.4813 | -1.055 | -4.176 | 1.09e-08 | 5.61e-06 | 0.146 | -2.776 | -9.288 | 1.957e-07 | 1.426e-05 | 4.412 | 2.141 | 7.738 | 7.116e-10 | 1.907e-07 | 8.128 | 3.023 | 10.92 |  |
| St3gal5 | ST3 beta-galactoside alpha-2,3 | 0.000299 | 0.01071 | 1.804 | 0.8511 | 4.367 | 1.637e-08 | 7.655e-06 | 4.255 | 2.089 | 9.06 | 3.072e-09 | 6.271e-07 | 0.2269 | -2.14 | -10.02 | 4.05e-07 | 2.395e-05 | 0.3359 | -1.574 | -7.373 |  |
| Chrna4 | cholinergic receptor, nicotini | 0.0004875 | 0.01446 | 0.4376 | -1.192 | -4.158 | 2.462e-08 | 1.005e-05 | 0.1253 | -2.997 | -8.834 | 3.297e-08 | 3.672e-06 | 6.61 | 2.725 | 8.674 | 4.045e-08 | 4.315e-06 | 6.452 | 2.69 | 8.564 |  |
| Galnt15 | polypeptide N-acetylgalactosam | 2.221e-10 | 7.141e-07 | 3.335 | 1.738 | 11.68 | 0.0001336 | 0.007234 | 1.777 | 0.8298 | 4.713 | 3.176e-09 | 6.383e-07 | 3.097 | 1.631 | 10 | 1.587e-06 | 7.037e-05 | 2.134 | 1.094 | 6.709 |  |
| Tcp11l2 | t-complex 11 (mouse) like 2 | 8.017e-07 | 0.0002171 | 2.006 | 1.005 | 7.037 | 0.01435 | 0.1603 | 0.7305 | -0.453 | -2.682 | 6.054e-10 | 1.923e-07 | 3.305 | 1.724 | 11.03 | 2.573e-13 | 4.412e-10 | 6.271 | 2.649 | 16.94 |  |
| 5830473C10Rik | NA | 0.0002746 | 0.01015 | 0.6063 | -0.7218 | -4.404 | 2.905e-08 | 1.132e-05 | 0.3087 | -1.696 | -8.743 | 4.286e-08 | 4.428e-06 | 2.892 | 1.532 | 8.533 | 1.953e-07 | 1.365e-05 | 2.62 | 1.39 | 7.739 |  |
| Aplnr | apelin receptor | 9.887e-11 | 3.633e-07 | 0.3188 | -1.649 | -12.23 | 0.04666 | 0.3005 | 1.264 | 0.3385 | 2.121 | 1.606e-15 | 2.065e-11 | 0.1037 | -3.269 | -22.12 | 6.379e-14 | 2.121e-10 | 0.1544 | -2.695 | -18.24 |  |
| Flcn | folliculin | 0.001598 | 0.03038 | 1.358 | 0.441 | 3.649 | 4.471e-05 | 0.003249 | 0.5979 | -0.742 | -5.189 | 7.206e-13 | 1.324e-09 | 4.352 | 2.122 | 16.03 | 8.906e-10 | 2.182e-07 | 2.69 | 1.427 | 10.78 |  |
| Rdh11 | retinol dehydrogenase 11 | 0.004053 | 0.05265 | 0.6246 | -0.679 | -3.246 | 6.818e-09 | 4.277e-06 | 0.1941 | -2.365 | -9.557 | 5.134e-09 | 9.501e-07 | 4.683 | 2.228 | 9.722 | 8.214e-11 | 3.292e-08 | 7.118 | 2.831 | 12.36 |  |
| Cyp2c55 | cytochrome P450, family 2, sub | 0.001619 | 0.03062 | 0.4465 | -1.163 | -3.644 | 3.078e-08 | 1.171e-05 | 0.1022 | -3.291 | -8.712 | 1.08e-08 | 1.615e-06 | 9.516 | 3.25 | 9.294 | 1.968e-08 | 2.399e-06 | 8.77 | 3.133 | 8.957 |  |
| Map2k6 | mitogen-activated protein kina | 0.001812 | 0.03287 | 0.6057 | -0.7233 | -3.595 | 9.592e-05 | 0.005765 | 2.229 | 1.156 | 4.857 | 1.896e-10 | 8.556e-08 | 0.1652 | -2.598 | -11.79 | 1.133e-11 | 8.569e-09 | 0.1215 | -3.041 | -13.8 |  |
| Asl | argininosuccinate lyase | 0.009784 | 0.09138 | 1.231 | 0.2993 | 2.856 | 2.535e-09 | 2.038e-06 | 2.391 | 1.258 | 10.14 | 3.416e-11 | 2.253e-08 | 0.3559 | -1.49 | -12.98 | 1.269e-10 | 4.535e-08 | 0.383 | -1.384 | -12.06 |  |
| Dpy19l3 | dpy-19-like 3 (C. elegans) | 4.68e-05 | 0.00347 | 0.4116 | -1.281 | -5.169 | 1.84e-08 | 8.061e-06 | 0.1608 | -2.637 | -8.995 | 2.952e-06 | 0.0001128 | 3.344 | 1.742 | 6.417 | 3.975e-08 | 4.261e-06 | 5.018 | 2.327 | 8.573 |  |
| Pmvk | phosphomevalonate kinase | 3.942e-05 | 0.003101 | 0.4512 | -1.148 | -5.245 | 2.015e-08 | 8.497e-06 | 0.2007 | -2.317 | -8.944 | 1.208e-06 | 5.725e-05 | 3.117 | 1.64 | 6.839 | 1.8e-07 | 1.276e-05 | 3.645 | 1.866 | 7.781 |  |
| Aacs | acetoacetyl-CoA synthetase | 0.003645 | 0.04914 | 0.4722 | -1.082 | -3.292 | 2.175e-08 | 9.026e-06 | 0.09067 | -3.463 | -8.902 | 3.361e-09 | 6.65e-07 | 12.05 | 3.591 | 9.971 | 4.066e-09 | 7.252e-07 | 11.72 | 3.551 | 9.858 |  |
| Prok1 | prokineticin 1 | 0.002809 | 0.04223 | 0.716 | -0.482 | -3.406 | 3.43e-08 | 1.279e-05 | 0.3663 | -1.449 | -8.653 | 7.084e-09 | 1.176e-06 | 2.786 | 1.478 | 9.535 | 1.326e-08 | 1.758e-06 | 2.681 | 1.423 | 9.178 |  |
| Asic5 | acid-sensing (proton-gated) io | 0.00694 | 0.07458 | 1.434 | 0.5203 | 3.009 | 1.983e-08 | 8.497e-06 | 3.56 | 1.832 | 8.953 | 1.77e-09 | 4.178e-07 | 0.2567 | -1.962 | -10.36 | 1.612e-09 | 3.427e-07 | 0.2548 | -1.973 | -10.41 |  |
| Sdsl | serine dehydratase-like | 0.021 | 0.142 | 1.271 | 0.3461 | 2.505 | 1.502e-09 | 1.68e-06 | 3.269 | 1.709 | 10.46 | 3.938e-12 | 4.824e-09 | 0.2158 | -2.212 | -14.62 | 7.66e-11 | 3.158e-08 | 0.2722 | -1.877 | -12.41 |  |
| Gabbr2 | gamma-aminobutyric acid (GABA) | 0.0002108 | 0.008731 | 0.6316 | -0.6629 | -4.517 | 5.475e-08 | 1.984e-05 | 0.3638 | -1.459 | -8.402 | 5.481e-07 | 3.126e-05 | 2.236 | 1.161 | 7.223 | 2.421e-07 | 1.622e-05 | 2.34 | 1.227 | 7.63 |  |
| Tnfaip2 | tumor necrosis factor, alpha-i | 3.192e-06 | 0.0005703 | 0.3113 | -1.684 | -6.38 | 6.942e-06 | 0.0008544 | 0.2717 | -1.88 | -6.022 | 1.014e-07 | 8.579e-06 | 5.045 | 2.335 | 8.077 | 6.718e-06 | 0.0002177 | 0.2983 | -1.745 | -6.037 |  |
| As3mt | arsenic (+3 oxidation state) m | 0.001069 | 0.02382 | 1.571 | 0.6518 | 3.822 | 6.795e-08 | 2.3e-05 | 3.187 | 1.672 | 8.287 | 5.185e-07 | 3.024e-05 | 0.391 | -1.355 | -7.25 | 1.428e-08 | 1.883e-06 | 0.3063 | -1.707 | -9.136 |  |
| Slc22a5 | solute carrier family 22 (orga | 3.096e-05 | 0.002719 | 1.82 | 0.8636 | 5.351 | 8.93e-09 | 5.327e-06 | 3.471 | 1.795 | 9.402 | 3.552e-05 | 0.0007776 | 0.5229 | -0.9353 | -5.29 | 1.355e-09 | 3.057e-07 | 0.2755 | -1.86 | -10.52 |  |
| Acacb | acetyl-Coenzyme A carboxylase | 0.009387 | 0.08895 | 1.559 | 0.6402 | 2.874 | 9.303e-06 | 0.00104 | 0.3411 | -1.552 | -5.889 | 1.91e-13 | 5.459e-10 | 18.36 | 4.198 | 17.21 | 4.929e-09 | 8.326e-07 | 5.198 | 2.378 | 9.745 |  |
| Defb1 | defensin beta 1 | 0.001728 | 0.0319 | 1.714 | 0.7775 | 3.615 | 1.072e-07 | 3.362e-05 | 4.135 | 2.048 | 8.048 | 7.286e-08 | 6.694e-06 | 0.26 | -1.944 | -8.25 | 1.354e-07 | 1.051e-05 | 0.2741 | -1.867 | -7.927 |  |
| Cyp2c70 | cytochrome P450, family 2, sub | 0.01031 | 0.09412 | 0.5894 | -0.7626 | -2.832 | 5.821e-08 | 1.996e-05 | 0.1575 | -2.666 | -8.369 | 6.13e-09 | 1.065e-06 | 7.146 | 2.837 | 9.618 | 4.939e-09 | 8.326e-07 | 7.332 | 2.874 | 9.744 |  |
| 2810416G20Rik | NA | 0.003136 | 0.04534 | 1.689 | 0.7562 | 3.358 | 8.798e-08 | 2.865e-05 | 4.507 | 2.172 | 8.151 | 1.899e-07 | 1.4e-05 | 0.2656 | -1.913 | -7.754 | 8.024e-09 | 1.242e-06 | 0.1982 | -2.335 | -9.463 |  |
| Abca8a | ATP-binding cassette, sub-fami | 0.0001889 | 0.008099 | 0.4546 | -1.137 | -4.564 | 8.539e-08 | 2.816e-05 | 0.1885 | -2.408 | -8.167 | 4.818e-07 | 2.856e-05 | 3.969 | 1.989 | 7.287 | 1.698e-06 | 7.44e-05 | 3.537 | 1.823 | 6.677 |  |
| Cyp39a1 | cytochrome P450, family 39, su | 0.001666 | 0.03121 | 1.847 | 0.8851 | 3.631 | 5.722e-08 | 1.989e-05 | 5.338 | 2.416 | 8.378 | 1.518e-06 | 6.723e-05 | 0.2878 | -1.797 | -6.73 | 1.463e-09 | 3.217e-07 | 0.1439 | -2.797 | -10.47 |  |
| Sqle | squalene epoxidase | 5.211e-05 | 0.00371 | 0.3459 | -1.531 | -5.122 | 3.788e-08 | 1.392e-05 | 0.1214 | -3.042 | -8.599 | 1.622e-05 | 0.0004229 | 3.596 | 1.847 | 5.638 | 8.032e-08 | 7.149e-06 | 6.432 | 2.685 | 8.199 |  |
| Hes6 | hairy and enhancer of split 6 | 2.853e-06 | 0.0005396 | 0.4253 | -1.234 | -6.432 | 1.081e-08 | 5.61e-06 | 0.2319 | -2.109 | -9.293 | 1.046e-06 | 5.149e-05 | 2.735 | 1.451 | 6.909 | 1.814e-05 | 0.0004694 | 2.256 | 1.174 | 5.588 |  |
| Mvk | mevalonate kinase | 1.142e-08 | 9.789e-06 | 0.368 | -1.442 | -9.262 | 1.385e-10 | 2.741e-07 | 0.2161 | -2.21 | -12 | 0.0003388 | 0.004421 | 1.665 | 0.7357 | 4.314 | 1.282e-08 | 1.735e-06 | 2.966 | 1.569 | 9.197 |  |
| Pdk4 | pyruvate dehydrogenase kinase, | 1.749e-08 | 1.328e-05 | 3.788 | 1.921 | 9.023 | 0.09038 | 0.4051 | 0.7329 | -0.4483 | -1.779 | 3.375e-15 | 2.17e-11 | 31.22 | 4.964 | 21.28 | 1.29e-08 | 1.738e-06 | 4.421 | 2.144 | 9.193 |  |
| Col15a1 | collagen, type XV, alpha 1 | 5.464e-09 | 6.389e-06 | 0.1909 | -2.389 | -9.685 | 0.009709 | 0.1289 | 0.5608 | -0.8344 | -2.859 | 8.707e-10 | 2.545e-07 | 0.1324 | -2.917 | -10.8 | 2.669e-06 | 0.000104 | 0.298 | -1.746 | -6.464 |  |
| BC029214 | NA | 0.01098 | 0.0977 | 0.6775 | -0.5616 | -2.804 | 1.376e-07 | 4.165e-05 | 0.2723 | -1.877 | -7.919 | 3.537e-08 | 3.822e-06 | 3.72 | 1.895 | 8.636 | 9.869e-09 | 1.426e-06 | 4.143 | 2.051 | 9.345 |  |
| Hsd3b2 | hydroxy-delta-5-steroid dehydr | 0.001013 | 0.02294 | 0.6036 | -0.7284 | -3.845 | 1.875e-07 | 5.42e-05 | 0.2995 | -1.74 | -7.76 | 2.692e-07 | 1.847e-05 | 2.974 | 1.572 | 7.577 | 7.987e-07 | 4.052e-05 | 2.753 | 1.461 | 7.039 |  |
| Ern1 | endoplasmic reticulum (ER) to | 0.006232 | 0.07013 | 1.33 | 0.4117 | 3.057 | 0.0002231 | 0.01043 | 0.6088 | -0.7161 | -4.493 | 5.73e-09 | 1.017e-06 | 2.685 | 1.425 | 9.658 | 2.287e-11 | 1.428e-08 | 3.886 | 1.958 | 13.27 |  |
| Efna1 | ephrin A1 | 8.467e-06 | 0.001152 | 0.515 | -0.9574 | -5.931 | 2.515e-08 | 1.006e-05 | 0.311 | -1.685 | -8.822 | 3.254e-06 | 0.0001217 | 2.183 | 1.126 | 6.371 | 7.733e-06 | 0.0002429 | 2.079 | 1.056 | 5.972 |  |
| Ddit4 | DNA-damage-inducible transcrip | 2.516e-11 | 1.405e-07 | 41.39 | 5.371 | 13.2 | 6.18e-07 | 0.0001359 | 10.92 | 3.449 | 7.164 | 0.0002563 | 0.003558 | 3.934 | 1.976 | 4.433 | 4.503e-08 | 4.728e-06 | 13.84 | 3.791 | 8.506 |  |
| Fabp5 | fatty acid binding protein 5, | 0.003314 | 0.04685 | 0.4807 | -1.057 | -3.334 | 1.401e-07 | 4.191e-05 | 0.128 | -2.966 | -7.909 | 5.799e-09 | 1.022e-06 | 10.2 | 3.351 | 9.651 | 1.185e-06 | 5.634e-05 | 5.198 | 2.378 | 6.849 |  |
| Plxna1 | plexin A1 | 1.774e-08 | 1.328e-05 | 0.4759 | -1.071 | -9.015 | 0.05527 | 0.3242 | 0.8201 | -0.2862 | -2.036 | 6.035e-08 | 5.858e-06 | 0.4708 | -1.087 | -8.35 | 4.952e-09 | 8.326e-07 | 0.4152 | -1.268 | -9.743 |  |
| Inmt | indolethylamine N-methyltransf | 1.92e-05 | 0.001944 | 3.437 | 1.781 | 5.563 | 0.06744 | 0.357 | 0.6018 | -0.7326 | -1.934 | 2.011e-10 | 8.805e-08 | 17.39 | 4.12 | 11.75 | 4.853e-09 | 8.326e-07 | 10.71 | 3.421 | 9.754 |  |
| Afm | afamin | 0.003822 | 0.05078 | 0.7697 | -0.3775 | -3.272 | 2.212e-07 | 6.184e-05 | 0.4836 | -1.048 | -7.676 | 6.811e-08 | 6.417e-06 | 2.067 | 1.047 | 8.286 | 2.431e-07 | 1.623e-05 | 1.951 | 0.9643 | 7.628 |  |
| Gm38481 | NA | 1.29e-06 | 0.0002983 | 0.3116 | -1.682 | -6.808 | 6.249e-09 | 4.122e-06 | 0.1427 | -2.809 | -9.607 | 0.0002206 | 0.003181 | 2.326 | 1.218 | 4.497 | 1.199e-07 | 9.638e-06 | 4.478 | 2.163 | 7.99 |  |
| Selenbp2 | selenium binding protein 2 | 0.001932 | 0.03427 | 0.5892 | -0.7631 | -3.567 | 2.625e-07 | 7.033e-05 | 0.2641 | -1.921 | -7.59 | 9.8e-07 | 4.921e-05 | 3.087 | 1.626 | 6.94 | 1.473e-07 | 1.105e-05 | 3.598 | 1.847 | 7.883 |  |
| Acadm | acyl-Coenzyme A dehydrogenase, | 0.01128 | 0.09901 | 1.292 | 0.3694 | 2.791 | 1.633e-07 | 4.828e-05 | 2.339 | 1.226 | 7.831 | 1.857e-09 | 4.304e-07 | 0.3542 | -1.497 | -10.33 | 3.815e-07 | 2.277e-05 | 0.4753 | -1.073 | -7.402 |  |
| Nsdhl | NAD(P) dependent steroid dehyd | 6.442e-06 | 0.0009578 | 0.4182 | -1.258 | -6.056 | 1.17e-08 | 5.679e-06 | 0.2069 | -2.273 | -9.248 | 0.0004692 | 0.005715 | 1.931 | 0.9497 | 4.174 | 1.254e-08 | 1.716e-06 | 4.273 | 2.095 | 9.209 |  |
| Cebpd | CCAAT/enhancer binding protein | 2.624e-11 | 1.405e-07 | 6.165 | 2.624 | 13.17 | 5.737e-06 | 0.0007521 | 2.714 | 1.44 | 6.109 | 0.001641 | 0.01494 | 1.734 | 0.7939 | 3.638 | 5.512e-11 | 2.403e-08 | 6.765 | 2.758 | 12.64 |  |
| Lrrfip1 | leucine rich repeat (in FLII) | 0.00161 | 0.03052 | 0.7185 | -0.4769 | -3.646 | 0.001361 | 0.03775 | 1.49 | 0.5754 | 3.718 | 3.055e-07 | 2.01e-05 | 0.4742 | -1.077 | -7.513 | 4.466e-12 | 3.961e-09 | 0.2364 | -2.08 | -14.52 |  |
| Slc7a8 | solute carrier family 7 (catio | 0.01973 | 0.1371 | 1.416 | 0.502 | 2.535 | 8.905e-05 | 0.005449 | 0.452 | -1.146 | -4.889 | 7.503e-11 | 4.196e-08 | 6.473 | 2.694 | 12.42 | 1.752e-09 | 3.663e-07 | 4.751 | 2.248 | 10.36 |  |
| Maff | v-maf musculoaponeurotic fibro | 2.604e-05 | 0.002395 | 2.119 | 1.083 | 5.427 | 0.08343 | 0.3925 | 0.7421 | -0.4303 | -1.822 | 4.511e-10 | 1.507e-07 | 5.471 | 2.452 | 11.22 | 6.862e-09 | 1.103e-06 | 4.253 | 2.088 | 9.553 |  |
| Ldha | lactate dehydrogenase A | 0.0003234 | 0.01126 | 0.7248 | -0.4643 | -4.333 | 0.0194 | 0.1902 | 1.25 | 0.3223 | 2.542 | 2.939e-10 | 1.129e-07 | 0.3925 | -1.349 | -11.49 | 3.702e-08 | 4.018e-06 | 0.4963 | -1.011 | -8.612 |  |
| Zfyve1 | zinc finger, FYVE domain conta | 8.312e-05 | 0.004882 | 1.558 | 0.6398 | 4.919 | 0.004897 | 0.08627 | 0.7136 | -0.4868 | -3.163 | 3.313e-06 | 0.0001233 | 1.875 | 0.9066 | 6.363 | 1.622e-13 | 3.476e-10 | 5.553 | 2.473 | 17.36 |  |
| Serpina4-ps1 | serine (or cysteine) peptidase | 0.00206 | 0.03568 | 0.3079 | -1.699 | -3.54 | 3.244e-07 | 8.262e-05 | 0.05254 | -4.25 | -7.483 | 5.444e-07 | 3.112e-05 | 13.93 | 3.8 | 7.226 | 4.433e-07 | 2.562e-05 | 14.45 | 3.853 | 7.328 |  |
| Mocos | molybdenum cofactor sulfurase | 0.0004358 | 0.01351 | 1.623 | 0.6982 | 4.206 | 0.001776 | 0.0448 | 0.6122 | -0.7079 | -3.604 | 2.134e-06 | 8.7e-05 | 2.289 | 1.195 | 6.569 | 3.634e-13 | 5.498e-10 | 8.132 | 3.024 | 16.63 |  |
| Hsdl2 | hydroxysteroid dehydrogenase l | 6.648e-05 | 0.00433 | 1.534 | 0.6178 | 5.016 | 1.071e-07 | 3.362e-05 | 2.255 | 1.173 | 8.048 | 1.063e-05 | 0.0003082 | 0.5798 | -0.7863 | -5.828 | 2.397e-06 | 9.648e-05 | 0.5438 | -0.8789 | -6.514 |  |
| Acot2 | acyl-CoA thioesterase 2 | 7.465e-07 | 0.0002043 | 3.958 | 1.985 | 7.072 | 2.659e-07 | 7.051e-05 | 5.729 | 2.518 | 7.583 | 2.252e-08 | 2.826e-06 | 0.1506 | -2.731 | -8.883 | 0.001493 | 0.01372 | 2.19 | 1.131 | 3.678 |  |
| Crat | carnitine acetyltransferase | 0.008839 | 0.08612 | 1.355 | 0.4381 | 2.901 | 3.215e-07 | 8.262e-05 | 2.528 | 1.338 | 7.488 | 5.432e-07 | 3.112e-05 | 0.4366 | -1.196 | -7.227 | 1.551e-08 | 1.998e-06 | 0.3526 | -1.504 | -9.09 |  |
| AA986860 | expressed sequence AA986860 | 0.02102 | 0.142 | 1.214 | 0.2793 | 2.505 | 5.631e-08 | 1.984e-05 | 2.153 | 1.106 | 8.387 | 5.417e-07 | 3.112e-05 | 0.5423 | -0.8829 | -7.229 | 2.944e-11 | 1.611e-08 | 0.3303 | -1.598 | -13.09 |  |
| 1810064F22Rik | RIKEN cDNA 1810064F22 gene | 0.002001 | 0.03494 | 0.518 | -0.949 | -3.552 | 3.359e-07 | 8.472e-05 | 0.1948 | -2.36 | -7.466 | 1.031e-06 | 5.09e-05 | 4.066 | 2.024 | 6.916 | 2.852e-07 | 1.798e-05 | 4.623 | 2.209 | 7.548 |  |
| Rab30 | RAB30, member RAS oncogene fam | 0.118 | 0.3741 | 1.275 | 0.3504 | 1.634 | 1.849e-08 | 8.061e-06 | 4.865 | 2.282 | 8.992 | 3.238e-10 | 1.173e-07 | 0.1554 | -2.686 | -11.43 | 2.41e-11 | 1.442e-08 | 0.1158 | -3.11 | -13.23 |  |
| Fgf11 | fibroblast growth factor 11 | 0.0007772 | 0.01919 | 0.6598 | -0.5999 | -3.958 | 0.01177 | 0.1437 | 1.411 | 0.4971 | 2.772 | 2.511e-10 | 9.786e-08 | 0.2632 | -1.926 | -11.6 | 7.664e-08 | 6.845e-06 | 0.3882 | -1.365 | -8.223 |  |
| 1810055G02Rik | RIKEN cDNA 1810055G02 gene | 0.1381 | 0.4078 | 1.208 | 0.2726 | 1.545 | 1.571e-08 | 7.482e-06 | 3.723 | 1.896 | 9.083 | 3.344e-11 | 2.253e-08 | 0.1753 | -2.512 | -12.99 | 1.021e-10 | 3.92e-08 | 0.1948 | -2.36 | -12.21 |  |
| Zwint | ZW10 interactor | 0.0003082 | 0.01098 | 1.473 | 0.5588 | 4.354 | 0.002456 | 0.055 | 1.44 | 0.526 | 3.464 | 3.857e-07 | 2.409e-05 | 0.4863 | -1.04 | -7.397 | 9.747e-08 | 8.167e-06 | 2.201 | 1.138 | 8.097 |  |
| Fdft1 | farnesyl diphosphate farnesyl | 0.03351 | 0.1879 | 0.7511 | -0.4129 | -2.283 | 2.132e-07 | 6.095e-05 | 0.3195 | -1.646 | -7.695 | 1.148e-07 | 9.346e-06 | 3.004 | 1.587 | 8.012 | 1.069e-09 | 2.545e-07 | 4.327 | 2.113 | 10.67 |  |
| Gcat | glycine C-acetyltransferase (2 | 3.628e-06 | 0.0006393 | 0.5957 | -0.7474 | -6.32 | 2.542e-08 | 1.006e-05 | 0.4253 | -1.234 | -8.816 | 3.818e-05 | 0.000815 | 1.603 | 0.6812 | 5.259 | 7.276e-06 | 0.0002308 | 1.714 | 0.7772 | 6 |  |
| Cyp1a2 | cytochrome P450, family 1, sub | 0.001895 | 0.03389 | 0.7217 | -0.4705 | -3.576 | 4.874e-07 | 0.0001117 | 0.4558 | -1.134 | -7.281 | 7.923e-07 | 4.168e-05 | 2.021 | 1.015 | 7.043 | 1.434e-06 | 6.517e-05 | 1.964 | 0.974 | 6.757 |  |
| Cpsf4l | cleavage and polyadenylation s | 0.05436 | 0.2465 | 1.17 | 0.2267 | 2.044 | 2.622e-07 | 7.033e-05 | 1.994 | 0.9958 | 7.59 | 2.741e-08 | 3.233e-06 | 0.4777 | -1.066 | -8.775 | 2.213e-09 | 4.518e-07 | 0.4229 | -1.242 | -10.22 |  |
| Slc22a28 | solute carrier family 22, memb | 0.003014 | 0.04427 | 0.6162 | -0.6985 | -3.375 | 5.293e-07 | 0.0001194 | 0.2926 | -1.773 | -7.24 | 5.444e-07 | 3.112e-05 | 3.113 | 1.638 | 7.226 | 8.805e-07 | 4.389e-05 | 3 | 1.585 | 6.992 |  |
| Slc19a1 | solute carrier family 19 (fola | 0.01099 | 0.09772 | 1.291 | 0.3686 | 2.803 | 2.479e-07 | 6.783e-05 | 2.274 | 1.185 | 7.618 | 8.958e-10 | 2.589e-07 | 0.3409 | -1.553 | -10.78 | 4.397e-06 | 0.0001533 | 0.5368 | -0.8977 | -6.231 |  |
| Aox3 | aldehyde oxidase 3 | 0.0403 | 0.2081 | 1.253 | 0.3255 | 2.193 | 2.944e-05 | 0.002411 | 0.5199 | -0.9436 | -5.373 | 3.447e-12 | 4.434e-09 | 5.257 | 2.394 | 14.73 | 3.19e-08 | 3.537e-06 | 2.663 | 1.413 | 8.692 |  |
| Rnpep | arginyl aminopeptidase (aminop | 8.1e-05 | 0.004801 | 0.6745 | -0.568 | -4.93 | 1.767e-07 | 5.165e-05 | 0.479 | -1.062 | -7.79 | 2.393e-05 | 0.000573 | 1.613 | 0.6898 | 5.465 | 3.984e-06 | 0.0001425 | 1.732 | 0.7923 | 6.277 |  |
| Zim1 | zinc finger, imprinted 1 | 1.391e-09 | 2.386e-06 | 10.23 | 3.355 | 10.5 | 0.08041 | 0.3877 | 1.62 | 0.6961 | 1.842 | 3.203e-13 | 6.866e-10 | 57.95 | 5.857 | 16.74 | 6.36e-06 | 0.00021 | 4.35 | 2.121 | 6.062 |  |
| Cyp51 | cytochrome P450, family 51 | 0.003249 | 0.04625 | 0.4944 | -1.016 | -3.342 | 6.475e-07 | 0.00014 | 0.1685 | -2.569 | -7.141 | 1.558e-06 | 6.85e-05 | 4.716 | 2.238 | 6.718 | 4.988e-07 | 2.777e-05 | 5.357 | 2.421 | 7.269 |  |
| Abcb1b | ATP-binding cassette, sub-fami | 1.807e-08 | 1.328e-05 | 2.777 | 1.473 | 9.005 | 0.004437 | 0.08152 | 1.538 | 0.6207 | 3.206 | 5.965e-09 | 1.044e-06 | 3.31 | 1.727 | 9.634 | 0.0001597 | 0.002499 | 1.779 | 0.831 | 4.636 |  |
| Acot12 | acyl-CoA thioesterase 12 | 0.02706 | 0.1654 | 1.205 | 0.2691 | 2.386 | 8.161e-07 | 0.0001693 | 1.916 | 0.938 | 7.029 | 6.516e-08 | 6.185e-06 | 0.4908 | -1.027 | -8.309 | 1.34e-07 | 1.048e-05 | 0.5069 | -0.9801 | -7.932 |  |
| Pold2 | polymerase (DNA directed), del | 0.01443 | 0.1138 | 0.7524 | -0.4105 | -2.679 | 3.408e-07 | 8.511e-05 | 0.3917 | -1.352 | -7.459 | 4.548e-06 | 0.00016 | 2.061 | 1.043 | 6.216 | 1.164e-09 | 2.697e-07 | 3.438 | 1.782 | 10.62 |  |
| Sort1 | sortilin 1 | 0.0007881 | 0.01929 | 0.6286 | -0.6698 | -3.952 | 4.198e-07 | 9.818e-05 | 0.3598 | -1.475 | -7.355 | 5.961e-05 | 0.001148 | 1.919 | 0.94 | 5.064 | 1.308e-07 | 1.035e-05 | 2.779 | 1.475 | 7.945 |  |
| Ifi205 | interferon activated gene 205 | 0.0102 | 0.09358 | 1.5 | 0.585 | 2.837 | 0.002092 | 0.05016 | 0.5502 | -0.8621 | -3.533 | 4.036e-08 | 4.186e-06 | 3.823 | 1.935 | 8.565 | 1.098e-09 | 2.59e-07 | 5.301 | 2.406 | 10.65 |  |
| Sc5d | sterol-C5-desaturase | 1.02e-06 | 0.0002547 | 0.4713 | -1.085 | -6.921 | 1.136e-08 | 5.624e-06 | 0.3038 | -1.719 | -9.265 | 0.003093 | 0.02393 | 1.493 | 0.5778 | 3.364 | 2.09e-07 | 1.449e-05 | 2.503 | 1.324 | 7.705 |  |
| Sik1 | salt inducible kinase 1 | 0.002067 | 0.03574 | 1.909 | 0.9329 | 3.538 | 0.01589 | 0.1691 | 0.5657 | -0.822 | -2.635 | 3.198e-08 | 3.577e-06 | 5.697 | 2.51 | 8.691 | 7.063e-09 | 1.122e-06 | 6.748 | 2.754 | 9.536 |  |
| Wbscr27 | NA | 0.01734 | 0.1275 | 1.435 | 0.5207 | 2.595 | 0.0009862 | 0.03063 | 0.5301 | -0.9157 | -3.856 | 6.218e-08 | 6.013e-06 | 3.561 | 1.832 | 8.334 | 4.161e-10 | 1.176e-07 | 5.567 | 2.477 | 11.27 |  |
| Cars | cysteinyl-tRNA synthetase | 0.02039 | 0.1397 | 0.7686 | -0.3798 | -2.519 | 1.171e-06 | 0.0002299 | 0.4285 | -1.222 | -6.854 | 1.482e-07 | 1.145e-05 | 2.464 | 1.301 | 7.881 | 3.605e-07 | 2.172e-05 | 2.341 | 1.227 | 7.43 |  |
| Hsd3b3 | hydroxy-delta-5-steroid dehydr | 0.009242 | 0.08817 | 0.7057 | -0.5029 | -2.881 | 1.194e-06 | 0.0002326 | 0.3753 | -1.414 | -6.845 | 2.094e-07 | 1.505e-05 | 2.776 | 1.473 | 7.704 | 2.055e-06 | 8.641e-05 | 2.394 | 1.259 | 6.587 |  |
| Cyp4f14 | cytochrome P450, family 4, sub | 0.001076 | 0.02388 | 0.6245 | -0.6792 | -3.819 | 5.894e-07 | 0.0001307 | 0.3505 | -1.512 | -7.187 | 2.745e-05 | 0.0006366 | 2.075 | 1.053 | 5.404 | 3.63e-07 | 2.177e-05 | 2.726 | 1.447 | 7.427 |  |
| Ppp1r15a | protein phosphatase 1, regulat | 0.0005532 | 0.01569 | 1.897 | 0.9239 | 4.104 | 0.03162 | 0.2478 | 0.6526 | -0.6157 | -2.311 | 4.692e-07 | 2.813e-05 | 3.483 | 1.8 | 7.3 | 3.253e-10 | 9.617e-08 | 7.055 | 2.819 | 11.43 |  |
| 1700039E15Rik | NA | 0.1242 | 0.3848 | 1.148 | 0.1997 | 1.605 | 5.684e-07 | 0.0001271 | 2.086 | 1.061 | 7.205 | 3.585e-09 | 7.039e-07 | 0.3913 | -1.354 | -9.933 | 1.761e-08 | 2.175e-06 | 0.4265 | -1.229 | -9.019 |  |
| 1600002H07Rik | NA | 2.029e-05 | 0.002015 | 1.981 | 0.9863 | 5.538 | 8.526e-08 | 2.816e-05 | 3.296 | 1.721 | 8.167 | 0.001511 | 0.01407 | 0.6085 | -0.7166 | -3.673 | 2.448e-07 | 1.624e-05 | 0.3567 | -1.487 | -7.625 |  |
| Lpin1 | lipin 1 | 1.728e-07 | 6.947e-05 | 2.914 | 1.543 | 7.802 | 5.376e-09 | 3.738e-06 | 4.818 | 2.269 | 9.695 | 0.000362 | 0.004666 | 0.5255 | -0.9283 | -4.285 | 1.226e-05 | 0.0003474 | 0.4208 | -1.249 | -5.764 |  |
| Fdps | farnesyl diphosphate synthetas | 1.109e-06 | 0.0002692 | 0.3213 | -1.638 | -6.88 | 1.084e-08 | 5.61e-06 | 0.163 | -2.617 | -9.291 | 0.009022 | 0.05257 | 1.687 | 0.7541 | 2.892 | 5.771e-08 | 5.644e-06 | 4.543 | 2.184 | 8.374 |  |
| Ms4a4a | membrane-spanning 4-domains, s | 1.579e-08 | 1.27e-05 | 3.996 | 1.998 | 9.08 | 0.1827 | 0.5434 | 1.283 | 0.3595 | 1.38 | 9.387e-12 | 1.006e-08 | 10.27 | 3.361 | 13.94 | 2.731e-06 | 0.0001055 | 2.94 | 1.556 | 6.453 |  |
| Insig1 | insulin induced gene 1 | 2.01e-06 | 0.0004254 | 0.3963 | -1.335 | -6.597 | 2.101e-09 | 1.949e-06 | 0.1823 | -2.456 | -10.25 | 0.07945 | 0.2437 | 1.329 | 0.4098 | 1.848 | 2.176e-11 | 1.428e-08 | 7.733 | 2.951 | 13.31 |  |
| Fbxo32 | F-box protein 32 | 2.846e-05 | 0.002548 | 1.899 | 0.9252 | 5.388 | 0.1288 | 0.4691 | 0.8 | -0.3219 | -1.584 | 7.488e-07 | 3.967e-05 | 2.514 | 1.33 | 7.07 | 4.265e-11 | 2.034e-08 | 5.32 | 2.411 | 12.82 |  |
| Gucd1 | guanylyl cyclase domain contai | 5.907e-06 | 0.0008923 | 1.946 | 0.9607 | 6.096 | 0.1306 | 0.4718 | 0.8156 | -0.294 | -1.576 | 2.305e-06 | 9.283e-05 | 2.185 | 1.128 | 6.532 | 1.768e-12 | 2.165e-09 | 6.217 | 2.636 | 15.27 |  |
| Mlxipl | MLX interacting protein-like | 1.344e-05 | 0.001516 | 0.6024 | -0.7312 | -5.722 | 1.104e-07 | 3.421e-05 | 0.4309 | -1.214 | -8.033 | 4.847e-05 | 0.0009787 | 1.649 | 0.7214 | 5.154 | 4.332e-05 | 0.0009277 | 1.657 | 0.7283 | 5.203 |  |
| Cdip1 | cell death inducing Trp53 targ | 0.00809 | 0.08148 | 1.411 | 0.4965 | 2.941 | 4.14e-07 | 9.77e-05 | 2.771 | 1.471 | 7.362 | 3.551e-05 | 0.0007776 | 0.5075 | -0.9785 | -5.291 | 1.378e-09 | 3.057e-07 | 0.2599 | -1.944 | -10.51 |  |
| Slc11a1 | solute carrier family 11 (prot | 0.002882 | 0.0429 | 1.421 | 0.5072 | 3.394 | 0.0163 | 0.1716 | 0.7251 | -0.4637 | -2.623 | 7.89e-09 | 1.261e-06 | 2.929 | 1.55 | 9.473 | 6.348e-08 | 5.996e-06 | 2.571 | 1.362 | 8.323 |  |
| Lncpint | long non-protein coding RNA, T | 1.21e-07 | 5.533e-05 | 2.975 | 1.573 | 7.985 | 0.02268 | 0.2067 | 1.49 | 0.5755 | 2.469 | 2.104e-05 | 0.0005165 | 2.284 | 1.192 | 5.522 | 6.093e-08 | 5.853e-06 | 3.484 | 1.801 | 8.345 |  |
| Npc1 | NPC intracellular cholesterol | 2.294e-07 | 8.429e-05 | 2.839 | 1.505 | 7.658 | 0.0304 | 0.2423 | 1.456 | 0.542 | 2.33 | 3.638e-07 | 2.305e-05 | 3.029 | 1.599 | 7.426 | 7.366e-06 | 0.0002331 | 2.447 | 1.291 | 5.995 |  |
| Srebf1 | sterol regulatory element bind | 7.111e-10 | 1.407e-06 | 0.1573 | -2.669 | -10.92 | 8.962e-11 | 2.305e-07 | 0.08513 | -3.554 | -12.3 | 0.02962 | 0.1248 | 1.544 | 0.6269 | 2.343 | 2.646e-07 | 1.716e-05 | 4.084 | 2.03 | 7.585 |  |
| Ddi2 | DNA-damage inducible protein 2 | 6.411e-08 | 3.436e-05 | 1.884 | 0.914 | 8.318 | 0.6116 | 0.8458 | 0.9546 | -0.06708 | -0.5159 | 3.995e-10 | 1.387e-07 | 2.566 | 1.359 | 11.29 | 2.675e-11 | 1.496e-08 | 2.997 | 1.584 | 13.16 |  |
| Slc27a1 | solute carrier family 27 (fatt | 3.912e-08 | 2.34e-05 | 3.206 | 1.681 | 8.582 | 1.58e-09 | 1.693e-06 | 5.338 | 2.416 | 10.43 | 0.04968 | 0.1777 | 0.7329 | -0.4483 | -2.089 | 8.095e-08 | 7.18e-06 | 0.2956 | -1.758 | -8.195 |  |
| Hykk | hydroxylysine kinase 1 | 0.01536 | 0.1183 | 0.6967 | -0.5214 | -2.65 | 0.002298 | 0.05273 | 1.757 | 0.813 | 3.492 | 7.083e-10 | 2.169e-07 | 0.1955 | -2.355 | -10.93 | 2.334e-07 | 1.576e-05 | 0.319 | -1.648 | -7.649 |  |
| Osgin1 | oxidative stress induced growt | 0.05023 | 0.2356 | 0.6936 | -0.5278 | -2.084 | 1.83e-08 | 8.061e-06 | 0.1543 | -2.696 | -8.998 | 9.722e-06 | 0.0002858 | 3.091 | 1.628 | 5.869 | 1.275e-13 | 3.08e-10 | 29.4 | 4.878 | 17.58 |  |
| Rhou | ras homolog family member U | 0.0002413 | 0.009464 | 1.744 | 0.8022 | 4.459 | 0.09685 | 0.416 | 0.7733 | -0.3709 | -1.742 | 4.3e-07 | 2.621e-05 | 2.727 | 1.447 | 7.343 | 1.371e-09 | 3.057e-07 | 4.205 | 2.072 | 10.51 |  |
| Prss8 | protease, serine 8 (prostasin) | 0.00178 | 0.03254 | 0.5768 | -0.7938 | -3.603 | 1.126e-06 | 0.0002263 | 0.2888 | -1.792 | -6.873 | 2.691e-05 | 0.000626 | 2.473 | 1.306 | 5.413 | 8.796e-07 | 4.389e-05 | 3.221 | 1.688 | 6.992 |  |
| Chac1 | ChaC, cation transport regulat | 0.005603 | 0.06563 | 1.777 | 0.8296 | 3.104 | 0.00605 | 0.09743 | 0.5102 | -0.9709 | -3.07 | 4.044e-10 | 1.387e-07 | 9.88 | 3.305 | 11.29 | 6.215e-07 | 3.31e-05 | 4.278 | 2.097 | 7.161 |  |
| 8430408G22Rik | NA | 2.368e-09 | 3.623e-06 | 14.6 | 3.868 | 10.18 | 0.6945 | 0.8858 | 0.8832 | -0.1791 | -0.3984 | 2.425e-10 | 9.673e-08 | 28.59 | 4.837 | 11.62 | 1.317e-13 | 3.08e-10 | 158.1 | 7.305 | 17.55 |  |
| Plxnd1 | plexin D1 | 2.111e-08 | 1.444e-05 | 0.4099 | -1.287 | -8.919 | 0.004296 | 0.07963 | 0.6832 | -0.5497 | -3.22 | 1.704e-09 | 4.059e-07 | 0.3208 | -1.64 | -10.38 | 0.001754 | 0.01557 | 0.6735 | -0.5703 | -3.609 |  |
| Prnd | prion like protein doppel | 2.612e-07 | 9.463e-05 | 0.2335 | -2.098 | -7.592 | 0.3428 | 0.6864 | 0.8023 | -0.3178 | -0.9717 | 1.045e-08 | 1.59e-06 | 0.1417 | -2.819 | -9.312 | 6.259e-08 | 5.963e-06 | 0.1741 | -2.522 | -8.33 |  |
| Gm3571 | predicted gene 3571 | 0.0001929 | 0.008202 | 0.4587 | -1.125 | -4.555 | 4.907e-07 | 0.0001117 | 0.2291 | -2.126 | -7.278 | 0.0003647 | 0.004688 | 2.231 | 1.158 | 4.282 | 1.244e-06 | 5.841e-05 | 3.594 | 1.846 | 6.825 |  |
| Fam149a | family with sequence similarit | 7.245e-05 | 0.004535 | 0.6332 | -0.6592 | -4.979 | 3.579e-07 | 8.709e-05 | 0.4461 | -1.165 | -7.434 | 0.0001219 | 0.002016 | 1.613 | 0.6894 | 4.753 | 1.407e-05 | 0.0003846 | 1.774 | 0.827 | 5.702 |  |
| Il6ra | interleukin 6 receptor, alpha | 9.384e-11 | 3.633e-07 | 6.589 | 2.72 | 12.26 | 4.763e-07 | 0.0001104 | 3.768 | 1.914 | 7.292 | 0.01375 | 0.07187 | 1.576 | 0.6562 | 2.701 | 5.115e-07 | 2.842e-05 | 3.394 | 1.763 | 7.257 |  |
| Cpt1b | carnitine palmitoyltransferase | 0.05394 | 0.2455 | 1.255 | 0.3277 | 2.048 | 2.185e-06 | 0.0003698 | 2.365 | 1.242 | 6.558 | 3.98e-08 | 4.145e-06 | 0.3529 | -1.503 | -8.572 | 7.521e-07 | 3.885e-05 | 0.4236 | -1.239 | -7.068 |  |
| Gjc3 | gap junction protein, gamma 3 | 0.01366 | 0.1108 | 1.643 | 0.7162 | 2.704 | 2.434e-06 | 0.0004046 | 4.11 | 2.039 | 6.507 | 3.294e-07 | 2.129e-05 | 0.2224 | -2.169 | -7.475 | 4.675e-06 | 0.0001614 | 0.2872 | -1.8 | -6.203 |  |
| Gng7 | guanine nucleotide binding pro | 0.0001269 | 0.006326 | 0.6488 | -0.6242 | -4.736 | 0.177 | 0.5374 | 1.163 | 0.2183 | 1.4 | 5.489e-09 | 1.001e-06 | 0.3794 | -1.398 | -9.683 | 1.661e-07 | 1.207e-05 | 0.4571 | -1.129 | -7.822 |  |
| 1500012F01Rik | NA | 5.741e-10 | 1.231e-06 | 2.679 | 1.422 | 11.06 | 3.466e-06 | 0.0005214 | 1.951 | 0.9645 | 6.342 | 0.001706 | 0.01537 | 1.424 | 0.5098 | 3.621 | 5.604e-06 | 0.0001892 | 1.817 | 0.8617 | 6.12 |  |
| Gamt | guanidinoacetate methyltransfe | 8.387e-10 | 1.541e-06 | 0.262 | -1.932 | -10.82 | 6.598e-07 | 0.0001414 | 0.3519 | -1.507 | -7.132 | 5.397e-08 | 5.299e-06 | 0.3198 | -1.645 | -8.409 | 0.07357 | 0.229 | 1.292 | 0.3694 | 1.888 |  |
| Akr1c6 | aldo-keto reductase family 1, | 0.007354 | 0.07668 | 0.7356 | -0.443 | -2.983 | 2.119e-06 | 0.0003633 | 0.4491 | -1.155 | -6.572 | 1.693e-06 | 7.259e-05 | 2.123 | 1.086 | 6.678 | 2.759e-06 | 0.0001063 | 2.069 | 1.049 | 6.448 |  |
| Plin4 | perilipin 4 | 0.0002698 | 0.01007 | 2.086 | 1.06 | 4.411 | 6.429e-07 | 0.00014 | 4.091 | 2.032 | 7.145 | 3.449e-07 | 2.201e-05 | 0.2565 | -1.963 | -7.453 | 0.001715 | 0.01527 | 0.5166 | -0.953 | -3.619 |  |
| Klk1b4 | kallikrein 1-related pepidase | 0.003509 | 0.04816 | 0.6167 | -0.6973 | -3.309 | 1.74e-06 | 0.0003176 | 0.316 | -1.662 | -6.665 | 5.693e-06 | 0.0001909 | 2.659 | 1.411 | 6.113 | 2.907e-06 | 0.0001101 | 2.795 | 1.483 | 6.424 |  |
| Clcn6 | chloride channel, voltage-sens | 0.007543 | 0.07799 | 0.7732 | -0.3712 | -2.972 | 1.699e-06 | 0.0003144 | 0.5047 | -0.9866 | -6.677 | 1.922e-05 | 0.000479 | 1.695 | 0.761 | 5.562 | 1.337e-07 | 1.048e-05 | 2.122 | 1.085 | 7.933 |  |
| Foxo1 | forkhead box O1 | 5.92e-08 | 3.24e-05 | 2.911 | 1.542 | 8.36 | 1.531e-05 | 0.001485 | 2.355 | 1.236 | 5.664 | 0.006326 | 0.04061 | 0.6524 | -0.6162 | -3.05 | 2.617e-07 | 1.713e-05 | 2.895 | 1.534 | 7.591 |  |
| Mbl1 | mannose-binding lectin (protei | 0.002201 | 0.03676 | 0.692 | -0.5312 | -3.511 | 1.741e-06 | 0.0003176 | 0.4374 | -1.193 | -6.665 | 2.655e-06 | 0.0001042 | 2.102 | 1.072 | 6.466 | 2.136e-05 | 0.0005313 | 1.884 | 0.9141 | 5.516 |  |
| Gm16551 | predicted gene 16551 | 0.01822 | 0.1313 | 0.5725 | -0.8047 | -2.572 | 2.753e-06 | 0.0004383 | 0.1911 | -2.388 | -6.449 | 1.083e-06 | 5.237e-05 | 5.142 | 2.362 | 6.892 | 9.348e-07 | 4.633e-05 | 5.229 | 2.387 | 6.963 |  |
| Crcp | calcitonin gene-related peptid | 0.04727 | 0.2285 | 1.305 | 0.3839 | 2.114 | 2.904e-06 | 0.0004547 | 2.603 | 1.38 | 6.424 | 4.457e-07 | 2.691e-05 | 0.3643 | -1.457 | -7.325 | 2.103e-07 | 1.454e-05 | 0.3458 | -1.532 | -7.702 |  |
| Acot1 | acyl-CoA thioesterase 1 | 5.516e-08 | 3.085e-05 | 2.83 | 1.501 | 8.398 | 3.864e-10 | 5.538e-07 | 5.253 | 2.393 | 11.32 | 3.51e-11 | 2.257e-08 | 0.1722 | -2.538 | -12.96 | 0.4862 | 0.7013 | 0.9082 | -0.1389 | -0.7095 |  |
| Sf1 | splicing factor 1 | 0.0004331 | 0.01344 | 1.38 | 0.4648 | 4.208 | 0.1187 | 0.4544 | 0.8627 | -0.2131 | -1.63 | 6.38e-08 | 6.123e-06 | 2.009 | 1.007 | 8.32 | 4.655e-08 | 4.813e-06 | 2.038 | 1.027 | 8.488 |  |
| Mir704 | microRNA 704 | 6.054e-05 | 0.004066 | 2.476 | 1.308 | 5.057 | 3.835e-07 | 9.22e-05 | 4.805 | 2.264 | 7.4 | 0.0002163 | 0.003133 | 0.4128 | -1.277 | -4.506 | 1.678e-05 | 0.0004411 | 0.3315 | -1.593 | -5.623 |  |
| Rarb | retinoic acid receptor, beta | 3.202e-05 | 0.002746 | 0.4913 | -1.025 | -5.336 | 0.002056 | 0.04948 | 0.5724 | -0.8048 | -3.54 | 0.0001427 | 0.002268 | 1.981 | 0.986 | 4.685 | 1.646e-07 | 1.203e-05 | 0.3193 | -1.647 | -7.827 |  |
| Rala | v-ral simian leukemia viral on | 0.003632 | 0.04908 | 0.7792 | -0.36 | -3.294 | 0.02228 | 0.2049 | 1.249 | 0.3204 | 2.478 | 9.236e-08 | 8.053e-06 | 0.5095 | -0.9729 | -8.126 | 2.094e-08 | 2.541e-06 | 0.4769 | -1.068 | -8.923 |  |
| Whamm | WAS protein homolog associated | 0.003218 | 0.04594 | 1.427 | 0.5127 | 3.347 | 0.02479 | 0.2167 | 0.7371 | -0.44 | -2.427 | 1.243e-07 | 9.858e-06 | 2.527 | 1.338 | 7.971 | 1.639e-08 | 2.098e-06 | 2.868 | 1.52 | 9.059 |  |
| Acer2 | alkaline ceramidase 2 | 6.867e-08 | 3.605e-05 | 2.729 | 1.448 | 8.281 | 0.01612 | 0.1703 | 1.458 | 0.5439 | 2.628 | 2.128e-09 | 4.802e-07 | 3.899 | 1.963 | 10.25 | 0.0008537 | 0.009026 | 1.683 | 0.7507 | 3.918 |  |
| Oplah | 5-oxoprolinase (ATP-hydrolysin | 0.3149 | 0.6165 | 0.9158 | -0.1269 | -1.031 | 1.127e-05 | 0.001191 | 1.797 | 0.8452 | 5.802 | 8.942e-11 | 4.792e-08 | 0.3167 | -1.659 | -12.3 | 1.02e-08 | 1.449e-06 | 0.4182 | -1.258 | -9.326 |  |
| Catsper2 | cation channel, sperm associat | 2.355e-05 | 0.002236 | 1.856 | 0.8925 | 5.472 | 2.21e-07 | 6.184e-05 | 2.792 | 1.481 | 7.676 | 0.001251 | 0.01213 | 0.6282 | -0.6708 | -3.754 | 5.424e-06 | 0.0001843 | 0.4678 | -1.096 | -6.135 |  |
| Arrdc2 | arrestin domain containing 2 | 9.104e-08 | 4.592e-05 | 3.927 | 1.973 | 8.133 | 0.006983 | 0.1072 | 1.819 | 0.8631 | 3.006 | 0.0003928 | 0.004984 | 2.188 | 1.13 | 4.25 | 6.861e-08 | 6.303e-06 | 4.599 | 2.201 | 8.282 |  |
| Gadd45b | growth arrest and DNA-damage-i | 1.515e-05 | 0.001638 | 3.092 | 1.628 | 5.669 | 9.237e-08 | 2.97e-05 | 6.782 | 2.762 | 8.126 | 0.05672 | 0.1942 | 0.6433 | -0.6364 | -2.023 | 2.703e-08 | 3.118e-06 | 0.1473 | -2.764 | -8.783 |  |
| Eps8l2 | EPS8-like 2 | 0.02504 | 0.1583 | 0.7992 | -0.3233 | -2.423 | 2.499e-06 | 0.0004094 | 0.4912 | -1.026 | -6.494 | 2.788e-08 | 3.234e-06 | 2.431 | 1.282 | 8.766 | 1.6e-05 | 0.0004248 | 1.772 | 0.8252 | 5.644 |  |
| Car4 | carbonic anhydrase 4 | 3.598e-07 | 0.0001234 | 2.361 | 1.239 | 7.431 | 0.3608 | 0.6992 | 1.136 | 0.1846 | 0.9354 | 2.991e-09 | 6.156e-07 | 3.566 | 1.834 | 10.04 | 4.878e-07 | 2.74e-05 | 2.514 | 1.33 | 7.28 |  |
| Rnft2 | ring finger protein, transmemb | 4.424e-07 | 0.0001371 | 0.5382 | -0.8937 | -7.329 | 0.07808 | 0.3827 | 0.8305 | -0.268 | -1.857 | 2.028e-08 | 2.621e-06 | 0.437 | -1.194 | -8.941 | 5.34e-05 | 0.00109 | 0.6229 | -0.6828 | -5.112 |  |
| Def8 | differentially expressed in FD | 0.002407 | 0.0386 | 0.7144 | -0.4851 | -3.472 | 0.04071 | 0.2822 | 1.285 | 0.3617 | 2.188 | 3.801e-08 | 4.039e-06 | 0.4017 | -1.316 | -8.597 | 1.169e-07 | 9.459e-06 | 0.4279 | -1.225 | -8.003 |  |
| Tmem140 | transmembrane protein 140 | 0.0002026 | 0.008473 | 2.162 | 1.113 | 4.534 | 0.1695 | 0.5283 | 0.7506 | -0.4139 | -1.425 | 5.322e-07 | 3.096e-05 | 3.852 | 1.946 | 7.238 | 4.502e-09 | 7.825e-07 | 6.207 | 2.634 | 9.798 |  |
| Pgd | phosphogluconate dehydrogenase | 1.418e-05 | 0.00156 | 0.5175 | -0.9504 | -5.699 | 9.073e-06 | 0.001019 | 0.4462 | -1.164 | -5.9 | 0.002751 | 0.02195 | 0.6489 | -0.6238 | -3.415 | 1.008e-06 | 4.94e-05 | 2.404 | 1.265 | 6.926 |  |
| Slc41a3 | solute carrier family 41, memb | 0.9058 | 0.9649 | 1.017 | 0.02456 | 0.1199 | 5.854e-09 | 3.962e-06 | 5.058 | 2.338 | 9.645 | 2.239e-11 | 1.883e-08 | 0.1265 | -2.982 | -13.29 | 1.199e-13 | 3.08e-10 | 0.0643 | -3.959 | -17.64 |  |
| Cntnap1 | contactin associated protein-l | 0.1801 | 0.4685 | 1.196 | 0.2586 | 1.389 | 3.427e-06 | 0.0005214 | 2.636 | 1.398 | 6.347 | 2.434e-08 | 3.014e-06 | 0.2866 | -1.803 | -8.84 | 1.367e-07 | 1.056e-05 | 0.3263 | -1.616 | -7.922 |  |
| Cyp21a2-ps | cytochrome P450, family 21, su | 0.00372 | 0.04984 | 0.6392 | -0.6457 | -3.283 | 2.453e-06 | 0.0004046 | 0.3503 | -1.513 | -6.503 | 1.002e-06 | 4.977e-05 | 2.814 | 1.493 | 6.929 | 4.874e-05 | 0.001019 | 2.158 | 1.11 | 5.152 |  |
| Map3k13 | mitogen-activated protein kina | 0.002603 | 0.04026 | 0.6809 | -0.5544 | -3.439 | 2.181e-06 | 0.0003698 | 0.4201 | -1.251 | -6.559 | 6.635e-07 | 3.639e-05 | 2.394 | 1.259 | 7.129 | 0.0001359 | 0.002221 | 1.779 | 0.8312 | 4.706 |  |
| Pank1 | pantothenate kinase 1 | 4.74e-05 | 0.003481 | 1.882 | 0.9124 | 5.164 | 0.002224 | 0.05182 | 1.662 | 0.7331 | 3.507 | 0.0001474 | 0.002324 | 0.5344 | -0.9041 | -4.671 | 3.469e-07 | 2.1e-05 | 2.717 | 1.442 | 7.45 |  |
| Usp3 | ubiquitin specific peptidase 3 | 1.591e-07 | 6.496e-05 | 2.126 | 1.088 | 7.844 | 0.6833 | 0.8814 | 1.048 | 0.06794 | 0.414 | 6.464e-09 | 1.108e-06 | 2.745 | 1.457 | 9.588 | 1.287e-09 | 2.93e-07 | 3.039 | 1.604 | 10.55 |  |
| Igfals | insulin-like growth factor bin | 1.226e-07 | 5.533e-05 | 0.2692 | -1.893 | -7.978 | 3.575e-05 | 0.002821 | 0.3573 | -1.485 | -5.288 | 4.347e-07 | 2.644e-05 | 0.2666 | -1.907 | -7.337 | 0.01635 | 0.07997 | 1.604 | 0.6816 | 2.622 |  |
| Slc37a4 | solute carrier family 37 (gluc | 0.8012 | 0.9228 | 1.028 | 0.04009 | 0.2552 | 1.182e-07 | 3.619e-05 | 2.802 | 1.487 | 7.997 | 1.343e-10 | 6.396e-08 | 0.2384 | -2.068 | -12.02 | 2.533e-11 | 1.448e-08 | 0.2072 | -2.271 | -13.2 |  |
| 1700001C19Rik | RIKEN cDNA 1700001C19 gene | 0.00959 | 0.09003 | 0.7613 | -0.3935 | -2.865 | 3.683e-06 | 0.0005466 | 0.4911 | -1.026 | -6.314 | 7.09e-06 | 0.0002266 | 1.872 | 0.9046 | 6.012 | 1.992e-06 | 8.429e-05 | 1.991 | 0.9932 | 6.601 |  |
| Ces1g | carboxylesterase 1G | 0.08999 | 0.3232 | 0.8508 | -0.2331 | -1.782 | 4.73e-06 | 0.0006577 | 0.5144 | -0.9592 | -6.198 | 2.769e-07 | 1.864e-05 | 2.119 | 1.084 | 7.563 | 2.373e-07 | 1.598e-05 | 2.136 | 1.095 | 7.641 |  |
| Sgk1 | serum/glucocorticoid regulated | 3.584e-05 | 0.002974 | 1.862 | 0.8969 | 5.286 | 0.0126 | 0.1493 | 1.464 | 0.5502 | 2.741 | 5.688e-09 | 1.016e-06 | 3.472 | 1.796 | 9.662 | 0.0006031 | 0.006966 | 0.5922 | -0.7558 | -4.067 |  |
| Crls1 | cardiolipin synthase 1 | 7.832e-05 | 0.004696 | 1.809 | 0.8554 | 4.945 | 0.03334 | 0.2558 | 1.383 | 0.4679 | 2.286 | 9.557e-10 | 2.624e-07 | 4.098 | 2.035 | 10.74 | 0.0001727 | 0.00266 | 0.5463 | -0.8723 | -4.603 |  |
| Abcb4 | ATP-binding cassette, sub-fami | 0.2019 | 0.4968 | 1.13 | 0.1761 | 1.32 | 1.938e-06 | 0.0003453 | 2.063 | 1.045 | 6.614 | 7.291e-07 | 3.899e-05 | 0.4878 | -1.036 | -7.083 | 9.591e-10 | 2.306e-07 | 0.3368 | -1.57 | -10.74 |  |
| Slc19a2 | solute carrier family 19 (thia | 0.01085 | 0.09695 | 1.361 | 0.4447 | 2.809 | 0.01318 | 0.1525 | 0.7024 | -0.5096 | -2.721 | 1.172e-07 | 9.449e-06 | 2.616 | 1.388 | 8.002 | 4.497e-08 | 4.728e-06 | 2.78 | 1.475 | 8.507 |  |
| Pptc7 | PTC7 protein phosphatase homol | 0.3073 | 0.6099 | 1.142 | 0.1918 | 1.048 | 6.875e-07 | 0.0001462 | 2.909 | 1.54 | 7.112 | 4.761e-07 | 2.828e-05 | 0.3629 | -1.462 | -7.292 | 3.898e-11 | 2.022e-08 | 0.1668 | -2.584 | -12.88 |  |
| Slc10a6 | solute carrier family 10 (sodi | 1.108e-08 | 9.789e-06 | 3.832 | 1.938 | 9.279 | 8.55e-05 | 0.005304 | 2.318 | 1.213 | 4.907 | 0.0002488 | 0.003484 | 2.024 | 1.017 | 4.446 | 5.914e-05 | 0.001176 | 2.234 | 1.159 | 5.067 |  |
| Gm15453 | NA | 0.002335 | 0.03786 | 1.63 | 0.7052 | 3.486 | 2.346e-06 | 0.0003944 | 2.952 | 1.562 | 6.524 | 4.146e-05 | 0.0008672 | 0.4483 | -1.157 | -5.222 | 3.243e-06 | 0.00012 | 0.3757 | -1.412 | -6.373 |  |
| Hps5 | HPS5, biogenesis of lysosomal | 0.001633 | 0.03077 | 0.7575 | -0.4006 | -3.64 | 0.05679 | 0.3291 | 1.2 | 0.2633 | 2.022 | 1.348e-06 | 6.166e-05 | 0.5671 | -0.8182 | -6.787 | 5.035e-09 | 8.41e-07 | 0.4434 | -1.173 | -9.733 |  |
| Gabarapl1 | gamma-aminobutyric acid (GABA) | 2.535e-09 | 3.623e-06 | 2.658 | 1.41 | 10.14 | 0.8933 | 0.964 | 1.016 | 0.02236 | 0.1358 | 1.463e-08 | 2.057e-06 | 2.621 | 1.39 | 9.123 | 6.598e-14 | 2.121e-10 | 6.841 | 2.774 | 18.21 |  |
| Smad3 | SMAD family member 3 | 2.222e-05 | 0.00216 | 0.6491 | -0.6236 | -5.498 | 0.003036 | 0.06339 | 0.7308 | -0.4525 | -3.372 | 0.0007408 | 0.008038 | 1.409 | 0.4944 | 3.979 | 9.529e-08 | 8.094e-06 | 0.4974 | -1.008 | -8.109 |  |
| Rorc | RAR-related orphan receptor ga | 4.007e-07 | 0.0001321 | 2.108 | 1.076 | 7.378 | 0.2275 | 0.5885 | 1.161 | 0.2148 | 1.245 | 9.166e-10 | 2.591e-07 | 3.292 | 1.719 | 10.76 | 2.733e-05 | 0.0006396 | 1.819 | 0.8634 | 5.406 |  |
| Gm19680 | predicted gene, 19680 | 0.001892 | 0.03387 | 0.6097 | -0.7138 | -3.576 | 2.772e-06 | 0.0004383 | 0.3482 | -1.522 | -6.446 | 1.6e-05 | 0.0004181 | 2.352 | 1.234 | 5.644 | 2.483e-05 | 0.0005965 | 2.283 | 1.191 | 5.449 |  |
| Fbxo31 | F-box protein 31 | 9.944e-09 | 9.136e-06 | 4.161 | 2.057 | 9.341 | 3.589e-07 | 8.709e-05 | 3.828 | 1.937 | 7.433 | 6.301e-05 | 0.001194 | 2.322 | 1.216 | 5.039 | 0.00204 | 0.01742 | 0.5529 | -0.8548 | -3.544 |  |
| Atp4a | ATPase, H+/K+ exchanging, gast | 0.004816 | 0.05919 | 0.7198 | -0.4744 | -3.17 | 3.745e-06 | 0.0005505 | 0.4612 | -1.116 | -6.306 | 5.53e-06 | 0.0001871 | 2.006 | 1.004 | 6.126 | 1.672e-05 | 0.0004401 | 1.895 | 0.922 | 5.625 |  |
| Adipor2 | adiponectin receptor 2 | 0.4116 | 0.6986 | 1.07 | 0.09713 | 0.8387 | 1.372e-05 | 0.001395 | 0.5812 | -0.7829 | -5.713 | 5.234e-11 | 3.284e-08 | 3.048 | 1.608 | 12.67 | 9.042e-08 | 7.842e-06 | 2.045 | 1.032 | 8.137 |  |
| Ctsl | cathepsin L | 7.612e-05 | 0.004651 | 1.711 | 0.7752 | 4.957 | 0.001085 | 0.03306 | 1.631 | 0.706 | 3.815 | 7.693e-05 | 0.001393 | 0.5554 | -0.8484 | -4.952 | 5.083e-06 | 0.0001743 | 2.079 | 1.056 | 6.165 |  |
| Thrsp | thyroid hormone responsive | 0.0007918 | 0.01934 | 0.331 | -1.595 | -3.95 | 2.047e-06 | 0.0003534 | 0.1128 | -3.148 | -6.588 | 0.0001228 | 0.002025 | 4.29 | 2.101 | 4.75 | 1.18e-05 | 0.000337 | 5.886 | 2.557 | 5.781 |  |
| Prkag3 | protein kinase, AMP-activated, | 1.125e-06 | 0.0002704 | 0.3394 | -1.559 | -6.874 | 0.338 | 0.6832 | 0.8331 | -0.2635 | -0.9816 | 9.904e-07 | 4.937e-05 | 0.3029 | -1.723 | -6.935 | 3.098e-08 | 3.45e-06 | 0.2232 | -2.164 | -8.708 |  |
| Ptprs | protein tyrosine phosphatase, | 1.406e-06 | 0.0003201 | 0.4546 | -1.137 | -6.767 | 0.3699 | 0.7064 | 0.8812 | -0.1824 | -0.9173 | 2.453e-07 | 1.701e-05 | 0.3779 | -1.404 | -7.624 | 1.333e-07 | 1.048e-05 | 0.3632 | -1.461 | -7.935 |  |
| Fgfr3 | fibroblast growth factor recep | 0.07454 | 0.2918 | 1.262 | 0.3352 | 1.882 | 0.0001052 | 0.006142 | 0.4947 | -1.015 | -4.817 | 3.144e-12 | 4.257e-09 | 7.402 | 2.888 | 14.8 | 7.91e-06 | 0.0002465 | 2.24 | 1.163 | 5.962 |  |
| Sesn1 | sestrin 1 | 3.112e-10 | 8.109e-07 | 2.528 | 1.338 | 11.46 | 0.9027 | 0.9676 | 0.9882 | -0.0171 | -0.1238 | 3.335e-08 | 3.682e-06 | 2.157 | 1.109 | 8.668 | 6.93e-16 | 1.783e-11 | 7.759 | 2.956 | 23.11 |  |
| Slc22a7 | solute carrier family 22 (orga | 0.07459 | 0.2919 | 0.6991 | -0.5164 | -1.881 | 6.596e-06 | 0.0008253 | 0.2564 | -1.963 | -6.045 | 3.831e-07 | 2.409e-05 | 4.676 | 2.225 | 7.4 | 8.04e-07 | 4.071e-05 | 4.334 | 2.116 | 7.036 |  |
| Lifr | LIF receptor alpha | 0.1542 | 0.4314 | 0.8498 | -0.2348 | -1.481 | 2.777e-06 | 0.0004383 | 0.4326 | -1.209 | -6.445 | 1.237e-09 | 3.089e-07 | 3.572 | 1.837 | 10.58 | 4.219e-06 | 0.0001492 | 2.122 | 1.085 | 6.251 |  |
| Gm10156 | predicted gene 10156 | 0.004125 | 0.0533 | 1.501 | 0.586 | 3.238 | 3.459e-06 | 0.0005214 | 2.564 | 1.358 | 6.343 | 2.108e-05 | 0.0005169 | 0.4683 | -1.095 | -5.521 | 5.101e-06 | 0.0001747 | 0.4287 | -1.222 | -6.163 |  |
| Adra1b | adrenergic receptor, alpha 1b | 0.01966 | 0.1369 | 0.7249 | -0.4641 | -2.536 | 5.762e-06 | 0.0007521 | 0.3999 | -1.322 | -6.107 | 3.864e-06 | 0.0001392 | 2.397 | 1.261 | 6.291 | 2.211e-06 | 9.072e-05 | 2.485 | 1.313 | 6.552 |  |
| Slc17a8 | solute carrier family 17 (sodi | 0.01205 | 0.1024 | 1.471 | 0.5566 | 2.761 | 5.448e-06 | 0.0007262 | 2.756 | 1.463 | 6.133 | 6.455e-06 | 0.0002114 | 0.3958 | -1.337 | -6.055 | 4.177e-06 | 0.0001485 | 0.3839 | -1.381 | -6.255 |  |
| Hsd17b10 | hydroxysteroid (17-beta) dehyd | 1.053e-05 | 0.001361 | 1.713 | 0.7763 | 5.833 | 2.519e-06 | 0.0004101 | 2.031 | 1.022 | 6.491 | 0.03726 | 0.1465 | 1.253 | 0.3254 | 2.232 | 4.776e-07 | 2.705e-05 | 0.4786 | -1.063 | -7.291 |  |
| Mafk | v-maf musculoaponeurotic fibro | 0.0002373 | 0.009383 | 1.758 | 0.8142 | 4.466 | 0.1228 | 0.4608 | 0.7859 | -0.3476 | -1.611 | 9.789e-06 | 0.0002871 | 2.252 | 1.171 | 5.865 | 2.553e-10 | 7.818e-08 | 4.973 | 2.314 | 11.59 |  |
| Acnat2 | acyl-coenzyme A amino acid N-a | 0.03833 | 0.2025 | 1.438 | 0.5239 | 2.218 | 6.044e-06 | 0.0007773 | 3.251 | 1.701 | 6.085 | 4.24e-06 | 0.0001511 | 0.326 | -1.617 | -6.248 | 3.863e-07 | 2.3e-05 | 0.2654 | -1.914 | -7.396 |  |
| Msmo1 | methylsterol monoxygenase 1 | 0.0002209 | 0.008969 | 0.4273 | -1.227 | -4.497 | 1.228e-06 | 0.0002375 | 0.2169 | -2.205 | -6.832 | 0.001573 | 0.01449 | 2.132 | 1.092 | 3.656 | 5.075e-06 | 0.0001743 | 3.586 | 1.842 | 6.165 |  |
| Fbxo36 | F-box protein 36 | 0.2623 | 0.5677 | 1.168 | 0.2237 | 1.154 | 6.786e-06 | 0.0008432 | 2.61 | 1.384 | 6.032 | 1.171e-07 | 9.449e-06 | 0.3078 | -1.7 | -8.002 | 5.647e-08 | 5.586e-06 | 0.291 | -1.781 | -8.385 |  |
| Plekhm2 | pleckstrin homology domain con | 0.005361 | 0.06379 | 1.316 | 0.3965 | 3.123 | 0.03588 | 0.2661 | 0.7911 | -0.338 | -2.25 | 1.697e-07 | 1.284e-05 | 2.123 | 1.086 | 7.811 | 1.103e-07 | 9.093e-06 | 2.169 | 1.117 | 8.033 |  |
| Spryd3 | SPRY domain containing 3 | 0.7362 | 0.8937 | 1.042 | 0.05964 | 0.3417 | 1.43e-06 | 0.0002685 | 2.632 | 1.396 | 6.759 | 2.402e-10 | 9.673e-08 | 0.2141 | -2.224 | -11.63 | 1.001e-08 | 1.437e-06 | 0.2901 | -1.785 | -9.337 |  |
| Nudt4 | nudix (nucleoside diphosphate | 0.02637 | 0.1631 | 0.7741 | -0.3694 | -2.398 | 0.00486 | 0.08621 | 1.492 | 0.5772 | 3.166 | 9.763e-07 | 4.921e-05 | 0.444 | -1.171 | -6.942 | 3.883e-09 | 7.034e-07 | 0.3146 | -1.668 | -9.886 |  |
| Arnt | aryl hydrocarbon receptor nucl | 0.0001233 | 0.006245 | 1.515 | 0.5994 | 4.748 | 0.3127 | 0.6636 | 0.8983 | -0.1547 | -1.036 | 3.009e-07 | 1.988e-05 | 2.056 | 1.04 | 7.521 | 2.444e-08 | 2.858e-06 | 2.333 | 1.222 | 8.838 |  |
| Ciart | circadian associated repressor | 0.08263 | 0.3073 | 1.388 | 0.4727 | 1.827 | 8.053e-06 | 0.0009547 | 3.537 | 1.822 | 5.954 | 4.883e-07 | 2.874e-05 | 0.2393 | -2.063 | -7.28 | 8.499e-07 | 4.262e-05 | 0.2524 | -1.986 | -7.009 |  |
| Zfp598 | zinc finger protein 598 | 0.0009756 | 0.02243 | 1.415 | 0.5011 | 3.861 | 0.02201 | 0.2038 | 0.7677 | -0.3814 | -2.483 | 8.792e-12 | 9.833e-09 | 3.97 | 1.989 | 13.99 | 0.0001622 | 0.002529 | 1.578 | 0.6583 | 4.63 |  |
| Mettl7a1 | methyltransferase like 7A1 | 0.001157 | 0.02496 | 1.449 | 0.535 | 3.788 | 0.1087 | 0.4378 | 0.8232 | -0.2806 | -1.679 | 1.689e-08 | 2.311e-06 | 2.637 | 1.399 | 9.042 | 1.392e-06 | 6.369e-05 | 2.067 | 1.048 | 6.772 |  |
| Retsat | retinol saturase (all trans re | 1.204e-06 | 0.0002867 | 1.872 | 0.9048 | 6.841 | 5.595e-08 | 1.984e-05 | 2.485 | 1.313 | 8.39 | 4.075e-05 | 0.000857 | 0.5914 | -0.7578 | -5.23 | 0.004282 | 0.03029 | 0.7236 | -0.4668 | -3.222 |  |
| Bcl6b | B cell CLL/lymphoma 6, member | 0.0001399 | 0.006708 | 0.4718 | -1.084 | -4.694 | 0.2306 | 0.5918 | 1.264 | 0.3378 | 1.237 | 9.413e-10 | 2.624e-07 | 0.1519 | -2.718 | -10.75 | 5.683e-06 | 0.0001916 | 0.3424 | -1.546 | -6.113 |  |
| Sec23a | SEC23 homolog A, COPII coat co | 0.01901 | 0.1345 | 0.8228 | -0.2814 | -2.552 | 0.009595 | 0.1283 | 1.296 | 0.3737 | 2.864 | 9.61e-09 | 1.489e-06 | 0.4568 | -1.131 | -9.36 | 1.043e-06 | 5.08e-05 | 0.5607 | -0.8346 | -6.91 |  |
| Tor1a | torsin family 1, member A (tor | 7.003e-05 | 0.004456 | 1.518 | 0.6018 | 4.993 | 7.91e-07 | 0.0001654 | 2.006 | 1.004 | 7.044 | 8.798e-06 | 0.0002653 | 0.5821 | -0.7808 | -5.914 | 0.004151 | 0.02958 | 0.7437 | -0.4272 | -3.236 |  |
| Rnf19b | ring finger protein 19B | 0.06395 | 0.2692 | 0.8241 | -0.2791 | -1.961 | 7.481e-06 | 0.0009034 | 0.4972 | -1.008 | -5.988 | 2.327e-06 | 9.31e-05 | 2.025 | 1.018 | 6.528 | 3.117e-07 | 1.918e-05 | 2.25 | 1.17 | 7.503 |  |
| Ip6k2 | inositol hexaphosphate kinase | 0.0002862 | 0.0104 | 1.542 | 0.6244 | 4.386 | 1.781e-06 | 0.0003226 | 2.175 | 1.121 | 6.654 | 2.46e-05 | 0.0005866 | 0.5547 | -0.8504 | -5.453 | 0.0005597 | 0.006572 | 0.6421 | -0.6392 | -4.099 |  |
| Apol10b | apolipoprotein L 10B | 6.551e-07 | 0.0001812 | 0.4419 | -1.178 | -7.136 | 0.02252 | 0.2059 | 0.7155 | -0.483 | -2.473 | 5.666e-05 | 0.0011 | 0.5286 | -0.9198 | -5.086 | 2.785e-06 | 0.0001069 | 0.4458 | -1.165 | -6.444 |  |
| Abcb10 | ATP-binding cassette, sub-fami | 0.0004283 | 0.01338 | 0.6655 | -0.5875 | -4.213 | 2.038e-06 | 0.0003534 | 0.4706 | -1.087 | -6.591 | 0.0002437 | 0.003429 | 1.603 | 0.6805 | 4.455 | 3.015e-05 | 0.0006961 | 1.764 | 0.8192 | 5.363 |  |
| Kdm3a | lysine (K)-specific demethylas | 0.009216 | 0.08803 | 1.255 | 0.3283 | 2.882 | 0.02306 | 0.2085 | 0.7946 | -0.3317 | -2.461 | 3.89e-08 | 4.084e-06 | 2.101 | 1.071 | 8.585 | 4.835e-07 | 2.728e-05 | 1.877 | 0.9088 | 7.285 |  |
| Mylip | myosin regulatory light chain | 3.145e-06 | 0.0005658 | 2.13 | 1.091 | 6.387 | 1.404e-06 | 0.0002656 | 2.58 | 1.368 | 6.767 | 0.045 | 0.1662 | 1.32 | 0.4001 | 2.139 | 2.102e-06 | 8.735e-05 | 0.4262 | -1.23 | -6.576 |  |
| Igf1r | insulin-like growth factor I r | 4.07e-07 | 0.0001325 | 1.912 | 0.9353 | 7.37 | 0.04344 | 0.2904 | 1.252 | 0.3237 | 2.156 | 0.0008076 | 0.008591 | 1.462 | 0.548 | 3.942 | 1.156e-08 | 1.607e-06 | 2.44 | 1.287 | 9.255 |  |
| Sall1 | spalt like transcription facto | 0.0659 | 0.2732 | 1.206 | 0.2703 | 1.946 | 8.917e-06 | 0.001015 | 1.96 | 0.9711 | 5.908 | 1.657e-06 | 7.151e-05 | 0.4938 | -1.018 | -6.689 | 6.678e-07 | 3.484e-05 | 0.4715 | -1.085 | -7.126 |  |
| Vegfa | vascular endothelial growth fa | 0.001255 | 0.02643 | 0.6386 | -0.6469 | -3.753 | 0.004242 | 0.07896 | 0.6338 | -0.658 | -3.226 | 4.443e-06 | 0.0001572 | 2.259 | 1.176 | 6.227 | 6.506e-06 | 0.0002126 | 0.4529 | -1.143 | -6.051 |  |
| Qtrt1 | queuine tRNA-ribosyltransferas | 0.0005766 | 0.01603 | 1.408 | 0.4934 | 4.086 | 0.1634 | 0.5193 | 0.8665 | -0.2068 | -1.447 | 1.437e-06 | 6.416e-05 | 1.858 | 0.8938 | 6.756 | 1.463e-08 | 1.92e-06 | 2.308 | 1.207 | 9.123 |  |
| Slc20a1 | solute carrier family 20, memb | 4.418e-05 | 0.003316 | 1.769 | 0.823 | 5.195 | 2.444e-07 | 6.759e-05 | 2.693 | 1.429 | 7.626 | 4.945e-09 | 9.218e-07 | 0.3097 | -1.691 | -9.743 | 0.4677 | 0.6879 | 0.9148 | -0.1285 | -0.7403 |  |
| Pik3r1 | phosphoinositide-3-kinase regu | 0.005155 | 0.06205 | 1.463 | 0.5488 | 3.14 | 0.002301 | 0.05276 | 1.649 | 0.722 | 3.492 | 1.002e-07 | 8.561e-06 | 0.3421 | -1.547 | -8.083 | 2.974e-05 | 0.0006882 | 2.039 | 1.028 | 5.369 |  |
| Gclc | glutamate-cysteine ligase, cat | 0.07397 | 0.2908 | 1.279 | 0.3548 | 1.886 | 0.0006391 | 0.02219 | 0.536 | -0.8998 | -4.042 | 2.847e-06 | 0.0001097 | 2.507 | 1.326 | 6.433 | 1.78e-10 | 5.797e-08 | 5.418 | 2.438 | 11.83 |  |
| Slc25a15 | solute carrier family 25 (mito | 0.007009 | 0.07489 | 0.7481 | -0.4188 | -3.005 | 0.03478 | 0.2616 | 1.296 | 0.3736 | 2.265 | 6.239e-07 | 3.481e-05 | 0.4687 | -1.093 | -7.159 | 5.399e-08 | 5.383e-06 | 0.4107 | -1.284 | -8.409 |  |
| Tram2 | translocating chain-associatin | 0.0502 | 0.2356 | 1.298 | 0.3767 | 2.084 | 8.252e-06 | 0.0009713 | 2.413 | 1.271 | 5.943 | 8.027e-08 | 7.271e-06 | 0.3246 | -1.623 | -8.199 | 3.098e-05 | 0.0007103 | 0.4798 | -1.059 | -5.351 |  |
| Col6a3 | collagen, type VI, alpha 3 | 1.567e-07 | 6.496e-05 | 0.3768 | -1.408 | -7.852 | 0.004163 | 0.0781 | 0.6214 | -0.6864 | -3.234 | 0.005282 | 0.03545 | 0.653 | -0.6149 | -3.13 | 1.445e-07 | 1.096e-05 | 0.3413 | -1.551 | -7.894 |  |
| Hmgcs2 | 3-hydroxy-3-methylglutaryl-Coe | 6.372e-07 | 0.0001782 | 5.652 | 2.499 | 7.149 | 0.01253 | 0.1489 | 2.196 | 1.135 | 2.744 | 5.447e-06 | 0.0001858 | 5.091 | 2.348 | 6.133 | 0.0001927 | 0.002899 | 3.35 | 1.744 | 4.556 |  |
| Arrdc3 | arrestin domain containing 3 | 1.983e-07 | 7.502e-05 | 4.082 | 2.029 | 7.731 | 0.9917 | 0.9979 | 1.002 | 0.003273 | 0.01054 | 1.378e-08 | 1.966e-06 | 6.201 | 2.632 | 9.156 | 1.415e-10 | 4.853e-08 | 10.89 | 3.445 | 11.98 |  |
| Sfxn5 | sideroflexin 5 | 1.312e-05 | 0.0015 | 0.5322 | -0.91 | -5.733 | 0.0005627 | 0.02027 | 0.5867 | -0.7693 | -4.096 | 5.631e-06 | 0.0001896 | 0.4784 | -1.064 | -6.118 | 0.001452 | 0.01345 | 1.56 | 0.6417 | 3.69 |  |
| Agpat9 | NA | 0.02846 | 0.1705 | 1.499 | 0.584 | 2.362 | 6.127e-06 | 0.0007841 | 3.431 | 1.779 | 6.079 | 3.816e-08 | 4.039e-06 | 0.1991 | -2.328 | -8.595 | 0.0001604 | 0.002506 | 0.4189 | -1.255 | -4.635 |  |
| Idi1 | isopentenyl-diphosphate delta | 3.843e-07 | 0.0001284 | 0.2427 | -2.043 | -7.399 | 3.096e-08 | 1.171e-05 | 0.1392 | -2.845 | -8.709 | 0.1234 | 0.3235 | 1.401 | 0.4865 | 1.609 | 3.418e-06 | 0.0001247 | 3.784 | 1.92 | 6.348 |  |
| Angptl2 | angiopoietin-like 2 | 3.395e-08 | 2.079e-05 | 0.284 | -1.816 | -8.659 | 0.0004451 | 0.01704 | 0.4858 | -1.042 | -4.197 | 0.09621 | 0.2767 | 0.7573 | -0.4011 | -1.746 | 5.833e-08 | 5.683e-06 | 0.2638 | -1.923 | -8.368 |  |
| Svil | supervillin | 0.6316 | 0.8419 | 0.9579 | -0.06209 | -0.487 | 4.925e-06 | 0.0006761 | 0.5241 | -0.9321 | -6.179 | 9.831e-08 | 8.458e-06 | 2.189 | 1.13 | 8.093 | 1.201e-09 | 2.758e-07 | 2.789 | 1.48 | 10.6 |  |
| Gpd1 | glycerol-3-phosphate dehydroge | 0.0003935 | 0.01265 | 0.5602 | -0.8359 | -4.249 | 0.06785 | 0.3575 | 1.365 | 0.4494 | 1.931 | 2.27e-11 | 1.883e-08 | 0.1376 | -2.861 | -13.28 | 0.0001674 | 0.002591 | 0.5018 | -0.9948 | -4.616 |  |
| Rnf186 | ring finger protein 186 | 0.001466 | 0.02883 | 0.6163 | -0.6982 | -3.686 | 4.044e-06 | 0.0005844 | 0.3776 | -1.405 | -6.27 | 3.888e-05 | 0.0008269 | 2.128 | 1.089 | 5.251 | 7.358e-05 | 0.001403 | 2.044 | 1.032 | 4.972 |  |
| Noct | nocturnin | 0.0001246 | 0.006271 | 2.243 | 1.165 | 4.744 | 1.135e-06 | 0.0002264 | 3.991 | 1.997 | 6.869 | 0.02121 | 0.09827 | 0.6272 | -0.673 | -2.501 | 1.406e-06 | 6.422e-05 | 0.283 | -1.821 | -6.767 |  |
| Bhmt | betaine-homocysteine methyltra | 0.0395 | 0.2058 | 1.344 | 0.4261 | 2.203 | 9.898e-06 | 0.001093 | 2.534 | 1.341 | 5.86 | 2.307e-06 | 9.283e-05 | 0.3831 | -1.384 | -6.532 | 2.901e-06 | 0.0001101 | 0.3892 | -1.361 | -6.425 |  |
| Abca2 | ATP-binding cassette, sub-fami | 0.001128 | 0.02444 | 0.612 | -0.7084 | -3.799 | 1.946e-06 | 0.0003453 | 0.3638 | -1.459 | -6.612 | 0.01126 | 0.06212 | 1.485 | 0.5703 | 2.792 | 7.56e-08 | 6.799e-06 | 3.207 | 1.681 | 8.231 |  |
| Tox | thymocyte selection-associated | 0.001676 | 0.03137 | 1.952 | 0.9647 | 3.629 | 4.268e-06 | 0.0006099 | 3.903 | 1.964 | 6.245 | 2.57e-05 | 0.0006055 | 0.334 | -1.582 | -5.433 | 9.385e-05 | 0.001679 | 0.3745 | -1.417 | -4.866 |  |
| Hmgcs1 | 3-hydroxy-3-methylglutaryl-Coe | 0.005752 | 0.06707 | 0.4629 | -1.111 | -3.092 | 6.283e-06 | 0.0007962 | 0.1672 | -2.58 | -6.067 | 2.74e-06 | 0.0001066 | 5.815 | 2.54 | 6.451 | 0.0001255 | 0.002086 | 3.646 | 1.866 | 4.741 |  |
| LOC101055995 | NA | 0.009796 | 0.09141 | 1.355 | 0.438 | 2.855 | 0.01436 | 0.1603 | 0.7136 | -0.4868 | -2.681 | 6.682e-06 | 0.0002173 | 2.021 | 1.015 | 6.039 | 1.476e-09 | 3.218e-07 | 3.385 | 1.759 | 10.47 |  |
| Ufsp1 | UFM1-specific peptidase 1 | 3.129e-05 | 0.00272 | 0.5731 | -0.8033 | -5.346 | 0.4931 | 0.7833 | 1.09 | 0.1241 | 0.6982 | 6.285e-07 | 3.492e-05 | 0.442 | -1.178 | -7.156 | 4.908e-09 | 8.326e-07 | 0.3289 | -1.604 | -9.748 |  |
| Serpina6 | serine (or cysteine) peptidase | 0.03356 | 0.1879 | 0.7724 | -0.3727 | -2.282 | 8.382e-06 | 0.000973 | 0.4516 | -1.147 | -5.936 | 1.292e-05 | 0.0003569 | 2.037 | 1.027 | 5.74 | 5.261e-07 | 2.909e-05 | 2.455 | 1.295 | 7.243 |  |
| Scd2 | stearoyl-Coenzyme A desaturase | 4.638e-08 | 2.651e-05 | 0.1812 | -2.464 | -8.49 | 0.01194 | 0.1447 | 0.5177 | -0.9498 | -2.765 | 1.27e-10 | 6.248e-08 | 0.07014 | -3.834 | -12.06 | 0.03709 | 0.1435 | 0.6112 | -0.7103 | -2.234 |  |
| Nr4a1 | nuclear receptor subfamily 4, | 9.736e-05 | 0.005433 | 0.3833 | -1.384 | -4.85 | 1.147e-06 | 0.0002269 | 0.2007 | -2.317 | -6.864 | 8.358e-06 | 0.0002575 | 3.618 | 1.855 | 5.937 | 0.006723 | 0.0421 | 1.925 | 0.9447 | 3.023 |  |
| Phlda2 | pleckstrin homology like domai | 0.0008547 | 0.02045 | 0.6553 | -0.6097 | -3.918 | 3.697e-06 | 0.0005466 | 0.4468 | -1.162 | -6.312 | 2.807e-05 | 0.0006467 | 1.892 | 0.9197 | 5.394 | 0.0003266 | 0.004389 | 1.668 | 0.7381 | 4.329 |  |
| Slc25a29 | solute carrier family 25 (mito | 0.005549 | 0.06518 | 1.323 | 0.4034 | 3.108 | 4.686e-06 | 0.0006551 | 1.935 | 0.9526 | 6.202 | 0.0003536 | 0.004582 | 0.6548 | -0.6108 | -4.295 | 4.785e-07 | 2.705e-05 | 0.4875 | -1.037 | -7.29 |  |
| Aqp4 | aquaporin 4 | 0.0002626 | 0.009906 | 0.5707 | -0.8092 | -4.423 | 0.058 | 0.3328 | 1.352 | 0.4354 | 2.011 | 0.0002616 | 0.003618 | 0.5408 | -0.8868 | -4.424 | 6.662e-12 | 5.355e-09 | 0.139 | -2.847 | -14.2 |  |
| Cd276 | CD276 antigen | 1.101e-05 | 0.001362 | 0.5797 | -0.7867 | -5.812 | 3.443e-07 | 8.515e-05 | 0.4372 | -1.194 | -7.453 | 0.0003212 | 0.004256 | 1.562 | 0.6429 | 4.336 | 0.0008942 | 0.00935 | 1.493 | 0.578 | 3.898 |  |
| Nr1i3 | nuclear receptor subfamily 1, | 0.06644 | 0.2744 | 1.255 | 0.3279 | 1.941 | 1.047e-05 | 0.001122 | 2.244 | 1.166 | 5.835 | 6.597e-06 | 0.0002156 | 0.4606 | -1.118 | -6.045 | 2.864e-07 | 1.799e-05 | 0.3799 | -1.396 | -7.546 |  |
| Hmox1 | heme oxygenase 1 | 0.008241 | 0.08239 | 1.61 | 0.6866 | 2.932 | 0.01509 | 0.1648 | 0.6002 | -0.7366 | -2.659 | 1.11e-05 | 0.0003192 | 2.809 | 1.49 | 5.809 | 8.156e-10 | 2.098e-07 | 6.867 | 2.78 | 10.84 |  |
| Tlcd2 | TLC domain containing 2 | 8.551e-06 | 0.001158 | 0.5098 | -0.9721 | -5.927 | 4.408e-06 | 0.0006264 | 0.4325 | -1.209 | -6.23 | 0.03532 | 0.141 | 0.7549 | -0.4056 | -2.258 | 4.558e-06 | 0.0001578 | 2.168 | 1.117 | 6.215 |  |
| Eif4ebp3 | eukaryotic translation initiat | 7.44e-09 | 8.132e-06 | 3.408 | 1.769 | 9.507 | 2.776e-09 | 2.164e-06 | 4.661 | 2.22 | 10.09 | 0.6805 | 0.847 | 1.061 | 0.08518 | 0.4179 | 7.603e-07 | 3.911e-05 | 0.3686 | -1.44 | -7.063 |  |
| Tmc7 | transmembrane channel-like gen | 0.1099 | 0.3596 | 0.7963 | -0.3287 | -1.673 | 0.0002836 | 0.01234 | 2.028 | 1.02 | 4.39 | 7.262e-06 | 0.0002303 | 0.4086 | -1.291 | -6.001 | 4.349e-11 | 2.034e-08 | 0.1481 | -2.755 | -12.81 |  |
| Nrep | neuronal regeneration related | 3.115e-08 | 1.955e-05 | 0.1992 | -2.328 | -8.705 | 0.0001463 | 0.007649 | 0.3587 | -1.479 | -4.674 | 1.988e-05 | 0.0004921 | 0.3242 | -1.625 | -5.547 | 0.005083 | 0.03418 | 0.5278 | -0.9218 | -3.147 |  |
| Ttyh2 | tweety family member 2 | 7.879e-09 | 8.132e-06 | 0.4411 | -1.181 | -9.474 | 9.051e-06 | 0.001019 | 0.547 | -0.8704 | -5.901 | 0.4786 | 0.7133 | 1.071 | 0.0986 | 0.7221 | 2.867e-07 | 1.799e-05 | 0.4896 | -1.03 | -7.545 |  |
| Decr1 | 2,4-dienoyl CoA reductase 1, m | 0.7656 | 0.9075 | 0.9758 | -0.03537 | -0.3023 | 6.905e-06 | 0.0008539 | 1.783 | 0.8339 | 6.024 | 6.507e-11 | 3.893e-08 | 0.3288 | -1.605 | -12.52 | 1.639e-07 | 1.203e-05 | 0.4988 | -1.003 | -7.829 |  |
| Spns2 | spinster homolog 2 | 0.01835 | 0.1317 | 1.329 | 0.4102 | 2.568 | 0.01609 | 0.1701 | 0.7086 | -0.4969 | -2.629 | 1.284e-06 | 6.007e-05 | 2.284 | 1.192 | 6.81 | 2.905e-08 | 3.278e-06 | 2.888 | 1.53 | 8.743 |  |
| Ly6a | lymphocyte antigen 6 complex, | 0.1909 | 0.483 | 0.8577 | -0.2215 | -1.354 | 1.495e-05 | 0.001467 | 0.467 | -1.098 | -5.675 | 9.15e-08 | 8.006e-06 | 2.745 | 1.457 | 8.13 | 2.218e-06 | 9.083e-05 | 2.256 | 1.174 | 6.551 |  |
| Fmn1 | formin 1 | 0.6307 | 0.8412 | 0.9548 | -0.06669 | -0.4882 | 8.397e-06 | 0.000973 | 0.5143 | -0.9593 | -5.935 | 2.392e-09 | 5.294e-07 | 2.873 | 1.523 | 10.17 | 2.028e-07 | 1.414e-05 | 2.227 | 1.155 | 7.72 |  |
| Trp53inp1 | transformation related protein | 5.443e-07 | 0.0001609 | 3.753 | 1.908 | 7.226 | 0.06593 | 0.353 | 1.524 | 0.6077 | 1.945 | 2.443e-09 | 5.294e-07 | 7.669 | 2.939 | 10.16 | 0.003385 | 0.02546 | 1.947 | 0.9615 | 3.324 |  |
| Mfsd7c | NA | 0.001399 | 0.02808 | 1.696 | 0.7621 | 3.706 | 2.628e-06 | 0.0004251 | 2.978 | 1.574 | 6.471 | 0.02163 | 0.0996 | 0.6777 | -0.5612 | -2.492 | 6.308e-08 | 5.988e-06 | 0.2725 | -1.875 | -8.326 |  |
| Acsl3 | acyl-CoA synthetase long-chain | 3.631e-05 | 0.002997 | 0.4579 | -1.127 | -5.281 | 7.721e-07 | 0.0001628 | 0.2909 | -1.781 | -7.055 | 3.818e-05 | 0.000815 | 2.344 | 1.229 | 5.259 | 0.00514 | 0.03447 | 1.664 | 0.7343 | 3.142 |  |
| Acot3 | acyl-CoA thioesterase 3 | 6.949e-05 | 0.004456 | 2.518 | 1.332 | 4.997 | 1.112e-06 | 0.0002253 | 4.5 | 2.17 | 6.879 | 0.008819 | 0.05174 | 0.5557 | -0.8475 | -2.902 | 1.401e-05 | 0.0003842 | 0.3152 | -1.666 | -5.704 |  |
| Slc18b1 | solute carrier family 18, subf | 1.242e-06 | 0.0002919 | 0.4916 | -1.025 | -6.826 | 0.9765 | 0.9927 | 0.9963 | -0.005296 | -0.02982 | 6.554e-09 | 1.116e-06 | 0.3356 | -1.575 | -9.58 | 1.767e-08 | 2.175e-06 | 0.3578 | -1.483 | -9.017 |  |
| Nfkbia | nuclear factor of kappa light | 2.018e-06 | 0.0004254 | 2.23 | 1.157 | 6.595 | 0.06998 | 0.3628 | 1.317 | 0.3974 | 1.915 | 1.795e-07 | 1.338e-05 | 2.82 | 1.496 | 7.782 | 0.0005908 | 0.006858 | 1.721 | 0.7832 | 4.076 |  |
| Arsi | arylsulfatase i | 0.000583 | 0.01616 | 0.7034 | -0.5075 | -4.081 | 0.2353 | 0.5952 | 1.133 | 0.18 | 1.224 | 1.749e-07 | 1.312e-05 | 0.479 | -1.062 | -7.795 | 4.265e-07 | 2.487e-05 | 0.4997 | -1.001 | -7.347 |  |
| Coq7 | demethyl-Q 7 | 0.0008919 | 0.02109 | 0.6922 | -0.5308 | -3.899 | 0.1852 | 0.5464 | 1.166 | 0.221 | 1.372 | 3.354e-08 | 3.687e-06 | 0.4084 | -1.292 | -8.665 | 2.69e-06 | 0.0001042 | 0.5129 | -0.9633 | -6.46 |  |
| Slc25a19 | solute carrier family 25 (mito | 9.828e-07 | 0.0002479 | 0.4039 | -1.308 | -6.939 | 0.03906 | 0.2765 | 0.7108 | -0.4926 | -2.208 | 4.427e-08 | 4.436e-06 | 0.2956 | -1.758 | -8.515 | 0.003339 | 0.0252 | 0.6208 | -0.6877 | -3.33 |  |
| Tifa | TRAF-interacting protein with | 0.4768 | 0.7465 | 0.9287 | -0.1066 | -0.7251 | 1.604e-05 | 0.00154 | 0.5063 | -0.9821 | -5.643 | 1.125e-07 | 9.218e-06 | 2.45 | 1.293 | 8.023 | 6.915e-08 | 6.33e-06 | 2.52 | 1.334 | 8.278 |  |
| Pex11a | peroxisomal biogenesis factor | 0.7854 | 0.9158 | 1.029 | 0.04057 | 0.276 | 6.609e-06 | 0.0008253 | 2.072 | 1.051 | 6.044 | 6.483e-10 | 2.034e-07 | 0.2935 | -1.768 | -10.98 | 1.583e-07 | 1.177e-05 | 0.4165 | -1.263 | -7.847 |  |
| Sec61g | SEC61, gamma subunit | 0.001845 | 0.03331 | 0.7012 | -0.512 | -3.587 | 5.789e-06 | 0.0007521 | 0.4893 | -1.031 | -6.105 | 4.797e-05 | 0.0009718 | 1.749 | 0.8066 | 5.159 | 0.0001095 | 0.001882 | 1.682 | 0.7505 | 4.799 |  |
| Gpr182 | G protein-coupled receptor 182 | 1.825e-06 | 0.0003979 | 2.427 | 1.279 | 6.643 | 0.03215 | 0.2505 | 1.439 | 0.5247 | 2.303 | 5.754e-06 | 0.0001927 | 2.442 | 1.288 | 6.108 | 0.0001656 | 0.00257 | 1.965 | 0.9746 | 4.621 |  |
| Tpi1 | triosephosphate isomerase 1 | 4.855e-05 | 0.003548 | 0.6026 | -0.7307 | -5.153 | 0.1334 | 0.4753 | 1.2 | 0.2625 | 1.565 | 1.826e-12 | 2.935e-09 | 0.1938 | -2.368 | -15.24 | 0.000811 | 0.008714 | 0.6543 | -0.612 | -3.94 |  |
| Egln1 | egl-9 family hypoxia-inducible | 0.2555 | 0.5598 | 0.9193 | -0.1214 | -1.171 | 0.000402 | 0.01584 | 1.434 | 0.5202 | 4.24 | 5.585e-09 | 1.012e-06 | 0.467 | -1.099 | -9.673 | 4.948e-07 | 2.761e-05 | 0.564 | -0.8262 | -7.273 |  |
| Ei24 | etoposide induced 2.4 mRNA | 0.3845 | 0.6774 | 0.9252 | -0.1122 | -0.8891 | 0.0001633 | 0.008255 | 1.614 | 0.6908 | 4.627 | 3.139e-07 | 2.05e-05 | 0.4874 | -1.037 | -7.5 | 3.615e-09 | 6.643e-07 | 0.3862 | -1.372 | -9.928 |  |
| Baiap2 | brain-specific angiogenesis in | 0.03115 | 0.1798 | 1.34 | 0.4222 | 2.318 | 1.357e-05 | 0.001385 | 2.349 | 1.232 | 5.718 | 4.835e-07 | 2.859e-05 | 0.3652 | -1.453 | -7.285 | 8.772e-05 | 0.001589 | 0.5082 | -0.9765 | -4.895 |  |
| Rhbdd2 | rhomboid domain containing 2 | 0.2082 | 0.5049 | 1.143 | 0.1934 | 1.301 | 7.088e-06 | 0.0008641 | 2.082 | 1.058 | 6.012 | 1.274e-05 | 0.0003532 | 0.5227 | -0.9361 | -5.747 | 2.398e-09 | 4.745e-07 | 0.3171 | -1.657 | -10.17 |  |
| Grem2 | gremlin 2, DAN family BMP anta | 0.0002165 | 0.00891 | 0.4281 | -1.224 | -4.506 | 0.08026 | 0.3877 | 0.6633 | -0.5922 | -1.843 | 0.0002452 | 0.003441 | 2.505 | 1.325 | 4.452 | 8.383e-10 | 2.124e-07 | 0.1074 | -3.22 | -10.82 |  |
| Cyp26a1 | cytochrome P450, family 26, su | 0.1289 | 0.3929 | 0.583 | -0.7784 | -1.584 | 1.81e-05 | 0.001687 | 0.1051 | -3.25 | -5.589 | 1.565e-06 | 6.866e-05 | 12.25 | 3.615 | 6.716 | 7.699e-07 | 3.937e-05 | 13.92 | 3.799 | 7.057 |  |
| Acss2 | acyl-CoA synthetase short-chai | 1.299e-06 | 0.0002983 | 0.3373 | -1.568 | -6.805 | 4.073e-07 | 9.7e-05 | 0.2484 | -2.009 | -7.37 | 0.2599 | 0.5115 | 0.8164 | -0.2927 | -1.16 | 3.025e-06 | 0.0001134 | 3.067 | 1.617 | 6.405 |  |
| Adap2 | ArfGAP with dual PH domains 2 | 1.79e-07 | 6.978e-05 | 3.391 | 1.762 | 7.784 | 0.01866 | 0.1864 | 1.609 | 0.6857 | 2.561 | 6.983e-10 | 2.164e-07 | 6.55 | 2.711 | 10.94 | 0.05024 | 0.1769 | 1.431 | 0.5167 | 2.084 |  |
| 2010003K11Rik | RIKEN cDNA 2010003K11 gene | 0.948 | 0.9815 | 0.9898 | -0.01481 | -0.06607 | 6.996e-06 | 0.0008569 | 3.022 | 1.596 | 6.018 | 3.127e-10 | 1.173e-07 | 0.1424 | -2.812 | -11.45 | 7.595e-08 | 6.807e-06 | 0.2466 | -2.02 | -8.228 |  |
| Shank2 | SH3 and multiple ankyrin repea | 0.01172 | 0.1009 | 0.7146 | -0.4848 | -2.774 | 1.026e-05 | 0.001117 | 0.4327 | -1.209 | -5.844 | 0.0001262 | 0.002068 | 1.875 | 0.9072 | 4.738 | 1.987e-06 | 8.429e-05 | 2.402 | 1.264 | 6.602 |  |
| Thbs2 | thrombospondin 2 | 8.855e-09 | 8.761e-06 | 4.667 | 2.223 | 9.407 | 0.06087 | 0.3414 | 1.47 | 0.5554 | 1.986 | 2.588e-13 | 6.237e-10 | 20.85 | 4.382 | 16.93 | 0.02659 | 0.1139 | 1.537 | 0.6197 | 2.394 |  |
| Cacng7 | calcium channel, voltage-depen | 0.01188 | 0.1017 | 0.6059 | -0.7228 | -2.768 | 0.02694 | 0.2267 | 1.668 | 0.7378 | 2.388 | 7.535e-09 | 1.235e-06 | 0.1521 | -2.717 | -9.499 | 1.085e-05 | 0.0003158 | 0.3155 | -1.665 | -5.819 |  |
| Pnpla7 | patatin-like phospholipase dom | 1.502e-08 | 1.246e-05 | 2.635 | 1.398 | 9.108 | 0.9833 | 0.9951 | 1.003 | 0.003844 | 0.02117 | 7.457e-14 | 2.398e-10 | 8.228 | 3.041 | 18.09 | 1.349e-06 | 6.219e-05 | 2.205 | 1.141 | 6.786 |  |
| Frrs1 | ferric-chelate reductase 1 | 0.04057 | 0.2088 | 1.228 | 0.2963 | 2.19 | 0.002999 | 0.06286 | 0.6874 | -0.5407 | -3.377 | 1.621e-05 | 0.0004229 | 1.785 | 0.8357 | 5.638 | 3.946e-10 | 1.137e-07 | 3.194 | 1.675 | 11.3 |  |
| Ttc39c | tetratricopeptide repeat domai | 0.001637 | 0.03079 | 0.6652 | -0.5881 | -3.639 | 6.431e-06 | 0.0008109 | 0.4481 | -1.158 | -6.057 | 0.0001421 | 0.00226 | 1.777 | 0.8298 | 4.687 | 7.316e-05 | 0.001397 | 1.841 | 0.8807 | 4.974 |  |
| Scpep1 | serine carboxypeptidase 1 | 0.07754 | 0.2981 | 1.214 | 0.2792 | 1.861 | 0.004179 | 0.0782 | 0.6718 | -0.5739 | -3.233 | 1.17e-06 | 5.584e-05 | 2.184 | 1.127 | 6.855 | 3.053e-08 | 3.417e-06 | 2.699 | 1.433 | 8.716 |  |
| Treh | trehalase (brush-border membra | 0.2644 | 0.5702 | 1.132 | 0.1783 | 1.148 | 1.981e-05 | 0.001822 | 2.027 | 1.02 | 5.549 | 2.373e-06 | 9.48e-05 | 0.4636 | -1.109 | -6.519 | 6.414e-08 | 6.002e-06 | 0.375 | -1.415 | -8.318 |  |
| Gm25783 | predicted gene, 25783 | 0.004017 | 0.05248 | 1.531 | 0.6143 | 3.25 | 8.342e-06 | 0.000973 | 2.511 | 1.328 | 5.938 | 6.591e-05 | 0.001233 | 0.4865 | -1.039 | -5.02 | 3.308e-05 | 0.0007484 | 0.4659 | -1.102 | -5.322 |  |
| Glrx | glutaredoxin | 0.002814 | 0.04226 | 1.615 | 0.6916 | 3.405 | 4.941e-06 | 0.0006761 | 2.799 | 1.485 | 6.178 | 0.004638 | 0.03225 | 0.6117 | -0.7092 | -3.187 | 3.096e-07 | 1.91e-05 | 0.3142 | -1.67 | -7.507 |  |
| Cyb5b | cytochrome b5 type B | 2.824e-07 | 0.0001009 | 0.4386 | -1.189 | -7.553 | 9.224e-05 | 0.005585 | 0.533 | -0.9078 | -4.874 | 2.113e-06 | 8.628e-05 | 0.4558 | -1.134 | -6.573 | 0.1081 | 0.2918 | 1.223 | 0.2901 | 1.682 |  |
| 1810010H24Rik | RIKEN cDNA 1810010H24 gene | 0.6553 | 0.8541 | 0.945 | -0.08168 | -0.4532 | 1.531e-05 | 0.001485 | 2.31 | 1.208 | 5.664 | 1.057e-06 | 5.169e-05 | 0.3888 | -1.363 | -6.904 | 5.101e-11 | 2.302e-08 | 0.1761 | -2.506 | -12.69 |  |
| Hlcs | holocarboxylase synthetase (bi | 0.02505 | 0.1583 | 0.8194 | -0.2874 | -2.422 | 0.01861 | 0.1861 | 1.283 | 0.3596 | 2.562 | 7.113e-08 | 6.629e-06 | 0.4751 | -1.074 | -8.263 | 1.714e-06 | 7.484e-05 | 0.5483 | -0.8671 | -6.673 |  |
| Prelid2 | PRELI domain containing 2 | 4.283e-06 | 0.0007154 | 1.982 | 0.9872 | 6.244 | 4.984e-06 | 0.0006783 | 2.227 | 1.155 | 6.174 | 0.02853 | 0.1216 | 1.328 | 0.4088 | 2.36 | 3.75e-05 | 0.0008239 | 0.5314 | -0.9122 | -5.266 |  |
| Slc25a10 | solute carrier family 25 (mito | 9.375e-05 | 0.005288 | 0.3731 | -1.422 | -4.867 | 0.1444 | 0.4923 | 1.439 | 0.5254 | 1.519 | 5.702e-12 | 6.667e-09 | 0.04162 | -4.587 | -14.33 | 0.0008411 | 0.008922 | 0.4186 | -1.256 | -3.924 |  |
| Tmem135 | transmembrane protein 135 | 0.005716 | 0.06677 | 0.7828 | -0.3533 | -3.095 | 0.04903 | 0.3067 | 1.217 | 0.2831 | 2.096 | 4.18e-09 | 7.965e-07 | 0.4261 | -1.231 | -9.842 | 2.616e-05 | 0.0006196 | 0.6248 | -0.6785 | -5.425 |  |
| Gbp2b | guanylate binding protein 2b | 0.008946 | 0.0867 | 0.6655 | -0.5875 | -2.896 | 1.195e-05 | 0.00125 | 0.3825 | -1.386 | -5.775 | 3.701e-05 | 0.0007966 | 2.253 | 1.172 | 5.272 | 2.155e-05 | 0.0005331 | 2.337 | 1.225 | 5.511 |  |
| Pcsk4 | proprotein convertase subtilis | 0.05775 | 0.2549 | 1.2 | 0.2636 | 2.013 | 1.417e-05 | 0.00143 | 1.844 | 0.8827 | 5.699 | 4.999e-05 | 0.001001 | 0.5999 | -0.7372 | -5.14 | 1.695e-07 | 1.218e-05 | 0.46 | -1.12 | -7.812 |  |
| Fh1 | fumarate hydratase 1 | 0.07008 | 0.2824 | 0.8493 | -0.2356 | -1.914 | 0.00586 | 0.09576 | 1.365 | 0.4492 | 3.084 | 6.418e-08 | 6.129e-06 | 0.4596 | -1.122 | -8.317 | 1.028e-06 | 5.016e-05 | 0.5238 | -0.9328 | -6.917 |  |
| Zfand4 | zinc finger, AN1-type domain 4 | 0.6831 | 0.8702 | 0.9497 | -0.07448 | -0.4143 | 2.187e-05 | 0.001927 | 0.4441 | -1.171 | -5.505 | 3.836e-08 | 4.044e-06 | 3.232 | 1.692 | 8.592 | 9.501e-08 | 8.094e-06 | 3.026 | 1.597 | 8.111 |  |
| Slc39a14 | solute carrier family 39 (zinc | 5.577e-07 | 0.000163 | 1.935 | 0.9526 | 7.215 | 0.06037 | 0.3396 | 1.241 | 0.311 | 1.991 | 0.007315 | 0.04512 | 1.349 | 0.4318 | 2.986 | 1.877e-09 | 3.894e-07 | 2.814 | 1.493 | 10.32 |  |
| Gm5499 | predicted pseudogene 5499 | 6.173e-07 | 0.0001745 | 0.4915 | -1.025 | -7.165 | 0.004822 | 0.08595 | 0.6895 | -0.5365 | -3.17 | 0.001141 | 0.0113 | 0.6623 | -0.5944 | -3.794 | 1.949e-05 | 0.0004943 | 0.5469 | -0.8706 | -5.556 |  |
| Mapkap1 | mitogen-activated protein kina | 0.0004113 | 0.01303 | 0.6958 | -0.5232 | -4.231 | 0.3171 | 0.6665 | 1.11 | 0.1502 | 1.026 | 5.683e-08 | 5.558e-06 | 0.4552 | -1.136 | -8.382 | 2.32e-06 | 9.414e-05 | 0.5416 | -0.8846 | -6.529 |  |
| Dsg1a | desmoglein 1 alpha | 0.02095 | 0.1418 | 0.7686 | -0.3796 | -2.507 | 1.683e-05 | 0.001607 | 0.4974 | -1.007 | -5.622 | 1.011e-05 | 0.0002956 | 1.96 | 0.9706 | 5.851 | 2.204e-05 | 0.0005419 | 1.883 | 0.9127 | 5.502 |  |
| Gstm6 | glutathione S-transferase, mu | 8.483e-05 | 0.004959 | 0.5043 | -0.9877 | -4.91 | 1.84e-06 | 0.000331 | 0.3344 | -1.58 | -6.639 | 0.1956 | 0.4304 | 1.227 | 0.2951 | 1.339 | 1.53e-06 | 6.842e-05 | 2.794 | 1.482 | 6.727 |  |
| Ly6c1 | lymphocyte antigen 6 complex, | 0.07137 | 0.2853 | 1.257 | 0.3303 | 1.904 | 0.005296 | 0.08999 | 0.6408 | -0.642 | -3.129 | 1.815e-08 | 2.455e-06 | 3.273 | 1.71 | 9.002 | 3.401e-06 | 0.0001243 | 2.308 | 1.207 | 6.351 |  |
| Ahcy | S-adenosylhomocysteine hydrola | 2.512e-06 | 0.0005009 | 1.998 | 0.9984 | 6.492 | 0.04251 | 0.288 | 1.314 | 0.3943 | 2.167 | 5.341e-07 | 3.096e-05 | 2.328 | 1.219 | 7.236 | 0.002146 | 0.01813 | 1.509 | 0.5933 | 3.522 |  |
| Gm9826 | predicted gene 9826 | 2.36e-06 | 0.0004856 | 1.92 | 0.9408 | 6.521 | 0.4374 | 0.7509 | 1.098 | 0.1353 | 0.7924 | 1.84e-09 | 4.303e-07 | 3.101 | 1.633 | 10.33 | 7.588e-05 | 0.001431 | 1.721 | 0.7836 | 4.958 |  |
| Ucp2 | uncoupling protein 2 (mitochon | 2.507e-06 | 0.0005009 | 3.639 | 1.863 | 6.493 | 0.03501 | 0.2628 | 1.703 | 0.7681 | 2.262 | 0.0002161 | 0.003133 | 2.67 | 1.417 | 4.506 | 8.197e-06 | 0.0002516 | 3.654 | 1.869 | 5.946 |  |
| Plin5 | perilipin 5 | 3.031e-09 | 3.899e-06 | 3.959 | 1.985 | 10.03 | 3.944e-09 | 2.898e-06 | 4.967 | 2.312 | 9.876 | 1.945e-05 | 0.0004834 | 0.4339 | -1.205 | -5.557 | 0.3157 | 0.5564 | 1.167 | 0.2231 | 1.029 |  |
| Nucks1 | nuclear casein kinase and cycl | 0.3492 | 0.6467 | 0.9314 | -0.1025 | -0.9587 | 0.0004229 | 0.01646 | 1.448 | 0.5339 | 4.219 | 2.753e-08 | 3.233e-06 | 0.4904 | -1.028 | -8.773 | 2.998e-07 | 1.858e-05 | 0.5428 | -0.8814 | -7.523 |  |
| Gprc5c | G protein-coupled receptor, fa | 4.038e-06 | 0.0006924 | 0.5186 | -0.9472 | -6.271 | 0.01246 | 0.1483 | 0.7116 | -0.4908 | -2.746 | 0.06939 | 0.2223 | 1.246 | 0.3175 | 1.919 | 2.317e-09 | 4.681e-07 | 0.3106 | -1.687 | -10.19 |  |
| Cyp2c38 | cytochrome P450, family 2, sub | 0.08622 | 0.3152 | 1.332 | 0.4133 | 1.805 | 2.423e-05 | 0.002099 | 2.788 | 1.479 | 5.459 | 3.497e-06 | 0.0001281 | 0.3322 | -1.59 | -6.338 | 2.99e-06 | 0.0001125 | 0.328 | -1.608 | -6.41 |  |
| 1810011O10Rik | NA | 0.0005156 | 0.01503 | 2.303 | 1.204 | 4.134 | 4.53e-06 | 0.0006368 | 4.414 | 2.142 | 6.218 | 0.005448 | 0.03636 | 0.5021 | -0.9939 | -3.116 | 1.381e-05 | 0.0003803 | 0.2829 | -1.821 | -5.71 |  |
| Foxq1 | forkhead box Q1 | 0.04453 | 0.2202 | 0.539 | -0.8918 | -2.144 | 2.204e-05 | 0.001928 | 0.1531 | -2.708 | -5.502 | 6.852e-06 | 0.0002206 | 6.711 | 2.747 | 6.028 | 8.523e-06 | 0.0002598 | 6.504 | 2.701 | 5.928 |  |
| Fbxl3 | F-box and leucine-rich repeat | 0.004695 | 0.05823 | 1.348 | 0.4312 | 3.182 | 0.06983 | 0.3627 | 0.8082 | -0.3072 | -1.916 | 9.134e-06 | 0.0002729 | 1.835 | 0.8755 | 5.897 | 1.747e-08 | 2.175e-06 | 2.531 | 1.34 | 9.024 |  |
| Abca5 | ATP-binding cassette, sub-fami | 0.0001143 | 0.005937 | 1.512 | 0.5964 | 4.781 | 1.969e-06 | 0.0003469 | 1.966 | 0.9751 | 6.607 | 0.5161 | 0.7419 | 0.9393 | -0.09033 | -0.6611 | 2.31e-07 | 1.567e-05 | 0.4844 | -1.046 | -7.654 |  |
| Ucp3 | uncoupling protein 3 (mitochon | 4.339e-05 | 0.003302 | 1.965 | 0.9742 | 5.202 | 0.6143 | 0.8475 | 0.9244 | -0.1135 | -0.512 | 1.881e-06 | 7.908e-05 | 2.566 | 1.36 | 6.628 | 1.11e-08 | 1.56e-06 | 3.741 | 1.903 | 9.278 |  |
| Cpt1a | carnitine palmitoyltransferase | 4.398e-05 | 0.003316 | 1.809 | 0.855 | 5.196 | 1.272e-06 | 0.0002442 | 2.508 | 1.327 | 6.814 | 1.147e-07 | 9.346e-06 | 0.3675 | -1.444 | -8.013 | 0.8724 | 0.9421 | 1.021 | 0.02932 | 0.1627 |  |
| Pi4k2a | phosphatidylinositol 4-kinase | 0.1046 | 0.3495 | 1.17 | 0.2261 | 1.7 | 0.0003753 | 0.01503 | 0.6277 | -0.6718 | -4.27 | 4.695e-05 | 0.0009582 | 1.685 | 0.7528 | 5.168 | 2.129e-11 | 1.428e-08 | 3.839 | 1.941 | 13.32 |  |
| D230025D16Rik | RIKEN cDNA D230025D16 gene | 2.662e-06 | 0.0005227 | 2.386 | 1.255 | 6.465 | 0.8495 | 0.95 | 1.031 | 0.04413 | 0.1922 | 1.68e-06 | 7.216e-05 | 2.677 | 1.42 | 6.682 | 1.65e-09 | 3.478e-07 | 4.63 | 2.211 | 10.4 |  |
| Klhl38 | kelch-like 38 | 8.108e-07 | 0.0002172 | 2.004 | 1.003 | 7.032 | 0.01588 | 0.1691 | 1.361 | 0.4446 | 2.635 | 0.1591 | 0.3793 | 0.8535 | -0.2285 | -1.463 | 1.059e-10 | 4.004e-08 | 3.739 | 1.903 | 12.18 |  |
| Slc1a4 | solute carrier family 1 (gluta | 0.02202 | 0.1464 | 0.7257 | -0.4625 | -2.483 | 2.054e-05 | 0.001873 | 0.4295 | -1.219 | -5.533 | 4.844e-06 | 0.0001682 | 2.399 | 1.262 | 6.187 | 7.893e-05 | 0.001466 | 2.011 | 1.008 | 4.941 |  |
| Lpgat1 | lysophosphatidylglycerol acylt | 0.02445 | 0.1558 | 0.6957 | -0.5235 | -2.434 | 0.01014 | 0.1319 | 1.65 | 0.7226 | 2.839 | 7.56e-10 | 2.261e-07 | 0.169 | -2.565 | -10.89 | 7.211e-05 | 0.001382 | 0.4434 | -1.173 | -4.981 |  |
| Xkr9 | X-linked Kx blood group relate | 0.01332 | 0.1091 | 0.7511 | -0.4129 | -2.716 | 1.772e-05 | 0.001664 | 0.4975 | -1.007 | -5.599 | 2.587e-05 | 0.0006077 | 1.872 | 0.9044 | 5.43 | 3.69e-05 | 0.0008155 | 1.838 | 0.8783 | 5.274 |  |
| Ifngr2 | interferon gamma receptor 2 | 0.01069 | 0.09611 | 0.7838 | -0.3515 | -2.815 | 0.05274 | 0.3175 | 1.235 | 0.3042 | 2.059 | 3.491e-06 | 0.0001281 | 0.5484 | -0.8668 | -6.338 | 1.078e-07 | 8.919e-06 | 0.4665 | -1.1 | -8.045 |  |
| F11 | coagulation factor XI | 0.001506 | 0.02933 | 0.6455 | -0.6316 | -3.675 | 8.447e-06 | 0.0009743 | 0.4333 | -1.206 | -5.932 | 2.075e-05 | 0.0005108 | 2.058 | 1.041 | 5.528 | 0.001664 | 0.0149 | 1.606 | 0.6838 | 3.632 |  |
| Ulk1 | unc-51 like kinase 1 | 1.102e-06 | 0.0002692 | 1.876 | 0.908 | 6.884 | 0.9571 | 0.9864 | 1.006 | 0.008493 | 0.05441 | 1.392e-06 | 6.249e-05 | 1.97 | 0.9785 | 6.771 | 1.592e-10 | 5.295e-08 | 3.294 | 1.72 | 11.9 |  |
| Kcnn2 | potassium intermediate/small c | 0.1248 | 0.3857 | 0.8333 | -0.2631 | -1.602 | 0.0001622 | 0.00823 | 1.866 | 0.8996 | 4.63 | 8.468e-05 | 0.001507 | 0.5421 | -0.8834 | -4.911 | 4.697e-12 | 4.027e-09 | 0.1644 | -2.605 | -14.48 |  |
| Gfra1 | glial cell line derived neurot | 0.4361 | 0.7168 | 1.109 | 0.1491 | 0.7948 | 3.777e-05 | 0.002927 | 2.248 | 1.169 | 5.263 | 2.095e-07 | 1.505e-05 | 0.3337 | -1.584 | -7.704 | 6.042e-07 | 3.238e-05 | 0.3597 | -1.475 | -7.175 |  |
| Cblb | Casitas B-lineage lymphoma b | 9.64e-08 | 4.592e-05 | 2.282 | 1.19 | 8.103 | 0.0002378 | 0.01098 | 1.712 | 0.776 | 4.465 | 0.09129 | 0.2675 | 1.219 | 0.2855 | 1.774 | 8.157e-06 | 0.0002513 | 1.941 | 0.9571 | 5.948 |  |
| Syde2 | synapse defective 1, Rho GTPas | 5.332e-07 | 0.0001595 | 2.756 | 1.463 | 7.237 | 0.005352 | 0.09057 | 1.678 | 0.7471 | 3.124 | 1.268e-09 | 3.136e-07 | 5.058 | 2.338 | 10.56 | 0.3965 | 0.6287 | 0.8755 | -0.1918 | -0.8665 |  |
| Cerkl | ceramide kinase-like | 0.005904 | 0.06825 | 1.545 | 0.6276 | 3.081 | 1.382e-05 | 0.001399 | 2.596 | 1.376 | 5.71 | 0.0001308 | 0.002123 | 0.4817 | -1.054 | -4.723 | 3.148e-05 | 0.0007191 | 0.4375 | -1.193 | -5.344 |  |
| Tead1 | TEA domain family member 1 | 0.06448 | 0.2702 | 1.277 | 0.353 | 1.957 | 0.06439 | 0.3485 | 1.336 | 0.4178 | 1.958 | 1.103e-07 | 9.121e-06 | 3.005 | 1.587 | 8.033 | 1.767e-08 | 2.175e-06 | 0.2909 | -1.782 | -9.017 |  |
| Slc43a1 | solute carrier family 43, memb | 4.236e-08 | 2.476e-05 | 3.163 | 1.661 | 8.539 | 0.6241 | 0.8524 | 1.083 | 0.1146 | 0.4977 | 5.427e-05 | 0.001067 | 2.126 | 1.088 | 5.104 | 3.467e-13 | 5.498e-10 | 11.73 | 3.553 | 16.67 |  |
| Fam198a | NA | 0.03521 | 0.1929 | 0.703 | -0.5084 | -2.259 | 2.49e-05 | 0.002142 | 0.3659 | -1.45 | -5.447 | 1.163e-05 | 0.000331 | 2.688 | 1.427 | 5.787 | 1.487e-05 | 0.0004023 | 2.638 | 1.399 | 5.677 |  |
| Sfxn2 | sideroflexin 2 | 0.5234 | 0.7773 | 1.09 | 0.1246 | 0.6495 | 3.956e-05 | 0.002984 | 2.282 | 1.19 | 5.243 | 7.001e-07 | 3.799e-05 | 0.3553 | -1.493 | -7.103 | 9.562e-08 | 8.094e-06 | 0.307 | -1.704 | -8.107 |  |
| Foxk1 | forkhead box K1 | 0.004213 | 0.05402 | 1.371 | 0.4548 | 3.229 | 0.02678 | 0.2257 | 0.7587 | -0.3985 | -2.391 | 0.000237 | 0.003367 | 1.612 | 0.6892 | 4.467 | 1.152e-10 | 4.175e-08 | 3.657 | 1.871 | 12.12 |  |
| Acsm3 | acyl-CoA synthetase medium-cha | 1.508e-07 | 6.496e-05 | 3.5 | 1.807 | 7.871 | 0.0006716 | 0.02291 | 2.132 | 1.092 | 4.021 | 1.789e-07 | 1.338e-05 | 3.884 | 1.958 | 7.784 | 0.4655 | 0.686 | 1.139 | 0.1871 | 0.7441 |  |
| Rbm3 | RNA binding motif (RNP1, RRM) | 0.006398 | 0.07131 | 1.442 | 0.5281 | 3.045 | 1.492e-05 | 0.001467 | 2.242 | 1.165 | 5.676 | 0.00014 | 0.002232 | 0.539 | -0.8916 | -4.693 | 3.046e-05 | 0.000702 | 0.4938 | -1.018 | -5.358 |  |
| Tmem50b | transmembrane protein 50B | 0.2642 | 0.57 | 1.119 | 0.1622 | 1.149 | 3.037e-05 | 0.00248 | 1.86 | 0.8955 | 5.36 | 3.013e-08 | 3.4e-06 | 0.3924 | -1.349 | -8.723 | 2.225e-05 | 0.0005467 | 0.5546 | -0.8504 | -5.497 |  |
| Cyp2d22 | cytochrome P450, family 2, sub | 3.7e-07 | 0.0001252 | 2.25 | 1.17 | 7.417 | 0.2887 | 0.6441 | 1.151 | 0.2034 | 1.09 | 2.189e-11 | 1.883e-08 | 4.919 | 2.298 | 13.3 | 0.002379 | 0.01954 | 1.516 | 0.6007 | 3.477 |  |
| Gk | glycerol kinase | 0.09778 | 0.3377 | 1.247 | 0.319 | 1.737 | 1.107e-05 | 0.001177 | 2.399 | 1.262 | 5.81 | 2.47e-09 | 5.294e-07 | 0.2427 | -2.043 | -10.16 | 0.0008629 | 0.009101 | 0.5795 | -0.7872 | -3.913 |  |
| Gpc1 | glypican 1 | 0.1447 | 0.4181 | 0.8056 | -0.3118 | -1.518 | 0.002851 | 0.06079 | 1.773 | 0.8262 | 3.399 | 2.103e-08 | 2.665e-06 | 0.2487 | -2.008 | -8.921 | 4.221e-06 | 0.0001492 | 0.3772 | -1.407 | -6.25 |  |
| Bach1 | BTB and CNC homology 1, basic | 0.1373 | 0.4068 | 1.161 | 0.2152 | 1.548 | 0.002786 | 0.05982 | 0.6779 | -0.5608 | -3.409 | 3.251e-06 | 0.0001217 | 1.959 | 0.9704 | 6.371 | 2.125e-08 | 2.543e-06 | 2.563 | 1.358 | 8.915 |  |
| Nnmt | nicotinamide N-methyltransfera | 0.01158 | 0.1002 | 0.5631 | -0.8286 | -2.78 | 0.06285 | 0.3464 | 1.619 | 0.6949 | 1.97 | 1.922e-07 | 1.413e-05 | 0.1731 | -2.53 | -7.747 | 4.238e-06 | 0.0001495 | 0.2431 | -2.041 | -6.248 |  |
| Grtp1 | GH regulated TBC protein 1 | 9.815e-06 | 0.001289 | 0.4666 | -1.1 | -5.864 | 0.8883 | 0.962 | 0.9784 | -0.03158 | -0.1423 | 4.715e-07 | 2.82e-05 | 0.3538 | -1.499 | -7.297 | 6.61e-08 | 6.117e-06 | 0.3067 | -1.705 | -8.302 |  |
| Trabd2b | TraB domain containing 2B | 3.521e-06 | 0.0006247 | 0.3749 | -1.416 | -6.334 | 0.5376 | 0.8065 | 1.122 | 0.1658 | 0.6272 | 3.1e-12 | 4.257e-09 | 0.08101 | -3.626 | -14.81 | 0.0001868 | 0.002831 | 0.4606 | -1.119 | -4.569 |  |
| Mtss1 | MTSS I-BAR domain containing 1 | 0.8816 | 0.9558 | 1.015 | 0.02143 | 0.1509 | 3.202e-05 | 0.002575 | 1.862 | 0.897 | 5.336 | 7.055e-09 | 1.176e-06 | 0.3574 | -1.484 | -9.537 | 4.32e-07 | 2.508e-05 | 0.453 | -1.142 | -7.341 |  |
| Col6a2 | collagen, type VI, alpha 2 | 9.431e-07 | 0.0002426 | 0.3902 | -1.358 | -6.958 | 0.004463 | 0.08159 | 0.5989 | -0.7396 | -3.204 | 0.0009646 | 0.00987 | 0.564 | -0.8262 | -3.866 | 0.0001069 | 0.001847 | 0.4904 | -1.028 | -4.81 |  |
| Vwa3b | von Willebrand factor A domain | 0.9199 | 0.9711 | 1.008 | 0.01209 | 0.1018 | 2.503e-05 | 0.002147 | 1.7 | 0.7652 | 5.445 | 4.86e-07 | 2.867e-05 | 0.5185 | -0.9475 | -7.282 | 2.788e-09 | 5.392e-07 | 0.4028 | -1.312 | -10.08 |  |
| Bace1 | beta-site APP cleaving enzyme | 9.645e-05 | 0.005393 | 0.5148 | -0.958 | -4.854 | 0.5058 | 0.7901 | 1.116 | 0.1582 | 0.6776 | 1.015e-05 | 0.0002963 | 0.4163 | -1.264 | -5.849 | 5.901e-09 | 9.73e-07 | 0.2358 | -2.084 | -9.641 |  |
| Gga2 | golgi associated, gamma adapti | 0.3211 | 0.6219 | 1.082 | 0.1139 | 1.017 | 3.942e-06 | 0.0005729 | 1.78 | 0.8322 | 6.282 | 2.991e-11 | 2.253e-08 | 0.329 | -1.604 | -13.08 | 0.0002223 | 0.003253 | 0.6824 | -0.5512 | -4.494 |  |
| Gpt | glutamic pyruvic transaminase, | 2.917e-08 | 1.876e-05 | 0.3435 | -1.542 | -8.741 | 0.4891 | 0.7804 | 1.107 | 0.1471 | 0.7048 | 1.394e-15 | 2.065e-11 | 0.05056 | -4.306 | -22.29 | 0.0008165 | 0.008758 | 0.5902 | -0.7607 | -3.937 |  |
| Bhmt2 | betaine-homocysteine methyltra | 0.5053 | 0.7644 | 1.069 | 0.09636 | 0.6784 | 4.714e-05 | 0.003375 | 1.825 | 0.8683 | 5.166 | 1.374e-06 | 6.199e-05 | 0.4814 | -1.055 | -6.778 | 9.614e-08 | 8.094e-06 | 0.4172 | -1.261 | -8.105 |  |
| Srxn1 | sulfiredoxin 1 homolog (S. cer | 8.89e-05 | 0.005087 | 1.863 | 0.8973 | 4.89 | 0.03592 | 0.2661 | 1.403 | 0.4884 | 2.249 | 0.008025 | 0.0483 | 0.6635 | -0.5919 | -2.944 | 1.677e-08 | 2.115e-06 | 3.527 | 1.819 | 9.046 |  |
| Tbc1d15 | TBC1 domain family, member 15 | 1.288e-05 | 0.001493 | 1.777 | 0.8291 | 5.741 | 0.4607 | 0.765 | 1.093 | 0.1285 | 0.7522 | 6.61e-06 | 0.0002158 | 1.94 | 0.9561 | 6.044 | 5.271e-07 | 2.909e-05 | 2.212 | 1.146 | 7.242 |  |
| Baiap2l1 | BAI1-associated protein 2-like | 0.02228 | 0.1473 | 1.434 | 0.5199 | 2.478 | 2.72e-05 | 0.002279 | 2.537 | 1.343 | 5.408 | 3.255e-05 | 0.0007306 | 0.4278 | -1.225 | -5.329 | 2.706e-05 | 0.0006363 | 0.4223 | -1.244 | -5.41 |  |
| Gm13244 | predicted gene 13244 | 7.752e-06 | 0.001072 | 0.5994 | -0.7384 | -5.971 | 0.4927 | 0.783 | 0.9316 | -0.1022 | -0.6988 | 1.146e-08 | 1.685e-06 | 0.4192 | -1.254 | -9.26 | 0.0001023 | 0.001786 | 0.6355 | -0.6541 | -4.829 |  |
| Gpd2 | glycerol phosphate dehydrogena | 0.007974 | 0.08069 | 0.6345 | -0.6564 | -2.947 | 0.06369 | 0.3473 | 1.431 | 0.5174 | 1.963 | 8.87e-09 | 1.391e-06 | 0.2038 | -2.295 | -9.406 | 6.48e-05 | 0.001276 | 0.4274 | -1.227 | -5.027 |  |
| Acot4 | acyl-CoA thioesterase 4 | 1.182e-05 | 0.001434 | 2.774 | 1.472 | 5.78 | 1.291e-05 | 0.001328 | 3.316 | 1.73 | 5.741 | 0.0502 | 0.1789 | 1.496 | 0.5813 | 2.084 | 9.611e-05 | 0.00171 | 0.3911 | -1.354 | -4.856 |  |
| Rtn4ip1 | reticulon 4 interacting protei | 0.0141 | 0.1123 | 0.7169 | -0.4801 | -2.69 | 0.05844 | 0.3342 | 1.342 | 0.4239 | 2.007 | 1.805e-06 | 7.647e-05 | 0.4062 | -1.3 | -6.648 | 5.488e-07 | 2.991e-05 | 0.3758 | -1.412 | -7.222 |  |
| Wdr6 | WD repeat domain 6 | 0.0001181 | 0.006076 | 0.6148 | -0.7018 | -4.767 | 3.68e-06 | 0.0005466 | 0.4665 | -1.1 | -6.314 | 0.002374 | 0.01964 | 1.475 | 0.561 | 3.478 | 0.0008371 | 0.008905 | 1.551 | 0.6333 | 3.926 |  |
| H2-Q10 | histocompatibility 2, Q region | 0.04117 | 0.2108 | 0.7815 | -0.3557 | -2.183 | 3.622e-05 | 0.002849 | 0.4937 | -1.018 | -5.282 | 5.301e-06 | 0.0001816 | 2.139 | 1.097 | 6.145 | 7.019e-05 | 0.001356 | 1.855 | 0.8912 | 4.992 |  |
| Slc41a2 | solute carrier family 41, memb | 0.1595 | 0.4387 | 0.7508 | -0.4136 | -1.461 | 4.826e-05 | 0.003412 | 0.3022 | -1.727 | -5.156 | 3.12e-06 | 0.0001178 | 3.949 | 1.981 | 6.391 | 3.675e-06 | 0.0001326 | 3.884 | 1.958 | 6.314 |  |
| Tmbim1 | transmembrane BAX inhibitor mo | 4.195e-06 | 0.0007052 | 1.93 | 0.9485 | 6.253 | 0.75 | 0.9081 | 1.041 | 0.05799 | 0.3231 | 9.747e-10 | 2.639e-07 | 3.439 | 1.782 | 10.73 | 3.085e-05 | 0.0007089 | 1.852 | 0.8894 | 5.353 |  |
| Trp53inp2 | transformation related protein | 0.04502 | 0.2215 | 0.7193 | -0.4754 | -2.138 | 0.019 | 0.1882 | 1.593 | 0.6714 | 2.552 | 2.014e-07 | 1.458e-05 | 0.2715 | -1.881 | -7.724 | 3.038e-06 | 0.0001136 | 0.3393 | -1.559 | -6.403 |  |
| Tbcb | tubulin folding cofactor B | 0.0004034 | 0.01287 | 0.6926 | -0.5299 | -4.239 | 0.3878 | 0.7176 | 1.095 | 0.1306 | 0.8829 | 2.028e-08 | 2.621e-06 | 0.428 | -1.224 | -8.941 | 2.074e-05 | 0.0005206 | 0.5917 | -0.757 | -5.528 |  |
| Slc7a6 | solute carrier family 7 (catio | 5.86e-05 | 0.003967 | 1.856 | 0.8923 | 5.071 | 0.0117 | 0.1431 | 1.493 | 0.5777 | 2.775 | 0.01674 | 0.08283 | 0.7055 | -0.5032 | -2.611 | 3.096e-07 | 1.91e-05 | 2.726 | 1.447 | 7.506 |  |
| Gm4737 | predicted gene 4737 | 1.304e-05 | 0.001498 | 1.796 | 0.8444 | 5.736 | 0.3009 | 0.6548 | 1.137 | 0.185 | 1.062 | 1.954e-07 | 1.426e-05 | 2.375 | 1.248 | 7.739 | 0.0002055 | 0.003047 | 1.659 | 0.7302 | 4.528 |  |
| P2rx4 | purinergic receptor P2X, ligan | 0.1478 | 0.4225 | 1.174 | 0.232 | 1.506 | 0.004151 | 0.07806 | 0.6644 | -0.5898 | -3.235 | 1.457e-06 | 6.498e-05 | 2.203 | 1.139 | 6.75 | 1.549e-07 | 1.158e-05 | 2.507 | 1.326 | 7.858 |  |
| Foxi1 | forkhead box I1 | 0.008903 | 0.08649 | 1.494 | 0.5792 | 2.898 | 2.081e-05 | 0.001876 | 2.475 | 1.307 | 5.527 | 0.0006779 | 0.007549 | 0.5435 | -0.8795 | -4.017 | 8.008e-06 | 0.0002476 | 0.4049 | -1.304 | -5.957 |  |
| Slc25a47 | solute carrier family 25, memb | 0.2108 | 0.5084 | 1.184 | 0.244 | 1.293 | 2.143e-05 | 0.001901 | 2.348 | 1.231 | 5.514 | 0.0001126 | 0.00189 | 0.5036 | -0.9898 | -4.787 | 7.034e-09 | 1.122e-06 | 0.2549 | -1.972 | -9.539 |  |
| Bet1l | Bet1 golgi vesicular membrane | 0.0003577 | 0.012 | 1.412 | 0.4976 | 4.29 | 5.59e-06 | 0.0007374 | 1.79 | 0.84 | 6.121 | 7.169e-08 | 6.64e-06 | 0.4832 | -1.049 | -8.259 | 0.8634 | 0.9374 | 1.015 | 0.02215 | 0.1743 |  |
| Gm6781 | predicted gene 6781 | 0.57 | 0.8068 | 0.9079 | -0.1394 | -0.5776 | 4.44e-05 | 0.003245 | 0.3577 | -1.483 | -5.192 | 3.504e-06 | 0.0001282 | 3.195 | 1.676 | 6.337 | 2.157e-08 | 2.569e-06 | 5.117 | 2.355 | 8.907 |  |
| Tmco6 | transmembrane and coiled-coil | 0.2903 | 0.5941 | 1.126 | 0.1711 | 1.086 | 0.0001543 | 0.007954 | 0.5483 | -0.8669 | -4.651 | 4e-05 | 0.0008455 | 1.871 | 0.9038 | 5.238 | 4.33e-11 | 2.034e-08 | 4.627 | 2.21 | 12.81 |  |
| Hspa12a | heat shock protein 12A | 0.0001541 | 0.007193 | 0.4619 | -1.114 | -4.652 | 0.3725 | 0.708 | 1.196 | 0.2586 | 0.9123 | 3.926e-10 | 1.383e-07 | 0.1279 | -2.966 | -11.31 | 0.000283 | 0.00393 | 0.45 | -1.152 | -4.391 |  |
| Lipg | lipase, endothelial | 0.001568 | 0.02997 | 2.006 | 1.004 | 3.657 | 1.243e-05 | 0.001289 | 3.657 | 1.871 | 5.758 | 0.0001229 | 0.002025 | 0.3715 | -1.429 | -4.749 | 0.0009056 | 0.009454 | 0.4441 | -1.171 | -3.893 |  |
| Cyp4a14 | cytochrome P450, family 4, sub | 0.08592 | 0.3148 | 1.401 | 0.4862 | 1.807 | 4.751e-05 | 0.003376 | 3.125 | 1.644 | 5.163 | 4.986e-06 | 0.0001721 | 0.2832 | -1.82 | -6.173 | 1.734e-05 | 0.0004525 | 0.3179 | -1.653 | -5.608 |  |
| Shroom3 | shroom family member 3 | 0.1358 | 0.405 | 1.193 | 0.2546 | 1.554 | 0.002461 | 0.055 | 0.628 | -0.6711 | -3.463 | 1.995e-05 | 0.0004926 | 1.993 | 0.995 | 5.546 | 3.59e-09 | 6.643e-07 | 3.439 | 1.782 | 9.932 |  |
| Fam63a | NA | 0.01702 | 0.1262 | 1.25 | 0.3218 | 2.603 | 0.02675 | 0.2257 | 0.7847 | -0.3498 | -2.391 | 6.036e-05 | 0.001158 | 1.608 | 0.685 | 5.058 | 4.344e-09 | 7.601e-07 | 2.514 | 1.33 | 9.82 |  |
| Slc25a37 | solute carrier family 25, memb | 0.2282 | 0.5287 | 0.8395 | -0.2524 | -1.243 | 1.735e-05 | 0.001646 | 0.393 | -1.347 | -5.608 | 1.217e-09 | 3.089e-07 | 5.117 | 2.355 | 10.59 | 0.0004619 | 0.005672 | 1.905 | 0.93 | 4.181 |  |
| Rgs2 | regulator of G-protein signali | 3.118e-05 | 0.002719 | 3.476 | 1.797 | 5.348 | 0.7021 | 0.8887 | 0.8986 | -0.1543 | -0.3881 | 1.53e-09 | 3.678e-07 | 14.38 | 3.846 | 10.45 | 2.437e-05 | 0.0005877 | 4.025 | 2.009 | 5.457 |  |
| Agpat3 | 1-acylglycerol-3-phosphate O-a | 0.03361 | 0.188 | 1.205 | 0.2688 | 2.282 | 2.565e-05 | 0.002177 | 1.691 | 0.7576 | 5.434 | 1.508e-07 | 1.162e-05 | 0.4945 | -1.016 | -7.871 | 0.002316 | 0.01912 | 0.7319 | -0.4503 | -3.489 |  |
| Adam11 | a disintegrin and metallopepti | 0.4881 | 0.753 | 0.8975 | -0.1561 | -0.7063 | 5.411e-05 | 0.00374 | 0.3964 | -1.335 | -5.106 | 5.57e-06 | 0.0001878 | 2.793 | 1.482 | 6.123 | 4.659e-08 | 4.813e-06 | 4.154 | 2.054 | 8.488 |  |
| Prpf6 | pre-mRNA splicing factor 6 | 0.01761 | 0.1285 | 1.278 | 0.354 | 2.587 | 0.05192 | 0.3155 | 0.793 | -0.3346 | -2.067 | 5.686e-06 | 0.0001909 | 1.887 | 0.9161 | 6.113 | 2.232e-07 | 1.53e-05 | 2.219 | 1.15 | 7.671 |  |
| Rnf19a | ring finger protein 19A | 0.007821 | 0.07971 | 1.278 | 0.3544 | 2.956 | 1.464e-05 | 0.001455 | 1.749 | 0.8064 | 5.684 | 1.189e-07 | 9.53e-06 | 0.483 | -1.05 | -7.994 | 0.0304 | 0.1246 | 0.8088 | -0.3061 | -2.33 |  |
| Acly | ATP citrate lyase | 8.869e-05 | 0.005087 | 0.3557 | -1.491 | -4.891 | 0.0002587 | 0.01154 | 0.3303 | -1.598 | -4.429 | 0.002278 | 0.01904 | 0.4451 | -1.168 | -3.496 | 0.0002439 | 0.003496 | 2.805 | 1.488 | 4.454 |  |
| Foxo3 | forkhead box O3 | 6.882e-06 | 0.0009945 | 1.722 | 0.7838 | 6.026 | 0.5569 | 0.8175 | 1.066 | 0.09197 | 0.5975 | 5.177e-05 | 0.001028 | 1.659 | 0.7303 | 5.125 | 8.346e-09 | 1.27e-06 | 2.541 | 1.345 | 9.441 |  |
| Zfp707 | zinc finger protein 707 | 0.2215 | 0.521 | 0.9061 | -0.1423 | -1.262 | 3.968e-05 | 0.002984 | 0.6158 | -0.6994 | -5.242 | 4.014e-05 | 0.0008477 | 1.566 | 0.6469 | 5.237 | 6.728e-08 | 6.203e-06 | 2.034 | 1.024 | 8.292 |  |
| Gm9903 | predicted gene 9903 | 0.1516 | 0.4277 | 0.8797 | -0.1849 | -1.491 | 0.002704 | 0.05866 | 1.416 | 0.502 | 3.422 | 1.306e-05 | 0.0003593 | 0.5828 | -0.779 | -5.735 | 8.41e-09 | 1.273e-06 | 0.4113 | -1.282 | -9.436 |  |
| Aldh4a1 | aldehyde dehydrogenase 4 famil | 2.401e-09 | 3.623e-06 | 0.4792 | -1.061 | -10.17 | 2.207e-09 | 1.949e-06 | 0.4169 | -1.262 | -10.22 | 0.2591 | 0.5107 | 1.096 | 0.1328 | 1.162 | 0.0005483 | 0.006477 | 1.385 | 0.4695 | 4.107 |  |
| 6430571L13Rik | RIKEN cDNA 6430571L13 gene | 2.75e-09 | 3.724e-06 | 0.2994 | -1.74 | -10.09 | 0.01782 | 0.1816 | 0.6941 | -0.5268 | -2.582 | 0.9222 | 0.9678 | 1.013 | 0.01869 | 0.09896 | 2.043e-14 | 8.758e-11 | 0.0792 | -3.658 | -19.37 |  |
| Map3k5 | mitogen-activated protein kina | 0.00632 | 0.07074 | 1.448 | 0.5345 | 3.051 | 8.962e-06 | 0.001016 | 2.336 | 1.224 | 5.905 | 0.1258 | 0.3281 | 0.8085 | -0.3066 | -1.598 | 1.311e-08 | 1.754e-06 | 0.2947 | -1.763 | -9.184 |  |
| Pfkfb1 | 6-phosphofructo-2-kinase/fruct | 0.2913 | 0.5955 | 1.13 | 0.176 | 1.084 | 2.074e-05 | 0.001876 | 2.088 | 1.062 | 5.529 | 1.214e-09 | 3.089e-07 | 0.271 | -1.884 | -10.59 | 0.000306 | 0.00418 | 0.5843 | -0.7751 | -4.357 |  |
| Slc35g1 | solute carrier family 35, memb | 0.27 | 0.5752 | 1.141 | 0.1902 | 1.135 | 4.465e-05 | 0.003249 | 2.041 | 1.029 | 5.19 | 2.68e-08 | 3.191e-06 | 0.3268 | -1.613 | -8.787 | 8.237e-05 | 0.00151 | 0.5345 | -0.9038 | -4.923 |  |
| Zswim4 | zinc finger SWIM-type containi | 0.2268 | 0.5268 | 0.8821 | -0.1811 | -1.247 | 3.912e-05 | 0.002982 | 0.5353 | -0.9015 | -5.248 | 5.654e-05 | 0.0011 | 1.752 | 0.809 | 5.087 | 4.528e-08 | 4.735e-06 | 2.553 | 1.352 | 8.503 |  |
| Cutal | cutA divalent cation tolerance | 0.002025 | 0.03519 | 0.6815 | -0.5532 | -3.547 | 1.548e-05 | 0.001491 | 0.4849 | -1.044 | -5.659 | 0.0002594 | 0.003595 | 1.689 | 0.7564 | 4.428 | 0.0004461 | 0.005535 | 1.643 | 0.7168 | 4.196 |  |
| Slco1a1 | solute carrier organic anion t | 0.007226 | 0.07612 | 0.6757 | -0.5655 | -2.991 | 2.324e-05 | 0.002026 | 0.4277 | -1.225 | -5.478 | 0.0006324 | 0.00716 | 1.788 | 0.838 | 4.046 | 2.149e-05 | 0.0005326 | 2.206 | 1.142 | 5.513 |  |
| Fst | follistatin | 0.4264 | 0.7099 | 0.8701 | -0.2007 | -0.812 | 7.115e-05 | 0.004645 | 0.3639 | -1.458 | -4.986 | 8.144e-08 | 7.325e-06 | 4.653 | 2.218 | 8.192 | 1.285e-05 | 0.0003605 | 2.938 | 1.555 | 5.743 |  |
| Dhcr7 | 7-dehydrocholesterol reductase | 2.417e-08 | 1.594e-05 | 0.3164 | -1.66 | -8.844 | 1.137e-08 | 5.624e-06 | 0.2402 | -2.058 | -9.265 | 0.002777 | 0.02211 | 1.626 | 0.7013 | 3.411 | 0.02683 | 0.1146 | 1.406 | 0.4914 | 2.39 |  |
| Thyn1 | thymocyte nuclear protein 1 | 0.002826 | 0.04237 | 0.7088 | -0.4966 | -3.403 | 0.1481 | 0.4986 | 1.197 | 0.2598 | 1.504 | 8.7e-09 | 1.373e-06 | 0.3522 | -1.505 | -9.417 | 0.0001153 | 0.00195 | 0.589 | -0.7637 | -4.777 |  |
| Wdr91 | WD repeat domain 91 | 0.351 | 0.6481 | 1.135 | 0.1829 | 0.9549 | 0.001346 | 0.03754 | 0.5572 | -0.8436 | -3.723 | 5.868e-07 | 3.288e-05 | 2.845 | 1.508 | 7.19 | 3.209e-07 | 1.961e-05 | 2.971 | 1.571 | 7.489 |  |
| Slc25a20 | solute carrier family 25 (mito | 0.0003197 | 0.01122 | 1.561 | 0.6426 | 4.338 | 7.326e-06 | 0.0008889 | 2.072 | 1.051 | 5.997 | 0.0002929 | 0.003945 | 0.6113 | -0.7101 | -4.376 | 0.004755 | 0.0326 | 0.6996 | -0.5153 | -3.176 |  |
| Adamts9 | a disintegrin-like and metallo | 0.07774 | 0.2984 | 0.837 | -0.2567 | -1.86 | 4.399e-05 | 0.003233 | 0.5552 | -0.8489 | -5.196 | 2.136e-07 | 1.529e-05 | 2.24 | 1.164 | 7.694 | 0.000624 | 0.007137 | 1.529 | 0.6129 | 4.052 |  |
| Insc | INSC spindle orientation adapt | 0.0032 | 0.04584 | 0.6503 | -0.6208 | -3.349 | 2.025e-05 | 0.001854 | 0.4308 | -1.215 | -5.539 | 0.0002156 | 0.003131 | 1.886 | 0.9153 | 4.507 | 0.0003748 | 0.004852 | 1.824 | 0.8671 | 4.27 |  |
| Pias4 | protein inhibitor of activated | 0.602 | 0.8263 | 0.9613 | -0.05691 | -0.5299 | 5.723e-05 | 0.003923 | 0.6392 | -0.6457 | -5.081 | 6.46e-06 | 0.0002114 | 1.638 | 0.7123 | 6.055 | 1.961e-08 | 2.399e-06 | 2.076 | 1.054 | 8.959 |  |
| 2900026A02Rik | RIKEN cDNA 2900026A02 gene | 0.05262 | 0.2422 | 0.7939 | -0.3329 | -2.06 | 3.075e-05 | 0.002501 | 0.4919 | -1.024 | -5.354 | 0.001234 | 0.01202 | 1.586 | 0.6656 | 3.76 | 1.305e-07 | 1.035e-05 | 2.651 | 1.407 | 7.946 |  |
| Rnf39 | ring finger protein 39 | 1.445e-07 | 6.407e-05 | 2.855 | 1.513 | 7.894 | 0.01589 | 0.1691 | 1.513 | 0.5978 | 2.635 | 0.8936 | 0.9551 | 0.9805 | -0.02845 | -0.1354 | 2.471e-11 | 1.445e-08 | 6.848 | 2.776 | 13.21 |  |
| Lvrn | laeverin | 0.0009834 | 0.02255 | 2.024 | 1.017 | 3.857 | 0.3575 | 0.6973 | 0.8157 | -0.2939 | -0.942 | 3.462e-07 | 2.204e-05 | 4.444 | 2.152 | 7.451 | 5.07e-06 | 0.0001743 | 3.436 | 1.781 | 6.166 |  |
| Tmem55b | NA | 0.02316 | 0.1505 | 1.261 | 0.3344 | 2.46 | 0.01052 | 0.135 | 0.73 | -0.4541 | -2.823 | 0.0003795 | 0.004852 | 1.553 | 0.6352 | 4.265 | 2.439e-10 | 7.558e-08 | 3.318 | 1.73 | 11.62 |  |
| Sec22c | SEC22 homolog C, vesicle traff | 0.02391 | 0.1534 | 0.7744 | -0.3688 | -2.444 | 0.04873 | 0.3064 | 1.297 | 0.3747 | 2.099 | 2.812e-07 | 1.888e-05 | 0.4209 | -1.249 | -7.555 | 8.287e-06 | 0.0002538 | 0.5063 | -0.9818 | -5.941 |  |
| Tnk2 | tyrosine kinase, non-receptor, | 2.221e-06 | 0.0004608 | 0.4399 | -1.185 | -6.55 | 0.007232 | 0.1099 | 0.6416 | -0.6402 | -2.991 | 0.02396 | 0.1074 | 0.7149 | -0.4842 | -2.444 | 1.124e-05 | 0.0003231 | 0.4506 | -1.15 | -5.803 |  |
| Rbl2 | RB transcriptional corepressor | 2.418e-05 | 0.002287 | 1.675 | 0.7441 | 5.46 | 0.01131 | 0.1403 | 1.366 | 0.4499 | 2.79 | 0.102 | 0.2871 | 0.8375 | -0.2559 | -1.714 | 2.435e-07 | 1.623e-05 | 2.202 | 1.139 | 7.627 |  |
| Aco2 | aconitase 2, mitochondrial | 0.5982 | 0.8239 | 1.044 | 0.0628 | 0.5355 | 8.919e-05 | 0.005449 | 1.6 | 0.6783 | 4.888 | 1.094e-07 | 9.11e-06 | 0.4889 | -1.032 | -8.037 | 3.509e-06 | 0.0001275 | 0.5688 | -0.8139 | -6.336 |  |
| Mgll | monoglyceride lipase | 0.5332 | 0.7837 | 1.113 | 0.1551 | 0.6341 | 8.419e-05 | 0.005248 | 2.679 | 1.422 | 4.913 | 1.942e-06 | 8.102e-05 | 0.2929 | -1.772 | -6.613 | 2.722e-07 | 1.737e-05 | 0.2451 | -2.028 | -7.571 |  |
| Serpina12 | serine (or cysteine) peptidase | 0.01353 | 0.1101 | 0.6447 | -0.6332 | -2.709 | 3.487e-05 | 0.002777 | 0.3621 | -1.466 | -5.299 | 0.0001233 | 0.002025 | 2.323 | 1.216 | 4.748 | 6.844e-05 | 0.001332 | 2.431 | 1.281 | 5.003 |  |
| Tbc1d17 | TBC1 domain family, member 17 | 0.0362 | 0.1961 | 1.364 | 0.4477 | 2.246 | 0.02454 | 0.2156 | 0.6719 | -0.5737 | -2.432 | 1.888e-05 | 0.0004739 | 2.324 | 1.217 | 5.57 | 4.904e-08 | 4.994e-06 | 3.599 | 1.848 | 8.46 |  |
| Tbx6 | T-box 6 | 0.002306 | 0.03768 | 1.672 | 0.7416 | 3.491 | 0.193 | 0.5541 | 0.7908 | -0.3387 | -1.347 | 1.508e-08 | 2.108e-06 | 4.344 | 2.119 | 9.106 | 0.0001043 | 0.00181 | 2.176 | 1.122 | 4.82 |  |
| Pank3 | pantothenate kinase 3 | 9.426e-05 | 0.005294 | 0.6202 | -0.6892 | -4.864 | 0.06332 | 0.3468 | 0.7957 | -0.3296 | -1.966 | 1.059e-08 | 1.593e-06 | 0.3675 | -1.444 | -9.305 | 0.02887 | 0.1205 | 1.288 | 0.3655 | 2.355 |  |
| Chrna2 | cholinergic receptor, nicotini | 0.00039 | 0.0126 | 2.565 | 1.359 | 4.253 | 8.901e-06 | 0.001015 | 4.702 | 2.233 | 5.909 | 1.818e-05 | 0.0004624 | 0.2579 | -1.955 | -5.587 | 0.07053 | 0.2227 | 0.6291 | -0.6686 | -1.91 |  |
| Als2cl | ALS2 C-terminal like | 4.16e-06 | 0.000704 | 1.989 | 0.992 | 6.257 | 0.04752 | 0.3034 | 1.316 | 0.3961 | 2.112 | 3.955e-08 | 4.136e-06 | 2.808 | 1.489 | 8.576 | 0.1014 | 0.2801 | 1.23 | 0.2983 | 1.718 |  |
| E230001N04Rik | RIKEN cDNA E230001N04 gene | 7.903e-09 | 8.132e-06 | 5.183 | 2.374 | 9.472 | 1.764e-08 | 8.061e-06 | 6.383 | 2.674 | 9.018 | 0.0003222 | 0.004261 | 0.4383 | -1.19 | -4.335 | 0.305 | 0.5465 | 1.222 | 0.289 | 1.053 |  |
| Stard4 | StAR-related lipid transfer (S | 0.01962 | 0.1367 | 0.647 | -0.6282 | -2.537 | 3.906e-05 | 0.002982 | 0.3444 | -1.538 | -5.249 | 7.607e-05 | 0.00138 | 2.54 | 1.345 | 4.957 | 5.465e-05 | 0.001108 | 2.609 | 1.384 | 5.101 |  |
| Tacc2 | transforming, acidic coiled-co | 1.942e-06 | 0.0004164 | 2.154 | 1.107 | 6.613 | 0.005693 | 0.09423 | 1.53 | 0.6132 | 3.097 | 0.1219 | 0.3213 | 1.228 | 0.2961 | 1.615 | 2.683e-06 | 0.0001041 | 2.273 | 1.185 | 6.461 |  |
| Slc43a2 | solute carrier family 43, memb | 0.001094 | 0.02407 | 1.461 | 0.5472 | 3.812 | 0.3253 | 0.673 | 0.888 | -0.1713 | -1.008 | 1.255e-05 | 0.00035 | 1.872 | 0.9048 | 5.753 | 1.287e-07 | 1.025e-05 | 2.38 | 1.251 | 7.953 |  |
| Osbpl11 | oxysterol binding protein-like | 6.697e-06 | 0.0009844 | 1.829 | 0.8713 | 6.038 | 0.1373 | 0.4797 | 1.201 | 0.2644 | 1.548 | 1.894e-08 | 2.511e-06 | 2.675 | 1.419 | 8.979 | 0.01949 | 0.09078 | 1.321 | 0.4016 | 2.54 |  |
| Pcx | pyruvate carboxylase | 0.007122 | 0.07561 | 0.7618 | -0.3925 | -2.997 | 0.1287 | 0.4691 | 1.186 | 0.2456 | 1.585 | 2.018e-07 | 1.458e-05 | 0.464 | -1.108 | -7.723 | 1.681e-05 | 0.0004413 | 0.5718 | -0.8065 | -5.622 |  |
| Pkp4 | plakophilin 4 | 0.0001623 | 0.007416 | 1.723 | 0.7852 | 4.629 | 0.501 | 0.7874 | 0.9091 | -0.1375 | -0.6853 | 2.095e-09 | 4.768e-07 | 3.746 | 1.905 | 10.25 | 0.0001569 | 0.002468 | 1.819 | 0.8629 | 4.644 |  |
| Ugt1a7c | UDP glucuronosyltransferase 1 | 0.002386 | 0.03842 | 3.162 | 1.661 | 3.476 | 1.983e-05 | 0.001822 | 8.796 | 3.137 | 5.549 | 0.0002383 | 0.003373 | 0.198 | -2.337 | -4.464 | 0.000713 | 0.007888 | 0.2347 | -2.091 | -3.995 |  |
| H2-K2 | histocompatibility 2, K region | 0.6433 | 0.8477 | 1.06 | 0.08474 | 0.4703 | 0.0004314 | 0.0167 | 0.5368 | -0.8977 | -4.21 | 8.241e-07 | 4.3e-05 | 2.614 | 1.387 | 7.024 | 1.409e-07 | 1.076e-05 | 2.95 | 1.561 | 7.906 |  |
| Arhgdia | Rho GDP dissociation inhibitor | 0.3189 | 0.6198 | 0.9104 | -0.1354 | -1.022 | 6.804e-05 | 0.004499 | 0.5804 | -0.7848 | -5.006 | 3.591e-05 | 0.0007805 | 1.702 | 0.7672 | 5.286 | 9.055e-08 | 7.842e-06 | 2.267 | 1.181 | 8.136 |  |
| Rassf4 | Ras association (RalGDS/AF-6) | 0.003371 | 0.04708 | 1.568 | 0.6491 | 3.326 | 0.04935 | 0.3077 | 0.7154 | -0.4832 | -2.093 | 0.002047 | 0.01766 | 1.69 | 0.7573 | 3.542 | 8.318e-11 | 3.292e-08 | 6.232 | 2.64 | 12.35 |  |
| Ifrd1 | interferon-related development | 2.442e-05 | 0.002301 | 1.922 | 0.9425 | 5.456 | 0.198 | 0.5611 | 1.208 | 0.2722 | 1.332 | 2.81e-06 | 0.000109 | 2.327 | 1.219 | 6.44 | 0.0004549 | 0.005612 | 1.732 | 0.7924 | 4.187 |  |
| Cbx7 | chromobox 7 | 2.853e-05 | 0.002548 | 1.7 | 0.7653 | 5.387 | 0.3963 | 0.7243 | 1.106 | 0.1457 | 0.8668 | 6.382e-07 | 3.538e-05 | 2.162 | 1.112 | 7.148 | 0.0001103 | 0.001892 | 1.678 | 0.7464 | 4.796 |  |
| Slc7a2 | solute carrier family 7 (catio | 0.0006687 | 0.01766 | 2.373 | 1.246 | 4.023 | 0.009812 | 0.1296 | 2.066 | 1.047 | 2.854 | 0.0008701 | 0.009091 | 0.3985 | -1.327 | -3.91 | 1.487e-05 | 0.0004023 | 3.803 | 1.927 | 5.677 |  |
| Atl2 | atlastin GTPase 2 | 0.09158 | 0.3263 | 1.248 | 0.3197 | 1.772 | 6.245e-05 | 0.004205 | 2.109 | 1.076 | 5.043 | 7.012e-05 | 0.001289 | 0.5047 | -0.9866 | -4.993 | 2.496e-06 | 9.911e-05 | 0.4108 | -1.284 | -6.495 |  |
| Hand2 | heart and neural crest derivat | 0.1747 | 0.4601 | 1.118 | 0.1606 | 1.407 | 5.735e-05 | 0.003923 | 1.609 | 0.6859 | 5.08 | 0.0001821 | 0.002745 | 0.6725 | -0.5724 | -4.58 | 1.114e-07 | 9.151e-06 | 0.4988 | -1.003 | -8.028 |  |
| Gm6430 | predicted gene 6430 | 5.563e-06 | 0.000862 | 1.683 | 0.7511 | 6.123 | 0.03741 | 0.2714 | 1.251 | 0.3236 | 2.23 | 0.09276 | 0.27 | 1.179 | 0.2372 | 1.766 | 1.809e-07 | 1.277e-05 | 2.064 | 1.045 | 7.778 |  |
| Meiob | meiosis specific with OB domai | 0.0137 | 0.111 | 0.7112 | -0.4917 | -2.703 | 4.203e-05 | 0.003122 | 0.4592 | -1.123 | -5.216 | 2.687e-05 | 0.0006255 | 2.112 | 1.079 | 5.414 | 0.0005752 | 0.006713 | 1.759 | 0.8144 | 4.087 |  |
| Stk38l | serine/threonine kinase 38 lik | 0.0007235 | 0.01835 | 0.6029 | -0.7301 | -3.989 | 1.455e-05 | 0.001455 | 0.4259 | -1.232 | -5.687 | 0.0009069 | 0.009399 | 1.718 | 0.7804 | 3.892 | 0.001744 | 0.0155 | 1.652 | 0.7241 | 3.611 |  |
| Rps6ka3 | ribosomal protein S6 kinase po | 0.000198 | 0.008348 | 0.6117 | -0.7091 | -4.544 | 0.4573 | 0.7624 | 1.102 | 0.14 | 0.758 | 1.084e-09 | 2.873e-07 | 0.2828 | -1.822 | -10.66 | 0.0004012 | 0.005116 | 0.605 | -0.725 | -4.241 |  |
| Gnb5 | guanine nucleotide binding pro | 1.998e-05 | 0.001999 | 0.5242 | -0.9317 | -5.545 | 0.5805 | 0.8293 | 0.9255 | -0.1117 | -0.5618 | 5.6e-05 | 0.001094 | 0.5223 | -0.937 | -5.091 | 6.949e-08 | 6.339e-06 | 0.348 | -1.523 | -8.275 |  |
| A830018L16Rik | RIKEN cDNA A830018L16 gene | 0.0001711 | 0.007657 | 0.6388 | -0.6466 | -4.607 | 0.3086 | 0.6608 | 1.128 | 0.1735 | 1.045 | 5.918e-11 | 3.625e-08 | 0.2615 | -1.935 | -12.59 | 0.002746 | 0.02173 | 0.6949 | -0.5251 | -3.415 |  |
| Tut1 | terminal uridylyl transferase | 1.502e-05 | 0.00163 | 1.638 | 0.7121 | 5.673 | 0.2664 | 0.6238 | 1.125 | 0.1698 | 1.143 | 0.00139 | 0.01318 | 1.424 | 0.5101 | 3.709 | 9.312e-08 | 8.038e-06 | 2.169 | 1.117 | 8.121 |  |
| Slc45a3 | solute carrier family 45, memb | 2.043e-05 | 0.002021 | 2.176 | 1.122 | 5.535 | 2.046e-06 | 0.0003534 | 2.989 | 1.58 | 6.589 | 0.006073 | 0.03946 | 0.6237 | -0.6811 | -3.068 | 0.005371 | 0.03558 | 0.6185 | -0.6931 | -3.122 |  |
| Lgals1 | lectin, galactose binding, sol | 0.001978 | 0.03465 | 0.5983 | -0.7411 | -3.557 | 2.086e-05 | 0.001876 | 0.389 | -1.362 | -5.526 | 0.000293 | 0.003945 | 1.998 | 0.9986 | 4.376 | 0.001155 | 0.01135 | 1.821 | 0.8646 | 3.788 |  |
| Myo5a | myosin VA | 3.789e-05 | 0.003053 | 1.782 | 0.8331 | 5.262 | 0.6003 | 0.8398 | 1.072 | 0.09975 | 0.5325 | 1.765e-06 | 7.493e-05 | 2.227 | 1.155 | 6.658 | 6.868e-06 | 0.0002208 | 2.064 | 1.045 | 6.027 |  |
| Wnt5b | wingless-type MMTV integration | 0.09314 | 0.3293 | 1.219 | 0.2851 | 1.763 | 7.412e-05 | 0.004766 | 1.933 | 0.9506 | 4.969 | 4.173e-05 | 0.000872 | 0.5269 | -0.9245 | -5.22 | 6.503e-06 | 0.0002126 | 0.4757 | -1.072 | -6.052 |  |
| Trafd1 | TRAF type zinc finger domain c | 1.401e-05 | 0.00156 | 1.868 | 0.9014 | 5.704 | 0.9468 | 0.9831 | 0.9913 | -0.01264 | -0.0676 | 1.236e-09 | 3.089e-07 | 3.558 | 1.831 | 10.58 | 3.79e-05 | 0.0008289 | 1.88 | 0.911 | 5.262 |  |
| Ivns1abp | influenza virus NS1A binding p | 9.872e-09 | 9.136e-06 | 2.637 | 1.399 | 9.345 | 2.992e-07 | 7.774e-05 | 2.519 | 1.333 | 7.524 | 0.004019 | 0.02911 | 1.447 | 0.5329 | 3.25 | 0.05506 | 0.1881 | 0.7933 | -0.3341 | -2.038 |  |
| Ranbp9 | RAN binding protein 9 | 2.255e-05 | 0.00218 | 1.823 | 0.8664 | 5.491 | 0.1594 | 0.5135 | 1.208 | 0.2728 | 1.462 | 1.937e-06 | 8.102e-05 | 2.209 | 1.143 | 6.614 | 0.001458 | 0.01349 | 1.556 | 0.6375 | 3.688 |  |
| Slc6a16 | solute carrier family 6, membe | 0.141 | 0.4119 | 1.164 | 0.219 | 1.533 | 7.001e-05 | 0.004587 | 1.795 | 0.844 | 4.993 | 0.0001558 | 0.002428 | 0.6041 | -0.7272 | -4.647 | 4.364e-07 | 2.528e-05 | 0.4513 | -1.148 | -7.335 |  |
| Rrm2 | ribonucleotide reductase M2 | 0.007314 | 0.07664 | 1.44 | 0.5258 | 2.986 | 1.808e-05 | 0.001687 | 2.242 | 1.165 | 5.59 | 2.067e-08 | 2.645e-06 | 0.303 | -1.723 | -8.93 | 0.3267 | 0.5666 | 0.8742 | -0.194 | -1.006 |  |
| Dsg1c | desmoglein 1 gamma | 0.002981 | 0.04394 | 0.626 | -0.6757 | -3.38 | 2.645e-05 | 0.002238 | 0.4111 | -1.282 | -5.421 | 0.001341 | 0.01289 | 1.76 | 0.8157 | 3.724 | 0.0001802 | 0.002755 | 2.006 | 1.004 | 4.584 |  |
| Parp16 | poly (ADP-ribose) polymerase f | 0.0007301 | 0.01848 | 0.6167 | -0.6973 | -3.985 | 0.4637 | 0.7672 | 1.113 | 0.1547 | 0.7471 | 1.463e-06 | 6.512e-05 | 0.408 | -1.293 | -6.748 | 2.056e-06 | 8.641e-05 | 0.4168 | -1.262 | -6.586 |  |
| Jkamp | JNK1/MAPK8-associated membrane | 0.03134 | 0.1804 | 0.8134 | -0.298 | -2.315 | 0.04485 | 0.2947 | 1.253 | 0.326 | 2.14 | 1.141e-05 | 0.0003267 | 0.5675 | -0.8172 | -5.796 | 3.262e-07 | 1.988e-05 | 0.4814 | -1.055 | -7.48 |  |
| Cyp2c29 | cytochrome P450, family 2, sub | 0.01147 | 0.09971 | 0.6754 | -0.5662 | -2.784 | 4.38e-05 | 0.003228 | 0.4201 | -1.251 | -5.198 | 0.000254 | 0.003538 | 1.984 | 0.9886 | 4.437 | 0.0001133 | 0.00193 | 2.094 | 1.066 | 4.784 |  |
| Tln2 | talin 2 | 3.787e-05 | 0.003053 | 0.5233 | -0.9342 | -5.262 | 0.6822 | 0.8805 | 0.9413 | -0.08727 | -0.4155 | 2.173e-07 | 1.541e-05 | 0.3549 | -1.495 | -7.685 | 2.9e-05 | 0.0006744 | 0.4842 | -1.046 | -5.38 |  |
| Smim20 | small integral membrane protei | 0.0002919 | 0.01058 | 0.642 | -0.6394 | -4.377 | 0.6252 | 0.8529 | 1.061 | 0.08575 | 0.4962 | 2.208e-07 | 1.556e-05 | 0.4268 | -1.228 | -7.677 | 8.703e-06 | 0.000264 | 0.5187 | -0.947 | -5.919 |  |
| Aim1 | NA | 1.765e-05 | 0.001846 | 2.444 | 1.289 | 5.6 | 2.95e-06 | 0.0004571 | 3.359 | 1.748 | 6.417 | 0.242 | 0.4909 | 0.81 | -0.3041 | -1.206 | 0.000393 | 0.005033 | 0.4757 | -1.072 | -4.25 |  |
| Enc1 | ectodermal-neural cortex 1 | 1.493e-05 | 0.001628 | 2.299 | 1.201 | 5.675 | 0.0106 | 0.1356 | 1.631 | 0.7059 | 2.819 | 3.859e-07 | 2.409e-05 | 3.282 | 1.714 | 7.397 | 0.3338 | 0.5724 | 0.8529 | -0.2296 | -0.9904 |  |
| 9130401M01Rik | RIKEN cDNA 9130401M01 gene | 0.3764 | 0.6705 | 0.9086 | -0.1382 | -0.9048 | 0.002161 | 0.05116 | 1.554 | 0.6362 | 3.519 | 8.123e-07 | 4.264e-05 | 0.4423 | -1.177 | -7.031 | 1.181e-06 | 5.634e-05 | 0.4517 | -1.147 | -6.85 |  |
| Rab27a | RAB27A, member RAS oncogene fa | 4.342e-07 | 0.0001362 | 2.225 | 1.154 | 7.338 | 3.083e-05 | 0.002501 | 1.995 | 0.9962 | 5.353 | 0.1844 | 0.4154 | 0.8486 | -0.2369 | -1.375 | 0.0005258 | 0.006265 | 1.637 | 0.7108 | 4.125 |  |
| Ergic1 | endoplasmic reticulum-golgi in | 5.663e-07 | 0.0001633 | 0.4396 | -1.186 | -7.207 | 0.0006574 | 0.02261 | 0.5805 | -0.7846 | -4.03 | 0.0001785 | 0.002705 | 0.5637 | -0.8271 | -4.589 | 0.04958 | 0.1752 | 0.7701 | -0.3768 | -2.09 |  |
| Serpina9 | serine (or cysteine) peptidase | 0.00675 | 0.07351 | 0.6945 | -0.5261 | -3.021 | 3.922e-05 | 0.002982 | 0.4727 | -1.081 | -5.247 | 0.001049 | 0.01056 | 1.659 | 0.7304 | 3.83 | 8.73e-05 | 0.001584 | 1.911 | 0.9341 | 4.898 |  |
| Cuedc2 | CUE domain containing 2 | 0.006057 | 0.06916 | 0.7755 | -0.3668 | -3.069 | 0.1584 | 0.5117 | 1.154 | 0.2072 | 1.465 | 7.176e-08 | 6.64e-06 | 0.4726 | -1.081 | -8.258 | 8.765e-05 | 0.001589 | 0.6413 | -0.641 | -4.896 |  |
| Ppp2r5e | protein phosphatase 2, regulat | 0.4763 | 0.7461 | 1.072 | 0.0997 | 0.726 | 0.0001493 | 0.007769 | 1.691 | 0.7581 | 4.665 | 1.316e-06 | 6.094e-05 | 0.4922 | -1.023 | -6.798 | 3.544e-06 | 0.0001282 | 0.5167 | -0.9525 | -6.331 |  |
| Cat | catalase | 0.0002352 | 0.009356 | 1.435 | 0.5215 | 4.47 | 0.6211 | 0.851 | 0.9531 | -0.06931 | -0.5022 | 2.172e-05 | 0.0005296 | 1.629 | 0.7039 | 5.508 | 5.911e-08 | 5.716e-06 | 2.097 | 1.068 | 8.361 |  |
| Gys2 | glycogen synthase 2 | 0.06513 | 0.2713 | 0.6887 | -0.5381 | -1.952 | 0.0192 | 0.189 | 1.779 | 0.8309 | 2.547 | 2.437e-08 | 3.014e-06 | 0.1572 | -2.67 | -8.839 | 0.00012 | 0.002013 | 0.3692 | -1.438 | -4.76 |  |
| Mcm7 | minichromosome maintenance com | 0.006243 | 0.07015 | 1.386 | 0.4711 | 3.056 | 0.1041 | 0.4294 | 0.8063 | -0.3107 | -1.703 | 0.0002633 | 0.003636 | 1.678 | 0.7467 | 4.422 | 7.986e-09 | 1.242e-06 | 3.029 | 1.599 | 9.466 |  |
| Lamb3 | laminin, beta 3 | 0.682 | 0.8697 | 1.047 | 0.06579 | 0.4158 | 0.0001714 | 0.008561 | 1.818 | 0.8623 | 4.606 | 2.876e-07 | 1.922e-05 | 0.404 | -1.308 | -7.543 | 4.287e-06 | 0.0001509 | 0.4723 | -1.082 | -6.243 |  |
| Nkiras1 | NFKB inhibitor interacting Ras | 0.3453 | 0.6431 | 0.9112 | -0.1342 | -0.9665 | 0.002388 | 0.05422 | 1.486 | 0.5712 | 3.476 | 4.31e-06 | 0.0001531 | 0.5178 | -0.9494 | -6.241 | 2.242e-07 | 1.53e-05 | 0.4454 | -1.167 | -7.669 |  |
| Tfpi2 | tissue factor pathway inhibito | 0.01431 | 0.1134 | 0.7138 | -0.4864 | -2.683 | 5.423e-05 | 0.00374 | 0.4681 | -1.095 | -5.105 | 0.0001781 | 0.002704 | 1.881 | 0.9114 | 4.59 | 0.0001722 | 0.002654 | 1.885 | 0.9143 | 4.604 |  |
| Me1 | malic enzyme 1, NADP(+)-depend | 9.38e-06 | 0.001244 | 0.4219 | -1.245 | -5.885 | 0.0003114 | 0.01322 | 0.4702 | -1.089 | -4.35 | 0.0002926 | 0.003945 | 0.4951 | -1.014 | -4.376 | 0.02887 | 0.1205 | 1.46 | 0.5457 | 2.355 |  |
| Gngt1 | guanine nucleotide binding pro | 0.01395 | 0.1118 | 0.7337 | -0.4467 | -2.695 | 5.246e-05 | 0.00365 | 0.4986 | -1.004 | -5.119 | 2.846e-05 | 0.0006531 | 1.97 | 0.9784 | 5.388 | 0.001071 | 0.01069 | 1.618 | 0.6939 | 3.821 |  |
| Btg2 | BTG anti-proliferation factor | 0.002493 | 0.03932 | 2.099 | 1.069 | 3.457 | 0.164 | 0.5202 | 0.6931 | -0.5289 | -1.445 | 0.0004823 | 0.005833 | 2.658 | 1.41 | 4.162 | 3.268e-09 | 6.226e-07 | 10.44 | 3.385 | 9.988 |  |
| Gm15401 | predicted gene 15401 | 0.2366 | 0.5381 | 1.236 | 0.3058 | 1.22 | 9.992e-05 | 0.005963 | 2.703 | 1.435 | 4.839 | 7.111e-05 | 0.001304 | 0.3872 | -1.369 | -4.987 | 4.224e-07 | 2.479e-05 | 0.2469 | -2.018 | -7.352 |  |
| Dpyd | dihydropyrimidine dehydrogenas | 1.222e-05 | 0.001461 | 2.028 | 1.02 | 5.766 | 0.0737 | 0.3715 | 1.315 | 0.3952 | 1.888 | 1.46e-07 | 1.135e-05 | 2.886 | 1.529 | 7.888 | 0.08943 | 0.2587 | 1.271 | 0.346 | 1.785 |  |
| Acss3 | acyl-CoA synthetase short-chai | 2.475e-05 | 0.002324 | 2.174 | 1.12 | 5.45 | 5.439e-06 | 0.0007262 | 2.812 | 1.491 | 6.133 | 0.8094 | 0.9157 | 1.039 | 0.05504 | 0.2445 | 4.427e-05 | 0.0009442 | 0.4446 | -1.169 | -5.194 |  |
| Kctd17 | potassium channel tetramerisat | 0.02823 | 0.1697 | 0.7585 | -0.3989 | -2.366 | 0.06325 | 0.3468 | 1.313 | 0.3924 | 1.967 | 3.9e-07 | 2.423e-05 | 0.3882 | -1.365 | -7.391 | 2.419e-05 | 0.0005848 | 0.497 | -1.009 | -5.46 |  |
| Isoc1 | isochorismatase domain contain | 3.663e-05 | 0.00301 | 0.5215 | -0.9394 | -5.277 | 0.2404 | 0.6003 | 0.8381 | -0.2549 | -1.21 | 3.07e-06 | 0.0001166 | 0.4211 | -1.248 | -6.398 | 0.0005173 | 0.006186 | 0.572 | -0.8058 | -4.132 |  |
| Immt | inner membrane protein, mitoch | 0.04035 | 0.2081 | 0.8629 | -0.2127 | -2.193 | 0.02075 | 0.1974 | 1.221 | 0.2883 | 2.511 | 2.735e-09 | 5.72e-07 | 0.4754 | -1.073 | -10.09 | 0.0006311 | 0.007198 | 0.7422 | -0.4302 | -4.047 |  |
| Nipal1 | NIPA-like domain containing 1 | 0.04207 | 0.2136 | 1.597 | 0.6752 | 2.172 | 7.787e-05 | 0.004958 | 3.53 | 1.82 | 4.947 | 0.0001221 | 0.002019 | 0.3257 | -1.618 | -4.752 | 3.246e-05 | 0.000737 | 0.2842 | -1.815 | -5.33 |  |
| Tspan31 | tetraspanin 31 | 0.3764 | 0.6705 | 0.9103 | -0.1355 | -0.9048 | 0.002424 | 0.05462 | 1.531 | 0.6149 | 3.469 | 1.209e-06 | 5.725e-05 | 0.4594 | -1.122 | -6.839 | 1.108e-06 | 5.359e-05 | 0.4572 | -1.129 | -6.881 |  |
| Syne4 | spectrin repeat containing, nu | 0.1805 | 0.469 | 0.8754 | -0.192 | -1.388 | 0.0001127 | 0.006428 | 0.5809 | -0.7837 | -4.787 | 2.827e-07 | 1.894e-05 | 2.211 | 1.145 | 7.552 | 0.0004865 | 0.005911 | 1.548 | 0.6304 | 4.159 |  |
| Gm26064 | predicted gene, 26064 | 4.546e-06 | 0.0007449 | 2.618 | 1.388 | 6.216 | 0.009874 | 0.1301 | 1.686 | 0.7535 | 2.851 | 4.911e-05 | 0.0009892 | 2.394 | 1.26 | 5.148 | 0.01587 | 0.07837 | 1.564 | 0.6448 | 2.635 |  |
| Steap3 | STEAP family member 3 | 4.081e-06 | 0.0006951 | 0.4792 | -1.061 | -6.266 | 0.007777 | 0.1145 | 0.663 | -0.5928 | -2.958 | 0.0004258 | 0.005291 | 0.5815 | -0.7821 | -4.216 | 0.003132 | 0.02399 | 0.6493 | -0.623 | -3.358 |  |
| Kcnh2 | potassium voltage-gated channe | 8.284e-05 | 0.004882 | 0.6479 | -0.6261 | -4.92 | 0.7926 | 0.9262 | 1.028 | 0.04012 | 0.2665 | 7.232e-09 | 1.193e-06 | 0.3985 | -1.327 | -9.523 | 0.0001055 | 0.001826 | 0.628 | -0.6712 | -4.815 |  |
| Cyp2u1 | cytochrome P450, family 2, sub | 3.141e-06 | 0.0005658 | 0.461 | -1.117 | -6.388 | 8.671e-07 | 0.0001784 | 0.3664 | -1.448 | -6.999 | 0.0002731 | 0.00375 | 1.795 | 0.8441 | 4.406 | 0.4439 | 0.6684 | 1.109 | 0.1496 | 0.781 |  |
| Plxna2 | plexin A2 | 0.6858 | 0.8713 | 1.054 | 0.07586 | 0.4105 | 0.0005184 | 0.01916 | 0.5346 | -0.9034 | -4.132 | 2.007e-08 | 2.621e-06 | 3.509 | 1.811 | 8.947 | 1.91e-05 | 0.0004878 | 2.184 | 1.127 | 5.565 |  |
| Rnf170-ps | ring finger protein 170, pseud | 1.878e-05 | 0.001923 | 0.5637 | -0.8269 | -5.573 | 0.1129 | 0.4448 | 0.8173 | -0.2911 | -1.658 | 0.01932 | 0.092 | 0.7508 | -0.4136 | -2.544 | 4.287e-07 | 2.495e-05 | 0.4371 | -1.194 | -7.344 |  |
| Gnl3 | guanine nucleotide binding pro | 9.42e-05 | 0.005294 | 1.603 | 0.6812 | 4.865 | 0.7241 | 0.8977 | 0.9597 | -0.05931 | -0.3579 | 6.863e-05 | 0.001267 | 1.702 | 0.7673 | 5.002 | 7.794e-09 | 1.23e-06 | 2.74 | 1.454 | 9.48 |  |
| C77080 | expressed sequence C77080 | 0.00701 | 0.07489 | 0.6953 | -0.5243 | -3.005 | 4.691e-05 | 0.003375 | 0.4773 | -1.067 | -5.168 | 0.0001782 | 0.002704 | 1.837 | 0.8773 | 4.589 | 0.0008314 | 0.008864 | 1.683 | 0.7511 | 3.929 |  |
| Tubb4b | tubulin, beta 4B class IVB | 0.2781 | 0.5834 | 0.8383 | -0.2545 | -1.115 | 9.113e-05 | 0.005555 | 0.4011 | -1.318 | -4.879 | 0.0002912 | 0.003936 | 2.136 | 1.095 | 4.378 | 5.728e-08 | 5.624e-06 | 4.272 | 2.095 | 8.378 |  |
| Ptges | prostaglandin E synthase | 7.459e-06 | 0.001048 | 0.434 | -1.204 | -5.989 | 0.8414 | 0.9469 | 1.034 | 0.04824 | 0.2028 | 3.033e-11 | 2.253e-08 | 0.136 | -2.878 | -13.07 | 0.0007161 | 0.007911 | 0.5435 | -0.8796 | -3.993 |  |
| Plcg1 | phospholipase C, gamma 1 | 0.3438 | 0.6421 | 1.082 | 0.1138 | 0.9698 | 0.001521 | 0.04092 | 0.7025 | -0.5095 | -3.67 | 3.879e-09 | 7.446e-07 | 2.412 | 1.27 | 9.886 | 0.0001503 | 0.002387 | 1.515 | 0.5992 | 4.663 |  |
| Cyp2a22 | cytochrome P450, family 2, sub | 0.004775 | 0.0588 | 2.223 | 1.152 | 3.174 | 4.039e-05 | 0.00302 | 4.751 | 2.248 | 5.234 | 0.0003715 | 0.004764 | 0.3079 | -1.7 | -4.274 | 0.0007163 | 0.007911 | 0.3326 | -1.588 | -3.993 |  |
| Lnx1 | ligand of numb-protein X 1 | 0.1005 | 0.3423 | 1.185 | 0.2452 | 1.722 | 0.01365 | 0.1551 | 0.7292 | -0.4557 | -2.705 | 2.892e-08 | 3.291e-06 | 2.574 | 1.364 | 8.746 | 0.0001274 | 0.002112 | 1.668 | 0.7384 | 4.734 |  |
| Amer1 | APC membrane recruitment 1 | 0.000223 | 0.009005 | 0.6146 | -0.7024 | -4.493 | 0.7375 | 0.9031 | 1.045 | 0.06287 | 0.3399 | 3.289e-06 | 0.0001228 | 0.4697 | -1.09 | -6.366 | 7.976e-07 | 4.052e-05 | 0.4336 | -1.206 | -7.04 |  |
| Cidec | cell death-inducing DFFA-like | 0.01683 | 0.1254 | 2.831 | 1.502 | 2.608 | 6.863e-05 | 0.004527 | 10.61 | 3.407 | 5.002 | 0.0001691 | 0.002596 | 0.1332 | -2.908 | -4.612 | 0.0002442 | 0.003498 | 0.1427 | -2.809 | -4.454 |  |
| Tsku | tsukushi, small leucine rich p | 0.007315 | 0.07664 | 1.87 | 0.9028 | 2.986 | 4.737e-05 | 0.003375 | 3.599 | 1.848 | 5.164 | 8.217e-05 | 0.001471 | 0.3229 | -1.631 | -4.924 | 0.001659 | 0.01487 | 0.4342 | -1.203 | -3.633 |  |
| Smyd1 | SET and MYND domain containing | 0.02026 | 0.1391 | 0.7501 | -0.4149 | -2.522 | 0.09175 | 0.4079 | 1.27 | 0.3447 | 1.771 | 7.409e-07 | 3.938e-05 | 0.4133 | -1.275 | -7.076 | 1.882e-05 | 0.0004832 | 0.4986 | -1.004 | -5.572 |  |
| Grn | granulin | 0.0007623 | 0.01895 | 1.839 | 0.8789 | 3.967 | 2.105e-05 | 0.001887 | 2.728 | 1.448 | 5.522 | 0.000961 | 0.009847 | 0.5217 | -0.9387 | -3.867 | 0.004899 | 0.03334 | 0.5874 | -0.7677 | -3.163 |  |
| Aifm2 | apoptosis-inducing factor, mit | 0.0002123 | 0.008764 | 1.891 | 0.9192 | 4.514 | 1.494e-05 | 0.001467 | 2.58 | 1.367 | 5.675 | 0.226 | 0.4703 | 0.8244 | -0.2786 | -1.249 | 0.0001152 | 0.001949 | 0.4778 | -1.066 | -4.778 |  |
| Mmab | methylmalonic aciduria (cobala | 0.0004432 | 0.01362 | 0.488 | -1.035 | -4.199 | 1.687e-05 | 0.001607 | 0.3209 | -1.64 | -5.621 | 0.01109 | 0.06136 | 1.689 | 0.756 | 2.799 | 0.0008586 | 0.009067 | 2.081 | 1.058 | 3.916 |  |
| Pgm3 | phosphoglucomutase 3 | 0.01742 | 0.1278 | 1.423 | 0.5086 | 2.592 | 6.452e-05 | 0.004334 | 2.246 | 1.167 | 5.029 | 0.001709 | 0.0154 | 0.5831 | -0.7781 | -3.62 | 1.864e-05 | 0.00048 | 0.4357 | -1.198 | -5.576 |  |
| Tmem57 | NA | 0.03279 | 0.1855 | 1.265 | 0.3395 | 2.294 | 0.05959 | 0.3375 | 0.7847 | -0.3498 | -1.997 | 2.324e-07 | 1.625e-05 | 2.363 | 1.241 | 7.651 | 5.451e-05 | 0.001106 | 1.774 | 0.8274 | 5.103 |  |
| Fkbp5 | FK506 binding protein 5 | 3.287e-09 | 4.026e-06 | 9.155 | 3.195 | 9.984 | 4.289e-09 | 3.065e-06 | 13.18 | 3.72 | 9.827 | 0.006166 | 0.0399 | 0.4753 | -1.073 | -3.061 | 0.1658 | 0.3806 | 0.7051 | -0.5041 | -1.438 |  |
| Ell | elongation factor RNA polymera | 1.094e-05 | 0.001362 | 1.563 | 0.6442 | 5.815 | 0.8152 | 0.9363 | 1.022 | 0.03104 | 0.2368 | 4.947e-10 | 1.631e-07 | 2.556 | 1.354 | 11.16 | 0.0007009 | 0.007784 | 1.4 | 0.4857 | 4.002 |  |
| Cep85l | centrosomal protein 85-like | 2.916e-06 | 0.0005475 | 2.705 | 1.435 | 6.422 | 0.00153 | 0.04108 | 1.959 | 0.97 | 3.668 | 0.7648 | 0.8926 | 0.9498 | -0.07427 | -0.3033 | 7.177e-06 | 0.0002288 | 2.771 | 1.471 | 6.006 |  |
| Slc36a1 | solute carrier family 36 (prot | 0.1566 | 0.4348 | 0.8334 | -0.263 | -1.472 | 0.0001404 | 0.007475 | 0.5029 | -0.9918 | -4.692 | 2.827e-06 | 0.0001092 | 2.394 | 1.26 | 6.437 | 0.0001268 | 0.002104 | 1.901 | 0.9268 | 4.736 |  |
| Por | P450 (cytochrome) oxidoreducta | 0.0001897 | 0.008099 | 1.855 | 0.8918 | 4.563 | 1.13e-05 | 0.001191 | 2.534 | 1.341 | 5.801 | 0.0009813 | 0.009989 | 0.564 | -0.8261 | -3.858 | 0.02399 | 0.1056 | 0.6959 | -0.523 | -2.443 |  |
| Stat5b | signal transducer and activato | 0.6983 | 0.8766 | 1.032 | 0.04477 | 0.3933 | 0.0006294 | 0.02194 | 0.6853 | -0.5453 | -4.048 | 5.987e-06 | 0.0001992 | 1.693 | 0.7593 | 6.089 | 9.585e-08 | 8.094e-06 | 2.015 | 1.011 | 8.106 |  |
| Lgals4 | lectin, galactose binding, sol | 4.268e-07 | 0.0001355 | 3.168 | 1.664 | 7.347 | 2.823e-07 | 7.409e-05 | 4.067 | 2.024 | 7.553 | 0.4791 | 0.7136 | 0.8833 | -0.1789 | -0.7213 | 0.001656 | 0.01485 | 0.5353 | -0.9015 | -3.634 |  |
| Akap5 | A kinase (PRKA) anchor protein | 0.8723 | 0.9523 | 1.021 | 0.03 | 0.1628 | 0.0002102 | 0.009931 | 1.98 | 0.9855 | 4.518 | 3.228e-06 | 0.000121 | 0.4097 | -1.287 | -6.375 | 1.666e-07 | 1.207e-05 | 0.3347 | -1.579 | -7.821 |  |
| Rnf125 | ring finger protein 125 | 0.205 | 0.5005 | 1.244 | 0.3151 | 1.31 | 0.006948 | 0.1067 | 0.5524 | -0.8562 | -3.008 | 1.025e-07 | 8.643e-06 | 4.367 | 2.127 | 8.071 | 3.761e-05 | 0.0008246 | 2.616 | 1.387 | 5.265 |  |
| Qsox1 | quiescin Q6 sulfhydryl oxidase | 0.89 | 0.9584 | 0.983 | -0.02471 | -0.14 | 0.0002294 | 0.01067 | 0.5229 | -0.9355 | -4.481 | 1.01e-07 | 8.577e-06 | 2.952 | 1.562 | 8.079 | 6.431e-06 | 0.0002113 | 2.251 | 1.171 | 6.057 |  |
| Erc2 | ELKS/RAB6-interacting/CAST fam | 0.06199 | 0.2645 | 0.7832 | -0.3526 | -1.977 | 0.0001114 | 0.006424 | 0.4961 | -1.011 | -4.792 | 4.698e-05 | 0.0009582 | 2.013 | 1.01 | 5.168 | 7.817e-05 | 0.001456 | 1.954 | 0.9662 | 4.945 |  |
| Bdh2 | 3-hydroxybutyrate dehydrogenas | 0.4818 | 0.7496 | 0.8877 | -0.1719 | -0.7168 | 0.0001969 | 0.009483 | 0.409 | -1.29 | -4.546 | 2.255e-07 | 1.585e-05 | 4.038 | 2.014 | 7.666 | 5.444e-05 | 0.001105 | 2.532 | 1.34 | 5.103 |  |
| Arl8a | ADP-ribosylation factor-like 8 | 0.04438 | 0.2198 | 1.237 | 0.3073 | 2.146 | 0.01831 | 0.1844 | 0.7395 | -0.4354 | -2.569 | 0.0006687 | 0.007462 | 1.549 | 0.6311 | 4.023 | 2.377e-09 | 4.739e-07 | 3.025 | 1.597 | 10.18 |  |
| Tmtc2 | transmembrane and tetratricope | 0.3174 | 0.6185 | 1.192 | 0.2531 | 1.025 | 0.0001736 | 0.008606 | 2.537 | 1.343 | 4.6 | 1.471e-05 | 0.0003946 | 0.3448 | -1.536 | -5.682 | 2.951e-06 | 0.0001113 | 0.3005 | -1.735 | -6.417 |  |
| Fam71e1 | family with sequence similarit | 0.003938 | 0.0518 | 1.446 | 0.5318 | 3.259 | 3.942e-05 | 0.002982 | 2.018 | 1.013 | 5.245 | 0.0001329 | 0.002142 | 0.5574 | -0.8431 | -4.716 | 0.003156 | 0.0241 | 0.6598 | -0.5998 | -3.355 |  |
| Camk2b | calcium/calmodulin-dependent p | 0.1923 | 0.4846 | 1.289 | 0.3663 | 1.349 | 0.0001573 | 0.008078 | 2.811 | 1.491 | 4.643 | 2.151e-05 | 0.0005263 | 0.321 | -1.639 | -5.512 | 1.043e-05 | 0.000305 | 0.3003 | -1.736 | -5.837 |  |
| Pcca | propionyl-Coenzyme A carboxyla | 0.00243 | 0.03875 | 0.7297 | -0.4547 | -3.468 | 0.2764 | 0.6324 | 1.128 | 0.1736 | 1.119 | 2.804e-08 | 3.234e-06 | 0.418 | -1.258 | -8.763 | 0.0003026 | 0.004145 | 0.6478 | -0.6264 | -4.362 |  |
| Abcd2 | ATP-binding cassette, sub-fami | 0.325 | 0.6256 | 0.8529 | -0.2296 | -1.009 | 0.003942 | 0.07556 | 1.837 | 0.8773 | 3.258 | 2.249e-06 | 9.098e-05 | 0.3228 | -1.631 | -6.544 | 1.377e-06 | 6.327e-05 | 0.3101 | -1.689 | -6.777 |  |
| Gstp1 | glutathione S-transferase, pi | 0.004704 | 0.05829 | 0.5841 | -0.7758 | -3.181 | 4.515e-05 | 0.003271 | 0.3544 | -1.496 | -5.185 | 0.000611 | 0.006969 | 2.122 | 1.085 | 4.061 | 0.0006574 | 0.007416 | 2.109 | 1.077 | 4.03 |  |
| Erlin2 | ER lipid raft associated 2 | 0.003431 | 0.04765 | 0.7477 | -0.4195 | -3.319 | 0.2382 | 0.5979 | 1.134 | 0.1819 | 1.216 | 3.772e-08 | 4.026e-06 | 0.4379 | -1.191 | -8.602 | 0.0002599 | 0.003679 | 0.6538 | -0.6131 | -4.427 |  |
| Man2a2 | mannosidase 2, alpha 2 | 0.0001266 | 0.006322 | 0.6214 | -0.6864 | -4.737 | 1.026e-05 | 0.001117 | 0.4993 | -1.002 | -5.844 | 0.0001458 | 0.002305 | 1.673 | 0.7422 | 4.676 | 0.2118 | 0.4401 | 1.152 | 0.2048 | 1.29 |  |
| Ces2c | carboxylesterase 2C | 0.07629 | 0.2962 | 0.6861 | -0.5435 | -1.869 | 0.0001175 | 0.006645 | 0.3208 | -1.64 | -4.769 | 0.0001232 | 0.002025 | 2.853 | 1.512 | 4.749 | 1.831e-05 | 0.0004733 | 3.431 | 1.778 | 5.584 |  |
| Tubb5 | tubulin, beta 5 class I | 0.8687 | 0.951 | 1.027 | 0.0381 | 0.1675 | 0.0002904 | 0.01253 | 0.4417 | -1.179 | -4.38 | 9.217e-06 | 0.0002747 | 2.767 | 1.468 | 5.893 | 2.83e-08 | 3.214e-06 | 4.539 | 2.182 | 8.758 |  |
| Bach2 | BTB and CNC homology, basic le | 0.0002698 | 0.01007 | 2.521 | 1.334 | 4.411 | 0.69 | 0.8838 | 0.9045 | -0.1448 | -0.4047 | 1.854e-05 | 0.0004685 | 3.6 | 1.848 | 5.578 | 1.69e-07 | 1.218e-05 | 6.013 | 2.588 | 7.813 |  |
| Nek6 | NIMA (never in mitosis gene a) | 0.002924 | 0.04338 | 0.6107 | -0.7114 | -3.388 | 3.904e-05 | 0.002982 | 0.405 | -1.304 | -5.249 | 0.000528 | 0.006233 | 1.93 | 0.9484 | 4.124 | 0.001767 | 0.01566 | 1.777 | 0.8293 | 3.606 |  |
| Wrb | tryptophan rich basic protein | 0.07406 | 0.2909 | 0.7553 | -0.4048 | -1.885 | 0.03135 | 0.2466 | 1.504 | 0.5884 | 2.315 | 4.139e-06 | 0.0001481 | 0.3603 | -1.473 | -6.259 | 3.021e-06 | 0.0001134 | 0.3518 | -1.507 | -6.406 |  |
| Psmd6 | proteasome (prosome, macropain | 0.4971 | 0.7592 | 1.06 | 0.08434 | 0.6916 | 0.0001124 | 0.006428 | 1.614 | 0.6908 | 4.788 | 7.88e-09 | 1.261e-06 | 0.416 | -1.265 | -9.474 | 0.0004994 | 0.00602 | 0.6811 | -0.554 | -4.147 |  |
| Nrg4 | neuregulin 4 | 0.02245 | 0.1479 | 1.658 | 0.7293 | 2.474 | 6.163e-05 | 0.004172 | 3.389 | 1.761 | 5.049 | 0.02925 | 0.1237 | 0.5912 | -0.7583 | -2.349 | 5.336e-07 | 2.933e-05 | 0.198 | -2.336 | -7.236 |  |
| Rasa3 | RAS p21 protein activator 3 | 5.175e-05 | 0.003698 | 2.223 | 1.153 | 5.125 | 0.3525 | 0.694 | 1.192 | 0.2533 | 0.952 | 0.0001916 | 0.002851 | 2.178 | 1.123 | 4.558 | 3.104e-06 | 0.0001157 | 2.979 | 1.575 | 6.393 |  |
| Eif2ak4 | eukaryotic translation initiat | 0.1025 | 0.346 | 1.179 | 0.2371 | 1.711 | 0.01481 | 0.1635 | 0.7385 | -0.4373 | -2.667 | 6.495e-05 | 0.001222 | 1.697 | 0.7629 | 5.026 | 6.598e-08 | 6.117e-06 | 2.395 | 1.26 | 8.302 |  |
| Slc25a34 | solute carrier family 25, memb | 2.198e-07 | 8.193e-05 | 3.582 | 1.841 | 7.679 | 4.146e-06 | 0.0005958 | 3.423 | 1.775 | 6.259 | 0.05821 | 0.1977 | 0.6937 | -0.5276 | -2.009 | 0.01209 | 0.06462 | 1.653 | 0.7247 | 2.76 |  |
| Fktn | fukutin | 3.348e-05 | 0.002833 | 0.5805 | -0.7847 | -5.316 | 0.2415 | 0.6011 | 0.864 | -0.2108 | -1.207 | 0.003374 | 0.02548 | 0.6888 | -0.5377 | -3.326 | 4.485e-07 | 2.581e-05 | 0.4402 | -1.184 | -7.322 |  |
| Fmo2 | flavin containing monooxygenas | 3.53e-10 | 8.254e-07 | 4.988 | 2.318 | 11.37 | 7.893e-10 | 9.668e-07 | 6.142 | 2.619 | 10.86 | 0.02904 | 0.1231 | 0.6949 | -0.5252 | -2.352 | 0.1082 | 0.2918 | 0.7708 | -0.3755 | -1.682 |  |
| Gm9956 | NA | 4.72e-05 | 0.003479 | 0.5707 | -0.8091 | -5.166 | 0.6976 | 0.887 | 0.9506 | -0.07307 | -0.3943 | 3.635e-08 | 3.896e-06 | 0.3586 | -1.479 | -8.622 | 0.0003953 | 0.005051 | 0.6034 | -0.7288 | -4.248 |  |
| Fibin | fin bud initiation factor homo | 7.161e-06 | 0.001018 | 2.497 | 1.32 | 6.007 | 0.3681 | 0.705 | 1.181 | 0.2395 | 0.9208 | 0.02491 | 0.1106 | 1.499 | 0.5838 | 2.425 | 5.894e-10 | 1.612e-07 | 6.315 | 2.659 | 11.04 |  |
| Gas7 | growth arrest specific 7 | 0.001089 | 0.02404 | 0.6166 | -0.6975 | -3.814 | 3.312e-05 | 0.002654 | 0.4501 | -1.152 | -5.321 | 0.0181 | 0.08786 | 1.43 | 0.5159 | 2.575 | 0.0004181 | 0.005272 | 1.798 | 0.8462 | 4.223 |  |
| Bhlhe40 | basic helix-loop-helix family, | 2.322e-05 | 0.00222 | 0.3973 | -1.332 | -5.478 | 0.07062 | 0.3644 | 0.6833 | -0.5493 | -1.91 | 0.03653 | 0.1444 | 0.6612 | -0.5968 | -2.241 | 2.114e-06 | 8.77e-05 | 0.2972 | -1.75 | -6.573 |  |
| Tbc1d8 | TBC1 domain family, member 8 | 0.02821 | 0.1696 | 1.471 | 0.5564 | 2.366 | 8.705e-05 | 0.00537 | 2.572 | 1.363 | 4.899 | 0.002622 | 0.02122 | 0.5415 | -0.885 | -3.435 | 7.964e-06 | 0.0002469 | 0.3451 | -1.535 | -5.959 |  |
| Tubb4b-ps1 | tubulin, beta 4B class IVB, ps | 0.1511 | 0.427 | 0.8036 | -0.3155 | -1.493 | 9.783e-05 | 0.005853 | 0.4315 | -1.212 | -4.848 | 0.001899 | 0.01661 | 1.775 | 0.8277 | 3.575 | 1.075e-07 | 8.917e-06 | 3.638 | 1.863 | 8.047 |  |
| Vwa3a | von Willebrand factor A domain | 3.038e-06 | 0.0005582 | 2.283 | 1.191 | 6.403 | 0.3133 | 0.6637 | 1.171 | 0.2276 | 1.034 | 6.937e-11 | 3.965e-08 | 5.822 | 2.541 | 12.47 | 0.1029 | 0.283 | 1.273 | 0.3482 | 1.709 |  |
| Kank1 | KN motif and ankyrin repeat do | 0.04048 | 0.2084 | 0.8025 | -0.3175 | -2.191 | 7.367e-05 | 0.00475 | 0.5539 | -0.8524 | -4.971 | 0.01069 | 0.05961 | 1.363 | 0.4469 | 2.815 | 4.757e-07 | 2.705e-05 | 2.231 | 1.158 | 7.293 |  |
| Igf1 | insulin-like growth factor 1 | 3.96e-05 | 0.003106 | 0.5528 | -0.8551 | -5.243 | 0.3866 | 0.7168 | 0.8883 | -0.1708 | -0.8852 | 0.001559 | 0.01439 | 0.6356 | -0.6539 | -3.66 | 1.637e-07 | 1.203e-05 | 0.3792 | -1.399 | -7.829 |  |
| Alas2 | aminolevulinic acid synthase 2 | 0.1059 | 0.3519 | 0.7158 | -0.4823 | -1.694 | 0.0001603 | 0.008176 | 0.3388 | -1.562 | -4.635 | 5.986e-06 | 0.0001992 | 3.731 | 1.899 | 6.089 | 0.0003569 | 0.004682 | 2.529 | 1.339 | 4.291 |  |
| Thbd | thrombomodulin | 0.02558 | 0.1599 | 0.6265 | -0.6745 | -2.412 | 0.08465 | 0.3955 | 1.516 | 0.6003 | 1.815 | 2.955e-07 | 1.959e-05 | 0.2022 | -2.306 | -7.53 | 7.623e-05 | 0.001437 | 0.3491 | -1.518 | -4.956 |  |
| D930015E06Rik | NA | 5.221e-05 | 0.00371 | 2.049 | 1.035 | 5.121 | 0.2137 | 0.5748 | 1.237 | 0.3071 | 1.284 | 0.0003952 | 0.004998 | 1.919 | 0.9402 | 4.248 | 1.706e-05 | 0.0004469 | 2.367 | 1.243 | 5.616 |  |
| Uhrf1bp1l | UHRF1 (ICBP90) binding protein | 8.192e-07 | 0.0002172 | 2.034 | 1.024 | 7.027 | 0.0005498 | 0.01995 | 1.634 | 0.7083 | 4.106 | 0.0002773 | 0.003793 | 1.627 | 0.7026 | 4.399 | 0.1398 | 0.342 | 1.186 | 0.2456 | 1.538 |  |
| Trit1 | tRNA isopentenyltransferase 1 | 1.722e-05 | 0.001808 | 1.86 | 0.8955 | 5.612 | 0.09931 | 0.4197 | 1.254 | 0.3264 | 1.729 | 3.389e-08 | 3.694e-06 | 2.856 | 1.514 | 8.659 | 0.2814 | 0.5212 | 1.144 | 0.1936 | 1.107 |  |
| Spsb1 | splA/ryanodine receptor domain | 2.472e-06 | 0.0005009 | 2.204 | 1.14 | 6.5 | 0.6527 | 0.8664 | 1.068 | 0.09483 | 0.4569 | 0.003808 | 0.02795 | 1.546 | 0.629 | 3.273 | 3.114e-11 | 1.669e-08 | 5.684 | 2.507 | 13.05 |  |
| Mycl | v-myc avian myelocytomatosis v | 1.72e-05 | 0.001808 | 2.297 | 1.2 | 5.612 | 0.002995 | 0.06284 | 1.808 | 0.8545 | 3.378 | 1.788e-05 | 0.0004562 | 2.48 | 1.31 | 5.595 | 0.2554 | 0.4917 | 0.8269 | -0.2742 | -1.171 |  |
| Odc1 | ornithine decarboxylase, struc | 0.001304 | 0.02694 | 1.721 | 0.7835 | 3.737 | 0.06307 | 0.3468 | 1.403 | 0.4883 | 1.968 | 0.003733 | 0.02751 | 0.593 | -0.7538 | -3.282 | 6.535e-07 | 3.431e-05 | 3.115 | 1.639 | 7.137 |  |
| Fam234b | family with sequence similarit | 0.5168 | 0.7729 | 1.076 | 0.1063 | 0.66 | 0.0002578 | 0.01151 | 1.795 | 0.8439 | 4.431 | 3.876e-07 | 2.414e-05 | 0.405 | -1.304 | -7.394 | 4.836e-05 | 0.001012 | 0.5325 | -0.9091 | -5.155 |  |
| Lrp2bp | Lrp2 binding protein | 1.228e-05 | 0.001462 | 0.4183 | -1.257 | -5.763 | 3.243e-06 | 0.0004995 | 0.3198 | -1.645 | -6.373 | 0.0001168 | 0.001948 | 2.204 | 1.14 | 4.772 | 0.9252 | 0.9668 | 1.016 | 0.02271 | 0.09505 |  |
| Sult1d1 | sulfotransferase family 1D, me | 0.04121 | 0.2108 | 1.559 | 0.6407 | 2.182 | 0.0001123 | 0.006428 | 3.168 | 1.664 | 4.789 | 8.629e-05 | 0.001531 | 0.3352 | -1.577 | -4.903 | 0.0001591 | 0.002493 | 0.3556 | -1.492 | -4.638 |  |
| Sntg2 | syntrophin, gamma 2 | 0.007945 | 0.08054 | 0.6986 | -0.5174 | -2.949 | 6.195e-05 | 0.004182 | 0.4837 | -1.048 | -5.047 | 8.727e-05 | 0.001546 | 1.92 | 0.9413 | 4.898 | 0.00298 | 0.02306 | 1.569 | 0.6496 | 3.38 |  |
| Bpnt1 | bisphosphate 3'-nucleotidase 1 | 8.924e-05 | 0.00509 | 0.6263 | -0.6751 | -4.888 | 0.8011 | 0.93 | 0.9715 | -0.04172 | -0.2553 | 4.976e-07 | 2.915e-05 | 0.4665 | -1.1 | -7.271 | 3.569e-05 | 0.0007962 | 0.5743 | -0.8001 | -5.288 |  |
| Atp11a | ATPase, class VI, type 11A | 0.0007085 | 0.01812 | 0.5116 | -0.9669 | -3.998 | 2.521e-05 | 0.002154 | 0.3398 | -1.557 | -5.442 | 0.0004016 | 0.00505 | 2.179 | 1.124 | 4.241 | 0.0239 | 0.1053 | 1.567 | 0.6477 | 2.445 |  |
| Pdk2 | pyruvate dehydrogenase kinase, | 3.821e-05 | 0.003053 | 1.517 | 0.601 | 5.258 | 0.08147 | 0.3899 | 0.842 | -0.2481 | -1.835 | 6.671e-15 | 3.432e-11 | 5.945 | 2.572 | 20.54 | 0.8491 | 0.9309 | 0.9834 | -0.02414 | -0.1928 |  |
| D5Ertd579e | DNA segment, Chr 5, ERATO Doi | 0.02734 | 0.1664 | 0.7394 | -0.4355 | -2.381 | 0.08084 | 0.3889 | 1.318 | 0.398 | 1.839 | 2.166e-07 | 1.541e-05 | 0.3438 | -1.54 | -7.687 | 0.0001113 | 0.0019 | 0.5139 | -0.9603 | -4.792 |  |
| Atp5s | NA | 0.1797 | 0.468 | 1.247 | 0.3189 | 1.39 | 0.0001808 | 0.008841 | 2.369 | 1.244 | 4.583 | 4.827e-05 | 0.000976 | 0.4074 | -1.296 | -5.156 | 9.319e-06 | 0.0002787 | 0.3586 | -1.48 | -5.888 |  |
| Jag2 | jagged 2 | 0.1063 | 0.3529 | 0.8292 | -0.2702 | -1.691 | 0.0737 | 0.3715 | 0.7809 | -0.3568 | -1.888 | 3.063e-07 | 2.01e-05 | 2.488 | 1.315 | 7.512 | 6.857e-06 | 0.0002208 | 0.4813 | -1.055 | -6.027 |  |
| Trpv4 | transient receptor potential c | 5.181e-06 | 0.0008278 | 1.718 | 0.7806 | 6.156 | 0.126 | 0.4656 | 1.181 | 0.2396 | 1.597 | 4.506e-10 | 1.507e-07 | 2.944 | 1.558 | 11.22 | 0.645 | 0.8142 | 1.046 | 0.06498 | 0.4678 |  |
| Bcl9l | B cell CLL/lymphoma 9-like | 0.28 | 0.5851 | 0.8827 | -0.18 | -1.11 | 0.006271 | 0.09957 | 1.501 | 0.5859 | 3.054 | 6.263e-06 | 0.0002068 | 0.4737 | -1.078 | -6.069 | 1.137e-06 | 5.479e-05 | 0.4293 | -1.22 | -6.868 |  |
| Tinagl1 | tubulointerstitial nephritis a | 0.00092 | 0.02143 | 1.519 | 0.6033 | 3.886 | 0.3009 | 0.6548 | 0.8735 | -0.1951 | -1.062 | 7.234e-10 | 2.189e-07 | 3.62 | 1.856 | 10.91 | 0.004813 | 0.0329 | 1.453 | 0.5392 | 3.171 |  |
| Ptp4a1 | protein tyrosine phosphatase 4 | 0.03258 | 0.185 | 1.212 | 0.2769 | 2.297 | 8.201e-05 | 0.005158 | 1.627 | 0.7025 | 4.925 | 1.891e-07 | 1.4e-05 | 0.4916 | -1.024 | -7.756 | 0.07029 | 0.2223 | 0.8394 | -0.2526 | -1.912 |  |
| Gm17709 | predicted gene, 17709 | 0.000833 | 0.02013 | 1.597 | 0.675 | 3.929 | 0.1099 | 0.4405 | 1.266 | 0.3401 | 1.673 | 0.01086 | 0.0604 | 0.6932 | -0.5286 | -2.808 | 8.89e-08 | 7.752e-06 | 2.894 | 1.533 | 8.146 |  |
| Slc15a4 | solute carrier family 15, memb | 1.589e-06 | 0.0003586 | 2.09 | 1.063 | 6.708 | 0.0007156 | 0.02402 | 1.681 | 0.7491 | 3.994 | 0.6981 | 0.8569 | 1.049 | 0.06834 | 0.3935 | 6.328e-05 | 0.001249 | 1.834 | 0.8748 | 5.037 |  |
| Pxdc1 | PX domain containing 1 | 0.1165 | 0.3716 | 1.264 | 0.3383 | 1.641 | 0.02233 | 0.205 | 0.6579 | -0.6041 | -2.477 | 5.28e-06 | 0.0001811 | 2.618 | 1.388 | 6.147 | 3.255e-06 | 0.0001201 | 2.711 | 1.439 | 6.371 |  |
| Cyp4a32 | cytochrome P450, family 4, sub | 0.05869 | 0.257 | 1.391 | 0.4761 | 2.005 | 0.0001381 | 0.007388 | 2.497 | 1.32 | 4.699 | 0.0001501 | 0.002358 | 0.4314 | -1.213 | -4.663 | 5.844e-05 | 0.001167 | 0.4007 | -1.319 | -5.072 |  |
| Cerk | ceramide kinase | 5.932e-06 | 0.0008923 | 2.064 | 1.046 | 6.094 | 0.00483 | 0.08604 | 1.562 | 0.6435 | 3.169 | 0.1201 | 0.3182 | 1.236 | 0.3053 | 1.624 | 0.0001105 | 0.001893 | 1.868 | 0.9015 | 4.795 |  |
| Fbxl21 | F-box and leucine-rich repeat | 0.002352 | 0.03805 | 1.479 | 0.5651 | 3.482 | 4.036e-05 | 0.00302 | 2.007 | 1.005 | 5.234 | 0.0002206 | 0.003181 | 0.5745 | -0.7995 | -4.498 | 0.008354 | 0.04928 | 0.6973 | -0.5202 | -2.926 |  |
| Igfbp5 | insulin-like growth factor bin | 1.895e-05 | 0.001926 | 0.2091 | -2.257 | -5.569 | 0.02988 | 0.24 | 0.4596 | -1.122 | -2.338 | 0.09327 | 0.2709 | 0.5813 | -0.7827 | -1.763 | 8.845e-06 | 0.000268 | 0.1621 | -2.625 | -5.911 |  |
| Gpcpd1 | glycerophosphocholine phosphod | 0.00069 | 0.01793 | 1.927 | 0.9465 | 4.009 | 0.001901 | 0.04684 | 1.998 | 0.9984 | 3.574 | 0.00254 | 0.02073 | 1.856 | 0.892 | 3.449 | 0.0006245 | 0.00714 | 0.4837 | -1.048 | -4.052 |  |
| LOC105243827 | NA | 4.382e-06 | 0.0007225 | 3.914 | 1.969 | 6.233 | 0.004472 | 0.08159 | 2.292 | 1.197 | 3.203 | 0.001452 | 0.01363 | 2.423 | 1.277 | 3.69 | 0.007062 | 0.04368 | 2.054 | 1.038 | 3.001 |  |
| St6galnac2 | ST6 (alpha-N-acetyl-neuraminyl | 0.002434 | 0.03876 | 1.414 | 0.5 | 3.468 | 0.1728 | 0.5307 | 0.846 | -0.2412 | -1.414 | 5.562e-10 | 1.811e-07 | 3.364 | 1.75 | 11.08 | 0.007115 | 0.04394 | 1.389 | 0.4735 | 2.998 |  |
| Tgif1 | TGFB-induced factor homeobox 1 | 0.004675 | 0.05815 | 1.487 | 0.572 | 3.183 | 0.06598 | 0.353 | 0.7508 | -0.4135 | -1.945 | 0.0225 | 0.1026 | 1.401 | 0.4868 | 2.473 | 6.252e-11 | 2.68e-08 | 5.54 | 2.47 | 12.55 |  |
| Lyve1 | lymphatic vessel endothelial h | 0.0009892 | 0.02264 | 2.308 | 1.207 | 3.855 | 3.686e-05 | 0.002873 | 3.872 | 1.953 | 5.274 | 3.336e-05 | 0.0007456 | 0.2826 | -1.823 | -5.318 | 0.2386 | 0.4719 | 0.7492 | -0.4165 | -1.215 |  |
| Cdk2ap2 | CDK2-associated protein 2 | 0.1831 | 0.4727 | 1.17 | 0.2261 | 1.379 | 0.01115 | 0.1392 | 0.6866 | -0.5426 | -2.797 | 3.098e-05 | 0.0007034 | 1.947 | 0.961 | 5.351 | 3.208e-07 | 1.961e-05 | 2.54 | 1.345 | 7.489 |  |
| Gse1 | genetic suppressor element 1, | 0.0004114 | 0.01303 | 0.5975 | -0.7431 | -4.23 | 2.848e-05 | 0.002363 | 0.4602 | -1.12 | -5.388 | 0.07798 | 0.2407 | 1.281 | 0.3575 | 1.858 | 0.0006799 | 0.007614 | 1.708 | 0.7726 | 4.015 |  |
| Ccl9 | chemokine (C-C motif) ligand 9 | 0.3383 | 0.6373 | 1.152 | 0.2043 | 0.981 | 0.005569 | 0.09303 | 0.5882 | -0.7656 | -3.106 | 9.632e-07 | 4.879e-05 | 3.001 | 1.585 | 6.948 | 1.121e-05 | 0.0003229 | 2.504 | 1.324 | 5.804 |  |
| Nxpe3 | neurexophilin and PC-esterase | 1.975e-05 | 0.001984 | 2.156 | 1.108 | 5.55 | 0.1876 | 0.5494 | 1.25 | 0.3223 | 1.364 | 0.2521 | 0.5021 | 1.196 | 0.2579 | 1.179 | 6.328e-09 | 1.037e-06 | 4.286 | 2.1 | 9.6 |  |
| Alpl | alkaline phosphatase, liver/bo | 0.4667 | 0.7387 | 0.8489 | -0.2364 | -0.7421 | 0.001992 | 0.04842 | 2.531 | 1.34 | 3.554 | 5.351e-05 | 0.001056 | 0.2905 | -1.783 | -5.111 | 5.106e-08 | 5.151e-06 | 0.1299 | -2.945 | -8.439 |  |
| Adh6-ps1 | alcohol dehydrogenase 6 (class | 0.03131 | 0.1803 | 0.6134 | -0.7051 | -2.316 | 0.0001233 | 0.006873 | 0.3055 | -1.711 | -4.748 | 0.0004607 | 0.005626 | 2.63 | 1.395 | 4.182 | 9.494e-05 | 0.001694 | 3.077 | 1.621 | 4.861 |  |
| Tspan7 | tetraspanin 7 | 0.0001191 | 0.006117 | 0.6111 | -0.7105 | -4.763 | 1.29e-05 | 0.001328 | 0.4954 | -1.013 | -5.741 | 0.00517 | 0.03488 | 1.427 | 0.513 | 3.139 | 0.02517 | 0.1096 | 1.315 | 0.3955 | 2.42 |  |
| Eepd1 | endonuclease/exonuclease/phosp | 0.0001844 | 0.00801 | 0.4733 | -1.079 | -4.575 | 0.0001406 | 0.007475 | 0.4035 | -1.309 | -4.691 | 0.139 | 0.3496 | 0.7588 | -0.3982 | -1.541 | 0.0004273 | 0.005363 | 2.127 | 1.089 | 4.214 |  |
| Il1r1 | interleukin 1 receptor, type I | 0.008165 | 0.08197 | 1.845 | 0.8836 | 2.937 | 7.329e-05 | 0.004737 | 3.412 | 1.771 | 4.974 | 6.205e-05 | 0.001185 | 0.3157 | -1.663 | -5.046 | 0.006652 | 0.04171 | 0.5007 | -0.998 | -3.028 |  |
| Zfyve26 | zinc finger, FYVE domain conta | 0.8727 | 0.9524 | 1.019 | 0.02784 | 0.1623 | 0.0005924 | 0.02105 | 0.5636 | -0.8273 | -4.074 | 1.218e-07 | 9.701e-06 | 2.829 | 1.5 | 7.982 | 1.527e-05 | 0.0004099 | 2.092 | 1.065 | 5.665 |  |
| Slc2a13 | solute carrier family 2 (facil | 0.02702 | 0.1653 | 0.6392 | -0.6457 | -2.387 | 0.06417 | 0.3482 | 1.545 | 0.6272 | 1.959 | 1.232e-08 | 1.79e-06 | 0.1505 | -2.732 | -9.219 | 0.001538 | 0.01402 | 0.471 | -1.086 | -3.665 |  |
| Adam23 | a disintegrin and metallopepti | 0.02898 | 0.1723 | 1.584 | 0.6636 | 2.353 | 0.000123 | 0.006873 | 3 | 1.585 | 4.749 | 3.115e-05 | 0.000706 | 0.3181 | -1.652 | -5.348 | 0.001806 | 0.01592 | 0.4629 | -1.111 | -3.596 |  |
| Pdzd7 | PDZ domain containing 7 | 1.561e-07 | 6.496e-05 | 0.3826 | -1.386 | -7.854 | 0.1825 | 0.5433 | 0.8188 | -0.2884 | -1.381 | 0.9818 | 0.9938 | 0.9969 | -0.00446 | -0.02307 | 2.368e-13 | 4.351e-10 | 0.1023 | -3.288 | -17.01 |  |
| Gm21839 | predicted gene, 21839 | 0.0001292 | 0.006363 | 2.008 | 1.006 | 4.728 | 0.0001988 | 0.00956 | 2.209 | 1.143 | 4.542 | 0.05233 | 0.1838 | 1.396 | 0.4808 | 2.063 | 0.001039 | 0.01047 | 0.5383 | -0.8934 | -3.834 |  |
| Lce6a | late cornified envelope 6A | 0.01344 | 0.1098 | 0.7067 | -0.5008 | -2.712 | 0.0001038 | 0.006095 | 0.4817 | -1.054 | -4.823 | 0.0006971 | 0.007686 | 1.754 | 0.8103 | 4.005 | 0.0004461 | 0.005535 | 1.801 | 0.8489 | 4.196 |  |
| Egfr | epidermal growth factor recept | 0.4066 | 0.6949 | 0.9049 | -0.1442 | -0.8477 | 0.0002435 | 0.01109 | 0.5372 | -0.8964 | -4.455 | 7.189e-05 | 0.001314 | 1.903 | 0.9281 | 4.982 | 6.589e-07 | 3.452e-05 | 2.512 | 1.329 | 7.133 |  |
| Itgb6 | integrin beta 6 | 2.776e-06 | 0.0005328 | 0.453 | -1.142 | -6.445 | 0.2567 | 0.616 | 0.8439 | -0.2449 | -1.168 | 0.458 | 0.6969 | 1.107 | 0.1469 | 0.7568 | 3.913e-12 | 3.861e-09 | 0.1397 | -2.839 | -14.62 |  |
| Bhlhe41 | basic helix-loop-helix family, | 5.959e-05 | 0.004013 | 0.3338 | -1.583 | -5.064 | 0.2354 | 0.5954 | 0.7308 | -0.4525 | -1.223 | 0.003829 | 0.02805 | 0.4601 | -1.12 | -3.271 | 1.865e-06 | 8.023e-05 | 0.2072 | -2.271 | -6.632 |  |
| Acot8 | acyl-CoA thioesterase 8 | 0.1372 | 0.4067 | 1.19 | 0.2509 | 1.549 | 0.0002104 | 0.009931 | 1.823 | 0.8661 | 4.518 | 0.0001233 | 0.002025 | 0.5576 | -0.8426 | -4.748 | 1.577e-05 | 0.0004213 | 0.499 | -1.003 | -5.651 |  |
| Asb12 | ankyrin repeat and SOCS box-co | 8.386e-08 | 4.314e-05 | 0.3606 | -1.472 | -8.176 | 0.2069 | 0.5691 | 0.8249 | -0.2778 | -1.304 | 6.34e-14 | 2.33e-10 | 0.08264 | -3.597 | -18.24 | 0.9372 | 0.9715 | 1.011 | 0.01573 | 0.07978 |  |
| Rnf5 | ring finger protein 5 | 0.01492 | 0.1162 | 0.7262 | -0.4616 | -2.664 | 0.1511 | 0.5034 | 1.236 | 0.3061 | 1.493 | 3.014e-07 | 1.988e-05 | 0.3718 | -1.428 | -7.52 | 0.0001689 | 0.002611 | 0.545 | -0.8756 | -4.612 |  |
| Pde4b | phosphodiesterase 4B, cAMP spe | 0.07841 | 0.2996 | 0.8098 | -0.3043 | -1.855 | 0.0001354 | 0.007302 | 0.5308 | -0.9138 | -4.708 | 0.004051 | 0.02929 | 1.498 | 0.5834 | 3.246 | 1.004e-06 | 4.93e-05 | 2.37 | 1.245 | 6.928 |  |
| Cml5 | NA | 0.02946 | 0.1738 | 0.5962 | -0.7462 | -2.345 | 0.000129 | 0.007122 | 0.2911 | -1.78 | -4.728 | 0.0004761 | 0.005774 | 2.737 | 1.453 | 4.168 | 0.0001281 | 0.00212 | 3.137 | 1.649 | 4.731 |  |
| Mid1ip1 | Mid1 interacting protein 1 (ga | 0.01527 | 0.1179 | 0.5977 | -0.7425 | -2.653 | 8.042e-05 | 0.005095 | 0.3223 | -1.633 | -4.933 | 0.7153 | 0.867 | 0.9244 | -0.1134 | -0.37 | 1.553e-08 | 1.998e-06 | 6.898 | 2.786 | 9.089 |  |
| Hmbs | hydroxymethylbilane synthase | 4.288e-05 | 0.003283 | 0.62 | -0.6896 | -5.208 | 0.03473 | 0.2616 | 0.7819 | -0.355 | -2.266 | 3.445e-07 | 2.201e-05 | 0.4727 | -1.081 | -7.453 | 0.5997 | 0.7841 | 1.055 | 0.07737 | 0.5334 |  |
| Zfp462 | zinc finger protein 462 | 0.07378 | 0.2905 | 0.8229 | -0.2813 | -1.887 | 0.2158 | 0.5772 | 0.8553 | -0.2255 | -1.278 | 1.951e-06 | 8.119e-05 | 2.113 | 1.08 | 6.611 | 2.389e-07 | 1.605e-05 | 0.4213 | -1.247 | -7.637 |  |
| Acat1 | acetyl-Coenzyme A acetyltransf | 0.5077 | 0.7659 | 0.9425 | -0.0854 | -0.6746 | 0.00186 | 0.04622 | 1.451 | 0.5368 | 3.584 | 1.833e-08 | 2.456e-06 | 0.4211 | -1.248 | -8.997 | 0.0002388 | 0.003443 | 0.6511 | -0.619 | -4.464 |  |
| Ssbp2 | single-stranded DNA binding pr | 0.5963 | 0.8222 | 1.07 | 0.09724 | 0.5384 | 0.0003085 | 0.01312 | 1.906 | 0.9303 | 4.354 | 3.814e-05 | 0.000815 | 0.4862 | -1.04 | -5.259 | 4.039e-07 | 2.394e-05 | 0.3638 | -1.459 | -7.374 |  |
| Apol9a | apolipoprotein L 9a | 0.04067 | 0.2091 | 0.7619 | -0.3924 | -2.189 | 0.0001454 | 0.007635 | 0.5028 | -0.992 | -4.677 | 1.864e-05 | 0.0004695 | 2.136 | 1.095 | 5.576 | 0.001856 | 0.01621 | 1.629 | 0.7039 | 3.585 |  |
| Ubr2 | ubiquitin protein ligase E3 co | 0.0001162 | 0.006028 | 1.528 | 0.6113 | 4.774 | 0.6214 | 0.8511 | 1.054 | 0.076 | 0.5016 | 6.453e-07 | 3.57e-05 | 2.003 | 1.002 | 7.143 | 0.0003458 | 0.004578 | 1.52 | 0.6039 | 4.305 |  |
| Tmem180 | NA | 0.002656 | 0.04077 | 0.6474 | -0.6273 | -3.43 | 6.147e-05 | 0.004172 | 0.4688 | -1.093 | -5.05 | 0.02407 | 0.1078 | 1.404 | 0.4891 | 2.441 | 0.0002045 | 0.003033 | 1.876 | 0.9076 | 4.53 |  |
| Gm5866 | predicted gene 5866 | 0.7065 | 0.8801 | 0.967 | -0.0484 | -0.382 | 0.001157 | 0.03432 | 1.482 | 0.5678 | 3.788 | 7.763e-08 | 7.056e-06 | 0.4537 | -1.14 | -8.217 | 5.437e-05 | 0.001105 | 0.612 | -0.7083 | -5.104 |  |
| Tef | thyrotroph embryonic factor | 0.1329 | 0.3999 | 0.8229 | -0.2812 | -1.567 | 0.02344 | 0.2101 | 1.435 | 0.5212 | 2.454 | 2.825e-06 | 0.0001092 | 0.4159 | -1.266 | -6.437 | 1.119e-05 | 0.0003228 | 0.4533 | -1.142 | -5.805 |  |
| B230340J04Rik | NA | 0.002673 | 0.0409 | 1.71 | 0.7737 | 3.427 | 5.25e-05 | 0.00365 | 2.58 | 1.367 | 5.119 | 0.0001316 | 0.002128 | 0.4453 | -1.167 | -4.72 | 0.02208 | 0.09933 | 0.6535 | -0.6138 | -2.482 |  |
| Ntm | neurotrimin | 0.7662 | 0.9079 | 0.9642 | -0.05257 | -0.3014 | 0.0006972 | 0.0236 | 1.773 | 0.8265 | 4.005 | 1.796e-08 | 2.444e-06 | 0.3033 | -1.721 | -9.008 | 0.0001109 | 0.001896 | 0.53 | -0.916 | -4.794 |  |
| Pdgfrb | platelet derived growth factor | 0.2007 | 0.4955 | 0.8576 | -0.2217 | -1.323 | 0.0002857 | 0.01239 | 0.5473 | -0.8696 | -4.387 | 4.339e-06 | 0.0001539 | 2.211 | 1.145 | 6.238 | 0.0003091 | 0.004215 | 1.74 | 0.799 | 4.353 |  |
| Unc5a | unc-5 netrin receptor A | 2.339e-05 | 0.002228 | 0.4834 | -1.049 | -5.475 | 0.03956 | 0.2783 | 0.7075 | -0.4991 | -2.202 | 2.713e-07 | 1.848e-05 | 0.3324 | -1.589 | -7.573 | 0.7783 | 0.8951 | 0.9593 | -0.05989 | -0.2854 |  |
| Ccdc77 | coiled-coil domain containing | 0.0007108 | 0.01816 | 1.655 | 0.7265 | 3.996 | 0.2461 | 0.6057 | 0.8368 | -0.257 | -1.195 | 0.0253 | 0.1118 | 1.396 | 0.4815 | 2.418 | 7.735e-11 | 3.158e-08 | 5.537 | 2.469 | 12.4 |  |
| Elovl6 | ELOVL family member 6, elongat | 7.018e-05 | 0.004456 | 0.3604 | -1.472 | -4.992 | 0.01344 | 0.1541 | 0.5189 | -0.9463 | -2.712 | 6.307e-06 | 0.0002075 | 0.2571 | -1.96 | -6.066 | 0.2517 | 0.4866 | 1.303 | 0.3814 | 1.18 |  |
| Atp8b1 | ATPase, class I, type 8B, memb | 0.704 | 0.879 | 0.9526 | -0.06998 | -0.3854 | 0.0002779 | 0.01217 | 0.5194 | -0.9451 | -4.398 | 2.191e-08 | 2.763e-06 | 3.411 | 1.77 | 8.898 | 0.0003504 | 0.004613 | 1.809 | 0.8552 | 4.299 |  |
| Rsad1 | radical S-adenosyl methionine | 0.1056 | 0.3512 | 1.217 | 0.2832 | 1.695 | 0.0001264 | 0.006992 | 1.914 | 0.9363 | 4.737 | 0.01049 | 0.05879 | 0.6989 | -0.5167 | -2.824 | 1.471e-07 | 1.105e-05 | 0.3679 | -1.443 | -7.884 |  |
| 5730508B09Rik | NA | 0.0001648 | 0.007477 | 0.5801 | -0.7857 | -4.623 | 0.5155 | 0.7953 | 1.097 | 0.1331 | 0.662 | 1.607e-10 | 7.514e-08 | 0.2154 | -2.215 | -11.9 | 0.008697 | 0.05068 | 0.6871 | -0.5414 | -2.908 |  |
| Doc2b | double C2, beta | 6.521e-06 | 0.0009641 | 2.368 | 1.244 | 6.05 | 0.9385 | 0.9799 | 1.013 | 0.019 | 0.07812 | 0.001557 | 0.01438 | 1.771 | 0.8243 | 3.66 | 5.366e-11 | 2.38e-08 | 7.21 | 2.85 | 12.66 |  |
| Gemin2 | gem nuclear organelle associat | 0.2213 | 0.521 | 1.133 | 0.1802 | 1.262 | 0.0002155 | 0.01015 | 1.695 | 0.7612 | 4.508 | 0.0006455 | 0.007247 | 0.6456 | -0.6312 | -4.038 | 6.882e-07 | 3.584e-05 | 0.4627 | -1.112 | -7.111 |  |
| Slc25a32 | solute carrier family 25, memb | 0.0001848 | 0.00801 | 1.796 | 0.8448 | 4.574 | 2.733e-05 | 0.002281 | 2.268 | 1.182 | 5.406 | 0.7669 | 0.8936 | 0.9587 | -0.06079 | -0.3004 | 0.0001404 | 0.002276 | 0.5178 | -0.9494 | -4.692 |  |
| Ap3m1-ps | adaptor-related protein comple | 0.7194 | 0.8863 | 0.9663 | -0.04947 | -0.3643 | 0.001462 | 0.0396 | 1.508 | 0.5923 | 3.687 | 1.219e-06 | 5.752e-05 | 0.4943 | -1.017 | -6.835 | 5.715e-06 | 0.0001922 | 0.5326 | -0.9088 | -6.111 |  |
| Them6 | thioesterase superfamily membe | 0.0004813 | 0.01434 | 0.6467 | -0.6288 | -4.163 | 0.7424 | 0.9054 | 1.042 | 0.05957 | 0.3333 | 9.606e-06 | 0.0002834 | 0.5098 | -0.9719 | -5.874 | 1.965e-06 | 8.355e-05 | 0.4687 | -1.093 | -6.608 |  |
| Arv1 | ARV1 homolog, fatty acid homeo | 0.001153 | 0.0249 | 1.554 | 0.6364 | 3.789 | 4.224e-05 | 0.003122 | 2.051 | 1.036 | 5.214 | 0.00197 | 0.01711 | 0.6352 | -0.6547 | -3.559 | 0.007746 | 0.04664 | 0.6856 | -0.5446 | -2.96 |  |
| Pck2 | phosphoenolpyruvate carboxykin | 6.504e-05 | 0.00429 | 1.727 | 0.7886 | 5.025 | 0.191 | 0.5522 | 1.19 | 0.2513 | 1.354 | 6.509e-07 | 3.593e-05 | 2.341 | 1.227 | 7.139 | 0.03677 | 0.1425 | 1.306 | 0.3847 | 2.238 |  |
| Fbxo21 | F-box protein 21 | 7.224e-05 | 0.004532 | 1.883 | 0.9129 | 4.98 | 0.7208 | 0.8964 | 1.056 | 0.07864 | 0.3625 | 3.161e-08 | 3.551e-06 | 3.356 | 1.747 | 8.697 | 0.001218 | 0.01181 | 1.689 | 0.7562 | 3.766 |  |
| Gm34985 | NA | 0.004077 | 0.05289 | 2.123 | 1.086 | 3.243 | 7.495e-05 | 0.0048 | 3.908 | 1.966 | 4.964 | 0.01969 | 0.09328 | 0.5249 | -0.9299 | -2.536 | 0.0001489 | 0.002373 | 0.3053 | -1.712 | -4.667 |  |
| Abhd17c | abhydrolase domain containing | 0.0002405 | 0.009444 | 0.6121 | -0.7081 | -4.461 | 2.203e-05 | 0.001928 | 0.4886 | -1.033 | -5.502 | 0.004828 | 0.0332 | 1.465 | 0.5511 | 3.169 | 0.02401 | 0.1057 | 1.342 | 0.4247 | 2.442 |  |
| Snord14e | small nucleolar RNA, C/D box 1 | 0.001795 | 0.03265 | 1.776 | 0.8282 | 3.599 | 5.347e-05 | 0.003707 | 2.624 | 1.392 | 5.111 | 0.002207 | 0.01859 | 0.5416 | -0.8848 | -3.51 | 0.004551 | 0.03158 | 0.5722 | -0.8055 | -3.195 |  |
| Eif1ax | eukaryotic translation initiat | 0.04515 | 0.2219 | 0.7976 | -0.3262 | -2.137 | 0.07517 | 0.3754 | 1.265 | 0.339 | 1.877 | 1.079e-06 | 5.237e-05 | 0.4498 | -1.153 | -6.893 | 6.262e-05 | 0.001238 | 0.5575 | -0.843 | -5.042 |  |
| Gin1 | gypsy retrotransposon integras | 0.4695 | 0.7414 | 0.8957 | -0.1588 | -0.7373 | 0.003349 | 0.06772 | 1.801 | 0.8486 | 3.329 | 1.272e-05 | 0.0003532 | 0.3906 | -1.356 | -5.747 | 7.654e-07 | 3.922e-05 | 0.3151 | -1.666 | -7.06 |  |
| Dbp | D site albumin promoter bindin | 9.832e-05 | 0.00545 | 0.2269 | -2.14 | -4.846 | 0.5558 | 0.8172 | 0.8049 | -0.3131 | -0.5992 | 0.0002518 | 0.003513 | 0.2256 | -2.148 | -4.441 | 1.09e-06 | 5.289e-05 | 0.09926 | -3.333 | -6.889 |  |
| Sephs2 | selenophosphate synthetase 2 | 0.01138 | 0.09935 | 1.422 | 0.5074 | 2.787 | 0.06293 | 0.3466 | 0.7452 | -0.4242 | -1.969 | 2.444e-10 | 9.673e-08 | 4.982 | 2.317 | 11.62 | 0.02636 | 0.1133 | 1.393 | 0.4783 | 2.398 |  |
| Slc38a6 | solute carrier family 38, memb | 0.1804 | 0.4689 | 1.154 | 0.2062 | 1.388 | 0.0001759 | 0.008673 | 1.751 | 0.8078 | 4.595 | 0.003033 | 0.0236 | 0.6835 | -0.5489 | -3.372 | 2.045e-07 | 1.422e-05 | 0.4187 | -1.256 | -7.716 |  |
| Pld5 | phospholipase D family, member | 5.09e-06 | 0.0008182 | 0.4972 | -1.008 | -6.164 | 0.6214 | 0.8511 | 0.9349 | -0.09707 | -0.5017 | 0.02209 | 0.1012 | 0.7348 | -0.4446 | -2.482 | 4.548e-11 | 2.089e-08 | 0.2047 | -2.288 | -12.77 |  |
| Zfyve16 | zinc finger, FYVE domain conta | 0.3525 | 0.6491 | 1.09 | 0.1239 | 0.952 | 0.0002412 | 0.01103 | 1.61 | 0.6868 | 4.459 | 0.0004707 | 0.005724 | 0.662 | -0.595 | -4.173 | 2.24e-07 | 1.53e-05 | 0.4686 | -1.094 | -7.67 |  |
| Abhd2 | abhydrolase domain containing | 9.641e-05 | 0.005393 | 2.672 | 1.418 | 4.854 | 1.75e-05 | 0.001655 | 3.829 | 1.937 | 5.604 | 0.1455 | 0.3597 | 0.7147 | -0.4847 | -1.515 | 0.003186 | 0.02425 | 0.4756 | -1.072 | -3.351 |  |
| Gm13363 | predicted gene 13363 | 0.02359 | 0.1522 | 1.246 | 0.3172 | 2.451 | 0.0001126 | 0.006428 | 1.662 | 0.7331 | 4.787 | 3.29e-07 | 2.129e-05 | 0.4797 | -1.06 | -7.476 | 0.2 | 0.4254 | 0.8779 | -0.1879 | -1.325 |  |
| Serpinh1 | serine (or cysteine) peptidase | 6.746e-06 | 0.000986 | 0.4079 | -1.294 | -6.035 | 0.004462 | 0.08159 | 0.5693 | -0.8127 | -3.204 | 0.0001401 | 0.002232 | 0.4658 | -1.102 | -4.693 | 0.162 | 0.3752 | 0.7895 | -0.341 | -1.452 |  |
| Fmo3 | flavin containing monooxygenas | 0.000337 | 0.01153 | 2.55 | 1.35 | 4.316 | 0.0002005 | 0.009604 | 3.204 | 1.68 | 4.539 | 0.1832 | 0.4139 | 1.387 | 0.4725 | 1.379 | 0.0003787 | 0.00489 | 0.363 | -1.462 | -4.266 |  |
| Slc25a51 | solute carrier family 25, memb | 0.1452 | 0.4185 | 1.242 | 0.3122 | 1.516 | 0.0001326 | 0.007225 | 2.218 | 1.149 | 4.717 | 0.01997 | 0.09424 | 0.6734 | -0.5705 | -2.529 | 3.762e-08 | 4.049e-06 | 0.2605 | -1.941 | -8.603 |  |
| Npas2 | neuronal PAS domain protein 2 | 0.43 | 0.7123 | 1.191 | 0.252 | 0.8055 | 0.003879 | 0.07458 | 0.4326 | -1.209 | -3.265 | 9.726e-08 | 8.396e-06 | 6.849 | 2.776 | 8.099 | 0.0001419 | 0.002294 | 3.046 | 1.607 | 4.687 |  |
| Fam83a | family with sequence similarit | 5.266e-05 | 0.003711 | 0.5662 | -0.8205 | -5.118 | 0.3967 | 0.7246 | 0.8923 | -0.1643 | -0.8662 | 2.906e-08 | 3.293e-06 | 0.3449 | -1.536 | -8.743 | 0.02288 | 0.1018 | 0.7407 | -0.433 | -2.465 |  |
| Hsd17b6 | hydroxysteroid (17-beta) dehyd | 0.03056 | 0.1774 | 0.7211 | -0.4717 | -2.328 | 0.0001577 | 0.008081 | 0.4623 | -1.113 | -4.642 | 0.0001019 | 0.001749 | 2.103 | 1.072 | 4.83 | 0.001032 | 0.01042 | 1.805 | 0.8518 | 3.837 |  |
| Fam126b | family with sequence similarit | 0.3086 | 0.6114 | 1.136 | 0.1841 | 1.045 | 0.0003232 | 0.01354 | 1.87 | 0.9033 | 4.334 | 5.831e-07 | 3.275e-05 | 0.3821 | -1.388 | -7.193 | 0.0007234 | 0.007972 | 0.5865 | -0.7697 | -3.989 |  |
| Gm10309 | predicted gene 10309 | 3.072e-06 | 0.0005605 | 2.449 | 1.292 | 6.398 | 0.6562 | 0.8682 | 1.078 | 0.108 | 0.4519 | 0.02639 | 0.115 | 1.445 | 0.5306 | 2.398 | 1.359e-11 | 9.987e-09 | 8.127 | 3.023 | 13.66 |  |
| Hn1l | NA | 5.144e-05 | 0.003685 | 0.5159 | -0.9547 | -5.128 | 1.465e-05 | 0.001455 | 0.4198 | -1.252 | -5.684 | 0.5508 | 0.7675 | 1.09 | 0.1238 | 0.6068 | 0.001214 | 0.01177 | 1.703 | 0.7683 | 3.767 |  |
| Cryz | crystallin, zeta | 7.8e-05 | 0.004696 | 0.6077 | -0.7186 | -4.946 | 0.2347 | 0.5951 | 0.8642 | -0.2106 | -1.225 | 1.356e-06 | 6.175e-05 | 0.4732 | -1.08 | -6.784 | 0.01127 | 0.06162 | 0.735 | -0.4442 | -2.792 |  |
| Tec | tec protein tyrosine kinase | 1.388e-05 | 0.001552 | 2.026 | 1.019 | 5.708 | 0.007475 | 0.1117 | 1.546 | 0.6283 | 2.976 | 0.3882 | 0.6388 | 1.127 | 0.1724 | 0.882 | 5.394e-05 | 0.001098 | 1.998 | 0.9982 | 5.107 |  |
| Tango2 | transport and golgi organizati | 7.519e-06 | 0.001051 | 2.225 | 1.154 | 5.985 | 2.462e-05 | 0.002125 | 2.367 | 1.243 | 5.452 | 0.007545 | 0.04612 | 0.6473 | -0.6274 | -2.972 | 0.1055 | 0.2876 | 1.282 | 0.358 | 1.696 |  |
| Zpr1 | ZPR1 zinc finger | 0.03201 | 0.1828 | 1.406 | 0.492 | 2.305 | 0.1039 | 0.429 | 0.7421 | -0.4303 | -1.704 | 1.079e-06 | 5.237e-05 | 3.056 | 1.612 | 6.894 | 7.899e-05 | 0.001466 | 2.227 | 1.155 | 4.941 |  |
| Prg4 | proteoglycan 4 (megakaryocyte | 0.01919 | 0.1352 | 2.289 | 1.195 | 2.547 | 0.1463 | 0.4957 | 0.5591 | -0.8387 | -1.512 | 3.366e-05 | 0.0007484 | 6.633 | 2.73 | 5.314 | 2.17e-06 | 8.932e-05 | 10.34 | 3.37 | 6.561 |  |
| Hfe | homeostatic iron regulator | 3.396e-05 | 0.00285 | 0.5156 | -0.9557 | -5.31 | 0.6308 | 0.8559 | 0.9305 | -0.1039 | -0.488 | 3.107e-09 | 6.293e-07 | 0.2544 | -1.975 | -10.02 | 0.008044 | 0.04785 | 0.6688 | -0.5803 | -2.943 |  |
| 9030617O03Rik | NA | 0.0651 | 0.2713 | 0.8192 | -0.2878 | -1.952 | 0.04794 | 0.3045 | 1.29 | 0.3675 | 2.107 | 9.343e-08 | 8.119e-06 | 0.403 | -1.311 | -8.12 | 0.0006212 | 0.007108 | 0.6352 | -0.6547 | -4.054 |  |
| Serpine2 | serine (or cysteine) peptidase | 0.08263 | 0.3073 | 0.6654 | -0.5876 | -1.827 | 0.04389 | 0.2916 | 1.764 | 0.8185 | 2.151 | 2.203e-05 | 0.0005351 | 0.261 | -1.938 | -5.502 | 2.617e-06 | 0.0001025 | 0.2059 | -2.28 | -6.473 |  |
| Nol3 | nucleolar protein 3 (apoptosis | 0.001283 | 0.02674 | 0.5851 | -0.7732 | -3.744 | 0.0101 | 0.1317 | 0.618 | -0.6944 | -2.841 | 0.0002079 | 0.003044 | 0.492 | -1.023 | -4.523 | 0.002376 | 0.01952 | 1.725 | 0.7869 | 3.478 |  |
| Ank1 | ankyrin 1, erythroid | 0.0006145 | 0.01671 | 0.6017 | -0.7328 | -4.059 | 0.03475 | 0.2616 | 0.715 | -0.484 | -2.265 | 0.03448 | 0.1388 | 1.365 | 0.4488 | 2.269 | 6.618e-06 | 0.0002152 | 0.4367 | -1.195 | -6.043 |  |
| Mpp3 | membrane protein, palmitoylate | 0.0005216 | 0.01508 | 0.5898 | -0.7618 | -4.129 | 0.2225 | 0.5838 | 0.8265 | -0.2749 | -1.259 | 0.04354 | 0.1626 | 1.352 | 0.4356 | 2.155 | 9.243e-09 | 1.359e-06 | 0.2686 | -1.896 | -9.382 |  |
| Gdf1 | growth differentiation factor | 1.419e-05 | 0.00156 | 0.4712 | -1.086 | -5.698 | 0.1306 | 0.4718 | 0.7816 | -0.3554 | -1.577 | 0.9562 | 0.9825 | 0.992 | -0.01162 | -0.05566 | 1.545e-09 | 3.34e-07 | 0.2208 | -2.179 | -10.44 |  |
| Dnajb4 | DnaJ heat shock protein family | 0.1417 | 0.413 | 1.21 | 0.2752 | 1.53 | 0.0002767 | 0.01217 | 1.914 | 0.9363 | 4.4 | 7.024e-05 | 0.001291 | 0.5058 | -0.9834 | -4.992 | 5.788e-05 | 0.001159 | 0.5 | -1 | -5.076 |  |
| Mcf2l | mcf.2 transforming sequence-li | 0.00059 | 0.0163 | 1.575 | 0.6555 | 4.076 | 0.7128 | 0.8932 | 0.9519 | -0.07104 | -0.3734 | 2.925e-07 | 1.944e-05 | 2.509 | 1.327 | 7.535 | 0.0001002 | 0.001762 | 1.805 | 0.8522 | 4.838 |  |
| Ano8 | anoctamin 8 | 4.75e-05 | 0.003481 | 0.6412 | -0.6411 | -5.163 | 0.9299 | 0.9778 | 0.991 | -0.01309 | -0.08911 | 2.41e-09 | 5.294e-07 | 0.3833 | -1.384 | -10.17 | 0.001487 | 0.01368 | 0.7068 | -0.5006 | -3.68 |  |
| Dpy19l1 | dpy-19-like 1 (C. elegans) | 0.07296 | 0.2884 | 1.288 | 0.3648 | 1.893 | 0.000219 | 0.0103 | 2.037 | 1.026 | 4.501 | 7.157e-06 | 0.0002284 | 0.4151 | -1.268 | -6.008 | 0.002897 | 0.02259 | 0.6087 | -0.7161 | -3.392 |  |
| Galnt2 | polypeptide N-acetylgalactosam | 0.09296 | 0.3289 | 0.8217 | -0.2834 | -1.764 | 0.04103 | 0.2833 | 1.333 | 0.4151 | 2.184 | 1.488e-05 | 0.000397 | 0.5004 | -0.9988 | -5.677 | 4.38e-06 | 0.0001529 | 0.4676 | -1.097 | -6.233 |  |
| Gm13233 | predicted gene 13233 | 0.0001138 | 0.005927 | 0.6005 | -0.7357 | -4.783 | 0.3855 | 0.7161 | 0.8941 | -0.1615 | -0.8872 | 1.703e-06 | 7.264e-05 | 0.4586 | -1.125 | -6.675 | 0.002024 | 0.01733 | 0.6608 | -0.5977 | -3.547 |  |
| Golt1a | golgi transport 1A | 0.339 | 0.6382 | 1.167 | 0.2227 | 0.9795 | 0.0003822 | 0.01522 | 2.214 | 1.147 | 4.262 | 3.948e-05 | 0.0008378 | 0.4044 | -1.306 | -5.244 | 9.403e-06 | 0.0002805 | 0.3621 | -1.466 | -5.884 |  |
| Atp6v1h | ATPase, H+ transporting, lysos | 0.001096 | 0.02407 | 1.632 | 0.7065 | 3.811 | 0.01926 | 0.1893 | 1.473 | 0.5584 | 2.546 | 0.006322 | 0.04059 | 0.6509 | -0.6194 | -3.05 | 3.997e-05 | 0.0008665 | 2.09 | 1.064 | 5.239 |  |
| Smim19 | small integral membrane protei | 0.04035 | 0.2081 | 1.204 | 0.2679 | 2.193 | 0.0001303 | 0.007133 | 1.606 | 0.6831 | 4.724 | 0.1467 | 0.3614 | 0.8693 | -0.2021 | -1.51 | 1.756e-07 | 1.251e-05 | 0.4852 | -1.043 | -7.794 |  |
| Fam129b | family with sequence similarit | 0.148 | 0.4229 | 1.34 | 0.4222 | 1.505 | 0.004241 | 0.07896 | 0.476 | -1.071 | -3.226 | 2.02e-10 | 8.805e-08 | 12.2 | 3.609 | 11.74 | 0.01032 | 0.05735 | 1.828 | 0.8702 | 2.832 |  |
| Trim63 | tripartite motif-containing 63 | 3.4e-07 | 0.0001198 | 1.876 | 0.9076 | 7.46 | 0.6571 | 0.8688 | 1.046 | 0.06487 | 0.4506 | 0.07209 | 0.228 | 0.8391 | -0.2531 | -1.899 | 4.919e-15 | 2.924e-11 | 6.874 | 2.781 | 20.87 |  |
| Slc39a13 | solute carrier family 39 (meta | 0.01399 | 0.112 | 0.7403 | -0.4338 | -2.693 | 0.199 | 0.562 | 1.192 | 0.2531 | 1.328 | 1.665e-06 | 7.172e-05 | 0.4415 | -1.18 | -6.686 | 6.991e-05 | 0.001352 | 0.543 | -0.8811 | -4.994 |  |
| Lsr | lipolysis stimulated lipoprote | 0.122 | 0.3815 | 0.8015 | -0.3191 | -1.615 | 0.0003016 | 0.01291 | 0.4931 | -1.02 | -4.363 | 2.137e-05 | 0.0005236 | 2.288 | 1.194 | 5.515 | 0.0004411 | 0.005489 | 1.878 | 0.9093 | 4.201 |  |
| Cmbl | carboxymethylenebutenolidase-l | 0.9238 | 0.9728 | 1.01 | 0.01387 | 0.0968 | 0.0004366 | 0.01682 | 1.639 | 0.7127 | 4.205 | 3.557e-05 | 0.000778 | 0.5625 | -0.8301 | -5.29 | 1.026e-07 | 8.566e-06 | 0.4157 | -1.266 | -8.071 |  |
| Asxl1 | additional sex combs like 1 | 5.651e-05 | 0.003906 | 1.451 | 0.5371 | 5.087 | 0.3783 | 0.7113 | 1.081 | 0.1126 | 0.9011 | 0.03939 | 0.1521 | 1.193 | 0.255 | 2.204 | 2.587e-08 | 2.998e-06 | 2.026 | 1.019 | 8.807 |  |
| Fam46a | NA | 6.964e-06 | 0.001001 | 2.628 | 1.394 | 6.02 | 0.003641 | 0.07106 | 1.869 | 0.9021 | 3.293 | 0.01583 | 0.07958 | 1.59 | 0.6688 | 2.637 | 0.004704 | 0.03233 | 1.749 | 0.8068 | 3.181 |  |
| Gm3579 | NA | 0.09134 | 0.3258 | 1.709 | 0.773 | 1.774 | 0.0001718 | 0.008561 | 5.186 | 2.375 | 4.605 | 1.428e-07 | 1.113e-05 | 0.07324 | -3.771 | -7.9 | 0.04273 | 0.1583 | 0.4886 | -1.033 | -2.164 |  |
| Nfic | nuclear factor I/C | 8.709e-05 | 0.005044 | 0.6121 | -0.7082 | -4.899 | 0.2299 | 0.5912 | 0.8634 | -0.2118 | -1.238 | 8.386e-07 | 4.367e-05 | 0.4629 | -1.111 | -7.015 | 0.02698 | 0.115 | 0.7695 | -0.3781 | -2.387 |  |
| Stxbp4 | syntaxin binding protein 4 | 5.799e-05 | 0.003957 | 0.4886 | -1.033 | -5.076 | 0.0751 | 0.3752 | 0.7309 | -0.4523 | -1.878 | 0.2388 | 0.4868 | 0.8289 | -0.2708 | -1.214 | 1.992e-06 | 8.429e-05 | 0.3604 | -1.472 | -6.601 |  |
| Orai3 | ORAI calcium release-activated | 0.02839 | 0.1702 | 1.285 | 0.3622 | 2.363 | 0.119 | 0.4547 | 0.8148 | -0.2955 | -1.629 | 3.381e-07 | 2.174e-05 | 2.384 | 1.253 | 7.463 | 0.0003601 | 0.00471 | 1.647 | 0.72 | 4.287 |  |
| Megf9 | multiple EGF-like-domains 9 | 0.03869 | 0.2035 | 0.749 | -0.4169 | -2.213 | 0.07834 | 0.3834 | 1.332 | 0.4136 | 1.856 | 8.408e-08 | 7.536e-06 | 0.3106 | -1.687 | -8.175 | 0.0008932 | 0.009348 | 0.5726 | -0.8045 | -3.899 |  |
| Desi2 | desumoylating isopeptidase 2 | 0.9276 | 0.9744 | 0.989 | -0.0159 | -0.09203 | 0.0004636 | 0.01767 | 1.808 | 0.8543 | 4.179 | 6.475e-05 | 0.001219 | 0.5171 | -0.9515 | -5.027 | 2.782e-08 | 3.193e-06 | 0.3166 | -1.659 | -8.767 |  |
| Ddhd1 | DDHD domain containing 1 | 0.0001436 | 0.006817 | 1.881 | 0.9112 | 4.682 | 0.4931 | 0.7833 | 1.118 | 0.1608 | 0.6983 | 6.9e-05 | 0.001272 | 2.093 | 1.066 | 5 | 1.929e-05 | 0.0004914 | 2.274 | 1.185 | 5.561 |  |
| Gck | glucokinase | 0.09674 | 0.3356 | 0.6071 | -0.72 | -1.743 | 0.0002511 | 0.01134 | 0.222 | -2.171 | -4.442 | 0.001426 | 0.01344 | 3.19 | 1.674 | 3.698 | 8.633e-06 | 0.0002622 | 6.41 | 2.68 | 5.922 |  |
| Zfp36l1 | zinc finger protein 36, C3H ty | 0.4326 | 0.7139 | 1.142 | 0.1911 | 0.8009 | 0.005312 | 0.09007 | 0.5423 | -0.8828 | -3.127 | 6.919e-06 | 0.0002225 | 2.978 | 1.574 | 6.023 | 3.764e-06 | 0.0001356 | 3.133 | 1.647 | 6.303 |  |
| Dot1l | DOT1-like, histone H3 methyltr | 0.2427 | 0.5457 | 0.8836 | -0.1785 | -1.204 | 0.01029 | 0.1331 | 1.411 | 0.4971 | 2.833 | 6.017e-08 | 5.858e-06 | 0.3905 | -1.357 | -8.351 | 0.0005267 | 0.006272 | 0.6285 | -0.6701 | -4.125 |  |
| Tfrc | transferrin receptor | 0.0005308 | 0.01522 | 0.4289 | -1.221 | -4.121 | 0.4374 | 0.7509 | 1.212 | 0.2779 | 0.7925 | 0.006507 | 0.04145 | 0.5048 | -0.9861 | -3.038 | 8.424e-10 | 2.124e-07 | 0.08768 | -3.512 | -10.82 |  |
| Sparcl1 | SPARC-like 1 | 0.09795 | 0.3378 | 0.7643 | -0.3879 | -1.736 | 0.04315 | 0.2897 | 1.485 | 0.5708 | 2.159 | 3.621e-06 | 0.0001316 | 0.3422 | -1.547 | -6.321 | 2.586e-05 | 0.0006143 | 0.398 | -1.329 | -5.431 |  |
| Ppp1r15b | protein phosphatase 1, regulat | 0.01636 | 0.1233 | 1.376 | 0.4607 | 2.621 | 0.1889 | 0.5501 | 0.822 | -0.2829 | -1.36 | 9.651e-06 | 0.000284 | 2.189 | 1.13 | 5.872 | 1.366e-05 | 0.0003774 | 2.144 | 1.1 | 5.715 |  |
| Nos2 | nitric oxide synthase 2, induc | 0.0003709 | 0.0122 | 0.5879 | -0.7663 | -4.275 | 0.783 | 0.9214 | 1.042 | 0.05922 | 0.2792 | 4.961e-08 | 4.908e-06 | 0.3164 | -1.66 | -8.454 | 0.0004879 | 0.005919 | 0.5678 | -0.8164 | -4.157 |  |
| Hdac9 | histone deacetylase 9 | 6.638e-05 | 0.00433 | 0.6469 | -0.6283 | -5.017 | 0.5497 | 0.8139 | 0.9394 | -0.09018 | -0.6085 | 0.005485 | 0.03647 | 0.7438 | -0.4271 | -3.113 | 3.425e-08 | 3.749e-06 | 0.4391 | -1.187 | -8.654 |  |
| Acss1 | acyl-CoA synthetase short-chai | 0.0009071 | 0.02127 | 1.652 | 0.7245 | 3.892 | 0.44 | 0.7527 | 1.128 | 0.1735 | 0.788 | 0.004594 | 0.03199 | 0.637 | -0.6507 | -3.191 | 3.978e-10 | 1.137e-07 | 4.937 | 2.304 | 11.3 |  |
| Lrrc38 | leucine rich repeat containing | 1.69e-06 | 0.0003779 | 0.4289 | -1.221 | -6.679 | 0.3056 | 0.6594 | 0.8541 | -0.2275 | -1.051 | 0.7524 | 0.8855 | 0.9566 | -0.06408 | -0.3199 | 4.213e-12 | 3.871e-09 | 0.1323 | -2.918 | -14.57 |  |
| Odf3l1 | outer dense fiber of sperm tai | 7.588e-05 | 0.004651 | 0.6291 | -0.6685 | -4.958 | 0.4036 | 0.7297 | 1.099 | 0.1361 | 0.8534 | 3.006e-12 | 4.257e-09 | 0.219 | -2.191 | -14.84 | 0.1469 | 0.3536 | 0.8569 | -0.2229 | -1.509 |  |
| Thbs1 | thrombospondin 1 | 0.03773 | 0.2008 | 1.633 | 0.7077 | 2.225 | 0.05491 | 0.3237 | 0.5876 | -0.7672 | -2.039 | 6.669e-09 | 1.129e-06 | 10.08 | 3.334 | 9.57 | 0.005258 | 0.03503 | 2.13 | 1.091 | 3.132 |  |
| Creld2 | cysteine-rich with EGF-like do | 0.2141 | 0.5127 | 1.227 | 0.2947 | 1.283 | 0.0003763 | 0.01503 | 2.234 | 1.16 | 4.269 | 1.418e-05 | 0.0003823 | 0.3702 | -1.434 | -5.698 | 0.0001662 | 0.002576 | 0.4469 | -1.162 | -4.619 |  |
| Fbxo45 | F-box protein 45 | 0.01346 | 0.1099 | 0.7074 | -0.4994 | -2.711 | 0.2162 | 0.5779 | 1.213 | 0.2783 | 1.277 | 1.65e-06 | 7.144e-05 | 0.3923 | -1.35 | -6.691 | 9.272e-05 | 0.001665 | 0.5059 | -0.983 | -4.871 |  |
| Afap1l1 | actin filament associated prot | 0.0001197 | 0.006134 | 0.6272 | -0.6731 | -4.761 | 0.3719 | 0.7075 | 0.8995 | -0.1528 | -0.9135 | 0.002368 | 0.01961 | 0.6883 | -0.5389 | -3.48 | 2.004e-06 | 8.464e-05 | 0.4925 | -1.022 | -6.598 |  |
| Gpc4 | glypican 4 | 9.578e-07 | 0.0002439 | 0.4443 | -1.17 | -6.951 | 0.0002402 | 0.01103 | 0.5401 | -0.8888 | -4.461 | 0.003185 | 0.02444 | 0.6515 | -0.6181 | -3.351 | 0.233 | 0.4656 | 0.8545 | -0.2269 | -1.23 |  |
| Setdb2 | SET domain, bifurcated 2 | 0.0014 | 0.02808 | 1.862 | 0.897 | 3.706 | 8.285e-05 | 0.005194 | 2.656 | 1.409 | 4.92 | 0.1327 | 0.3393 | 0.7497 | -0.4156 | -1.568 | 0.0004153 | 0.005252 | 0.4599 | -1.121 | -4.226 |  |
| Dbf4 | DBF4 zinc finger | 0.2489 | 0.5526 | 1.175 | 0.2327 | 1.188 | 0.01239 | 0.148 | 0.6429 | -0.6374 | -2.749 | 3.811e-05 | 0.000815 | 2.187 | 1.129 | 5.259 | 1.064e-06 | 5.175e-05 | 2.792 | 1.481 | 6.9 |  |
| Vnn1 | vanin 1 | 1.066e-05 | 0.001362 | 4.447 | 2.153 | 5.827 | 0.001814 | 0.04543 | 2.972 | 1.571 | 3.595 | 0.0001099 | 0.001858 | 3.842 | 1.942 | 4.798 | 0.6309 | 0.8051 | 0.8721 | -0.1975 | -0.4879 |  |
| Pde4a | phosphodiesterase 4A, cAMP spe | 5.252e-05 | 0.003711 | 0.5213 | -0.9398 | -5.119 | 0.1309 | 0.4719 | 0.7889 | -0.3422 | -1.575 | 0.654 | 0.8317 | 0.9385 | -0.09152 | -0.4551 | 4.912e-08 | 4.994e-06 | 0.3075 | -1.701 | -8.46 |  |
| Fam214a | family with sequence similarit | 9.395e-08 | 4.592e-05 | 3.111 | 1.638 | 8.117 | 1.624e-06 | 0.0003027 | 3.029 | 1.599 | 6.698 | 0.07375 | 0.2316 | 1.335 | 0.4171 | 1.887 | 0.1881 | 0.41 | 0.8116 | -0.3012 | -1.363 |  |
| Slc22a30 | solute carrier family 22, memb | 0.05481 | 0.2478 | 0.6949 | -0.5251 | -2.04 | 0.0002437 | 0.01109 | 0.3904 | -1.357 | -4.455 | 8.851e-05 | 0.001564 | 2.602 | 1.379 | 4.892 | 0.0007789 | 0.008436 | 2.167 | 1.116 | 3.957 |  |
| Maged1 | melanoma antigen, family D, 1 | 0.04132 | 0.2112 | 0.7626 | -0.391 | -2.181 | 0.09606 | 0.4149 | 1.293 | 0.3706 | 1.747 | 1.06e-06 | 5.173e-05 | 0.3907 | -1.356 | -6.902 | 0.0001286 | 0.002124 | 0.5252 | -0.929 | -4.73 |  |
| Slc6a6 | solute carrier family 6 (neuro | 0.04442 | 0.22 | 1.309 | 0.3882 | 2.145 | 0.01186 | 0.1442 | 0.6631 | -0.5928 | -2.769 | 3.332e-11 | 2.253e-08 | 5.964 | 2.576 | 13 | 0.07934 | 0.24 | 1.289 | 0.3665 | 1.849 |  |
| Ppp1r14c | protein phosphatase 1, regulat | 3.887e-05 | 0.003078 | 0.6023 | -0.7314 | -5.251 | 0.1291 | 0.4696 | 0.8346 | -0.2609 | -1.583 | 0.8447 | 0.934 | 0.9792 | -0.03028 | -0.1985 | 1.661e-08 | 2.105e-06 | 0.3839 | -1.381 | -9.052 |  |
| Xdh | xanthine dehydrogenase | 0.0003565 | 0.01199 | 1.517 | 0.6009 | 4.292 | 0.8152 | 0.9363 | 0.9732 | -0.03923 | -0.2368 | 0.0001596 | 0.002478 | 1.637 | 0.7112 | 4.637 | 1.472e-07 | 1.105e-05 | 2.312 | 1.209 | 7.884 |  |
| Cyp1b1 | cytochrome P450, family 1, sub | 2.841e-05 | 0.002548 | 4.568 | 2.191 | 5.389 | 0.02457 | 0.2157 | 2.25 | 1.17 | 2.432 | 0.0005346 | 0.006299 | 3.567 | 1.835 | 4.118 | 0.01206 | 0.06446 | 2.346 | 1.23 | 2.761 |  |
| Mir185 | microRNA 185 | 1.061e-05 | 0.001362 | 2.419 | 1.274 | 5.829 | 5.875e-06 | 0.0007594 | 2.985 | 1.578 | 6.098 | 0.002408 | 0.01987 | 0.5619 | -0.8316 | -3.472 | 0.7488 | 0.8793 | 0.9475 | -0.07775 | -0.3246 |  |
| Nufip1 | nuclear fragile X mental retar | 7.596e-05 | 0.004651 | 1.595 | 0.6735 | 4.958 | 0.1722 | 0.53 | 1.171 | 0.2276 | 1.416 | 0.297 | 0.5516 | 1.117 | 0.1594 | 1.071 | 1.376e-07 | 1.058e-05 | 2.263 | 1.178 | 7.919 |  |
| Dcaf4 | DDB1 and CUL4 associated facto | 0.006995 | 0.07482 | 0.7138 | -0.4864 | -3.006 | 0.2604 | 0.6189 | 1.166 | 0.2218 | 1.158 | 7.219e-08 | 6.656e-06 | 0.3626 | -1.463 | -8.255 | 0.001326 | 0.01253 | 0.6324 | -0.6611 | -3.729 |  |
| Elmo3 | engulfment and cell motility 3 | 1.305e-05 | 0.001498 | 0.6372 | -0.6502 | -5.736 | 0.6627 | 0.8713 | 0.9597 | -0.05938 | -0.4427 | 9.879e-11 | 5.082e-08 | 0.349 | -1.519 | -12.23 | 0.0543 | 0.1866 | 0.8386 | -0.2539 | -2.045 |  |
| Usp54 | ubiquitin specific peptidase 5 | 0.0001375 | 0.006625 | 1.831 | 0.8725 | 4.701 | 0.5291 | 0.8023 | 1.102 | 0.1407 | 0.6406 | 3.095e-07 | 2.026e-05 | 2.88 | 1.526 | 7.507 | 0.003651 | 0.02696 | 1.59 | 0.6692 | 3.292 |  |
| Gnptab | N-acetylglucosamine-1-phosphat | 0.0001049 | 0.005646 | 0.6085 | -0.7167 | -4.818 | 0.2585 | 0.6173 | 0.8677 | -0.2047 | -1.163 | 3.736e-07 | 2.362e-05 | 0.4329 | -1.208 | -7.413 | 0.05781 | 0.1944 | 0.7966 | -0.328 | -2.013 |  |
| Acsm5 | acyl-CoA synthetase medium-cha | 0.04457 | 0.2202 | 1.347 | 0.4293 | 2.143 | 0.05535 | 0.3244 | 0.7159 | -0.4822 | -2.035 | 1.006e-08 | 1.549e-06 | 4.135 | 2.048 | 9.334 | 0.00528 | 0.03511 | 1.61 | 0.6867 | 3.13 |  |
| BC021614 | NA | 0.8156 | 0.9303 | 0.9652 | -0.05117 | -0.2364 | 0.000714 | 0.02401 | 0.492 | -1.023 | -3.994 | 4.232e-07 | 2.586e-05 | 3.348 | 1.743 | 7.351 | 7.815e-05 | 0.001456 | 2.254 | 1.173 | 4.946 |  |
| Pbx1 | pre B cell leukemia homeobox 1 | 0.1985 | 0.4926 | 1.111 | 0.1523 | 1.33 | 0.0001633 | 0.008255 | 1.544 | 0.627 | 4.627 | 0.04394 | 0.1637 | 0.8294 | -0.2698 | -2.15 | 1.274e-08 | 1.733e-06 | 0.4493 | -1.154 | -9.2 |  |
| Hlf | hepatic leukemia factor | 0.6721 | 0.8635 | 1.057 | 0.07944 | 0.4296 | 0.0002781 | 0.01217 | 1.948 | 0.9622 | 4.398 | 0.001065 | 0.01067 | 0.5846 | -0.7744 | -3.823 | 1.164e-08 | 1.609e-06 | 0.2728 | -1.874 | -9.251 |  |
| Per1 | period circadian clock 1 | 4.565e-12 | 5.872e-08 | 5.071 | 2.342 | 14.5 | 3.353e-11 | 9.583e-08 | 5.591 | 2.483 | 12.99 | 0.1418 | 0.3537 | 0.8289 | -0.2707 | -1.53 | 0.4019 | 0.6332 | 0.9003 | -0.1515 | -0.8565 |  |
| Atf5 | activating transcription facto | 0.2259 | 0.5261 | 1.22 | 0.2871 | 1.249 | 0.0004529 | 0.01731 | 2.202 | 1.139 | 4.189 | 1.77e-05 | 0.0004521 | 0.3764 | -1.41 | -5.599 | 0.0001935 | 0.002908 | 0.4518 | -1.146 | -4.554 |  |
| Cdh23 | cadherin 23 (otocadherin) | 6.669e-05 | 0.004332 | 0.5259 | -0.9273 | -5.015 | 0.3936 | 0.7228 | 0.8761 | -0.1908 | -0.872 | 1.318e-08 | 1.894e-06 | 0.2755 | -1.86 | -9.181 | 0.09974 | 0.2774 | 0.7848 | -0.3497 | -1.726 |  |
| Nt5dc2 | 5'-nucleotidase domain contain | 0.1686 | 0.4517 | 1.345 | 0.4274 | 1.428 | 0.0003874 | 0.0154 | 2.842 | 1.507 | 4.256 | 7.362e-06 | 0.0002329 | 0.2561 | -1.965 | -5.995 | 0.0009227 | 0.009578 | 0.4137 | -1.273 | -3.885 |  |
| Ptk6 | PTK6 protein tyrosine kinase 6 | 0.3248 | 0.6254 | 0.8759 | -0.1911 | -1.009 | 0.0005347 | 0.01951 | 0.5276 | -0.9224 | -4.118 | 5.639e-06 | 0.0001896 | 2.409 | 1.268 | 6.117 | 0.0002389 | 0.003443 | 1.899 | 0.9256 | 4.463 |  |
| Ctps2 | cytidine 5'-triphosphate synth | 0.002636 | 0.04066 | 1.611 | 0.6883 | 3.433 | 0.002219 | 0.05178 | 1.78 | 0.8321 | 3.508 | 0.0001275 | 0.002081 | 0.4864 | -1.04 | -4.734 | 0.01184 | 0.06384 | 1.524 | 0.6083 | 2.769 |  |
| 6230400D17Rik | RIKEN cDNA 6230400D17 gene | 0.0001056 | 0.005668 | 1.866 | 0.8996 | 4.815 | 0.1652 | 0.5224 | 1.247 | 0.3185 | 1.441 | 0.05536 | 0.1909 | 1.335 | 0.4164 | 2.035 | 2.557e-06 | 0.0001009 | 2.509 | 1.327 | 6.484 |  |
| Dhcr24 | 24-dehydrocholesterol reductas | 0.001463 | 0.0288 | 0.5881 | -0.7658 | -3.687 | 0.6151 | 0.848 | 1.091 | 0.1255 | 0.5108 | 9.757e-07 | 4.921e-05 | 0.3346 | -1.58 | -6.942 | 0.0001067 | 0.001845 | 0.4683 | -1.095 | -4.81 |  |
| Tmem71 | transmembrane protein 71 | 6.7e-05 | 0.004341 | 1.827 | 0.8699 | 5.013 | 0.04958 | 0.3088 | 1.346 | 0.4292 | 2.09 | 0.4286 | 0.6741 | 1.112 | 0.1536 | 0.808 | 5.292e-06 | 0.0001805 | 2.248 | 1.168 | 6.146 |  |
| 9630013D21Rik | RIKEN cDNA 9630013D21 gene | 0.000249 | 0.009689 | 0.5102 | -0.9707 | -4.446 | 0.7415 | 0.9054 | 1.062 | 0.08641 | 0.3344 | 2.928e-09 | 6.075e-07 | 0.1888 | -2.405 | -10.05 | 0.004449 | 0.03105 | 0.5878 | -0.7667 | -3.205 |  |
| Inf2 | inverted formin, FH2 and WH2 d | 0.5955 | 0.8218 | 1.061 | 0.08511 | 0.5395 | 0.003425 | 0.06839 | 0.6509 | -0.6196 | -3.319 | 4.106e-07 | 2.527e-05 | 2.416 | 1.273 | 7.366 | 9.35e-05 | 0.001675 | 1.792 | 0.8412 | 4.868 |  |
| Lrrc58 | leucine rich repeat containing | 0.0008992 | 0.0212 | 1.77 | 0.8237 | 3.896 | 0.5966 | 0.8376 | 0.911 | -0.1345 | -0.5378 | 0.0008481 | 0.008912 | 1.877 | 0.9081 | 3.921 | 4.632e-08 | 4.813e-06 | 3.908 | 1.967 | 8.491 |  |
| Slc22a23 | solute carrier family 22, memb | 0.1704 | 0.4542 | 0.881 | -0.1828 | -1.422 | 0.0003176 | 0.01333 | 0.6329 | -0.66 | -4.341 | 4.072e-07 | 2.512e-05 | 2.052 | 1.037 | 7.37 | 0.01101 | 0.06043 | 1.314 | 0.3944 | 2.802 |  |
| Gm4876 | predicted gene 4876 | 0.01921 | 0.1352 | 0.7075 | -0.4992 | -2.547 | 0.06569 | 0.3524 | 1.368 | 0.4516 | 1.947 | 5.678e-10 | 1.826e-07 | 0.1926 | -2.376 | -11.07 | 0.03829 | 0.1469 | 0.7188 | -0.4762 | -2.218 |  |
| Pole4 | polymerase (DNA-directed), eps | 0.1738 | 0.4593 | 1.219 | 0.2857 | 1.41 | 0.0003166 | 0.01333 | 2.057 | 1.041 | 4.343 | 0.003695 | 0.02729 | 0.6032 | -0.7292 | -3.286 | 1.016e-06 | 4.969e-05 | 0.3448 | -1.536 | -6.923 |  |
| Slc16a10 | solute carrier family 16 (mono | 0.2545 | 0.5585 | 0.8672 | -0.2056 | -1.173 | 0.007365 | 0.1109 | 1.535 | 0.6184 | 2.983 | 0.001433 | 0.01348 | 0.6115 | -0.7095 | -3.696 | 1.316e-08 | 1.754e-06 | 0.2947 | -1.763 | -9.182 |  |
| Trpv2 | transient receptor potential c | 9.781e-05 | 0.005446 | 2.532 | 1.34 | 4.848 | 0.1394 | 0.4839 | 1.418 | 0.5035 | 1.539 | 2.696e-06 | 0.0001056 | 3.879 | 1.956 | 6.459 | 0.08216 | 0.2455 | 1.468 | 0.5542 | 1.83 |  |
| Steap4 | STEAP family member 4 | 1.716e-05 | 0.001808 | 0.3859 | -1.374 | -5.613 | 0.001261 | 0.03632 | 0.471 | -1.086 | -3.751 | 0.0005292 | 0.006242 | 0.4648 | -1.105 | -4.123 | 0.3764 | 0.6106 | 1.183 | 0.2426 | 0.9048 |  |
| Fsd1l | fibronectin type III and SPRY | 0.0001009 | 0.005512 | 0.5646 | -0.8246 | -4.835 | 0.1647 | 0.5217 | 0.8173 | -0.2911 | -1.442 | 0.1809 | 0.4107 | 0.8356 | -0.2591 | -1.386 | 5.98e-07 | 3.218e-05 | 0.3946 | -1.342 | -7.18 |  |
| Sbds | SBDS ribosome maturation facto | 0.000108 | 0.00574 | 1.608 | 0.6854 | 4.805 | 0.3269 | 0.6742 | 1.125 | 0.1696 | 1.005 | 0.03352 | 0.136 | 1.281 | 0.3567 | 2.283 | 2.469e-07 | 1.624e-05 | 2.283 | 1.191 | 7.62 |  |
| Clcn1 | chloride channel, voltage-sens | 0.0003345 | 0.01146 | 0.6716 | -0.5744 | -4.319 | 0.2269 | 0.5883 | 0.8728 | -0.1962 | -1.247 | 0.2065 | 0.4454 | 1.141 | 0.1902 | 1.306 | 1.541e-08 | 1.998e-06 | 0.3992 | -1.325 | -9.094 |  |
| Sulf2 | sulfatase 2 | 0.8876 | 0.9574 | 1.027 | 0.03849 | 0.1432 | 0.000865 | 0.02761 | 2.369 | 1.244 | 3.912 | 2.072e-06 | 8.499e-05 | 0.2609 | -1.938 | -6.583 | 1.404e-05 | 0.0003845 | 0.3123 | -1.679 | -5.703 |  |
| Utp14a | UTP14A small subunit processom | 0.0001436 | 0.006817 | 1.585 | 0.6644 | 4.682 | 0.3529 | 0.6943 | 1.117 | 0.1597 | 0.9511 | 0.003894 | 0.02842 | 1.421 | 0.5073 | 3.263 | 2.592e-06 | 0.000102 | 2.01 | 1.007 | 6.477 |  |
| Tcn2 | transcobalamin 2 | 0.0001261 | 0.006309 | 1.654 | 0.726 | 4.739 | 0.8049 | 0.9316 | 1.032 | 0.04538 | 0.2503 | 2.478e-08 | 3.035e-06 | 2.794 | 1.482 | 8.83 | 0.003301 | 0.02494 | 1.474 | 0.5598 | 3.335 |  |
| Ache | acetylcholinesterase | 6.523e-05 | 0.004291 | 0.5957 | -0.7474 | -5.024 | 0.48 | 0.7753 | 0.9159 | -0.1267 | -0.7198 | 0.05782 | 0.1968 | 0.7966 | -0.328 | -2.013 | 8.738e-09 | 1.307e-06 | 0.3453 | -1.534 | -9.414 |  |
| Nr1d1 | nuclear receptor subfamily 1, | 1.584e-07 | 6.496e-05 | 0.2853 | -1.81 | -7.846 | 1.536e-05 | 0.001485 | 0.3426 | -1.545 | -5.663 | 0.6069 | 0.8024 | 0.9125 | -0.1321 | -0.5227 | 0.01656 | 0.08075 | 0.6325 | -0.6609 | -2.616 |  |
| Nid2 | nidogen 2 | 3.524e-07 | 0.0001225 | 0.3559 | -1.491 | -7.442 | 2.541e-05 | 0.002164 | 0.4093 | -1.289 | -5.438 | 0.005165 | 0.03486 | 0.6203 | -0.6889 | -3.14 | 0.7068 | 0.8545 | 1.06 | 0.08374 | 0.3816 |  |
| Tars | threonyl-tRNA synthetase | 0.3672 | 0.6619 | 0.8911 | -0.1664 | -0.9226 | 0.0004893 | 0.01837 | 0.5408 | -0.8869 | -4.156 | 0.0004186 | 0.005217 | 1.783 | 0.8343 | 4.223 | 1.557e-06 | 6.954e-05 | 2.509 | 1.327 | 6.718 |  |
| Rell1 | RELT-like 1 | 0.003725 | 0.04984 | 0.6609 | -0.5974 | -3.283 | 0.000102 | 0.006022 | 0.4863 | -1.04 | -4.83 | 0.001249 | 0.01212 | 1.68 | 0.7485 | 3.755 | 0.008746 | 0.05085 | 1.494 | 0.5793 | 2.906 |  |
| Reep6 | receptor accessory protein 6 | 0.07031 | 0.283 | 0.7882 | -0.3433 | -1.912 | 0.07223 | 0.3681 | 1.323 | 0.4033 | 1.898 | 7.149e-07 | 3.857e-05 | 0.3802 | -1.395 | -7.093 | 0.0003547 | 0.004664 | 0.5569 | -0.8446 | -4.294 |  |
| Ppif | peptidylprolyl isomerase F (cy | 0.3404 | 0.6395 | 0.8768 | -0.1896 | -0.9766 | 0.01016 | 0.1319 | 1.572 | 0.6522 | 2.838 | 4.142e-07 | 2.543e-05 | 0.3378 | -1.566 | -7.361 | 0.0002139 | 0.003159 | 0.5142 | -0.9596 | -4.511 |  |
| 2310015D24Rik | RIKEN cDNA 2310015D24 gene | 5.747e-05 | 0.003932 | 0.3611 | -1.47 | -5.08 | 0.1606 | 0.5153 | 0.7077 | -0.4988 | -1.457 | 0.9377 | 0.9749 | 1.018 | 0.02507 | 0.07912 | 1.13e-08 | 1.58e-06 | 0.1305 | -2.937 | -9.268 |  |
| Fam96a | NA | 0.1803 | 0.4688 | 0.8537 | -0.2281 | -1.388 | 0.0259 | 0.2218 | 1.383 | 0.4679 | 2.407 | 1.256e-06 | 5.894e-05 | 0.427 | -1.228 | -6.821 | 0.0001147 | 0.001946 | 0.5508 | -0.8603 | -4.779 |  |
| Mrpl48 | mitochondrial ribosomal protei | 0.1275 | 0.3902 | 0.8182 | -0.2894 | -1.59 | 0.03896 | 0.2761 | 1.391 | 0.4759 | 2.21 | 3.632e-05 | 0.0007877 | 0.482 | -1.053 | -5.281 | 4.372e-06 | 0.0001528 | 0.4225 | -1.243 | -6.234 |  |
| Ms4a6c | membrane-spanning 4-domains, s | 0.0001786 | 0.007906 | 2.893 | 1.533 | 4.588 | 0.3401 | 0.6841 | 1.307 | 0.3863 | 0.9773 | 0.0005413 | 0.006347 | 2.838 | 1.505 | 4.113 | 3.585e-05 | 0.0007992 | 3.822 | 1.934 | 5.286 |  |
| Cnst | consortin, connexin sorting pr | 0.004263 | 0.05445 | 1.641 | 0.7142 | 3.224 | 0.03567 | 0.2656 | 1.506 | 0.5906 | 2.253 | 4.553e-05 | 0.0009331 | 2.391 | 1.257 | 5.181 | 0.001584 | 0.01434 | 0.5409 | -0.8865 | -3.653 |  |
| Lmbr1l | limb region 1 like | 0.03684 | 0.198 | 1.313 | 0.3931 | 2.237 | 0.1251 | 0.4644 | 0.794 | -0.3328 | -1.601 | 1.562e-05 | 0.0004126 | 2.126 | 1.088 | 5.655 | 1.547e-05 | 0.0004141 | 2.128 | 1.089 | 5.659 |  |
| Aldoc | aldolase C, fructose-bisphosph | 0.01158 | 0.1002 | 0.6158 | -0.6994 | -2.779 | 0.0001769 | 0.008686 | 0.3876 | -1.367 | -4.592 | 0.002778 | 0.02212 | 1.919 | 0.9401 | 3.41 | 0.0009789 | 0.01 | 2.091 | 1.064 | 3.859 |  |
| Asb2 | ankyrin repeat and SOCS box-co | 0.03325 | 0.1869 | 0.7562 | -0.4033 | -2.287 | 0.0002521 | 0.01134 | 0.5262 | -0.9264 | -4.44 | 0.01465 | 0.07519 | 1.43 | 0.5162 | 2.672 | 2.459e-05 | 0.0005918 | 2.075 | 1.053 | 5.453 |  |
| Xab2 | XPA binding protein 2 | 0.0001208 | 0.006157 | 1.501 | 0.5862 | 4.757 | 0.4531 | 0.7599 | 1.08 | 0.1116 | 0.7651 | 0.016 | 0.08014 | 1.279 | 0.3553 | 2.632 | 1.382e-07 | 1.058e-05 | 2.098 | 1.069 | 7.916 |  |
| Gm826 | predicted gene 826 | 0.003323 | 0.04687 | 1.807 | 0.8538 | 3.332 | 0.000105 | 0.006142 | 2.752 | 1.46 | 4.817 | 0.002589 | 0.02104 | 0.512 | -0.9657 | -3.441 | 0.006429 | 0.04063 | 0.5532 | -0.854 | -3.043 |  |
| Gata6 | GATA binding protein 6 | 0.006012 | 0.0689 | 1.6 | 0.6779 | 3.073 | 0.0001333 | 0.007234 | 2.347 | 1.231 | 4.714 | 0.0009454 | 0.009743 | 0.5225 | -0.9364 | -3.874 | 0.007289 | 0.04464 | 0.6063 | -0.722 | -2.987 |  |
| Cldnd1 | claudin domain containing 1 | 0.2905 | 0.5942 | 1.15 | 0.202 | 1.086 | 0.0005999 | 0.02117 | 1.861 | 0.8958 | 4.069 | 1.115e-05 | 0.0003202 | 0.4403 | -1.183 | -5.807 | 0.0002738 | 0.003832 | 0.5367 | -0.8978 | -4.405 |  |
| Zfp280b | zinc finger protein 280B | 4.401e-05 | 0.003316 | 0.5039 | -0.9889 | -5.196 | 0.01831 | 0.1844 | 0.6696 | -0.5786 | -2.569 | 0.3449 | 0.5995 | 0.8695 | -0.2017 | -0.9674 | 7.966e-05 | 0.001474 | 0.49 | -1.029 | -4.937 |  |
| Rbp7 | retinol binding protein 7, cel | 0.001942 | 0.03432 | 1.894 | 0.9216 | 3.565 | 0.1825 | 0.5431 | 0.7461 | -0.4225 | -1.381 | 3.388e-11 | 2.253e-08 | 12.79 | 3.677 | 12.98 | 0.2243 | 0.4551 | 1.279 | 0.3551 | 1.254 |  |
| Cnppd1 | cyclin Pas1/PHO80 domain conta | 0.0002804 | 0.01028 | 1.603 | 0.6811 | 4.395 | 0.949 | 0.9837 | 0.9918 | -0.01188 | -0.06478 | 7.38e-05 | 0.001343 | 1.795 | 0.8439 | 4.971 | 4.938e-07 | 2.761e-05 | 2.354 | 1.235 | 7.274 |  |
| Lncenc1 | long non-coding RNA, embryonic | 5.898e-06 | 0.0008923 | 1.699 | 0.7651 | 6.096 | 0.5186 | 0.7968 | 1.07 | 0.09758 | 0.6571 | 1.382e-12 | 2.37e-09 | 4.369 | 2.127 | 15.47 | 0.3747 | 0.6091 | 0.9171 | -0.1248 | -0.908 |  |
| Ube4a | ubiquitination factor E4A | 0.0005238 | 0.01508 | 1.776 | 0.8285 | 4.127 | 0.02271 | 0.2067 | 1.501 | 0.5864 | 2.469 | 0.07189 | 0.2275 | 0.7485 | -0.4179 | -1.901 | 4.331e-05 | 0.0009277 | 2.21 | 1.144 | 5.203 |  |
| Erbb4 | erb-b2 receptor tyrosine kinas | 0.003717 | 0.04982 | 0.6159 | -0.6992 | -3.284 | 0.000123 | 0.006873 | 0.4363 | -1.196 | -4.749 | 0.01551 | 0.07837 | 1.534 | 0.6172 | 2.646 | 0.001264 | 0.01211 | 1.834 | 0.8746 | 3.75 |  |
| Pnpla3 | patatin-like phospholipase dom | 0.003644 | 0.04914 | 0.3983 | -1.328 | -3.292 | 0.3834 | 0.7144 | 1.343 | 0.4254 | 0.8913 | 6.247e-08 | 6.019e-06 | 0.07792 | -3.682 | -8.331 | 0.001906 | 0.01654 | 0.3347 | -1.579 | -3.573 |  |
| Matn2 | matrilin 2 | 0.01093 | 0.09741 | 0.601 | -0.7345 | -2.806 | 0.129 | 0.4694 | 1.405 | 0.4905 | 1.584 | 1.205e-09 | 3.089e-07 | 0.1217 | -3.038 | -10.59 | 0.03807 | 0.1462 | 0.6431 | -0.637 | -2.221 |  |
| Mir133a-1 | microRNA 133a-1 | 0.008375 | 0.08331 | 0.6338 | -0.6579 | -2.925 | 0.11 | 0.4407 | 1.361 | 0.4451 | 1.672 | 0.07161 | 0.2269 | 0.7226 | -0.4688 | -1.903 | 2.795e-10 | 8.457e-08 | 0.1396 | -2.84 | -11.53 |  |
| Rnf170 | ring finger protein 170 | 0.0001278 | 0.00635 | 0.6487 | -0.6244 | -4.733 | 0.3173 | 0.6668 | 0.895 | -0.1601 | -1.026 | 0.05188 | 0.1826 | 0.8129 | -0.2988 | -2.068 | 2.731e-07 | 1.739e-05 | 0.4685 | -1.094 | -7.569 |  |
| Pnpla2 | patatin-like phospholipase dom | 5.5e-06 | 0.0008574 | 1.982 | 0.9869 | 6.128 | 2.15e-05 | 0.001901 | 2.071 | 1.05 | 5.512 | 0.03288 | 0.1343 | 0.7556 | -0.4044 | -2.292 | 0.2393 | 0.4726 | 1.16 | 0.214 | 1.213 |  |
| Ampd3 | adenosine monophosphate deamin | 9.94e-05 | 0.005463 | 1.866 | 0.8997 | 4.841 | 0.086 | 0.3972 | 1.317 | 0.3972 | 1.806 | 0.4682 | 0.7053 | 1.11 | 0.1506 | 0.7395 | 1.736e-06 | 7.556e-05 | 2.562 | 1.357 | 6.666 |  |
| Mir365-2 | microRNA 365-2 | 0.006854 | 0.07426 | 0.4993 | -1.002 | -3.015 | 0.3035 | 0.6574 | 1.334 | 0.4154 | 1.056 | 0.0004257 | 0.005291 | 0.3451 | -1.535 | -4.216 | 3.383e-07 | 2.052e-05 | 0.1521 | -2.717 | -7.462 |  |
| Gm9992 | NA | 4.986e-05 | 0.003603 | 2.246 | 1.168 | 5.142 | 0.02036 | 0.1956 | 1.599 | 0.6771 | 2.52 | 0.5769 | 0.7834 | 1.103 | 0.1411 | 0.5672 | 3.114e-05 | 0.0007119 | 2.515 | 1.331 | 5.348 |  |
| Epha2 | Eph receptor A2 | 0.2755 | 0.581 | 1.173 | 0.2298 | 1.121 | 0.0005757 | 0.02057 | 1.988 | 0.9913 | 4.087 | 0.0001823 | 0.002746 | 0.4902 | -1.028 | -4.58 | 1.798e-05 | 0.000466 | 0.4187 | -1.256 | -5.592 |  |
| Cyp7a1 | cytochrome P450, family 7, sub | 0.4701 | 0.7418 | 1.145 | 0.1957 | 0.7364 | 0.0007138 | 0.02401 | 2.388 | 1.256 | 3.995 | 7.75e-05 | 0.001401 | 0.3684 | -1.441 | -4.949 | 7.635e-06 | 0.0002404 | 0.2994 | -1.74 | -5.978 |  |
| Maob | monoamine oxidase B | 0.07194 | 0.2864 | 0.8023 | -0.3179 | -1.9 | 0.06678 | 0.3551 | 1.305 | 0.3838 | 1.939 | 2.292e-07 | 1.606e-05 | 0.3781 | -1.403 | -7.658 | 0.001052 | 0.01055 | 0.6149 | -0.7015 | -3.828 |  |
| Rsrp1 | arginine/serine rich protein 1 | 0.003339 | 0.04693 | 1.356 | 0.4394 | 3.33 | 0.4353 | 0.7497 | 0.9175 | -0.1243 | -0.7961 | 1.73e-07 | 1.301e-05 | 2.185 | 1.127 | 7.801 | 0.0008918 | 0.009337 | 1.478 | 0.5636 | 3.899 |  |
| Lrrc39 | leucine rich repeat containing | 1.097e-05 | 0.001362 | 0.3788 | -1.401 | -5.814 | 0.9754 | 0.9924 | 1.006 | 0.008909 | 0.03125 | 1.727e-11 | 1.587e-08 | 0.08494 | -3.557 | -13.48 | 0.01935 | 0.09034 | 0.628 | -0.6712 | -2.544 |  |
| Dock4 | dedicator of cytokinesis 4 | 1.777e-05 | 0.00185 | 0.636 | -0.6529 | -5.597 | 0.9741 | 0.992 | 0.9969 | -0.004531 | -0.03283 | 0.007398 | 0.04551 | 0.768 | -0.3808 | -2.981 | 9.691e-11 | 3.777e-08 | 0.3382 | -1.564 | -12.24 |  |
| Ky | kyphoscoliosis peptidase | 2.134e-08 | 1.444e-05 | 0.3684 | -1.441 | -8.913 | 0.4507 | 0.7586 | 0.9031 | -0.1471 | -0.7693 | 0.9533 | 0.9817 | 0.9927 | -0.01051 | -0.05934 | 2.026e-15 | 2.606e-11 | 0.0684 | -3.87 | -21.86 |  |
| Mocs1 | molybdenum cofactor synthesis | 0.001899 | 0.03392 | 1.601 | 0.6794 | 3.575 | 0.4988 | 0.7858 | 0.8982 | -0.1549 | -0.6888 | 0.001048 | 0.01056 | 1.738 | 0.7974 | 3.83 | 8.135e-08 | 7.191e-06 | 3.261 | 1.705 | 8.192 |  |
| D430041D05Rik | RIKEN cDNA D430041D05 gene | 2.792e-05 | 0.002531 | 0.5405 | -0.8876 | -5.397 | 0.2645 | 0.6219 | 0.8565 | -0.2234 | -1.148 | 0.8818 | 0.9503 | 0.9814 | -0.02713 | -0.1506 | 7.289e-10 | 1.931e-07 | 0.2561 | -1.965 | -10.91 |  |
| Bmp1 | bone morphogenetic protein 1 | 0.1588 | 0.4376 | 0.8506 | -0.2335 | -1.464 | 0.02707 | 0.2272 | 1.366 | 0.4503 | 2.386 | 0.0001859 | 0.002785 | 0.5748 | -0.7988 | -4.571 | 6.139e-07 | 3.28e-05 | 0.4197 | -1.252 | -7.167 |  |
| AI607873 | NA | 3.278e-05 | 0.002801 | 2.321 | 1.215 | 5.326 | 0.01355 | 0.1547 | 1.66 | 0.7308 | 2.708 | 0.04368 | 0.163 | 1.452 | 0.538 | 2.153 | 0.001567 | 0.0142 | 1.884 | 0.9138 | 3.657 |  |
| Slc9a1 | solute carrier family 9 (sodiu | 0.0008177 | 0.0199 | 1.407 | 0.4928 | 3.936 | 0.1927 | 0.5537 | 0.8707 | -0.1997 | -1.348 | 0.5864 | 0.7905 | 1.054 | 0.07584 | 0.553 | 4.053e-12 | 3.861e-09 | 4.005 | 2.002 | 14.6 |  |
| Ick | intestinal cell kinase | 0.09857 | 0.3392 | 0.8414 | -0.2492 | -1.733 | 0.0003438 | 0.01415 | 0.6017 | -0.7329 | -4.307 | 0.02465 | 0.1096 | 1.304 | 0.3828 | 2.43 | 1.362e-06 | 6.266e-05 | 2.097 | 1.068 | 6.782 |  |
| Fcamr | Fc receptor, IgA, IgM, high af | 0.002274 | 0.03749 | 1.915 | 0.9376 | 3.497 | 0.0001348 | 0.007282 | 2.817 | 1.494 | 4.71 | 0.2914 | 0.5466 | 0.802 | -0.3183 | -1.084 | 0.0001737 | 0.002672 | 0.392 | -1.351 | -4.6 |  |
| Fam13a | family with sequence similarit | 1.966e-05 | 0.001983 | 3.241 | 1.697 | 5.552 | 0.001285 | 0.03672 | 2.555 | 1.353 | 3.743 | 0.000901 | 0.009349 | 2.469 | 1.304 | 3.895 | 0.4234 | 0.6509 | 0.8273 | -0.2735 | -0.8173 |  |
| Lrat | lecithin-retinol acyltransfera | 0.421 | 0.706 | 0.9028 | -0.1476 | -0.8215 | 0.0007209 | 0.02406 | 0.5555 | -0.8481 | -3.99 | 7.257e-05 | 0.001325 | 1.972 | 0.9795 | 4.978 | 1.405e-05 | 0.0003845 | 2.177 | 1.122 | 5.703 |  |
| Echdc1 | enoyl Coenzyme A hydratase dom | 0.0002437 | 0.009528 | 0.5091 | -0.9741 | -4.455 | 4.859e-05 | 0.003425 | 0.3969 | -1.333 | -5.153 | 0.09392 | 0.2721 | 1.339 | 0.4213 | 1.759 | 0.01267 | 0.0669 | 1.576 | 0.6559 | 2.738 |  |
| Cdkn2c | cyclin dependent kinase inhibi | 0.004573 | 0.05736 | 0.5924 | -0.7555 | -3.193 | 0.3785 | 0.7113 | 1.191 | 0.2521 | 0.9006 | 1.122e-07 | 9.218e-06 | 0.2366 | -2.08 | -8.024 | 0.001637 | 0.01473 | 0.5201 | -0.943 | -3.639 |  |
| Smco1 | single-pass membrane protein w | 0.0001284 | 0.00635 | 0.5657 | -0.8218 | -4.731 | 0.02326 | 0.2094 | 0.7046 | -0.5051 | -2.457 | 0.5438 | 0.762 | 1.085 | 0.1175 | 0.6176 | 1.728e-05 | 0.0004516 | 0.4771 | -1.068 | -5.61 |  |
| Glns-ps1 | glutamine synthetase pseudogen | 0.000125 | 0.006278 | 2.067 | 1.047 | 4.742 | 0.2548 | 0.6147 | 1.237 | 0.3065 | 1.173 | 0.2518 | 0.5018 | 1.219 | 0.2855 | 1.18 | 1.158e-07 | 9.459e-06 | 3.83 | 1.938 | 8.008 |  |
| Figf | NA | 0.002427 | 0.03874 | 1.754 | 0.8105 | 3.469 | 0.2503 | 0.61 | 1.255 | 0.3274 | 1.184 | 0.01331 | 0.07009 | 0.6176 | -0.6952 | -2.716 | 5.709e-08 | 5.624e-06 | 4.422 | 2.145 | 8.379 |  |
| Apol9b | apolipoprotein L 9b | 0.01942 | 0.1359 | 0.7076 | -0.4989 | -2.542 | 0.0002293 | 0.01067 | 0.4861 | -1.041 | -4.481 | 9.241e-05 | 0.001623 | 2.067 | 1.048 | 4.873 | 0.01422 | 0.07246 | 1.492 | 0.5775 | 2.686 |  |
| Loxl4 | lysyl oxidase-like 4 | 0.04042 | 0.2083 | 0.7226 | -0.4688 | -2.192 | 0.0003357 | 0.014 | 0.4689 | -1.093 | -4.317 | 0.001483 | 0.01386 | 1.818 | 0.8625 | 3.681 | 0.0003443 | 0.004567 | 2.013 | 1.009 | 4.307 |  |
| Cxx1a | NA | 0.001677 | 0.03137 | 0.6197 | -0.6904 | -3.628 | 0.0001018 | 0.006022 | 0.4705 | -1.088 | -4.831 | 0.0004981 | 0.005979 | 1.821 | 0.8647 | 4.149 | 0.1325 | 0.3305 | 1.254 | 0.327 | 1.569 |  |
| Ube2e2 | ubiquitin-conjugating enzyme E | 0.7039 | 0.879 | 0.9422 | -0.08583 | -0.3856 | 0.003206 | 0.06581 | 1.843 | 0.8818 | 3.348 | 1.074e-05 | 0.0003102 | 0.3738 | -1.42 | -5.823 | 6.085e-06 | 0.0002017 | 0.3578 | -1.483 | -6.082 |  |
| Slc13a2 | solute carrier family 13 (sodi | 0.02405 | 0.1541 | 0.663 | -0.5929 | -2.442 | 0.0002644 | 0.01171 | 0.4147 | -1.27 | -4.42 | 0.000786 | 0.008453 | 2.073 | 1.052 | 3.953 | 0.001481 | 0.01365 | 1.972 | 0.9794 | 3.682 |  |
| Ank2 | ankyrin 2, brain | 6.743e-05 | 0.004358 | 0.5207 | -0.9415 | -5.01 | 0.03533 | 0.2643 | 0.7061 | -0.502 | -2.258 | 5.49e-06 | 0.0001868 | 0.417 | -1.262 | -6.129 | 0.7858 | 0.8989 | 0.9615 | -0.0567 | -0.2754 |  |
| Slc16a7 | solute carrier family 16 (mono | 0.4667 | 0.7387 | 1.155 | 0.2084 | 0.742 | 0.0008638 | 0.02761 | 2.463 | 1.3 | 3.913 | 4.842e-06 | 0.0001682 | 0.2673 | -1.903 | -6.187 | 0.0002404 | 0.003461 | 0.3863 | -1.372 | -4.461 |  |
| Eno1 | enolase 1, alpha non-neuron | 0.007432 | 0.07718 | 0.7339 | -0.4463 | -2.979 | 0.2955 | 0.6507 | 1.141 | 0.1905 | 1.074 | 7.408e-08 | 6.757e-06 | 0.3916 | -1.353 | -8.241 | 0.002866 | 0.02242 | 0.6795 | -0.5575 | -3.397 |  |
| Slc46a1 | solute carrier family 46, memb | 2.006e-05 | 0.002 | 0.475 | -1.074 | -5.543 | 0.006425 | 0.1013 | 0.6166 | -0.6976 | -3.043 | 0.0003914 | 0.00497 | 0.535 | -0.9024 | -4.252 | 0.2983 | 0.5392 | 0.8546 | -0.2267 | -1.068 |  |
| Palld | palladin, cytoskeletal associa | 0.001064 | 0.0238 | 0.6088 | -0.716 | -3.824 | 0.05225 | 0.3163 | 0.7284 | -0.4573 | -2.064 | 0.06504 | 0.2128 | 1.32 | 0.4005 | 1.952 | 1.304e-05 | 0.0003645 | 0.4424 | -1.177 | -5.736 |  |
| Abca8b | ATP-binding cassette, sub-fami | 0.0001329 | 0.006463 | 0.5248 | -0.9303 | -4.716 | 3.933e-05 | 0.002982 | 0.428 | -1.224 | -5.246 | 0.08309 | 0.2511 | 1.314 | 0.3943 | 1.824 | 0.03522 | 0.1383 | 1.403 | 0.4882 | 2.259 |  |
| Gm40892 | NA | 1.286e-05 | 0.001493 | 2.432 | 1.282 | 5.742 | 0.003427 | 0.06839 | 1.837 | 0.877 | 3.319 | 0.02487 | 0.1104 | 1.509 | 0.5934 | 2.426 | 0.01928 | 0.09018 | 1.54 | 0.6227 | 2.545 |  |
| Idh3a | isocitrate dehydrogenase 3 (NA | 0.2328 | 0.534 | 0.8441 | -0.2445 | -1.23 | 0.02237 | 0.2052 | 1.497 | 0.582 | 2.476 | 1.356e-06 | 6.175e-05 | 0.3594 | -1.477 | -6.784 | 0.0001707 | 0.002635 | 0.499 | -1.003 | -4.608 |  |
| Pakap | paralemmin A kinase anchor pro | 0.0001612 | 0.007392 | 0.501 | -0.9972 | -4.632 | 0.2278 | 0.5885 | 0.8028 | -0.3169 | -1.244 | 5.362e-07 | 3.1e-05 | 0.3065 | -1.706 | -7.234 | 0.1708 | 0.3878 | 0.7928 | -0.335 | -1.421 |  |
| Optn | optineurin | 0.0007038 | 0.01805 | 1.542 | 0.6247 | 4.001 | 0.1032 | 0.4278 | 1.244 | 0.3155 | 1.708 | 1.082e-06 | 5.237e-05 | 2.264 | 1.179 | 6.892 | 0.1571 | 0.3685 | 0.8401 | -0.2514 | -1.47 |  |
| Fam78b | family with sequence similarit | 0.0007619 | 0.01895 | 0.5389 | -0.8919 | -3.967 | 0.01189 | 0.1443 | 0.6003 | -0.7362 | -2.767 | 0.03056 | 0.1275 | 1.488 | 0.5733 | 2.328 | 0.0004178 | 0.00527 | 0.4862 | -1.04 | -4.224 |  |
| Gm10639 | predicted gene 10639 | 0.1492 | 0.4242 | 0.8376 | -0.2556 | -1.5 | 0.0005275 | 0.01935 | 0.562 | -0.8314 | -4.124 | 1.338e-05 | 0.0003653 | 2.097 | 1.068 | 5.725 | 0.002107 | 0.01787 | 1.579 | 0.6588 | 3.53 |  |
| Rasd1 | RAS, dexamethasone-induced 1 | 0.006272 | 0.07039 | 0.4449 | -1.169 | -3.054 | 0.2833 | 0.6385 | 1.413 | 0.4992 | 1.103 | 1.886e-08 | 2.511e-06 | 0.07358 | -3.765 | -8.981 | 0.007834 | 0.04703 | 0.4238 | -1.239 | -2.955 |  |
| Ccl2 | chemokine (C-C motif) ligand 2 | 0.0001949 | 0.008245 | 0.484 | -1.047 | -4.551 | 0.0287 | 0.2351 | 0.6409 | -0.6418 | -2.358 | 1.336e-05 | 0.0003651 | 0.3678 | -1.443 | -5.725 | 0.3776 | 0.6117 | 1.171 | 0.2274 | 0.9024 |  |
| Slco5a1 | solute carrier organic anion t | 0.001147 | 0.02481 | 0.6939 | -0.5272 | -3.792 | 0.382 | 0.7135 | 1.107 | 0.1471 | 0.8939 | 0.06345 | 0.2096 | 0.8126 | -0.2993 | -1.965 | 3.867e-10 | 1.13e-07 | 0.3028 | -1.723 | -11.32 |  |
| Tbk1 | TANK-binding kinase 1 | 0.000155 | 0.007207 | 1.534 | 0.6171 | 4.649 | 0.3219 | 0.6708 | 1.117 | 0.1595 | 1.016 | 0.1662 | 0.3899 | 1.156 | 0.2089 | 1.437 | 1.166e-07 | 9.459e-06 | 2.241 | 1.164 | 8.004 |  |
| Lrtm1 | leucine-rich repeats and trans | 0.01871 | 0.133 | 0.5415 | -0.8849 | -2.559 | 0.2305 | 0.5917 | 1.42 | 0.506 | 1.237 | 1.305e-06 | 6.069e-05 | 0.1676 | -2.577 | -6.802 | 0.0004274 | 0.005363 | 0.3307 | -1.596 | -4.214 |  |
| Lrp4 | low density lipoprotein recept | 0.8571 | 0.9466 | 1.018 | 0.02609 | 0.1824 | 0.0002407 | 0.01103 | 1.687 | 0.7548 | 4.46 | 0.003392 | 0.0256 | 0.697 | -0.5207 | -3.324 | 1.137e-09 | 2.66e-07 | 0.3153 | -1.665 | -10.63 |  |
| Vtcn1 | V-set domain containing T cell | 0.2341 | 0.5357 | 0.8352 | -0.2599 | -1.227 | 0.0006629 | 0.02273 | 0.4969 | -1.009 | -4.026 | 1.166e-05 | 0.000331 | 2.536 | 1.343 | 5.787 | 0.0008903 | 0.009325 | 1.872 | 0.9049 | 3.9 |  |
| Insig2 | insulin induced gene 2 | 0.07625 | 0.2961 | 1.24 | 0.3108 | 1.87 | 0.0001392 | 0.007429 | 1.897 | 0.9236 | 4.696 | 0.925 | 0.9691 | 1.012 | 0.01736 | 0.09534 | 2.329e-09 | 4.681e-07 | 0.2763 | -1.856 | -10.19 |  |
| Palm2 | NA | 0.0002391 | 0.009419 | 0.6111 | -0.7106 | -4.463 | 0.969 | 0.9898 | 1.005 | 0.007409 | 0.03933 | 2.456e-08 | 3.022e-06 | 0.3437 | -1.541 | -8.835 | 0.002184 | 0.01837 | 0.6539 | -0.613 | -3.514 |  |
| Zfp9 | zinc finger protein 9 | 0.4272 | 0.7108 | 1.143 | 0.1932 | 0.8105 | 0.0007227 | 0.02408 | 2.182 | 1.125 | 3.989 | 4.412e-07 | 2.671e-05 | 0.2653 | -1.915 | -7.33 | 0.002997 | 0.02315 | 0.5426 | -0.8821 | -3.377 |  |
| Trim24 | tripartite motif-containing 24 | 0.08295 | 0.308 | 1.319 | 0.3995 | 1.825 | 0.0004444 | 0.01704 | 2.124 | 1.087 | 4.197 | 0.001721 | 0.01546 | 0.5482 | -0.8672 | -3.617 | 7.138e-05 | 0.001375 | 0.4368 | -1.195 | -4.985 |  |
| Gm4184 | NA | 0.03394 | 0.1892 | 0.7084 | -0.4974 | -2.277 | 0.0003173 | 0.01333 | 0.4594 | -1.122 | -4.342 | 0.0003232 | 0.004265 | 2.052 | 1.037 | 4.334 | 0.002263 | 0.01882 | 1.787 | 0.8374 | 3.499 |  |
| Utp11l | NA | 0.0005374 | 0.01539 | 1.602 | 0.6795 | 4.116 | 0.6873 | 0.8829 | 0.9462 | -0.07977 | -0.4084 | 0.004043 | 0.02925 | 1.502 | 0.5872 | 3.247 | 9.802e-09 | 1.424e-06 | 3.228 | 1.691 | 9.349 |  |
| Tgm1 | transglutaminase 1, K polypept | 0.5826 | 0.8145 | 1.102 | 0.1405 | 0.5587 | 0.005033 | 0.08748 | 0.5222 | -0.9374 | -3.151 | 3.648e-05 | 0.0007886 | 2.739 | 1.454 | 5.279 | 2.68e-06 | 0.0001041 | 3.434 | 1.78 | 6.462 |  |
| Rhbdl3 | rhomboid like 3 | 0.0002723 | 0.01013 | 0.5662 | -0.8205 | -4.407 | 0.4571 | 0.7622 | 0.8907 | -0.1671 | -0.7584 | 7.199e-06 | 0.0002289 | 0.4279 | -1.225 | -6.005 | 0.001761 | 0.01562 | 0.6005 | -0.7357 | -3.607 |  |
| C730034F03Rik | RIKEN cDNA C730034F03 gene | 0.002126 | 0.03624 | 1.599 | 0.6771 | 3.526 | 0.0001295 | 0.007127 | 2.105 | 1.074 | 4.727 | 0.02231 | 0.102 | 0.6968 | -0.5211 | -2.477 | 0.004674 | 0.03223 | 0.6286 | -0.6697 | -3.183 |  |
| Cdh1 | cadherin 1 | 0.0578 | 0.2551 | 0.7159 | -0.4822 | -2.013 | 0.0004019 | 0.01584 | 0.4347 | -1.202 | -4.24 | 0.002701 | 0.02166 | 1.864 | 0.8982 | 3.423 | 0.0001079 | 0.00186 | 2.397 | 1.261 | 4.806 |  |
| Slc45a4 | solute carrier family 45, memb | 0.0002248 | 0.009062 | 0.6051 | -0.7248 | -4.49 | 0.4974 | 0.7852 | 0.9126 | -0.132 | -0.6911 | 0.003996 | 0.02898 | 0.6712 | -0.5751 | -3.252 | 1.302e-06 | 6.034e-05 | 0.4343 | -1.203 | -6.804 |  |
| Cxx1b | NA | 0.001889 | 0.03385 | 0.6206 | -0.6882 | -3.577 | 0.0001234 | 0.006873 | 0.4728 | -1.081 | -4.748 | 0.0005672 | 0.006587 | 1.818 | 0.8627 | 4.093 | 0.1504 | 0.3589 | 1.244 | 0.3152 | 1.496 |  |
| Angptl1 | angiopoietin-like 1 | 9.25e-05 | 0.005241 | 0.4178 | -1.259 | -4.872 | 0.05266 | 0.3172 | 0.6462 | -0.63 | -2.06 | 0.004921 | 0.03367 | 0.5378 | -0.8949 | -3.161 | 0.00222 | 0.0186 | 0.5024 | -0.993 | -3.507 |  |
| Lbr | lamin B receptor | 0.7111 | 0.882 | 1.054 | 0.0756 | 0.3758 | 0.003687 | 0.07173 | 0.5814 | -0.7825 | -3.287 | 7.727e-06 | 0.0002421 | 2.49 | 1.316 | 5.973 | 1.387e-05 | 0.0003813 | 2.392 | 1.258 | 5.708 |  |
| Ints2 | integrator complex subunit 2 | 0.0005232 | 0.01508 | 0.6533 | -0.6141 | -4.128 | 0.9423 | 0.9813 | 1.009 | 0.0129 | 0.07325 | 3.54e-06 | 0.0001292 | 0.489 | -1.032 | -6.332 | 4.271e-05 | 0.0009171 | 0.5552 | -0.849 | -5.209 |  |
| LOC102643131 | NA | 0.06325 | 0.2673 | 1.214 | 0.2799 | 1.967 | 0.0003694 | 0.01494 | 1.647 | 0.7201 | 4.277 | 7.579e-07 | 4.003e-05 | 0.4661 | -1.101 | -7.064 | 0.1748 | 0.3934 | 0.859 | -0.2193 | -1.407 |  |
| Rai14 | retinoic acid induced 14 | 0.07851 | 0.2999 | 1.251 | 0.3236 | 1.854 | 0.0833 | 0.3924 | 0.7703 | -0.3764 | -1.823 | 2.902e-06 | 0.0001113 | 2.343 | 1.228 | 6.424 | 0.0001901 | 0.002875 | 1.83 | 0.872 | 4.561 |  |
| Klhdc3 | kelch domain containing 3 | 0.4322 | 0.7134 | 0.9132 | -0.1311 | -0.8016 | 0.01038 | 0.1338 | 1.461 | 0.5473 | 2.829 | 1.071e-05 | 0.0003097 | 0.4852 | -1.043 | -5.825 | 2.032e-05 | 0.0005124 | 0.5028 | -0.9919 | -5.538 |  |
| Acad11 | acyl-Coenzyme A dehydrogenase | 0.001294 | 0.02689 | 1.486 | 0.5718 | 3.74 | 0.07801 | 0.3827 | 1.262 | 0.3361 | 1.858 | 5.535e-06 | 0.0001871 | 2.036 | 1.026 | 6.125 | 0.0715 | 0.2247 | 0.8017 | -0.3188 | -1.903 |  |
| Bcap29 | B cell receptor associated pro | 0.03465 | 0.1915 | 1.362 | 0.4456 | 2.267 | 0.0003655 | 0.01481 | 1.994 | 0.9957 | 4.281 | 0.0197 | 0.0933 | 0.685 | -0.5459 | -2.535 | 5.135e-05 | 0.001059 | 0.4651 | -1.104 | -5.129 |  |
| Sec13 | SEC13 homolog, nuclear pore an | 0.01504 | 0.1168 | 0.8048 | -0.3133 | -2.66 | 0.2201 | 0.5814 | 1.13 | 0.1764 | 1.266 | 7.35e-08 | 6.728e-06 | 0.4784 | -1.064 | -8.246 | 0.005133 | 0.03444 | 0.755 | -0.4054 | -3.142 |  |
| Chka | choline kinase alpha | 0.003853 | 0.05104 | 1.552 | 0.6341 | 3.268 | 0.4476 | 0.7576 | 0.884 | -0.1779 | -0.7746 | 0.0002798 | 0.00382 | 1.911 | 0.9343 | 4.396 | 7.58e-07 | 3.908e-05 | 2.832 | 1.502 | 7.064 |  |
| Nfyb | nuclear transcription factor-Y | 0.06996 | 0.2821 | 0.8532 | -0.2291 | -1.915 | 0.0002593 | 0.01154 | 0.6476 | -0.6268 | -4.428 | 2.774e-08 | 3.234e-06 | 2.218 | 1.149 | 8.769 | 0.74 | 0.8737 | 1.031 | 0.0441 | 0.3366 |  |
| Cyr61 | NA | 0.5143 | 0.7706 | 1.089 | 0.1232 | 0.664 | 0.0004287 | 0.01666 | 0.5266 | -0.9252 | -4.213 | 0.04116 | 0.1564 | 1.36 | 0.4438 | 2.183 | 2.241e-11 | 1.428e-08 | 6.505 | 2.701 | 13.29 |  |
| Cyp2c39 | cytochrome P450, family 2, sub | 0.1034 | 0.3477 | 1.392 | 0.477 | 1.707 | 0.0005397 | 0.01964 | 2.568 | 1.361 | 4.114 | 0.0002158 | 0.003133 | 0.3842 | -1.38 | -4.507 | 0.000495 | 0.005978 | 0.4144 | -1.271 | -4.151 |  |
| Cirbp | cold inducible RNA binding pro | 9.809e-05 | 0.005449 | 1.982 | 0.9867 | 4.847 | 4.064e-05 | 0.00303 | 2.395 | 1.26 | 5.231 | 0.1281 | 0.3319 | 0.7824 | -0.354 | -1.587 | 0.04966 | 0.1754 | 0.724 | -0.466 | -2.09 |  |
| Slc35f5 | solute carrier family 35, memb | 0.001865 | 0.03353 | 1.462 | 0.5477 | 3.583 | 0.0802 | 0.3876 | 1.26 | 0.3334 | 1.843 | 0.04026 | 0.1545 | 0.7752 | -0.3674 | -2.194 | 6.781e-06 | 0.0002191 | 2.014 | 1.01 | 6.032 |  |
| Dclre1b | DNA cross-link repair 1B | 1.781e-07 | 6.978e-05 | 2.309 | 1.207 | 7.786 | 1.039e-05 | 0.001119 | 2.101 | 1.071 | 5.838 | 0.2408 | 0.4895 | 1.153 | 0.2053 | 1.209 | 0.2456 | 0.4801 | 1.151 | 0.2031 | 1.196 |  |
| Deptor | DEP domain containing MTOR-int | 0.0001884 | 0.008099 | 0.5748 | -0.799 | -4.565 | 0.1581 | 0.5115 | 0.8102 | -0.3036 | -1.466 | 1.089e-05 | 0.0003141 | 0.4616 | -1.115 | -5.817 | 0.06738 | 0.2161 | 0.7734 | -0.3708 | -1.934 |  |
| Tmem150a | transmembrane protein 150A | 0.0002101 | 0.008717 | 0.5556 | -0.8479 | -4.519 | 6.775e-05 | 0.004492 | 0.4627 | -1.112 | -5.008 | 0.4522 | 0.6925 | 1.115 | 0.1576 | 0.7667 | 0.005837 | 0.03788 | 1.552 | 0.6343 | 3.086 |  |
| Plcd4 | phospholipase C, delta 4 | 0.00014 | 0.006708 | 0.3758 | -1.412 | -4.693 | 0.08081 | 0.3889 | 0.6352 | -0.6548 | -1.839 | 0.7446 | 0.8819 | 0.9273 | -0.1089 | -0.3303 | 2.161e-06 | 8.909e-05 | 0.2233 | -2.163 | -6.563 |  |
| Gm16412 | predicted pseudogene 16412 | 0.04631 | 0.2257 | 0.7104 | -0.4933 | -2.124 | 0.0003992 | 0.0158 | 0.4457 | -1.166 | -4.243 | 0.0002595 | 0.003595 | 2.183 | 1.126 | 4.428 | 0.002238 | 0.01868 | 1.855 | 0.8913 | 3.504 |  |
| Psmd11 | proteasome (prosome, macropain | 0.02502 | 0.1583 | 1.239 | 0.3095 | 2.423 | 0.1243 | 0.4633 | 0.8453 | -0.2425 | -1.604 | 0.008096 | 0.04863 | 1.33 | 0.4115 | 2.94 | 2.209e-08 | 2.619e-06 | 2.37 | 1.245 | 8.893 |  |
| Coa6 | cytochrome c oxidase assembly | 0.1411 | 0.4121 | 1.39 | 0.475 | 1.532 | 0.0006385 | 0.02219 | 2.795 | 1.483 | 4.042 | 0.0001036 | 0.001772 | 0.3213 | -1.638 | -4.823 | 0.0005864 | 0.006822 | 0.3829 | -1.385 | -4.079 |  |
| Lox | lysyl oxidase | 0.0002982 | 0.0107 | 2.306 | 1.205 | 4.368 | 0.418 | 0.7391 | 1.206 | 0.27 | 0.827 | 2.162e-05 | 0.0005281 | 3.172 | 1.665 | 5.51 | 0.001197 | 0.01165 | 2.204 | 1.14 | 3.773 |  |
| Mapk1ip1 | mitogen-activated protein kina | 0.0003786 | 0.01239 | 1.67 | 0.7399 | 4.266 | 0.7515 | 0.9089 | 1.047 | 0.06588 | 0.321 | 5.178e-05 | 0.001028 | 1.964 | 0.9738 | 5.125 | 2.126e-05 | 0.0005295 | 2.068 | 1.048 | 5.517 |  |
| Gemin5 | gem nuclear organelle associat | 0.006869 | 0.07436 | 1.39 | 0.4747 | 3.014 | 0.3863 | 0.7165 | 0.8919 | -0.1651 | -0.8858 | 0.0001743 | 0.002659 | 1.733 | 0.7936 | 4.599 | 2.344e-06 | 9.482e-05 | 2.182 | 1.126 | 6.524 |  |
| Gm22645 | predicted gene, 22645 | 0.0009781 | 0.02246 | 0.601 | -0.7345 | -3.86 | 0.0001059 | 0.006165 | 0.4718 | -1.084 | -4.814 | 0.009104 | 0.05293 | 1.518 | 0.602 | 2.888 | 0.04489 | 0.1635 | 1.362 | 0.4461 | 2.14 |  |
| 1700055D18Rik | RIKEN cDNA 1700055D18 gene | 0.0009046 | 0.02123 | 1.76 | 0.8157 | 3.893 | 0.2197 | 0.581 | 1.243 | 0.3142 | 1.267 | 1.293e-07 | 1.021e-05 | 3.543 | 1.825 | 7.951 | 0.1784 | 0.3982 | 0.801 | -0.3201 | -1.395 |  |
| Khdrbs3 | KH domain containing, RNA bind | 0.215 | 0.5136 | 1.168 | 0.2237 | 1.28 | 0.0007313 | 0.02424 | 1.77 | 0.8236 | 3.984 | 0.0009264 | 0.009582 | 0.5975 | -0.7431 | -3.883 | 2.11e-05 | 0.000526 | 0.4808 | -1.057 | -5.521 |  |
| St8sia5 | ST8 alpha-N-acetyl-neuraminide | 2.795e-05 | 0.002531 | 0.6341 | -0.6573 | -5.396 | 0.7056 | 0.8908 | 0.9624 | -0.05524 | -0.3833 | 0.1802 | 0.4101 | 0.8795 | -0.1853 | -1.389 | 1.112e-10 | 4.087e-08 | 0.3252 | -1.621 | -12.15 |  |
| Noc4l | NOC4 like | 0.2638 | 0.5696 | 0.8709 | -0.1995 | -1.15 | 0.0007796 | 0.02555 | 0.5694 | -0.8124 | -3.957 | 0.001334 | 0.01284 | 1.634 | 0.7084 | 3.727 | 8.187e-06 | 0.0002516 | 2.189 | 1.13 | 5.947 |  |
| Rsc1a1 | regulatory solute carrier prot | 0.0003697 | 0.01219 | 1.904 | 0.9292 | 4.276 | 0.8861 | 0.9614 | 0.9745 | -0.03731 | -0.1451 | 1.823e-08 | 2.455e-06 | 4.415 | 2.142 | 9 | 0.004699 | 0.03232 | 1.69 | 0.7572 | 3.181 |  |
| Enpep | glutamyl aminopeptidase | 1.806e-05 | 0.00187 | 0.4016 | -1.316 | -5.59 | 0.001279 | 0.03663 | 0.4853 | -1.043 | -3.745 | 0.001574 | 0.01449 | 0.5203 | -0.9427 | -3.656 | 0.636 | 0.8081 | 1.09 | 0.1239 | 0.4806 |  |
| Mt2 | metallothionein 2 | 0.0002894 | 0.0105 | 4.612 | 2.205 | 4.381 | 0.0003033 | 0.01294 | 6.053 | 2.598 | 4.361 | 0.002992 | 0.0234 | 0.2749 | -1.863 | -3.378 | 0.2276 | 0.4591 | 1.609 | 0.6864 | 1.245 |  |
| Gdap1 | ganglioside-induced differenti | 2.978e-05 | 0.002642 | 0.4815 | -1.054 | -5.368 | 0.3851 | 0.7161 | 0.8667 | -0.2064 | -0.888 | 0.9321 | 0.9723 | 0.9872 | -0.01856 | -0.08624 | 2.038e-10 | 6.552e-08 | 0.1737 | -2.525 | -11.74 |  |
| Slc25a22 | solute carrier family 25 (mito | 2.685e-05 | 0.002458 | 2.043 | 1.031 | 5.414 | 0.001566 | 0.04169 | 1.77 | 0.824 | 3.658 | 0.6217 | 0.8112 | 0.9301 | -0.1045 | -0.5011 | 0.002392 | 0.01961 | 1.653 | 0.7248 | 3.475 |  |
| Ccnd3 | cyclin D3 | 0.0008078 | 0.0197 | 1.879 | 0.9101 | 3.942 | 0.0001083 | 0.006288 | 2.484 | 1.312 | 4.804 | 0.01128 | 0.0622 | 0.613 | -0.706 | -2.791 | 0.06149 | 0.2032 | 0.7066 | -0.5011 | -1.981 |  |
| Morc4 | microrchidia 4 | 0.002571 | 0.04003 | 0.5887 | -0.7644 | -3.444 | 0.5318 | 0.8033 | 1.123 | 0.1671 | 0.6363 | 9.913e-08 | 8.499e-06 | 0.2559 | -1.967 | -8.089 | 0.002813 | 0.02211 | 0.5634 | -0.8278 | -3.405 |  |
| Mir3064 | microRNA 3064 | 0.0009157 | 0.02137 | 1.628 | 0.703 | 3.888 | 0.0001171 | 0.006634 | 2.029 | 1.021 | 4.77 | 0.01365 | 0.07152 | 0.6898 | -0.5357 | -2.705 | 0.04811 | 0.1718 | 0.749 | -0.417 | -2.105 |  |
| Fam134b | NA | 1.082e-06 | 0.0002677 | 2.609 | 1.384 | 6.892 | 7.251e-05 | 0.004698 | 2.27 | 1.182 | 4.978 | 0.3523 | 0.6063 | 1.156 | 0.2094 | 0.9523 | 0.08839 | 0.2568 | 1.314 | 0.394 | 1.791 |  |
| Mir181a-2 | microRNA 181a-2 | 0.0003888 | 0.0126 | 0.5355 | -0.901 | -4.255 | 0.1474 | 0.4976 | 0.7697 | -0.3777 | -1.507 | 0.5303 | 0.7526 | 1.108 | 0.1481 | 0.6386 | 3.784e-07 | 2.264e-05 | 0.3039 | -1.718 | -7.406 |  |
| Zrsr1 | zinc finger (CCCH type), RNA b | 0.0002083 | 0.008662 | 1.967 | 0.9758 | 4.522 | 0.1334 | 0.4753 | 1.319 | 0.3994 | 1.564 | 0.02155 | 0.09935 | 1.505 | 0.5894 | 2.493 | 0.000104 | 0.001806 | 2.203 | 1.14 | 4.822 |  |
| Arhgap26 | novel protein | 0.000254 | 0.009734 | 1.756 | 0.8122 | 4.437 | 0.3626 | 0.7004 | 1.15 | 0.2018 | 0.9318 | 0.04531 | 0.1668 | 1.346 | 0.4282 | 2.135 | 8.715e-07 | 4.361e-05 | 2.644 | 1.403 | 6.997 |  |
| Car1 | carbonic anhydrase 1 | 0.06228 | 0.2648 | 0.6292 | -0.6685 | -1.975 | 0.0004872 | 0.01834 | 0.3152 | -1.666 | -4.158 | 8.968e-06 | 0.0002689 | 4.563 | 2.19 | 5.905 | 0.04307 | 0.159 | 1.743 | 0.8012 | 2.16 |  |
| Tsc22d3 | TSC22 domain family, member 3 | 5.487e-06 | 0.0008574 | 2.493 | 1.318 | 6.129 | 8.694e-06 | 0.0009984 | 2.84 | 1.506 | 5.919 | 0.5731 | 0.7814 | 0.9107 | -0.1349 | -0.5729 | 0.08354 | 0.2481 | 0.7428 | -0.429 | -1.822 |  |
| Gm6762 | predicted pseudogene 6762 | 0.005542 | 0.06518 | 0.6534 | -0.614 | -3.109 | 0.000203 | 0.009678 | 0.4798 | -1.059 | -4.533 | 0.002085 | 0.01785 | 1.699 | 0.7648 | 3.534 | 0.01565 | 0.07754 | 1.486 | 0.5716 | 2.642 |  |
| Lfng | LFNG O-fucosylpeptide 3-beta-N | 0.001788 | 0.03259 | 1.875 | 0.907 | 3.601 | 0.001037 | 0.03182 | 2.208 | 1.143 | 3.835 | 0.0004334 | 0.005367 | 0.4471 | -1.161 | -4.208 | 0.116 | 0.3045 | 1.369 | 0.4534 | 1.643 |  |
| LOC105247125 | NA | 8.346e-05 | 0.00489 | 4.03 | 2.011 | 4.917 | 0.03595 | 0.2661 | 2.126 | 1.088 | 2.249 | 0.00685 | 0.04306 | 2.55 | 1.351 | 3.015 | 0.004894 | 0.03332 | 2.67 | 1.417 | 3.163 |  |
| Bnip3 | BCL2/adenovirus E1B interactin | 1.746e-06 | 0.0003852 | 2.171 | 1.118 | 6.664 | 5.039e-06 | 0.0006822 | 2.337 | 1.225 | 6.169 | 0.05924 | 0.2003 | 0.775 | -0.3677 | -2 | 0.7967 | 0.9045 | 1.034 | 0.04798 | 0.261 |  |
| Adamts20 | a disintegrin-like and metallo | 0.0001647 | 0.007477 | 0.5894 | -0.7626 | -4.623 | 0.122 | 0.4599 | 0.8037 | -0.3152 | -1.615 | 0.8807 | 0.9502 | 0.9811 | -0.02746 | -0.152 | 4.927e-07 | 2.761e-05 | 0.402 | -1.315 | -7.276 |  |
| Sema7a | sema domain, immunoglobulin do | 0.0008691 | 0.02069 | 1.446 | 0.5324 | 3.91 | 0.8706 | 0.9564 | 0.9817 | -0.02658 | -0.165 | 1.38e-06 | 6.218e-05 | 2.015 | 1.011 | 6.776 | 0.0002363 | 0.003415 | 1.587 | 0.6664 | 4.468 |  |
| P2ry1 | purinergic receptor P2Y, G-pro | 0.02146 | 0.1439 | 0.7242 | -0.4656 | -2.495 | 0.2193 | 0.5806 | 1.214 | 0.28 | 1.268 | 0.0005788 | 0.006692 | 0.5607 | -0.8348 | -4.084 | 1.159e-06 | 5.564e-05 | 0.3784 | -1.402 | -6.859 |  |
| Tmem25 | transmembrane protein 25 | 0.01519 | 0.1176 | 0.666 | -0.5863 | -2.656 | 0.1965 | 0.5594 | 1.274 | 0.3491 | 1.336 | 0.01433 | 0.07407 | 0.6378 | -0.6487 | -2.682 | 2.104e-08 | 2.541e-06 | 0.2241 | -2.157 | -8.92 |  |
| Pikfyve | phosphoinositide kinase, FYVE | 0.1545 | 0.4318 | 1.151 | 0.2026 | 1.48 | 0.04227 | 0.2873 | 0.7838 | -0.3515 | -2.17 | 0.0003715 | 0.004764 | 1.56 | 0.6411 | 4.274 | 1.287e-06 | 6e-05 | 2.03 | 1.021 | 6.809 |  |
| Sorbs3 | sorbin and SH3 domain containi | 2.543e-06 | 0.0005032 | 2.58 | 1.367 | 6.486 | 3.665e-05 | 0.002865 | 2.49 | 1.316 | 5.277 | 0.3444 | 0.5992 | 0.8564 | -0.2236 | -0.9684 | 0.118 | 0.3076 | 1.299 | 0.3773 | 1.633 |  |
| Gclm | glutamate-cysteine ligase, mod | 0.1507 | 0.4264 | 1.164 | 0.2192 | 1.494 | 0.007469 | 0.1117 | 0.699 | -0.5167 | -2.976 | 0.1279 | 0.3314 | 1.194 | 0.2553 | 1.588 | 1.111e-10 | 4.087e-08 | 3.87 | 1.952 | 12.15 |  |
| Abi3 | ABI gene family, member 3 | 0.0002026 | 0.008473 | 2.135 | 1.094 | 4.534 | 0.156 | 0.5094 | 1.339 | 0.4208 | 1.474 | 8.064e-05 | 0.001451 | 2.468 | 1.304 | 4.932 | 0.01352 | 0.06987 | 1.643 | 0.7159 | 2.709 |  |
| Gm10680 | predicted gene 10680 | 0.7045 | 0.8791 | 0.9357 | -0.09587 | -0.3847 | 0.004471 | 0.08159 | 1.924 | 0.9443 | 3.203 | 2.941e-05 | 0.0006713 | 0.3618 | -1.467 | -5.374 | 6.403e-06 | 0.0002109 | 0.3178 | -1.654 | -6.059 |  |
| Epg5 | ectopic P-granules autophagy p | 0.8231 | 0.9333 | 1.019 | 0.02652 | 0.2265 | 0.001723 | 0.04406 | 0.7066 | -0.501 | -3.617 | 0.0009886 | 0.01005 | 1.409 | 0.4945 | 3.855 | 4.691e-08 | 4.827e-06 | 2.126 | 1.088 | 8.484 |  |
| Gm42368 | NA | 0.06937 | 0.2812 | 1.695 | 0.761 | 1.919 | 0.0005114 | 0.01901 | 3.84 | 1.941 | 4.137 | 3.486e-06 | 0.0001281 | 0.1483 | -2.754 | -6.339 | 0.0851 | 0.2512 | 0.5796 | -0.787 | -1.812 |  |
| 3010026O09Rik | NA | 0.003607 | 0.04894 | 0.5161 | -0.9544 | -3.297 | 0.000244 | 0.01109 | 0.3473 | -1.526 | -4.454 | 0.2848 | 0.5394 | 1.273 | 0.3486 | 1.099 | 0.0003452 | 0.004573 | 2.576 | 1.365 | 4.306 |  |
| Synpo2l | synaptopodin 2-like | 0.0001568 | 0.00723 | 0.4947 | -1.015 | -4.644 | 0.08326 | 0.3924 | 0.7212 | -0.4716 | -1.823 | 0.9496 | 0.9805 | 0.9894 | -0.01534 | -0.06407 | 1.466e-06 | 6.605e-05 | 0.3263 | -1.616 | -6.747 |  |
| Fgf1 | fibroblast growth factor 1 | 0.02828 | 0.1699 | 1.389 | 0.4742 | 2.365 | 0.000373 | 0.01502 | 2.019 | 1.014 | 4.272 | 2.027e-06 | 8.357e-05 | 0.3665 | -1.448 | -6.593 | 0.4474 | 0.6709 | 0.8887 | -0.1703 | -0.7751 |  |
| Dnttip2 | deoxynucleotidyltransferase, t | 0.000295 | 0.01063 | 1.548 | 0.6301 | 4.373 | 0.4946 | 0.7838 | 1.086 | 0.1186 | 0.6957 | 0.005695 | 0.0376 | 1.403 | 0.4888 | 3.097 | 1.896e-06 | 8.14e-05 | 2.064 | 1.046 | 6.625 |  |
| Mpc1 | mitochondrial pyruvate carrier | 0.5309 | 0.7823 | 0.909 | -0.1376 | -0.6377 | 0.008763 | 0.1215 | 1.672 | 0.7418 | 2.905 | 4.604e-06 | 0.0001614 | 0.3614 | -1.468 | -6.21 | 7.742e-05 | 0.00145 | 0.4444 | -1.17 | -4.95 |  |
| Slc4a1 | solute carrier family 4 (anion | 0.0008673 | 0.02069 | 0.6148 | -0.7018 | -3.911 | 0.0001193 | 0.006732 | 0.4962 | -1.011 | -4.762 | 0.003298 | 0.02509 | 1.575 | 0.6557 | 3.336 | 0.1816 | 0.4022 | 1.207 | 0.272 | 1.384 |  |
| Emp1 | epithelial membrane protein 1 | 0.0001481 | 0.006954 | 0.4085 | -1.292 | -4.669 | 0.08888 | 0.4028 | 0.6664 | -0.5854 | -1.788 | 0.0001598 | 0.002481 | 0.3776 | -1.405 | -4.636 | 0.02887 | 0.1205 | 0.6098 | -0.7137 | -2.355 |  |
| Stom | stomatin | 0.02135 | 0.1433 | 0.7899 | -0.3402 | -2.498 | 0.0003744 | 0.01503 | 0.6206 | -0.6883 | -4.271 | 0.9503 | 0.9808 | 0.9935 | -0.00942 | -0.06313 | 7.619e-07 | 3.912e-05 | 2.076 | 1.054 | 7.062 |  |
| Etl4 | enhancer trap locus 4 | 0.07227 | 0.2871 | 1.205 | 0.2685 | 1.898 | 0.06855 | 0.3593 | 0.7998 | -0.3224 | -1.925 | 3.551e-08 | 3.822e-06 | 2.529 | 1.338 | 8.634 | 0.01102 | 0.06047 | 1.351 | 0.4343 | 2.802 |  |
| Dmpk | dystrophia myotonica-protein k | 0.000279 | 0.01026 | 0.5092 | -0.9736 | -4.397 | 0.4174 | 0.7386 | 0.8604 | -0.217 | -0.828 | 3.267e-07 | 2.122e-05 | 0.2843 | -1.814 | -7.48 | 0.07502 | 0.2317 | 0.7292 | -0.4556 | -1.878 |  |
| Tmem120a | transmembrane protein 120A | 0.3061 | 0.6089 | 0.8147 | -0.2956 | -1.05 | 0.02079 | 0.1977 | 1.785 | 0.8358 | 2.51 | 2.568e-05 | 0.0006054 | 0.3131 | -1.675 | -5.434 | 1.862e-05 | 0.00048 | 0.3037 | -1.719 | -5.577 |  |
| Plekhm1 | pleckstrin homology domain con | 0.82 | 0.9316 | 1.023 | 0.03265 | 0.2306 | 0.002118 | 0.0505 | 0.6639 | -0.5909 | -3.528 | 0.0005031 | 0.006025 | 1.561 | 0.6427 | 4.144 | 1.377e-07 | 1.058e-05 | 2.342 | 1.228 | 7.918 |  |
| Arhgef26 | Rho guanine nucleotide exchang | 0.9469 | 0.9813 | 0.99 | -0.01447 | -0.06747 | 0.002199 | 0.05157 | 1.855 | 0.8913 | 3.511 | 8.489e-06 | 0.0002597 | 0.3806 | -1.394 | -5.93 | 1.641e-05 | 0.0004343 | 0.3995 | -1.324 | -5.633 |  |
| Gm12060 | NA | 0.0001564 | 0.00723 | 1.724 | 0.7857 | 4.646 | 0.07915 | 0.3847 | 1.293 | 0.3702 | 1.85 | 2.189e-05 | 0.0005328 | 2.028 | 1.02 | 5.504 | 0.2356 | 0.4685 | 1.17 | 0.2266 | 1.223 |  |
| Ppip5k2 | diphosphoinositol pentakisphos | 0.1211 | 0.3797 | 1.275 | 0.3508 | 1.619 | 0.0006685 | 0.02284 | 2.044 | 1.031 | 4.023 | 2.887e-05 | 0.0006606 | 0.4126 | -1.277 | -5.382 | 0.004317 | 0.03046 | 0.5889 | -0.7638 | -3.218 |  |
| Irf4 | interferon regulatory factor 4 | 0.0001005 | 0.005499 | 4.419 | 2.144 | 4.837 | 0.8746 | 0.9576 | 1.06 | 0.08383 | 0.1599 | 1.341e-09 | 3.253e-07 | 34.57 | 5.112 | 10.53 | 0.03975 | 0.1504 | 2.097 | 1.068 | 2.2 |  |
| Gm9824 | predicted pseudogene 9824 | 2.293e-05 | 0.002207 | 0.5119 | -0.966 | -5.484 | 2.344e-05 | 0.002037 | 0.4535 | -1.141 | -5.474 | 0.3515 | 0.6056 | 1.136 | 0.1841 | 0.954 | 0.09264 | 0.2645 | 1.266 | 0.3408 | 1.766 |  |
| Kcnj2 | potassium inwardly-rectifying | 0.008822 | 0.08609 | 0.7069 | -0.5004 | -2.902 | 0.0882 | 0.4013 | 0.7761 | -0.3658 | -1.793 | 1.922e-05 | 0.000479 | 0.4827 | -1.051 | -5.563 | 0.002694 | 0.02141 | 1.566 | 0.6467 | 3.424 |  |
| Glyctk | glycerate kinase | 0.9934 | 0.9978 | 1.001 | 0.001337 | 0.008369 | 0.001805 | 0.04526 | 1.602 | 0.6797 | 3.597 | 1.229e-06 | 5.789e-05 | 0.4367 | -1.195 | -6.831 | 0.0001093 | 0.001881 | 0.5587 | -0.8399 | -4.8 |  |
| Krt222 | keratin 222 | 0.001896 | 0.0339 | 0.716 | -0.482 | -3.575 | 0.6638 | 0.872 | 1.05 | 0.07037 | 0.4411 | 0.0002668 | 0.003672 | 0.6363 | -0.6522 | -4.416 | 1.298e-06 | 6.033e-05 | 0.4983 | -1.005 | -6.805 |  |
| Mknk2 | MAP kinase-interacting serine/ | 0.08326 | 0.3087 | 1.222 | 0.2894 | 1.823 | 0.0003363 | 0.014 | 1.754 | 0.8107 | 4.317 | 2.709e-08 | 3.212e-06 | 0.347 | -1.527 | -8.781 | 0.8333 | 0.9234 | 0.9746 | -0.03708 | -0.2133 |  |
| Mchr1 | melanin-concentrating hormone | 0.0002512 | 0.009689 | 0.5332 | -0.9072 | -4.442 | 0.2312 | 0.5928 | 0.8131 | -0.2984 | -1.235 | 0.3937 | 0.6435 | 0.8735 | -0.195 | -0.8717 | 4.773e-07 | 2.705e-05 | 0.3228 | -1.631 | -7.291 |  |
| Abcg1 | ATP binding cassette subfamily | 0.0003114 | 0.01102 | 4.673 | 2.224 | 4.35 | 0.4796 | 0.7753 | 1.353 | 0.436 | 0.7205 | 6.656e-07 | 3.643e-05 | 15.92 | 3.993 | 7.128 | 0.02367 | 0.1046 | 2.588 | 1.372 | 2.449 |  |
| Akap6 | A kinase (PRKA) anchor protein | 0.001296 | 0.02689 | 0.6588 | -0.602 | -3.739 | 0.5894 | 0.8336 | 1.075 | 0.1045 | 0.5485 | 0.01151 | 0.06316 | 0.7117 | -0.4907 | -2.782 | 1.198e-08 | 1.649e-06 | 0.3234 | -1.629 | -9.235 |  |
| AA474408 | expressed sequence AA474408 | 0.1007 | 0.3426 | 1.216 | 0.2823 | 1.721 | 0.0006078 | 0.02136 | 1.727 | 0.7886 | 4.063 | 0.07569 | 0.2359 | 0.7919 | -0.3367 | -1.874 | 2.084e-06 | 8.729e-05 | 0.4406 | -1.182 | -6.58 |  |
| Pbld1 | phenazine biosynthesis-like pr | 0.0002199 | 0.008969 | 1.976 | 0.9829 | 4.499 | 0.07161 | 0.3666 | 1.406 | 0.4918 | 1.903 | 2.992e-06 | 0.0001142 | 2.896 | 1.534 | 6.41 | 0.8016 | 0.9065 | 0.9586 | -0.06094 | -0.2546 |  |
| Cav2 | caveolin 2 | 0.0004562 | 0.0139 | 0.5607 | -0.8347 | -4.186 | 0.6649 | 0.8725 | 0.9306 | -0.1037 | -0.4396 | 6.822e-06 | 0.0002202 | 0.4014 | -1.317 | -6.03 | 0.0006884 | 0.007686 | 0.5449 | -0.8759 | -4.01 |  |
| Gm10069 | predicted gene 10069 | 0.7281 | 0.8904 | 0.9611 | -0.05718 | -0.3525 | 0.004058 | 0.07695 | 1.54 | 0.6228 | 3.245 | 5.236e-05 | 0.001038 | 0.5323 | -0.9098 | -5.12 | 3.323e-06 | 0.0001221 | 0.4568 | -1.13 | -6.361 |  |
| Nmrk2 | nicotinamide riboside kinase 2 | 0.0001805 | 0.00795 | 0.1878 | -2.413 | -4.584 | 0.3133 | 0.6637 | 0.6398 | -0.6443 | -1.034 | 0.667 | 0.8398 | 0.8398 | -0.2518 | -0.4367 | 2.793e-08 | 3.193e-06 | 0.0301 | -5.054 | -8.765 |  |
| Krt18 | keratin 18 | 0.2535 | 0.5572 | 0.8406 | -0.2504 | -1.176 | 0.001044 | 0.03197 | 0.512 | -0.9657 | -3.832 | 0.0001102 | 0.001861 | 2.172 | 1.119 | 4.797 | 0.000277 | 0.003867 | 2.037 | 1.027 | 4.4 |  |
| Hba-a1 | hemoglobin alpha, adult chain | 0.03922 | 0.2048 | 0.649 | -0.6238 | -2.206 | 0.000491 | 0.01841 | 0.3816 | -1.39 | -4.155 | 0.001676 | 0.0152 | 2.179 | 1.124 | 3.629 | 0.001147 | 0.01129 | 2.257 | 1.174 | 3.791 |  |
| Ociad2 | OCIA domain containing 2 | 3.366e-05 | 0.002839 | 0.4882 | -1.035 | -5.314 | 2.696e-05 | 0.002266 | 0.4214 | -1.247 | -5.412 | 0.007158 | 0.04444 | 1.557 | 0.6388 | 2.995 | 0.9912 | 0.9954 | 0.9983 | -0.002388 | -0.0112 |  |
| Pter | phosphotriesterase related | 0.04414 | 0.2193 | 0.7441 | -0.4265 | -2.148 | 0.0005435 | 0.01975 | 0.512 | -0.9657 | -4.111 | 0.0326 | 0.1334 | 1.414 | 0.4994 | 2.297 | 4.979e-05 | 0.001033 | 2.171 | 1.118 | 5.142 |  |
| Gm34595 | NA | 0.0001934 | 0.008211 | 0.2819 | -1.827 | -4.554 | 0.0249 | 0.2171 | 0.4503 | -1.151 | -2.425 | 0.6489 | 0.828 | 1.151 | 0.2032 | 0.4623 | 5.8e-05 | 0.00116 | 0.2131 | -2.23 | -5.075 |  |
| Myo19 | myosin XIX | 0.512 | 0.7688 | 0.9231 | -0.1154 | -0.6676 | 0.009975 | 0.1308 | 1.497 | 0.5824 | 2.847 | 2.011e-06 | 8.317e-05 | 0.4206 | -1.249 | -6.597 | 0.0002428 | 0.003487 | 0.5571 | -0.8441 | -4.456 |  |
| Mmp15 | matrix metallopeptidase 15 | 7.699e-05 | 0.00469 | 0.5082 | -0.9766 | -4.952 | 4.709e-05 | 0.003375 | 0.4336 | -1.206 | -5.167 | 0.1449 | 0.3588 | 1.255 | 0.3278 | 1.517 | 0.112 | 0.2984 | 1.283 | 0.3591 | 1.662 |  |
| H3f3b | H3.3 histone B | 0.0003504 | 0.01188 | 1.435 | 0.5215 | 4.299 | 0.2365 | 0.5963 | 1.129 | 0.1752 | 1.221 | 0.6695 | 0.8415 | 0.9609 | -0.05756 | -0.4332 | 7.26e-08 | 6.553e-06 | 2.138 | 1.097 | 8.252 |  |
| Fam78a | family with sequence similarit | 0.06194 | 0.2643 | 0.7506 | -0.414 | -1.978 | 0.03773 | 0.2728 | 1.465 | 0.5512 | 2.225 | 0.2356 | 0.4826 | 1.215 | 0.2804 | 1.223 | 1.057e-11 | 8.236e-09 | 0.1107 | -3.176 | -13.85 |  |
| Clybl | citrate lyase beta like | 0.001107 | 0.02416 | 0.6629 | -0.593 | -3.807 | 0.8538 | 0.9513 | 1.024 | 0.03442 | 0.1867 | 1.373e-06 | 6.199e-05 | 0.4485 | -1.157 | -6.778 | 0.0003914 | 0.005016 | 0.6047 | -0.7256 | -4.252 |  |
| Tspan12 | tetraspanin 12 | 0.07235 | 0.2874 | 0.7378 | -0.4386 | -1.897 | 0.114 | 0.4466 | 1.368 | 0.4521 | 1.653 | 7.083e-06 | 0.0002266 | 0.348 | -1.523 | -6.013 | 0.0002004 | 0.00299 | 0.4508 | -1.15 | -4.539 |  |
| Eif4ebp1 | eukaryotic translation initiat | 0.0004899 | 0.0145 | 1.455 | 0.5412 | 4.156 | 0.3946 | 0.7237 | 0.9113 | -0.1341 | -0.87 | 0.5802 | 0.7857 | 1.057 | 0.08022 | 0.5623 | 1.399e-11 | 9.995e-09 | 3.852 | 1.946 | 13.64 |  |
| Erlin1 | ER lipid raft associated 1 | 0.2611 | 0.5663 | 0.8636 | -0.2115 | -1.156 | 0.000873 | 0.02779 | 0.5564 | -0.8458 | -3.908 | 0.00454 | 0.03175 | 1.559 | 0.6404 | 3.196 | 3.779e-06 | 0.0001358 | 2.399 | 1.263 | 6.301 |  |
| Gm15590 | predicted gene 15590 | 0.1138 | 0.3662 | 1.188 | 0.2479 | 1.654 | 0.03597 | 0.2661 | 0.7584 | -0.3989 | -2.249 | 0.02874 | 0.1222 | 1.308 | 0.3871 | 2.357 | 8.09e-09 | 1.242e-06 | 2.935 | 1.553 | 9.458 |  |
| AF357355 | NA | 0.0003191 | 0.01121 | 0.5434 | -0.88 | -4.339 | 0.223 | 0.5841 | 0.8112 | -0.3018 | -1.258 | 0.01801 | 0.08753 | 0.6724 | -0.5725 | -2.577 | 4.065e-05 | 0.0008787 | 0.4468 | -1.162 | -5.231 |  |
| Nfe2l2 | nuclear factor, erythroid deri | 0.001367 | 0.02766 | 1.42 | 0.5061 | 3.716 | 0.6209 | 0.8509 | 0.9454 | -0.08096 | -0.5024 | 0.007511 | 0.04599 | 1.36 | 0.4436 | 2.974 | 2.471e-08 | 2.876e-06 | 2.492 | 1.318 | 8.832 |  |
| Vldlr | very low density lipoprotein r | 0.2778 | 0.5833 | 1.123 | 0.1671 | 1.116 | 0.0953 | 0.4135 | 1.24 | 0.3102 | 1.751 | 4.744e-07 | 2.825e-05 | 0.4363 | -1.197 | -7.294 | 0.0001456 | 0.002343 | 1.702 | 0.7671 | 4.676 |  |
| Pmf1 | polyamine-modulated factor 1 | 0.01652 | 0.1238 | 0.6634 | -0.592 | -2.617 | 0.0003708 | 0.01497 | 0.4524 | -1.144 | -4.275 | 0.004123 | 0.02963 | 1.744 | 0.8025 | 3.238 | 0.002552 | 0.02061 | 1.808 | 0.8542 | 3.447 |  |
| Cgref1 | cell growth regulator with EF | 0.8413 | 0.9403 | 0.9718 | -0.0412 | -0.2029 | 0.00347 | 0.06884 | 1.737 | 0.7963 | 3.314 | 1.207e-05 | 0.0003396 | 0.4107 | -1.284 | -5.771 | 2.105e-05 | 0.0005251 | 0.4267 | -1.229 | -5.522 |  |
| Adrbk2 | NA | 0.001531 | 0.02966 | 0.598 | -0.7418 | -3.667 | 0.7909 | 0.9254 | 1.046 | 0.06431 | 0.2687 | 1.696e-05 | 0.0004372 | 0.422 | -1.245 | -5.618 | 3.504e-05 | 0.0007851 | 0.4433 | -1.174 | -5.296 |  |
| Cxcl14 | chemokine (C-X-C motif) ligand | 6.646e-05 | 0.00433 | 0.4855 | -1.043 | -5.016 | 0.002574 | 0.05678 | 0.556 | -0.8468 | -3.443 | 0.4656 | 0.703 | 1.125 | 0.1694 | 0.7439 | 0.003398 | 0.02553 | 0.5919 | -0.7565 | -3.323 |  |
| Itga7 | integrin alpha 7 | 0.002021 | 0.03517 | 0.5833 | -0.7776 | -3.548 | 0.7104 | 0.8923 | 1.07 | 0.09769 | 0.3767 | 3.037e-06 | 0.0001157 | 0.3445 | -1.537 | -6.403 | 0.0002027 | 0.003014 | 0.4702 | -1.089 | -4.534 |  |
| Plxna3 | plexin A3 | 0.0001898 | 0.008099 | 0.6887 | -0.5381 | -4.562 | 0.3791 | 0.7117 | 0.9167 | -0.1255 | -0.8995 | 1.926e-08 | 2.54e-06 | 0.4478 | -1.159 | -8.97 | 0.5487 | 0.748 | 0.9468 | -0.07883 | -0.6101 |  |
| Psmb10 | proteasome (prosome, macropain | 0.3328 | 0.6326 | 0.8497 | -0.2349 | -0.9925 | 0.001159 | 0.03432 | 0.4794 | -1.061 | -3.787 | 0.0003069 | 0.004105 | 2.188 | 1.129 | 4.356 | 4.386e-05 | 0.0009363 | 2.545 | 1.348 | 5.198 |  |
| Dld | dihydrolipoamide dehydrogenase | 0.01163 | 0.1005 | 0.7211 | -0.4718 | -2.777 | 0.3246 | 0.6725 | 1.151 | 0.203 | 1.01 | 2.474e-07 | 1.711e-05 | 0.3743 | -1.418 | -7.619 | 0.003934 | 0.02848 | 0.6568 | -0.6064 | -3.259 |  |
| Gm8181 | predicted gene 8181 | 0.001606 | 0.03048 | 1.346 | 0.4287 | 3.647 | 0.5502 | 0.8141 | 0.9431 | -0.08454 | -0.6078 | 5.648e-09 | 1.016e-06 | 2.37 | 1.245 | 9.666 | 0.03297 | 0.1322 | 1.227 | 0.295 | 2.291 |  |
| Gale | galactose-4-epimerase, UDP | 0.0002501 | 0.009689 | 0.5379 | -0.8945 | -4.444 | 0.0001029 | 0.006057 | 0.4508 | -1.149 | -4.826 | 0.7847 | 0.9024 | 1.043 | 0.06105 | 0.2769 | 0.004581 | 0.03172 | 1.629 | 0.7039 | 3.192 |  |
| Calm2 | calmodulin 2 | 3.729e-06 | 0.0006526 | 0.5497 | -0.8633 | -6.308 | 0.6745 | 0.8762 | 0.9533 | -0.06903 | -0.4263 | 0.8007 | 0.9111 | 0.9738 | -0.03836 | -0.2559 | 1.138e-12 | 1.463e-09 | 0.1969 | -2.344 | -15.64 |  |
| Gm4956 | predicted gene 4956 | 0.03292 | 0.1858 | 1.384 | 0.4688 | 2.292 | 0.1881 | 0.5497 | 0.7956 | -0.3298 | -1.363 | 0.0004565 | 0.005592 | 1.916 | 0.9379 | 4.186 | 2.438e-06 | 9.77e-05 | 2.747 | 1.458 | 6.506 |  |
| Dhrs9 | dehydrogenase/reductase (SDR f | 0.8306 | 0.9359 | 0.9589 | -0.06058 | -0.2168 | 0.001916 | 0.04706 | 0.4411 | -1.181 | -3.571 | 1.951e-05 | 0.0004845 | 3.251 | 1.701 | 5.556 | 2.637e-05 | 0.0006228 | 3.16 | 1.66 | 5.422 |  |
| Lhpp | phospholysine phosphohistidine | 0.5377 | 0.7869 | 0.9015 | -0.1496 | -0.627 | 0.009972 | 0.1308 | 1.746 | 0.8038 | 2.847 | 8.742e-06 | 0.0002644 | 0.3423 | -1.547 | -5.917 | 6.502e-05 | 0.001279 | 0.4023 | -1.314 | -5.026 |  |
| Kctd20 | potassium channel tetramerisat | 8.61e-05 | 0.005 | 1.621 | 0.6968 | 4.904 | 0.9877 | 0.9965 | 0.9982 | -0.002617 | -0.01556 | 0.03326 | 0.1355 | 1.28 | 0.356 | 2.287 | 4.672e-10 | 1.306e-07 | 3.346 | 1.742 | 11.19 |  |
| Dysf | dysferlin | 0.1774 | 0.4642 | 0.8696 | -0.2016 | -1.398 | 0.000404 | 0.01589 | 0.6059 | -0.7229 | -4.238 | 1.037e-08 | 1.588e-06 | 2.773 | 1.471 | 9.317 | 0.5636 | 0.7588 | 1.066 | 0.09273 | 0.5872 |  |
| Unc119 | unc-119 lipid binding chaperon | 0.0003683 | 0.01219 | 0.6776 | -0.5614 | -4.278 | 0.9945 | 0.9987 | 1.001 | 0.001076 | 0.006929 | 4.598e-08 | 4.566e-06 | 0.4289 | -1.221 | -8.495 | 0.004084 | 0.02927 | 0.7239 | -0.4662 | -3.243 |  |
| Gm5597 | predicted gene 5597 | 0.2729 | 0.5787 | 1.166 | 0.2221 | 1.128 | 0.001014 | 0.03131 | 1.861 | 0.8959 | 3.844 | 3.74e-06 | 0.0001354 | 0.3894 | -1.361 | -6.306 | 0.006146 | 0.03931 | 0.6325 | -0.6608 | -3.063 |  |
| Mir1a-1 | microRNA 1a-1 | 0.0001104 | 0.005829 | 0.4827 | -1.051 | -4.796 | 0.3629 | 0.7005 | 0.8459 | -0.2414 | -0.9311 | 0.9897 | 0.9969 | 1.002 | 0.003142 | 0.01309 | 2.575e-09 | 5.056e-07 | 0.1854 | -2.431 | -10.13 |  |
| Mir29a | microRNA 29a | 0.05843 | 0.2563 | 1.313 | 0.3932 | 2.007 | 0.101 | 0.4237 | 0.7586 | -0.3986 | -1.72 | 0.004003 | 0.02903 | 1.622 | 0.6977 | 3.251 | 1.673e-07 | 1.209e-05 | 3.199 | 1.678 | 7.818 |  |
| Mmachc | methylmalonic aciduria cblC ty | 0.07998 | 0.3019 | 0.8272 | -0.2736 | -1.845 | 0.09281 | 0.4104 | 1.24 | 0.3098 | 1.765 | 3.847e-07 | 2.409e-05 | 0.4346 | -1.202 | -7.398 | 0.003026 | 0.0233 | 0.6839 | -0.5482 | -3.373 |  |
| Gbas | NA | 0.5032 | 0.7635 | 0.9296 | -0.1053 | -0.6818 | 0.01153 | 0.1419 | 1.422 | 0.5083 | 2.781 | 2.222e-06 | 9.031e-05 | 0.4639 | -1.108 | -6.55 | 0.0003258 | 0.00438 | 0.6018 | -0.7327 | -4.33 |  |
| Maf | avian musculoaponeurotic fibro | 0.003352 | 0.04701 | 0.6877 | -0.5401 | -3.329 | 0.3237 | 0.6719 | 0.874 | -0.1943 | -1.012 | 0.03022 | 0.1267 | 1.333 | 0.4146 | 2.333 | 8.48e-08 | 7.444e-06 | 0.3655 | -1.452 | -8.17 |  |
| Gm5510 | predicted pseudogene 5510 | 1.752e-06 | 0.0003852 | 0.4654 | -1.103 | -6.662 | 9.621e-05 | 0.005769 | 0.5171 | -0.9515 | -4.855 | 0.03349 | 0.1359 | 0.7504 | -0.4143 | -2.283 | 0.822 | 0.917 | 0.9717 | -0.04137 | -0.228 |  |
| Plbd1 | phospholipase B domain contain | 0.6329 | 0.8426 | 1.077 | 0.1064 | 0.485 | 0.001304 | 0.03704 | 1.958 | 0.9695 | 3.736 | 2.355e-07 | 1.642e-05 | 0.28 | -1.836 | -7.644 | 0.005225 | 0.03491 | 0.5934 | -0.753 | -3.135 |  |
| Chpf2 | chondroitin polymerizing facto | 0.2013 | 0.4962 | 1.154 | 0.2064 | 1.322 | 0.4463 | 0.7567 | 1.105 | 0.1436 | 0.777 | 2.658e-06 | 0.0001042 | 0.4645 | -1.106 | -6.466 | 2.745e-07 | 1.743e-05 | 2.453 | 1.295 | 7.567 |  |
| Nrarp | Notch-regulated ankyrin repeat | 0.003285 | 0.04659 | 0.6253 | -0.6774 | -3.337 | 0.6134 | 0.8468 | 1.089 | 0.1233 | 0.5133 | 1.868e-06 | 7.865e-05 | 0.3599 | -1.474 | -6.632 | 0.0004719 | 0.005754 | 0.5258 | -0.9275 | -4.172 |  |
| Laptm4b | lysosomal-associated protein t | 0.03841 | 0.2027 | 0.8037 | -0.3152 | -2.217 | 0.1849 | 0.5461 | 1.174 | 0.2311 | 1.373 | 2.026e-06 | 8.357e-05 | 0.4907 | -1.027 | -6.593 | 0.0008355 | 0.008895 | 0.6544 | -0.6118 | -3.927 |  |
| Rab1b | RAB1B, member RAS oncogene fam | 0.05338 | 0.2442 | 0.7945 | -0.3318 | -2.053 | 0.1138 | 0.4462 | 1.245 | 0.3162 | 1.654 | 9.053e-08 | 7.976e-06 | 0.3685 | -1.44 | -8.136 | 0.01 | 0.05602 | 0.7053 | -0.5037 | -2.846 |  |
| Glul | glutamate-ammonia ligase (glut | 0.000385 | 0.01253 | 1.966 | 0.9753 | 4.259 | 0.927 | 0.9767 | 1.018 | 0.02513 | 0.09276 | 0.001805 | 0.016 | 1.869 | 0.9023 | 3.597 | 1.853e-07 | 1.302e-05 | 3.859 | 1.948 | 7.766 |  |
| Vkorc1l1 | vitamin K epoxide reductase co | 0.8176 | 0.9306 | 0.9782 | -0.0318 | -0.2337 | 0.002637 | 0.05768 | 1.467 | 0.5528 | 3.433 | 0.0006823 | 0.007571 | 0.6605 | -0.5984 | -4.014 | 1.912e-07 | 1.34e-05 | 0.449 | -1.155 | -7.75 |  |
| Nsmaf | neutral sphingomyelinase (N-SM | 0.000477 | 0.01427 | 1.367 | 0.451 | 4.167 | 0.8778 | 0.9588 | 0.9863 | -0.01994 | -0.1557 | 1.383e-08 | 1.966e-06 | 2.122 | 1.085 | 9.154 | 0.01202 | 0.06436 | 1.255 | 0.3275 | 2.763 |  |
| Jag1 | jagged 1 | 0.5771 | 0.8109 | 1.076 | 0.1056 | 0.5669 | 0.005398 | 0.09124 | 0.6207 | -0.6879 | -3.12 | 0.002317 | 0.01928 | 1.638 | 0.7122 | 3.489 | 8.422e-08 | 7.419e-06 | 3.179 | 1.668 | 8.174 |  |
| Ddx21 | DEAD (Asp-Glu-Ala-Asp) box pol | 0.0004037 | 0.01287 | 1.507 | 0.5913 | 4.238 | 0.6114 | 0.8457 | 1.061 | 0.0852 | 0.5162 | 0.005578 | 0.03698 | 1.39 | 0.4746 | 3.106 | 1.234e-06 | 5.823e-05 | 2.062 | 1.044 | 6.829 |  |
| Slc25a35 | solute carrier family 25, memb | 0.0007044 | 0.01805 | 0.545 | -0.8757 | -4 | 0.982 | 0.9947 | 1.004 | 0.005917 | 0.02284 | 9.855e-07 | 4.926e-05 | 0.3156 | -1.664 | -6.937 | 0.0005685 | 0.006656 | 0.5065 | -0.9813 | -4.092 |  |
| Lin7a | lin-7 homolog A (C. elegans) | 0.413 | 0.6998 | 0.8613 | -0.2154 | -0.8361 | 0.001448 | 0.03946 | 0.4585 | -1.125 | -3.692 | 0.000238 | 0.003373 | 2.395 | 1.26 | 4.465 | 4.292e-05 | 0.0009208 | 2.769 | 1.469 | 5.207 |  |
| Cntrl | centriolin | 0.001608 | 0.0305 | 1.694 | 0.7605 | 3.646 | 0.0001813 | 0.008847 | 2.19 | 1.131 | 4.582 | 0.007877 | 0.04772 | 0.6265 | -0.6746 | -2.953 | 0.07086 | 0.2235 | 0.7392 | -0.4359 | -1.908 |  |
| Rps15 | ribosomal protein S15 | 0.3743 | 0.6687 | 0.8767 | -0.1899 | -0.9089 | 0.00135 | 0.03754 | 0.5285 | -0.9199 | -3.722 | 6.11e-06 | 0.0002028 | 2.623 | 1.391 | 6.08 | 0.00231 | 0.01909 | 1.74 | 0.7987 | 3.49 |  |
| Prkch | protein kinase C, eta | 1.081e-05 | 0.001362 | 2.061 | 1.044 | 5.821 | 1.856e-05 | 0.001723 | 2.271 | 1.183 | 5.578 | 0.3012 | 0.556 | 0.8655 | -0.2084 | -1.061 | 0.2957 | 0.5367 | 0.864 | -0.2109 | -1.074 |  |
| Trpv1 | transient receptor potential c | 0.001201 | 0.02571 | 0.719 | -0.476 | -3.772 | 0.6556 | 0.8678 | 1.048 | 0.06761 | 0.4528 | 9.034e-09 | 1.408e-06 | 0.4064 | -1.299 | -9.395 | 0.02622 | 0.113 | 0.7945 | -0.3319 | -2.401 |  |
| Gm22887 | predicted gene, 22887 | 0.0186 | 0.1326 | 1.539 | 0.6224 | 2.562 | 0.0005222 | 0.01924 | 2.276 | 1.187 | 4.128 | 0.04317 | 0.1616 | 0.6715 | -0.5746 | -2.159 | 0.0004399 | 0.005488 | 0.4607 | -1.118 | -4.202 |  |
| Rpusd3 | RNA pseudouridylate synthase d | 0.0007132 | 0.0182 | 0.5865 | -0.7699 | -3.995 | 0.0001526 | 0.0079 | 0.4791 | -1.062 | -4.656 | 0.03028 | 0.1268 | 1.407 | 0.4923 | 2.332 | 0.08466 | 0.2502 | 1.304 | 0.383 | 1.814 |  |
| Gm379 | predicted gene 379 | 0.01081 | 0.09668 | 0.6513 | -0.6185 | -2.811 | 0.0003496 | 0.01428 | 0.4602 | -1.12 | -4.3 | 0.003668 | 0.02713 | 1.733 | 0.793 | 3.29 | 0.007981 | 0.04756 | 1.636 | 0.7104 | 2.947 |  |
| Igfbp3 | insulin-like growth factor bin | 0.002095 | 0.03592 | 2.108 | 1.076 | 3.532 | 0.7207 | 0.8964 | 0.9134 | -0.1306 | -0.3626 | 1.303e-06 | 6.069e-05 | 4.822 | 2.27 | 6.803 | 0.0006334 | 0.007215 | 2.549 | 1.35 | 4.046 |  |
| Ctdp1 | CTD (carboxy-terminal domain, | 0.0003337 | 0.01146 | 1.609 | 0.686 | 4.32 | 0.4479 | 0.7576 | 1.106 | 0.1454 | 0.7742 | 0.1903 | 0.4232 | 1.178 | 0.2358 | 1.356 | 1.254e-07 | 1.002e-05 | 2.613 | 1.386 | 7.967 |  |
| Hyi | hydroxypyruvate isomerase (put | 0.3959 | 0.6868 | 0.8814 | -0.1821 | -0.8676 | 0.001461 | 0.0396 | 0.5301 | -0.9157 | -3.687 | 1.265e-05 | 0.0003519 | 2.5 | 1.322 | 5.75 | 0.001065 | 0.01065 | 1.839 | 0.879 | 3.823 |  |
| Slc7a1 | solute carrier family 7 (catio | 0.0007442 | 0.01869 | 1.5 | 0.5849 | 3.977 | 0.2461 | 0.6057 | 1.155 | 0.208 | 1.195 | 0.4492 | 0.6904 | 0.9174 | -0.1244 | -0.772 | 1.766e-07 | 1.255e-05 | 2.387 | 1.255 | 7.791 |  |
| Per3 | period circadian clock 3 | 0.01844 | 0.132 | 0.6488 | -0.6242 | -2.566 | 0.2835 | 0.6387 | 1.246 | 0.3172 | 1.102 | 0.000456 | 0.005592 | 0.4615 | -1.116 | -4.186 | 2.981e-06 | 0.0001123 | 0.3059 | -1.709 | -6.412 |  |
| 4930429B21Rik | NA | 0.6979 | 0.8766 | 0.9525 | -0.07019 | -0.3937 | 0.006526 | 0.1024 | 1.559 | 0.6404 | 3.036 | 1.927e-06 | 8.083e-05 | 0.4083 | -1.292 | -6.617 | 0.0003496 | 0.004611 | 0.5588 | -0.8397 | -4.3 |  |
| Atad2 | ATPase family, AAA domain cont | 0.0004317 | 0.01343 | 1.595 | 0.6735 | 4.21 | 0.6061 | 0.8436 | 1.071 | 0.09916 | 0.5239 | 0.006518 | 0.04151 | 1.446 | 0.5322 | 3.037 | 1.326e-06 | 6.136e-05 | 2.283 | 1.191 | 6.795 |  |
| Eda2r | ectodysplasin A2 receptor | 0.0001349 | 0.006523 | 1.701 | 0.7664 | 4.709 | 0.8402 | 0.9464 | 0.9731 | -0.03933 | -0.2042 | 0.2116 | 0.4517 | 0.8526 | -0.2301 | -1.291 | 2.955e-12 | 3.041e-09 | 6.265 | 2.647 | 14.85 |  |
| Rabl3 | RAB, member RAS oncogene famil | 0.513 | 0.7697 | 1.094 | 0.13 | 0.666 | 0.001637 | 0.04289 | 1.79 | 0.8402 | 3.639 | 0.0004959 | 0.005961 | 0.5406 | -0.8873 | -4.15 | 1.092e-05 | 0.0003174 | 0.4224 | -1.243 | -5.816 |  |
| Tapt1 | transmembrane anterior posteri | 0.0002857 | 0.0104 | 1.502 | 0.5865 | 4.387 | 0.6348 | 0.8581 | 1.054 | 0.0763 | 0.4823 | 2.799e-08 | 3.234e-06 | 2.434 | 1.284 | 8.764 | 0.1072 | 0.2903 | 1.187 | 0.247 | 1.687 |  |
| C4bp-ps1 | complement component 4 binding | 0.1361 | 0.4055 | 1.306 | 0.3851 | 1.553 | 0.0009726 | 0.03033 | 2.193 | 1.133 | 3.862 | 0.002092 | 0.0179 | 0.5142 | -0.9597 | -3.533 | 0.000129 | 0.002126 | 0.4105 | -1.285 | -4.729 |  |
| Prodh | proline dehydrogenase | 0.0007031 | 0.01805 | 1.812 | 0.858 | 4.001 | 0.05592 | 0.3263 | 1.429 | 0.515 | 2.03 | 0.3793 | 0.6302 | 0.8638 | -0.2112 | -0.8992 | 3.641e-05 | 0.0008073 | 2.362 | 1.24 | 5.28 |  |
| Ccdc88c | coiled-coil domain containing | 0.0003233 | 0.01126 | 0.6462 | -0.6299 | -4.334 | 0.4938 | 0.7835 | 0.9203 | -0.1199 | -0.6971 | 0.1961 | 0.4313 | 0.8628 | -0.213 | -1.337 | 6.994e-08 | 6.357e-06 | 0.4014 | -1.317 | -8.272 |  |
| Hcar1 | hydrocarboxylic acid receptor | 0.001107 | 0.02416 | 0.4536 | -1.14 | -3.807 | 0.7463 | 0.9071 | 1.084 | 0.1163 | 0.3281 | 2.862e-08 | 3.272e-06 | 0.1366 | -2.872 | -8.751 | 0.01273 | 0.06707 | 0.5366 | -0.898 | -2.736 |  |
| Gm5105 | predicted gene 5105 | 0.0003656 | 0.01215 | 0.6105 | -0.7119 | -4.281 | 0.1996 | 0.5623 | 0.8345 | -0.261 | -1.326 | 0.8455 | 0.9343 | 1.025 | 0.03597 | 0.1974 | 2.457e-07 | 1.624e-05 | 0.3819 | -1.389 | -7.623 |  |
| Pld2 | phospholipase D2 | 0.0003934 | 0.01265 | 0.65 | -0.6214 | -4.25 | 0.2303 | 0.5914 | 0.8621 | -0.2141 | -1.237 | 0.249 | 0.4989 | 0.8765 | -0.1902 | -1.187 | 2.799e-06 | 0.0001072 | 0.4891 | -1.032 | -6.441 |  |
| Hba-a2 | hemoglobin alpha, adult chain | 0.04677 | 0.2274 | 0.6434 | -0.6363 | -2.12 | 0.0006635 | 0.02273 | 0.3711 | -1.43 | -4.026 | 0.00153 | 0.0142 | 2.307 | 1.206 | 3.668 | 0.001907 | 0.01654 | 2.258 | 1.175 | 3.573 |  |
| Coro6 | coronin 6 | 0.0002508 | 0.009689 | 1.813 | 0.858 | 4.443 | 0.09423 | 0.4118 | 1.321 | 0.4015 | 1.757 | 2.971e-05 | 0.0006775 | 2.198 | 1.136 | 5.369 | 0.2828 | 0.5228 | 1.176 | 0.2335 | 1.104 |  |
| Mtmr11 | myotubularin related protein 1 | 0.2554 | 0.5597 | 0.8022 | -0.318 | -1.171 | 0.001327 | 0.03726 | 0.4358 | -1.198 | -3.729 | 0.0001269 | 0.002075 | 2.655 | 1.409 | 4.736 | 0.0005065 | 0.006085 | 2.349 | 1.232 | 4.141 |  |
| Jph2 | junctophilin 2 | 0.00086 | 0.02056 | 0.6051 | -0.7247 | -3.915 | 0.0834 | 0.3925 | 0.7583 | -0.3992 | -1.822 | 0.3637 | 0.6169 | 1.14 | 0.1885 | 0.9295 | 1.277e-05 | 0.0003594 | 0.4459 | -1.165 | -5.746 |  |
| BC005685 | NA | 0.0001441 | 0.006824 | 4.233 | 2.082 | 4.681 | 0.03964 | 0.2787 | 2.232 | 1.158 | 2.201 | 0.01629 | 0.08118 | 2.425 | 1.278 | 2.623 | 0.006158 | 0.03935 | 2.812 | 1.492 | 3.062 |  |
| Mir107 | microRNA 107 | 0.02476 | 0.1572 | 0.6618 | -0.5956 | -2.428 | 0.0005255 | 0.01931 | 0.4361 | -1.197 | -4.126 | 0.003098 | 0.02396 | 1.871 | 0.9037 | 3.363 | 0.003145 | 0.02404 | 1.869 | 0.9019 | 3.356 |  |
| Ephb2 | Eph receptor B2 | 0.007564 | 0.07809 | 0.7131 | -0.4879 | -2.971 | 0.3053 | 0.6593 | 1.152 | 0.2044 | 1.052 | 4.918e-09 | 9.218e-07 | 0.2966 | -1.753 | -9.747 | 0.0873 | 0.2552 | 0.7991 | -0.3235 | -1.798 |  |
| Gm40902 | NA | 0.0001016 | 0.005524 | 2.646 | 1.404 | 4.832 | 0.02742 | 0.2289 | 1.763 | 0.818 | 2.379 | 0.006834 | 0.04304 | 1.945 | 0.9598 | 3.016 | 0.02095 | 0.09565 | 1.738 | 0.7978 | 2.507 |  |
| Cyp2d40 | cytochrome P450, family 2, sub | 0.03226 | 0.1838 | 0.7005 | -0.5135 | -2.301 | 0.0005546 | 0.02005 | 0.472 | -1.083 | -4.103 | 0.000391 | 0.004966 | 2.055 | 1.039 | 4.252 | 0.01266 | 0.06686 | 1.59 | 0.6695 | 2.739 |  |
| Twf2 | twinfilin actin binding protei | 0.001429 | 0.02844 | 1.362 | 0.4456 | 3.697 | 0.5245 | 0.8007 | 0.938 | -0.09237 | -0.6477 | 0.2124 | 0.4528 | 0.8888 | -0.1701 | -1.288 | 1.653e-11 | 1.149e-08 | 3.444 | 1.784 | 13.51 |  |
| Tnxb | tenascin XB | 0.08604 | 0.315 | 0.773 | -0.3714 | -1.806 | 0.0007942 | 0.02583 | 0.5137 | -0.961 | -3.949 | 0.0001185 | 0.001969 | 2.105 | 1.074 | 4.765 | 0.00584 | 0.03789 | 1.619 | 0.6951 | 3.085 |  |
| Ctgf | NA | 0.0007532 | 0.01884 | 3.081 | 1.623 | 3.972 | 0.0001667 | 0.00838 | 4.702 | 2.233 | 4.618 | 0.01817 | 0.08804 | 0.45 | -1.152 | -2.573 | 0.1457 | 0.3516 | 0.6251 | -0.6779 | -1.514 |  |
| Spice1 | spindle and centriole associat | 0.000351 | 0.01188 | 1.574 | 0.6546 | 4.298 | 0.6844 | 0.8815 | 1.053 | 0.07432 | 0.4124 | 0.04734 | 0.1721 | 1.277 | 0.3526 | 2.113 | 6.364e-08 | 5.996e-06 | 2.618 | 1.388 | 8.322 |  |
| Ppargc1a | peroxisome proliferative activ | 0.4237 | 0.7083 | 1.185 | 0.2454 | 0.8167 | 0.001161 | 0.03432 | 2.542 | 1.346 | 3.786 | 0.02208 | 0.1012 | 0.5676 | -0.817 | -2.482 | 2.835e-07 | 1.792e-05 | 0.1786 | -2.485 | -7.551 |  |
| 1700025G04Rik | RIKEN cDNA 1700025G04 gene | 0.02901 | 0.1724 | 0.7432 | -0.4283 | -2.353 | 0.2489 | 0.6085 | 1.194 | 0.2558 | 1.188 | 1.242e-05 | 0.0003474 | 0.4512 | -1.148 | -5.758 | 0.0002029 | 0.003015 | 0.5344 | -0.904 | -4.534 |  |
| Mfhas1 | malignant fibrous histiocytoma | 0.3989 | 0.6891 | 0.8793 | -0.1856 | -0.862 | 0.001625 | 0.04275 | 0.5256 | -0.9281 | -3.642 | 0.000389 | 0.004947 | 2.005 | 1.004 | 4.254 | 4.507e-05 | 0.0009565 | 2.335 | 1.223 | 5.186 |  |
| Sgpl1 | sphingosine phosphate lyase 1 | 0.000577 | 0.01603 | 2.036 | 1.025 | 4.086 | 0.8236 | 0.9395 | 1.048 | 0.06706 | 0.2258 | 4.567e-07 | 2.745e-05 | 4.029 | 2.011 | 7.313 | 0.00511 | 0.03432 | 1.821 | 0.8645 | 3.144 |  |
| Ceacam2 | carcinoembryonic antigen-relat | 0.02213 | 0.1467 | 1.397 | 0.4826 | 2.481 | 0.2314 | 0.5929 | 0.8213 | -0.2841 | -1.234 | 8.142e-08 | 7.325e-06 | 3.353 | 1.746 | 8.192 | 0.01706 | 0.08239 | 1.469 | 0.5545 | 2.602 |  |
| Hbb-bt | hemoglobin, beta adult t chain | 0.03016 | 0.1762 | 0.6923 | -0.5306 | -2.334 | 0.000589 | 0.02101 | 0.4676 | -1.097 | -4.077 | 0.001496 | 0.01395 | 1.887 | 0.9159 | 3.678 | 0.005144 | 0.03447 | 1.72 | 0.7824 | 3.141 |  |
| Klf15 | Kruppel-like factor 15 | 2.719e-05 | 0.00248 | 2.303 | 1.203 | 5.408 | 0.002117 | 0.0505 | 1.904 | 0.9288 | 3.528 | 0.4445 | 0.6868 | 1.141 | 0.1901 | 0.78 | 0.01714 | 0.08264 | 1.552 | 0.6338 | 2.6 |  |
| Gsta1 | glutathione S-transferase, alp | 0.2514 | 0.5553 | 0.8601 | -0.2174 | -1.181 | 0.001349 | 0.03754 | 0.5701 | -0.8106 | -3.722 | 1.845e-05 | 0.0004681 | 2.181 | 1.125 | 5.581 | 0.004059 | 0.02911 | 1.574 | 0.6544 | 3.245 |  |
| Gsta2 | glutathione S-transferase, alp | 0.1661 | 0.4484 | 0.8319 | -0.2655 | -1.437 | 0.001112 | 0.03354 | 0.562 | -0.8314 | -3.805 | 6.699e-05 | 0.00125 | 2.02 | 1.014 | 5.013 | 0.002984 | 0.02307 | 1.606 | 0.6837 | 3.379 |  |
| Gpr160 | G protein-coupled receptor 160 | 0.0003813 | 0.01246 | 1.789 | 0.8388 | 4.263 | 0.2168 | 0.578 | 1.229 | 0.2969 | 1.275 | 1.582e-06 | 6.922e-05 | 2.725 | 1.446 | 6.71 | 0.4156 | 0.6437 | 1.132 | 0.1792 | 0.8314 |  |
| Txnip | thioredoxin interacting protei | 0.005159 | 0.06207 | 1.631 | 0.706 | 3.14 | 0.0003415 | 0.0141 | 2.214 | 1.147 | 4.31 | 0.03345 | 0.1359 | 0.6771 | -0.5625 | -2.284 | 0.005872 | 0.03805 | 0.5908 | -0.7593 | -3.083 |  |
| Sec14l5 | SEC14-like lipid binding 5 | 1.237e-05 | 0.001466 | 0.4538 | -1.14 | -5.76 | 0.9651 | 0.9885 | 1.007 | 0.01037 | 0.04427 | 0.3989 | 0.6486 | 0.8785 | -0.1869 | -0.8621 | 2.293e-12 | 2.457e-09 | 0.1041 | -3.264 | -15.06 |  |
| Ptpmt1 | protein tyrosine phosphatase, | 0.01146 | 0.09962 | 0.72 | -0.474 | -2.784 | 0.4058 | 0.7304 | 1.126 | 0.1711 | 0.8493 | 0.0001512 | 0.002372 | 0.5475 | -0.869 | -4.66 | 1.361e-05 | 0.0003771 | 0.4776 | -1.066 | -5.717 |  |
| Ramp2 | receptor (calcitonin) activity | 0.08534 | 0.3133 | 1.258 | 0.3306 | 1.81 | 0.1211 | 0.4578 | 0.7846 | -0.3499 | -1.619 | 1.599e-05 | 0.0004181 | 2.188 | 1.129 | 5.645 | 0.0001914 | 0.002884 | 1.882 | 0.9121 | 4.559 |  |
| Apoa4 | apolipoprotein A-IV | 0.6171 | 0.8342 | 1.204 | 0.2676 | 0.5079 | 0.00214 | 0.05092 | 4.583 | 2.196 | 3.523 | 0.0001354 | 0.002171 | 0.1521 | -2.717 | -4.708 | 3.337e-05 | 0.000753 | 0.1192 | -3.069 | -5.318 |  |
| Rxrg | retinoid X receptor gamma | 0.0006795 | 0.01776 | 0.5168 | -0.9523 | -4.016 | 0.692 | 0.8849 | 0.9248 | -0.1128 | -0.4019 | 7.504e-05 | 0.001363 | 0.4091 | -1.289 | -4.963 | 0.000128 | 0.002119 | 0.4265 | -1.229 | -4.732 |  |
| Acat2 | acetyl-Coenzyme A acetyltransf | 0.02418 | 0.1544 | 0.6309 | -0.6644 | -2.439 | 0.000648 | 0.02236 | 0.4059 | -1.301 | -4.036 | 0.0307 | 0.1277 | 1.618 | 0.6939 | 2.325 | 0.0005946 | 0.006889 | 2.322 | 1.215 | 4.073 |  |
| Klf11 | Kruppel-like factor 11 | 0.9812 | 0.9937 | 0.9968 | -0.004677 | -0.02389 | 0.002703 | 0.05866 | 0.5773 | -0.7927 | -3.422 | 2.152e-05 | 0.0005263 | 2.269 | 1.182 | 5.512 | 2.155e-05 | 0.0005331 | 2.269 | 1.182 | 5.512 |  |
| Ino80c | INO80 complex subunit C | 0.02291 | 0.1496 | 0.7571 | -0.4014 | -2.465 | 0.277 | 0.6327 | 1.161 | 0.2154 | 1.118 | 1.162e-06 | 5.566e-05 | 0.4282 | -1.223 | -6.858 | 0.002189 | 0.0184 | 0.6476 | -0.6268 | -3.513 |  |
| Adck3 | NA | 0.08259 | 0.3073 | 1.282 | 0.3581 | 1.828 | 0.0949 | 0.4128 | 0.7545 | -0.4064 | -1.753 | 1.381e-07 | 1.08e-05 | 3.247 | 1.699 | 7.917 | 0.01184 | 0.06384 | 1.51 | 0.5944 | 2.769 |  |
| Sec14l4 | SEC14-like lipid binding 4 | 0.7069 | 0.8803 | 0.9651 | -0.05132 | -0.3815 | 0.003475 | 0.06887 | 1.441 | 0.5273 | 3.313 | 1.643e-08 | 2.261e-06 | 0.3965 | -1.335 | -9.058 | 0.01312 | 0.06845 | 0.7572 | -0.4012 | -2.723 |  |
| Fam107a | family with sequence similarit | 1.931e-07 | 7.415e-05 | 5.071 | 2.342 | 7.745 | 1.386e-06 | 0.000264 | 5.366 | 2.424 | 6.774 | 0.9134 | 0.9646 | 1.026 | 0.0365 | 0.1102 | 0.406 | 0.6365 | 0.8229 | -0.2812 | -0.8489 |  |
| Sult1e1 | sulfotransferase family 1E, me | 0.1559 | 0.4336 | 1.556 | 0.6376 | 1.475 | 0.001246 | 0.03609 | 3.788 | 1.922 | 3.756 | 0.001585 | 0.01454 | 0.3014 | -1.73 | -3.653 | 0.0002303 | 0.003346 | 0.2298 | -2.122 | -4.479 |  |
| Cxadr | coxsackie virus and adenovirus | 0.8913 | 0.959 | 0.9788 | -0.03099 | -0.1385 | 0.002667 | 0.05817 | 0.5331 | -0.9076 | -3.428 | 2.31e-06 | 9.283e-05 | 3.034 | 1.601 | 6.532 | 0.0004445 | 0.005524 | 2.04 | 1.029 | 4.197 |  |
| Dtx2 | deltex 2, E3 ubiquitin ligase | 0.002072 | 0.03574 | 0.7062 | -0.5019 | -3.537 | 0.5937 | 0.8363 | 0.9389 | -0.09101 | -0.5421 | 3.799e-09 | 7.348e-07 | 0.3443 | -1.538 | -9.898 | 0.06311 | 0.2065 | 1.236 | 0.3059 | 1.968 |  |
| Gm5785 | NA | 0.0001058 | 0.005668 | 1.937 | 0.9541 | 4.814 | 8.299e-05 | 0.005194 | 2.225 | 1.154 | 4.919 | 0.02047 | 0.09587 | 0.6847 | -0.5465 | -2.517 | 0.8136 | 0.9129 | 0.9647 | -0.05186 | -0.2389 |  |
| Gm3776 | predicted gene 3776 | 0.3258 | 0.6262 | 0.8777 | -0.1882 | -1.007 | 0.001594 | 0.04214 | 0.5716 | -0.8069 | -3.65 | 1.856e-05 | 0.0004685 | 2.206 | 1.142 | 5.578 | 0.002306 | 0.01907 | 1.641 | 0.7145 | 3.491 |  |
| Odf3l2 | outer dense fiber of sperm tai | 0.0001682 | 0.007582 | 1.883 | 0.9128 | 4.614 | 0.7474 | 0.9075 | 0.9484 | -0.07644 | -0.3266 | 0.4238 | 0.6702 | 1.13 | 0.1769 | 0.8165 | 3.93e-11 | 2.022e-08 | 6.919 | 2.791 | 12.88 |  |
| Hgs | HGF-regulated tyrosine kinase | 0.0003636 | 0.01211 | 1.434 | 0.5197 | 4.283 | 0.2823 | 0.6378 | 1.116 | 0.1586 | 1.105 | 0.8256 | 0.9239 | 1.021 | 0.02968 | 0.2233 | 1.356e-07 | 1.051e-05 | 2.076 | 1.053 | 7.926 |  |
| Gm9909 | NA | 2.493e-05 | 0.00233 | 0.4302 | -1.217 | -5.447 | 0.7824 | 0.9212 | 1.053 | 0.07403 | 0.28 | 0.7918 | 0.9063 | 1.046 | 0.06547 | 0.2675 | 6.683e-13 | 9.551e-10 | 0.06522 | -3.939 | -16.09 |  |
| Rasgef1b | RasGEF domain family, member 1 | 0.000429 | 0.01338 | 2.577 | 1.366 | 4.212 | 0.0001589 | 0.008128 | 3.432 | 1.779 | 4.639 | 0.08822 | 0.2615 | 0.6433 | -0.6365 | -1.792 | 0.1043 | 0.2855 | 0.6577 | -0.6044 | -1.702 |  |
| Tubb2a | tubulin, beta 2A class IIA | 0.7309 | 0.8916 | 0.9351 | -0.09685 | -0.3488 | 0.00261 | 0.05724 | 0.4572 | -1.129 | -3.437 | 8.623e-06 | 0.0002625 | 3.486 | 1.801 | 5.923 | 0.000384 | 0.004946 | 2.455 | 1.296 | 4.26 |  |
| Slc25a17 | solute carrier family 25 (mito | 0.6366 | 0.8447 | 0.9521 | -0.07077 | -0.4798 | 0.001187 | 0.03485 | 0.6333 | -0.6591 | -3.777 | 0.03547 | 0.1414 | 1.287 | 0.3644 | 2.256 | 3.33e-08 | 3.676e-06 | 2.64 | 1.401 | 8.669 |  |
| Rpl3 | ribosomal protein L3 | 0.0004624 | 0.01404 | 1.995 | 0.9961 | 4.18 | 0.2534 | 0.6133 | 1.258 | 0.3316 | 1.176 | 0.005528 | 0.03671 | 1.755 | 0.8117 | 3.11 | 0.0002056 | 0.003047 | 2.269 | 1.182 | 4.528 |  |
| Hamp2 | hepcidin antimicrobial peptide | 0.01407 | 0.1123 | 0.5972 | -0.7438 | -2.691 | 0.0004815 | 0.01822 | 0.3891 | -1.362 | -4.163 | 0.005588 | 0.03702 | 1.919 | 0.9402 | 3.105 | 0.00683 | 0.04259 | 1.883 | 0.9133 | 3.016 |  |
| Sall4 | spalt like transcription facto | 0.0004768 | 0.01427 | 0.6401 | -0.6436 | -4.167 | 0.1948 | 0.5567 | 0.8437 | -0.2452 | -1.342 | 0.2734 | 0.5266 | 0.8763 | -0.1906 | -1.126 | 8.323e-06 | 0.0002546 | 0.4983 | -1.005 | -5.939 |  |
| Dgat2l6 | diacylglycerol O-acyltransfera | 0.0002647 | 0.009954 | 0.6437 | -0.6354 | -4.419 | 0.6922 | 0.8849 | 0.9537 | -0.06835 | -0.4017 | 0.2275 | 0.4721 | 0.8729 | -0.1961 | -1.245 | 6.828e-09 | 1.103e-06 | 0.3523 | -1.505 | -9.556 |  |
| Gm9958 | predicted gene 9958 | 0.00118 | 0.02534 | 1.682 | 0.7505 | 3.779 | 0.0002414 | 0.01103 | 2.067 | 1.048 | 4.459 | 0.09943 | 0.2827 | 0.7706 | -0.3759 | -1.728 | 0.02795 | 0.1177 | 0.6995 | -0.5157 | -2.37 |  |
| Tdg | thymine DNA glycosylase | 0.236 | 0.5376 | 1.174 | 0.2313 | 1.222 | 0.001301 | 0.03698 | 1.786 | 0.837 | 3.737 | 3.168e-06 | 0.0001191 | 0.3995 | -1.324 | -6.384 | 0.02735 | 0.1159 | 0.7103 | -0.4936 | -2.381 |  |
| Gm10053 | predicted gene 10053 | 0.02717 | 0.1659 | 0.7712 | -0.3748 | -2.384 | 0.2551 | 0.6148 | 1.163 | 0.218 | 1.172 | 9.197e-07 | 4.713e-05 | 0.4351 | -1.201 | -6.971 | 0.003157 | 0.0241 | 0.67 | -0.5779 | -3.355 |  |
| Taf1a | TATA-box binding protein assoc | 0.000316 | 0.01113 | 1.819 | 0.8632 | 4.343 | 0.0001443 | 0.007607 | 2.144 | 1.101 | 4.68 | 0.02876 | 0.1222 | 0.7007 | -0.5131 | -2.357 | 0.3718 | 0.6066 | 0.8712 | -0.1989 | -0.9137 |  |
| Arid5a | AT rich interactive domain 5A | 0.001917 | 0.03408 | 1.49 | 0.5751 | 3.571 | 0.7149 | 0.8938 | 0.9522 | -0.07062 | -0.3705 | 0.002593 | 0.02106 | 1.523 | 0.607 | 3.44 | 2.901e-07 | 1.811e-05 | 2.514 | 1.33 | 7.539 |  |
| Prss27 | protease, serine 27 | 0.0004248 | 0.0133 | 0.611 | -0.7108 | -4.217 | 0.257 | 0.6163 | 0.851 | -0.2327 | -1.167 | 7.168e-07 | 3.857e-05 | 0.4035 | -1.309 | -7.092 | 0.5074 | 0.7183 | 0.9172 | -0.1246 | -0.6749 |  |
| Asns | asparagine synthetase | 0.06072 | 0.2614 | 1.886 | 0.9152 | 1.988 | 0.001007 | 0.03118 | 4.275 | 2.096 | 3.847 | 0.05817 | 0.1976 | 0.4953 | -1.014 | -2.01 | 6.697e-05 | 0.001307 | 0.1733 | -2.528 | -5.013 |  |
| Kcnb1 | potassium voltage gated channe | 0.0004719 | 0.01422 | 0.4499 | -1.152 | -4.172 | 0.2204 | 0.582 | 0.7508 | -0.4135 | -1.265 | 0.003924 | 0.02859 | 0.5047 | -0.9866 | -3.26 | 0.0006053 | 0.006985 | 0.4262 | -1.23 | -4.065 |  |
| Gm5808 | predicted gene 5808 | 0.9524 | 0.9828 | 1.007 | 0.0094 | 0.06041 | 0.002728 | 0.05901 | 1.547 | 0.6293 | 3.418 | 1.056e-06 | 5.169e-05 | 0.4423 | -1.177 | -6.904 | 0.0006935 | 0.007729 | 0.6229 | -0.683 | -4.007 |  |
| Pir | pirin | 0.002409 | 0.03861 | 1.652 | 0.7244 | 3.472 | 0.6319 | 0.8567 | 0.9201 | -0.1201 | -0.4866 | 0.005896 | 0.03854 | 1.629 | 0.7042 | 3.081 | 1.167e-07 | 9.459e-06 | 3.553 | 1.829 | 8.004 |  |
| Mertk | MER proto-oncogene tyrosine ki | 3.323e-05 | 0.002821 | 2.057 | 1.04 | 5.32 | 0.002774 | 0.05966 | 1.728 | 0.7892 | 3.411 | 0.0503 | 0.1792 | 1.362 | 0.4462 | 2.083 | 0.1673 | 0.383 | 1.237 | 0.3069 | 1.433 |  |
| Eng | endoglin | 0.1995 | 0.4939 | 1.14 | 0.189 | 1.327 | 0.01609 | 0.1701 | 0.7355 | -0.4432 | -2.629 | 9.07e-10 | 2.591e-07 | 3.206 | 1.681 | 10.77 | 0.182 | 0.4025 | 1.161 | 0.2158 | 1.383 |  |
| Trim25 | tripartite motif-containing 25 | 3.297e-05 | 0.002808 | 2.006 | 1.005 | 5.323 | 0.002329 | 0.0532 | 1.715 | 0.7786 | 3.487 | 0.1836 | 0.4142 | 1.218 | 0.2848 | 1.378 | 0.07164 | 0.225 | 1.313 | 0.3933 | 1.902 |  |
| Gpd1l | glycerol-3-phosphate dehydroge | 0.487 | 0.7522 | 0.9206 | -0.1194 | -0.7082 | 0.01053 | 0.1351 | 1.477 | 0.5627 | 2.822 | 8.447e-08 | 7.544e-06 | 0.3514 | -1.509 | -8.172 | 0.008621 | 0.05034 | 0.6889 | -0.5376 | -2.912 |  |
| Gm14964 | predicted gene 14964 | 5.701e-06 | 0.0008729 | 2.592 | 1.374 | 6.112 | 0.0002449 | 0.01111 | 2.273 | 1.184 | 4.453 | 0.03164 | 0.1304 | 1.484 | 0.5691 | 2.311 | 0.9988 | 0.9993 | 0.9997 | -0.000366 | -0.001486 |  |
| Abcb1a | ATP-binding cassette, sub-fami | 0.01652 | 0.1238 | 1.319 | 0.3994 | 2.617 | 0.3436 | 0.6871 | 0.8857 | -0.1752 | -0.97 | 1.124e-06 | 5.395e-05 | 2.218 | 1.149 | 6.874 | 0.002618 | 0.02099 | 1.489 | 0.5744 | 3.436 |  |
| Stmn2 | stathmin-like 2 | 0.00467 | 0.05812 | 1.644 | 0.717 | 3.184 | 0.2423 | 0.6018 | 1.249 | 0.3211 | 1.205 | 9.974e-07 | 4.963e-05 | 3.271 | 1.71 | 6.931 | 0.04703 | 0.169 | 0.6963 | -0.5222 | -2.117 |  |
| Man2a1 | mannosidase 2, alpha 1 | 5.835e-05 | 0.003964 | 1.848 | 0.8859 | 5.073 | 7.17e-05 | 0.004669 | 2.041 | 1.03 | 4.983 | 0.5075 | 0.7354 | 0.9144 | -0.1291 | -0.6749 | 0.1299 | 0.3263 | 0.811 | -0.3022 | -1.58 |  |
| Suv420h2 | NA | 0.4575 | 0.733 | 0.9157 | -0.1271 | -0.7577 | 0.001715 | 0.04402 | 0.6079 | -0.7181 | -3.619 | 1.165e-06 | 5.571e-05 | 2.394 | 1.26 | 6.857 | 0.01121 | 0.06133 | 1.427 | 0.5134 | 2.794 |  |
| Clec9a | C-type lectin domain family 9, | 0.0001831 | 0.00801 | 2.478 | 1.309 | 4.578 | 0.0001881 | 0.009131 | 2.918 | 1.545 | 4.566 | 0.01498 | 0.07646 | 0.5611 | -0.8338 | -2.662 | 0.6914 | 0.8447 | 1.091 | 0.1261 | 0.4027 |  |
| Fam20a | family with sequence similarit | 0.002327 | 0.03782 | 1.631 | 0.7061 | 3.487 | 0.5039 | 0.7889 | 0.8931 | -0.1631 | -0.6806 | 2.539e-09 | 5.397e-07 | 4.754 | 2.249 | 10.14 | 0.1218 | 0.3136 | 1.282 | 0.3585 | 1.616 |  |
| Paqr9 | progestin and adipoQ receptor | 0.0005706 | 0.01597 | 0.4575 | -1.128 | -4.09 | 0.549 | 0.8135 | 0.8712 | -0.199 | -0.6096 | 1.218e-06 | 5.752e-05 | 0.239 | -2.065 | -6.835 | 0.02674 | 0.1143 | 0.606 | -0.7225 | -2.391 |  |
| Cacna2d4 | calcium channel, voltage-depen | 0.0005735 | 0.016 | 0.5964 | -0.7456 | -4.088 | 0.2418 | 0.6015 | 0.8349 | -0.2603 | -1.206 | 0.7713 | 0.8958 | 1.042 | 0.05885 | 0.2946 | 2.662e-07 | 1.716e-05 | 0.35 | -1.515 | -7.582 |  |
| Gnpat | glyceronephosphate O-acyltrans | 0.01477 | 0.1154 | 0.7948 | -0.3313 | -2.668 | 0.3424 | 0.6861 | 1.104 | 0.1429 | 0.9726 | 3.417e-07 | 2.192e-05 | 0.4951 | -1.014 | -7.457 | 0.007046 | 0.04361 | 0.7535 | -0.4084 | -3.002 |  |
| Hbb-bs | hemoglobin, beta adult s chain | 0.05584 | 0.2501 | 0.6828 | -0.5504 | -2.03 | 0.0008811 | 0.02798 | 0.4197 | -1.252 | -3.905 | 0.002094 | 0.01791 | 2.069 | 1.049 | 3.533 | 0.00197 | 0.01696 | 2.08 | 1.057 | 3.559 |  |
| Fign | fidgetin | 0.05014 | 0.2356 | 0.704 | -0.5064 | -2.085 | 0.1478 | 0.4983 | 1.35 | 0.4328 | 1.506 | 0.01751 | 0.08576 | 0.6202 | -0.6892 | -2.59 | 1.179e-07 | 9.509e-06 | 0.2287 | -2.128 | -7.998 |  |
| Cd93 | CD93 antigen | 0.1311 | 0.397 | 0.8338 | -0.2623 | -1.574 | 0.07346 | 0.371 | 1.294 | 0.3724 | 1.889 | 0.002733 | 0.02186 | 0.649 | -0.6236 | -3.417 | 8.369e-07 | 4.221e-05 | 0.4117 | -1.28 | -7.016 |  |
| Aurka | aurora kinase A | 0.0003103 | 0.011 | 0.6223 | -0.6843 | -4.351 | 0.9386 | 0.9799 | 0.99 | -0.01452 | -0.07804 | 0.05004 | 0.1786 | 0.7795 | -0.3593 | -2.086 | 6.579e-09 | 1.071e-06 | 0.3186 | -1.65 | -9.577 |  |
| Simc1 | SUMO-interacting motifs contai | 0.09786 | 0.3377 | 1.259 | 0.3327 | 1.737 | 0.1157 | 0.4493 | 0.7723 | -0.3727 | -1.645 | 3.981e-05 | 0.0008421 | 2.143 | 1.1 | 5.24 | 9.869e-05 | 0.001744 | 2.023 | 1.017 | 4.844 |  |
| Tbcel | tubulin folding cofactor E-lik | 0.2236 | 0.5232 | 1.173 | 0.2303 | 1.256 | 0.04511 | 0.2959 | 0.7251 | -0.4638 | -2.137 | 0.001082 | 0.01082 | 1.701 | 0.7667 | 3.817 | 2.226e-06 | 9.087e-05 | 2.489 | 1.316 | 6.549 |  |
| Smim8 | small integral membrane protei | 0.5332 | 0.7837 | 1.073 | 0.1019 | 0.6341 | 0.001937 | 0.04731 | 1.6 | 0.6779 | 3.566 | 0.00297 | 0.02327 | 0.662 | -0.5951 | -3.381 | 2.818e-06 | 0.0001073 | 0.4559 | -1.133 | -6.438 |  |
| Mat2a | methionine adenosyltransferase | 0.2016 | 0.4965 | 0.8825 | -0.1804 | -1.32 | 0.0439 | 0.2916 | 1.273 | 0.3477 | 2.151 | 0.003732 | 0.02751 | 0.7115 | -0.4912 | -3.282 | 4.647e-07 | 2.655e-05 | 0.4687 | -1.093 | -7.304 |  |
| Rin3 | Ras and Rab interactor 3 | 0.2039 | 0.4991 | 1.166 | 0.2221 | 1.313 | 0.05395 | 0.3212 | 0.7527 | -0.4098 | -2.048 | 0.0003026 | 0.004058 | 1.751 | 0.8081 | 4.362 | 9.678e-06 | 0.0002861 | 2.125 | 1.088 | 5.871 |  |
| 2200002D01Rik | RIKEN cDNA 2200002D01 gene | 0.9733 | 0.9904 | 1.005 | 0.00714 | 0.03394 | 0.003562 | 0.07004 | 0.5657 | -0.8219 | -3.302 | 1.234e-05 | 0.0003462 | 2.51 | 1.327 | 5.761 | 6.4e-05 | 0.001261 | 2.234 | 1.16 | 5.033 |  |
| Mir677 | microRNA 677 | 0.1706 | 0.4546 | 1.226 | 0.2936 | 1.422 | 0.001344 | 0.03754 | 1.879 | 0.9099 | 3.723 | 0.1654 | 0.3888 | 0.7979 | -0.3258 | -1.44 | 1.511e-06 | 6.785e-05 | 0.3479 | -1.523 | -6.732 |  |
| Ska2 | spindle and kinetochore associ | 0.0004971 | 0.01462 | 0.5972 | -0.7436 | -4.149 | 0.4111 | 0.7345 | 0.8839 | -0.178 | -0.8396 | 0.3459 | 0.6004 | 0.8769 | -0.1895 | -0.9654 | 2.204e-07 | 1.516e-05 | 0.3518 | -1.507 | -7.678 |  |
| Glrx3 | glutaredoxin 3 | 0.1953 | 0.4884 | 1.158 | 0.2113 | 1.34 | 0.001387 | 0.03821 | 1.616 | 0.692 | 3.71 | 0.1451 | 0.359 | 0.834 | -0.2619 | -1.516 | 1.219e-06 | 5.787e-05 | 0.4413 | -1.18 | -6.835 |  |
| Edn1 | endothelin 1 | 0.0006688 | 0.01766 | 1.774 | 0.827 | 4.022 | 0.6936 | 0.8854 | 1.07 | 0.09723 | 0.3997 | 1.518e-06 | 6.723e-05 | 2.859 | 1.516 | 6.73 | 0.007229 | 0.04435 | 1.595 | 0.6736 | 2.991 |  |
| Aox1 | aldehyde oxidase 1 | 0.1381 | 0.4078 | 1.216 | 0.2827 | 1.545 | 0.07815 | 0.3829 | 0.7568 | -0.4021 | -1.857 | 0.001403 | 0.01325 | 1.673 | 0.7428 | 3.705 | 2.259e-06 | 9.193e-05 | 2.482 | 1.312 | 6.542 |  |
| Sypl2 | synaptophysin-like 2 | 0.0005398 | 0.01543 | 0.5968 | -0.7447 | -4.114 | 0.4424 | 0.7543 | 0.8902 | -0.1679 | -0.7838 | 0.1847 | 0.4156 | 0.828 | -0.2724 | -1.374 | 4.207e-07 | 2.476e-05 | 0.364 | -1.458 | -7.354 |  |
| D630039A03Rik | RIKEN cDNA D630039A03 gene | 0.08277 | 0.3075 | 0.7572 | -0.4012 | -1.826 | 0.001124 | 0.03367 | 0.5043 | -0.9877 | -3.8 | 0.01092 | 0.06067 | 1.597 | 0.6752 | 2.806 | 0.000216 | 0.003176 | 2.121 | 1.084 | 4.507 |  |
| Fam45a | family with sequence similarit | 0.01651 | 0.1238 | 0.7692 | -0.3786 | -2.617 | 0.3581 | 0.6978 | 1.118 | 0.161 | 0.9407 | 9.813e-07 | 4.921e-05 | 0.4666 | -1.1 | -6.939 | 0.003778 | 0.02769 | 0.6977 | -0.5192 | -3.277 |  |
| Adgrl4 | adhesion G protein-coupled rec | 0.0005526 | 0.01569 | 1.5 | 0.5853 | 4.104 | 0.4382 | 0.7514 | 1.097 | 0.1335 | 0.791 | 5.797e-07 | 3.263e-05 | 2.18 | 1.124 | 7.196 | 0.1542 | 0.3643 | 1.174 | 0.2314 | 1.481 |  |
| Magt1 | magnesium transporter 1 | 0.0273 | 0.1662 | 0.8062 | -0.3108 | -2.382 | 0.2688 | 0.6263 | 1.129 | 0.1757 | 1.138 | 8.631e-07 | 4.476e-05 | 0.4997 | -1.001 | -7.001 | 0.004431 | 0.03099 | 0.7277 | -0.4585 | -3.207 |  |
| Parp4 | poly (ADP-ribose) polymerase f | 0.003213 | 0.04594 | 1.381 | 0.4653 | 3.347 | 0.6789 | 0.879 | 0.9532 | -0.06909 | -0.4201 | 0.0009598 | 0.009841 | 1.504 | 0.589 | 3.868 | 1.76e-06 | 7.634e-05 | 2.02 | 1.014 | 6.66 |  |
| Gm42035 | NA | 0.1384 | 0.4082 | 1.443 | 0.5292 | 1.544 | 0.001269 | 0.03648 | 2.869 | 1.521 | 3.748 | 2.538e-06 | 0.0001006 | 0.1847 | -2.437 | -6.487 | 0.168 | 0.3839 | 0.689 | -0.5374 | -1.431 |  |
| Tmem184c | transmembrane protein 184C | 0.06695 | 0.2758 | 0.7936 | -0.3336 | -1.938 | 0.1623 | 0.5178 | 1.227 | 0.2956 | 1.451 | 3.435e-06 | 0.000127 | 0.4362 | -1.197 | -6.346 | 0.00155 | 0.01411 | 0.6195 | -0.6908 | -3.662 |  |
| Kbtbd13 | kelch repeat and BTB (POZ) dom | 0.0004913 | 0.01452 | 0.5586 | -0.8402 | -4.154 | 0.3142 | 0.6644 | 0.8426 | -0.247 | -1.032 | 0.734 | 0.8771 | 0.9485 | -0.07635 | -0.3447 | 2.162e-07 | 1.491e-05 | 0.3071 | -1.703 | -7.688 |  |
| Itgb3 | integrin beta 3 | 0.0009174 | 0.02139 | 1.702 | 0.7669 | 3.887 | 0.000282 | 0.0123 | 2.035 | 1.025 | 4.392 | 0.4107 | 0.6589 | 0.8817 | -0.1816 | -0.8403 | 0.01246 | 0.06604 | 0.6627 | -0.5935 | -2.746 |  |
| Mrps6 | mitochondrial ribosomal protei | 0.000522 | 0.01508 | 0.6871 | -0.5413 | -4.129 | 0.7068 | 0.8912 | 0.9598 | -0.05919 | -0.3815 | 6.841e-08 | 6.422e-06 | 0.4384 | -1.19 | -8.283 | 0.08917 | 0.2584 | 0.837 | -0.2566 | -1.787 |  |
| Fgf6 | fibroblast growth factor 6 | 0.0004866 | 0.01446 | 0.5935 | -0.7526 | -4.159 | 0.2353 | 0.5952 | 0.8339 | -0.262 | -1.224 | 0.8358 | 0.929 | 0.9716 | -0.04162 | -0.21 | 5.588e-07 | 3.033e-05 | 0.3711 | -1.43 | -7.214 |  |
| Wnt5a | wingless-type MMTV integration | 0.0005096 | 0.01491 | 0.608 | -0.7179 | -4.139 | 0.4531 | 0.7599 | 0.8969 | -0.157 | -0.7651 | 0.4675 | 0.7047 | 0.907 | -0.1408 | -0.7408 | 9.435e-08 | 8.094e-06 | 0.3434 | -1.542 | -8.114 |  |
| Pgam1 | phosphoglycerate mutase 1 | 0.007618 | 0.07841 | 0.7742 | -0.3693 | -2.968 | 0.4507 | 0.7586 | 1.082 | 0.1133 | 0.7694 | 1.07e-07 | 8.994e-06 | 0.4674 | -1.097 | -8.049 | 0.01824 | 0.08653 | 0.7843 | -0.3505 | -2.571 |  |
| Cxxc5 | CXXC finger 5 | 0.0003733 | 0.01225 | 0.5816 | -0.7819 | -4.272 | 0.5182 | 0.7968 | 0.906 | -0.1425 | -0.6578 | 0.5955 | 0.7959 | 0.9278 | -0.1082 | -0.5394 | 1.732e-08 | 2.173e-06 | 0.2851 | -1.81 | -9.028 |  |
| D1Ertd622e | DNA segment, Chr 1, ERATO Doi | 0.0009948 | 0.02269 | 0.6025 | -0.7309 | -3.853 | 0.06192 | 0.3439 | 0.7351 | -0.444 | -1.978 | 0.438 | 0.6815 | 1.121 | 0.1645 | 0.7913 | 8.04e-05 | 0.001486 | 0.4913 | -1.025 | -4.933 |  |
| BC023105 | cDNA sequence BC023105 | 0.004952 | 0.06025 | 0.495 | -1.015 | -3.158 | 0.0005213 | 0.01924 | 0.3369 | -1.57 | -4.129 | 0.2413 | 0.4901 | 1.343 | 0.425 | 1.208 | 0.002139 | 0.01808 | 2.362 | 1.24 | 3.523 |  |
| Poldip2 | polymerase (DNA-directed), del | 0.2425 | 0.5455 | 0.8949 | -0.1602 | -1.205 | 0.04316 | 0.2897 | 1.266 | 0.3398 | 2.159 | 5.407e-07 | 3.112e-05 | 0.4819 | -1.053 | -7.23 | 0.006101 | 0.03911 | 0.7337 | -0.4467 | -3.066 |  |
| Ypel2 | yippee like 2 | 0.1282 | 0.3918 | 0.8071 | -0.3091 | -1.587 | 0.001451 | 0.03946 | 0.5546 | -0.8506 | -3.691 | 1.515e-07 | 1.164e-05 | 3.202 | 1.679 | 7.869 | 0.8006 | 0.9062 | 0.9629 | -0.05461 | -0.2559 |  |
| Ighv1-54 | immunoglobulin heavy variable | 0.03833 | 0.2025 | 2.29 | 1.196 | 2.218 | 0.0008587 | 0.02751 | 5.647 | 2.498 | 3.916 | 0.004822 | 0.03318 | 0.2732 | -1.872 | -3.17 | 0.002567 | 0.02068 | 0.2442 | -2.034 | -3.444 |  |
| Peg3 | paternally expressed 3 | 4.921e-05 | 0.003581 | 8.987 | 3.168 | 5.147 | 0.003697 | 0.07184 | 5.252 | 2.393 | 3.286 | 0.004578 | 0.03192 | 4.445 | 2.152 | 3.193 | 0.8005 | 0.9062 | 1.127 | 0.1726 | 0.256 |  |
| Myc | myelocytomatosis oncogene | 0.001701 | 0.03161 | 2.253 | 1.172 | 3.622 | 0.8809 | 0.9597 | 0.9605 | -0.05809 | -0.1518 | 7.962e-05 | 0.001434 | 3.362 | 1.749 | 4.938 | 2.343e-05 | 0.0005687 | 3.836 | 1.94 | 5.474 |  |
| Glmn | glomulin, FKBP associated prot | 0.4305 | 0.7126 | 0.9313 | -0.1027 | -0.8047 | 0.01742 | 0.1793 | 1.312 | 0.3915 | 2.592 | 0.002556 | 0.02083 | 0.716 | -0.4819 | -3.446 | 6.263e-07 | 3.315e-05 | 0.4997 | -1.001 | -7.158 |  |
| Dcun1d3 | DCN1, defective in cullin nedd | 0.02998 | 0.1756 | 1.324 | 0.4053 | 2.337 | 0.0009826 | 0.03056 | 1.731 | 0.7918 | 3.858 | 0.8162 | 0.9193 | 0.9694 | -0.04476 | -0.2356 | 9.785e-06 | 0.0002887 | 0.4618 | -1.115 | -5.866 |  |
| Wdr34 | WD repeat domain 34 | 0.1981 | 0.492 | 1.212 | 0.2776 | 1.331 | 0.001645 | 0.04306 | 1.862 | 0.8971 | 3.637 | 0.004414 | 0.03113 | 0.6017 | -0.7328 | -3.209 | 8.117e-05 | 0.001494 | 0.4582 | -1.126 | -4.929 |  |
| Sh2b2 | SH2B adaptor protein 2 | 0.1381 | 0.4078 | 1.27 | 0.3445 | 1.545 | 0.08688 | 0.3985 | 0.7194 | -0.4752 | -1.801 | 2.322e-06 | 9.31e-05 | 3.022 | 1.595 | 6.529 | 0.002079 | 0.01767 | 1.82 | 0.8639 | 3.536 |  |
| Piezo1 | piezo-type mechanosensitive io | 0.625 | 0.8382 | 1.048 | 0.06771 | 0.4965 | 0.008543 | 0.1203 | 0.7217 | -0.4706 | -2.916 | 1.657e-07 | 1.261e-05 | 2.248 | 1.169 | 7.823 | 0.0073 | 0.04469 | 1.362 | 0.4462 | 2.987 |  |
| Lrrc16a | NA | 0.5532 | 0.7962 | 1.108 | 0.1478 | 0.6031 | 0.01436 | 0.1603 | 0.5834 | -0.7774 | -2.681 | 0.0001869 | 0.002796 | 2.34 | 1.226 | 4.569 | 1.206e-05 | 0.0003428 | 2.926 | 1.549 | 5.771 |  |
| Slc17a4 | solute carrier family 17 (sodi | 0.3675 | 0.6622 | 0.8679 | -0.2044 | -0.9221 | 0.002228 | 0.05186 | 0.5287 | -0.9195 | -3.506 | 0.0002504 | 0.003499 | 2.112 | 1.079 | 4.443 | 0.0002823 | 0.003924 | 2.094 | 1.066 | 4.392 |  |
| Thoc7 | THO complex 7 | 0.3174 | 0.6185 | 0.895 | -0.1601 | -1.025 | 0.03294 | 0.2539 | 1.341 | 0.4233 | 2.291 | 0.0007931 | 0.008511 | 0.6261 | -0.6755 | -3.95 | 3.929e-06 | 0.0001407 | 0.4748 | -1.075 | -6.284 |  |
| Cry2 | cryptochrome 2 (photolyase-lik | 0.6431 | 0.8477 | 0.9467 | -0.07903 | -0.4704 | 0.021 | 0.1988 | 0.7081 | -0.498 | -2.506 | 0.006131 | 0.03974 | 0.6765 | -0.5638 | -3.064 | 3.826e-09 | 6.979e-07 | 3.533 | 1.821 | 9.894 |  |
| Lpl | lipoprotein lipase | 0.4082 | 0.6958 | 0.8681 | -0.2041 | -0.8448 | 0.002405 | 0.05437 | 0.5026 | -0.9926 | -3.473 | 0.0001266 | 0.002071 | 2.384 | 1.254 | 4.737 | 0.000439 | 0.005483 | 2.162 | 1.112 | 4.203 |  |
| Tfdp2 | transcription factor Dp 2 | 0.006557 | 0.07229 | 1.375 | 0.4593 | 3.034 | 0.408 | 0.7321 | 0.9004 | -0.1514 | -0.8451 | 0.102 | 0.2871 | 1.218 | 0.2842 | 1.714 | 1.006e-08 | 1.437e-06 | 2.924 | 1.548 | 9.334 |  |
| Oser1 | oxidative stress responsive se | 0.0005647 | 0.01589 | 1.513 | 0.5973 | 4.095 | 0.1993 | 0.562 | 1.172 | 0.2291 | 1.328 | 0.8901 | 0.9536 | 1.016 | 0.02236 | 0.1399 | 1.389e-06 | 6.369e-05 | 2.117 | 1.082 | 6.772 |  |
| Kdm7a | lysine (K)-specific demethylas | 0.00284 | 0.04252 | 1.412 | 0.4976 | 3.401 | 0.3275 | 0.6747 | 0.8865 | -0.1738 | -1.004 | 8.466e-11 | 4.633e-08 | 3.938 | 1.977 | 12.34 | 0.8204 | 0.9165 | 1.026 | 0.03688 | 0.2301 |  |
| Tcf15 | transcription factor 15 | 0.0006694 | 0.01766 | 0.6105 | -0.7118 | -4.022 | 0.235 | 0.5952 | 0.8372 | -0.2564 | -1.224 | 0.1341 | 0.3416 | 0.8107 | -0.3027 | -1.562 | 2.284e-05 | 0.0005574 | 0.4785 | -1.064 | -5.486 |  |
| Ephx2 | epoxide hydrolase 2, cytoplasm | 0.003333 | 0.04688 | 1.728 | 0.7893 | 3.331 | 0.02707 | 0.2272 | 1.59 | 0.6688 | 2.386 | 0.0007997 | 0.008544 | 2.034 | 1.024 | 3.946 | 0.01895 | 0.08906 | 0.6317 | -0.6628 | -2.553 |  |
| Tmem255b | transmembrane protein 255B | 0.02908 | 0.1727 | 0.7602 | -0.3955 | -2.351 | 0.2856 | 0.6413 | 1.163 | 0.2183 | 1.097 | 0.0003314 | 0.004353 | 0.5757 | -0.7965 | -4.323 | 1.506e-05 | 0.0004053 | 0.4847 | -1.045 | -5.672 |  |
| Got1 | glutamic-oxaloacetic transamin | 0.003436 | 0.04765 | 1.641 | 0.715 | 3.318 | 0.0004685 | 0.0178 | 2.091 | 1.064 | 4.175 | 0.2345 | 0.4813 | 0.8183 | -0.2893 | -1.226 | 0.004343 | 0.03056 | 0.5909 | -0.7591 | -3.216 |  |
| Ndufb3 | NADH:ubiquinone oxidoreductase | 0.002241 | 0.03715 | 0.6153 | -0.7006 | -3.503 | 0.6233 | 0.8522 | 1.085 | 0.118 | 0.4988 | 7.869e-09 | 1.261e-06 | 0.2372 | -2.076 | -9.474 | 0.09794 | 0.274 | 0.7682 | -0.3804 | -1.736 |  |
| Lsmem2 | leucine-rich single-pass membr | 0.001561 | 0.02994 | 0.6269 | -0.6736 | -3.659 | 0.128 | 0.4685 | 0.7868 | -0.3459 | -1.588 | 0.3763 | 0.6277 | 1.135 | 0.1825 | 0.9049 | 1.183e-05 | 0.0003373 | 0.4458 | -1.166 | -5.78 |  |
| Gna12 | guanine nucleotide binding pro | 0.2054 | 0.501 | 0.8815 | -0.182 | -1.309 | 0.04833 | 0.3052 | 1.271 | 0.346 | 2.103 | 0.005794 | 0.03803 | 0.7217 | -0.4705 | -3.089 | 4.59e-07 | 2.63e-05 | 0.4622 | -1.114 | -7.311 |  |
| Nqo2 | N-ribosyldihydronicotinamide q | 3.924e-06 | 0.0006774 | 0.4751 | -1.074 | -6.284 | 9.223e-05 | 0.005585 | 0.5051 | -0.9854 | -4.874 | 0.2237 | 0.4672 | 0.8496 | -0.2351 | -1.256 | 0.8734 | 0.9427 | 0.9793 | -0.03021 | -0.1614 |  |
| Gpihbp1 | GPI-anchored HDL-binding prote | 0.04677 | 0.2274 | 1.361 | 0.4446 | 2.119 | 0.2168 | 0.578 | 0.803 | -0.3165 | -1.275 | 2.273e-06 | 9.178e-05 | 2.833 | 1.503 | 6.539 | 0.002857 | 0.02237 | 1.718 | 0.7808 | 3.398 |  |
| Tmem189 | transmembrane protein 189 | 0.9984 | 0.9992 | 1 | 0.0003333 | 0.002065 | 0.001713 | 0.04401 | 1.615 | 0.6913 | 3.619 | 1.276e-08 | 1.844e-06 | 0.3238 | -1.627 | -9.199 | 0.02024 | 0.09338 | 0.734 | -0.4461 | -2.523 |  |
| Ptch2 | patched 2 | 0.005554 | 0.0652 | 0.5029 | -0.9916 | -3.108 | 0.5553 | 0.8169 | 1.17 | 0.2265 | 0.6 | 2.176e-07 | 1.541e-05 | 0.1554 | -2.686 | -7.684 | 0.01181 | 0.06374 | 0.5111 | -0.9683 | -2.77 |  |
| Med4 | mediator complex subunit 4 | 0.007401 | 0.07698 | 1.549 | 0.6318 | 2.98 | 0.0005722 | 0.02053 | 2.036 | 1.026 | 4.089 | 0.001089 | 0.01088 | 0.5413 | -0.8856 | -3.814 | 0.2171 | 0.4468 | 0.8145 | -0.296 | -1.275 |  |
| Cntfr | ciliary neurotrophic factor re | 0.7748 | 0.9109 | 0.9394 | -0.09021 | -0.29 | 0.006858 | 0.1057 | 2.158 | 1.109 | 3.014 | 0.0004588 | 0.005614 | 0.3722 | -1.426 | -4.184 | 3.207e-06 | 0.0001192 | 0.2217 | -2.173 | -6.378 |  |
| Tob1 | transducer of ErbB-2.1 | 0.001029 | 0.02327 | 0.5687 | -0.8143 | -3.838 | 0.06571 | 0.3524 | 0.7126 | -0.4889 | -1.947 | 0.4747 | 0.7106 | 1.125 | 0.1693 | 0.7285 | 8.113e-05 | 0.001494 | 0.452 | -1.146 | -4.929 |  |
| Derl3 | Der1-like domain family, membe | 0.0003428 | 0.01171 | 2.851 | 1.511 | 4.309 | 0.0002518 | 0.01134 | 3.587 | 1.843 | 4.441 | 0.8933 | 0.955 | 1.037 | 0.05221 | 0.1359 | 0.01302 | 0.06814 | 0.4838 | -1.047 | -2.726 |  |
| Wsb1 | WD repeat and SOCS box-contain | 0.4316 | 0.7132 | 0.8784 | -0.1871 | -0.8027 | 0.002492 | 0.05544 | 0.5164 | -0.9533 | -3.457 | 3.203e-05 | 0.0007214 | 2.571 | 1.362 | 5.336 | 0.001525 | 0.01394 | 1.914 | 0.9366 | 3.669 |  |
| Gpatch1 | G patch domain containing 1 | 0.0007859 | 0.01928 | 1.39 | 0.4751 | 3.953 | 0.9158 | 0.972 | 0.9895 | -0.01522 | -0.1071 | 0.03262 | 0.1334 | 1.233 | 0.3022 | 2.296 | 2.273e-08 | 2.683e-06 | 2.248 | 1.169 | 8.878 |  |
| Gm16418 | predicted pseudogene 16418 | 0.002268 | 0.03744 | 0.5016 | -0.9955 | -3.498 | 0.0004152 | 0.01625 | 0.3729 | -1.423 | -4.227 | 0.2031 | 0.4406 | 1.329 | 0.4102 | 1.316 | 0.01108 | 0.06072 | 1.831 | 0.8727 | 2.799 |  |
| Them4 | thioesterase superfamily membe | 0.1623 | 0.4428 | 0.835 | -0.2602 | -1.451 | 0.07994 | 0.3867 | 1.312 | 0.3914 | 1.845 | 0.0005954 | 0.006838 | 0.5744 | -0.7998 | -4.072 | 9.493e-06 | 0.000282 | 0.4491 | -1.155 | -5.879 |  |
| Mr1 | major histocompatibility compl | 0.0002727 | 0.01013 | 0.5179 | -0.9493 | -4.407 | 0.0392 | 0.277 | 0.6771 | -0.5625 | -2.207 | 0.0001043 | 0.00178 | 0.4545 | -1.138 | -4.82 | 0.9237 | 0.966 | 0.9843 | -0.02288 | -0.09697 |  |
| Chst15 | carbohydrate sulfotransferase | 0.0005964 | 0.01643 | 2.156 | 1.108 | 4.072 | 0.0002756 | 0.01214 | 2.671 | 1.418 | 4.402 | 0.6075 | 0.8026 | 0.8978 | -0.1556 | -0.5219 | 0.01743 | 0.08363 | 0.5853 | -0.7728 | -2.592 |  |
| Obp2a | odorant binding protein 2A | 0.8875 | 0.9574 | 1.018 | 0.02568 | 0.1433 | 0.003665 | 0.07148 | 1.622 | 0.6976 | 3.29 | 5.623e-05 | 0.001097 | 0.5003 | -0.9991 | -5.089 | 4.58e-05 | 0.0009693 | 0.4942 | -1.017 | -5.179 |  |
| Dhx32 | DEAH (Asp-Glu-Ala-His) box pol | 0.001298 | 0.02689 | 0.7062 | -0.5018 | -3.738 | 0.5326 | 0.8038 | 0.9325 | -0.1009 | -0.6351 | 1.431e-08 | 2.022e-06 | 0.3942 | -1.343 | -9.135 | 0.3511 | 0.5891 | 1.102 | 0.1404 | 0.9549 |  |
| Slc40a1 | solute carrier family 40 (iron | 0.0008866 | 0.02102 | 2.036 | 1.026 | 3.902 | 0.5548 | 0.8169 | 1.138 | 0.1868 | 0.6006 | 0.002331 | 0.01936 | 2.005 | 1.004 | 3.486 | 3.866e-05 | 0.0008421 | 2.853 | 1.513 | 5.253 |  |
| Zfp185 | zinc finger protein 185 | 0.003466 | 0.04785 | 0.6547 | -0.6112 | -3.314 | 0.6625 | 0.8713 | 1.069 | 0.09668 | 0.4431 | 2.751e-07 | 1.861e-05 | 0.3467 | -1.528 | -7.566 | 0.007991 | 0.0476 | 0.662 | -0.5952 | -2.946 |  |
| Clec1b | C-type lectin domain family 1, | 0.0001317 | 0.006443 | 1.839 | 0.8792 | 4.72 | 0.0001373 | 0.007357 | 2.051 | 1.036 | 4.702 | 0.1396 | 0.3505 | 0.8044 | -0.3139 | -1.538 | 0.4495 | 0.6728 | 0.8966 | -0.1574 | -0.7715 |  |
| Fntb | farnesyltransferase, CAAX box, | 0.3278 | 0.6275 | 0.9084 | -0.1386 | -1.003 | 0.009084 | 0.124 | 0.7208 | -0.4723 | -2.889 | 0.09895 | 0.282 | 0.834 | -0.2619 | -1.731 | 6.098e-08 | 5.853e-06 | 2.4 | 1.263 | 8.344 |  |
| Sctr | secretin receptor | 0.1456 | 0.4192 | 1.231 | 0.2999 | 1.514 | 0.07015 | 0.3635 | 0.7329 | -0.4483 | -1.913 | 9.06e-08 | 7.976e-06 | 3.398 | 1.765 | 8.136 | 0.03882 | 0.1481 | 1.394 | 0.4797 | 2.212 |  |
| Cited4 | Cbp/p300-interacting transacti | 0.000694 | 0.01799 | 0.4283 | -1.223 | -4.007 | 0.6013 | 0.8405 | 0.8755 | -0.1918 | -0.531 | 0.2179 | 0.4601 | 0.7446 | -0.4255 | -1.272 | 1.222e-07 | 9.79e-06 | 0.1572 | -2.669 | -7.98 |  |
| Parp3 | poly (ADP-ribose) polymerase f | 0.0007438 | 0.01869 | 0.5708 | -0.809 | -3.977 | 0.3441 | 0.6876 | 0.8507 | -0.2332 | -0.9691 | 6.677e-06 | 0.0002173 | 0.3934 | -1.346 | -6.039 | 0.1024 | 0.2822 | 0.7677 | -0.3814 | -1.712 |  |
| 1500011K16Rik | NA | 9.896e-06 | 0.001292 | 0.4889 | -1.032 | -5.861 | 0.0002856 | 0.01239 | 0.5306 | -0.9143 | -4.387 | 0.1098 | 0.3008 | 0.7995 | -0.3229 | -1.673 | 0.8732 | 0.9427 | 0.9786 | -0.0312 | -0.1617 |  |
| Mcam | melanoma cell adhesion molecul | 0.0009015 | 0.02121 | 0.5587 | -0.8399 | -3.895 | 0.5409 | 0.8082 | 0.8958 | -0.1588 | -0.6222 | 0.002687 | 0.0216 | 0.5708 | -0.809 | -3.425 | 4.119e-05 | 0.0008866 | 0.425 | -1.234 | -5.225 |  |
| Ubxn4 | UBX domain protein 4 | 0.05569 | 0.2499 | 1.176 | 0.2333 | 2.032 | 0.1532 | 0.5064 | 0.8695 | -0.2018 | -1.485 | 0.06516 | 0.213 | 1.185 | 0.2455 | 1.952 | 5.246e-08 | 5.25e-06 | 2.084 | 1.06 | 8.424 |  |
| mt-Tm | mitochondrially encoded tRNA m | 0.06098 | 0.2617 | 0.462 | -1.114 | -1.986 | 0.001165 | 0.0344 | 0.1752 | -2.513 | -3.785 | 0.0009365 | 0.00967 | 5.219 | 2.384 | 3.878 | 0.007955 | 0.04749 | 3.511 | 1.812 | 2.948 |  |
| Pfn2 | profilin 2 | 0.2822 | 0.587 | 1.169 | 0.2256 | 1.105 | 0.00207 | 0.04976 | 1.808 | 0.8545 | 3.538 | 5.684e-05 | 0.001103 | 0.4547 | -1.137 | -5.084 | 0.003175 | 0.02421 | 0.5947 | -0.7497 | -3.352 |  |
| Onecut1 | one cut domain, family member | 0.1836 | 0.4731 | 0.5366 | -0.898 | -1.378 | 0.001897 | 0.04683 | 0.1478 | -2.758 | -3.575 | 0.0009724 | 0.009922 | 6.766 | 2.758 | 3.862 | 0.0007919 | 0.008548 | 7.067 | 2.821 | 3.95 |  |
| Slc39a2 | solute carrier family 39 (zinc | 0.7017 | 0.8778 | 1.037 | 0.05292 | 0.3886 | 0.002262 | 0.05233 | 1.478 | 0.5639 | 3.499 | 0.02353 | 0.106 | 0.776 | -0.3658 | -2.452 | 1.659e-07 | 1.207e-05 | 0.4453 | -1.167 | -7.823 |  |
| Arhgap6 | Rho GTPase activating protein | 0.3637 | 0.6589 | 0.8795 | -0.1852 | -0.9295 | 0.002445 | 0.05487 | 0.5676 | -0.8172 | -3.466 | 0.000151 | 0.00237 | 2.024 | 1.017 | 4.661 | 0.0006664 | 0.007492 | 1.838 | 0.8784 | 4.024 |  |
| Itpk1 | inositol 1,3,4-triphosphate 5/ | 0.1136 | 0.366 | 0.7982 | -0.3251 | -1.655 | 0.001712 | 0.04401 | 0.5581 | -0.8414 | -3.619 | 0.108 | 0.2978 | 1.285 | 0.3621 | 1.682 | 2.143e-05 | 0.0005326 | 2.276 | 1.187 | 5.514 |  |
| Rab22a | RAB22A, member RAS oncogene fa | 0.774 | 0.9105 | 0.967 | -0.04848 | -0.2911 | 0.009122 | 0.1243 | 1.483 | 0.5688 | 2.887 | 1.03e-05 | 0.0003005 | 0.4777 | -1.066 | -5.842 | 0.0003416 | 0.00454 | 0.5799 | -0.7862 | -4.31 |  |
| Wdtc1 | WD and tetratricopeptide repea | 0.7793 | 0.9133 | 1.02 | 0.02857 | 0.2841 | 0.003559 | 0.07004 | 0.7615 | -0.393 | -3.303 | 8.39e-09 | 1.332e-06 | 2.056 | 1.04 | 9.438 | 0.05453 | 0.1871 | 1.169 | 0.225 | 2.042 |  |
| Pkdcc | protein kinase domain containi | 0.04233 | 0.2143 | 0.6427 | -0.6377 | -2.169 | 0.02065 | 0.197 | 0.5455 | -0.8743 | -2.513 | 6.509e-05 | 0.001224 | 3.07 | 1.618 | 5.025 | 0.01056 | 0.05843 | 0.5327 | -0.9086 | -2.821 |  |
| Gnb4 | guanine nucleotide binding pro | 0.01751 | 0.1281 | 0.7968 | -0.3277 | -2.59 | 0.2762 | 0.6321 | 1.123 | 0.1676 | 1.12 | 1.087e-08 | 1.616e-06 | 0.4096 | -1.288 | -9.29 | 0.168 | 0.384 | 0.8716 | -0.1983 | -1.431 |  |
| Slc2a4 | solute carrier family 2 (facil | 0.0001835 | 0.00801 | 0.6458 | -0.6309 | -4.577 | 0.8232 | 0.9395 | 1.026 | 0.03693 | 0.2264 | 1.569e-11 | 1.552e-08 | 0.2421 | -2.046 | -13.55 | 0.7796 | 0.8958 | 1.03 | 0.04284 | 0.2837 |  |
| Lin9 | lin-9 homolog (C. elegans) | 0.02298 | 0.1498 | 0.6448 | -0.6331 | -2.463 | 0.0008108 | 0.0262 | 0.4358 | -1.198 | -3.94 | 0.007509 | 0.04599 | 1.787 | 0.8373 | 2.974 | 0.006358 | 0.04031 | 1.813 | 0.8582 | 3.048 |  |
| Wdr81 | WD repeat domain 81 | 0.02358 | 0.1522 | 1.261 | 0.3344 | 2.451 | 0.2936 | 0.6492 | 0.8863 | -0.1741 | -1.079 | 0.01518 | 0.07713 | 1.317 | 0.3969 | 2.656 | 2.83e-07 | 1.792e-05 | 2.186 | 1.129 | 7.552 |  |
| Thap2 | THAP domain containing, apopto | 0.1347 | 0.4029 | 1.192 | 0.254 | 1.559 | 0.05192 | 0.3155 | 1.318 | 0.3984 | 2.067 | 0.002793 | 0.02219 | 1.524 | 0.6081 | 3.408 | 1.044e-05 | 0.000305 | 0.4858 | -1.041 | -5.837 |  |
| Slco2b1 | solute carrier organic anion t | 0.01642 | 0.1235 | 1.348 | 0.4307 | 2.62 | 0.3941 | 0.7231 | 0.8892 | -0.1694 | -0.871 | 0.002239 | 0.0188 | 1.549 | 0.631 | 3.504 | 2.506e-06 | 9.917e-05 | 2.249 | 1.169 | 6.493 |  |
| Cnp | 2',3'-cyclic nucleotide 3' pho | 0.8223 | 0.933 | 0.9658 | -0.05024 | -0.2275 | 0.003535 | 0.06979 | 0.5496 | -0.8636 | -3.306 | 0.0007362 | 0.008 | 1.949 | 0.963 | 3.981 | 5.766e-06 | 0.0001928 | 2.784 | 1.477 | 6.107 |  |
| Egln3 | egl-9 family hypoxia-inducible | 0.02772 | 0.1679 | 1.697 | 0.763 | 2.374 | 0.3205 | 0.6697 | 0.7645 | -0.3874 | -1.019 | 0.0002792 | 0.003815 | 2.924 | 1.548 | 4.397 | 2.73e-05 | 0.0006395 | 3.741 | 1.903 | 5.407 |  |
| Rfxank | regulatory factor X-associated | 0.6027 | 0.8265 | 0.9481 | -0.07687 | -0.5289 | 0.002742 | 0.05923 | 0.6655 | -0.5874 | -3.416 | 0.004685 | 0.03248 | 1.421 | 0.5067 | 3.182 | 2.819e-06 | 0.0001073 | 2.035 | 1.025 | 6.438 |  |
| Cacna1i | calcium channel, voltage-depen | 0.0004291 | 0.01338 | 1.779 | 0.8314 | 4.212 | 0.628 | 0.8544 | 1.083 | 0.1149 | 0.4921 | 0.6384 | 0.8211 | 1.074 | 0.1032 | 0.4773 | 8.009e-09 | 1.242e-06 | 4.131 | 2.046 | 9.464 |  |
| Trib2 | tribbles pseudokinase 2 | 0.03807 | 0.2018 | 1.294 | 0.3719 | 2.221 | 0.001449 | 0.03946 | 1.66 | 0.7312 | 3.691 | 0.9338 | 0.9729 | 1.011 | 0.01542 | 0.08408 | 7.916e-06 | 0.0002465 | 0.4686 | -1.093 | -5.962 |  |
| Kitl | kit ligand | 0.007907 | 0.08035 | 1.327 | 0.4077 | 2.951 | 0.2667 | 0.6241 | 1.138 | 0.1868 | 1.143 | 1.929e-06 | 8.083e-05 | 2.002 | 1.001 | 6.616 | 0.03682 | 0.1427 | 0.7908 | -0.3386 | -2.237 |  |
| Dhrs7b | dehydrogenase/reductase (SDR f | 0.6636 | 0.8587 | 0.9578 | -0.06217 | -0.4415 | 0.000786 | 0.02563 | 0.6335 | -0.6586 | -3.953 | 9.59e-10 | 2.624e-07 | 3.151 | 1.656 | 10.74 | 0.3971 | 0.629 | 1.097 | 0.1335 | 0.8655 |  |
| Gm4861 | predicted gene 4861 | 0.01053 | 0.09536 | 0.6429 | -0.6373 | -2.822 | 0.1999 | 0.5623 | 1.278 | 0.3542 | 1.326 | 0.9797 | 0.9931 | 0.9956 | -0.006378 | -0.02578 | 1.382e-10 | 4.805e-08 | 0.1278 | -2.968 | -12 |  |
| Filip1l | filamin A interacting protein | 0.0008344 | 0.02014 | 0.5999 | -0.7372 | -3.928 | 0.467 | 0.7685 | 0.8921 | -0.1647 | -0.7415 | 0.7213 | 0.8706 | 1.053 | 0.07439 | 0.3618 | 3.055e-08 | 3.417e-06 | 0.2888 | -1.792 | -8.716 |  |
| Olfml1 | olfactomedin-like 1 | 0.0004251 | 0.0133 | 0.4772 | -1.067 | -4.216 | 0.01086 | 0.1372 | 0.5582 | -0.8411 | -2.808 | 0.002878 | 0.02268 | 0.5207 | -0.9413 | -3.395 | 0.3543 | 0.5916 | 1.2 | 0.2629 | 0.9483 |  |
| Ptprd | protein tyrosine phosphatase, | 0.02185 | 0.1459 | 0.7703 | -0.3765 | -2.487 | 0.3638 | 0.7014 | 1.122 | 0.1665 | 0.9293 | 0.001151 | 0.01137 | 0.6468 | -0.6285 | -3.79 | 6.791e-06 | 0.0002192 | 0.4999 | -1 | -6.032 |  |
| Tmem50a | transmembrane protein 50A | 0.0364 | 0.1965 | 1.249 | 0.3206 | 2.243 | 0.2346 | 0.5951 | 0.8662 | -0.2073 | -1.225 | 0.008184 | 0.04902 | 1.375 | 0.4597 | 2.936 | 6.007e-07 | 3.226e-05 | 2.18 | 1.124 | 7.178 |  |
| Trappc6b | trafficking protein particle c | 0.3213 | 0.6221 | 0.8831 | -0.1794 | -1.017 | 0.0421 | 0.2868 | 1.369 | 0.4533 | 2.172 | 9.62e-06 | 0.0002835 | 0.4553 | -1.135 | -5.873 | 0.0007945 | 0.008573 | 0.5892 | -0.7631 | -3.949 |  |
| Cptp | ceramide-1-phosphate transfer | 0.04119 | 0.2108 | 0.7588 | -0.3983 | -2.182 | 0.2564 | 0.616 | 1.191 | 0.2523 | 1.168 | 0.0003132 | 0.004164 | 0.5475 | -0.8691 | -4.347 | 2.673e-05 | 0.0006292 | 0.4721 | -1.083 | -5.416 |  |
| Cdsn | corneodesmosin | 0.001939 | 0.03432 | 0.4371 | -1.194 | -3.566 | 0.8159 | 0.9364 | 1.067 | 0.09345 | 0.2359 | 1.078e-07 | 9.031e-06 | 0.1293 | -2.951 | -8.045 | 0.02195 | 0.09893 | 0.5316 | -0.9115 | -2.485 |  |
| Gm8242 | predicted gene 8242 | 0.9151 | 0.9693 | 0.9879 | -0.0175 | -0.1079 | 0.005452 | 0.09177 | 1.513 | 0.5979 | 3.116 | 1.943e-06 | 8.102e-05 | 0.4429 | -1.175 | -6.613 | 0.001181 | 0.01154 | 0.6279 | -0.6713 | -3.779 |  |
| Sema3g | sema domain, immunoglobulin do | 0.05518 | 0.2485 | 1.23 | 0.2981 | 2.036 | 0.2001 | 0.5623 | 0.8529 | -0.2296 | -1.325 | 9.234e-07 | 4.722e-05 | 2.17 | 1.118 | 6.969 | 0.008811 | 0.05116 | 1.381 | 0.4655 | 2.903 |  |
| Gypc | glycophorin C | 0.0008744 | 0.02077 | 0.5345 | -0.9038 | -3.908 | 0.487 | 0.7791 | 0.8743 | -0.1938 | -0.7083 | 1.567e-06 | 6.866e-05 | 0.3075 | -1.701 | -6.715 | 0.1062 | 0.2886 | 0.743 | -0.4286 | -1.692 |  |
| Cdc14b | CDC14 cell division cycle 14B | 0.2327 | 0.534 | 1.196 | 0.2583 | 1.231 | 0.06651 | 0.3545 | 0.716 | -0.482 | -1.941 | 3.361e-05 | 0.0007483 | 2.332 | 1.222 | 5.315 | 0.0003145 | 0.004271 | 1.999 | 0.999 | 4.345 |  |
| Impa2 | inositol (myo)-1(or 4)-monopho | 0.1948 | 0.488 | 1.191 | 0.2521 | 1.342 | 0.001903 | 0.04684 | 1.735 | 0.7947 | 3.574 | 6.239e-05 | 0.001189 | 0.4869 | -1.038 | -5.044 | 0.009622 | 0.05452 | 0.6646 | -0.5894 | -2.863 |  |
| Slc46a3 | solute carrier family 46, memb | 0.7169 | 0.8851 | 0.9539 | -0.06807 | -0.3677 | 0.002669 | 0.05817 | 0.5943 | -0.7507 | -3.428 | 1.596e-07 | 1.222e-05 | 3.011 | 1.59 | 7.842 | 0.03531 | 0.1386 | 1.373 | 0.4578 | 2.258 |  |
| Foxp1 | forkhead box P1 | 0.0006108 | 0.01664 | 2.152 | 1.106 | 4.061 | 0.141 | 0.487 | 1.408 | 0.4939 | 1.533 | 0.02486 | 0.1104 | 1.651 | 0.7237 | 2.426 | 0.001326 | 0.01253 | 2.162 | 1.112 | 3.729 |  |
| Sesn2 | sestrin 2 | 0.0003292 | 0.0114 | 1.943 | 0.9584 | 4.326 | 0.0251 | 0.2181 | 1.553 | 0.6348 | 2.421 | 0.0003583 | 0.004632 | 2.058 | 1.041 | 4.29 | 0.7753 | 0.8932 | 0.9525 | -0.07022 | -0.2893 |  |
| Col5a3 | collagen, type V, alpha 3 | 6.333e-05 | 0.004198 | 0.4937 | -1.018 | -5.037 | 0.002313 | 0.05299 | 0.5607 | -0.8347 | -3.49 | 0.01546 | 0.07821 | 0.6661 | -0.5863 | -2.647 | 0.8743 | 0.9432 | 1.025 | 0.0355 | 0.1603 |  |
| Pomt1 | protein-O-mannosyltransferase | 0.0006455 | 0.01724 | 0.6227 | -0.6835 | -4.038 | 0.08559 | 0.3963 | 0.778 | -0.3622 | -1.809 | 2.528e-05 | 0.0005997 | 0.4969 | -1.009 | -5.441 | 0.81 | 0.9114 | 1.032 | 0.04517 | 0.2436 |  |
| Mir193a | microRNA 193a | 0.03518 | 0.1927 | 0.6253 | -0.6774 | -2.26 | 0.263 | 0.6206 | 1.327 | 0.4085 | 1.152 | 0.003254 | 0.02484 | 0.4674 | -1.097 | -3.342 | 2.09e-06 | 8.735e-05 | 0.2237 | -2.16 | -6.579 |  |
| Gm24707 | predicted gene, 24707 | 0.0009213 | 0.02143 | 2.424 | 1.278 | 3.885 | 0.4222 | 0.7419 | 1.247 | 0.3188 | 0.8195 | 0.1666 | 0.3905 | 1.431 | 0.5171 | 1.435 | 2.222e-06 | 9.087e-05 | 5.131 | 2.359 | 6.55 |  |
| Mcm4 | minichromosome maintenance com | 0.02274 | 0.1487 | 1.253 | 0.3257 | 2.468 | 0.317 | 0.6665 | 0.8949 | -0.1603 | -1.026 | 0.01367 | 0.0716 | 1.311 | 0.3909 | 2.704 | 3.979e-07 | 2.364e-05 | 2.095 | 1.067 | 7.381 |  |
| Atoh8 | atonal bHLH transcription fact | 0.001074 | 0.02387 | 1.55 | 0.6318 | 3.82 | 0.6455 | 0.8626 | 1.065 | 0.09141 | 0.4671 | 9.321e-06 | 0.0002771 | 2.095 | 1.067 | 5.888 | 0.006194 | 0.03951 | 1.469 | 0.5544 | 3.059 |  |
| Adh1 | alcohol dehydrogenase 1 (class | 0.01062 | 0.09572 | 1.317 | 0.3969 | 2.818 | 0.3451 | 0.6882 | 0.8943 | -0.1611 | -0.967 | 0.3165 | 0.5721 | 1.116 | 0.1585 | 1.027 | 4.304e-09 | 7.584e-07 | 2.859 | 1.516 | 9.825 |  |
| Chpt1 | choline phosphotransferase 1 | 0.189 | 0.4805 | 1.186 | 0.246 | 1.36 | 0.002162 | 0.05116 | 1.685 | 0.753 | 3.519 | 0.223 | 0.4666 | 0.8414 | -0.2492 | -1.258 | 2.928e-06 | 0.0001106 | 0.4141 | -1.272 | -6.42 |  |
| Tigd2 | tigger transposable element de | 0.3033 | 0.6062 | 1.14 | 0.1896 | 1.057 | 0.03884 | 0.2757 | 0.7222 | -0.4695 | -2.211 | 3.976e-07 | 2.464e-05 | 2.734 | 1.451 | 7.382 | 0.01448 | 0.07339 | 1.44 | 0.5264 | 2.678 |  |
| Plvap | plasmalemma vesicle associated | 0.0009022 | 0.02121 | 1.891 | 0.9189 | 3.894 | 0.4138 | 0.7361 | 1.175 | 0.233 | 0.8346 | 1.906e-05 | 0.000477 | 2.711 | 1.439 | 5.566 | 0.02656 | 0.1138 | 1.536 | 0.619 | 2.395 |  |
| Slc6a8 | solute carrier family 6 (neuro | 0.07042 | 0.2831 | 1.231 | 0.3004 | 1.911 | 0.001656 | 0.04313 | 1.597 | 0.6757 | 3.634 | 0.9772 | 0.9921 | 1.003 | 0.004984 | 0.02895 | 2.131e-06 | 8.815e-05 | 0.4566 | -1.131 | -6.569 |  |
| Slco1a4 | solute carrier organic anion t | 0.07279 | 0.2883 | 1.482 | 0.568 | 1.894 | 0.001434 | 0.03923 | 2.482 | 1.311 | 3.696 | 0.0006415 | 0.007212 | 0.3985 | -1.327 | -4.04 | 0.01241 | 0.06584 | 0.5349 | -0.9027 | -2.748 |  |
| Mal2 | mal, T cell differentiation pr | 0.0007025 | 0.01805 | 0.5161 | -0.9543 | -4.001 | 0.1595 | 0.5135 | 0.7514 | -0.4124 | -1.461 | 4.5e-06 | 0.000159 | 0.3242 | -1.625 | -6.221 | 0.9982 | 0.9991 | 0.9996 | -0.000588 | -0.002251 |  |
| Lsmem1 | leucine-rich single-pass membr | 0.002234 | 0.03707 | 0.5322 | -0.9101 | -3.505 | 0.2458 | 0.6055 | 0.7752 | -0.3674 | -1.196 | 0.3835 | 0.6342 | 1.192 | 0.2535 | 0.891 | 1.939e-06 | 8.284e-05 | 0.2714 | -1.881 | -6.614 |  |
| Ly6c2 | lymphocyte antigen 6 complex, | 0.03276 | 0.1854 | 1.436 | 0.5217 | 2.294 | 0.3091 | 0.661 | 0.8231 | -0.2808 | -1.044 | 8.664e-06 | 0.0002631 | 2.78 | 1.475 | 5.921 | 0.001283 | 0.01225 | 1.909 | 0.9325 | 3.743 |  |
| Vma21-ps | VMA21 vacuolar H+-ATPase homol | 0.1874 | 0.4782 | 0.8494 | -0.2354 | -1.365 | 0.08275 | 0.3921 | 1.295 | 0.3728 | 1.827 | 1.328e-05 | 0.0003641 | 0.4723 | -1.082 | -5.728 | 0.0008319 | 0.008864 | 0.5978 | -0.7423 | -3.929 |  |
| Gm14845 | predicted gene 14845 | 0.1874 | 0.4782 | 0.8494 | -0.2354 | -1.365 | 0.08275 | 0.3921 | 1.295 | 0.3728 | 1.827 | 1.328e-05 | 0.0003641 | 0.4723 | -1.082 | -5.728 | 0.0008319 | 0.008864 | 0.5978 | -0.7423 | -3.929 |  |
| Tuba8 | tubulin, alpha 8 | 0.006941 | 0.07458 | 1.813 | 0.858 | 3.009 | 0.2322 | 0.5936 | 1.334 | 0.4158 | 1.232 | 4.441e-06 | 0.0001572 | 3.851 | 1.945 | 6.227 | 0.06173 | 0.2036 | 0.6514 | -0.6183 | -1.979 |  |
| Gm23462 | predicted gene, 23462 | 0.001312 | 0.02706 | 2.239 | 1.163 | 3.734 | 0.0004762 | 0.01804 | 2.9 | 1.536 | 4.168 | 0.4956 | 0.7261 | 0.8486 | -0.2368 | -0.6941 | 0.01768 | 0.08446 | 0.5426 | -0.8821 | -2.586 |  |
| Gas2 | growth arrest specific 2 | 0.6092 | 0.8302 | 1.063 | 0.08866 | 0.5193 | 0.01491 | 0.1641 | 0.6887 | -0.5381 | -2.664 | 1.81e-06 | 7.656e-05 | 2.367 | 1.243 | 6.647 | 0.002792 | 0.02199 | 1.555 | 0.6374 | 3.408 |  |
| Cdh4 | cadherin 4 | 0.000655 | 0.0174 | 0.5895 | -0.7624 | -4.031 | 0.8295 | 0.9417 | 0.9667 | -0.04883 | -0.2182 | 0.2295 | 0.4749 | 0.8369 | -0.2568 | -1.24 | 1.54e-08 | 1.998e-06 | 0.271 | -1.884 | -9.094 |  |
| Irf5 | interferon regulatory factor 5 | 0.9373 | 0.9779 | 0.9828 | -0.02504 | -0.07962 | 0.004179 | 0.0782 | 0.4345 | -1.203 | -3.232 | 0.0008443 | 0.008884 | 2.551 | 1.351 | 3.923 | 3.526e-06 | 0.0001279 | 4.537 | 2.182 | 6.334 |  |
| Ak3l2-ps | adenylate kinase 3-like 2, pse | 0.01692 | 0.1257 | 1.42 | 0.506 | 2.606 | 0.6813 | 0.8803 | 1.069 | 0.09575 | 0.4167 | 2.668e-08 | 3.191e-06 | 3.654 | 1.87 | 8.79 | 0.007027 | 0.04353 | 0.6422 | -0.6389 | -3.003 |  |
| Chd2 | chromodomain helicase DNA bind | 0.1017 | 0.3444 | 1.181 | 0.2402 | 1.715 | 0.09677 | 0.416 | 0.8186 | -0.2887 | -1.743 | 2.527e-08 | 3.08e-06 | 2.554 | 1.353 | 8.82 | 0.1429 | 0.3466 | 1.176 | 0.2339 | 1.525 |  |
| Atp2a3 | ATPase, Ca++ transporting, ubi | 0.0007321 | 0.01852 | 2.414 | 1.272 | 3.984 | 0.2073 | 0.5697 | 1.407 | 0.4922 | 1.303 | 0.001884 | 0.01652 | 2.38 | 1.251 | 3.578 | 0.005542 | 0.03648 | 2.124 | 1.087 | 3.109 |  |
| Pole | polymerase (DNA directed), eps | 0.04513 | 0.2218 | 1.247 | 0.3185 | 2.137 | 0.3232 | 0.6716 | 0.8836 | -0.1786 | -1.013 | 0.07171 | 0.2271 | 0.8064 | -0.3104 | -1.902 | 5.952e-10 | 1.612e-07 | 3.486 | 1.802 | 11.04 |  |
| Igkv19-93 | immunoglobulin kappa chain var | 6.299e-05 | 0.004187 | 7.723 | 2.949 | 5.039 | 0.002245 | 0.05211 | 5.371 | 2.425 | 3.503 | 0.2038 | 0.4415 | 1.793 | 0.8422 | 1.314 | 0.2687 | 0.5075 | 1.658 | 0.7293 | 1.138 |  |
| Lurap1l | leucine rich adaptor protein 1 | 0.02462 | 0.1566 | 0.7675 | -0.3818 | -2.431 | 0.3613 | 0.6998 | 1.128 | 0.1736 | 0.9343 | 0.001375 | 0.01308 | 0.6422 | -0.639 | -3.714 | 7.771e-06 | 0.0002438 | 0.4906 | -1.027 | -5.97 |  |
| S1pr1 | sphingosine-1-phosphate recept | 0.07738 | 0.298 | 1.401 | 0.4865 | 1.862 | 0.188 | 0.5497 | 0.7467 | -0.4215 | -1.363 | 1.995e-05 | 0.0004926 | 3.005 | 1.587 | 5.546 | 0.0007544 | 0.00823 | 2.199 | 1.137 | 3.971 |  |
| Dclre1c | DNA cross-link repair 1C | 0.01563 | 0.1198 | 1.424 | 0.5095 | 2.643 | 0.4602 | 0.7645 | 0.8877 | -0.1718 | -0.7531 | 0.0001806 | 0.002731 | 1.956 | 0.9681 | 4.584 | 5.564e-05 | 0.001123 | 2.108 | 1.076 | 5.094 |  |
| Gngt2 | guanine nucleotide binding pro | 0.01523 | 0.1178 | 1.447 | 0.5332 | 2.654 | 0.4346 | 0.7494 | 0.8769 | -0.1895 | -0.7974 | 9.814e-07 | 4.921e-05 | 2.882 | 1.527 | 6.939 | 0.008589 | 0.05019 | 1.56 | 0.6412 | 2.914 |  |
| Ceacam1 | carcinoembryonic antigen-relat | 0.001121 | 0.02434 | 1.856 | 0.8922 | 3.801 | 0.06175 | 0.3434 | 1.464 | 0.5496 | 1.979 | 0.0001172 | 0.001951 | 2.34 | 1.226 | 4.77 | 0.4487 | 0.6721 | 0.8713 | -0.1987 | -0.7728 |  |
| Pim1 | proviral integration site 1 | 0.07284 | 0.2883 | 0.7076 | -0.4991 | -1.894 | 0.001653 | 0.04312 | 0.4558 | -1.133 | -3.635 | 3.485e-05 | 0.0007666 | 2.887 | 1.53 | 5.299 | 0.2109 | 0.4392 | 1.295 | 0.3731 | 1.292 |  |
| Gm25321 | predicted gene, 25321 | 0.07904 | 0.3004 | 0.7582 | -0.3994 | -1.851 | 0.00167 | 0.04343 | 0.526 | -0.9268 | -3.63 | 0.0001132 | 0.001897 | 2.19 | 1.131 | 4.785 | 0.07067 | 0.2231 | 1.367 | 0.4513 | 1.909 |  |
| E130308A19Rik | RIKEN cDNA E130308A19 gene | 0.0008016 | 0.01956 | 0.6592 | -0.6012 | -3.945 | 0.4238 | 0.7431 | 0.903 | -0.1472 | -0.8166 | 0.8241 | 0.923 | 0.9743 | -0.03759 | -0.2252 | 1.34e-07 | 1.048e-05 | 0.3994 | -1.324 | -7.932 |  |
| Als2cr12 | NA | 0.01407 | 0.1123 | 0.612 | -0.7084 | -2.691 | 0.4556 | 0.7611 | 1.179 | 0.237 | 0.7609 | 1.435e-06 | 6.416e-05 | 0.259 | -1.949 | -6.757 | 0.005952 | 0.03838 | 0.5405 | -0.8875 | -3.077 |  |
| Rassf6 | Ras association (RalGDS/AF-6) | 0.01941 | 0.1359 | 1.43 | 0.5164 | 2.542 | 0.3026 | 0.6563 | 0.8384 | -0.2543 | -1.058 | 1.526e-08 | 2.122e-06 | 4.069 | 2.025 | 9.099 | 0.2112 | 0.4396 | 1.22 | 0.2874 | 1.292 |  |
| Lepr | leptin receptor | 0.0004946 | 0.01457 | 2.989 | 1.58 | 4.152 | 0.0003174 | 0.01333 | 3.877 | 1.955 | 4.341 | 0.06922 | 0.2221 | 0.5742 | -0.8005 | -1.92 | 0.4455 | 0.6695 | 0.7986 | -0.3244 | -0.7782 |  |
| 5430403G16Rik | RIKEN cDNA 5430403G16 gene | 0.003502 | 0.04813 | 0.5866 | -0.7696 | -3.31 | 0.0005954 | 0.0211 | 0.46 | -1.12 | -4.072 | 0.1051 | 0.2928 | 1.35 | 0.4324 | 1.698 | 0.02445 | 0.1071 | 1.537 | 0.6199 | 2.434 |  |
| Nudt2 | nudix (nucleoside diphosphate | 0.0002935 | 0.01059 | 0.5538 | -0.8527 | -4.375 | 0.0002742 | 0.0121 | 0.4946 | -1.016 | -4.404 | 0.06608 | 0.2153 | 1.333 | 0.4151 | 1.944 | 0.7337 | 0.8701 | 1.052 | 0.07366 | 0.345 |  |
| Zscan26 | zinc finger and SCAN domain co | 0.001077 | 0.02388 | 1.8 | 0.8477 | 3.819 | 0.3526 | 0.6941 | 1.189 | 0.25 | 0.9517 | 0.03385 | 0.1369 | 1.468 | 0.554 | 2.278 | 5.539e-05 | 0.001118 | 2.361 | 1.239 | 5.096 |  |
| Gm4841 | predicted gene 4841 | 0.00111 | 0.02419 | 0.4404 | -1.183 | -3.806 | 0.007534 | 0.112 | 0.4686 | -1.093 | -2.973 | 0.09556 | 0.2755 | 1.511 | 0.5958 | 1.749 | 0.01952 | 0.09087 | 0.5491 | -0.8649 | -2.54 |  |
| Nepro | nucleolus and neural progenito | 0.00118 | 0.02534 | 1.687 | 0.7543 | 3.779 | 0.5176 | 0.7963 | 1.114 | 0.1556 | 0.6588 | 0.003007 | 0.02347 | 1.668 | 0.7381 | 3.376 | 9.978e-05 | 0.001759 | 2.082 | 1.058 | 4.84 |  |
| Mir122 | microRNA 122 | 0.05158 | 0.239 | 0.6085 | -0.7167 | -2.071 | 0.001442 | 0.03939 | 0.3505 | -1.513 | -3.693 | 0.00882 | 0.05174 | 2.144 | 1.1 | 2.902 | 0.002881 | 0.02252 | 2.441 | 1.287 | 3.395 |  |
| Tbx3 | T-box 3 | 0.007804 | 0.07961 | 1.52 | 0.6036 | 2.957 | 0.2319 | 0.5932 | 1.229 | 0.2978 | 1.233 | 5.802e-06 | 0.0001938 | 2.576 | 1.365 | 6.104 | 0.05916 | 0.1977 | 0.7333 | -0.4475 | -2.001 |  |
| Gm8989 | predicted gene 8989 | 0.1641 | 0.4453 | 0.7548 | -0.4059 | -1.444 | 0.00215 | 0.05105 | 0.4442 | -1.171 | -3.521 | 9.827e-05 | 0.001702 | 2.813 | 1.492 | 4.846 | 0.01685 | 0.0817 | 1.744 | 0.8028 | 2.608 |  |
| Kcnc1 | potassium voltage gated channe | 0.003258 | 0.04626 | 0.6866 | -0.5424 | -3.341 | 0.3388 | 0.6836 | 0.8777 | -0.1882 | -0.98 | 0.25 | 0.5 | 1.157 | 0.2107 | 1.185 | 6.243e-07 | 3.311e-05 | 0.4137 | -1.273 | -7.159 |  |
| Tmem79 | transmembrane protein 79 | 0.004381 | 0.05552 | 0.7248 | -0.4643 | -3.212 | 0.4489 | 0.758 | 0.9125 | -0.1321 | -0.7724 | 1.621e-07 | 1.238e-05 | 0.4232 | -1.241 | -7.834 | 0.1389 | 0.3407 | 1.184 | 0.2441 | 1.541 |  |
| Tmie | transmembrane inner ear | 0.001051 | 0.02359 | 1.551 | 0.6336 | 3.829 | 0.4439 | 0.7554 | 1.112 | 0.153 | 0.7812 | 3.079e-06 | 0.0001167 | 2.234 | 1.16 | 6.397 | 0.1349 | 0.3343 | 1.216 | 0.2825 | 1.558 |  |
| Hs2st1 | heparan sulfate 2-O-sulfotrans | 0.004847 | 0.05949 | 0.7247 | -0.4646 | -3.167 | 0.6886 | 0.8834 | 1.05 | 0.07058 | 0.4067 | 0.002959 | 0.0232 | 0.6861 | -0.5436 | -3.383 | 1.958e-06 | 8.339e-05 | 0.479 | -1.062 | -6.609 |  |
| Pafah2 | platelet-activating factor ace | 0.04113 | 0.2108 | 0.7331 | -0.4479 | -2.183 | 0.2463 | 0.6058 | 1.223 | 0.29 | 1.195 | 1.718e-07 | 1.296e-05 | 0.2965 | -1.754 | -7.805 | 0.05426 | 0.1865 | 0.7272 | -0.4596 | -2.045 |  |
| Plpp1 | phospholipid phosphatase 1 | 0.02018 | 0.1389 | 1.428 | 0.5138 | 2.524 | 0.1208 | 0.4574 | 1.311 | 0.3903 | 1.621 | 0.0001088 | 0.001844 | 2.101 | 1.071 | 4.802 | 0.005147 | 0.03449 | 0.6154 | -0.7004 | -3.141 |  |
| Fam69a | NA | 0.1443 | 0.4174 | 0.7568 | -0.402 | -1.519 | 0.1198 | 0.4556 | 1.423 | 0.5087 | 1.625 | 3.35e-05 | 0.0007471 | 0.3437 | -1.541 | -5.316 | 0.0005442 | 0.006437 | 0.4379 | -1.191 | -4.111 |  |
| Fam189a2 | family with sequence similarit | 0.2004 | 0.495 | 1.164 | 0.2191 | 1.324 | 0.2587 | 0.6174 | 0.854 | -0.2276 | -1.163 | 2.251e-09 | 5.035e-07 | 3.607 | 1.851 | 10.21 | 0.01064 | 0.05882 | 0.7019 | -0.5107 | -2.818 |  |
| Tmem254c | transmembrane protein 254c | 0.7613 | 0.9054 | 1.054 | 0.07541 | 0.308 | 0.001124 | 0.03367 | 2.145 | 1.101 | 3.8 | 1.042e-09 | 2.792e-07 | 0.1372 | -2.865 | -10.68 | 0.4411 | 0.6658 | 0.864 | -0.2108 | -0.786 |  |
| Mthfs | 5, 10-methenyltetrahydrofolate | 0.6964 | 0.8756 | 0.9555 | -0.06561 | -0.3958 | 0.01309 | 0.1521 | 1.448 | 0.5342 | 2.724 | 0.0005929 | 0.006819 | 0.5988 | -0.7397 | -4.074 | 1.045e-05 | 0.0003052 | 0.4798 | -1.06 | -5.836 |  |
| Sema6c | sema domain, transmembrane dom | 0.009721 | 0.09086 | 0.7549 | -0.4057 | -2.858 | 0.9526 | 0.9856 | 0.993 | -0.01011 | -0.06023 | 0.0055 | 0.03654 | 1.398 | 0.4838 | 3.112 | 9.452e-10 | 2.294e-07 | 0.3142 | -1.67 | -10.74 |  |
| Serpine1 | serine (or cysteine) peptidase | 0.001519 | 0.02952 | 2.901 | 1.536 | 3.671 | 0.7658 | 0.9151 | 1.109 | 0.1495 | 0.3019 | 5.405e-05 | 0.001064 | 5.067 | 2.341 | 5.106 | 0.000759 | 0.008274 | 3.529 | 1.819 | 3.968 |  |
| Serinc2 | serine incorporator 2 | 0.7437 | 0.8976 | 1.052 | 0.07294 | 0.3316 | 0.004457 | 0.08159 | 1.783 | 0.8341 | 3.204 | 0.000307 | 0.004105 | 0.4831 | -1.05 | -4.356 | 5.244e-05 | 0.001075 | 0.4252 | -1.234 | -5.119 |  |
| Rnf122 | ring finger protein 122 | 0.01781 | 0.1295 | 0.7447 | -0.4252 | -2.582 | 0.5037 | 0.7888 | 0.9121 | -0.1327 | -0.681 | 0.008618 | 0.05092 | 1.439 | 0.5254 | 2.912 | 1.813e-07 | 1.277e-05 | 0.3782 | -1.403 | -7.777 |  |
| Stx2 | syntaxin 2 | 0.719 | 0.8861 | 0.9592 | -0.06003 | -0.3649 | 0.01309 | 0.1521 | 1.444 | 0.5301 | 2.724 | 9.433e-06 | 0.0002799 | 0.4797 | -1.06 | -5.882 | 0.0008044 | 0.008658 | 0.6111 | -0.7106 | -3.943 |  |
| Cyyr1 | cysteine and tyrosine-rich pro | 0.008312 | 0.08296 | 1.371 | 0.4549 | 2.929 | 0.2976 | 0.6523 | 0.8726 | -0.1966 | -1.07 | 3.161e-10 | 1.173e-07 | 3.858 | 1.948 | 11.45 | 0.9691 | 0.986 | 1.005 | 0.006674 | 0.03922 |  |
| Tc2n | tandem C2 domains, nuclear | 0.0009549 | 0.02205 | 1.983 | 0.9879 | 3.87 | 0.2752 | 0.6314 | 1.265 | 0.3388 | 1.122 | 0.001235 | 0.01202 | 2.073 | 1.051 | 3.76 | 0.004464 | 0.03112 | 1.861 | 0.8959 | 3.204 |  |
| Uspl1 | ubiquitin specific peptidase l | 0.08762 | 0.3183 | 1.267 | 0.3413 | 1.796 | 0.002145 | 0.051 | 1.731 | 0.7919 | 3.522 | 0.115 | 0.3097 | 0.7884 | -0.343 | -1.648 | 9.823e-05 | 0.001738 | 0.497 | -1.009 | -4.846 |  |
| Tppp3 | tubulin polymerization-promoti | 0.0005435 | 0.01552 | 0.497 | -1.009 | -4.111 | 0.1061 | 0.4328 | 0.7114 | -0.4913 | -1.693 | 0.006399 | 0.04094 | 0.5671 | -0.8184 | -3.045 | 0.01292 | 0.06772 | 0.6014 | -0.7336 | -2.73 |  |
| Atf6b | activating transcription facto | 0.03335 | 0.1874 | 0.7628 | -0.3906 | -2.285 | 0.0017 | 0.04385 | 0.6019 | -0.7325 | -3.622 | 0.9587 | 0.9836 | 1.007 | 0.009808 | 0.05239 | 2.609e-05 | 0.0006185 | 2.022 | 1.016 | 5.427 |  |
| Mirt2 | myocardial infraction associat | 0.4605 | 0.7349 | 0.8922 | -0.1646 | -0.7525 | 0.003372 | 0.06787 | 0.5506 | -0.861 | -3.326 | 1.274e-05 | 0.0003532 | 2.598 | 1.377 | 5.746 | 0.007567 | 0.0458 | 1.638 | 0.7119 | 2.971 |  |
| Gsta4 | glutathione S-transferase, alp | 6.85e-06 | 0.0009945 | 0.4339 | -1.204 | -6.028 | 8.9e-05 | 0.005449 | 0.4488 | -1.156 | -4.889 | 0.5408 | 0.7598 | 0.9099 | -0.1362 | -0.6223 | 0.9663 | 0.9847 | 0.9935 | -0.009356 | -0.04274 |  |
| Gadd45a | growth arrest and DNA-damage-i | 0.229 | 0.5295 | 1.289 | 0.3662 | 1.241 | 0.08294 | 0.3922 | 0.6428 | -0.6374 | -1.825 | 0.1423 | 0.3546 | 0.7101 | -0.4939 | -1.527 | 8.117e-10 | 2.098e-07 | 11.35 | 3.505 | 10.84 |  |
| Tbc1d7 | TBC1 domain family, member 7 | 0.02649 | 0.1635 | 0.6809 | -0.5546 | -2.396 | 0.3758 | 0.7097 | 1.188 | 0.2481 | 0.9059 | 1.191e-05 | 0.0003363 | 0.3623 | -1.465 | -5.777 | 0.001356 | 0.01276 | 0.5201 | -0.9432 | -3.72 |  |
| Aamdc | adipogenesis associated Mth938 | 0.001205 | 0.02576 | 0.5655 | -0.8225 | -3.77 | 0.3725 | 0.708 | 0.8494 | -0.2354 | -0.9122 | 0.01856 | 0.08937 | 0.6541 | -0.6124 | -2.563 | 0.0001076 | 0.001857 | 0.4511 | -1.149 | -4.807 |  |
| Gm13160 | predicted gene 13160 | 0.1387 | 0.4088 | 1.278 | 0.3534 | 1.542 | 0.1039 | 0.429 | 0.726 | -0.4619 | -1.704 | 0.01891 | 0.0906 | 1.56 | 0.6412 | 2.554 | 5.863e-07 | 3.162e-05 | 3.494 | 1.805 | 7.19 |  |
| Gprin3 | GPRIN family member 3 | 0.006093 | 0.06933 | 1.753 | 0.8099 | 3.067 | 0.4232 | 0.7428 | 0.8377 | -0.2555 | -0.8176 | 3.233e-10 | 1.173e-07 | 9.898 | 3.307 | 11.43 | 0.7049 | 0.8533 | 0.9258 | -0.1112 | -0.3842 |  |
| Gm9870 | predicted gene 9870 | 0.07765 | 0.2982 | 0.7118 | -0.4904 | -1.86 | 0.001751 | 0.04451 | 0.4582 | -1.126 | -3.61 | 0.001889 | 0.01655 | 2.046 | 1.033 | 3.577 | 0.006685 | 0.04189 | 1.833 | 0.8738 | 3.026 |  |
| Sobp | sine oculis binding protein | 0.000931 | 0.02161 | 0.588 | -0.7661 | -3.881 | 0.85 | 0.9501 | 0.9695 | -0.04474 | -0.1916 | 0.4452 | 0.6874 | 1.124 | 0.1684 | 0.7789 | 8.845e-10 | 2.182e-07 | 0.1985 | -2.332 | -10.79 |  |
| Arxes1 | adipocyte-related X-chromosome | 0.2691 | 0.5745 | 0.8342 | -0.2616 | -1.137 | 0.05742 | 0.3312 | 1.463 | 0.5489 | 2.016 | 8.803e-07 | 4.538e-05 | 0.2948 | -1.762 | -6.992 | 0.01522 | 0.07609 | 0.6289 | -0.6691 | -2.655 |  |
| Selenbp1 | selenium binding protein 1 | 0.2079 | 0.5046 | 0.7776 | -0.363 | -1.301 | 0.002692 | 0.05854 | 0.4569 | -1.13 | -3.424 | 0.001283 | 0.0124 | 2.21 | 1.144 | 3.743 | 0.001158 | 0.01137 | 2.23 | 1.157 | 3.787 |  |
| Slc22a3 | solute carrier family 22 (orga | 0.6207 | 0.8361 | 1.106 | 0.1447 | 0.5027 | 0.004799 | 0.08567 | 2.115 | 1.081 | 3.172 | 0.0001838 | 0.002762 | 0.3677 | -1.443 | -4.576 | 0.0003295 | 0.004412 | 0.3884 | -1.364 | -4.325 |  |
| Pik3r5 | phosphoinositide-3-kinase regu | 0.0003869 | 0.01255 | 2.317 | 1.212 | 4.257 | 0.0372 | 0.2708 | 1.685 | 0.7524 | 2.232 | 0.02685 | 0.1165 | 1.677 | 0.7456 | 2.39 | 0.05542 | 0.1888 | 1.553 | 0.6347 | 2.034 |  |
| Gm5424 | predicted gene 5424 | 0.0003916 | 0.01264 | 2.175 | 1.121 | 4.252 | 0.03713 | 0.2706 | 1.621 | 0.6969 | 2.233 | 0.03047 | 0.1273 | 1.594 | 0.6729 | 2.329 | 0.05085 | 0.1786 | 1.516 | 0.6003 | 2.078 |  |
| AI464131 | NA | 0.0002728 | 0.01013 | 0.4701 | -1.089 | -4.406 | 0.006136 | 0.09816 | 0.5374 | -0.8958 | -3.064 | 0.01361 | 0.07136 | 0.6018 | -0.7325 | -2.706 | 0.5779 | 0.7693 | 1.112 | 0.1532 | 0.5657 |  |
| Kcng4 | potassium voltage-gated channe | 0.001189 | 0.02549 | 0.5666 | -0.8196 | -3.776 | 0.4272 | 0.7449 | 0.8656 | -0.2082 | -0.8105 | 0.5099 | 0.7371 | 0.8953 | -0.1596 | -0.6711 | 7.91e-07 | 4.029e-05 | 0.3132 | -1.675 | -7.044 |  |
| Slc3a1 | solute carrier family 3, membe | 0.6333 | 0.8429 | 1.099 | 0.1358 | 0.4845 | 0.01562 | 0.1678 | 0.5447 | -0.8765 | -2.643 | 1.113e-06 | 5.351e-05 | 4.324 | 2.112 | 6.879 | 0.006904 | 0.04292 | 1.898 | 0.9247 | 3.011 |  |
| Sardh | sarcosine dehydrogenase | 0.001481 | 0.02901 | 0.6542 | -0.6122 | -3.682 | 0.8426 | 0.9474 | 0.9729 | -0.03958 | -0.2012 | 8.892e-07 | 4.575e-05 | 0.4139 | -1.273 | -6.987 | 0.0239 | 0.1053 | 0.7345 | -0.4452 | -2.445 |  |
| B430212C06Rik | RIKEN cDNA B430212C06 gene | 0.01208 | 0.1026 | 0.7438 | -0.427 | -2.76 | 0.4797 | 0.7753 | 1.096 | 0.1318 | 0.7203 | 1.313e-07 | 1.033e-05 | 0.3934 | -1.346 | -7.943 | 0.0653 | 0.2113 | 0.7953 | -0.3305 | -1.95 |  |
| Pknox2 | Pbx/knotted 1 homeobox 2 | 0.8878 | 0.9575 | 1.013 | 0.01882 | 0.143 | 0.003162 | 0.06522 | 1.437 | 0.5226 | 3.354 | 0.06986 | 0.2233 | 0.8257 | -0.2763 | -1.915 | 4.06e-08 | 4.315e-06 | 0.4248 | -1.235 | -8.562 |  |
| Ripply3 | ripply transcriptional repress | 0.006898 | 0.07445 | 1.308 | 0.387 | 3.012 | 0.5403 | 0.8079 | 0.9364 | -0.09473 | -0.623 | 2.634e-08 | 3.181e-06 | 2.359 | 1.238 | 8.797 | 0.1571 | 0.3685 | 1.154 | 0.2069 | 1.47 |  |
| Tmem254b | transmembrane protein 254b | 0.022 | 0.1464 | 0.6444 | -0.6339 | -2.484 | 0.3337 | 0.6797 | 1.23 | 0.2992 | 0.9907 | 4.475e-08 | 4.461e-06 | 0.1922 | -2.379 | -8.51 | 0.1486 | 0.3562 | 0.7474 | -0.4201 | -1.503 |  |
| Traj7 | T cell receptor alpha joining | 0.002712 | 0.0413 | 0.4271 | -1.227 | -3.421 | 0.0007292 | 0.0242 | 0.3095 | -1.692 | -3.985 | 0.3912 | 0.641 | 1.27 | 0.3445 | 0.8765 | 0.01472 | 0.07432 | 2.07 | 1.049 | 2.67 |  |
| P2rx3 | purinergic receptor P2X, ligan | 0.966 | 0.9882 | 1.006 | 0.008197 | 0.04314 | 0.005992 | 0.097 | 1.615 | 0.6911 | 3.074 | 0.0001158 | 0.001936 | 0.5021 | -0.9939 | -4.775 | 5.905e-05 | 0.001176 | 0.4814 | -1.055 | -5.068 |  |
| Msr1 | macrophage scavenger receptor | 0.02535 | 0.1594 | 1.662 | 0.7329 | 2.417 | 0.398 | 0.7256 | 0.8067 | -0.3099 | -0.8637 | 0.0002444 | 0.003436 | 2.789 | 1.48 | 4.453 | 7.493e-05 | 0.001419 | 3.136 | 1.649 | 4.964 |  |
| Ddx56 | DEAD (Asp-Glu-Ala-Asp) box pol | 0.000935 | 0.02169 | 1.443 | 0.5293 | 3.879 | 0.8555 | 0.952 | 1.021 | 0.02979 | 0.1845 | 0.2535 | 0.5035 | 1.13 | 0.1758 | 1.176 | 2.393e-08 | 2.81e-06 | 2.501 | 1.323 | 8.85 |  |
| Samhd1 | SAM domain and HD domain, 1 | 0.001541 | 0.02972 | 1.649 | 0.7215 | 3.665 | 0.6834 | 0.8814 | 1.069 | 0.0964 | 0.4138 | 0.002676 | 0.02153 | 1.669 | 0.739 | 3.427 | 3.73e-05 | 0.0008228 | 2.198 | 1.136 | 5.269 |  |
| Oas2 | 2'-5' oligoadenylate synthetas | 0.9265 | 0.9741 | 1.013 | 0.01886 | 0.09342 | 0.007962 | 0.1158 | 0.6138 | -0.7041 | -2.948 | 1.532e-05 | 0.0004067 | 2.383 | 1.253 | 5.664 | 0.0005031 | 0.006053 | 1.888 | 0.9165 | 4.144 |  |
| Lyz2 | lysozyme 2 | 0.09998 | 0.3415 | 1.54 | 0.623 | 1.725 | 0.1696 | 0.5283 | 0.6557 | -0.609 | -1.425 | 0.001731 | 0.01549 | 2.695 | 1.43 | 3.615 | 1.33e-05 | 0.0003698 | 4.809 | 2.266 | 5.727 |  |
| Olfm1 | olfactomedin 1 | 0.006868 | 0.07436 | 0.7152 | -0.4837 | -3.014 | 0.7056 | 0.8908 | 0.9508 | -0.07277 | -0.3832 | 2.05e-08 | 2.637e-06 | 0.3366 | -1.571 | -8.935 | 0.06881 | 0.2192 | 1.264 | 0.3381 | 1.923 |  |
| Fzd7 | frizzled class receptor 7 | 0.007748 | 0.07924 | 0.5883 | -0.7653 | -2.96 | 0.0009871 | 0.03063 | 0.4415 | -1.18 | -3.856 | 0.02042 | 0.09571 | 1.64 | 0.7133 | 2.518 | 0.07628 | 0.234 | 1.443 | 0.5295 | 1.87 |  |
| Ncf1 | neutrophil cytosolic factor 1 | 0.0003222 | 0.01126 | 2.12 | 1.084 | 4.335 | 0.02338 | 0.2099 | 1.655 | 0.7264 | 2.455 | 0.06314 | 0.2089 | 1.453 | 0.539 | 1.968 | 0.06551 | 0.2119 | 1.448 | 0.5338 | 1.949 |  |
| Adgrl2 | adhesion G protein-coupled rec | 0.003323 | 0.04687 | 0.7142 | -0.4856 | -3.332 | 0.8129 | 0.9355 | 1.029 | 0.04135 | 0.2399 | 0.005106 | 0.0346 | 0.7062 | -0.5019 | -3.145 | 1.429e-06 | 6.506e-05 | 0.4734 | -1.079 | -6.759 |  |
| Gm15446 | predicted gene 15446 | 0.8046 | 0.9242 | 1.03 | 0.04257 | 0.2508 | 0.004102 | 0.0774 | 0.6369 | -0.6509 | -3.241 | 0.2274 | 0.4719 | 1.174 | 0.2316 | 1.246 | 3.529e-09 | 6.643e-07 | 3.602 | 1.849 | 9.942 |  |
| Muc2 | mucin 2 | 0.004224 | 0.05411 | 1.619 | 0.6952 | 3.228 | 0.4117 | 0.7348 | 1.16 | 0.2137 | 0.8384 | 4.167e-07 | 2.552e-05 | 3.331 | 1.736 | 7.358 | 0.231 | 0.4633 | 0.8171 | -0.2915 | -1.235 |  |
| Mier1 | MEIR1 treanscription regulator | 0.00103 | 0.02327 | 1.795 | 0.8441 | 3.837 | 0.1365 | 0.479 | 1.323 | 0.4038 | 1.551 | 0.2059 | 0.4445 | 1.244 | 0.315 | 1.307 | 0.0004684 | 0.005729 | 2.008 | 1.006 | 4.175 |  |
| Gm7308 | predicted pseudogene 7308 | 0.001094 | 0.02407 | 0.5178 | -0.9497 | -3.812 | 0.6964 | 0.8867 | 0.9223 | -0.1167 | -0.3959 | 0.4249 | 0.6712 | 0.8572 | -0.2223 | -0.8145 | 6.136e-08 | 5.867e-06 | 0.2064 | -2.277 | -8.341 |  |
| n-R5s193 | nuclear encoded rRNA 5S 193 | 0.02977 | 0.1749 | 1.566 | 0.6467 | 2.34 | 0.001397 | 0.0384 | 2.317 | 1.212 | 3.707 | 0.008599 | 0.05081 | 0.5427 | -0.8819 | -2.913 | 0.01412 | 0.0721 | 0.5688 | -0.814 | -2.689 |  |
| Gm17296 | NA | 0.2259 | 0.526 | 0.8468 | -0.24 | -1.25 | 0.002851 | 0.06079 | 0.5855 | -0.7723 | -3.399 | 3.674e-05 | 0.0007929 | 2.158 | 1.11 | 5.276 | 0.03127 | 0.1274 | 1.402 | 0.4873 | 2.316 |  |
| Zfp771 | zinc finger protein 771 | 0.2979 | 0.6015 | 1.107 | 0.1461 | 1.069 | 0.0479 | 0.3045 | 0.7895 | -0.341 | -2.108 | 0.04198 | 0.1584 | 1.253 | 0.3255 | 2.173 | 2.657e-07 | 1.716e-05 | 2.197 | 1.136 | 7.583 |  |
| 2310040G07Rik | RIKEN cDNA 2310040G07 gene | 0.01104 | 0.09789 | 0.5523 | -0.8565 | -2.801 | 0.5652 | 0.8208 | 1.158 | 0.2116 | 0.5849 | 0.002053 | 0.0177 | 0.4395 | -1.186 | -3.541 | 6.89e-06 | 0.0002213 | 0.2469 | -2.018 | -6.025 |  |
| Manea | mannosidase, endo-alpha | 0.5939 | 0.821 | 1.066 | 0.09239 | 0.5419 | 0.004194 | 0.07834 | 1.571 | 0.6518 | 3.231 | 1.699e-06 | 7.26e-05 | 0.4213 | -1.247 | -6.677 | 0.03175 | 0.1288 | 0.7416 | -0.4313 | -2.309 |  |
| Cabyr | calcium-binding tyrosine-(Y)-p | 0.4374 | 0.7181 | 0.7676 | -0.3816 | -0.7924 | 0.004103 | 0.0774 | 0.2781 | -1.846 | -3.241 | 0.000118 | 0.001963 | 5.714 | 2.514 | 4.767 | 0.00195 | 0.01684 | 3.68 | 1.88 | 3.563 |  |
| Ftl1 | ferritin light polypeptide 1 | 0.1266 | 0.3886 | 1.185 | 0.2452 | 1.594 | 0.09332 | 0.4109 | 0.8007 | -0.3207 | -1.762 | 0.3195 | 0.5747 | 1.127 | 0.172 | 1.021 | 1.649e-08 | 2.1e-06 | 2.879 | 1.526 | 9.056 |  |
| Gm32819 | NA | 0.001012 | 0.02294 | 2.282 | 1.191 | 3.845 | 0.1649 | 0.5219 | 1.442 | 0.5281 | 1.442 | 1.239e-05 | 0.0003469 | 3.873 | 1.953 | 5.759 | 0.9211 | 0.9646 | 1.024 | 0.034 | 0.1003 |  |
| Gm13675 | predicted gene 13675 | 0.023 | 0.1499 | 0.6962 | -0.5224 | -2.463 | 0.4275 | 0.7451 | 1.151 | 0.2033 | 0.81 | 0.0001366 | 0.002185 | 0.4688 | -1.093 | -4.704 | 0.0001494 | 0.002376 | 0.4717 | -1.084 | -4.665 |  |
| Paqr7 | progestin and adipoQ receptor | 0.0456 | 0.2233 | 0.6388 | -0.6465 | -2.132 | 0.2726 | 0.6298 | 1.324 | 0.4048 | 1.128 | 1.251e-06 | 5.884e-05 | 0.2079 | -2.266 | -6.822 | 0.01464 | 0.07405 | 0.5405 | -0.8877 | -2.673 |  |
| Tet1 | tet methylcytosine dioxygenase | 0.08138 | 0.3049 | 0.7483 | -0.4182 | -1.835 | 0.1766 | 0.5372 | 1.299 | 0.3777 | 1.401 | 0.04516 | 0.1665 | 0.6909 | -0.5334 | -2.137 | 3.618e-07 | 2.174e-05 | 0.2766 | -1.854 | -7.429 |  |
| Tcaf2 | TRPM8 channel-associated facto | 0.001337 | 0.02735 | 0.6123 | -0.7077 | -3.726 | 0.4256 | 0.7438 | 0.881 | -0.1828 | -0.8134 | 2.803e-05 | 0.0006465 | 0.4593 | -1.123 | -5.395 | 0.04205 | 0.1567 | 0.731 | -0.452 | -2.172 |  |
| Ncaph | non-SMC condensin I complex, s | 0.9435 | 0.9802 | 1.008 | 0.01092 | 0.07178 | 0.005297 | 0.08999 | 0.6767 | -0.5633 | -3.128 | 0.01202 | 0.0652 | 1.376 | 0.4606 | 2.763 | 2.721e-07 | 1.737e-05 | 2.399 | 1.262 | 7.571 |  |
| Abca1 | ATP-binding cassette, sub-fami | 0.2028 | 0.498 | 1.187 | 0.2479 | 1.317 | 0.09861 | 0.4189 | 0.7653 | -0.3858 | -1.732 | 9.302e-05 | 0.001629 | 2.006 | 1.004 | 4.87 | 0.0003112 | 0.004237 | 1.862 | 0.8969 | 4.35 |  |
| Foxa3 | forkhead box A3 | 0.9117 | 0.9681 | 1.018 | 0.02587 | 0.1123 | 0.005503 | 0.09222 | 1.801 | 0.8485 | 3.112 | 0.002074 | 0.0178 | 0.5386 | -0.8928 | -3.537 | 4.328e-06 | 0.0001517 | 0.3357 | -1.575 | -6.239 |  |
| Arl4d | ADP-ribosylation factor-like 4 | 0.0003481 | 0.01186 | 3.33 | 1.736 | 4.302 | 0.02039 | 0.1956 | 2.302 | 1.203 | 2.519 | 0.4568 | 0.6961 | 1.262 | 0.3354 | 0.7589 | 0.009702 | 0.05486 | 2.401 | 1.264 | 2.859 |  |
| A230050P20Rik | NA | 0.9201 | 0.9712 | 1.014 | 0.01948 | 0.1015 | 0.008668 | 0.1212 | 0.6326 | -0.6606 | -2.91 | 9.147e-05 | 0.001608 | 2.035 | 1.025 | 4.877 | 0.0001021 | 0.001785 | 2.021 | 1.015 | 4.83 |  |
| Zfand5 | zinc finger, AN1-type domain 5 | 0.001235 | 0.02619 | 1.5 | 0.5852 | 3.76 | 0.8667 | 0.9551 | 1.022 | 0.03131 | 0.17 | 0.2026 | 0.4399 | 1.168 | 0.2246 | 1.318 | 5.21e-08 | 5.235e-06 | 2.708 | 1.437 | 8.428 |  |
| Pde7b | phosphodiesterase 7B | 0.01331 | 0.1091 | 0.7229 | -0.4681 | -2.716 | 0.5381 | 0.8067 | 1.093 | 0.1277 | 0.6264 | 0.002263 | 0.01894 | 0.6326 | -0.6606 | -3.499 | 7.797e-06 | 0.000244 | 0.4579 | -1.127 | -5.969 |  |
| Mir708 | microRNA 708 | 0.001505 | 0.02933 | 0.5557 | -0.8476 | -3.675 | 0.762 | 0.9131 | 0.9436 | -0.08379 | -0.307 | 4.07e-07 | 2.512e-05 | 0.2751 | -1.862 | -7.37 | 0.1049 | 0.2864 | 0.7426 | -0.4293 | -1.699 |  |
| Ralgapa2 | Ral GTPase activating protein, | 0.6554 | 0.8542 | 0.9445 | -0.08232 | -0.4531 | 0.01847 | 0.1851 | 1.466 | 0.5515 | 2.565 | 0.0005363 | 0.006313 | 0.5667 | -0.8194 | -4.117 | 2.554e-05 | 0.0006078 | 0.4724 | -1.082 | -5.436 |  |
| Rgs5 | regulator of G-protein signali | 0.5637 | 0.8029 | 0.9062 | -0.142 | -0.5872 | 0.0215 | 0.2014 | 1.64 | 0.714 | 2.494 | 1.265e-06 | 5.928e-05 | 0.2859 | -1.807 | -6.817 | 0.009397 | 0.05368 | 0.5899 | -0.7616 | -2.874 |  |
| Avl9 | AVL9 cell migration associated | 0.01522 | 0.1178 | 1.345 | 0.4275 | 2.655 | 0.5215 | 0.7987 | 0.9174 | -0.1243 | -0.6525 | 1.392e-05 | 0.0003766 | 2.009 | 1.007 | 5.707 | 0.001493 | 0.01372 | 1.568 | 0.6489 | 3.678 |  |
| Hif3a | hypoxia inducible factor 3, al | 0.005411 | 0.06411 | 1.514 | 0.5981 | 3.119 | 0.7781 | 0.9199 | 0.9561 | -0.06481 | -0.2857 | 4.393e-05 | 0.0009084 | 2.131 | 1.092 | 5.197 | 0.0003745 | 0.00485 | 1.862 | 0.897 | 4.271 |  |
| Gstm4 | glutathione S-transferase, mu | 0.00107 | 0.02382 | 0.5501 | -0.8622 | -3.821 | 0.0006283 | 0.02193 | 0.4727 | -1.081 | -4.049 | 0.2791 | 0.533 | 1.21 | 0.275 | 1.113 | 0.1385 | 0.3399 | 1.303 | 0.3814 | 1.543 |  |
| Vcan | versican | 0.001 | 0.02275 | 0.4889 | -1.033 | -3.85 | 0.1706 | 0.529 | 0.7315 | -0.451 | -1.421 | 0.00075 | 0.008116 | 0.4452 | -1.167 | -3.973 | 0.0635 | 0.2073 | 0.6703 | -0.5772 | -1.965 |  |
| Nova1 | NOVA alternative splicing regu | 0.2986 | 0.602 | 1.145 | 0.1956 | 1.067 | 0.06296 | 0.3467 | 0.7438 | -0.427 | -1.969 | 7.26e-06 | 0.0002303 | 2.305 | 1.205 | 6.001 | 0.003559 | 0.02644 | 1.583 | 0.663 | 3.303 |  |
| Tmem182 | transmembrane protein 182 | 0.00154 | 0.02972 | 0.6621 | -0.5949 | -3.665 | 0.7694 | 0.9162 | 1.04 | 0.05708 | 0.2972 | 3.426e-10 | 1.224e-07 | 0.2455 | -2.026 | -11.39 | 0.6976 | 0.8487 | 1.05 | 0.07009 | 0.3942 |  |
| Pebp4 | phosphatidylethanolamine bindi | 0.001502 | 0.02929 | 0.678 | -0.5607 | -3.676 | 0.3719 | 0.7077 | 0.892 | -0.1649 | -0.9134 | 0.6566 | 0.8337 | 0.9491 | -0.07542 | -0.4513 | 1.777e-06 | 7.667e-05 | 0.4626 | -1.112 | -6.655 |  |
| Mreg | melanoregulin | 0.214 | 0.5127 | 0.8489 | -0.2363 | -1.284 | 0.009165 | 0.1244 | 0.6469 | -0.6284 | -2.885 | 1.187e-06 | 5.652e-05 | 2.604 | 1.381 | 6.848 | 0.3222 | 0.5626 | 0.8677 | -0.2047 | -1.015 |  |
| Usp33 | ubiquitin specific peptidase 3 | 0.00447 | 0.05634 | 1.359 | 0.443 | 3.203 | 0.3395 | 0.6839 | 1.117 | 0.1601 | 0.9785 | 1.349e-06 | 6.166e-05 | 2.04 | 1.028 | 6.786 | 0.2499 | 0.485 | 0.883 | -0.1796 | -1.185 |  |
| Omd | osteomodulin | 0.006276 | 0.07041 | 1.831 | 0.8723 | 3.054 | 0.001097 | 0.03336 | 2.442 | 1.288 | 3.811 | 0.08069 | 0.2463 | 0.6709 | -0.5757 | -1.84 | 0.04441 | 0.1624 | 0.628 | -0.6713 | -2.145 |  |
| Ocstamp | osteoclast stimulatory transme | 0.7436 | 0.8975 | 1.044 | 0.06273 | 0.3317 | 0.005755 | 0.09478 | 1.615 | 0.6918 | 3.092 | 0.0008004 | 0.008544 | 0.5675 | -0.8174 | -3.946 | 4.728e-05 | 0.0009953 | 0.4763 | -1.07 | -5.165 |  |
| Esr1 | estrogen receptor 1 (alpha) | 0.001624 | 0.03069 | 0.5363 | -0.8988 | -3.642 | 0.6099 | 0.8449 | 0.9004 | -0.1514 | -0.5184 | 0.007996 | 0.0482 | 0.5758 | -0.7964 | -2.946 | 3.111e-05 | 0.0007119 | 0.367 | -1.446 | -5.349 |  |
| Stc2 | stanniocalcin 2 | 0.07332 | 0.2893 | 1.335 | 0.4173 | 1.89 | 0.002293 | 0.05265 | 1.882 | 0.9126 | 3.493 | 4.477e-05 | 0.0009221 | 0.419 | -1.255 | -5.189 | 0.3511 | 0.5891 | 0.8521 | -0.2309 | -0.9548 |  |
| Sbk1 | SH3-binding kinase 1 | 0.1061 | 0.3524 | 1.491 | 0.5767 | 1.693 | 0.002338 | 0.05332 | 2.648 | 1.405 | 3.485 | 0.001432 | 0.01347 | 0.3843 | -1.38 | -3.696 | 0.007737 | 0.04661 | 0.4649 | -1.105 | -2.961 |  |
| Comt | catechol-O-methyltransferase | 0.08576 | 0.3144 | 0.6977 | -0.5193 | -1.808 | 0.00261 | 0.05724 | 0.4449 | -1.169 | -3.437 | 0.05124 | 0.1813 | 1.572 | 0.6527 | 2.074 | 0.0005393 | 0.006395 | 2.454 | 1.295 | 4.115 |  |
| Gm7278 | predicted gene 7278 | 0.002133 | 0.03633 | 1.834 | 0.8749 | 3.525 | 0.06231 | 0.3448 | 1.495 | 0.5799 | 1.975 | 0.4 | 0.6496 | 0.8503 | -0.2339 | -0.8601 | 0.0005395 | 0.006396 | 2.172 | 1.119 | 4.114 |  |
| Olfr558 | olfactory receptor 558 | 0.03322 | 0.1868 | 0.6954 | -0.5241 | -2.287 | 0.3801 | 0.7123 | 1.184 | 0.2433 | 0.8976 | 9.364e-05 | 0.001637 | 0.4288 | -1.222 | -4.867 | 0.0003451 | 0.004573 | 0.4728 | -1.081 | -4.306 |  |
| Banp | BTG3 associated nuclear protei | 0.01414 | 0.1125 | 1.666 | 0.7361 | 2.688 | 0.00172 | 0.04406 | 2.253 | 1.172 | 3.617 | 0.4146 | 0.6623 | 0.841 | -0.2499 | -0.8331 | 0.002126 | 0.018 | 0.4804 | -1.058 | -3.526 |  |
| Col14a1 | collagen, type XIV, alpha 1 | 0.002489 | 0.0393 | 0.5955 | -0.7478 | -3.458 | 0.0007848 | 0.02562 | 0.4959 | -1.012 | -3.954 | 0.04954 | 0.1774 | 1.41 | 0.4953 | 2.091 | 0.225 | 0.4558 | 1.228 | 0.2966 | 1.252 |  |
| Slc4a10 | solute carrier family 4, sodiu | 0.000418 | 0.01316 | 0.4444 | -1.17 | -4.224 | 0.02122 | 0.2001 | 0.5666 | -0.8196 | -2.5 | 0.984 | 0.9948 | 1.004 | 0.006144 | 0.02025 | 0.00234 | 0.01928 | 0.4805 | -1.057 | -3.485 |  |
| Higd1a | HIG1 domain family, member 1A | 0.01603 | 0.1214 | 0.7248 | -0.4643 | -2.631 | 0.5232 | 0.8 | 1.099 | 0.1357 | 0.6499 | 2.341e-05 | 0.0005623 | 0.4801 | -1.058 | -5.475 | 0.001035 | 0.01044 | 0.5981 | -0.7416 | -3.836 |  |
| Gpr65 | G-protein coupled receptor 65 | 0.001304 | 0.02694 | 2.499 | 1.322 | 3.737 | 0.3046 | 0.6584 | 1.358 | 0.441 | 1.054 | 0.007097 | 0.0442 | 2.238 | 1.162 | 2.999 | 0.001075 | 0.01073 | 2.789 | 1.48 | 3.819 |  |
| Mc5r | melanocortin 5 receptor | 0.06633 | 0.2742 | 1.634 | 0.7082 | 1.942 | 0.8747 | 0.9577 | 1.049 | 0.06889 | 0.1597 | 3.322e-08 | 3.682e-06 | 11.03 | 3.463 | 8.67 | 0.0009583 | 0.009827 | 0.3427 | -1.545 | -3.869 |  |
| Dhrs7 | dehydrogenase/reductase (SDR f | 0.2011 | 0.496 | 0.8106 | -0.3029 | -1.322 | 0.06368 | 0.3473 | 1.446 | 0.5322 | 1.963 | 1.544e-08 | 2.136e-06 | 0.2056 | -2.282 | -9.092 | 0.384 | 0.618 | 0.8566 | -0.2234 | -0.8901 |  |
| Gcnt2 | glucosaminyl (N-acetyl) transf | 0.00155 | 0.02983 | 1.599 | 0.6775 | 3.662 | 0.5734 | 0.8251 | 1.091 | 0.1253 | 0.5724 | 2.237e-06 | 9.078e-05 | 2.508 | 1.327 | 6.546 | 0.1193 | 0.3099 | 1.257 | 0.3299 | 1.628 |  |
| Maged2 | melanoma antigen, family D, 2 | 0.00153 | 0.02966 | 0.6266 | -0.6745 | -3.668 | 0.4228 | 0.7425 | 0.8839 | -0.1781 | -0.8185 | 2.433e-05 | 0.0005806 | 0.4667 | -1.099 | -5.458 | 0.06732 | 0.216 | 0.7633 | -0.3897 | -1.935 |  |
| Fam69b | NA | 0.3186 | 0.6197 | 0.8726 | -0.1967 | -1.023 | 0.02645 | 0.2247 | 1.459 | 0.5452 | 2.397 | 3.722e-09 | 7.254e-07 | 0.2353 | -2.087 | -9.911 | 0.5192 | 0.7278 | 0.9086 | -0.1382 | -0.6561 |  |
| Mcee | methylmalonyl CoA epimerase | 0.4695 | 0.7414 | 0.9283 | -0.1073 | -0.7373 | 0.03017 | 0.2411 | 1.321 | 0.4018 | 2.334 | 3.363e-07 | 2.168e-05 | 0.4383 | -1.19 | -7.465 | 0.04708 | 0.1691 | 0.7915 | -0.3374 | -2.116 |  |
| Mamstr | MEF2 activating motif and SAP | 0.05495 | 0.248 | 0.7333 | -0.4475 | -2.039 | 0.3079 | 0.6606 | 0.8283 | -0.2717 | -1.046 | 0.00184 | 0.01623 | 1.819 | 0.8629 | 3.588 | 1.18e-05 | 0.000337 | 0.3815 | -1.39 | -5.781 |  |
| Efr3b | EFR3 homolog B | 0.00232 | 0.03777 | 0.6381 | -0.6482 | -3.488 | 0.1767 | 0.5372 | 0.8078 | -0.3079 | -1.4 | 0.6285 | 0.8153 | 1.072 | 0.1 | 0.4914 | 2.179e-05 | 0.000537 | 0.4598 | -1.121 | -5.506 |  |
| Col6a6 | collagen, type VI, alpha 6 | 0.187 | 0.4777 | 0.818 | -0.2899 | -1.366 | 0.1124 | 0.4443 | 1.335 | 0.4169 | 1.661 | 0.000641 | 0.007212 | 0.5216 | -0.9391 | -4.041 | 5.697e-05 | 0.001144 | 0.4409 | -1.181 | -5.083 |  |
| Fndc3a | fibronectin type III domain co | 0.009205 | 0.08802 | 1.362 | 0.4454 | 2.883 | 0.6248 | 0.8528 | 0.939 | -0.0908 | -0.4967 | 9.544e-07 | 4.852e-05 | 2.261 | 1.177 | 6.953 | 0.01901 | 0.08922 | 1.349 | 0.4319 | 2.552 |  |
| Ar | androgen receptor | 0.01205 | 0.1024 | 0.6208 | -0.6878 | -2.761 | 0.6059 | 0.8435 | 1.113 | 0.1545 | 0.5242 | 0.0001916 | 0.002851 | 0.4222 | -1.244 | -4.558 | 0.0001371 | 0.002239 | 0.4109 | -1.283 | -4.702 |  |
| Dupd1 | dual specificity phosphatase a | 0.002373 | 0.03827 | 0.6572 | -0.6057 | -3.479 | 0.2626 | 0.6203 | 0.8482 | -0.2375 | -1.153 | 0.6482 | 0.8276 | 1.063 | 0.08834 | 0.4632 | 4.185e-06 | 0.0001485 | 0.4374 | -1.193 | -6.254 |  |
| Tirap | toll-interleukin 1 receptor (T | 0.001913 | 0.03405 | 0.5228 | -0.9357 | -3.572 | 0.0007847 | 0.02562 | 0.4276 | -1.226 | -3.954 | 0.02687 | 0.1165 | 1.608 | 0.6856 | 2.389 | 0.5279 | 0.7341 | 1.136 | 0.1844 | 0.6424 |  |
| Nav1 | neuron navigator 1 | 0.9489 | 0.9819 | 0.9932 | -0.009823 | -0.06488 | 0.005833 | 0.09545 | 0.6817 | -0.5529 | -3.086 | 5.561e-07 | 3.165e-05 | 2.292 | 1.197 | 7.216 | 0.01691 | 0.08192 | 1.349 | 0.4323 | 2.606 |  |
| Ptchd4 | patched domain containing 4 | 0.06723 | 0.2762 | 0.7902 | -0.3397 | -1.935 | 0.2206 | 0.582 | 1.2 | 0.2626 | 1.265 | 1.937e-07 | 1.42e-05 | 0.3563 | -1.489 | -7.743 | 0.1136 | 0.3013 | 0.8021 | -0.3181 | -1.655 |  |
| Cxcl13 | chemokine (C-X-C motif) ligand | 0.0007169 | 0.01824 | 9.601 | 3.263 | 3.993 | 0.02652 | 0.225 | 4.981 | 2.316 | 2.395 | 0.7735 | 0.8969 | 0.8344 | -0.2611 | -0.2917 | 0.002452 | 0.02 | 8.584 | 3.102 | 3.464 |  |
| Zfp180 | zinc finger protein 180 | 0.5854 | 0.8162 | 1.076 | 0.1051 | 0.5545 | 0.005629 | 0.09342 | 1.62 | 0.6958 | 3.102 | 0.001705 | 0.01537 | 0.5937 | -0.7521 | -3.621 | 8.469e-05 | 0.001547 | 0.4931 | -1.02 | -4.911 |  |
| Cnnm3 | cyclin M3 | 0.00252 | 0.03955 | 1.435 | 0.5212 | 3.453 | 0.9285 | 0.9773 | 0.9888 | -0.01624 | -0.0909 | 0.00448 | 0.03144 | 1.443 | 0.5295 | 3.202 | 2.23e-06 | 9.092e-05 | 2.118 | 1.083 | 6.548 |  |
| Mum1l1 | NA | 0.001494 | 0.02917 | 0.6487 | -0.6243 | -3.678 | 0.6126 | 0.8465 | 0.9309 | -0.1033 | -0.5144 | 0.5698 | 0.7794 | 0.9282 | -0.1075 | -0.5779 | 1.641e-07 | 1.203e-05 | 0.3646 | -1.456 | -7.828 |  |
| Mn1 | meningioma 1 | 0.5868 | 0.8167 | 1.106 | 0.1448 | 0.5524 | 0.02567 | 0.2207 | 0.5956 | -0.7476 | -2.411 | 2.761e-05 | 0.0006393 | 2.93 | 1.551 | 5.402 | 0.0008427 | 0.00893 | 2.183 | 1.126 | 3.924 |  |
| Fgf2 | fibroblast growth factor 2 | 0.29 | 0.594 | 0.8357 | -0.259 | -1.087 | 0.06396 | 0.3478 | 1.467 | 0.5529 | 1.961 | 7.161e-07 | 3.857e-05 | 0.2771 | -1.851 | -7.092 | 0.03671 | 0.1423 | 0.6669 | -0.5844 | -2.239 |  |
| Fmo5 | flavin containing monooxygenas | 0.003484 | 0.04802 | 1.683 | 0.7506 | 3.312 | 0.3579 | 0.6977 | 1.191 | 0.2524 | 0.9411 | 1.111e-06 | 5.351e-05 | 3.267 | 1.708 | 6.88 | 0.4006 | 0.6321 | 0.8626 | -0.2132 | -0.8588 |  |
| Gm13375 | predicted gene 13375 | 0.001617 | 0.03061 | 0.5799 | -0.7861 | -3.644 | 0.5812 | 0.8297 | 0.9056 | -0.1431 | -0.5608 | 1.384e-06 | 6.226e-05 | 0.3297 | -1.601 | -6.774 | 0.1803 | 0.4005 | 0.7966 | -0.3281 | -1.388 |  |
| Tlr4 | toll-like receptor 4 | 0.001595 | 0.03035 | 1.738 | 0.7975 | 3.65 | 0.2787 | 0.6343 | 1.221 | 0.2879 | 1.114 | 0.06152 | 0.2054 | 1.389 | 0.4742 | 1.981 | 0.000273 | 0.003824 | 2.077 | 1.055 | 4.406 |  |
| Psip1 | PC4 and SFRS1 interacting prot | 0.9037 | 0.9641 | 1.015 | 0.02198 | 0.1225 | 0.01007 | 0.1315 | 0.6582 | -0.6034 | -2.842 | 0.001091 | 0.0109 | 1.681 | 0.7494 | 3.813 | 1.312e-05 | 0.0003658 | 2.184 | 1.127 | 5.733 |  |
| Mettl7a3 | methyltransferase like 7A3 | 0.2325 | 0.5339 | 1.165 | 0.2208 | 1.231 | 0.09233 | 0.4096 | 0.771 | -0.3751 | -1.768 | 2.579e-05 | 0.000607 | 2.095 | 1.067 | 5.432 | 0.001524 | 0.01394 | 1.648 | 0.7208 | 3.669 |  |
| Antxr2 | anthrax toxin receptor 2 | 0.02133 | 0.1433 | 0.7404 | -0.4336 | -2.498 | 0.4653 | 0.7679 | 1.112 | 0.1529 | 0.7445 | 5.164e-06 | 0.0001774 | 0.4442 | -1.171 | -6.157 | 0.005691 | 0.03724 | 0.6649 | -0.5888 | -3.097 |  |
| Gm13271 | predicted gene 13271 | 0.06076 | 0.2614 | 1.181 | 0.2406 | 1.987 | 0.2065 | 0.5691 | 0.8784 | -0.187 | -1.306 | 2.653e-08 | 3.189e-06 | 2.244 | 1.166 | 8.793 | 0.389 | 0.6224 | 1.084 | 0.1168 | 0.8805 |  |
| Slc12a2 | solute carrier family 12, memb | 0.001807 | 0.0328 | 1.725 | 0.7863 | 3.596 | 0.4465 | 0.7569 | 1.149 | 0.2009 | 0.7766 | 0.174 | 0.4012 | 1.264 | 0.3376 | 1.41 | 8.633e-06 | 0.0002622 | 2.673 | 1.418 | 5.922 |  |
| Dll4 | delta like canonical Notch lig | 0.001363 | 0.02762 | 0.5671 | -0.8183 | -3.718 | 0.8139 | 0.9357 | 0.9578 | -0.06213 | -0.2386 | 4.424e-08 | 4.436e-06 | 0.2409 | -2.053 | -8.516 | 0.3829 | 0.6168 | 0.8615 | -0.2152 | -0.8922 |  |
| Naa20 | N(alpha)-acetyltransferase 20, | 0.589 | 0.8178 | 1.085 | 0.1174 | 0.5492 | 0.005757 | 0.09478 | 1.72 | 0.7821 | 3.092 | 0.000144 | 0.002284 | 0.4677 | -1.096 | -4.681 | 0.001041 | 0.01048 | 0.5367 | -0.8977 | -3.833 |  |
| Csmd1 | CUB and Sushi multiple domains | 0.0005171 | 0.01505 | 2.011 | 1.008 | 4.133 | 0.003219 | 0.06598 | 1.953 | 0.9654 | 3.346 | 0.2913 | 0.5465 | 0.8182 | -0.2895 | -1.084 | 0.1349 | 0.3343 | 1.334 | 0.4162 | 1.558 |  |
| Dpep1 | dipeptidase 1 | 0.0002318 | 0.009256 | 2.115 | 1.081 | 4.476 | 0.0007416 | 0.02455 | 2.199 | 1.137 | 3.978 | 0.6463 | 0.8263 | 1.089 | 0.1232 | 0.4659 | 0.2851 | 0.5256 | 0.8176 | -0.2905 | -1.098 |  |
| Gm13167 | predicted gene 13167 | 0.1964 | 0.4898 | 1.225 | 0.2925 | 1.337 | 0.09451 | 0.4122 | 0.7297 | -0.4546 | -1.755 | 0.03663 | 0.1448 | 1.451 | 0.537 | 2.24 | 6.923e-07 | 3.598e-05 | 3.259 | 1.704 | 7.109 |  |
| Fdx1 | ferredoxin 1 | 0.2658 | 0.5716 | 0.8573 | -0.2221 | -1.145 | 0.004005 | 0.07638 | 0.5961 | -0.7463 | -3.251 | 3.195e-05 | 0.0007209 | 2.195 | 1.134 | 5.337 | 0.05244 | 0.1824 | 1.355 | 0.4382 | 2.062 |  |
| Adamts15 | a disintegrin-like and metallo | 0.2105 | 0.5082 | 0.8315 | -0.2662 | -1.294 | 0.09468 | 0.4125 | 1.345 | 0.4271 | 1.754 | 3.406e-06 | 0.0001261 | 0.3708 | -1.431 | -6.35 | 0.0093 | 0.05328 | 0.6378 | -0.6488 | -2.878 |  |
| BC005537 | cDNA sequence BC005537 | 0.002383 | 0.03838 | 1.405 | 0.4909 | 3.477 | 0.9118 | 0.9702 | 0.9871 | -0.01875 | -0.1122 | 0.0372 | 0.1464 | 1.27 | 0.3453 | 2.232 | 2.316e-07 | 1.567e-05 | 2.272 | 1.184 | 7.653 |  |
| Tanc2 | tetratricopeptide repeat, anky | 0.002308 | 0.03768 | 0.6715 | -0.5746 | -3.491 | 0.9518 | 0.9852 | 0.9918 | -0.01193 | -0.06126 | 6.18e-06 | 0.0002046 | 0.468 | -1.095 | -6.075 | 0.003696 | 0.02721 | 0.6632 | -0.5926 | -3.286 |  |
| Rmdn3 | regulator of microtubule dynam | 0.6478 | 0.8495 | 0.9468 | -0.07887 | -0.4638 | 0.0221 | 0.2042 | 1.414 | 0.4993 | 2.482 | 1.509e-05 | 0.0004018 | 0.4808 | -1.056 | -5.671 | 0.00163 | 0.01467 | 0.6249 | -0.6782 | -3.641 |  |
| Aldh3b2 | aldehyde dehydrogenase 3 famil | 0.006177 | 0.06977 | 0.5328 | -0.9083 | -3.061 | 0.7196 | 0.8962 | 1.093 | 0.1278 | 0.3641 | 6.722e-07 | 3.671e-05 | 0.2009 | -2.316 | -7.123 | 0.02421 | 0.1063 | 0.5772 | -0.7928 | -2.439 |  |
| Gbp10 | guanylate-binding protein 10 | 0.6691 | 0.862 | 0.9163 | -0.126 | -0.4337 | 0.005635 | 0.09345 | 0.4776 | -1.066 | -3.101 | 0.0439 | 0.1636 | 1.607 | 0.6846 | 2.151 | 1.583e-06 | 7.037e-05 | 4.395 | 2.136 | 6.71 |  |
| Rasgrp3 | RAS, guanyl releasing protein | 0.001834 | 0.0332 | 0.6757 | -0.5655 | -3.59 | 0.6606 | 0.8709 | 0.944 | -0.08308 | -0.4457 | 0.1647 | 0.3879 | 0.8415 | -0.2489 | -1.442 | 9.707e-07 | 4.795e-05 | 0.4357 | -1.198 | -6.945 |  |
| Rhpn2 | rhophilin, Rho GTPase binding | 0.08993 | 0.3231 | 1.308 | 0.3872 | 1.782 | 0.1837 | 0.5445 | 0.7824 | -0.3541 | -1.377 | 0.09295 | 0.2703 | 1.338 | 0.42 | 1.764 | 2.681e-07 | 1.72e-05 | 3.492 | 1.804 | 7.579 |  |
| 1700017B05Rik | RIKEN cDNA 1700017B05 gene | 0.3814 | 0.6742 | 1.143 | 0.1933 | 0.8951 | 0.05288 | 0.3178 | 0.6945 | -0.5259 | -2.058 | 4.462e-05 | 0.0009206 | 2.342 | 1.228 | 5.19 | 0.0008307 | 0.008863 | 1.905 | 0.9298 | 3.93 |  |
| Ece1 | endothelin converting enzyme 1 | 0.1571 | 0.4354 | 1.16 | 0.2147 | 1.47 | 0.08161 | 0.3899 | 0.8028 | -0.3169 | -1.834 | 0.5738 | 0.7819 | 1.065 | 0.09148 | 0.5718 | 9.009e-09 | 1.332e-06 | 2.835 | 1.503 | 9.397 |  |
| Mkl1 | NA | 0.001352 | 0.02749 | 0.6233 | -0.6819 | -3.721 | 0.9666 | 0.9889 | 0.9936 | -0.009201 | -0.04243 | 0.4683 | 0.7053 | 1.108 | 0.1484 | 0.7393 | 8.763e-10 | 2.182e-07 | 0.2227 | -2.167 | -10.79 |  |
| Lbp | lipopolysaccharide binding pro | 0.7518 | 0.9011 | 0.93 | -0.1047 | -0.3206 | 0.009838 | 0.1298 | 2.148 | 1.103 | 2.853 | 3.529e-08 | 3.822e-06 | 0.1173 | -3.091 | -8.638 | 0.1531 | 0.3625 | 0.6918 | -0.5316 | -1.485 |  |
| Mir181b-2 | microRNA 181b-2 | 0.00127 | 0.02661 | 0.5242 | -0.9319 | -3.748 | 0.6801 | 0.8798 | 0.9182 | -0.1231 | -0.4184 | 0.7911 | 0.9057 | 0.9506 | -0.07314 | -0.2685 | 3.516e-08 | 3.832e-06 | 0.1957 | -2.353 | -8.64 |  |
| Anxa2 | annexin A2 | 0.000277 | 0.01021 | 0.4085 | -1.291 | -4.4 | 0.009693 | 0.1289 | 0.5024 | -0.9932 | -2.86 | 0.02542 | 0.1122 | 0.5837 | -0.7767 | -2.416 | 0.7171 | 0.8608 | 0.9214 | -0.1182 | -0.3675 |  |
| Htra1 | HtrA serine peptidase 1 | 0.009208 | 0.08802 | 0.5869 | -0.7689 | -2.883 | 0.004335 | 0.08016 | 0.4948 | -1.015 | -3.216 | 0.3977 | 0.6474 | 0.8394 | -0.2525 | -0.8642 | 0.002899 | 0.02259 | 1.988 | 0.991 | 3.392 |  |
| Gdpd1 | glycerophosphodiester phosphod | 0.2055 | 0.501 | 1.201 | 0.2637 | 1.309 | 0.2251 | 0.5866 | 1.23 | 0.2985 | 1.252 | 0.0001522 | 0.002382 | 2.039 | 1.028 | 4.657 | 5.123e-05 | 0.001058 | 0.4561 | -1.133 | -5.13 |  |
| Fkbp14 | FK506 binding protein 14 | 0.01008 | 0.09302 | 0.6752 | -0.5667 | -2.842 | 0.6608 | 0.8709 | 1.076 | 0.1051 | 0.4454 | 0.0003422 | 0.004459 | 0.5208 | -0.9412 | -4.309 | 8.333e-05 | 0.001527 | 0.475 | -1.074 | -4.918 |  |
| Lrrk1 | leucine-rich repeat kinase 1 | 0.06766 | 0.2771 | 1.263 | 0.3373 | 1.932 | 0.2623 | 0.6202 | 0.8478 | -0.2383 | -1.154 | 1.977e-05 | 0.00049 | 2.087 | 1.061 | 5.55 | 0.002368 | 0.01949 | 1.586 | 0.6654 | 3.479 |  |
| Tdg-ps | thymine DNA glycosylase, pseud | 0.3055 | 0.6084 | 1.178 | 0.2365 | 1.052 | 0.004207 | 0.07853 | 1.814 | 0.8592 | 3.23 | 2.832e-05 | 0.0006504 | 0.3984 | -1.328 | -5.39 | 0.04018 | 0.1516 | 0.6875 | -0.5405 | -2.195 |  |
| Ms4a6b | membrane-spanning 4-domains, s | 0.0008893 | 0.02104 | 3.68 | 1.88 | 3.901 | 0.09313 | 0.4109 | 2.008 | 1.006 | 1.763 | 0.03408 | 0.1377 | 2.299 | 1.201 | 2.275 | 0.014 | 0.07161 | 2.679 | 1.422 | 2.693 |  |
| Oxtr | oxytocin receptor | 0.001942 | 0.03432 | 0.5667 | -0.8194 | -3.565 | 0.8798 | 0.9594 | 1.029 | 0.04166 | 0.1532 | 1.163e-08 | 1.699e-06 | 0.199 | -2.329 | -9.252 | 0.3255 | 0.5656 | 0.8387 | -0.2538 | -1.008 |  |
| Pdhb | pyruvate dehydrogenase (lipoam | 0.001941 | 0.03432 | 0.6265 | -0.6745 | -3.565 | 0.7323 | 0.9002 | 0.9476 | -0.07767 | -0.347 | 3.976e-06 | 0.0001429 | 0.4058 | -1.301 | -6.278 | 0.02844 | 0.119 | 0.7123 | -0.4895 | -2.362 |  |
| Adgrg2 | adhesion G protein-coupled rec | 0.000363 | 0.01211 | 2.335 | 1.223 | 4.284 | 0.003128 | 0.06477 | 2.196 | 1.135 | 3.359 | 0.1231 | 0.323 | 1.418 | 0.5036 | 1.61 | 0.4551 | 0.6775 | 0.8478 | -0.2383 | -0.7617 |  |
| Olfr1160 | olfactory receptor 1160 | 0.07863 | 0.3001 | 0.6488 | -0.6241 | -1.854 | 0.002679 | 0.05835 | 0.3883 | -1.365 | -3.426 | 0.0007247 | 0.007905 | 2.772 | 1.471 | 3.988 | 0.0551 | 0.1881 | 1.683 | 0.7514 | 2.037 |  |
| Ugt1a8 | UDP glucuronosyltransferase 1 | 0.6109 | 0.8311 | 1.236 | 0.3052 | 0.5169 | 0.006515 | 0.1023 | 4.353 | 2.122 | 3.037 | 0.0003993 | 0.00504 | 0.1492 | -2.745 | -4.243 | 0.0004601 | 0.00566 | 0.1533 | -2.706 | -4.183 |  |
| Nes | nestin | 0.0001323 | 0.006446 | 0.3365 | -1.572 | -4.718 | 0.002199 | 0.05157 | 0.3831 | -1.384 | -3.511 | 0.9208 | 0.9675 | 1.026 | 0.03674 | 0.1007 | 0.1162 | 0.3049 | 0.6601 | -0.5992 | -1.642 |  |
| Slc15a2 | solute carrier family 15 (H+/p | 0.0003586 | 0.01201 | 2.445 | 1.29 | 4.289 | 0.01359 | 0.1549 | 1.949 | 0.963 | 2.707 | 0.7048 | 0.8604 | 1.092 | 0.1266 | 0.3844 | 0.01744 | 0.08363 | 1.807 | 0.8538 | 2.592 |  |
| Cyp4b1 | cytochrome P450, family 4, sub | 0.005683 | 0.06645 | 1.747 | 0.8052 | 3.097 | 0.8022 | 0.9303 | 0.9473 | -0.0781 | -0.2539 | 6.295e-06 | 0.0002073 | 3.312 | 1.728 | 6.066 | 0.004117 | 0.02943 | 1.895 | 0.9224 | 3.239 |  |
| Adra1a | adrenergic receptor, alpha 1a | 0.8536 | 0.945 | 0.9792 | -0.03035 | -0.1869 | 0.01194 | 0.1447 | 1.445 | 0.5313 | 2.766 | 0.002178 | 0.01844 | 0.6483 | -0.6253 | -3.516 | 7.985e-06 | 0.0002472 | 0.4798 | -1.06 | -5.958 |  |
| Apol11b | apolipoprotein L 11b | 0.03181 | 0.1821 | 0.6472 | -0.6277 | -2.308 | 0.002002 | 0.04862 | 0.4529 | -1.143 | -3.552 | 0.01445 | 0.07445 | 1.739 | 0.7978 | 2.679 | 0.0208 | 0.09528 | 1.679 | 0.7476 | 2.51 |  |
| Tspan3 | tetraspanin 3 | 0.001849 | 0.03333 | 0.661 | -0.5972 | -3.586 | 0.5064 | 0.7904 | 0.9117 | -0.1333 | -0.6766 | 1.549e-05 | 0.0004105 | 0.489 | -1.032 | -5.659 | 0.06285 | 0.2059 | 0.7795 | -0.3594 | -1.97 |  |
| St3gal6 | ST3 beta-galactoside alpha-2,3 | 0.102 | 0.3449 | 1.271 | 0.3457 | 1.714 | 0.08106 | 0.3892 | 1.355 | 0.4385 | 1.838 | 0.002988 | 0.02338 | 1.678 | 0.7465 | 3.379 | 0.0001589 | 0.002491 | 0.4915 | -1.025 | -4.639 |  |
| Tiam2 | T cell lymphoma invasion and m | 0.5211 | 0.776 | 0.9035 | -0.1464 | -0.6532 | 0.005598 | 0.09332 | 0.5653 | -0.823 | -3.104 | 0.00705 | 0.044 | 1.667 | 0.7369 | 3.002 | 3.742e-05 | 0.0008239 | 2.45 | 1.293 | 5.268 |  |
| Ppm1b | protein phosphatase 1B, magnes | 0.0202 | 0.1389 | 1.352 | 0.4349 | 2.524 | 0.5024 | 0.7885 | 0.908 | -0.1393 | -0.683 | 1.205e-05 | 0.0003396 | 2.128 | 1.09 | 5.771 | 0.00317 | 0.02418 | 1.551 | 0.633 | 3.353 |  |
| Tia1 | cytotoxic granule-associated R | 0.3594 | 0.6552 | 0.9084 | -0.1385 | -0.9381 | 0.05449 | 0.3226 | 1.281 | 0.3569 | 2.043 | 0.01685 | 0.0832 | 0.7465 | -0.4219 | -2.608 | 2.081e-06 | 8.729e-05 | 0.4781 | -1.065 | -6.581 |  |
| Hivep2 | human immunodeficiency virus t | 0.1123 | 0.3634 | 0.7829 | -0.3531 | -1.661 | 0.1926 | 0.5536 | 1.265 | 0.3392 | 1.349 | 3.73e-05 | 0.0008022 | 0.4272 | -1.227 | -5.269 | 0.001594 | 0.01439 | 0.5548 | -0.8501 | -3.65 |  |
| Stc1 | stanniocalcin 1 | 0.001721 | 0.03186 | 2.066 | 1.047 | 3.617 | 0.4012 | 0.7281 | 1.226 | 0.2938 | 0.8578 | 6.344e-05 | 0.0012 | 3.025 | 1.597 | 5.036 | 0.0496 | 0.1753 | 1.583 | 0.6627 | 2.09 |  |
| Slamf7 | SLAM family member 7 | 0.0006614 | 0.01753 | 2.41 | 1.269 | 4.027 | 0.03738 | 0.2713 | 1.779 | 0.8313 | 2.23 | 0.1954 | 0.4301 | 1.378 | 0.4624 | 1.34 | 0.02296 | 0.1021 | 1.803 | 0.8502 | 2.463 |  |
| Slc9a3r1 | solute carrier family 9 (sodiu | 0.3504 | 0.6477 | 1.184 | 0.2435 | 0.9562 | 0.06326 | 0.3468 | 0.6631 | -0.5926 | -1.967 | 0.001537 | 0.01425 | 2.032 | 1.023 | 3.666 | 3.277e-05 | 0.0007419 | 2.801 | 1.486 | 5.326 |  |
| Mmp11 | matrix metallopeptidase 11 | 0.002144 | 0.03638 | 1.536 | 0.619 | 3.522 | 0.8036 | 0.9311 | 1.037 | 0.0524 | 0.252 | 8.739e-07 | 4.52e-05 | 2.543 | 1.347 | 6.995 | 0.08149 | 0.2443 | 1.277 | 0.3532 | 1.835 |  |
| Gm25121 | predicted gene, 25121 | 0.1051 | 0.3503 | 0.7505 | -0.4141 | -1.698 | 0.003003 | 0.06286 | 0.5089 | -0.9746 | -3.376 | 0.0005723 | 0.006634 | 2.133 | 1.093 | 4.089 | 0.03952 | 0.1498 | 1.504 | 0.5886 | 2.203 |  |
| Clca3a1 | chloride channel accessory 3A1 | 0.0004721 | 0.01422 | 2.718 | 1.442 | 4.171 | 0.008368 | 0.119 | 2.292 | 1.197 | 2.926 | 0.6917 | 0.853 | 0.8997 | -0.1524 | -0.4024 | 0.02939 | 0.1218 | 1.851 | 0.8887 | 2.346 |  |
| Spry1 | sprouty RTK signaling antagoni | 0.3144 | 0.6161 | 0.8969 | -0.1569 | -1.032 | 0.06485 | 0.3495 | 1.276 | 0.3516 | 1.954 | 0.03024 | 0.1267 | 0.7639 | -0.3886 | -2.333 | 1.244e-06 | 5.841e-05 | 0.4547 | -1.137 | -6.825 |  |
| Snord1c | small nucleolar RNA, C/D box 1 | 0.0001454 | 0.006838 | 2.013 | 1.01 | 4.677 | 0.0003928 | 0.01557 | 2.122 | 1.085 | 4.25 | 0.4762 | 0.7116 | 0.8878 | -0.1717 | -0.726 | 0.8143 | 0.9131 | 0.9617 | -0.05627 | -0.238 |  |
| Gm24253 | predicted gene, 24253 | 0.0001454 | 0.006838 | 2.013 | 1.01 | 4.677 | 0.0003928 | 0.01557 | 2.122 | 1.085 | 4.25 | 0.4762 | 0.7116 | 0.8878 | -0.1717 | -0.726 | 0.8143 | 0.9131 | 0.9617 | -0.05627 | -0.238 |  |
| Nr0b2 | nuclear receptor subfamily 0, | 0.01477 | 0.1154 | 0.5655 | -0.8225 | -2.668 | 0.001821 | 0.04551 | 0.4032 | -1.31 | -3.593 | 0.02519 | 0.1115 | 1.762 | 0.8171 | 2.42 | 0.06993 | 0.2217 | 1.565 | 0.6466 | 1.915 |  |
| Bzw2 | basic leucine zipper and W2 do | 0.04213 | 0.2139 | 1.287 | 0.3641 | 2.171 | 0.4348 | 0.7495 | 1.116 | 0.1581 | 0.797 | 2.891e-06 | 0.0001112 | 2.266 | 1.18 | 6.426 | 0.006151 | 0.03933 | 0.6771 | -0.5625 | -3.062 |  |
| Prr32 | proline rich 32 | 0.001639 | 0.03079 | 0.5279 | -0.9217 | -3.638 | 0.2934 | 0.649 | 0.7991 | -0.3235 | -1.079 | 1.87e-05 | 0.0004705 | 0.3422 | -1.547 | -5.575 | 0.3829 | 0.6167 | 0.8423 | -0.2476 | -0.8923 |  |
| Gfod1 | glucose-fructose oxidoreductas | 0.05214 | 0.2407 | 1.269 | 0.3433 | 2.065 | 0.3057 | 0.6594 | 0.8665 | -0.2068 | -1.051 | 2.85e-06 | 0.0001097 | 2.252 | 1.171 | 6.433 | 0.01609 | 0.0791 | 1.394 | 0.4788 | 2.629 |  |
| Fmr1 | fragile X mental retardation 1 | 0.03175 | 0.1818 | 0.6374 | -0.6498 | -2.309 | 0.3909 | 0.7201 | 1.224 | 0.292 | 0.877 | 1.106e-06 | 5.339e-05 | 0.2299 | -2.121 | -6.882 | 0.03343 | 0.1333 | 0.6138 | -0.7041 | -2.284 |  |
| Mir100 | microRNA 100 | 0.06913 | 0.2805 | 0.7778 | -0.3625 | -1.921 | 0.2734 | 0.6305 | 1.19 | 0.2515 | 1.126 | 0.001239 | 0.01204 | 0.5836 | -0.777 | -3.758 | 4.876e-05 | 0.001019 | 0.478 | -1.065 | -5.151 |  |
| Fam53a | family with sequence similarit | 0.08642 | 0.3157 | 0.8192 | -0.2877 | -1.803 | 0.004452 | 0.08159 | 0.6575 | -0.605 | -3.205 | 1.244e-05 | 0.0003476 | 2.009 | 1.006 | 5.757 | 0.7588 | 0.8843 | 0.963 | -0.05441 | -0.3113 |  |
| Alkbh2 | alkB homolog 2, alpha-ketoglut | 0.09764 | 0.3374 | 1.208 | 0.2726 | 1.738 | 0.2072 | 0.5697 | 0.8456 | -0.2419 | -1.303 | 6.831e-06 | 0.0002202 | 2.051 | 1.036 | 6.029 | 0.007857 | 0.0471 | 1.422 | 0.5076 | 2.954 |  |
| Dhrs3 | dehydrogenase/reductase (SDR f | 0.2815 | 0.5863 | 0.8888 | -0.17 | -1.107 | 0.02065 | 0.197 | 0.7286 | -0.4569 | -2.513 | 0.1554 | 0.3742 | 0.8418 | -0.2485 | -1.477 | 2.041e-06 | 8.605e-05 | 2.157 | 1.109 | 6.59 |  |
| Rhbdf2 | rhomboid 5 homolog 2 | 0.07756 | 0.2981 | 1.251 | 0.3235 | 1.861 | 0.2377 | 0.5975 | 0.8407 | -0.2504 | -1.217 | 0.01648 | 0.08183 | 1.413 | 0.4986 | 2.618 | 2.913e-06 | 0.0001102 | 2.335 | 1.223 | 6.423 |  |
| Gm14719 | predicted gene 14719 | 0.002304 | 0.03768 | 0.6156 | -0.7 | -3.491 | 0.9449 | 0.9824 | 0.9886 | -0.01661 | -0.07 | 1.586e-06 | 6.926e-05 | 0.3601 | -1.474 | -6.709 | 0.01623 | 0.07959 | 0.6706 | -0.5766 | -2.625 |  |
| Sh3d21 | SH3 domain containing 21 | 0.002359 | 0.03809 | 1.467 | 0.5527 | 3.481 | 0.7077 | 0.8917 | 0.9517 | -0.07144 | -0.3803 | 1.307e-09 | 3.203e-07 | 3.564 | 1.834 | 10.54 | 0.826 | 0.9194 | 1.027 | 0.03875 | 0.2228 |  |
| Gm4297 | predicted gene 4297 | 0.5111 | 0.7684 | 0.8501 | -0.2342 | -0.669 | 0.00656 | 0.1027 | 0.4185 | -1.257 | -3.034 | 0.001112 | 0.01107 | 2.75 | 1.459 | 3.805 | 0.0004478 | 0.00555 | 3.05 | 1.609 | 4.194 |  |
| Slc10a2 | solute carrier family 10, memb | 0.1346 | 0.4029 | 0.7404 | -0.4336 | -1.559 | 0.003439 | 0.06858 | 0.4693 | -1.092 | -3.318 | 0.0009489 | 0.00976 | 2.265 | 1.18 | 3.873 | 0.01687 | 0.08178 | 1.734 | 0.7942 | 2.607 |  |
| Gm9847 | predicted pseudogene 9847 | 0.1171 | 0.3727 | 0.6532 | -0.6144 | -1.638 | 0.003423 | 0.06839 | 0.3601 | -1.474 | -3.32 | 0.000116 | 0.001938 | 3.897 | 1.962 | 4.775 | 0.15 | 0.3585 | 1.532 | 0.6153 | 1.497 |  |
| Ets2 | E26 avian leukemia oncogene 2, | 0.1582 | 0.4368 | 0.8638 | -0.2113 | -1.466 | 0.1371 | 0.4795 | 1.201 | 0.2642 | 1.549 | 4.3e-09 | 8.133e-07 | 0.3412 | -1.551 | -9.825 | 0.4379 | 0.6633 | 1.091 | 0.125 | 0.7917 |  |
| Gphn | gephyrin | 0.05129 | 0.2383 | 1.168 | 0.2243 | 2.073 | 0.2107 | 0.5726 | 0.8916 | -0.1655 | -1.293 | 5.433e-09 | 9.982e-07 | 2.216 | 1.148 | 9.689 | 0.8592 | 0.9355 | 1.015 | 0.0213 | 0.1797 |  |
| Neb | nebulin | 0.344 | 0.6423 | 0.8668 | -0.2063 | -0.9692 | 0.004847 | 0.08613 | 0.5753 | -0.7976 | -3.168 | 0.0001846 | 0.002773 | 2.094 | 1.066 | 4.574 | 0.006536 | 0.04116 | 1.633 | 0.7077 | 3.036 |  |
| Rdh13 | retinol dehydrogenase 13 (all- | 0.8675 | 0.9508 | 1.019 | 0.02658 | 0.1691 | 0.007722 | 0.114 | 1.465 | 0.551 | 2.961 | 0.004399 | 0.03106 | 0.6816 | -0.553 | -3.21 | 8.617e-06 | 0.0002622 | 0.493 | -1.02 | -5.923 |  |
| Fgf21 | fibroblast growth factor 21 | 0.2984 | 0.6019 | 1.194 | 0.2558 | 1.068 | 0.004946 | 0.08656 | 1.86 | 0.8953 | 3.159 | 0.005414 | 0.03616 | 0.5671 | -0.8184 | -3.119 | 0.0004499 | 0.005563 | 0.4665 | -1.1 | -4.192 |  |
| Nutf2-ps1 | nuclear transport factor 2, ps | 0.01581 | 0.1208 | 0.7515 | -0.4121 | -2.637 | 0.5721 | 0.8244 | 1.076 | 0.1062 | 0.5745 | 8.472e-06 | 0.0002597 | 0.4947 | -1.015 | -5.931 | 0.005014 | 0.0339 | 0.6879 | -0.5397 | -3.153 |  |
| Nutf2-ps2 | nuclear transport factor 2, ps | 0.01581 | 0.1208 | 0.7515 | -0.4121 | -2.637 | 0.5721 | 0.8244 | 1.076 | 0.1062 | 0.5745 | 8.472e-06 | 0.0002597 | 0.4947 | -1.015 | -5.931 | 0.005014 | 0.0339 | 0.6879 | -0.5397 | -3.153 |  |
| Il13ra1 | interleukin 13 receptor, alpha | 0.8514 | 0.9444 | 1.028 | 0.04008 | 0.1897 | 0.01311 | 0.1522 | 0.6239 | -0.6806 | -2.723 | 0.003045 | 0.02366 | 1.717 | 0.78 | 3.371 | 7.734e-06 | 0.0002429 | 2.606 | 1.382 | 5.972 |  |
| Pstpip2 | proline-serine-threonine phosp | 0.07419 | 0.2912 | 0.7549 | -0.4056 | -1.884 | 0.003713 | 0.07202 | 0.56 | -0.8366 | -3.284 | 0.4841 | 0.7176 | 1.124 | 0.1681 | 0.7129 | 0.0001174 | 0.001979 | 2.181 | 1.125 | 4.769 |  |
| Zdhhc14 | zinc finger, DHHC domain conta | 0.1034 | 0.3477 | 1.235 | 0.3043 | 1.707 | 0.1997 | 0.5623 | 0.8237 | -0.2799 | -1.326 | 0.005718 | 0.03772 | 1.521 | 0.6046 | 3.095 | 9.545e-06 | 0.0002829 | 2.216 | 1.148 | 5.877 |  |
| Ninj1 | ninjurin 1 | 0.09617 | 0.3346 | 1.295 | 0.3731 | 1.746 | 0.2134 | 0.5747 | 0.7983 | -0.325 | -1.285 | 0.01025 | 0.05775 | 1.584 | 0.6635 | 2.835 | 5.697e-06 | 0.0001918 | 2.696 | 1.431 | 6.112 |  |
| Nbeal2 | neurobeachin-like 2 | 0.2028 | 0.4979 | 1.146 | 0.1969 | 1.317 | 0.1106 | 0.4419 | 0.8149 | -0.2954 | -1.669 | 1.063e-06 | 5.181e-05 | 2.189 | 1.13 | 6.901 | 0.04713 | 0.1692 | 1.272 | 0.3466 | 2.116 |  |
| Gpnmb | glycoprotein (transmembrane) n | 0.001042 | 0.02345 | 2.417 | 1.273 | 3.833 | 0.1032 | 0.4278 | 1.593 | 0.6713 | 1.708 | 0.01273 | 0.06789 | 1.994 | 0.996 | 2.736 | 0.03763 | 0.145 | 1.754 | 0.8105 | 2.227 |  |
| Dusp10 | dual specificity phosphatase 1 | 0.0002757 | 0.01018 | 0.4272 | -1.227 | -4.402 | 0.007197 | 0.1095 | 0.5045 | -0.9871 | -2.993 | 0.09169 | 0.268 | 0.6873 | -0.541 | -1.772 | 0.5648 | 0.7596 | 0.8835 | -0.1788 | -0.5854 |  |
| Rpl3-ps2 | ribosomal protein L3, pseudoge | 0.001905 | 0.03397 | 1.858 | 0.8937 | 3.573 | 0.3367 | 0.6819 | 1.224 | 0.2913 | 0.9845 | 0.01959 | 0.09304 | 1.619 | 0.6953 | 2.538 | 0.000615 | 0.007056 | 2.161 | 1.112 | 4.058 |  |
| Eva1c | eva-1 homolog C (C. elegans) | 0.00196 | 0.03451 | 1.468 | 0.5538 | 3.561 | 0.5579 | 0.8177 | 1.079 | 0.1097 | 0.5959 | 9.636e-07 | 4.879e-05 | 2.272 | 1.184 | 6.948 | 0.3928 | 0.6258 | 1.109 | 0.1488 | 0.8735 |  |
| Mir1a-2 | microRNA 1a-2 | 0.001417 | 0.0283 | 0.4544 | -1.138 | -3.701 | 0.864 | 0.9541 | 0.9572 | -0.06312 | -0.1735 | 0.7805 | 0.9003 | 1.068 | 0.09516 | 0.2825 | 4.088e-09 | 7.252e-07 | 0.1001 | -3.32 | -9.855 |  |
| Mlycd | malonyl-CoA decarboxylase | 0.002196 | 0.0367 | 1.41 | 0.4958 | 3.512 | 0.7743 | 0.9185 | 1.034 | 0.04854 | 0.2906 | 0.1291 | 0.3334 | 1.185 | 0.2448 | 1.583 | 7.138e-07 | 3.695e-05 | 2.139 | 1.097 | 7.094 |  |
| Pcdhga7 | protocadherin gamma subfamily | 0.01482 | 0.1156 | 0.6285 | -0.6699 | -2.667 | 0.002012 | 0.04878 | 0.4813 | -1.055 | -3.55 | 0.1293 | 0.3336 | 1.352 | 0.4354 | 1.582 | 0.01654 | 0.08066 | 1.647 | 0.7199 | 2.616 |  |
| Sult1a1 | sulfotransferase family 1A, ph | 0.0003525 | 0.01191 | 2.092 | 1.065 | 4.297 | 0.009964 | 0.1308 | 1.784 | 0.8351 | 2.847 | 0.2329 | 0.4792 | 1.261 | 0.334 | 1.23 | 0.205 | 0.4318 | 1.28 | 0.3558 | 1.31 |  |
| Actn1 | actinin, alpha 1 | 0.1223 | 0.3819 | 1.287 | 0.3639 | 1.614 | 0.1903 | 0.551 | 0.7782 | -0.3618 | -1.356 | 0.001901 | 0.01662 | 1.844 | 0.883 | 3.574 | 3.998e-05 | 0.0008665 | 2.452 | 1.294 | 5.238 |  |
| Pmepa1 | prostate transmembrane protein | 0.8825 | 0.9558 | 0.9757 | -0.03555 | -0.1497 | 0.005144 | 0.08874 | 0.5424 | -0.8826 | -3.141 | 2.819e-08 | 3.237e-06 | 4.852 | 2.279 | 8.76 | 0.3249 | 0.5651 | 1.2 | 0.2626 | 1.009 |  |
| Syt7 | synaptotagmin VII | 0.01227 | 0.1036 | 1.329 | 0.4105 | 2.753 | 0.4734 | 0.7714 | 0.9145 | -0.1289 | -0.7307 | 7.015e-09 | 1.176e-06 | 2.945 | 1.558 | 9.54 | 0.7171 | 0.8608 | 1.042 | 0.06002 | 0.3675 |  |
| Agmo | alkylglycerol monooxygenase | 0.002215 | 0.03689 | 1.532 | 0.615 | 3.508 | 0.6039 | 0.8423 | 1.079 | 0.1094 | 0.5272 | 1.729e-05 | 0.0004433 | 2.11 | 1.077 | 5.61 | 0.03305 | 0.1323 | 1.356 | 0.4397 | 2.29 |  |
| Ssx2ip | synovial sarcoma, X 2 interact | 0.01785 | 0.1296 | 0.7539 | -0.4076 | -2.581 | 0.5324 | 0.8037 | 1.086 | 0.1187 | 0.6353 | 0.02267 | 0.1031 | 0.7437 | -0.4272 | -2.47 | 1.766e-06 | 7.649e-05 | 0.4501 | -1.152 | -6.658 |  |
| Snx7 | sorting nexin 7 | 0.1877 | 0.4787 | 0.8364 | -0.2577 | -1.364 | 0.1266 | 0.4668 | 1.28 | 0.3563 | 1.594 | 9.073e-06 | 0.0002714 | 0.429 | -1.221 | -5.9 | 0.00705 | 0.04362 | 0.6501 | -0.6212 | -3.002 |  |
| Sec61b | Sec61 beta subunit | 0.0003103 | 0.011 | 0.5174 | -0.9506 | -4.351 | 0.0008391 | 0.02701 | 0.4949 | -1.015 | -3.925 | 0.2951 | 0.5501 | 1.195 | 0.2573 | 1.075 | 0.7888 | 0.9003 | 0.956 | -0.06498 | -0.2715 |  |
| Trim7 | tripartite motif-containing 7 | 0.1953 | 0.4885 | 0.8605 | -0.2167 | -1.34 | 0.07753 | 0.3817 | 1.28 | 0.3561 | 1.861 | 0.7389 | 0.8796 | 0.9594 | -0.05986 | -0.3379 | 9.579e-09 | 1.4e-06 | 0.3168 | -1.658 | -9.362 |  |
| Syne3 | spectrin repeat containing, nu | 0.002423 | 0.03874 | 1.539 | 0.6219 | 3.47 | 0.937 | 0.9795 | 1.012 | 0.01698 | 0.08007 | 1.549e-06 | 6.824e-05 | 2.496 | 1.32 | 6.72 | 0.02027 | 0.09344 | 1.41 | 0.4952 | 2.522 |  |
| Gm7676 | predicted gene 7676 | 0.02706 | 0.1654 | 1.46 | 0.5459 | 2.386 | 0.4709 | 0.7699 | 0.8712 | -0.199 | -0.7349 | 0.0002651 | 0.003651 | 2.155 | 1.108 | 4.419 | 0.0002212 | 0.003239 | 2.184 | 1.127 | 4.496 |  |
| Gm6194 | NA | 0.4677 | 0.7396 | 1.114 | 0.1558 | 0.7404 | 0.006273 | 0.09957 | 1.694 | 0.7604 | 3.054 | 1.064e-05 | 0.0003082 | 0.3941 | -1.343 | -5.828 | 0.05478 | 0.1876 | 0.7218 | -0.4703 | -2.04 |  |
| Gm6030 | predicted gene 6030 | 0.7987 | 0.9215 | 0.9685 | -0.04623 | -0.2585 | 0.008319 | 0.1187 | 0.6508 | -0.6197 | -2.928 | 2.424e-05 | 0.0005792 | 2.099 | 1.07 | 5.459 | 0.003409 | 0.02558 | 1.57 | 0.6507 | 3.321 |  |
| Coro2b | coronin, actin binding protein | 0.108 | 0.356 | 1.18 | 0.2392 | 1.683 | 0.1859 | 0.5471 | 0.8524 | -0.2304 | -1.37 | 2.406e-07 | 1.673e-05 | 2.279 | 1.189 | 7.633 | 0.1725 | 0.3901 | 1.165 | 0.2203 | 1.415 |  |
| Fads2 | fatty acid desaturase 2 | 0.1868 | 0.4777 | 0.8279 | -0.2724 | -1.367 | 0.1387 | 0.4827 | 1.287 | 0.3636 | 1.542 | 0.0001164 | 0.001942 | 0.4857 | -1.042 | -4.773 | 0.0007601 | 0.008281 | 0.5486 | -0.8661 | -3.968 |  |
| Apln | apelin | 0.002653 | 0.04075 | 0.6168 | -0.6971 | -3.43 | 0.9995 | 0.9998 | 0.9999 | -0.00015 | -0.0006238 | 1.981e-06 | 8.226e-05 | 0.3609 | -1.47 | -6.604 | 0.01135 | 0.06193 | 0.6503 | -0.6208 | -2.788 |  |
| Klhl23 | kelch-like 23 | 0.7175 | 0.8854 | 1.046 | 0.06544 | 0.367 | 0.007712 | 0.1139 | 1.542 | 0.6249 | 2.962 | 0.006095 | 0.03956 | 0.6602 | -0.5989 | -3.067 | 2.084e-05 | 0.0005216 | 0.4732 | -1.079 | -5.527 |  |
| Dnaja1 | DnaJ heat shock protein family | 0.4949 | 0.7574 | 0.9257 | -0.1114 | -0.6952 | 0.04287 | 0.289 | 1.329 | 0.4102 | 2.163 | 1.259e-05 | 0.000351 | 0.4965 | -1.01 | -5.752 | 0.004932 | 0.0335 | 0.6807 | -0.5549 | -3.16 |  |
| Stat3 | signal transducer and activato | 0.05102 | 0.2378 | 1.275 | 0.3502 | 2.076 | 0.3318 | 0.6783 | 0.8714 | -0.1986 | -0.9947 | 7.623e-06 | 0.00024 | 2.151 | 1.105 | 5.979 | 0.008301 | 0.04909 | 1.455 | 0.5414 | 2.929 |  |
| Hgf | hepatocyte growth factor | 0.8989 | 0.9623 | 1.017 | 0.0243 | 0.1287 | 0.005466 | 0.09183 | 1.62 | 0.6958 | 3.115 | 2.605e-08 | 3.161e-06 | 0.2831 | -1.821 | -8.803 | 0.3598 | 0.5971 | 0.8743 | -0.1939 | -0.9374 |  |
| Marcks | myristoylated alanine rich pro | 0.0005507 | 0.01567 | 0.4899 | -1.029 | -4.106 | 0.02469 | 0.2164 | 0.6068 | -0.7206 | -2.429 | 0.01523 | 0.07728 | 0.6033 | -0.729 | -2.654 | 0.4812 | 0.6976 | 0.8723 | -0.1971 | -0.7178 |  |
| Gm13275 | predicted gene 13275 | 0.01828 | 0.1314 | 1.328 | 0.4093 | 2.57 | 0.4669 | 0.7684 | 0.9077 | -0.1397 | -0.7417 | 4.432e-08 | 4.436e-06 | 2.8 | 1.485 | 8.515 | 0.3649 | 0.6007 | 1.119 | 0.1618 | 0.9272 |  |
| Gm13276 | predicted gene 13276 | 0.01828 | 0.1314 | 1.328 | 0.4093 | 2.57 | 0.4669 | 0.7684 | 0.9077 | -0.1397 | -0.7417 | 4.432e-08 | 4.436e-06 | 2.8 | 1.485 | 8.515 | 0.3649 | 0.6007 | 1.119 | 0.1618 | 0.9272 |  |
| Gm13277 | predicted gene 13277 | 0.01828 | 0.1314 | 1.328 | 0.4093 | 2.57 | 0.4669 | 0.7684 | 0.9077 | -0.1397 | -0.7417 | 4.432e-08 | 4.436e-06 | 2.8 | 1.485 | 8.515 | 0.3649 | 0.6007 | 1.119 | 0.1618 | 0.9272 |  |
| Gm13278 | predicted gene 13278 | 0.01828 | 0.1314 | 1.328 | 0.4093 | 2.57 | 0.4669 | 0.7684 | 0.9077 | -0.1397 | -0.7417 | 4.432e-08 | 4.436e-06 | 2.8 | 1.485 | 8.515 | 0.3649 | 0.6007 | 1.119 | 0.1618 | 0.9272 |  |
| Gm13279 | predicted gene 13279 | 0.01828 | 0.1314 | 1.328 | 0.4093 | 2.57 | 0.4669 | 0.7684 | 0.9077 | -0.1397 | -0.7417 | 4.432e-08 | 4.436e-06 | 2.8 | 1.485 | 8.515 | 0.3649 | 0.6007 | 1.119 | 0.1618 | 0.9272 |  |
| Gm13285 | predicted gene 13285 | 0.01828 | 0.1314 | 1.328 | 0.4093 | 2.57 | 0.4669 | 0.7684 | 0.9077 | -0.1397 | -0.7417 | 4.432e-08 | 4.436e-06 | 2.8 | 1.485 | 8.515 | 0.3649 | 0.6007 | 1.119 | 0.1618 | 0.9272 |  |
| Rbfox2 | RNA binding protein, fox-1 hom | 0.02339 | 0.1515 | 0.8046 | -0.3136 | -2.455 | 0.326 | 0.6736 | 1.111 | 0.1522 | 1.007 | 0.9903 | 0.997 | 1.001 | 0.001728 | 0.01235 | 3.21e-09 | 6.162e-07 | 0.3792 | -1.399 | -9.998 |  |
| Tmem100 | transmembrane protein 100 | 0.94 | 0.9787 | 1.016 | 0.02303 | 0.07621 | 0.006703 | 0.1042 | 2.116 | 1.081 | 3.024 | 1.081e-07 | 9.031e-06 | 0.158 | -2.662 | -8.043 | 0.1373 | 0.3378 | 0.701 | -0.5125 | -1.548 |  |
| Lrrc28 | leucine rich repeat containing | 0.6481 | 0.8496 | 1.045 | 0.0636 | 0.4633 | 0.007361 | 0.1109 | 1.399 | 0.4845 | 2.983 | 0.1129 | 0.3063 | 0.8413 | -0.2493 | -1.658 | 1.491e-06 | 6.707e-05 | 0.4954 | -1.013 | -6.739 |  |
| Ostn | osteocrin | 0.002377 | 0.03831 | 0.493 | -1.02 | -3.478 | 0.1848 | 0.5461 | 0.7186 | -0.4768 | -1.374 | 0.842 | 0.9324 | 1.046 | 0.06489 | 0.2019 | 3.675e-05 | 0.0008129 | 0.3088 | -1.695 | -5.275 |  |
| Gm13080 | predicted gene 13080 | 0.002169 | 0.03649 | 0.6573 | -0.6053 | -3.517 | 0.4555 | 0.761 | 0.8981 | -0.155 | -0.7611 | 3.308e-05 | 0.0007407 | 0.4989 | -1.003 | -5.322 | 0.07994 | 0.2413 | 0.7858 | -0.3478 | -1.845 |  |
| Pglyrp1 | peptidoglycan recognition prot | 0.0005621 | 0.01586 | 2.1 | 1.07 | 4.097 | 0.02015 | 0.1944 | 1.718 | 0.7803 | 2.525 | 0.2007 | 0.4373 | 1.3 | 0.3786 | 1.323 | 0.1017 | 0.2809 | 1.405 | 0.4909 | 1.716 |  |
| Ncbp2 | nuclear cap binding protein su | 0.8307 | 0.936 | 1.028 | 0.03943 | 0.2166 | 0.008512 | 0.1201 | 1.546 | 0.6285 | 2.918 | 4.552e-06 | 0.00016 | 0.4236 | -1.239 | -6.215 | 0.01549 | 0.07697 | 0.6936 | -0.5278 | -2.647 |  |
| Cd86 | CD86 antigen | 0.001877 | 0.03369 | 2.422 | 1.276 | 3.58 | 0.266 | 0.6234 | 1.398 | 0.4829 | 1.144 | 0.02126 | 0.0984 | 1.968 | 0.9765 | 2.5 | 0.001811 | 0.01595 | 2.647 | 1.404 | 3.595 |  |
| Chst11 | carbohydrate sulfotransferase | 0.02101 | 0.142 | 1.367 | 0.4512 | 2.505 | 0.4896 | 0.7807 | 0.9012 | -0.15 | -0.704 | 2.716e-07 | 1.848e-05 | 2.817 | 1.494 | 7.572 | 0.1322 | 0.3303 | 1.239 | 0.3097 | 1.57 |  |
| Ddit3 | DNA-damage inducible transcrip | 0.7367 | 0.894 | 0.9429 | -0.0849 | -0.341 | 0.02054 | 0.1967 | 1.672 | 0.7412 | 2.516 | 0.003244 | 0.02479 | 0.5315 | -0.9118 | -3.343 | 1.284e-05 | 0.0003605 | 0.3377 | -1.566 | -5.743 |  |
| Ubtd2 | ubiquitin domain containing 2 | 0.00257 | 0.04003 | 0.6228 | -0.6832 | -3.444 | 0.6998 | 0.8877 | 0.9383 | -0.09181 | -0.3912 | 0.03951 | 0.1525 | 0.7176 | -0.4787 | -2.203 | 7.924e-06 | 0.0002465 | 0.4074 | -1.295 | -5.961 |  |
| A830035O19Rik | RIKEN cDNA A830035O19 gene | 0.0859 | 0.3147 | 1.251 | 0.3227 | 1.807 | 0.2534 | 0.6133 | 0.8418 | -0.2485 | -1.176 | 0.002694 | 0.02163 | 1.591 | 0.6698 | 3.424 | 3.212e-05 | 0.0007311 | 2.062 | 1.044 | 5.335 |  |
| Fbxo30 | F-box protein 30 | 0.003317 | 0.04685 | 1.396 | 0.4811 | 3.333 | 0.7435 | 0.9057 | 1.04 | 0.05666 | 0.3317 | 0.549 | 0.7662 | 0.9354 | -0.0964 | -0.6096 | 3.364e-08 | 3.698e-06 | 2.584 | 1.37 | 8.663 |  |
| Lrp11 | low density lipoprotein recept | 0.3242 | 0.6245 | 0.8934 | -0.1626 | -1.011 | 0.0758 | 0.3773 | 1.28 | 0.3564 | 1.873 | 4.547e-06 | 0.00016 | 0.4681 | -1.095 | -6.216 | 0.01637 | 0.08005 | 0.7261 | -0.4618 | -2.621 |  |
| Mettl24 | methyltransferase like 24 | 0.1243 | 0.385 | 0.8309 | -0.2673 | -1.604 | 0.1942 | 0.5557 | 1.202 | 0.2649 | 1.344 | 1.234e-05 | 0.0003462 | 0.4825 | -1.051 | -5.761 | 0.007304 | 0.0447 | 0.6854 | -0.5451 | -2.986 |  |
| 1600014C10Rik | RIKEN cDNA 1600014C10 gene | 0.3372 | 0.6365 | 0.8897 | -0.1685 | -0.9833 | 0.0676 | 0.3571 | 0.7621 | -0.3919 | -1.933 | 5.078e-06 | 0.0001749 | 2.231 | 1.157 | 6.165 | 0.0173 | 0.08319 | 0.7133 | -0.4873 | -2.596 |  |
| 6430548M08Rik | RIKEN cDNA 6430548M08 gene | 0.01212 | 0.1027 | 1.459 | 0.5451 | 2.759 | 0.5926 | 0.8355 | 0.9156 | -0.1271 | -0.5438 | 0.3511 | 0.6053 | 1.154 | 0.2066 | 0.9547 | 5.894e-08 | 5.716e-06 | 3.507 | 1.81 | 8.362 |  |
| Stard5 | StAR-related lipid transfer (S | 0.0008183 | 0.0199 | 2.222 | 1.152 | 3.936 | 0.009695 | 0.1289 | 1.986 | 0.99 | 2.86 | 0.04541 | 0.1671 | 1.607 | 0.684 | 2.134 | 0.5417 | 0.7429 | 0.8711 | -0.199 | -0.6209 |  |
| Camkmt | calmodulin-lysine N-methyltran | 0.9707 | 0.9895 | 0.995 | -0.007247 | -0.03723 | 0.01158 | 0.1422 | 1.558 | 0.6401 | 2.779 | 4.75e-05 | 0.0009673 | 0.4662 | -1.101 | -5.163 | 0.0008007 | 0.008621 | 0.5581 | -0.8413 | -3.945 |  |
| Camk2n1 | calcium/calmodulin-dependent p | 0.101 | 0.3433 | 1.251 | 0.3232 | 1.719 | 0.2321 | 0.5935 | 0.827 | -0.2741 | -1.232 | 1.99e-05 | 0.0004921 | 2.207 | 1.142 | 5.547 | 0.004993 | 0.03382 | 1.569 | 0.6496 | 3.154 |  |
| Inpp5d | inositol polyphosphate-5-phosp | 0.001247 | 0.02634 | 2.599 | 1.378 | 3.756 | 0.09153 | 0.4073 | 1.705 | 0.7695 | 1.773 | 0.05497 | 0.19 | 1.764 | 0.8192 | 2.038 | 0.02112 | 0.09617 | 2.008 | 1.006 | 2.503 |  |
| Mir692-3 | microRNA 692-3 | 0.1213 | 0.3803 | 1.192 | 0.2535 | 1.618 | 0.1711 | 0.5293 | 0.8333 | -0.2631 | -1.42 | 0.4808 | 0.715 | 1.089 | 0.1233 | 0.7185 | 6.459e-08 | 6.019e-06 | 2.688 | 1.427 | 8.314 |  |
| Ganc | glucosidase, alpha; neutral C | 0.06933 | 0.2811 | 0.8055 | -0.312 | -1.919 | 0.2668 | 0.6241 | 1.164 | 0.2197 | 1.142 | 0.1846 | 0.4154 | 0.844 | -0.2447 | -1.374 | 2.655e-07 | 1.716e-05 | 0.3922 | -1.35 | -7.584 |  |
| Tbl2 | transducin (beta)-like 2 | 0.9354 | 0.9772 | 1.007 | 0.01068 | 0.08207 | 0.007443 | 0.1114 | 1.374 | 0.4584 | 2.978 | 1.176e-07 | 9.449e-06 | 0.4537 | -1.14 | -8 | 0.1697 | 0.3864 | 0.8687 | -0.2031 | -1.425 |  |
| Mettl20 | NA | 0.007922 | 0.08042 | 1.445 | 0.531 | 2.95 | 0.7795 | 0.9204 | 0.959 | -0.06043 | -0.2837 | 7.865e-06 | 0.0002449 | 2.26 | 1.176 | 5.965 | 0.006563 | 0.04127 | 1.514 | 0.5982 | 3.034 |  |
| Dnajc12 | DnaJ heat shock protein family | 0.8689 | 0.951 | 0.9799 | -0.02935 | -0.1673 | 0.01606 | 0.17 | 1.46 | 0.5461 | 2.63 | 0.001556 | 0.01438 | 0.614 | -0.7036 | -3.66 | 3.325e-05 | 0.0007516 | 0.4922 | -1.023 | -5.319 |  |
| Setmar | SET domain without mariner tra | 0.9552 | 0.9841 | 0.9909 | -0.01317 | -0.05686 | 0.01076 | 0.1364 | 1.707 | 0.7711 | 2.813 | 0.0243 | 0.1084 | 0.6514 | -0.6185 | -2.437 | 1.225e-06 | 5.802e-05 | 0.3006 | -1.734 | -6.833 |  |
| Myod1 | myogenic differentiation 1 | 0.001371 | 0.02772 | 0.554 | -0.8521 | -3.715 | 0.9959 | 0.9991 | 0.999 | -0.001414 | -0.00521 | 0.7867 | 0.9034 | 1.049 | 0.06891 | 0.2743 | 1.567e-09 | 3.36e-07 | 0.1626 | -2.621 | -10.43 |  |
| Plk3 | polo like kinase 3 | 0.1914 | 0.4836 | 0.7567 | -0.4022 | -1.352 | 0.004738 | 0.08487 | 0.4607 | -1.118 | -3.178 | 0.001998 | 0.0173 | 2.231 | 1.158 | 3.553 | 0.006464 | 0.04082 | 1.987 | 0.9906 | 3.041 |  |
| Mpeg1 | macrophage expressed gene 1 | 0.003299 | 0.04671 | 3.072 | 1.619 | 3.336 | 0.8427 | 0.9474 | 1.083 | 0.1154 | 0.201 | 0.0002096 | 0.003062 | 5.289 | 2.403 | 4.52 | 0.0007663 | 0.008338 | 4.31 | 2.108 | 3.964 |  |
| Dnajb9 | DnaJ heat shock protein family | 0.002826 | 0.04237 | 1.694 | 0.7606 | 3.403 | 0.6175 | 0.8497 | 1.097 | 0.1342 | 0.5073 | 0.01913 | 0.09134 | 1.541 | 0.6242 | 2.549 | 5.163e-05 | 0.001062 | 2.387 | 1.255 | 5.126 |  |
| Ngfr | nerve growth factor receptor ( | 0.8814 | 0.9558 | 0.9805 | -0.02834 | -0.1511 | 0.01019 | 0.1321 | 0.6464 | -0.6296 | -2.837 | 0.002041 | 0.01763 | 1.656 | 0.728 | 3.544 | 4.015e-05 | 0.0008694 | 2.108 | 1.076 | 5.236 |  |
| Cln8 | ceroid-lipofuscinosis, neurona | 0.6287 | 0.8401 | 0.9267 | -0.1099 | -0.4911 | 0.03268 | 0.2529 | 1.524 | 0.6076 | 2.295 | 0.001711 | 0.0154 | 0.5407 | -0.8872 | -3.62 | 4.753e-05 | 0.000998 | 0.416 | -1.265 | -5.163 |  |
| P2ry2 | purinergic receptor P2Y, G-pro | 0.6995 | 0.8769 | 1.057 | 0.08051 | 0.3916 | 0.081 | 0.3892 | 1.363 | 0.4471 | 1.838 | 0.0137 | 0.07172 | 1.525 | 0.6088 | 2.703 | 2.643e-07 | 1.716e-05 | 0.306 | -1.709 | -7.586 |  |
| Cox7a1 | cytochrome c oxidase subunit 7 | 0.002329 | 0.03782 | 0.6712 | -0.5751 | -3.487 | 0.373 | 0.7083 | 0.884 | -0.1779 | -0.9114 | 6.74e-06 | 0.0002186 | 0.4696 | -1.09 | -6.035 | 0.5816 | 0.7717 | 0.9322 | -0.1012 | -0.5602 |  |
| Pcolce2 | procollagen C-endopeptidase en | 0.4281 | 0.7114 | 0.8558 | -0.2247 | -0.8088 | 0.007273 | 0.1103 | 0.5062 | -0.9823 | -2.988 | 0.0008122 | 0.008633 | 2.296 | 1.199 | 3.939 | 0.002115 | 0.01793 | 2.105 | 1.074 | 3.528 |  |
| Pawr | PRKC, apoptosis, WT1, regulato | 0.04016 | 0.2077 | 1.337 | 0.4192 | 2.195 | 0.4139 | 0.7362 | 0.8775 | -0.1885 | -0.8344 | 6.413e-05 | 0.00121 | 2.074 | 1.053 | 5.032 | 0.001477 | 0.01363 | 1.706 | 0.7705 | 3.683 |  |
| Rnd3 | Rho family GTPase 3 | 0.1605 | 0.4404 | 0.8342 | -0.2616 | -1.457 | 0.004863 | 0.08621 | 0.6275 | -0.6724 | -3.166 | 4.141e-05 | 0.0008668 | 2.038 | 1.027 | 5.223 | 0.3085 | 0.5493 | 1.153 | 0.2055 | 1.045 |  |
| Cic | capicua transcriptional repres | 0.9001 | 0.9628 | 1.014 | 0.02032 | 0.1272 | 0.01411 | 0.1587 | 0.703 | -0.5083 | -2.689 | 0.003954 | 0.02874 | 1.484 | 0.5699 | 3.257 | 1.117e-05 | 0.0003224 | 2.022 | 1.016 | 5.806 |  |
| Sucnr1 | succinate receptor 1 | 0.07443 | 0.2916 | 0.6897 | -0.5359 | -1.882 | 0.00341 | 0.06826 | 0.4605 | -1.119 | -3.321 | 0.007791 | 0.0473 | 1.895 | 0.9224 | 2.958 | 0.0154 | 0.0767 | 1.773 | 0.8263 | 2.649 |  |
| Hilpda | hypoxia inducible lipid drople | 0.4306 | 0.7126 | 1.125 | 0.1696 | 0.8045 | 0.007852 | 0.1149 | 1.667 | 0.7369 | 2.954 | 0.1543 | 0.3727 | 0.789 | -0.3419 | -1.481 | 9.304e-06 | 0.0002787 | 0.3896 | -1.36 | -5.888 |  |
| Acsf3 | acyl-CoA synthetase family mem | 0.006796 | 0.07386 | 0.7232 | -0.4675 | -3.018 | 0.8172 | 0.937 | 1.03 | 0.04291 | 0.2342 | 2.105e-06 | 8.621e-05 | 0.4615 | -1.116 | -6.575 | 0.02366 | 0.1046 | 0.7497 | -0.4156 | -2.449 |  |
| Ttpa | tocopherol (alpha) transfer pr | 0.08692 | 0.3168 | 1.448 | 0.534 | 1.8 | 0.003508 | 0.06936 | 2.236 | 1.161 | 3.309 | 0.005696 | 0.0376 | 0.4979 | -1.006 | -3.096 | 0.01394 | 0.07144 | 0.545 | -0.8756 | -2.695 |  |
| Sema4a | sema domain, immunoglobulin do | 0.1486 | 0.4236 | 1.264 | 0.3375 | 1.503 | 0.186 | 0.5474 | 0.777 | -0.3639 | -1.369 | 0.001645 | 0.01496 | 1.859 | 0.8947 | 3.637 | 8.363e-05 | 0.001531 | 2.313 | 1.21 | 4.916 |  |
| Nceh1 | neutral cholesterol ester hydr | 0.002792 | 0.04202 | 1.803 | 0.8503 | 3.408 | 0.001554 | 0.04155 | 2.115 | 1.081 | 3.661 | 0.203 | 0.4405 | 0.7793 | -0.3597 | -1.316 | 0.2391 | 0.4725 | 0.7946 | -0.3316 | -1.213 |  |
| Gm17167 | predicted gene 17167 | 0.01203 | 0.1024 | 1.922 | 0.9423 | 2.762 | 0.002596 | 0.0571 | 2.618 | 1.388 | 3.44 | 0.6153 | 0.8068 | 0.8761 | -0.1908 | -0.5105 | 0.00603 | 0.03874 | 0.4513 | -1.148 | -3.071 |  |
| Ahdc1 | AT hook, DNA binding motif, co | 0.09522 | 0.3328 | 0.8318 | -0.2658 | -1.751 | 0.2311 | 0.5927 | 1.166 | 0.2218 | 1.235 | 0.1359 | 0.3445 | 0.8361 | -0.2583 | -1.554 | 5.246e-07 | 2.908e-05 | 0.434 | -1.204 | -7.245 |  |
| Gm5417 | predicted gene 5417 | 0.5711 | 0.8073 | 1.088 | 0.1218 | 0.5759 | 0.008903 | 0.1228 | 1.653 | 0.7252 | 2.898 | 0.0338 | 0.1368 | 0.6935 | -0.528 | -2.279 | 2.048e-05 | 0.000516 | 0.4112 | -1.282 | -5.534 |  |
| Mt1 | metallothionein 1 | 0.0002273 | 0.009098 | 3.599 | 1.848 | 4.485 | 0.0007686 | 0.02525 | 3.815 | 1.932 | 3.963 | 0.523 | 0.7472 | 0.816 | -0.2934 | -0.6501 | 0.9289 | 0.9681 | 1.029 | 0.04076 | 0.09032 |  |
| n-R5s88 | nuclear encoded rRNA 5S 88 | 0.5485 | 0.7934 | 0.8599 | -0.2177 | -0.6104 | 0.008449 | 0.1198 | 0.4255 | -1.233 | -2.921 | 0.0003581 | 0.004632 | 3.196 | 1.676 | 4.29 | 0.002234 | 0.01868 | 2.584 | 1.369 | 3.505 |  |
| B4galt4 | UDP-Gal:betaGlcNAc beta 1,4-ga | 0.3288 | 0.6286 | 1.108 | 0.1474 | 1.001 | 0.07933 | 0.385 | 0.7999 | -0.3221 | -1.849 | 3.539e-06 | 0.0001292 | 2.03 | 1.021 | 6.332 | 0.02623 | 0.113 | 1.308 | 0.3872 | 2.401 |  |
| Ift22 | intraflagellar transport 22 | 0.04458 | 0.2202 | 0.8099 | -0.3042 | -2.143 | 0.3839 | 0.715 | 1.109 | 0.1495 | 0.8903 | 2.49e-06 | 9.915e-05 | 0.4966 | -1.01 | -6.496 | 0.03525 | 0.1384 | 0.784 | -0.3511 | -2.259 |  |
| Wdr62 | WD repeat domain 62 | 0.1416 | 0.4129 | 1.232 | 0.3007 | 1.53 | 0.1562 | 0.5094 | 0.7886 | -0.3425 | -1.474 | 0.7491 | 0.8839 | 0.9528 | -0.0698 | -0.3243 | 1.086e-08 | 1.535e-06 | 3.999 | 1.999 | 9.291 |  |
| Ormdl3 | ORM1-like 3 (S. cerevisiae) | 0.7195 | 0.8863 | 1.063 | 0.0888 | 0.3642 | 0.02181 | 0.2032 | 0.6081 | -0.7176 | -2.488 | 4.403e-07 | 2.671e-05 | 3.885 | 1.958 | 7.331 | 0.09959 | 0.2771 | 1.377 | 0.4613 | 1.727 |  |
| Rgma | repulsive guidance molecule fa | 0.009169 | 0.08781 | 0.6485 | -0.6249 | -2.885 | 0.438 | 0.7513 | 0.8688 | -0.2028 | -0.7914 | 0.2706 | 0.5235 | 1.205 | 0.2688 | 1.133 | 2.64e-06 | 0.000103 | 0.3451 | -1.535 | -6.469 |  |
| Zdhhc23 | zinc finger, DHHC domain conta | 0.0003593 | 0.01202 | 2.595 | 1.376 | 4.288 | 0.006236 | 0.09934 | 2.235 | 1.16 | 3.056 | 0.6748 | 0.844 | 1.109 | 0.1496 | 0.4258 | 0.1727 | 0.3903 | 1.411 | 0.497 | 1.414 |  |
| Apcs | serum amyloid P-component | 0.05795 | 0.2553 | 0.6636 | -0.5916 | -2.012 | 0.003905 | 0.07496 | 0.4553 | -1.135 | -3.262 | 0.07448 | 0.2333 | 1.522 | 0.6063 | 1.882 | 0.004716 | 0.03238 | 2.034 | 1.024 | 3.18 |  |
| Kdelr3 | KDEL (Lys-Asp-Glu-Leu) endopla | 0.002135 | 0.03633 | 0.5157 | -0.9555 | -3.524 | 0.3464 | 0.6893 | 0.807 | -0.3093 | -0.9643 | 0.001745 | 0.01558 | 0.4755 | -1.073 | -3.611 | 0.008561 | 0.05012 | 0.5487 | -0.8659 | -2.915 |  |
| LOC101055727 | NA | 0.466 | 0.7384 | 1.08 | 0.1107 | 0.7433 | 0.008282 | 0.1184 | 1.43 | 0.5162 | 2.93 | 0.3511 | 0.6053 | 0.8977 | -0.1557 | -0.9548 | 2.445e-06 | 9.781e-05 | 0.4794 | -1.061 | -6.505 |  |
| Cgnl1 | cingulin-like 1 | 0.5574 | 0.7986 | 1.077 | 0.1069 | 0.5967 | 0.03383 | 0.2578 | 0.7156 | -0.4828 | -2.279 | 1.69e-07 | 1.282e-05 | 2.893 | 1.533 | 7.813 | 0.2425 | 0.4764 | 1.178 | 0.2363 | 1.205 |  |
| Zfp932 | zinc finger protein 932 | 0.5807 | 0.8131 | 1.078 | 0.1087 | 0.5615 | 0.03365 | 0.2571 | 0.696 | -0.5227 | -2.281 | 0.1233 | 0.3235 | 1.267 | 0.3414 | 1.609 | 4.5e-07 | 2.584e-05 | 2.934 | 1.553 | 7.32 |  |
| Gm12653 | predicted gene 12653 | 0.000997 | 0.02272 | 0.5282 | -0.921 | -3.852 | 0.001321 | 0.03721 | 0.4811 | -1.056 | -3.731 | 0.1263 | 0.329 | 1.336 | 0.4179 | 1.595 | 0.9579 | 0.9809 | 0.9903 | -0.014 | -0.05344 |  |
| Ndufb6 | NADH:ubiquinone oxidoreductase | 0.3027 | 0.6058 | 0.8772 | -0.189 | -1.058 | 0.09169 | 0.4078 | 1.296 | 0.3745 | 1.772 | 1.047e-05 | 0.0003051 | 0.4531 | -1.142 | -5.835 | 0.01098 | 0.06032 | 0.6837 | -0.5486 | -2.803 |  |
| Utp14b | UTP14B small subunit processom | 0.01298 | 0.1074 | 0.6237 | -0.6811 | -2.727 | 0.002528 | 0.0561 | 0.4932 | -1.02 | -3.451 | 0.003021 | 0.02356 | 1.896 | 0.923 | 3.374 | 0.7375 | 0.8722 | 1.067 | 0.09297 | 0.3399 |  |
| Dexi | dexamethasone-induced transcri | 0.02875 | 0.1714 | 0.6355 | -0.654 | -2.357 | 0.003156 | 0.06515 | 0.466 | -1.102 | -3.355 | 0.001077 | 0.01078 | 2.236 | 1.161 | 3.818 | 0.5561 | 0.7539 | 1.134 | 0.182 | 0.5987 |  |
| Dusp7 | dual specificity phosphatase 7 | 0.03399 | 0.1893 | 1.479 | 0.5642 | 2.276 | 0.01185 | 0.1442 | 1.756 | 0.8121 | 2.769 | 0.0007272 | 0.007919 | 0.4722 | -1.083 | -3.987 | 0.2266 | 0.4575 | 1.265 | 0.3388 | 1.248 |  |
| Cnksr1 | connector enhancer of kinase s | 0.1396 | 0.41 | 1.208 | 0.2721 | 1.538 | 0.1647 | 0.5216 | 0.8112 | -0.3018 | -1.443 | 0.538 | 0.7583 | 1.088 | 0.1214 | 0.6266 | 7.138e-08 | 6.465e-06 | 3.032 | 1.6 | 8.261 |  |
| LOC100043921 | NA | 0.001244 | 0.02629 | 5.594 | 2.484 | 3.757 | 0.01088 | 0.1372 | 4.584 | 2.196 | 2.808 | 0.4848 | 0.7181 | 0.6995 | -0.5156 | -0.7118 | 0.07166 | 0.225 | 2.599 | 1.378 | 1.902 |  |
| Gm10382 | predicted gene 10382 | 0.576 | 0.8107 | 0.9351 | -0.09687 | -0.5686 | 0.009494 | 0.1275 | 0.6697 | -0.5784 | -2.869 | 0.1538 | 0.3721 | 1.211 | 0.2767 | 1.482 | 4.159e-06 | 0.0001482 | 2.247 | 1.168 | 6.257 |  |
| Cd163 | CD163 antigen | 0.002308 | 0.03768 | 2.1 | 1.071 | 3.491 | 0.3238 | 0.6719 | 1.29 | 0.3671 | 1.012 | 0.0005997 | 0.006871 | 2.58 | 1.367 | 4.069 | 0.03877 | 0.148 | 1.674 | 0.7432 | 2.212 |  |
| Zmat5 | zinc finger, matrin type 5 | 0.04982 | 0.235 | 0.7808 | -0.357 | -2.088 | 0.3779 | 0.7111 | 1.135 | 0.1825 | 0.9018 | 1.561e-05 | 0.0004126 | 0.4799 | -1.059 | -5.655 | 0.00733 | 0.0448 | 0.6787 | -0.5591 | -2.985 |  |
| Syk | spleen tyrosine kinase | 0.002275 | 0.03749 | 2.724 | 1.446 | 3.497 | 0.2219 | 0.5831 | 1.534 | 0.6169 | 1.261 | 0.05396 | 0.1875 | 1.902 | 0.9276 | 2.048 | 0.002576 | 0.02073 | 2.948 | 1.56 | 3.443 |  |
| Gm13286 | predicted gene 13286 | 0.00469 | 0.05823 | 1.336 | 0.4184 | 3.182 | 0.8177 | 0.9372 | 0.9751 | -0.03634 | -0.2336 | 2.394e-08 | 2.99e-06 | 2.419 | 1.275 | 8.849 | 0.5411 | 0.7427 | 1.064 | 0.08956 | 0.6217 |  |
| Mir133a-2 | microRNA 133a-2 | 0.001564 | 0.02997 | 0.5234 | -0.9339 | -3.658 | 0.9356 | 0.9792 | 0.983 | -0.02472 | -0.08184 | 0.9419 | 0.9769 | 0.9858 | -0.02063 | -0.07376 | 5.518e-09 | 9.158e-07 | 0.1531 | -2.707 | -9.68 |  |
| Apoo-ps | apolipoprotein O, pseudogene | 0.007164 | 0.07577 | 0.7652 | -0.3862 | -2.995 | 0.8187 | 0.9376 | 1.025 | 0.03543 | 0.2322 | 5.776e-07 | 3.258e-05 | 0.4943 | -1.017 | -7.197 | 0.09422 | 0.2673 | 0.842 | -0.2482 | -1.757 |  |
| Prox1 | prospero homeobox 1 | 0.004212 | 0.05402 | 2.898 | 1.535 | 3.229 | 0.001818 | 0.04549 | 4.059 | 2.021 | 3.594 | 0.03792 | 0.1484 | 0.4483 | -1.158 | -2.223 | 0.5695 | 0.7631 | 0.8116 | -0.3011 | -0.5783 |  |
| Cmss1 | cms small ribosomal subunit 1 | 0.01399 | 0.112 | 0.685 | -0.5457 | -2.693 | 0.6465 | 0.8632 | 1.08 | 0.1116 | 0.4656 | 0.1013 | 0.2858 | 0.7677 | -0.3813 | -1.718 | 6.147e-07 | 3.28e-05 | 0.332 | -1.591 | -7.167 |  |
| Prrg1 | proline rich Gla (G-carboxyglu | 0.003547 | 0.04848 | 0.6657 | -0.587 | -3.304 | 0.8378 | 0.9456 | 0.9702 | -0.0436 | -0.2074 | 0.01448 | 0.07459 | 0.6968 | -0.5211 | -2.677 | 1.416e-05 | 0.0003867 | 0.4636 | -1.109 | -5.699 |  |
| Syap1 | synapse associated protein 1 | 0.1539 | 0.431 | 0.8311 | -0.2669 | -1.482 | 0.1888 | 0.5501 | 1.223 | 0.2899 | 1.361 | 3.96e-05 | 0.000839 | 0.4883 | -1.034 | -5.243 | 0.004244 | 0.0301 | 0.6434 | -0.6363 | -3.226 |  |
| Fnip2 | folliculin interacting protein | 0.4394 | 0.72 | 1.13 | 0.1763 | 0.7889 | 0.06545 | 0.3514 | 0.6996 | -0.5153 | -1.949 | 0.0001084 | 0.00184 | 2.259 | 1.176 | 4.804 | 0.001511 | 0.01387 | 1.865 | 0.8991 | 3.673 |  |
| Gcnt1 | glucosaminyl (N-acetyl) transf | 0.001831 | 0.03316 | 2.049 | 1.035 | 3.591 | 0.1198 | 0.4556 | 1.468 | 0.5542 | 1.625 | 0.1413 | 0.353 | 1.398 | 0.4836 | 1.532 | 0.006541 | 0.04117 | 1.943 | 0.9583 | 3.035 |  |
| Tmem144 | transmembrane protein 144 | 0.6474 | 0.8494 | 0.9233 | -0.1152 | -0.4644 | 0.0105 | 0.1349 | 0.5631 | -0.8287 | -2.824 | 0.07687 | 0.2385 | 1.421 | 0.5068 | 1.865 | 7.08e-06 | 0.000226 | 3.103 | 1.634 | 6.013 |  |
| Slc17a9 | solute carrier family 17, memb | 0.001435 | 0.02853 | 1.848 | 0.8861 | 3.695 | 0.001634 | 0.04288 | 2.046 | 1.033 | 3.64 | 0.5743 | 0.782 | 0.9012 | -0.15 | -0.5711 | 0.2833 | 0.5233 | 0.8181 | -0.2896 | -1.103 |  |
| Tlr7 | toll-like receptor 7 | 0.001947 | 0.03437 | 2.424 | 1.277 | 3.564 | 0.167 | 0.5254 | 1.524 | 0.6083 | 1.434 | 0.03913 | 0.1515 | 1.824 | 0.8668 | 2.208 | 0.008757 | 0.05091 | 2.205 | 1.141 | 2.905 |  |
| Mettl11b | methyltransferase like 11B | 0.002791 | 0.04202 | 0.3652 | -1.453 | -3.408 | 0.2288 | 0.5898 | 0.6478 | -0.6263 | -1.241 | 0.8174 | 0.9197 | 0.9271 | -0.1093 | -0.234 | 5.773e-05 | 0.001156 | 0.1932 | -2.372 | -5.078 |  |
| Bche | butyrylcholinesterase | 0.5416 | 0.7895 | 0.9063 | -0.142 | -0.6211 | 0.04991 | 0.3094 | 1.479 | 0.5646 | 2.087 | 0.000362 | 0.004666 | 0.4753 | -1.073 | -4.285 | 0.0004642 | 0.005693 | 0.4841 | -1.047 | -4.179 |  |
| Gm9855 | predicted pseudogene 9855 | 0.222 | 0.5218 | 1.179 | 0.2376 | 1.26 | 0.006113 | 0.09799 | 1.606 | 0.6836 | 3.065 | 9.258e-05 | 0.001624 | 0.498 | -1.006 | -4.872 | 0.1234 | 0.3158 | 0.7943 | -0.3322 | -1.609 |  |
| Rps10-ps1 | ribosomal protein S10, pseudog | 0.5375 | 0.7868 | 0.9157 | -0.127 | -0.6274 | 0.008833 | 0.1222 | 0.6176 | -0.6951 | -2.901 | 0.0001049 | 0.001786 | 2.098 | 1.069 | 4.818 | 0.009565 | 0.05429 | 1.554 | 0.6357 | 2.866 |  |
| Ifi30 | interferon gamma inducible pro | 0.00176 | 0.03232 | 2.51 | 1.328 | 3.608 | 0.07899 | 0.3846 | 1.748 | 0.806 | 1.851 | 0.9927 | 0.9977 | 1.003 | 0.003716 | 0.009218 | 0.0009505 | 0.00978 | 2.95 | 1.561 | 3.872 |  |
| March1 | membrane-associated ring finge | 0.001271 | 0.02661 | 2.028 | 1.02 | 3.747 | 0.04569 | 0.2974 | 1.609 | 0.6865 | 2.131 | 0.1908 | 0.4241 | 1.323 | 0.4039 | 1.354 | 0.05899 | 0.1973 | 1.513 | 0.5972 | 2.003 |  |
| Nav2 | neuron navigator 2 | 0.003602 | 0.04893 | 0.6058 | -0.7232 | -3.297 | 0.5146 | 0.795 | 0.8875 | -0.1722 | -0.6634 | 0.8083 | 0.9151 | 1.042 | 0.05907 | 0.2459 | 6.677e-07 | 3.484e-05 | 0.3052 | -1.712 | -7.126 |  |
| Mmd | monocyte to macrophage differe | 0.1275 | 0.3902 | 0.7597 | -0.3966 | -1.59 | 0.2194 | 0.5808 | 1.296 | 0.3741 | 1.268 | 2.307e-05 | 0.0005571 | 0.3542 | -1.497 | -5.481 | 0.007379 | 0.04502 | 0.5686 | -0.8146 | -2.982 |  |
| Gm7984 | predicted gene 7984 | 0.9797 | 0.993 | 1.004 | 0.00515 | 0.02581 | 0.01264 | 0.1497 | 1.566 | 0.6468 | 2.74 | 6.922e-05 | 0.001275 | 0.469 | -1.092 | -4.998 | 0.001102 | 0.01096 | 0.5616 | -0.8324 | -3.809 |  |
| Arhgap20 | Rho GTPase activating protein | 0.04851 | 0.2319 | 0.7617 | -0.3928 | -2.101 | 0.4263 | 0.7444 | 0.8829 | -0.1796 | -0.8121 | 0.01147 | 0.06303 | 1.484 | 0.57 | 2.784 | 8.941e-06 | 0.0002699 | 0.4324 | -1.209 | -5.907 |  |
| Gm12816 | predicted gene 12816 | 0.003184 | 0.04568 | 1.76 | 0.8155 | 3.351 | 0.4551 | 0.7607 | 1.164 | 0.2194 | 0.7618 | 0.01943 | 0.09243 | 1.599 | 0.6776 | 2.542 | 0.000477 | 0.005807 | 2.16 | 1.111 | 4.167 |  |
| Adrb2 | adrenergic receptor, beta 2 | 0.003297 | 0.0467 | 2.254 | 1.172 | 3.336 | 0.7206 | 0.8964 | 1.11 | 0.1508 | 0.3628 | 9.343e-07 | 4.768e-05 | 6.411 | 2.681 | 6.963 | 0.3305 | 0.5693 | 1.305 | 0.384 | 0.9974 |  |
| Sh2d4a | SH2 domain containing 4A | 0.3599 | 0.6558 | 0.8855 | -0.1754 | -0.9371 | 0.007781 | 0.1145 | 0.6351 | -0.655 | -2.958 | 3.53e-05 | 0.000774 | 2.122 | 1.085 | 5.293 | 0.09979 | 0.2774 | 1.278 | 0.3538 | 1.726 |  |
| Lama1 | laminin, alpha 1 | 0.768 | 0.9085 | 0.9608 | -0.05767 | -0.2991 | 0.0107 | 0.136 | 0.6407 | -0.6423 | -2.815 | 1.33e-05 | 0.0003643 | 2.313 | 1.21 | 5.727 | 0.01807 | 0.08596 | 1.458 | 0.5441 | 2.575 |  |
| Acaca | acetyl-Coenzyme A carboxylase | 0.0007663 | 0.01899 | 0.4214 | -1.247 | -3.964 | 0.01456 | 0.1617 | 0.5016 | -0.9953 | -2.675 | 0.03043 | 0.1271 | 0.5734 | -0.8025 | -2.33 | 0.8889 | 0.9504 | 1.034 | 0.04874 | 0.1415 |  |
| Fasn | fatty acid synthase | 0.0002262 | 0.009093 | 0.2268 | -2.141 | -4.487 | 0.001935 | 0.04731 | 0.2477 | -2.013 | -3.567 | 0.6211 | 0.8107 | 0.8337 | -0.2624 | -0.5021 | 0.8216 | 0.917 | 0.9206 | -0.1194 | -0.2285 |  |
| Cox8b | cytochrome c oxidase subunit 8 | 0.2044 | 0.4996 | 0.7793 | -0.3598 | -1.312 | 0.1467 | 0.4964 | 1.404 | 0.4899 | 1.51 | 1.822e-06 | 7.697e-05 | 0.2508 | -1.995 | -6.643 | 0.08026 | 0.2419 | 0.6814 | -0.5535 | -1.843 |  |
| Esm1 | endothelial cell-specific mole | 0.8473 | 0.9425 | 0.97 | -0.04392 | -0.1951 | 0.01884 | 0.1874 | 1.603 | 0.681 | 2.556 | 1.208e-06 | 5.725e-05 | 0.3106 | -1.687 | -6.839 | 0.06199 | 0.2041 | 0.7132 | -0.4877 | -1.977 |  |
| Gm5850 | predicted gene 5850 | 0.003123 | 0.04533 | 1.734 | 0.7943 | 3.36 | 0.3503 | 0.6922 | 1.204 | 0.2675 | 0.9563 | 0.06444 | 0.2116 | 1.421 | 0.5069 | 1.957 | 0.0005029 | 0.006053 | 2.104 | 1.073 | 4.144 |  |
| Gm5851 | predicted gene 5851 | 0.003123 | 0.04533 | 1.734 | 0.7943 | 3.36 | 0.3503 | 0.6922 | 1.204 | 0.2675 | 0.9563 | 0.06444 | 0.2116 | 1.421 | 0.5069 | 1.957 | 0.0005029 | 0.006053 | 2.104 | 1.073 | 4.144 |  |
| Slc9a6 | solute carrier family 9 (sodiu | 0.2692 | 0.5745 | 1.235 | 0.3041 | 1.136 | 0.08035 | 0.3877 | 0.6674 | -0.5833 | -1.842 | 2.02e-08 | 2.621e-06 | 6.155 | 2.622 | 8.943 | 0.8911 | 0.9512 | 1.029 | 0.04065 | 0.1387 |  |
| Atp2b4 | ATPase, Ca++ transporting, pla | 0.007235 | 0.07615 | 0.6884 | -0.5387 | -2.991 | 0.8597 | 0.9534 | 1.027 | 0.03816 | 0.179 | 8.908e-06 | 0.0002683 | 0.4457 | -1.166 | -5.908 | 0.00965 | 0.05465 | 0.6761 | -0.5647 | -2.862 |  |
| Gm5879 | predicted gene 5879 | 0.003391 | 0.0472 | 1.714 | 0.7774 | 3.324 | 0.6715 | 0.8751 | 1.086 | 0.1191 | 0.4304 | 0.00596 | 0.03887 | 1.727 | 0.7883 | 3.076 | 0.0001616 | 0.002523 | 2.276 | 1.187 | 4.631 |  |
| Taf7 | TATA-box binding protein assoc | 0.02285 | 0.1493 | 1.37 | 0.4538 | 2.466 | 0.5598 | 0.8185 | 0.9144 | -0.1291 | -0.593 | 0.07759 | 0.2397 | 1.297 | 0.3751 | 1.861 | 1.274e-06 | 5.968e-05 | 2.591 | 1.374 | 6.814 |  |
| Adamts4 | a disintegrin-like and metallo | 0.004609 | 0.05764 | 1.576 | 0.6558 | 3.19 | 0.7028 | 0.8891 | 1.067 | 0.09417 | 0.3871 | 1.207e-07 | 9.644e-06 | 3.479 | 1.799 | 7.986 | 0.6188 | 0.7973 | 0.9241 | -0.1138 | -0.5055 |  |
| Gm14480 | predicted gene 14480 | 0.003699 | 0.04968 | 0.6548 | -0.6108 | -3.286 | 0.6087 | 0.8448 | 0.9238 | -0.1144 | -0.5201 | 0.0683 | 0.22 | 0.7618 | -0.3924 | -1.927 | 2.859e-05 | 0.0006656 | 0.4676 | -1.097 | -5.386 |  |
| Epha7 | Eph receptor A7 | 0.03135 | 0.1804 | 0.7725 | -0.3724 | -2.315 | 0.4751 | 0.7724 | 1.101 | 0.1385 | 0.7279 | 0.1713 | 0.3973 | 0.8409 | -0.25 | -1.419 | 4.879e-07 | 2.74e-05 | 0.411 | -1.283 | -7.28 |  |
| Bloc1s5 | biogenesis of lysosomal organe | 0.05565 | 0.2499 | 0.7249 | -0.4641 | -2.032 | 0.388 | 0.7177 | 1.18 | 0.2385 | 0.8825 | 0.0001857 | 0.002784 | 0.4526 | -1.144 | -4.571 | 0.0009932 | 0.01011 | 0.5126 | -0.964 | -3.853 |  |
| Pcbd1 | pterin 4 alpha carbinolamine d | 0.02414 | 0.1543 | 0.5896 | -0.7621 | -2.44 | 0.5564 | 0.8173 | 1.166 | 0.2211 | 0.5982 | 4.904e-06 | 0.00017 | 0.2308 | -2.115 | -6.181 | 0.02417 | 0.1062 | 0.5607 | -0.8347 | -2.439 |  |
| Gm5627 | predicted gene 5627 | 0.00321 | 0.04592 | 0.5603 | -0.8356 | -3.348 | 0.6329 | 0.8574 | 0.9055 | -0.1433 | -0.4851 | 7.84e-07 | 4.133e-05 | 0.2629 | -1.927 | -7.048 | 0.59 | 0.7775 | 0.9014 | -0.1497 | -0.5476 |  |
| Col3a1 | collagen, type III, alpha 1 | 0.0003547 | 0.01198 | 0.447 | -1.162 | -4.294 | 0.00409 | 0.07736 | 0.4871 | -1.038 | -3.242 | 0.9919 | 0.9975 | 1.002 | 0.003042 | 0.01026 | 0.2206 | 0.4508 | 0.7712 | -0.3747 | -1.264 |  |
| Lamtor1 | late endosomal/lysosomal adapt | 0.00225 | 0.03717 | 0.5219 | -0.9382 | -3.502 | 0.06349 | 0.3469 | 0.6494 | -0.6229 | -1.965 | 0.0017 | 0.01535 | 0.4786 | -1.063 | -3.623 | 0.6934 | 0.8459 | 1.085 | 0.1174 | 0.4 |  |
| Btg1 | BTG anti-proliferation factor | 0.003606 | 0.04894 | 2.395 | 1.26 | 3.297 | 0.7156 | 0.8942 | 1.123 | 0.1671 | 0.3695 | 0.0007017 | 0.00773 | 3.194 | 1.675 | 4.002 | 0.00105 | 0.01053 | 3.038 | 1.603 | 3.83 |  |
| Uxt | ubiquitously expressed prefold | 0.01989 | 0.1378 | 1.56 | 0.6412 | 2.531 | 0.003136 | 0.06485 | 2.009 | 1.007 | 3.358 | 0.007824 | 0.04744 | 0.5663 | -0.8203 | -2.956 | 0.3324 | 0.5712 | 0.8261 | -0.2757 | -0.9934 |  |
| Fhl3 | four and a half LIM domains 3 | 0.4361 | 0.7168 | 1.144 | 0.1941 | 0.7947 | 0.06745 | 0.357 | 0.6789 | -0.5587 | -1.934 | 0.03784 | 0.1482 | 1.51 | 0.5949 | 2.224 | 4.524e-06 | 0.000157 | 3.168 | 1.663 | 6.218 |  |
| Nnt | nicotinamide nucleotide transh | 0.147 | 0.4215 | 1.277 | 0.3527 | 1.509 | 0.005784 | 0.09507 | 1.808 | 0.8547 | 3.09 | 0.0002864 | 0.00389 | 0.4591 | -1.123 | -4.385 | 0.1506 | 0.3592 | 0.7669 | -0.3828 | -1.495 |  |
| Neu2 | neuraminidase 2 | 0.06701 | 0.2759 | 0.7372 | -0.4399 | -1.937 | 0.3525 | 0.694 | 1.194 | 0.2558 | 0.952 | 0.03668 | 0.1448 | 0.6797 | -0.557 | -2.239 | 5.24e-06 | 0.0001791 | 0.3463 | -1.53 | -6.151 |  |
| Ndrg1 | N-myc downstream regulated gen | 0.512 | 0.7688 | 1.163 | 0.2182 | 0.6676 | 0.05923 | 0.3361 | 0.585 | -0.7736 | -2 | 0.0005116 | 0.006085 | 2.792 | 1.481 | 4.137 | 0.0004699 | 0.005735 | 2.817 | 1.494 | 4.173 |  |
| Cep70 | centrosomal protein 70 | 0.7057 | 0.8795 | 0.9481 | -0.07686 | -0.3831 | 0.0107 | 0.136 | 0.6293 | -0.6682 | -2.815 | 6.779e-07 | 3.688e-05 | 2.958 | 1.564 | 7.119 | 0.3515 | 0.5895 | 1.156 | 0.2096 | 0.954 |  |
| Gm15432 | predicted gene 15432 | 0.05223 | 0.2409 | 0.6381 | -0.6481 | -2.064 | 0.004453 | 0.08159 | 0.4381 | -1.191 | -3.205 | 0.0008145 | 0.008654 | 2.557 | 1.355 | 3.938 | 0.437 | 0.6626 | 1.208 | 0.2728 | 0.7931 |  |
| Ntf3 | neurotrophin 3 | 0.6073 | 0.8293 | 1.084 | 0.1168 | 0.5222 | 0.01117 | 0.1392 | 1.67 | 0.7396 | 2.796 | 0.00228 | 0.01904 | 0.5524 | -0.8561 | -3.496 | 0.0005158 | 0.006171 | 0.4957 | -1.012 | -4.134 |  |
| N4bp2l1 | NEDD4 binding protein 2-like 1 | 0.001871 | 0.03361 | 1.965 | 0.9742 | 3.581 | 0.002254 | 0.05219 | 2.184 | 1.127 | 3.501 | 0.8631 | 0.9415 | 1.037 | 0.05204 | 0.1746 | 0.1026 | 0.2823 | 0.7023 | -0.5099 | -1.711 |  |
| A530099J19Rik | NA | 0.001628 | 0.03075 | 2.357 | 1.237 | 3.641 | 0.03434 | 0.2598 | 1.883 | 0.9129 | 2.271 | 0.7744 | 0.8971 | 0.9278 | -0.1081 | -0.2905 | 0.008824 | 0.05122 | 2.114 | 1.08 | 2.902 |  |
| Erbb2ip | NA | 0.004714 | 0.05835 | 1.38 | 0.4648 | 3.18 | 0.9213 | 0.9742 | 0.9881 | -0.01731 | -0.1001 | 0.2478 | 0.4973 | 1.141 | 0.1906 | 1.191 | 1.598e-07 | 1.185e-05 | 2.388 | 1.256 | 7.842 |  |
| mt-Tg | mitochondrially encoded tRNA g | 0.1498 | 0.4252 | 0.612 | -0.7083 | -1.498 | 0.005733 | 0.09465 | 0.3012 | -1.731 | -3.094 | 0.003398 | 0.02562 | 3.298 | 1.722 | 3.323 | 0.01714 | 0.08264 | 2.544 | 1.347 | 2.6 |  |
| Ankrd2 | ankyrin repeat domain 2 (stret | 0.01102 | 0.09785 | 1.892 | 0.9197 | 2.802 | 0.2377 | 0.5975 | 1.388 | 0.4727 | 1.217 | 0.2592 | 0.5108 | 0.7487 | -0.4176 | -1.161 | 8.88e-05 | 0.001603 | 3.383 | 1.758 | 4.89 |  |
| Gm13237 | predicted gene 13237 | 0.6913 | 0.8735 | 1.069 | 0.09591 | 0.4029 | 0.03426 | 0.2594 | 0.6417 | -0.64 | -2.272 | 0.01398 | 0.07277 | 1.627 | 0.7024 | 2.694 | 1.201e-05 | 0.0003417 | 2.839 | 1.505 | 5.773 |  |
| Icam1 | intercellular adhesion molecul | 0.001901 | 0.03394 | 2.103 | 1.073 | 3.574 | 0.1001 | 0.4215 | 1.529 | 0.6124 | 1.724 | 0.1685 | 0.3933 | 1.385 | 0.4698 | 1.429 | 0.01178 | 0.06363 | 1.881 | 0.9112 | 2.771 |  |
| Ptprf | protein tyrosine phosphatase, | 0.1346 | 0.4028 | 0.7958 | -0.3295 | -1.56 | 0.00869 | 0.1212 | 0.6041 | -0.727 | -2.909 | 0.8856 | 0.9516 | 0.9769 | -0.03373 | -0.1458 | 3.476e-05 | 0.0007794 | 2.34 | 1.226 | 5.3 |  |
| Rassf2 | Ras association (RalGDS/AF-6) | 0.001477 | 0.02898 | 2.86 | 1.516 | 3.683 | 0.04935 | 0.3077 | 2.027 | 1.019 | 2.093 | 0.1634 | 0.3858 | 1.572 | 0.6525 | 1.447 | 0.07798 | 0.2373 | 1.787 | 0.8378 | 1.858 |  |
| Odf3b | outer dense fiber of sperm tai | 0.8115 | 0.9281 | 0.9567 | -0.06388 | -0.2417 | 0.02389 | 0.2123 | 1.699 | 0.7646 | 2.445 | 5.175e-05 | 0.001028 | 0.3575 | -1.484 | -5.125 | 0.002484 | 0.02018 | 0.4995 | -1.001 | -3.459 |  |
| Mafb | v-maf musculoaponeurotic fibro | 0.3923 | 0.6841 | 1.134 | 0.1814 | 0.8745 | 0.01051 | 0.135 | 1.617 | 0.6929 | 2.823 | 0.1817 | 0.4119 | 0.8042 | -0.3144 | -1.384 | 2.968e-05 | 0.0006878 | 0.4293 | -1.22 | -5.37 |  |
| Fnbp1 | formin binding protein 1 | 0.003346 | 0.047 | 1.712 | 0.7759 | 3.33 | 0.5751 | 0.8258 | 1.115 | 0.1571 | 0.5699 | 0.0004264 | 0.005297 | 2.108 | 1.076 | 4.215 | 0.006231 | 0.03969 | 1.717 | 0.7803 | 3.057 |  |
| 4931406P16Rik | RIKEN cDNA 4931406P16 gene | 0.004042 | 0.05265 | 1.461 | 0.5467 | 3.247 | 0.9523 | 0.9855 | 1.008 | 0.01206 | 0.06054 | 8.172e-07 | 4.281e-05 | 2.456 | 1.296 | 7.028 | 0.1108 | 0.2964 | 1.238 | 0.3077 | 1.669 |  |
| Shq1 | SHQ1 homolog (S. cerevisiae) | 3e-04 | 0.01072 | 2.092 | 1.065 | 4.366 | 0.002458 | 0.055 | 2 | 0.9997 | 3.463 | 0.5004 | 0.7298 | 1.136 | 0.1834 | 0.6863 | 0.963 | 0.9832 | 1.009 | 0.01256 | 0.04701 |  |
| Popdc2 | popeye domain containing 2 | 0.003772 | 0.05031 | 0.5066 | -0.9811 | -3.277 | 0.7206 | 0.8964 | 0.9148 | -0.1285 | -0.3628 | 0.000163 | 0.002522 | 0.3493 | -1.518 | -4.628 | 0.004799 | 0.03283 | 0.4862 | -1.04 | -3.172 |  |
| Atp6v0a2 | ATPase, H+ transporting, lysos | 0.8254 | 0.934 | 1.027 | 0.0379 | 0.2235 | 0.01244 | 0.1482 | 1.465 | 0.5511 | 2.747 | 2.565e-05 | 0.0006052 | 0.4968 | -1.009 | -5.434 | 0.009811 | 0.0553 | 0.6925 | -0.5302 | -2.854 |  |
| Cyp4a10 | cytochrome P450, family 4, sub | 0.2736 | 0.5794 | 1.333 | 0.4148 | 1.126 | 0.007593 | 0.1128 | 2.452 | 1.294 | 2.969 | 0.002256 | 0.01889 | 0.3756 | -1.413 | -3.5 | 0.006514 | 0.04105 | 0.4276 | -1.226 | -3.037 |  |
| Gm25357 | predicted gene, 25357 | 0.001437 | 0.02853 | 0.4979 | -1.006 | -3.695 | 0.01986 | 0.1928 | 0.5681 | -0.8158 | -2.532 | 0.7243 | 0.8718 | 1.077 | 0.1067 | 0.3577 | 0.03424 | 0.1356 | 0.625 | -0.678 | -2.273 |  |
| Igsf6 | immunoglobulin superfamily, me | 0.002165 | 0.03647 | 2.119 | 1.084 | 3.518 | 0.153 | 0.5063 | 1.455 | 0.5414 | 1.486 | 0.009653 | 0.05529 | 1.953 | 0.9655 | 2.862 | 0.06419 | 0.2088 | 1.581 | 0.6611 | 1.959 |  |
| Baz1a | bromodomain adjacent to zinc f | 0.001601 | 0.03042 | 2.27 | 1.183 | 3.648 | 0.05466 | 0.3231 | 1.721 | 0.783 | 2.041 | 0.2884 | 0.5434 | 1.308 | 0.3873 | 1.091 | 0.03332 | 0.133 | 1.755 | 0.8118 | 2.286 |  |
| Smagp | small cell adhesion glycoprote | 0.3187 | 0.6197 | 0.8195 | -0.2872 | -1.023 | 0.008217 | 0.1181 | 0.5088 | -0.975 | -2.934 | 0.001386 | 0.01316 | 2.206 | 1.142 | 3.71 | 0.007155 | 0.04411 | 1.894 | 0.9216 | 2.995 |  |
| Mxra8 | matrix-remodelling associated | 0.1333 | 0.4006 | 0.7821 | -0.3545 | -1.565 | 0.2274 | 0.5885 | 1.26 | 0.3338 | 1.245 | 1.653e-06 | 7.146e-05 | 0.3164 | -1.66 | -6.69 | 0.1185 | 0.3085 | 0.7553 | -0.4048 | -1.631 |  |
| Phospho1 | phosphatase, orphan 1 | 0.3467 | 0.6445 | 1.159 | 0.2124 | 0.9637 | 0.07912 | 0.3847 | 0.7157 | -0.4826 | -1.85 | 1.01e-07 | 8.577e-06 | 3.867 | 1.951 | 8.079 | 0.5844 | 0.774 | 1.098 | 0.1343 | 0.5559 |  |
| Acvr2a | activin receptor IIA | 0.8744 | 0.953 | 0.988 | -0.01741 | -0.1601 | 0.06753 | 0.3571 | 1.188 | 0.2487 | 1.933 | 0.1052 | 0.2929 | 1.15 | 0.2022 | 1.697 | 5.494e-08 | 5.457e-06 | 0.4998 | -1.001 | -8.4 |  |
| Ninl | ninein-like | 0.03624 | 0.1961 | 1.254 | 0.3261 | 2.245 | 0.4702 | 0.7696 | 0.916 | -0.1265 | -0.7361 | 5.018e-07 | 2.934e-05 | 2.229 | 1.156 | 7.267 | 0.2196 | 0.4497 | 1.15 | 0.2016 | 1.267 |  |
| Cyp20a1 | cytochrome P450, family 20, su | 0.3512 | 0.6482 | 1.107 | 0.1468 | 0.9546 | 0.09672 | 0.416 | 0.8027 | -0.3172 | -1.743 | 0.03476 | 0.1394 | 1.303 | 0.3816 | 2.265 | 7.331e-06 | 0.0002322 | 2.014 | 1.01 | 5.997 |  |
| Tbc1d1 | TBC1 domain family, member 1 | 0.004019 | 0.05248 | 1.738 | 0.7976 | 3.25 | 0.2928 | 0.6486 | 1.243 | 0.3138 | 1.08 | 0.9133 | 0.9646 | 0.9797 | -0.02965 | -0.1103 | 2.169e-05 | 0.0005356 | 2.792 | 1.481 | 5.509 |  |
| Tmem97 | transmembrane protein 97 | 0.2624 | 0.5678 | 0.8041 | -0.3145 | -1.153 | 0.008523 | 0.1201 | 0.5207 | -0.9415 | -2.917 | 0.01334 | 0.07019 | 1.755 | 0.8112 | 2.715 | 0.001875 | 0.01631 | 2.099 | 1.07 | 3.58 |  |
| Ibtk | inhibitor of Bruton agammaglob | 0.3601 | 0.6559 | 1.109 | 0.1491 | 0.9367 | 0.3244 | 0.6723 | 0.8764 | -0.1903 | -1.01 | 9.085e-08 | 7.976e-06 | 2.674 | 1.419 | 8.134 | 0.03269 | 0.1314 | 0.7577 | -0.4003 | -2.295 |  |
| Lilr4b | leukocyte immunoglobulin-like | 0.009754 | 0.09113 | 1.555 | 0.6372 | 2.857 | 0.8501 | 0.9501 | 0.9656 | -0.05054 | -0.1915 | 0.0002898 | 0.003924 | 2.1 | 1.07 | 4.38 | 0.000607 | 0.006992 | 1.99 | 0.993 | 4.064 |  |
| Srsf3 | serine/arginine-rich splicing | 0.7493 | 0.9004 | 1.055 | 0.0772 | 0.324 | 0.0123 | 0.1473 | 1.712 | 0.776 | 2.752 | 3.955e-05 | 0.0008387 | 0.3872 | -1.369 | -5.243 | 0.01137 | 0.06198 | 0.6039 | -0.7277 | -2.788 |  |
| mt-Tf | mitochondrially encoded tRNA p | 0.0009099 | 0.0213 | 0.4453 | -1.167 | -3.891 | 0.01795 | 0.1824 | 0.5302 | -0.9153 | -2.579 | 0.08386 | 0.2526 | 0.6607 | -0.598 | -1.819 | 0.6362 | 0.8082 | 0.8964 | -0.1579 | -0.4803 |  |
| Spock2 | sparc/osteonectin, cwcv and ka | 0.1699 | 0.4537 | 1.313 | 0.3924 | 1.424 | 0.2021 | 0.5644 | 0.7422 | -0.4301 | -1.319 | 0.0003689 | 0.004738 | 2.447 | 1.291 | 4.277 | 0.0008968 | 0.009374 | 2.26 | 1.177 | 3.897 |  |
| Gm11451 | predicted gene 11451 | 0.2956 | 0.5996 | 0.8274 | -0.2734 | -1.074 | 0.1211 | 0.4577 | 1.402 | 0.4876 | 1.619 | 0.05379 | 0.187 | 0.673 | -0.5714 | -2.049 | 5.373e-06 | 0.0001831 | 0.3053 | -1.712 | -6.139 |  |
| Gmip | Gem-interacting protein | 0.003136 | 0.04534 | 1.842 | 0.8816 | 3.358 | 0.2436 | 0.6033 | 1.295 | 0.3733 | 1.202 | 0.1 | 0.2839 | 1.41 | 0.496 | 1.725 | 0.001886 | 0.0164 | 2.041 | 1.029 | 3.578 |  |
| 2610020H08Rik | NA | 0.002319 | 0.03777 | 1.748 | 0.806 | 3.489 | 0.1423 | 0.4893 | 1.336 | 0.4176 | 1.528 | 0.000626 | 0.007113 | 2.035 | 1.025 | 4.051 | 0.5863 | 0.7755 | 1.102 | 0.14 | 0.5531 |  |
| Gvin1 | GTPase, very large interferon | 0.05682 | 0.2524 | 0.6394 | -0.6451 | -2.022 | 0.005244 | 0.08946 | 0.4405 | -1.183 | -3.133 | 0.001691 | 0.0153 | 2.407 | 1.267 | 3.625 | 0.3337 | 0.5722 | 1.271 | 0.3463 | 0.9906 |  |
| B3glct | beta-3-glucosyltransferase | 0.004729 | 0.05845 | 0.7275 | -0.4589 | -3.178 | 0.9998 | 0.9998 | 1 | 4.533e-05 | 0.0002653 | 3.46e-06 | 0.0001277 | 0.4989 | -1.003 | -6.343 | 0.0284 | 0.119 | 0.7718 | -0.3737 | -2.363 |  |
| Rpl38 | ribosomal protein L38 | 0.7975 | 0.9209 | 0.9655 | -0.0506 | -0.2601 | 0.01319 | 0.1525 | 0.6479 | -0.6262 | -2.72 | 7.393e-05 | 0.001345 | 2.084 | 1.059 | 4.97 | 0.00525 | 0.03501 | 1.588 | 0.6676 | 3.132 |  |
| Hmgb2 | high mobility group box 2 | 0.4664 | 0.7387 | 1.119 | 0.1617 | 0.7425 | 0.06482 | 0.3495 | 0.7054 | -0.5035 | -1.954 | 0.2387 | 0.4867 | 1.222 | 0.2897 | 1.214 | 6.343e-07 | 3.348e-05 | 3.262 | 1.706 | 7.151 |  |
| Chchd10 | coiled-coil-helix-coiled-coil- | 0.05884 | 0.2574 | 1.409 | 0.4951 | 2.004 | 0.1832 | 0.5439 | 1.322 | 0.4031 | 1.379 | 0.000593 | 0.006819 | 2.147 | 1.103 | 4.074 | 0.006269 | 0.03985 | 0.5639 | -0.8266 | -3.054 |  |
| Cript | cysteine-rich PDZ-binding prot | 0.9673 | 0.9888 | 0.9941 | -0.008467 | -0.04151 | 0.0169 | 0.1759 | 1.547 | 0.6291 | 2.607 | 2.626e-05 | 0.0006146 | 0.4317 | -1.212 | -5.424 | 0.005207 | 0.03482 | 0.6153 | -0.7007 | -3.136 |  |
| Prkce | protein kinase C, epsilon | 0.06206 | 0.2647 | 1.178 | 0.2367 | 1.977 | 0.328 | 0.6756 | 0.9062 | -0.1421 | -1.003 | 5.074e-08 | 5e-06 | 2.155 | 1.108 | 8.442 | 0.8284 | 0.9205 | 1.02 | 0.02881 | 0.2196 |  |
| Tenm4 | teneurin transmembrane protein | 0.03506 | 0.1924 | 0.5388 | -0.8921 | -2.261 | 0.5141 | 0.795 | 1.24 | 0.31 | 0.6642 | 6.599e-05 | 0.001234 | 0.2224 | -2.169 | -5.019 | 0.003374 | 0.0254 | 0.3693 | -1.437 | -3.326 |  |
| Dgka | diacylglycerol kinase, alpha | 0.0004772 | 0.01427 | 2.55 | 1.35 | 4.167 | 0.001451 | 0.03946 | 2.667 | 1.415 | 3.691 | 0.7243 | 0.8718 | 0.9157 | -0.127 | -0.3577 | 0.8516 | 0.9321 | 0.9544 | -0.06729 | -0.1896 |  |
| Stap1 | signal transducing adaptor fam | 0.003452 | 0.04776 | 4.149 | 2.053 | 3.316 | 0.00243 | 0.05468 | 5.817 | 2.54 | 3.468 | 0.07945 | 0.2437 | 0.4195 | -1.253 | -1.848 | 0.7602 | 0.8849 | 0.8646 | -0.2098 | -0.3095 |  |
| Ppp1r10 | protein phosphatase 1, regulat | 0.5229 | 0.777 | 1.133 | 0.1803 | 0.6504 | 0.05957 | 0.3375 | 0.6349 | -0.6554 | -1.998 | 2.656e-06 | 0.0001042 | 3.902 | 1.964 | 6.466 | 0.08897 | 0.258 | 1.457 | 0.5431 | 1.788 |  |
| Crot | carnitine O-octanoyltransferas | 0.005773 | 0.06723 | 0.6647 | -0.5892 | -3.091 | 0.9864 | 0.996 | 1.003 | 0.003883 | 0.01722 | 8.396e-05 | 0.001496 | 0.491 | -1.026 | -4.914 | 0.00177 | 0.01568 | 0.5934 | -0.7529 | -3.605 |  |
| Itlnb | NA | 0.414 | 0.7009 | 0.8582 | -0.2207 | -0.8342 | 0.08155 | 0.3899 | 1.489 | 0.5741 | 1.834 | 2.406e-05 | 0.0005756 | 0.3338 | -1.583 | -5.463 | 0.01194 | 0.06405 | 0.5738 | -0.8014 | -2.766 |  |
| Fabp2 | fatty acid binding protein 2, | 0.7104 | 0.8815 | 1.08 | 0.1106 | 0.3767 | 0.01367 | 0.1552 | 1.918 | 0.9398 | 2.704 | 0.00501 | 0.03409 | 0.495 | -1.015 | -3.153 | 0.0001834 | 0.002795 | 0.3603 | -1.473 | -4.577 |  |
| Midn | midnolin | 0.5932 | 0.8207 | 1.079 | 0.1095 | 0.5429 | 0.04612 | 0.299 | 0.7035 | -0.5074 | -2.126 | 0.2592 | 0.5108 | 1.195 | 0.2565 | 1.161 | 5.561e-07 | 3.024e-05 | 3.019 | 1.594 | 7.216 |  |
| Il22ra2 | interleukin 22 receptor, alpha | 0.00212 | 0.03621 | 2.83 | 1.501 | 3.527 | 0.125 | 0.4644 | 1.749 | 0.8062 | 1.601 | 0.007557 | 0.04616 | 2.612 | 1.385 | 2.971 | 0.1492 | 0.3572 | 1.624 | 0.6994 | 1.5 |  |
| Tspan9 | tetraspanin 9 | 0.003725 | 0.04984 | 0.576 | -0.7958 | -3.283 | 0.2522 | 0.6119 | 0.791 | -0.3382 | -1.179 | 0.7039 | 0.8599 | 0.9315 | -0.1024 | -0.3855 | 0.0001136 | 0.001933 | 0.4146 | -1.27 | -4.783 |  |
| Rit1 | Ras-like without CAAX 1 | 0.3202 | 0.6211 | 1.137 | 0.1849 | 1.019 | 0.1132 | 0.4454 | 0.7815 | -0.3556 | -1.657 | 0.1389 | 0.3494 | 1.237 | 0.3064 | 1.542 | 1.919e-06 | 8.226e-05 | 2.489 | 1.315 | 6.619 |  |
| Exph5 | exophilin 5 | 0.727 | 0.8897 | 0.946 | -0.08005 | -0.3541 | 0.01477 | 0.1633 | 0.6097 | -0.7139 | -2.668 | 0.002575 | 0.02094 | 1.806 | 0.8528 | 3.443 | 0.0004079 | 0.005177 | 2.069 | 1.049 | 4.234 |  |
| Aldh1a7 | aldehyde dehydrogenase family | 0.002716 | 0.0413 | 0.5043 | -0.9877 | -3.42 | 0.1686 | 0.5273 | 0.713 | -0.4881 | -1.428 | 0.001096 | 0.01094 | 0.4336 | -1.206 | -3.811 | 0.3652 | 0.601 | 0.8161 | -0.2932 | -0.9267 |  |
| Gja1 | gap junction protein, alpha 1 | 0.6617 | 0.8576 | 0.9176 | -0.1241 | -0.4441 | 0.04156 | 0.2853 | 1.647 | 0.7201 | 2.178 | 6.273e-06 | 0.0002069 | 0.276 | -1.857 | -6.068 | 0.03928 | 0.1491 | 0.6262 | -0.6752 | -2.206 |  |
| Atp10a | ATPase, class V, type 10A | 0.145 | 0.4184 | 0.8383 | -0.2545 | -1.517 | 0.2315 | 0.5929 | 1.185 | 0.245 | 1.234 | 1.471e-05 | 0.0003946 | 0.4848 | -1.044 | -5.682 | 0.02263 | 0.1011 | 0.73 | -0.4541 | -2.47 |  |
| Haao | 3-hydroxyanthranilate 3,4-diox | 0.003505 | 0.04813 | 2.261 | 1.177 | 3.309 | 0.2418 | 0.6015 | 1.422 | 0.5075 | 1.206 | 0.2085 | 0.448 | 1.42 | 0.5062 | 1.3 | 0.0009898 | 0.0101 | 2.831 | 1.501 | 3.855 |  |
| Scand1 | SCAN domain-containing 1 | 0.08056 | 0.303 | 0.8093 | -0.3053 | -1.841 | 0.3387 | 0.6836 | 1.143 | 0.1924 | 0.9801 | 2.702e-06 | 0.0001056 | 0.4434 | -1.173 | -6.458 | 0.09385 | 0.2665 | 0.8013 | -0.3197 | -1.759 |  |
| Plcg2 | phospholipase C, gamma 2 | 0.002426 | 0.03874 | 2.611 | 1.385 | 3.469 | 0.05085 | 0.3118 | 1.974 | 0.9814 | 2.078 | 0.776 | 0.8979 | 0.9163 | -0.1261 | -0.2884 | 0.006237 | 0.03972 | 2.525 | 1.337 | 3.056 |  |
| Gm8074 | predicted gene 8074 | 0.9023 | 0.9636 | 1.016 | 0.02335 | 0.1244 | 0.02179 | 0.203 | 0.6817 | -0.5528 | -2.488 | 7.869e-05 | 0.00142 | 2.023 | 1.017 | 4.943 | 0.002473 | 0.02011 | 1.638 | 0.7118 | 3.461 |  |
| LOC100862446 | NA | 0.1433 | 0.4154 | 1.2 | 0.2634 | 1.523 | 0.2206 | 0.582 | 0.8359 | -0.2587 | -1.265 | 0.5507 | 0.7675 | 1.083 | 0.115 | 0.6071 | 2.27e-07 | 1.545e-05 | 2.734 | 1.451 | 7.663 |  |
| Cd300a | CD300A molecule | 0.3725 | 0.6671 | 1.084 | 0.1167 | 0.9123 | 0.4043 | 0.7299 | 0.9145 | -0.129 | -0.852 | 1.382e-07 | 1.08e-05 | 2.158 | 1.11 | 7.917 | 0.01514 | 0.07586 | 0.7725 | -0.3724 | -2.657 |  |
| Rgs7bp | regulator of G-protein signall | 0.0636 | 0.2681 | 0.6641 | -0.5906 | -1.964 | 0.3925 | 0.7216 | 1.241 | 0.311 | 0.874 | 1.072e-05 | 0.0003097 | 0.2645 | -1.919 | -5.825 | 0.02704 | 0.1151 | 0.5799 | -0.7861 | -2.386 |  |
| Gm12538 | predicted gene 12538 | 0.9091 | 0.967 | 1.023 | 0.03237 | 0.1157 | 0.02063 | 0.197 | 0.5617 | -0.8321 | -2.514 | 1.335e-05 | 0.0003651 | 3.375 | 1.755 | 5.726 | 0.01273 | 0.06707 | 1.788 | 0.8387 | 2.736 |  |
| Paox | polyamine oxidase (exo-N4-amin | 0.1778 | 0.465 | 1.421 | 0.5065 | 1.397 | 0.2068 | 0.5691 | 0.6784 | -0.5598 | -1.305 | 0.0001447 | 0.002293 | 3.626 | 1.859 | 4.679 | 0.003016 | 0.02325 | 2.532 | 1.34 | 3.375 |  |
| 0610009B22Rik | RIKEN cDNA 0610009B22 gene | 0.1894 | 0.481 | 0.7982 | -0.3252 | -1.359 | 0.198 | 0.5611 | 1.299 | 0.3771 | 1.332 | 0.0001108 | 0.001868 | 0.4184 | -1.257 | -4.794 | 0.004096 | 0.02933 | 0.5548 | -0.8499 | -3.241 |  |
| Apobec1 | apolipoprotein B mRNA editing | 0.08964 | 0.3225 | 1.422 | 0.5076 | 1.784 | 0.329 | 0.6761 | 0.7917 | -0.3369 | -1.001 | 0.0003121 | 0.004156 | 2.559 | 1.356 | 4.349 | 0.001181 | 0.01154 | 2.263 | 1.178 | 3.779 |  |
| St6galnac5 | ST6 (alpha-N-acetyl-neuraminyl | 0.01372 | 0.111 | 1.932 | 0.9501 | 2.702 | 0.7535 | 0.9095 | 0.9123 | -0.1324 | -0.3184 | 1.368e-06 | 6.199e-05 | 6.11 | 2.611 | 6.78 | 0.1141 | 0.3017 | 1.554 | 0.6363 | 1.652 |  |
| Adcyap1r1 | adenylate cyclase activating p | 0.03098 | 0.1791 | 1.264 | 0.3377 | 2.321 | 0.5408 | 0.8082 | 0.9284 | -0.1071 | -0.6223 | 6.582e-07 | 3.626e-05 | 2.199 | 1.137 | 7.133 | 0.2295 | 0.4612 | 1.147 | 0.1976 | 1.24 |  |
| Trbv20 | T cell receptor beta, variable | 0.001433 | 0.0285 | 8.066 | 3.012 | 3.696 | 0.01537 | 0.1665 | 5.878 | 2.555 | 2.65 | 0.7658 | 0.8931 | 0.8295 | -0.2696 | -0.302 | 0.0812 | 0.2438 | 3.116 | 1.64 | 1.837 |  |
| Hip1r | huntingtin interacting protein | 0.3289 | 0.6287 | 1.153 | 0.2052 | 1.001 | 0.1161 | 0.4504 | 0.7587 | -0.3984 | -1.643 | 0.003104 | 0.02397 | 1.688 | 0.755 | 3.362 | 0.0001374 | 0.002241 | 2.079 | 1.056 | 4.701 |  |
| Syp | synaptophysin | 0.004663 | 0.05812 | 0.6292 | -0.6684 | -3.185 | 0.7143 | 0.8938 | 0.9381 | -0.09222 | -0.3713 | 6.011e-06 | 0.0001998 | 0.379 | -1.4 | -6.088 | 0.1681 | 0.384 | 0.7962 | -0.3289 | -1.43 |  |
| Cd300lg | CD300 molecule like family mem | 0.02296 | 0.1498 | 1.301 | 0.3794 | 2.463 | 0.6197 | 0.8507 | 0.9383 | -0.09187 | -0.5041 | 7.496e-07 | 3.967e-05 | 2.286 | 1.193 | 7.07 | 0.205 | 0.4318 | 1.166 | 0.221 | 1.31 |  |
| Paqr4 | progestin and adipoQ receptor | 0.03574 | 0.1946 | 0.7193 | -0.4754 | -2.252 | 0.5243 | 0.8007 | 1.119 | 0.1619 | 0.648 | 6.624e-06 | 0.000216 | 0.3796 | -1.397 | -6.043 | 0.03787 | 0.1458 | 0.7002 | -0.5142 | -2.224 |  |
| Mest | mesoderm specific transcript | 0.00214 | 0.03634 | 0.4015 | -1.317 | -3.523 | 0.08225 | 0.3915 | 0.5708 | -0.809 | -1.83 | 0.08398 | 0.2529 | 0.5969 | -0.7445 | -1.819 | 0.07185 | 0.2254 | 0.5831 | -0.7781 | -1.901 |  |
| Tmem126a | transmembrane protein 126A | 0.005188 | 0.06228 | 0.6754 | -0.5661 | -3.138 | 0.7763 | 0.9194 | 0.9583 | -0.06148 | -0.288 | 1.195e-05 | 0.000337 | 0.4533 | -1.142 | -5.776 | 0.07421 | 0.2303 | 0.7725 | -0.3724 | -1.884 |  |
| Ccl7 | chemokine (C-C motif) ligand 7 | 0.003255 | 0.04626 | 0.5397 | -0.8898 | -3.342 | 0.2326 | 0.5939 | 0.7643 | -0.3878 | -1.231 | 0.000571 | 0.006625 | 0.4374 | -1.193 | -4.09 | 0.2964 | 0.5373 | 0.8051 | -0.3128 | -1.072 |  |
| Ccdc146 | coiled-coil domain containing | 0.004917 | 0.06007 | 0.6791 | -0.5582 | -3.161 | 0.4365 | 0.7507 | 0.8914 | -0.1659 | -0.7941 | 0.3873 | 0.6381 | 0.8883 | -0.171 | -0.8837 | 4.359e-05 | 0.0009328 | 0.4979 | -1.006 | -5.2 |  |
| Wfdc1 | WAP four-disulfide core domain | 0.1251 | 0.3863 | 0.7283 | -0.4573 | -1.601 | 0.007365 | 0.1109 | 0.4972 | -1.008 | -2.983 | 0.001049 | 0.01056 | 2.295 | 1.198 | 3.83 | 0.1623 | 0.3756 | 1.37 | 0.4541 | 1.451 |  |
| Ccdc28a | coiled-coil domain containing | 0.977 | 0.9918 | 1.003 | 0.004593 | 0.02919 | 0.01765 | 0.1805 | 0.7162 | -0.4815 | -2.586 | 3.661e-06 | 0.0001328 | 2.127 | 1.089 | 6.316 | 0.04445 | 0.1624 | 1.292 | 0.3697 | 2.145 |  |
| Mettl7b | methyltransferase like 7B | 0.01597 | 0.1213 | 0.7008 | -0.5129 | -2.633 | 0.3071 | 0.66 | 0.8458 | -0.2416 | -1.048 | 4.106e-05 | 0.00086 | 0.4615 | -1.115 | -5.227 | 0.1729 | 0.3906 | 1.233 | 0.3017 | 1.414 |  |
| Gm12407 | predicted gene 12407 | 0.001952 | 0.03439 | 5.488 | 2.456 | 3.563 | 0.01503 | 0.1646 | 4.501 | 2.17 | 2.661 | 0.559 | 0.7726 | 0.7326 | -0.4488 | -0.5943 | 0.09896 | 0.276 | 2.474 | 1.307 | 1.73 |  |
| Gm2457 | NA | 0.001952 | 0.03439 | 5.488 | 2.456 | 3.563 | 0.01503 | 0.1646 | 4.501 | 2.17 | 2.661 | 0.559 | 0.7726 | 0.7326 | -0.4488 | -0.5943 | 0.09896 | 0.276 | 2.474 | 1.307 | 1.73 |  |
| Zfp422-rs1 | NA | 0.005266 | 0.063 | 1.437 | 0.5235 | 3.131 | 0.5543 | 0.8167 | 1.086 | 0.119 | 0.6015 | 0.5051 | 0.7336 | 1.09 | 0.1243 | 0.6787 | 8.178e-06 | 0.0002516 | 2.128 | 1.089 | 5.947 |  |
| Deb1 | NA | 0.009125 | 0.08754 | 0.7127 | -0.4886 | -2.887 | 0.8853 | 0.9611 | 1.02 | 0.02927 | 0.1462 | 0.00899 | 0.05245 | 0.6895 | -0.5365 | -2.894 | 2.282e-05 | 0.0005574 | 0.4941 | -1.017 | -5.486 |  |
| Tmem2 | NA | 0.5222 | 0.7767 | 1.116 | 0.1579 | 0.6514 | 0.01451 | 0.1614 | 1.703 | 0.7679 | 2.676 | 0.1361 | 0.3449 | 0.7513 | -0.4125 | -1.553 | 3.203e-05 | 0.0007298 | 0.3744 | -1.417 | -5.336 |  |
| Cxcl9 | chemokine (C-X-C motif) ligand | 0.5996 | 0.8247 | 1.116 | 0.1583 | 0.5335 | 0.05284 | 0.3178 | 0.606 | -0.7226 | -2.058 | 2.562e-05 | 0.0006052 | 3.402 | 1.766 | 5.435 | 0.0139 | 0.07128 | 1.836 | 0.8763 | 2.696 |  |
| Csf1r | colony stimulating factor 1 re | 0.04557 | 0.2233 | 1.531 | 0.6148 | 2.132 | 0.4906 | 0.7812 | 0.847 | -0.2396 | -0.7023 | 0.002607 | 0.02116 | 2.122 | 1.086 | 3.438 | 0.0001452 | 0.002337 | 2.784 | 1.477 | 4.678 |  |
| Cldn22 | claudin 22 | 0.3154 | 0.6168 | 0.8388 | -0.2536 | -1.03 | 0.111 | 0.4428 | 1.4 | 0.4859 | 1.668 | 2.911e-07 | 1.94e-05 | 0.2443 | -2.033 | -7.537 | 0.5003 | 0.7125 | 0.8795 | -0.1852 | -0.6864 |  |
| Lsm10 | U7 snRNP-specific Sm-like prot | 0.2107 | 0.5084 | 0.8082 | -0.3073 | -1.293 | 0.009966 | 0.1308 | 0.5741 | -0.8005 | -2.847 | 0.08867 | 0.2623 | 1.381 | 0.4659 | 1.79 | 0.0009008 | 0.009407 | 2.019 | 1.014 | 3.895 |  |
| Napb | N-ethylmaleimide sensitive fus | 0.007985 | 0.08077 | 1.521 | 0.6048 | 2.947 | 0.8352 | 0.9447 | 0.9651 | -0.05121 | -0.2108 | 0.7823 | 0.9011 | 1.045 | 0.06298 | 0.2801 | 4.793e-08 | 4.912e-06 | 3.745 | 1.905 | 8.473 |  |
| Cebpb | CCAAT/enhancer binding protein | 0.01291 | 0.1072 | 1.734 | 0.7945 | 2.73 | 0.2231 | 0.5841 | 1.35 | 0.433 | 1.258 | 0.3307 | 0.5857 | 0.8023 | -0.3179 | -0.997 | 0.0002781 | 0.003879 | 2.643 | 1.402 | 4.398 |  |
| Gm13283 | predicted gene 13283 | 0.125 | 0.386 | 1.179 | 0.2373 | 1.601 | 0.2524 | 0.6122 | 0.8665 | -0.2066 | -1.179 | 2.757e-07 | 1.861e-05 | 2.343 | 1.228 | 7.565 | 0.5297 | 0.7354 | 1.075 | 0.1038 | 0.6396 |  |
| Ric8b | RIC8 guanine nucleotide exchan | 0.02347 | 0.1518 | 0.6948 | -0.5254 | -2.453 | 0.6419 | 0.8608 | 1.086 | 0.1197 | 0.4722 | 0.008041 | 0.04836 | 0.6196 | -0.6906 | -2.943 | 3.435e-05 | 0.000773 | 0.422 | -1.245 | -5.305 |  |
| Hspb3 | heat shock protein 3 | 0.005422 | 0.06419 | 0.6281 | -0.6709 | -3.118 | 0.5185 | 0.7968 | 0.8905 | -0.1673 | -0.6572 | 0.4946 | 0.7252 | 0.8926 | -0.164 | -0.6958 | 1.37e-05 | 0.000378 | 0.3932 | -1.347 | -5.714 |  |
| Gm29718 | predicted gene, 29718 | 0.004596 | 0.0575 | 0.6089 | -0.7156 | -3.191 | 0.6245 | 0.8526 | 0.9126 | -0.1319 | -0.4971 | 6.601e-07 | 3.628e-05 | 0.2968 | -1.752 | -7.132 | 0.9965 | 0.9982 | 1.001 | 0.00108 | 0.004396 |  |
| Plin3 | perilipin 3 | 0.7749 | 0.9109 | 0.9639 | -0.05302 | -0.2899 | 0.06217 | 0.3444 | 0.7435 | -0.4275 | -1.976 | 0.1206 | 0.319 | 0.7984 | -0.3248 | -1.621 | 5.412e-07 | 2.956e-05 | 2.729 | 1.448 | 7.229 |  |
| Grb10 | growth factor receptor bound p | 0.4316 | 0.7132 | 1.115 | 0.1571 | 0.8027 | 0.1306 | 0.4718 | 0.7764 | -0.3651 | -1.576 | 8.881e-08 | 7.877e-06 | 3.356 | 1.747 | 8.146 | 0.4111 | 0.6403 | 0.8827 | -0.18 | -0.8395 |  |
| Ccl19 | predicted 2023 | 0.002125 | 0.03624 | 5.315 | 2.41 | 3.526 | 0.01562 | 0.1678 | 4.399 | 2.137 | 2.643 | 0.5484 | 0.7658 | 0.7285 | -0.4571 | -0.6105 | 0.104 | 0.285 | 2.42 | 1.275 | 1.703 |  |
| Grb14 | growth factor receptor bound p | 0.0199 | 0.1378 | 0.4754 | -1.073 | -2.531 | 0.6816 | 0.8803 | 1.156 | 0.2088 | 0.4164 | 2.809e-05 | 0.0006467 | 0.1762 | -2.505 | -5.394 | 0.00915 | 0.05267 | 0.3951 | -1.34 | -2.886 |  |
| Prob1 | proline rich basic protein 1 | 0.1264 | 0.3883 | 1.292 | 0.3696 | 1.595 | 0.02805 | 0.2323 | 1.568 | 0.6493 | 2.369 | 0.0001773 | 0.002694 | 0.4459 | -1.165 | -4.591 | 0.2136 | 0.4425 | 1.254 | 0.3261 | 1.285 |  |
| Ccdc69 | coiled-coil domain containing | 0.6464 | 0.8492 | 1.053 | 0.0744 | 0.4657 | 0.06214 | 0.3443 | 0.7719 | -0.3735 | -1.976 | 7.06e-08 | 6.604e-06 | 2.726 | 1.447 | 8.267 | 0.5628 | 0.7584 | 0.9311 | -0.103 | -0.5885 |  |
| Igsf11 | immunoglobulin superfamily, me | 0.2556 | 0.5598 | 1.255 | 0.328 | 1.171 | 0.01006 | 0.1315 | 1.922 | 0.9424 | 2.843 | 0.0005547 | 0.006462 | 0.4178 | -1.259 | -4.103 | 0.07144 | 0.2247 | 0.667 | -0.5843 | -1.904 |  |
| Atp10d | ATPase, class V, type 10D | 0.002535 | 0.03963 | 2.483 | 1.312 | 3.45 | 0.01115 | 0.1392 | 2.393 | 1.259 | 2.797 | 0.202 | 0.439 | 1.464 | 0.5497 | 1.319 | 0.3617 | 0.5981 | 0.7637 | -0.3889 | -0.9335 |  |
| Gm22362 | predicted gene, 22362 | 0.5692 | 0.8064 | 1.112 | 0.1531 | 0.5788 | 0.06208 | 0.3442 | 0.6514 | -0.6184 | -1.976 | 0.006715 | 0.04243 | 1.835 | 0.8759 | 3.024 | 7.461e-05 | 0.001415 | 2.71 | 1.438 | 4.966 |  |
| Tmcc3 | transmembrane and coiled coil | 0.03651 | 0.1968 | 1.266 | 0.3407 | 2.241 | 0.4853 | 0.7781 | 0.9152 | -0.1279 | -0.711 | 8.769e-08 | 7.805e-06 | 2.562 | 1.357 | 8.153 | 0.7751 | 0.8932 | 1.034 | 0.04821 | 0.2895 |  |
| Rtp3 | receptor transporter protein 3 | 0.1341 | 0.4021 | 1.157 | 0.2102 | 1.562 | 0.7778 | 0.9198 | 0.9689 | -0.04556 | -0.2861 | 1.469e-07 | 1.138e-05 | 2.239 | 1.163 | 7.885 | 0.01436 | 0.07301 | 0.7603 | -0.3954 | -2.681 |  |
| P2ry10 | purinergic receptor P2Y, G-pro | 0.002245 | 0.03716 | 2.386 | 1.255 | 3.503 | 0.06088 | 0.3414 | 1.792 | 0.8419 | 1.986 | 0.2626 | 0.5149 | 1.368 | 0.4524 | 1.153 | 0.05897 | 0.1973 | 1.724 | 0.7859 | 2.003 |  |
| Slc9a2 | solute carrier family 9 (sodiu | 0.006191 | 0.06979 | 0.6755 | -0.566 | -3.06 | 0.777 | 0.9195 | 0.9574 | -0.06285 | -0.2871 | 0.09715 | 0.2784 | 0.7831 | -0.3527 | -1.741 | 1.388e-05 | 0.0003813 | 0.4485 | -1.157 | -5.708 |  |
| Gm13287 | predicted gene 13287 | 0.005143 | 0.06194 | 1.377 | 0.4619 | 3.141 | 0.6331 | 0.8575 | 1.06 | 0.08436 | 0.4849 | 7.097e-07 | 3.843e-05 | 2.209 | 1.143 | 7.097 | 0.9492 | 0.977 | 0.9928 | -0.01039 | -0.06448 |  |
| Stard8 | START domain containing 8 | 0.5455 | 0.7913 | 0.9472 | -0.07828 | -0.6149 | 0.152 | 0.5042 | 1.168 | 0.2243 | 1.49 | 0.1817 | 0.4119 | 1.143 | 0.193 | 1.384 | 1.442e-07 | 1.096e-05 | 0.4663 | -1.101 | -7.894 |  |
| Angpt1 | angiopoietin 1 | 0.004967 | 0.06038 | 0.4955 | -1.013 | -3.157 | 0.688 | 0.8831 | 0.8983 | -0.1547 | -0.4075 | 0.0001163 | 0.001942 | 0.3125 | -1.678 | -4.773 | 0.01903 | 0.08929 | 0.5371 | -0.8968 | -2.551 |  |
| Adam12 | a disintegrin and metallopepti | 0.5232 | 0.7771 | 0.9289 | -0.1064 | -0.6499 | 0.06572 | 0.3524 | 1.299 | 0.3771 | 1.947 | 1.604e-06 | 6.983e-05 | 0.4346 | -1.202 | -6.704 | 0.1813 | 0.4016 | 0.8418 | -0.2484 | -1.385 |  |
| Cxcr4 | chemokine (C-X-C motif) recept | 0.001099 | 0.02409 | 3.145 | 1.653 | 3.81 | 0.01486 | 0.1638 | 2.582 | 1.368 | 2.666 | 0.9327 | 0.9725 | 1.029 | 0.04061 | 0.08546 | 0.1027 | 0.2826 | 1.757 | 0.8129 | 1.71 |  |
| Gm7819 | NA | 0.0008547 | 0.02045 | 2.164 | 1.114 | 3.918 | 0.005181 | 0.08884 | 2.079 | 1.056 | 3.138 | 0.4583 | 0.6972 | 1.177 | 0.2356 | 0.7563 | 0.846 | 0.9297 | 0.9584 | -0.06131 | -0.1968 |  |
| Mdga1 | MAM domain containing glycosyl | 0.01047 | 0.09494 | 0.6947 | -0.5255 | -2.825 | 0.3223 | 0.6709 | 0.8566 | -0.2233 | -1.015 | 0.529 | 0.752 | 1.095 | 0.1305 | 0.6407 | 5.621e-05 | 0.001133 | 0.4873 | -1.037 | -5.089 |  |
| Tpgs2 | tubulin polyglutamylase comple | 0.006438 | 0.0716 | 0.5903 | -0.7604 | -3.042 | 0.8983 | 0.9658 | 0.9738 | -0.03829 | -0.1295 | 0.0008316 | 0.008799 | 0.4744 | -1.076 | -3.929 | 0.0007337 | 0.008058 | 0.4696 | -1.091 | -3.983 |  |
| Cyp2b10 | cytochrome P450, family 2, sub | 0.007452 | 0.07736 | 3.872 | 1.953 | 2.977 | 0.1266 | 0.4668 | 2.358 | 1.237 | 1.594 | 0.000959 | 0.009836 | 6.867 | 2.78 | 3.868 | 0.3894 | 0.6228 | 0.6451 | -0.6323 | -0.8799 |  |
| Ptch1 | patched 1 | 0.5257 | 0.7788 | 0.9402 | -0.089 | -0.6459 | 0.203 | 0.5658 | 1.16 | 0.2146 | 1.316 | 1.893e-07 | 1.4e-05 | 0.4443 | -1.171 | -7.755 | 0.1007 | 0.2791 | 1.197 | 0.2598 | 1.721 |  |
| Ppp1r3d | protein phosphatase 1, regulat | 0.003701 | 0.04968 | 0.617 | -0.6966 | -3.286 | 0.2716 | 0.6292 | 0.8215 | -0.2836 | -1.131 | 0.0002163 | 0.003133 | 0.4841 | -1.046 | -4.506 | 0.4171 | 0.6452 | 0.8751 | -0.1924 | -0.8287 |  |
| Cadm1 | cell adhesion molecule 1 | 0.006058 | 0.06916 | 0.6522 | -0.6167 | -3.069 | 0.6824 | 0.8807 | 0.9339 | -0.09871 | -0.4152 | 0.1225 | 0.3224 | 0.7819 | -0.3549 | -1.612 | 2.489e-05 | 0.000597 | 0.4356 | -1.199 | -5.448 |  |
| F730043M19Rik | RIKEN cDNA F730043M19 gene | 0.005948 | 0.06859 | 0.7003 | -0.514 | -3.077 | 0.8701 | 0.9562 | 0.9776 | -0.03273 | -0.1656 | 2.332e-05 | 0.0005611 | 0.4993 | -1.002 | -5.476 | 0.02547 | 0.1105 | 0.7362 | -0.4418 | -2.415 |  |
| Strip2 | striatin interacting protein 2 | 0.8875 | 0.9574 | 0.984 | -0.02324 | -0.1432 | 0.02318 | 0.209 | 1.387 | 0.4722 | 2.459 | 3.379e-08 | 3.694e-06 | 0.344 | -1.54 | -8.661 | 0.767 | 0.8886 | 1.038 | 0.0534 | 0.3004 |  |
| 4930445K14Rik | NA | 0.6488 | 0.85 | 0.9371 | -0.09366 | -0.4624 | 0.01479 | 0.1634 | 0.642 | -0.6394 | -2.668 | 8.112e-05 | 0.001457 | 2.134 | 1.094 | 4.929 | 0.02368 | 0.1046 | 1.457 | 0.5435 | 2.449 |  |
| 9030612E09Rik | RIKEN cDNA 9030612E09 gene | 0.00595 | 0.06859 | 0.6072 | -0.7198 | -3.077 | 0.5095 | 0.7924 | 0.8791 | -0.1859 | -0.6717 | 0.5267 | 0.7502 | 0.8919 | -0.1651 | -0.6443 | 1.742e-05 | 0.000454 | 0.3695 | -1.436 | -5.606 |  |
| Snhg5 | small nucleolar RNA host gene | 0.006584 | 0.07245 | 1.635 | 0.7097 | 3.032 | 0.8755 | 0.9578 | 1.031 | 0.04395 | 0.1587 | 0.001234 | 0.01201 | 1.951 | 0.9641 | 3.76 | 0.0006571 | 0.007416 | 2.047 | 1.033 | 4.03 |  |
| Efna2 | ephrin A2 | 0.006097 | 0.06933 | 0.715 | -0.484 | -3.066 | 0.9358 | 0.9792 | 0.9895 | -0.01523 | -0.08155 | 2.738e-06 | 0.0001066 | 0.4615 | -1.116 | -6.452 | 0.1082 | 0.2918 | 0.8174 | -0.2908 | -1.682 |  |
| Fstl1 | follistatin-like 1 | 0.002081 | 0.03579 | 0.5151 | -0.9571 | -3.535 | 0.002873 | 0.06107 | 0.4705 | -1.088 | -3.396 | 0.2975 | 0.5524 | 1.246 | 0.3172 | 1.07 | 0.8037 | 0.908 | 1.053 | 0.07469 | 0.2518 |  |
| Bpgm | 2,3-bisphosphoglycerate mutase | 0.005371 | 0.06385 | 0.6546 | -0.6112 | -3.122 | 0.3153 | 0.6652 | 0.8476 | -0.2386 | -1.03 | 0.9571 | 0.9829 | 1.008 | 0.01169 | 0.0545 | 3.741e-05 | 0.0008239 | 0.457 | -1.13 | -5.268 |  |
| Zfand2a | zinc finger, AN1-type domain 2 | 0.1838 | 0.4733 | 1.372 | 0.4564 | 1.377 | 0.08713 | 0.3991 | 1.631 | 0.7057 | 1.799 | 0.0001962 | 0.002897 | 0.3183 | -1.652 | -4.548 | 0.02176 | 0.09839 | 1.871 | 0.9038 | 2.489 |  |
| Arhgef37 | Rho guanine nucleotide exchang | 0.03312 | 0.1865 | 1.581 | 0.6604 | 2.289 | 0.5607 | 0.8189 | 0.8693 | -0.202 | -0.5917 | 1.629e-06 | 7.08e-05 | 4.337 | 2.117 | 6.696 | 0.1521 | 0.3614 | 1.386 | 0.4707 | 1.489 |  |
| Gm5558 | predicted gene 5558 | 0.001298 | 0.02689 | 0.4917 | -1.024 | -3.738 | 0.0136 | 0.1549 | 0.5444 | -0.8773 | -2.706 | 0.8824 | 0.9505 | 1.032 | 0.04495 | 0.1498 | 0.1214 | 0.3128 | 0.7142 | -0.4856 | -1.618 |  |
| Mapkbp1 | mitogen-activated protein kina | 0.1762 | 0.4623 | 1.236 | 0.3061 | 1.402 | 0.2125 | 0.574 | 0.7941 | -0.3327 | -1.288 | 2.714e-07 | 1.848e-05 | 3.509 | 1.811 | 7.573 | 0.6638 | 0.8264 | 1.076 | 0.1055 | 0.4412 |  |
| Ubac1 | ubiquitin associated domain co | 0.07598 | 0.2956 | 0.7856 | -0.3481 | -1.872 | 0.3898 | 0.7192 | 1.144 | 0.1935 | 0.8792 | 0.009074 | 0.0528 | 0.6649 | -0.5887 | -2.889 | 5.673e-05 | 0.001141 | 0.4877 | -1.036 | -5.085 |  |
| Tprn | taperin | 0.1429 | 0.4149 | 1.28 | 0.3557 | 1.525 | 0.2567 | 0.6161 | 0.7998 | -0.3222 | -1.168 | 2.902e-05 | 0.0006635 | 2.593 | 1.374 | 5.38 | 0.01786 | 0.08514 | 1.579 | 0.6594 | 2.581 |  |
| Flot2 | flotillin 2 | 0.2229 | 0.5226 | 0.8327 | -0.2642 | -1.258 | 0.1874 | 0.5493 | 1.265 | 0.3392 | 1.365 | 0.0001916 | 0.002851 | 0.4835 | -1.048 | -4.558 | 0.003496 | 0.02611 | 0.5899 | -0.7615 | -3.31 |  |
| Fzd4 | frizzled class receptor 4 | 0.1673 | 0.4497 | 1.275 | 0.3505 | 1.433 | 0.01801 | 0.1826 | 1.677 | 0.7457 | 2.577 | 6.184e-05 | 0.001182 | 0.3917 | -1.352 | -5.047 | 0.5411 | 0.7427 | 1.122 | 0.1666 | 0.6218 |  |
| Lmbr1 | limb region 1 | 0.7199 | 0.8864 | 1.042 | 0.05931 | 0.3638 | 0.08405 | 0.3943 | 1.275 | 0.3508 | 1.818 | 0.07078 | 0.2252 | 1.267 | 0.3409 | 1.909 | 1.299e-06 | 6.033e-05 | 0.4306 | -1.215 | -6.804 |  |
| S1pr3 | sphingosine-1-phosphate recept | 0.2687 | 0.5744 | 0.8262 | -0.2754 | -1.138 | 0.1622 | 0.5177 | 1.334 | 0.4158 | 1.452 | 0.0001761 | 0.00268 | 0.4297 | -1.219 | -4.594 | 0.004256 | 0.03017 | 0.5528 | -0.8552 | -3.225 |  |
| Wbp5 | NA | 0.218 | 0.5173 | 0.8215 | -0.2837 | -1.272 | 0.01013 | 0.1319 | 0.5948 | -0.7494 | -2.84 | 0.0004252 | 0.00529 | 2.042 | 1.03 | 4.216 | 0.1488 | 0.3566 | 1.29 | 0.3669 | 1.502 |  |
| Ces2g | carboxylesterase 2G | 0.1674 | 0.45 | 1.296 | 0.3738 | 1.433 | 0.2485 | 0.6081 | 0.7754 | -0.367 | -1.189 | 0.002524 | 0.02062 | 1.982 | 0.9866 | 3.452 | 0.0003311 | 0.004429 | 2.355 | 1.236 | 4.323 |  |
| Adgrl3 | adhesion G protein-coupled rec | 0.00477 | 0.05876 | 1.471 | 0.5566 | 3.175 | 0.3722 | 0.708 | 1.14 | 0.1894 | 0.9129 | 3.589e-05 | 0.0007805 | 2.021 | 1.015 | 5.286 | 0.6575 | 0.8223 | 1.062 | 0.08644 | 0.4501 |  |
| Dapp1 | dual adaptor for phosphotyrosi | 0.001547 | 0.02979 | 2.495 | 1.319 | 3.663 | 0.01954 | 0.1911 | 2.117 | 1.082 | 2.539 | 0.9224 | 0.9679 | 0.9734 | -0.03891 | -0.09863 | 0.07157 | 0.2248 | 1.683 | 0.7506 | 1.903 |  |
| Plekha8 | pleckstrin homology domain con | 0.7466 | 0.8991 | 0.9596 | -0.05955 | -0.3277 | 0.0417 | 0.2855 | 1.383 | 0.468 | 2.176 | 0.05252 | 0.1842 | 0.7524 | -0.4104 | -2.061 | 9.309e-06 | 0.0002787 | 0.4437 | -1.172 | -5.888 |  |
| Tead4 | TEA domain family member 4 | 0.6822 | 0.8698 | 0.9473 | -0.07806 | -0.4155 | 0.4781 | 0.7743 | 0.8946 | -0.1607 | -0.7229 | 9.586e-06 | 0.0002831 | 2.312 | 1.209 | 5.875 | 0.0001476 | 0.002364 | 0.5136 | -0.9612 | -4.67 |  |
| Rpsa | ribosomal protein SA | 0.1025 | 0.3459 | 0.7103 | -0.4935 | -1.711 | 0.00764 | 0.1133 | 0.4958 | -1.012 | -2.966 | 0.006321 | 0.04059 | 1.95 | 0.9636 | 3.05 | 0.07555 | 0.2324 | 1.508 | 0.5922 | 1.875 |  |
| Zfp655 | zinc finger protein 655 | 0.006301 | 0.07058 | 1.508 | 0.5922 | 3.052 | 0.9077 | 0.9692 | 1.019 | 0.02697 | 0.1175 | 3.779e-05 | 0.0008108 | 2.172 | 1.119 | 5.263 | 0.01337 | 0.06932 | 1.492 | 0.5769 | 2.714 |  |
| Plxnc1 | plexin C1 | 0.002348 | 0.03804 | 2.356 | 1.236 | 3.483 | 0.06301 | 0.3468 | 1.774 | 0.8267 | 1.969 | 0.1317 | 0.3378 | 1.527 | 0.6111 | 1.572 | 0.1282 | 0.3236 | 1.534 | 0.6171 | 1.587 |  |
| Prkcdbp | NA | 0.09038 | 0.3236 | 0.6332 | -0.6593 | -1.779 | 0.359 | 0.6983 | 1.33 | 0.4116 | 0.9389 | 0.0001086 | 0.001842 | 0.259 | -1.949 | -4.803 | 0.005486 | 0.03617 | 0.4165 | -1.263 | -3.113 |  |
| Cdk5 | cyclin-dependent kinase 5 | 0.5117 | 0.7688 | 0.9293 | -0.1058 | -0.6681 | 0.06794 | 0.3575 | 1.285 | 0.3615 | 1.93 | 1.981e-07 | 1.439e-05 | 0.3948 | -1.341 | -7.732 | 0.7292 | 0.868 | 0.9587 | -0.06087 | -0.351 |  |
| Pctp | phosphatidylcholine transfer p | 0.5556 | 0.7979 | 0.9031 | -0.147 | -0.5994 | 0.06791 | 0.3575 | 1.474 | 0.5601 | 1.93 | 0.0001204 | 0.001996 | 0.4123 | -1.278 | -4.758 | 0.005188 | 0.03472 | 0.5575 | -0.8429 | -3.138 |  |
| Depdc7 | DEP domain containing 7 | 0.583 | 0.8148 | 0.9213 | -0.1183 | -0.558 | 0.01761 | 0.1804 | 0.6377 | -0.6489 | -2.587 | 0.0472 | 0.1719 | 1.405 | 0.4911 | 2.115 | 0.0001252 | 0.002085 | 2.145 | 1.101 | 4.741 |  |
| Sh3bp2 | SH3-domain binding protein 2 | 0.2735 | 0.5793 | 1.141 | 0.1903 | 1.126 | 0.1697 | 0.5285 | 0.8208 | -0.2849 | -1.425 | 0.04317 | 0.1616 | 1.319 | 0.3999 | 2.159 | 2.02e-05 | 0.00051 | 2.036 | 1.026 | 5.54 |  |
| Parm1 | prostate androgen-regulated mu | 0.006465 | 0.07175 | 0.4543 | -1.138 | -3.04 | 0.8805 | 0.9596 | 0.9543 | -0.06742 | -0.1522 | 2.552e-05 | 0.0006045 | 0.2132 | -2.229 | -5.436 | 0.02643 | 0.1134 | 0.5059 | -0.983 | -2.397 |  |
| Rnu73b | U73B small nuclear RNA | 0.354 | 0.6505 | 1.274 | 0.3493 | 0.9489 | 0.1229 | 0.4611 | 0.6149 | -0.7016 | -1.611 | 0.000216 | 0.003133 | 3.524 | 1.817 | 4.507 | 0.003488 | 0.02606 | 2.523 | 1.335 | 3.311 |  |
| Dennd2a | DENN/MADD domain containing 2A | 0.09787 | 0.3377 | 1.217 | 0.2828 | 1.737 | 0.3264 | 0.6738 | 0.8742 | -0.1939 | -1.006 | 2.757e-07 | 1.861e-05 | 2.549 | 1.35 | 7.565 | 0.6569 | 0.822 | 1.057 | 0.08047 | 0.451 |  |
| Nwd1 | NACHT and WD repeat domain con | 0.007335 | 0.07668 | 0.7036 | -0.5071 | -2.984 | 0.8212 | 0.9388 | 0.9686 | -0.04603 | -0.229 | 0.1592 | 0.3795 | 0.8281 | -0.2722 | -1.462 | 7.792e-06 | 0.000244 | 0.463 | -1.111 | -5.969 |  |
| Pla2g7 | phospholipase A2, group VII (p | 0.1515 | 0.4275 | 1.341 | 0.4235 | 1.492 | 0.05582 | 0.3259 | 1.605 | 0.6822 | 2.031 | 0.1054 | 0.2933 | 1.441 | 0.5276 | 1.696 | 0.0004505 | 0.005569 | 0.4051 | -1.304 | -4.192 |  |
| Gabrr1 | gamma-aminobutyric acid (GABA) | 0.1559 | 0.4336 | 1.194 | 0.2561 | 1.475 | 0.2422 | 0.6017 | 0.8422 | -0.2477 | -1.205 | 0.8703 | 0.9448 | 0.9784 | -0.03147 | -0.1654 | 9.517e-08 | 8.094e-06 | 2.914 | 1.543 | 8.11 |  |
| Slc25a42 | solute carrier family 25, memb | 0.02328 | 0.1512 | 1.364 | 0.4477 | 2.457 | 0.6724 | 0.8754 | 0.9379 | -0.09255 | -0.4292 | 1.327e-06 | 6.115e-05 | 2.56 | 1.356 | 6.795 | 0.2001 | 0.4256 | 1.201 | 0.2645 | 1.325 |  |
| Fam219a | family with sequence similarit | 0.03955 | 0.2059 | 1.223 | 0.2901 | 2.202 | 0.5148 | 0.795 | 0.9309 | -0.1033 | -0.6631 | 0.7039 | 0.8599 | 1.039 | 0.05563 | 0.3856 | 1.752e-07 | 1.251e-05 | 2.18 | 1.125 | 7.795 |  |
| Cd68 | CD68 antigen | 0.007282 | 0.07652 | 2.005 | 1.004 | 2.988 | 0.8453 | 0.9484 | 1.056 | 0.07859 | 0.1977 | 0.003668 | 0.02713 | 2.314 | 1.211 | 3.289 | 0.0003912 | 0.005016 | 2.958 | 1.565 | 4.252 |  |
| AB124611 | cDNA sequence AB124611 | 0.002954 | 0.04365 | 2.655 | 1.409 | 3.384 | 0.1016 | 0.4248 | 1.797 | 0.8456 | 1.716 | 0.08202 | 0.2487 | 1.784 | 0.8353 | 1.831 | 0.07569 | 0.2327 | 1.808 | 0.8546 | 1.874 |  |
| Stk11ip | serine/threonine kinase 11 int | 0.004984 | 0.06053 | 1.421 | 0.5065 | 3.155 | 0.9565 | 0.9863 | 1.007 | 0.0105 | 0.05527 | 0.8864 | 0.952 | 1.018 | 0.02544 | 0.1447 | 6.417e-08 | 6.002e-06 | 2.756 | 1.463 | 8.317 |  |
| Hacd1 | 3-hydroxyacyl-CoA dehydratase | 0.05499 | 0.2481 | 0.8067 | -0.3099 | -2.038 | 0.4722 | 0.7708 | 1.096 | 0.1318 | 0.7327 | 0.3458 | 0.6003 | 0.8945 | -0.1608 | -0.9656 | 8.824e-07 | 4.39e-05 | 0.4462 | -1.164 | -6.991 |  |
| Angptl4 | angiopoietin-like 4 | 0.002847 | 0.04258 | 2.187 | 1.129 | 3.4 | 0.005759 | 0.09478 | 2.321 | 1.215 | 3.092 | 0.2435 | 0.4924 | 0.7386 | -0.4371 | -1.202 | 0.6266 | 0.8022 | 1.133 | 0.1797 | 0.4941 |  |
| Gm23264 | predicted gene, 23264 | 0.001427 | 0.02843 | 2.068 | 1.048 | 3.698 | 0.01825 | 0.1842 | 1.818 | 0.8625 | 2.571 | 0.1121 | 0.3049 | 1.43 | 0.5163 | 1.662 | 0.8948 | 0.9527 | 1.029 | 0.0416 | 0.1339 |  |
| Ablim1 | actin-binding LIM protein 1 | 0.007189 | 0.07593 | 1.743 | 0.8018 | 2.993 | 0.9066 | 0.9691 | 1.026 | 0.03767 | 0.1189 | 0.0006102 | 0.006964 | 2.284 | 1.192 | 4.062 | 0.001261 | 0.01209 | 2.144 | 1.101 | 3.751 |  |
| Sult1c2 | sulfotransferase family, cytos | 0.2911 | 0.5953 | 0.8166 | -0.2924 | -1.084 | 0.01293 | 0.1512 | 0.5469 | -0.8707 | -2.729 | 0.03169 | 0.1306 | 1.605 | 0.6823 | 2.31 | 0.001945 | 0.0168 | 2.075 | 1.053 | 3.565 |  |
| Tmeff2 | transmembrane protein with EGF | 0.1187 | 0.3756 | 1.287 | 0.364 | 1.63 | 0.3174 | 0.6668 | 0.8288 | -0.2708 | -1.025 | 8.195e-06 | 0.0002534 | 2.74 | 1.454 | 5.946 | 0.08044 | 0.2423 | 1.366 | 0.4503 | 1.842 |  |
| Ndn | necdin | 0.02861 | 0.171 | 0.6422 | -0.6389 | -2.359 | 0.6494 | 0.8648 | 1.108 | 0.1479 | 0.4615 | 0.0002386 | 0.003373 | 0.3993 | -1.324 | -4.464 | 0.002297 | 0.01904 | 0.4876 | -1.036 | -3.493 |  |
| E030011O05Rik | NA | 0.00603 | 0.06904 | 1.581 | 0.661 | 3.071 | 0.6755 | 0.8768 | 1.078 | 0.1082 | 0.4249 | 1.01e-06 | 4.996e-05 | 3.101 | 1.633 | 6.925 | 0.9144 | 0.9617 | 1.018 | 0.02566 | 0.1088 |  |
| Akr1c14 | aldo-keto reductase family 1, | 0.07984 | 0.3016 | 0.6716 | -0.5744 | -1.846 | 0.01999 | 0.1937 | 0.5244 | -0.9311 | -2.528 | 0.0006413 | 0.007212 | 2.598 | 1.378 | 4.04 | 0.378 | 0.6119 | 0.8081 | -0.3074 | -0.9017 |  |
| Kcne4 | potassium voltage-gated channe | 0.1776 | 0.4647 | 1.338 | 0.4203 | 1.397 | 0.2381 | 0.5979 | 0.7408 | -0.4328 | -1.216 | 1.696e-05 | 0.0004372 | 3.607 | 1.851 | 5.618 | 0.04401 | 0.1615 | 1.634 | 0.7082 | 2.15 |  |
| Cth | cystathionase (cystathionine g | 0.9891 | 0.9963 | 1.002 | 0.002837 | 0.0139 | 0.03782 | 0.2731 | 1.451 | 0.5372 | 2.224 | 1.106e-07 | 9.121e-06 | 0.288 | -1.796 | -8.032 | 0.3989 | 0.6307 | 1.143 | 0.1928 | 0.8621 |  |
| Upp1 | uridine phosphorylase 1 | 0.2556 | 0.5598 | 1.15 | 0.2017 | 1.17 | 0.1806 | 0.5411 | 0.8219 | -0.2829 | -1.387 | 8.492e-06 | 0.0002597 | 2.173 | 1.119 | 5.93 | 0.09179 | 0.263 | 1.261 | 0.3343 | 1.771 |  |
| Snapin | SNAP-associated protein | 0.3289 | 0.6287 | 0.8948 | -0.1603 | -1.001 | 0.1359 | 0.4782 | 1.226 | 0.2945 | 1.554 | 6.235e-06 | 0.0002061 | 0.4779 | -1.065 | -6.071 | 0.1036 | 0.2843 | 0.8127 | -0.2992 | -1.705 |  |
| Il4ra | interleukin 4 receptor, alpha | 0.004256 | 0.0544 | 1.945 | 0.96 | 3.225 | 0.3264 | 0.6738 | 1.278 | 0.3544 | 1.006 | 0.005872 | 0.03843 | 2.008 | 1.005 | 3.083 | 0.02182 | 0.0985 | 1.755 | 0.8112 | 2.488 |  |
| Hsf2 | heat shock factor 2 | 0.03073 | 0.1782 | 1.258 | 0.3314 | 2.325 | 0.6382 | 0.8598 | 0.9457 | -0.08051 | -0.4774 | 0.1643 | 0.3871 | 1.169 | 0.2254 | 1.444 | 2.631e-06 | 0.0001028 | 2.014 | 1.01 | 6.47 |  |
| Dhrs1 | dehydrogenase/reductase (SDR f | 0.07281 | 0.2883 | 0.7706 | -0.3759 | -1.894 | 0.4244 | 0.7434 | 1.142 | 0.1915 | 0.8155 | 5.536e-06 | 0.0001871 | 0.3973 | -1.332 | -6.125 | 0.1039 | 0.2847 | 0.7735 | -0.3705 | -1.704 |  |
| Slc25a30 | solute carrier family 25, memb | 0.07419 | 0.2912 | 1.434 | 0.5198 | 1.884 | 0.4208 | 0.7412 | 0.8303 | -0.2683 | -0.8219 | 0.0001623 | 0.002512 | 2.638 | 1.399 | 4.63 | 0.004569 | 0.03165 | 1.952 | 0.9652 | 3.193 |  |
| Slc8b1 | solute carrier family 8 (sodiu | 0.01293 | 0.1073 | 1.37 | 0.4547 | 2.729 | 0.8618 | 0.9535 | 0.9762 | -0.03477 | -0.1764 | 8.751e-07 | 4.52e-05 | 2.423 | 1.277 | 6.995 | 0.3058 | 0.547 | 1.142 | 0.1918 | 1.051 |  |
| Ighv14-2 | immunoglobulin heavy variable | 0.001421 | 0.02833 | 7.39 | 2.886 | 3.7 | 0.007337 | 0.1108 | 6.747 | 2.754 | 2.984 | 0.7459 | 0.8824 | 0.8232 | -0.2807 | -0.3285 | 0.4388 | 0.664 | 1.597 | 0.675 | 0.79 |  |
| Slc35a4 | solute carrier family 35, memb | 0.5875 | 0.817 | 1.095 | 0.1316 | 0.5513 | 0.01649 | 0.1725 | 1.669 | 0.7391 | 2.618 | 0.0003548 | 0.004596 | 0.4593 | -1.122 | -4.294 | 0.01444 | 0.07324 | 0.6155 | -0.7003 | -2.679 |  |
| Rnf144b | ring finger protein 144B | 0.9655 | 0.9881 | 1.006 | 0.008423 | 0.04382 | 0.02336 | 0.2099 | 0.679 | -0.5584 | -2.455 | 1.381e-05 | 0.0003743 | 2.301 | 1.202 | 5.71 | 0.0282 | 0.1184 | 1.412 | 0.4982 | 2.366 |  |
| Ankrd28 | ankyrin repeat domain 28 | 0.6459 | 0.8491 | 1.052 | 0.07324 | 0.4665 | 0.01966 | 0.1917 | 1.386 | 0.4712 | 2.536 | 0.6269 | 0.8144 | 0.9428 | -0.08493 | -0.4938 | 2.764e-06 | 0.0001063 | 0.4636 | -1.109 | -6.447 |  |
| Thumpd3 | THUMP domain containing 3 | 0.144 | 0.4168 | 1.258 | 0.331 | 1.521 | 0.01081 | 0.1368 | 1.652 | 0.7238 | 2.811 | 0.0001902 | 0.002839 | 0.4706 | -1.088 | -4.561 | 0.7066 | 0.8544 | 0.9389 | -0.09103 | -0.3818 |  |
| Rsad2 | radical S-adenosyl methionine | 0.1668 | 0.4493 | 1.361 | 0.4451 | 1.435 | 0.01087 | 0.1372 | 2.043 | 1.031 | 2.808 | 0.0006811 | 0.007562 | 0.3884 | -1.364 | -4.015 | 0.2617 | 0.499 | 0.7618 | -0.3926 | -1.155 |  |
| Zfp930 | zinc finger protein 930 | 0.08272 | 0.3075 | 0.6972 | -0.5204 | -1.827 | 0.008704 | 0.1212 | 0.5069 | -0.9803 | -2.908 | 0.001731 | 0.01549 | 2.186 | 1.128 | 3.615 | 0.4299 | 0.6566 | 1.19 | 0.2514 | 0.8056 |  |
| Pqlc3 | PQ loop repeat containing | 0.4396 | 0.72 | 1.15 | 0.2011 | 0.7886 | 0.01512 | 0.165 | 1.743 | 0.8019 | 2.658 | 4.492e-05 | 0.0009237 | 0.3662 | -1.449 | -5.187 | 0.2205 | 0.4507 | 0.7828 | -0.3533 | -1.265 |  |
| Dcst1 | DC-STAMP domain containing 1 | 0.03278 | 0.1854 | 1.352 | 0.4351 | 2.294 | 0.6199 | 0.8507 | 0.9246 | -0.1131 | -0.5038 | 9.419e-07 | 4.798e-05 | 2.725 | 1.446 | 6.959 | 0.3508 | 0.5889 | 1.148 | 0.1985 | 0.9554 |  |
| Serf1 | small EDRK-rich factor 1 | 0.004371 | 0.05546 | 0.5471 | -0.8701 | -3.213 | 0.2488 | 0.6085 | 0.7681 | -0.3806 | -1.188 | 0.001726 | 0.01548 | 0.4754 | -1.073 | -3.616 | 0.1973 | 0.4223 | 0.7602 | -0.3956 | -1.334 |  |
| Ffar2 | free fatty acid receptor 2 | 0.009456 | 0.08932 | 0.6526 | -0.6157 | -2.871 | 0.9461 | 0.9829 | 1.012 | 0.01736 | 0.06843 | 5.812e-05 | 0.001124 | 0.4376 | -1.192 | -5.075 | 0.006935 | 0.04306 | 0.6126 | -0.707 | -3.009 |  |
| Nuak1 | NUAK family, SNF1-like kinase, | 0.0868 | 0.3166 | 1.341 | 0.4237 | 1.801 | 0.3945 | 0.7236 | 0.8455 | -0.2422 | -0.8702 | 0.2461 | 0.4951 | 1.238 | 0.3079 | 1.195 | 2.186e-06 | 8.981e-05 | 3.226 | 1.69 | 6.557 |  |
| Hrct1 | histidine rich carboxyl termin | 0.4471 | 0.7258 | 1.113 | 0.1547 | 0.7755 | 0.09625 | 0.4152 | 0.7516 | -0.412 | -1.746 | 0.0001131 | 0.001896 | 2.064 | 1.046 | 4.786 | 0.00717 | 0.04411 | 1.574 | 0.6544 | 2.995 |  |
| Wdr93 | WD repeat domain 93 | 0.2424 | 0.5454 | 0.8408 | -0.2502 | -1.205 | 0.1881 | 0.5497 | 1.261 | 0.3349 | 1.363 | 4.947e-05 | 0.0009934 | 0.4443 | -1.17 | -5.145 | 0.01828 | 0.08668 | 0.6668 | -0.5847 | -2.57 |  |
| Clmn | calmin | 0.06442 | 0.2702 | 1.377 | 0.4619 | 1.957 | 0.5527 | 0.8156 | 0.8897 | -0.1686 | -0.6039 | 1.175e-07 | 9.449e-06 | 4.193 | 2.068 | 8 | 0.5025 | 0.7147 | 0.8848 | -0.1765 | -0.6828 |  |
| Crlf3 | cytokine receptor-like factor | 0.003382 | 0.04716 | 2.101 | 1.071 | 3.325 | 0.1008 | 0.4234 | 1.576 | 0.6559 | 1.72 | 0.2148 | 0.4561 | 1.368 | 0.4522 | 1.281 | 0.03588 | 0.1401 | 1.734 | 0.7942 | 2.25 |  |
| Snca | synuclein, alpha | 0.2728 | 0.5786 | 0.7905 | -0.3392 | -1.128 | 0.01212 | 0.1459 | 0.5063 | -0.9818 | -2.759 | 0.003442 | 0.02583 | 2.133 | 1.093 | 3.317 | 0.01976 | 0.09172 | 1.784 | 0.8349 | 2.534 |  |
| Srms | src-related kinase lacking C-t | 0.5104 | 0.768 | 1.084 | 0.1163 | 0.6702 | 0.08548 | 0.3963 | 0.773 | -0.3714 | -1.809 | 1.855e-05 | 0.0004685 | 2.085 | 1.06 | 5.578 | 0.04673 | 0.1684 | 1.322 | 0.4029 | 2.12 |  |
| Ighv6-7 | immunoglobulin heavy variable | 0.479 | 0.7479 | 0.8004 | -0.3212 | -0.7214 | 0.0943 | 0.412 | 1.899 | 0.9253 | 1.757 | 0.0005375 | 0.006321 | 0.2487 | -2.007 | -4.116 | 0.002004 | 0.0172 | 0.301 | -1.732 | -3.552 |  |
| Gm5815 | predicted pseudogene 5815 | 0.06072 | 0.2614 | 1.296 | 0.3736 | 1.988 | 0.6101 | 0.8449 | 1.083 | 0.1152 | 0.518 | 0.04011 | 0.154 | 0.731 | -0.4521 | -2.196 | 7.936e-06 | 0.0002465 | 2.341 | 1.227 | 5.961 |  |
| Crem | cAMP responsive element modula | 0.2046 | 0.4999 | 0.8025 | -0.3174 | -1.311 | 0.01245 | 0.1482 | 0.5797 | -0.7866 | -2.747 | 8.784e-05 | 0.001554 | 2.459 | 1.298 | 4.895 | 0.6833 | 0.8404 | 1.079 | 0.1098 | 0.414 |  |
| Sel1l3 | sel-1 suppressor of lin-12-lik | 0.006007 | 0.0689 | 0.5693 | -0.8126 | -3.073 | 0.2386 | 0.5984 | 0.7684 | -0.3801 | -1.215 | 0.6903 | 0.8522 | 0.922 | -0.1171 | -0.4043 | 0.0005917 | 0.006865 | 0.4412 | -1.18 | -4.075 |  |
| Trim67 | tripartite motif-containing 67 | 0.22 | 0.5196 | 0.7757 | -0.3665 | -1.266 | 0.2121 | 0.5736 | 1.358 | 0.4414 | 1.289 | 6.703e-06 | 0.0002177 | 0.2654 | -1.914 | -6.038 | 0.1237 | 0.3163 | 0.7024 | -0.5095 | -1.607 |  |
| Epb41l2 | erythrocyte membrane protein b | 0.01156 | 0.1001 | 0.7161 | -0.4818 | -2.78 | 0.9042 | 0.9682 | 1.017 | 0.025 | 0.1219 | 3.15e-06 | 0.0001188 | 0.4316 | -1.212 | -6.386 | 0.1204 | 0.3116 | 0.8078 | -0.308 | -1.622 |  |
| Nrn1 | neuritin 1 | 0.6891 | 0.8725 | 1.067 | 0.09412 | 0.406 | 0.05791 | 0.3328 | 0.6821 | -0.5519 | -2.012 | 0.002326 | 0.01934 | 1.848 | 0.8856 | 3.487 | 0.0005034 | 0.006053 | 2.074 | 1.052 | 4.144 |  |
| Ralgps2 | Ral GEF with PH domain and SH3 | 0.002523 | 0.03955 | 3.221 | 1.687 | 3.452 | 0.01488 | 0.1639 | 2.911 | 1.541 | 2.665 | 0.6129 | 0.8056 | 0.8263 | -0.2752 | -0.514 | 0.1978 | 0.4228 | 1.64 | 0.7133 | 1.332 |  |
| Postn | periostin, osteoblast specific | 0.0102 | 0.09358 | 2.023 | 1.016 | 2.837 | 0.1317 | 0.4723 | 1.587 | 0.6664 | 1.572 | 0.001789 | 0.01589 | 2.663 | 1.413 | 3.601 | 0.3661 | 0.6019 | 0.7776 | -0.363 | -0.9247 |  |
| Slc4a4 | solute carrier family 4 (anion | 0.8832 | 0.956 | 1.027 | 0.03826 | 0.1488 | 0.2628 | 0.6203 | 1.275 | 0.3506 | 1.152 | 0.003088 | 0.02389 | 1.929 | 0.9478 | 3.364 | 1.648e-06 | 7.261e-05 | 0.2708 | -1.885 | -6.691 |  |
| Esrrg | estrogen-related receptor gamm | 0.7776 | 0.9124 | 0.9525 | -0.07027 | -0.2863 | 0.04587 | 0.2983 | 1.535 | 0.6184 | 2.129 | 0.00133 | 0.01281 | 0.4992 | -1.002 | -3.728 | 0.0007823 | 0.008466 | 0.4785 | -1.064 | -3.955 |  |
| Hvcn1 | hydrogen voltage-gated channel | 0.04544 | 0.2228 | 2.458 | 1.298 | 2.134 | 0.02432 | 0.2145 | 3.371 | 1.753 | 2.436 | 0.003991 | 0.02896 | 0.2227 | -2.167 | -3.253 | 0.2436 | 0.4778 | 1.742 | 0.8004 | 1.202 |  |
| Hnrnpf | heterogeneous nuclear ribonucl | 0.02182 | 0.1458 | 1.416 | 0.502 | 2.487 | 0.7665 | 0.9153 | 0.9514 | -0.07187 | -0.301 | 0.02753 | 0.1186 | 1.44 | 0.5256 | 2.378 | 2.709e-05 | 0.0006363 | 2.291 | 1.196 | 5.41 |  |
| Sertad2 | SERTA domain containing 2 | 0.5906 | 0.8187 | 1.079 | 0.1094 | 0.5468 | 0.0764 | 0.3788 | 0.7359 | -0.4425 | -1.869 | 0.1396 | 0.3505 | 1.263 | 0.3373 | 1.539 | 7.056e-06 | 0.0002255 | 2.494 | 1.318 | 6.014 |  |
| Filip1 | filamin A interacting protein | 0.05325 | 0.2438 | 1.272 | 0.3474 | 2.054 | 0.6237 | 0.8523 | 0.9332 | -0.09968 | -0.4983 | 0.5267 | 0.7502 | 0.9206 | -0.1193 | -0.6443 | 1.468e-07 | 1.105e-05 | 2.752 | 1.461 | 7.885 |  |
| Osbpl6 | oxysterol binding protein-like | 0.6123 | 0.8317 | 0.9163 | -0.1261 | -0.5148 | 0.2632 | 0.6206 | 0.7936 | -0.3336 | -1.151 | 1.346e-05 | 0.0003668 | 2.897 | 1.535 | 5.722 | 0.002841 | 0.02226 | 0.5314 | -0.9121 | -3.401 |  |
| Gm28041 | predicted gene, 28041 | 0.18 | 0.4684 | 1.252 | 0.3237 | 1.389 | 0.2619 | 0.6201 | 0.802 | -0.3183 | -1.155 | 0.00395 | 0.02872 | 1.779 | 0.8314 | 3.257 | 0.000359 | 0.004702 | 2.136 | 1.095 | 4.289 |  |
| Adgrg6 | adhesion G protein-coupled rec | 0.1024 | 0.3456 | 1.319 | 0.399 | 1.712 | 0.3804 | 0.7123 | 0.8424 | -0.2474 | -0.897 | 0.003057 | 0.02371 | 1.815 | 0.8602 | 3.369 | 0.0004159 | 0.005252 | 2.113 | 1.079 | 4.226 |  |
| Coro2a | coronin, actin binding protein | 0.003493 | 0.04808 | 2.162 | 1.113 | 3.311 | 0.1095 | 0.4397 | 1.587 | 0.666 | 1.675 | 0.0538 | 0.187 | 1.687 | 0.7544 | 2.049 | 0.1275 | 0.3227 | 1.5 | 0.5853 | 1.59 |  |
| Zc3h12c | zinc finger CCCH type containi | 0.009102 | 0.08751 | 0.7021 | -0.5103 | -2.888 | 0.9592 | 0.987 | 0.9925 | -0.01084 | -0.05187 | 0.06646 | 0.2161 | 0.7707 | -0.3758 | -1.941 | 1.132e-05 | 0.0003251 | 0.4593 | -1.123 | -5.8 |  |
| Nol6 | nucleolar protein family 6 (RN | 0.111 | 0.3613 | 0.8462 | -0.2409 | -1.668 | 0.936 | 0.9792 | 0.9904 | -0.0139 | -0.08131 | 5.704e-07 | 3.225e-05 | 0.4538 | -1.14 | -7.203 | 0.008863 | 0.05141 | 1.374 | 0.4589 | 2.9 |  |
| Zbed4 | zinc finger, BED type containi | 0.9373 | 0.9779 | 1.011 | 0.01637 | 0.07962 | 0.0248 | 0.2167 | 1.506 | 0.5903 | 2.427 | 0.1058 | 0.2937 | 0.7677 | -0.3815 | -1.694 | 8.079e-06 | 0.0002492 | 0.3949 | -1.34 | -5.953 |  |
| Runx1 | runt related transcription fac | 0.02479 | 0.1573 | 1.551 | 0.6335 | 2.427 | 0.2239 | 0.5848 | 1.308 | 0.3876 | 1.255 | 0.1771 | 0.4057 | 0.7578 | -0.4 | -1.399 | 0.0007395 | 0.008104 | 2.2 | 1.138 | 3.98 |  |
| Crim1 | cysteine rich transmembrane BM | 0.006425 | 0.07155 | 1.281 | 0.3573 | 3.043 | 0.9626 | 0.9879 | 1.005 | 0.006603 | 0.04752 | 9.667e-08 | 8.373e-06 | 2.059 | 1.042 | 8.102 | 0.9383 | 0.9718 | 1.007 | 0.01009 | 0.07841 |  |
| Gde1 | glycerophosphodiester phosphod | 0.2572 | 0.5618 | 1.2 | 0.2632 | 1.166 | 0.08498 | 0.3959 | 1.398 | 0.4838 | 1.812 | 0.0653 | 0.2133 | 1.397 | 0.482 | 1.95 | 0.0001625 | 0.002533 | 0.4525 | -1.144 | -4.629 |  |
| Gm22951 | predicted gene, 22951 | 0.1005 | 0.3423 | 1.408 | 0.4935 | 1.722 | 0.06477 | 0.3493 | 1.583 | 0.6628 | 1.955 | 0.07061 | 0.2249 | 1.515 | 0.5996 | 1.91 | 0.002121 | 0.01796 | 0.4642 | -1.107 | -3.527 |  |
| Gkn3 | gastrokine 3 | 0.01838 | 0.1317 | 0.7439 | -0.4269 | -2.568 | 0.7955 | 0.9276 | 1.036 | 0.05167 | 0.2626 | 3.33e-06 | 0.0001238 | 0.448 | -1.158 | -6.36 | 0.1435 | 0.3476 | 0.8251 | -0.2773 | -1.523 |  |
| Dlx6 | distal-less homeobox 6 | 0.9428 | 0.9801 | 1.015 | 0.02172 | 0.07266 | 0.02413 | 0.2137 | 1.819 | 0.8629 | 2.44 | 0.009972 | 0.05657 | 0.5241 | -0.9321 | -2.847 | 9.501e-05 | 0.001694 | 0.3318 | -1.592 | -4.861 |  |
| Sod2 | superoxide dismutase 2, mitoch | 0.06842 | 0.2791 | 0.8091 | -0.3055 | -1.926 | 0.4617 | 0.7657 | 1.103 | 0.1408 | 0.7504 | 4.582e-06 | 0.0001608 | 0.4732 | -1.079 | -6.212 | 0.1506 | 0.3592 | 0.8353 | -0.2597 | -1.495 |  |
| Zfyve21 | zinc finger, FYVE domain conta | 0.6369 | 0.8448 | 0.9401 | -0.08907 | -0.4793 | 0.1094 | 0.4396 | 0.7746 | -0.3684 | -1.675 | 0.0677 | 0.2188 | 0.7614 | -0.3933 | -1.932 | 6.549e-06 | 0.0002135 | 2.348 | 1.231 | 6.048 |  |
| 9830004L10Rik | RIKEN cDNA 9830004L10 gene | 0.009395 | 0.08895 | 0.6482 | -0.6255 | -2.874 | 0.8497 | 0.9501 | 0.9663 | -0.04945 | -0.192 | 0.0878 | 0.2607 | 0.7433 | -0.428 | -1.795 | 2.458e-05 | 0.0005918 | 0.4061 | -1.3 | -5.453 |  |
| Ighv5-12 | immunoglobulin heavy variable | 0.001774 | 0.0325 | 6.181 | 2.628 | 3.604 | 0.01838 | 0.1847 | 4.643 | 2.215 | 2.567 | 0.7994 | 0.9104 | 1.153 | 0.2057 | 0.2575 | 0.2108 | 0.439 | 2.046 | 1.033 | 1.293 |  |
| Gm23677 | predicted gene, 23677 | 0.2201 | 0.5196 | 0.8019 | -0.3185 | -1.266 | 0.6157 | 0.8483 | 1.111 | 0.1518 | 0.51 | 0.02894 | 0.1228 | 1.568 | 0.6487 | 2.354 | 3.31e-07 | 2.013e-05 | 0.2399 | -2.059 | -7.473 |  |
| C4a | complement component 4A (Rodge | 0.5756 | 0.8105 | 0.8845 | -0.1771 | -0.5691 | 0.01906 | 0.1886 | 0.5216 | -0.9391 | -2.551 | 0.005399 | 0.03609 | 2.09 | 1.063 | 3.12 | 0.001847 | 0.01615 | 2.334 | 1.223 | 3.587 |  |
| Acyp1 | acylphosphatase 1, erythrocyte | 0.01385 | 0.1115 | 1.524 | 0.6077 | 2.698 | 0.2695 | 0.6273 | 1.233 | 0.3027 | 1.136 | 0.5324 | 0.7542 | 0.897 | -0.1568 | -0.6353 | 0.0003159 | 0.004281 | 2.102 | 1.072 | 4.343 |  |
| Otub2 | OTU domain, ubiquitin aldehyde | 0.009474 | 0.08943 | 0.4856 | -1.042 | -2.87 | 0.123 | 0.4611 | 0.619 | -0.692 | -1.61 | 0.523 | 0.7473 | 1.196 | 0.2586 | 0.6501 | 0.003652 | 0.02696 | 0.4035 | -1.309 | -3.291 |  |
| Aff3 | AF4/FMR2 family, member 3 | 0.002756 | 0.04164 | 2.284 | 1.191 | 3.414 | 0.04145 | 0.285 | 1.866 | 0.9 | 2.179 | 0.7296 | 0.8748 | 1.097 | 0.134 | 0.3505 | 0.0671 | 0.2155 | 1.671 | 0.7404 | 1.936 |  |
| Alg12 | asparagine-linked glycosylatio | 0.1527 | 0.4294 | 1.284 | 0.361 | 1.487 | 0.01207 | 0.1457 | 1.733 | 0.7932 | 2.761 | 0.0006327 | 0.00716 | 0.4742 | -1.076 | -4.046 | 0.4177 | 0.6458 | 0.8585 | -0.2202 | -0.8276 |  |
| Gm6517 | predicted gene 6517 | 0.4517 | 0.7282 | 0.8635 | -0.2117 | -0.7676 | 0.01732 | 0.1786 | 0.556 | -0.8468 | -2.595 | 0.001212 | 0.01185 | 2.201 | 1.138 | 3.768 | 0.01952 | 0.09087 | 1.702 | 0.7671 | 2.54 |  |
| Orm3 | orosomucoid 3 | 0.06212 | 0.2647 | 0.6282 | -0.6707 | -1.976 | 0.4946 | 0.7838 | 1.214 | 0.2794 | 0.6957 | 3.894e-06 | 0.0001401 | 0.1978 | -2.338 | -6.288 | 0.1833 | 0.4039 | 0.701 | -0.5125 | -1.378 |  |
| Cmklr1 | chemokine-like receptor 1 | 0.09886 | 0.3398 | 1.389 | 0.4736 | 1.731 | 0.3805 | 0.7123 | 0.8177 | -0.2904 | -0.8969 | 0.0001567 | 0.002438 | 2.625 | 1.392 | 4.645 | 0.007048 | 0.04361 | 1.866 | 0.8998 | 3.002 |  |
| Cdc42ep1 | CDC42 effector protein (Rho GT | 0.377 | 0.6709 | 0.8907 | -0.167 | -0.9035 | 0.134 | 0.4755 | 1.267 | 0.3416 | 1.562 | 8.561e-06 | 0.0002609 | 0.4353 | -1.2 | -5.926 | 0.1233 | 0.3158 | 0.7979 | -0.3258 | -1.609 |  |
| Slc6a13 | solute carrier family 6 (neuro | 0.01321 | 0.1086 | 0.5803 | -0.7852 | -2.72 | 0.9055 | 0.9684 | 1.029 | 0.04108 | 0.1202 | 2.529e-05 | 0.0005997 | 0.3034 | -1.721 | -5.44 | 0.02642 | 0.1134 | 0.5912 | -0.7582 | -2.397 |  |
| Mttp | microsomal triglyceride transf | 0.4963 | 0.7588 | 1.085 | 0.1182 | 0.693 | 0.0399 | 0.2793 | 1.36 | 0.4437 | 2.198 | 0.5434 | 0.7617 | 1.083 | 0.1155 | 0.6182 | 1.029e-05 | 0.0003022 | 0.4691 | -1.092 | -5.843 |  |
| Eln | elastin | 0.006905 | 0.07447 | 1.78 | 0.8318 | 3.011 | 0.52 | 0.7979 | 1.16 | 0.214 | 0.6549 | 0.0002303 | 0.003291 | 2.558 | 1.355 | 4.479 | 0.1155 | 0.3038 | 1.412 | 0.4979 | 1.646 |  |
| Amot | angiomotin | 0.7165 | 0.885 | 1.057 | 0.07954 | 0.3683 | 0.02438 | 0.2149 | 1.539 | 0.6222 | 2.435 | 0.02732 | 0.1179 | 0.6768 | -0.5633 | -2.381 | 0.0002188 | 0.003211 | 0.4781 | -1.065 | -4.501 |  |
| Pcdhb17 | protocadherin beta 17 | 0.129 | 0.3931 | 0.7612 | -0.3936 | -1.584 | 0.3383 | 0.6834 | 1.221 | 0.2884 | 0.981 | 0.001732 | 0.01549 | 0.5056 | -0.984 | -3.614 | 0.0008875 | 0.009299 | 0.4789 | -1.062 | -3.901 |  |
| Fam213a | NA | 0.2127 | 0.5109 | 1.19 | 0.2505 | 1.287 | 0.2333 | 0.5947 | 0.8219 | -0.283 | -1.229 | 3.462e-05 | 0.0007631 | 2.189 | 1.13 | 5.302 | 0.03922 | 0.149 | 1.385 | 0.4704 | 2.207 |  |
| Repin1 | replication initiator 1 | 0.5079 | 0.7661 | 0.9183 | -0.123 | -0.6742 | 0.02285 | 0.2075 | 0.6915 | -0.5322 | -2.466 | 0.6731 | 0.843 | 1.061 | 0.08557 | 0.4282 | 1.366e-05 | 0.0003774 | 2.207 | 1.142 | 5.715 |  |
| Gm22748 | predicted gene, 22748 | 0.4801 | 0.7483 | 1.163 | 0.2178 | 0.7196 | 0.01833 | 0.1844 | 1.892 | 0.9198 | 2.569 | 0.001115 | 0.0111 | 0.4173 | -1.261 | -3.804 | 0.0191 | 0.08958 | 0.5566 | -0.8453 | -2.55 |  |
| Csrnp1 | cysteine-serine-rich nuclear p | 0.06503 | 0.2712 | 1.377 | 0.4613 | 1.953 | 0.4979 | 0.7854 | 0.8748 | -0.193 | -0.6904 | 0.0005737 | 0.006644 | 2.082 | 1.058 | 4.088 | 0.002278 | 0.01891 | 1.872 | 0.9049 | 3.496 |  |
| Gm24564 | predicted gene, 24564 | 0.006056 | 0.06916 | 0.5048 | -0.9863 | -3.069 | 0.2225 | 0.5838 | 0.7176 | -0.4787 | -1.259 | 0.2191 | 0.4614 | 0.7338 | -0.4466 | -1.269 | 0.006223 | 0.03965 | 0.4743 | -1.076 | -3.057 |  |
| Cpne2 | copine II | 0.01567 | 0.12 | 0.7503 | -0.4144 | -2.641 | 0.5631 | 0.8203 | 0.9271 | -0.1092 | -0.588 | 7.092e-06 | 0.0002266 | 0.4886 | -1.033 | -6.012 | 0.5019 | 0.714 | 1.085 | 0.1175 | 0.6839 |  |
| A430105I19Rik | NA | 0.0721 | 0.2868 | 0.7715 | -0.3742 | -1.899 | 0.4622 | 0.7662 | 1.129 | 0.1748 | 0.7497 | 1.32e-06 | 6.094e-05 | 0.3617 | -1.467 | -6.797 | 0.4148 | 0.6429 | 0.8828 | -0.1798 | -0.8328 |  |
| Rtn4rl1 | reticulon 4 receptor-like 1 | 0.389 | 0.6811 | 0.8494 | -0.2356 | -0.8807 | 0.1344 | 0.476 | 1.408 | 0.4938 | 1.56 | 2.766e-06 | 0.0001075 | 0.27 | -1.889 | -6.447 | 0.3199 | 0.5605 | 0.8129 | -0.2989 | -1.02 |  |
| Sox17 | SRY (sex determining region Y) | 0.01229 | 0.1037 | 1.333 | 0.415 | 2.752 | 0.9125 | 0.9706 | 0.9863 | -0.01985 | -0.1113 | 4.494e-07 | 2.707e-05 | 2.312 | 1.209 | 7.321 | 0.5701 | 0.7636 | 1.068 | 0.09537 | 0.5774 |  |
| Cks1b | CDC28 protein kinase 1b | 0.06287 | 0.2662 | 0.7304 | -0.4533 | -1.97 | 0.2912 | 0.6471 | 0.815 | -0.2952 | -1.084 | 0.0003634 | 0.004679 | 0.4731 | -1.08 | -4.284 | 0.02617 | 0.1128 | 1.521 | 0.6055 | 2.402 |  |
| Igkv1-117 | immunoglobulin kappa variable | 0.002606 | 0.04029 | 10.49 | 3.392 | 3.438 | 0.03396 | 0.2583 | 6.309 | 2.657 | 2.277 | 0.7086 | 0.8634 | 1.328 | 0.4097 | 0.3791 | 0.1127 | 0.2997 | 3.465 | 1.793 | 1.659 |  |
| Mtss1l | NA | 0.01465 | 0.115 | 0.6948 | -0.5253 | -2.672 | 0.8901 | 0.9627 | 1.023 | 0.03255 | 0.14 | 0.0001351 | 0.002168 | 0.4952 | -1.014 | -4.709 | 0.006141 | 0.03929 | 0.633 | -0.6597 | -3.063 |  |
| Angpt2 | angiopoietin 2 | 0.4409 | 0.7214 | 0.9125 | -0.1321 | -0.7863 | 0.1135 | 0.4457 | 1.256 | 0.329 | 1.655 | 6.067e-07 | 3.393e-05 | 0.4005 | -1.32 | -7.173 | 0.7345 | 0.8704 | 0.9571 | -0.0633 | -0.3439 |  |
| Gen1 | GEN1, Holliday junction 5' fla | 0.4382 | 0.7189 | 0.9061 | -0.1422 | -0.791 | 0.1244 | 0.4633 | 1.267 | 0.3413 | 1.604 | 0.5099 | 0.7371 | 0.9125 | -0.1321 | -0.6709 | 1.641e-06 | 7.239e-05 | 0.401 | -1.318 | -6.693 |  |
| Plpp7 | phospholipid phosphatase 7 (in | 0.01537 | 0.1183 | 0.5776 | -0.7918 | -2.65 | 0.3092 | 0.661 | 0.7744 | -0.3689 | -1.043 | 0.5418 | 0.7604 | 1.151 | 0.2032 | 0.6207 | 0.0002208 | 0.003236 | 0.3605 | -1.472 | -4.497 |  |
| Smkr-ps | smal lysine rich protein 1, ps | 0.9941 | 0.9981 | 0.9992 | -0.001193 | -0.007429 | 0.02656 | 0.2252 | 0.7294 | -0.4551 | -2.395 | 3.094e-06 | 0.000117 | 2.181 | 1.125 | 6.395 | 0.1939 | 0.4179 | 1.178 | 0.2365 | 1.344 |  |
| Znrf2 | zinc and ring finger 2 | 0.008163 | 0.08197 | 0.6943 | -0.5264 | -2.937 | 0.6341 | 0.8581 | 0.9314 | -0.1025 | -0.4834 | 3.392e-06 | 0.0001259 | 0.4213 | -1.247 | -6.352 | 0.9022 | 0.9556 | 0.9832 | -0.02444 | -0.1245 |  |
| Fbxo22 | F-box protein 22 | 0.03275 | 0.1854 | 0.7689 | -0.3792 | -2.294 | 0.6691 | 0.8745 | 1.061 | 0.08484 | 0.4338 | 2.201e-06 | 8.958e-05 | 0.4393 | -1.187 | -6.554 | 0.27 | 0.5087 | 0.8673 | -0.2054 | -1.134 |  |
| 1810011H11Rik | NA | 0.1858 | 0.4761 | 1.172 | 0.2286 | 1.37 | 0.2639 | 0.6211 | 0.8544 | -0.2269 | -1.15 | 9.465e-06 | 0.0002805 | 2.106 | 1.075 | 5.881 | 0.1258 | 0.3197 | 1.224 | 0.292 | 1.598 |  |
| Exd2 | exonuclease 3'-5' domain conta | 0.009116 | 0.08753 | 0.7512 | -0.4127 | -2.887 | 0.8992 | 0.9662 | 0.9851 | -0.02171 | -0.1284 | 0.737 | 0.8785 | 0.9637 | -0.05332 | -0.3405 | 6.351e-07 | 3.348e-05 | 0.4602 | -1.12 | -7.151 |  |
| Anxa4 | annexin A4 | 0.4075 | 0.6955 | 0.9083 | -0.1387 | -0.8461 | 0.02622 | 0.2235 | 0.7241 | -0.4657 | -2.401 | 0.8311 | 0.9264 | 0.9735 | -0.03881 | -0.2161 | 1.482e-05 | 0.000402 | 2.028 | 1.02 | 5.679 |  |
| Junb | jun B proto-oncogene | 0.2197 | 0.5193 | 1.236 | 0.3055 | 1.267 | 0.2455 | 0.6055 | 0.7893 | -0.3414 | -1.197 | 0.3215 | 0.5767 | 1.205 | 0.2685 | 1.017 | 3.544e-06 | 0.0001282 | 3.187 | 1.672 | 6.331 |  |
| Ctxn3 | cortexin 3 | 0.05612 | 0.2509 | 1.335 | 0.4172 | 2.028 | 0.504 | 0.7889 | 0.8915 | -0.1657 | -0.6805 | 0.8943 | 0.9556 | 1.021 | 0.03032 | 0.1345 | 2.45e-07 | 1.624e-05 | 3.29 | 1.718 | 7.624 |  |
| Wscd2 | WSC domain containing 2 | 0.009648 | 0.09035 | 0.7147 | -0.4845 | -2.862 | 0.7799 | 0.9206 | 0.9614 | -0.05673 | -0.2832 | 7.82e-06 | 0.0002441 | 0.4643 | -1.107 | -5.967 | 0.3523 | 0.59 | 0.8848 | -0.1766 | -0.9524 |  |
| Gm6485 | predicted gene 6485 | 0.01098 | 0.0977 | 1.325 | 0.4064 | 2.804 | 0.9326 | 0.9781 | 0.9899 | -0.0147 | -0.08571 | 0.8757 | 0.948 | 1.018 | 0.02516 | 0.1585 | 1.742e-07 | 1.248e-05 | 2.359 | 1.238 | 7.797 |  |
| Cdc14a | CDC14 cell division cycle 14A | 0.5375 | 0.7868 | 0.9184 | -0.1228 | -0.6273 | 0.1009 | 0.4235 | 1.318 | 0.3985 | 1.72 | 0.1353 | 0.3438 | 0.7934 | -0.3338 | -1.556 | 1.307e-05 | 0.0003647 | 0.4263 | -1.23 | -5.735 |  |
| Jarid2 | jumonji, AT rich interactive d | 0.0205 | 0.1401 | 0.7957 | -0.3296 | -2.517 | 0.8194 | 0.9379 | 1.025 | 0.03585 | 0.2313 | 0.8399 | 0.9314 | 1.021 | 0.02937 | 0.2047 | 1.583e-07 | 1.177e-05 | 0.4583 | -1.126 | -7.847 |  |
| Acaa2 | acetyl-Coenzyme A acyltransfer | 0.3966 | 0.6876 | 0.913 | -0.1314 | -0.8662 | 0.1375 | 0.4803 | 1.212 | 0.2776 | 1.547 | 3.312e-06 | 0.0001233 | 0.4806 | -1.057 | -6.363 | 0.3187 | 0.5593 | 0.8889 | -0.1699 | -1.023 |  |
| Frmd8 | FERM domain containing 8 | 0.01014 | 0.0934 | 1.385 | 0.4701 | 2.839 | 0.6934 | 0.8854 | 1.056 | 0.07836 | 0.4 | 0.7022 | 0.8593 | 1.05 | 0.07034 | 0.3879 | 5.955e-06 | 0.0001982 | 2.151 | 1.105 | 6.092 |  |
| Tlcd1 | TLC domain containing 1 | 0.01476 | 0.1154 | 0.6615 | -0.5961 | -2.669 | 0.5838 | 0.8307 | 0.903 | -0.1472 | -0.5569 | 6.929e-06 | 0.0002225 | 0.3601 | -1.474 | -6.023 | 0.6101 | 0.7917 | 1.092 | 0.1268 | 0.5181 |  |
| Gm24098 | predicted gene, 24098 | 0.1096 | 0.3591 | 1.191 | 0.2526 | 1.675 | 0.624 | 0.8523 | 0.9403 | -0.08883 | -0.4978 | 0.228 | 0.4727 | 0.8673 | -0.2054 | -1.244 | 3.514e-07 | 2.122e-05 | 2.345 | 1.23 | 7.443 |  |
| Cebpzos | CCAAT/enhancer binding protein | 0.1179 | 0.3739 | 0.66 | -0.5995 | -1.634 | 0.3667 | 0.7044 | 1.32 | 0.4009 | 0.9236 | 0.0002333 | 0.003325 | 0.2876 | -1.798 | -4.474 | 0.007167 | 0.04411 | 0.4342 | -1.204 | -2.995 |  |
| Irx3 | Iroquois related homeobox 3 | 0.1731 | 0.4581 | 1.227 | 0.295 | 1.413 | 0.2957 | 0.6508 | 0.832 | -0.2653 | -1.074 | 4.024e-06 | 0.0001444 | 2.703 | 1.435 | 6.272 | 0.2947 | 0.5355 | 1.186 | 0.2462 | 1.076 |  |
| 5430416O09Rik | NA | 0.008224 | 0.08225 | 1.58 | 0.6595 | 2.933 | 0.5391 | 0.807 | 1.122 | 0.1662 | 0.6249 | 9.211e-05 | 0.001618 | 2.298 | 1.2 | 4.874 | 0.27 | 0.5087 | 1.214 | 0.2794 | 1.134 |  |
| Shisa2 | shisa family member 2 | 0.02541 | 0.1596 | 0.6959 | -0.523 | -2.416 | 0.422 | 0.7418 | 0.8645 | -0.21 | -0.8199 | 0.3338 | 0.5893 | 1.177 | 0.2349 | 0.9904 | 7.749e-05 | 0.00145 | 0.4433 | -1.174 | -4.949 |  |
| Gas2l3 | growth arrest-specific 2 like | 0.6829 | 0.8702 | 1.078 | 0.1084 | 0.4146 | 0.07161 | 0.3666 | 0.665 | -0.5886 | -1.903 | 0.01579 | 0.07941 | 1.688 | 0.7556 | 2.638 | 0.0001503 | 0.002387 | 2.524 | 1.335 | 4.663 |  |
| Phlda1 | pleckstrin homology like domai | 0.1044 | 0.3492 | 0.7276 | -0.4587 | -1.701 | 0.01485 | 0.1638 | 0.5546 | -0.8505 | -2.666 | 0.5632 | 0.7754 | 1.128 | 0.1736 | 0.5879 | 0.002899 | 0.02259 | 2.002 | 1.002 | 3.392 |  |
| Relt | RELT tumor necrosis factor rec | 0.01089 | 0.09724 | 0.6906 | -0.534 | -2.807 | 0.7524 | 0.9092 | 0.9513 | -0.07201 | -0.3199 | 0.6934 | 0.8539 | 0.9439 | -0.08334 | -0.3999 | 4.216e-06 | 0.0001492 | 0.4054 | -1.303 | -6.251 |  |
| Atg10 | autophagy related 10 | 0.04591 | 0.2244 | 0.7115 | -0.4911 | -2.129 | 0.6096 | 0.8449 | 1.103 | 0.1416 | 0.5188 | 0.0003325 | 0.004362 | 0.4691 | -1.092 | -4.322 | 0.004612 | 0.03189 | 0.572 | -0.806 | -3.189 |  |
| Xrcc5 | X-ray repair complementing def | 0.0203 | 0.1392 | 0.6844 | -0.5471 | -2.521 | 0.8611 | 0.9534 | 1.032 | 0.04551 | 0.1773 | 0.04192 | 0.1582 | 0.699 | -0.5167 | -2.174 | 3.432e-05 | 0.000773 | 0.4172 | -1.261 | -5.305 |  |
| Tmem170b | transmembrane protein 170B | 0.6406 | 0.8463 | 1.054 | 0.07528 | 0.4741 | 0.1358 | 0.4781 | 0.8167 | -0.2921 | -1.554 | 5.666e-07 | 3.217e-05 | 2.385 | 1.254 | 7.207 | 0.3936 | 0.6261 | 0.9002 | -0.1517 | -0.8719 |  |
| Acta1 | actin, alpha 1, skeletal muscl | 0.01675 | 0.125 | 2.035 | 1.025 | 2.611 | 0.4424 | 0.7544 | 1.287 | 0.3641 | 0.7837 | 3.487e-05 | 0.0007666 | 4.854 | 2.279 | 5.299 | 0.499 | 0.7114 | 0.8144 | -0.2962 | -0.6886 |  |
| Rny3 | RNA, Y3 small cytoplasmic (ass | 0.3143 | 0.6159 | 0.7963 | -0.3285 | -1.032 | 0.01835 | 0.1845 | 0.5115 | -0.9672 | -2.568 | 0.001311 | 0.01265 | 2.465 | 1.302 | 3.734 | 0.0935 | 0.2659 | 1.531 | 0.614 | 1.761 |  |
| Cyp2r1 | cytochrome P450, family 2, sub | 0.6306 | 0.8412 | 0.8962 | -0.1581 | -0.4884 | 0.02811 | 0.2324 | 0.5333 | -0.9069 | -2.368 | 0.1446 | 0.3582 | 1.452 | 0.5385 | 1.518 | 0.0001054 | 0.001825 | 3.267 | 1.708 | 4.816 |  |
| Lmod2 | leiomodin 2 (cardiac) | 0.01741 | 0.1277 | 2.088 | 1.062 | 2.593 | 0.289 | 0.6443 | 1.442 | 0.5278 | 1.089 | 0.5295 | 0.7522 | 0.8195 | -0.2871 | -0.6399 | 0.0004303 | 0.005392 | 3.705 | 1.889 | 4.211 |  |
| Prss55 | protease, serine 55 | 0.4901 | 0.7544 | 1.166 | 0.2211 | 0.7031 | 0.3459 | 0.6889 | 1.283 | 0.3592 | 0.9654 | 0.001426 | 0.01344 | 2.418 | 1.274 | 3.698 | 8.669e-05 | 0.001578 | 0.3103 | -1.688 | -4.901 |  |
| Lgi3 | leucine-rich repeat LGI family | 0.8191 | 0.9311 | 0.9685 | -0.04623 | -0.2317 | 0.0507 | 0.3113 | 1.405 | 0.4907 | 2.079 | 3.156e-05 | 0.0007147 | 0.4452 | -1.167 | -5.342 | 0.05595 | 0.19 | 0.7354 | -0.4435 | -2.029 |  |
| Ddo | D-aspartate oxidase | 0.5227 | 0.777 | 1.103 | 0.1418 | 0.6507 | 0.123 | 0.4611 | 1.334 | 0.4152 | 1.611 | 0.07013 | 0.2239 | 1.372 | 0.4567 | 1.913 | 3.101e-05 | 0.0007103 | 0.4126 | -1.277 | -5.35 |  |
| Spag5 | sperm associated antigen 5 | 0.3604 | 0.6562 | 0.8682 | -0.204 | -0.9361 | 0.0454 | 0.2969 | 0.6829 | -0.5502 | -2.134 | 0.4968 | 0.7271 | 0.8918 | -0.1652 | -0.6921 | 6.23e-05 | 0.001233 | 2.304 | 1.204 | 5.044 |  |
| Gm13272 | predicted gene 13272 | 0.01719 | 0.1269 | 1.336 | 0.4175 | 2.598 | 0.8727 | 0.9568 | 0.9788 | -0.03086 | -0.1623 | 6.271e-07 | 3.491e-05 | 2.394 | 1.26 | 7.157 | 0.6328 | 0.8062 | 1.061 | 0.08538 | 0.4852 |  |
| Tagap | T cell activation Rho GTPase a | 0.008752 | 0.08564 | 1.919 | 0.9407 | 2.906 | 0.4595 | 0.764 | 1.222 | 0.2889 | 0.7542 | 0.05078 | 0.1803 | 1.667 | 0.7371 | 2.078 | 0.002624 | 0.02102 | 2.327 | 1.218 | 3.435 |  |
| Amotl2 | angiomotin-like 2 | 0.6554 | 0.8542 | 0.9309 | -0.1034 | -0.4531 | 0.0742 | 0.373 | 1.423 | 0.5086 | 1.884 | 5.633e-05 | 0.001098 | 0.4142 | -1.272 | -5.088 | 0.03532 | 0.1386 | 0.6763 | -0.5643 | -2.258 |  |
| Gm2810 | predicted pseudogene 2810 | 0.1134 | 0.3654 | 1.322 | 0.4028 | 1.656 | 0.4053 | 0.7304 | 0.844 | -0.2447 | -0.8501 | 0.005468 | 0.0364 | 1.777 | 0.8298 | 3.114 | 0.0004683 | 0.005729 | 2.162 | 1.112 | 4.175 |  |
| Tnfrsf23 | tumor necrosis factor receptor | 0.4425 | 0.7226 | 0.8725 | -0.1968 | -0.7835 | 0.1336 | 0.4754 | 1.38 | 0.4648 | 1.563 | 0.007515 | 0.04601 | 0.5671 | -0.8184 | -2.974 | 0.0004039 | 0.005138 | 0.4455 | -1.166 | -4.238 |  |
| Stk17b | serine/threonine kinase 17b (a | 0.007428 | 0.07717 | 2.667 | 1.415 | 2.979 | 0.4469 | 0.7571 | 1.353 | 0.4362 | 0.7759 | 0.007111 | 0.04423 | 2.95 | 1.561 | 2.998 | 0.01553 | 0.07713 | 2.597 | 1.377 | 2.645 |  |
| Samd4 | sterile alpha motif domain con | 0.01245 | 0.1047 | 1.401 | 0.4865 | 2.747 | 0.7806 | 0.9207 | 1.042 | 0.05918 | 0.2824 | 0.3485 | 0.6031 | 1.138 | 0.1863 | 0.9601 | 1.591e-05 | 0.0004232 | 2.137 | 1.096 | 5.647 |  |
| Ppm1l | protein phosphatase 1 (formerl | 0.766 | 0.9077 | 0.9609 | -0.05749 | -0.3017 | 0.8322 | 0.9432 | 1.034 | 0.04839 | 0.2146 | 9.845e-05 | 0.001703 | 2.016 | 1.012 | 4.845 | 3.282e-06 | 0.0001208 | 0.398 | -1.329 | -6.367 |  |
| Adprhl1 | ADP-ribosylhydrolase like 1 | 0.05118 | 0.2382 | 0.7974 | -0.3265 | -2.074 | 0.5889 | 0.8335 | 1.073 | 0.1023 | 0.5492 | 0.7663 | 0.8934 | 0.9646 | -0.05196 | -0.3013 | 6.241e-07 | 3.311e-05 | 0.425 | -1.235 | -7.159 |  |
| Slc2a5 | solute carrier family 2 (facil | 0.357 | 0.6537 | 0.8381 | -0.2548 | -0.9429 | 0.1702 | 0.5287 | 1.371 | 0.455 | 1.423 | 3.396e-05 | 0.0007523 | 0.3364 | -1.572 | -5.31 | 0.07434 | 0.2304 | 0.6795 | -0.5574 | -1.883 |  |
| Zfp951 | zinc finger protein 951 | 0.6074 | 0.8293 | 1.084 | 0.1162 | 0.522 | 0.08476 | 0.3955 | 0.7181 | -0.4777 | -1.814 | 0.0001939 | 0.002875 | 2.159 | 1.11 | 4.553 | 0.01225 | 0.06523 | 1.593 | 0.6714 | 2.754 |  |
| Dok3 | docking protein 3 | 0.00344 | 0.04765 | 2.522 | 1.334 | 3.318 | 0.03911 | 0.2767 | 2.072 | 1.051 | 2.208 | 0.8465 | 0.9347 | 1.062 | 0.08643 | 0.1962 | 0.09816 | 0.2744 | 1.699 | 0.7645 | 1.735 |  |
| Adcy4 | adenylate cyclase 4 | 0.02257 | 0.1482 | 1.321 | 0.4018 | 2.472 | 0.8065 | 0.9325 | 0.9675 | -0.04773 | -0.2482 | 3.56e-07 | 2.261e-05 | 2.504 | 1.324 | 7.437 | 0.8929 | 0.9518 | 1.017 | 0.02428 | 0.1363 |  |
| Myl1 | myosin, light polypeptide 1 | 0.02263 | 0.1484 | 0.5954 | -0.748 | -2.47 | 0.8194 | 0.9379 | 1.059 | 0.08287 | 0.2313 | 0.0002053 | 0.003012 | 0.353 | -1.502 | -4.528 | 0.007301 | 0.04469 | 0.5033 | -0.9906 | -2.986 |  |
| Bst1 | bone marrow stromal cell antig | 0.002466 | 0.03913 | 2.926 | 1.549 | 3.462 | 0.01867 | 0.1864 | 2.558 | 1.355 | 2.56 | 0.543 | 0.7613 | 1.234 | 0.3033 | 0.6189 | 0.5779 | 0.7693 | 1.212 | 0.2773 | 0.5658 |  |
| Tmpo | thymopoietin | 0.03486 | 0.192 | 1.349 | 0.4322 | 2.264 | 0.7263 | 0.8982 | 0.9459 | -0.0802 | -0.3551 | 0.05057 | 0.1798 | 1.352 | 0.435 | 2.08 | 3.72e-05 | 0.0008213 | 2.147 | 1.102 | 5.27 |  |
| Phxr1 | NA | 0.02762 | 0.1676 | 1.44 | 0.526 | 2.376 | 0.7693 | 0.9162 | 0.9475 | -0.07786 | -0.2972 | 6.228e-05 | 0.001188 | 2.335 | 1.223 | 5.044 | 0.02488 | 0.1086 | 1.503 | 0.5883 | 2.426 |  |
| Gnmt | glycine N-methyltransferase | 0.01245 | 0.1047 | 1.366 | 0.4496 | 2.747 | 0.9587 | 0.9867 | 1.007 | 0.01017 | 0.0525 | 0.6878 | 0.8508 | 1.052 | 0.07311 | 0.4078 | 9.713e-07 | 4.795e-05 | 2.37 | 1.245 | 6.944 |  |
| Tnfrsf12a | tumor necrosis factor receptor | 0.03064 | 0.1778 | 1.874 | 0.9063 | 2.326 | 0.5025 | 0.7885 | 1.244 | 0.3148 | 0.6829 | 0.3235 | 0.5788 | 0.7412 | -0.432 | -1.012 | 4.671e-05 | 0.0009856 | 4.615 | 2.206 | 5.17 |  |
| Adgre1 | adhesion G protein-coupled rec | 0.6751 | 0.8654 | 0.9215 | -0.118 | -0.4254 | 0.02744 | 0.229 | 0.582 | -0.7808 | -2.379 | 0.003575 | 0.02663 | 2.004 | 1.003 | 3.301 | 0.004079 | 0.02924 | 1.98 | 0.9855 | 3.243 |  |
| Cachd1 | cache domain containing 1 | 0.04258 | 0.2148 | 0.7211 | -0.4717 | -2.166 | 0.6473 | 0.8639 | 1.087 | 0.1197 | 0.4645 | 6.782e-07 | 3.688e-05 | 0.3082 | -1.698 | -7.119 | 0.7534 | 0.8819 | 0.9487 | -0.07597 | -0.3185 |  |
| Mir377 | microRNA 377 | 0.07962 | 0.301 | 0.7373 | -0.4397 | -1.847 | 0.5098 | 0.7925 | 1.14 | 0.1891 | 0.6712 | 0.01198 | 0.06503 | 0.6067 | -0.7209 | -2.764 | 0.0002358 | 0.003411 | 0.4458 | -1.165 | -4.469 |  |
| Fas | Fas (TNF receptor superfamily | 0.04395 | 0.2186 | 1.381 | 0.4659 | 2.15 | 0.6736 | 0.8759 | 0.9268 | -0.1096 | -0.4275 | 0.04152 | 0.1573 | 1.431 | 0.5171 | 2.179 | 5.535e-05 | 0.001118 | 2.313 | 1.21 | 5.096 |  |
| Zbtb10 | zinc finger and BTB domain con | 0.05265 | 0.2422 | 1.269 | 0.3442 | 2.06 | 0.629 | 0.855 | 0.935 | -0.097 | -0.4906 | 0.314 | 0.5698 | 1.14 | 0.1891 | 1.033 | 4.718e-06 | 0.0001627 | 2.196 | 1.135 | 6.199 |  |
| Olfr920 | olfactory receptor 920 | 0.1076 | 0.3554 | 1.312 | 0.3923 | 1.685 | 0.4129 | 0.7353 | 0.8524 | -0.2304 | -0.8363 | 0.0002931 | 0.003945 | 2.167 | 1.116 | 4.376 | 0.007944 | 0.04747 | 1.684 | 0.7521 | 2.949 |  |
| Entpd2 | ectonucleoside triphosphate di | 0.2448 | 0.5478 | 1.211 | 0.276 | 1.198 | 0.241 | 0.6007 | 0.7959 | -0.3293 | -1.208 | 6.844e-05 | 0.001266 | 2.399 | 1.262 | 5.003 | 0.0402 | 0.1516 | 1.468 | 0.5537 | 2.194 |  |
| Tmtc1 | transmembrane and tetratricope | 0.9937 | 0.9979 | 1.001 | 0.001433 | 0.007937 | 0.6593 | 0.8702 | 0.9359 | -0.09562 | -0.4475 | 5.435e-06 | 0.0001858 | 2.319 | 1.213 | 6.134 | 0.0001506 | 0.002389 | 0.5277 | -0.9223 | -4.662 |  |
| Snord116l1 | small nucleolar RNA, C/D box 1 | 0.4998 | 0.7611 | 1.198 | 0.2602 | 0.6873 | 0.1228 | 0.4608 | 0.6063 | -0.7218 | -1.611 | 0.001356 | 0.01298 | 2.913 | 1.542 | 3.72 | 0.002949 | 0.02288 | 2.645 | 1.403 | 3.384 |  |
| Ncapg2 | non-SMC condensin II complex, | 0.1622 | 0.4427 | 1.222 | 0.2889 | 1.451 | 0.3275 | 0.6747 | 0.8489 | -0.2364 | -1.004 | 0.0001133 | 0.001898 | 2.061 | 1.043 | 4.785 | 0.02401 | 0.1057 | 1.446 | 0.5326 | 2.442 |  |
| Lum | lumican | 0.009511 | 0.08965 | 0.504 | -0.9886 | -2.868 | 0.4066 | 0.7308 | 0.7869 | -0.3458 | -0.8478 | 0.08889 | 0.2627 | 0.6262 | -0.6752 | -1.788 | 0.003425 | 0.02568 | 0.4195 | -1.253 | -3.319 |  |
| Mir568 | microRNA 568 | 0.2687 | 0.5743 | 0.8258 | -0.2761 | -1.138 | 0.02426 | 0.2142 | 0.6156 | -0.6999 | -2.438 | 0.4264 | 0.6722 | 1.161 | 0.2158 | 0.8119 | 0.0007544 | 0.00823 | 2.079 | 1.056 | 3.971 |  |
| Glo1 | glyoxalase 1 | 0.284 | 0.5889 | 0.8303 | -0.2684 | -1.101 | 0.02057 | 0.1969 | 0.6048 | -0.7254 | -2.515 | 0.0002405 | 0.00339 | 2.283 | 1.191 | 4.46 | 0.508 | 0.7187 | 1.133 | 0.18 | 0.6741 |  |
| Eif4a2 | eukaryotic translation initiat | 0.433 | 0.7142 | 1.136 | 0.184 | 0.8002 | 0.1439 | 0.4916 | 0.7506 | -0.4139 | -1.521 | 6.463e-05 | 0.001218 | 2.406 | 1.267 | 5.028 | 0.04944 | 0.175 | 1.441 | 0.527 | 2.092 |  |
| Polk | polymerase (DNA directed), kap | 0.01241 | 0.1045 | 0.6082 | -0.7173 | -2.748 | 0.5422 | 0.8092 | 0.8757 | -0.1915 | -0.6201 | 0.2322 | 0.4784 | 0.7833 | -0.3523 | -1.232 | 0.0003623 | 0.004728 | 0.4277 | -1.225 | -4.285 |  |
| Gm21816 | predicted gene, 21816 | 0.02227 | 0.1473 | 0.755 | -0.4055 | -2.478 | 0.8728 | 0.9568 | 1.022 | 0.03141 | 0.1622 | 0.2862 | 0.5409 | 0.8727 | -0.1965 | -1.096 | 4.55e-06 | 0.0001577 | 0.462 | -1.114 | -6.216 |  |
| n-R5s136 | nuclear encoded rRNA 5S 136 | 0.2342 | 0.5358 | 0.6784 | -0.5598 | -1.227 | 0.01847 | 0.1851 | 0.3828 | -1.385 | -2.565 | 0.004731 | 0.03273 | 3.008 | 1.589 | 3.178 | 0.09114 | 0.2617 | 1.85 | 0.8874 | 1.775 |  |
| Gm15772 | predicted gene 15772 | 0.4245 | 0.7088 | 0.8206 | -0.2852 | -0.8152 | 0.02315 | 0.2088 | 0.4937 | -1.018 | -2.46 | 0.002548 | 0.02078 | 2.499 | 1.321 | 3.448 | 0.03303 | 0.1323 | 1.837 | 0.8777 | 2.29 |  |
| B230307C23Rik | RIKEN cDNA B230307C23 gene | 0.7741 | 0.9105 | 1.032 | 0.04529 | 0.2909 | 0.06958 | 0.3621 | 0.7828 | -0.3533 | -1.918 | 0.3923 | 0.6422 | 1.109 | 0.1491 | 0.8745 | 5.412e-06 | 0.0001841 | 2.066 | 1.046 | 6.136 |  |
| Synj2 | synaptojanin 2 | 0.01435 | 0.1134 | 1.557 | 0.639 | 2.682 | 0.9859 | 0.996 | 1.003 | 0.005039 | 0.01787 | 0.01928 | 0.09186 | 1.585 | 0.6644 | 2.545 | 0.0001254 | 0.002086 | 2.358 | 1.237 | 4.741 |  |
| Fam105a | NA | 0.003129 | 0.04534 | 2.041 | 1.029 | 3.359 | 0.02539 | 0.2193 | 1.835 | 0.8761 | 2.416 | 0.7877 | 0.9039 | 1.066 | 0.09164 | 0.2729 | 0.2859 | 0.5263 | 1.291 | 0.3681 | 1.096 |  |
| Gm13150 | NA | 0.6437 | 0.848 | 1.117 | 0.1591 | 0.4696 | 0.08344 | 0.3925 | 0.6027 | -0.7305 | -1.822 | 0.0002403 | 0.00339 | 3.151 | 1.656 | 4.461 | 0.01292 | 0.06772 | 2.018 | 1.013 | 2.73 |  |
| Ephb6 | Eph receptor B6 | 0.1153 | 0.3694 | 0.8434 | -0.2457 | -1.646 | 0.4193 | 0.7401 | 1.106 | 0.1456 | 0.8247 | 1.319e-06 | 6.094e-05 | 0.4629 | -1.111 | -6.797 | 0.7051 | 0.8533 | 0.9574 | -0.06278 | -0.384 |  |
| Cryab | crystallin, alpha B | 0.009901 | 0.09191 | 0.5857 | -0.7718 | -2.85 | 0.3954 | 0.7238 | 0.8246 | -0.2783 | -0.8686 | 6.523e-05 | 0.001225 | 0.3559 | -1.49 | -5.024 | 0.9737 | 0.9882 | 1.007 | 0.009886 | 0.03333 |  |
| Aldh3b3 | aldehyde dehydrogenase 3 famil | 0.0424 | 0.2145 | 0.6916 | -0.532 | -2.168 | 0.6678 | 0.8736 | 1.092 | 0.1265 | 0.4356 | 4.289e-05 | 0.0008918 | 0.379 | -1.4 | -5.208 | 0.04472 | 0.1631 | 0.671 | -0.5757 | -2.142 |  |
| Hspa12b | heat shock protein 12B | 0.5564 | 0.7982 | 1.063 | 0.08777 | 0.5982 | 0.106 | 0.4326 | 0.8157 | -0.2939 | -1.693 | 1.706e-06 | 7.264e-05 | 2.103 | 1.073 | 6.675 | 0.6575 | 0.8223 | 1.051 | 0.07234 | 0.4501 |  |
| Tubb6 | tubulin, beta 6 class V | 0.9125 | 0.9684 | 1.025 | 0.03494 | 0.1113 | 0.1575 | 0.5109 | 0.6852 | -0.5455 | -1.469 | 0.1168 | 0.3127 | 0.6766 | -0.5637 | -1.639 | 1.608e-06 | 7.108e-05 | 4.941 | 2.305 | 6.703 |  |
| Ercc1 | excision repair cross-compleme | 0.346 | 0.6438 | 1.133 | 0.1799 | 0.9651 | 0.1866 | 0.5484 | 0.8113 | -0.3016 | -1.368 | 4.383e-05 | 0.0009071 | 2.087 | 1.061 | 5.198 | 0.07522 | 0.232 | 1.304 | 0.3832 | 1.877 |  |
| Sema6d | sema domain, transmembrane dom | 0.0976 | 0.3373 | 0.8153 | -0.2946 | -1.738 | 0.786 | 0.9232 | 0.9625 | -0.05521 | -0.2752 | 0.04076 | 0.1556 | 1.325 | 0.4062 | 2.188 | 6.448e-06 | 0.0002116 | 0.4587 | -1.124 | -6.055 |  |
| Itgb2 | integrin beta 2 | 0.01061 | 0.09572 | 2.037 | 1.027 | 2.819 | 0.2915 | 0.6473 | 1.382 | 0.4669 | 1.083 | 0.5091 | 0.7366 | 1.204 | 0.2682 | 0.6722 | 0.002071 | 0.01762 | 2.66 | 1.411 | 3.537 |  |
| Fkbpl | FK506 binding protein-like | 0.02966 | 0.1745 | 1.551 | 0.6332 | 2.342 | 0.3008 | 0.6548 | 1.266 | 0.3399 | 1.062 | 0.316 | 0.5717 | 0.8096 | -0.3047 | -1.029 | 0.0007054 | 0.007821 | 2.273 | 1.185 | 4 |  |
| Myl6b | myosin, light polypeptide 6B | 0.01146 | 0.09962 | 0.6775 | -0.5617 | -2.784 | 0.6814 | 0.8803 | 0.9334 | -0.09945 | -0.4166 | 6.749e-05 | 0.001255 | 0.4642 | -1.107 | -5.009 | 0.2203 | 0.4504 | 0.8238 | -0.2797 | -1.265 |  |
| Dmtn | dematin actin binding protein | 0.01204 | 0.1024 | 0.7099 | -0.4942 | -2.762 | 0.7215 | 0.8966 | 0.9483 | -0.07655 | -0.3615 | 1.298e-05 | 0.0003574 | 0.4585 | -1.125 | -5.738 | 0.5208 | 0.7287 | 0.915 | -0.1282 | -0.6537 |  |
| Mctp2 | multiple C2 domains, transmemb | 0.01088 | 0.09716 | 1.94 | 0.9562 | 2.808 | 0.6654 | 0.8726 | 1.13 | 0.1769 | 0.4389 | 0.004587 | 0.03196 | 2.283 | 1.191 | 3.192 | 0.00598 | 0.0385 | 2.215 | 1.147 | 3.075 |  |
| Rps10 | ribosomal protein S10 | 0.9595 | 0.9857 | 1.007 | 0.0106 | 0.05138 | 0.03988 | 0.2793 | 0.6894 | -0.5367 | -2.198 | 0.0001875 | 0.002804 | 2.045 | 1.032 | 4.567 | 0.01389 | 0.07123 | 1.526 | 0.6095 | 2.697 |  |
| Tcaf1 | TRPM8 channel-associated facto | 0.01317 | 0.1085 | 0.7109 | -0.4923 | -2.721 | 0.864 | 0.9541 | 0.9746 | -0.03714 | -0.1735 | 3.262e-05 | 0.0007316 | 0.481 | -1.056 | -5.328 | 0.1341 | 0.3332 | 0.8069 | -0.3095 | -1.562 |  |
| Ccdc134 | coiled-coil domain containing | 0.4034 | 0.6931 | 0.8785 | -0.1869 | -0.8536 | 0.1759 | 0.5358 | 1.287 | 0.3635 | 1.403 | 0.04424 | 0.1644 | 0.6998 | -0.5149 | -2.147 | 0.0001263 | 0.002095 | 0.455 | -1.136 | -4.738 |  |
| Vcam1 | vascular cell adhesion molecul | 0.003096 | 0.04514 | 2.608 | 1.383 | 3.363 | 0.02128 | 0.2005 | 2.323 | 1.216 | 2.499 | 0.3812 | 0.632 | 1.323 | 0.4033 | 0.8955 | 0.8304 | 0.9218 | 1.07 | 0.09771 | 0.217 |  |
| Slc25a53 | solute carrier family 25, memb | 0.003532 | 0.04837 | 2.065 | 1.046 | 3.306 | 0.01752 | 0.1798 | 1.958 | 0.9697 | 2.59 | 0.8067 | 0.9141 | 0.9422 | -0.08594 | -0.2479 | 0.3737 | 0.6081 | 1.244 | 0.3154 | 0.9099 |  |
| Anxa8 | annexin A8 | 0.04968 | 0.2348 | 1.431 | 0.5167 | 2.089 | 0.6431 | 0.8615 | 0.909 | -0.1377 | -0.4705 | 4.503e-05 | 0.0009252 | 2.648 | 1.405 | 5.186 | 0.05262 | 0.1829 | 1.472 | 0.5582 | 2.06 |  |
| Gm23549 | NA | 0.569 | 0.8062 | 1.167 | 0.2224 | 0.5791 | 0.1091 | 0.4389 | 0.5896 | -0.7622 | -1.677 | 0.001446 | 0.01359 | 2.935 | 1.553 | 3.692 | 0.003352 | 0.02529 | 2.64 | 1.401 | 3.329 |  |
| Gpr1 | G protein-coupled receptor 1 | 0.5052 | 0.7644 | 1.101 | 0.1386 | 0.6785 | 0.1258 | 0.4656 | 0.7652 | -0.3862 | -1.598 | 2.736e-05 | 0.0006358 | 2.313 | 1.21 | 5.406 | 0.1189 | 0.3093 | 1.288 | 0.3646 | 1.629 |  |
| Edem1 | ER degradation enhancer, manno | 0.2175 | 0.5166 | 1.208 | 0.2731 | 1.274 | 0.7448 | 0.9064 | 0.9436 | -0.08375 | -0.33 | 0.03452 | 0.1388 | 0.6911 | -0.533 | -2.269 | 1.243e-06 | 5.841e-05 | 3.039 | 1.604 | 6.826 |  |
| Gm10762 | predicted gene 10762 | 0.04983 | 0.235 | 0.7002 | -0.5143 | -2.088 | 0.6659 | 0.873 | 1.093 | 0.1277 | 0.4383 | 0.01607 | 0.08042 | 0.6115 | -0.7095 | -2.63 | 0.0002152 | 0.003167 | 0.4303 | -1.216 | -4.508 |  |
| Ncan | neurocan | 0.06075 | 0.2614 | 0.7832 | -0.3525 | -1.987 | 0.612 | 0.8462 | 1.078 | 0.1081 | 0.5153 | 5.496e-06 | 0.0001868 | 0.4381 | -1.191 | -6.129 | 0.3369 | 0.5756 | 0.8759 | -0.1912 | -0.984 |  |
| Mogat2 | monoacylglycerol O-acyltransfe | 0.01221 | 0.1034 | 0.6414 | -0.6408 | -2.755 | 0.653 | 0.8665 | 0.9166 | -0.1256 | -0.4564 | 2.425e-05 | 0.0005792 | 0.3814 | -1.391 | -5.459 | 0.5503 | 0.749 | 0.8983 | -0.1548 | -0.6075 |  |
| LOC102642734 | NA | 0.1887 | 0.4801 | 0.8762 | -0.1906 | -1.361 | 0.3142 | 0.6644 | 1.126 | 0.1711 | 1.033 | 7.271e-07 | 3.896e-05 | 0.4707 | -1.087 | -7.085 | 0.9909 | 0.9953 | 1.001 | 0.001774 | 0.01156 |  |
| 2310067E19Rik | NA | 0.7126 | 0.8828 | 0.9501 | -0.07382 | -0.3736 | 0.08543 | 0.3962 | 1.341 | 0.4231 | 1.81 | 0.2629 | 0.5151 | 0.8413 | -0.2493 | -1.152 | 1.307e-05 | 0.0003647 | 0.423 | -1.241 | -5.735 |  |
| Gadd45g | growth arrest and DNA-damage-i | 0.003374 | 0.04708 | 2.562 | 1.357 | 3.326 | 0.01963 | 0.1916 | 2.337 | 1.225 | 2.537 | 0.3374 | 0.5929 | 1.356 | 0.4393 | 0.9829 | 0.9255 | 0.9668 | 0.9711 | -0.04235 | -0.09475 |  |
| Mapk8ip1 | mitogen-activated protein kina | 0.01583 | 0.1208 | 0.6975 | -0.5197 | -2.637 | 0.9811 | 0.9945 | 1.004 | 0.005593 | 0.02398 | 8.781e-06 | 0.0002651 | 0.4126 | -1.277 | -5.915 | 0.1818 | 0.4023 | 0.813 | -0.2988 | -1.383 |  |
| Dock8 | dedicator of cytokinesis 8 | 0.01209 | 0.1026 | 1.971 | 0.9792 | 2.76 | 0.4436 | 0.7554 | 1.255 | 0.3281 | 0.7816 | 0.1661 | 0.3899 | 1.473 | 0.5586 | 1.437 | 0.001841 | 0.01614 | 2.629 | 1.395 | 3.588 |  |
| Cdh13 | cadherin 13 | 0.7061 | 0.8797 | 0.9539 | -0.06808 | -0.3825 | 0.3282 | 0.6757 | 0.8639 | -0.211 | -1.002 | 2.635e-05 | 0.0006161 | 2.081 | 1.057 | 5.422 | 0.004276 | 0.03027 | 0.647 | -0.6282 | -3.222 |  |
| Senp8 | SUMO/sentrin specific peptidas | 0.4506 | 0.7276 | 0.8791 | -0.1859 | -0.7695 | 0.1495 | 0.5004 | 1.346 | 0.4285 | 1.499 | 0.005247 | 0.03528 | 0.563 | -0.8289 | -3.133 | 0.001042 | 0.01048 | 0.4951 | -1.014 | -3.833 |  |
| D8Ertd82e | NA | 0.08619 | 0.3152 | 1.294 | 0.3715 | 1.805 | 0.5063 | 0.7903 | 0.892 | -0.1649 | -0.6768 | 2.255e-05 | 0.0005456 | 2.359 | 1.238 | 5.491 | 0.1157 | 0.304 | 1.293 | 0.3709 | 1.645 |  |
| Myh9 | myosin, heavy polypeptide 9, n | 0.1313 | 0.3972 | 1.315 | 0.3948 | 1.574 | 0.4097 | 0.7333 | 0.8409 | -0.25 | -0.8421 | 0.0009466 | 0.009749 | 2.092 | 1.065 | 3.874 | 0.00488 | 0.03326 | 1.827 | 0.8697 | 3.165 |  |
| Akap13 | A kinase (PRKA) anchor protein | 0.01287 | 0.1071 | 1.417 | 0.5027 | 2.732 | 0.6669 | 0.8733 | 1.068 | 0.09513 | 0.4369 | 5.47e-05 | 0.001074 | 2.04 | 1.028 | 5.101 | 0.3466 | 0.5851 | 1.144 | 0.1943 | 0.9639 |  |
| Cd5l | CD5 antigen-like | 0.2097 | 0.5073 | 1.329 | 0.4102 | 1.296 | 0.3044 | 0.6584 | 0.7607 | -0.3947 | -1.054 | 0.002508 | 0.02051 | 2.294 | 1.198 | 3.455 | 0.002205 | 0.01851 | 2.324 | 1.217 | 3.51 |  |
| Idh1 | isocitrate dehydrogenase 1 (NA | 0.09093 | 0.3248 | 0.7647 | -0.3871 | -1.776 | 0.9158 | 0.972 | 1.019 | 0.02761 | 0.1071 | 9.862e-07 | 4.926e-05 | 0.3173 | -1.656 | -6.937 | 0.09987 | 0.2774 | 1.33 | 0.4119 | 1.726 |  |
| Pik3c2b | phosphatidylinositol-4-phospha | 0.01695 | 0.1259 | 0.7458 | -0.423 | -2.605 | 0.9576 | 0.9866 | 0.9929 | -0.01034 | -0.05382 | 0.3608 | 0.6144 | 0.8911 | -0.1664 | -0.9353 | 6.903e-06 | 0.0002214 | 0.4758 | -1.072 | -6.024 |  |
| Tspan17 | tetraspanin 17 | 0.01423 | 0.113 | 0.5875 | -0.7674 | -2.685 | 0.8654 | 0.9546 | 0.9606 | -0.05806 | -0.1717 | 8.35e-05 | 0.001488 | 0.3441 | -1.539 | -4.917 | 0.07463 | 0.2309 | 0.6649 | -0.5888 | -1.881 |  |
| Cyp2b9 | cytochrome P450, family 2, sub | 0.009126 | 0.08754 | 2.089 | 1.063 | 2.887 | 0.2714 | 0.629 | 1.407 | 0.4928 | 1.131 | 0.003376 | 0.02549 | 2.534 | 1.341 | 3.326 | 0.3711 | 0.606 | 1.291 | 0.369 | 0.9149 |  |
| Fcrls | Fc receptor-like S, scavenger | 0.1671 | 0.4495 | 0.7084 | -0.4974 | -1.434 | 0.3556 | 0.6964 | 1.309 | 0.3882 | 0.9457 | 4.047e-05 | 0.000852 | 0.252 | -1.989 | -5.233 | 0.09401 | 0.2668 | 0.6293 | -0.6682 | -1.758 |  |
| Rbp1 | retinol binding protein 1, cel | 0.9327 | 0.9759 | 1.016 | 0.02335 | 0.08557 | 0.04746 | 0.3034 | 0.6233 | -0.6821 | -2.112 | 0.0001488 | 0.00234 | 2.63 | 1.395 | 4.667 | 0.02562 | 0.111 | 1.648 | 0.7211 | 2.412 |  |
| Mir34a | microRNA 34a | 0.02875 | 0.1714 | 1.336 | 0.4177 | 2.357 | 0.931 | 0.9778 | 0.9873 | -0.01839 | -0.08771 | 5.678e-07 | 3.217e-05 | 2.637 | 1.399 | 7.206 | 0.6455 | 0.8146 | 0.9391 | -0.09067 | -0.467 |  |
| Mycbp2 | MYC binding protein 2, E3 ubiq | 0.6425 | 0.8473 | 0.9375 | -0.09309 | -0.4713 | 0.09687 | 0.416 | 1.326 | 0.4071 | 1.742 | 1.7e-05 | 0.0004372 | 0.4307 | -1.215 | -5.617 | 0.2023 | 0.4285 | 0.8206 | -0.2852 | -1.318 |  |
| Cstb | cystatin B | 0.7187 | 0.8861 | 0.9537 | -0.06835 | -0.3654 | 0.04208 | 0.2868 | 0.7166 | -0.4808 | -2.172 | 0.6058 | 0.8019 | 1.077 | 0.1075 | 0.5244 | 2.149e-05 | 0.0005326 | 2.188 | 1.13 | 5.513 |  |
| Gm10759 | NA | 0.01517 | 0.1175 | 0.6707 | -0.5762 | -2.656 | 0.5727 | 0.8247 | 0.903 | -0.1472 | -0.5735 | 0.6322 | 0.8179 | 0.9231 | -0.1155 | -0.4861 | 8.104e-05 | 0.001494 | 0.444 | -1.171 | -4.93 |  |
| Ptbp3 | polypyrimidine tract binding p | 0.003513 | 0.04816 | 2.18 | 1.124 | 3.308 | 0.02323 | 0.2093 | 1.984 | 0.9883 | 2.458 | 0.3633 | 0.6166 | 1.271 | 0.3463 | 0.9303 | 0.8706 | 0.9413 | 1.043 | 0.06142 | 0.165 |  |
| Tnfrsf19 | tumor necrosis factor receptor | 0.01711 | 0.1265 | 1.576 | 0.6565 | 2.601 | 0.8154 | 0.9363 | 1.05 | 0.07064 | 0.2365 | 0.1929 | 0.4267 | 1.295 | 0.3726 | 1.348 | 6.762e-05 | 0.001317 | 2.611 | 1.385 | 5.009 |  |
| Fzd9 | frizzled class receptor 9 | 0.02188 | 0.146 | 0.7425 | -0.4295 | -2.486 | 0.9161 | 0.972 | 1.015 | 0.0218 | 0.1067 | 0.6916 | 0.8529 | 0.9486 | -0.07618 | -0.4025 | 1.451e-06 | 6.568e-05 | 0.4124 | -1.278 | -6.752 |  |
| Phlda3 | pleckstrin homology like domai | 0.3709 | 0.6656 | 0.8689 | -0.2027 | -0.9154 | 0.8305 | 0.9424 | 0.9614 | -0.05684 | -0.2169 | 1.647e-05 | 0.0004278 | 0.3879 | -1.366 | -5.632 | 0.001055 | 0.01057 | 1.903 | 0.9285 | 3.827 |  |
| Slc24a3 | solute carrier family 24 (sodi | 0.2447 | 0.5478 | 0.7978 | -0.326 | -1.199 | 0.2703 | 0.628 | 1.288 | 0.3649 | 1.134 | 0.001056 | 0.01061 | 0.4537 | -1.14 | -3.827 | 0.005286 | 0.03514 | 0.524 | -0.9324 | -3.129 |  |
| Ptgs1 | prostaglandin-endoperoxide syn | 0.2779 | 0.5834 | 1.181 | 0.2405 | 1.115 | 0.264 | 0.6212 | 0.816 | -0.2933 | -1.149 | 0.09261 | 0.2699 | 1.335 | 0.4173 | 1.766 | 6.696e-05 | 0.001307 | 2.272 | 1.184 | 5.013 |  |
| Srd5a3 | steroid 5 alpha-reductase 3 | 0.4315 | 0.7132 | 1.113 | 0.1544 | 0.8028 | 0.03966 | 0.2788 | 1.415 | 0.5007 | 2.201 | 4.189e-05 | 0.0008747 | 0.4669 | -1.099 | -5.218 | 0.779 | 0.8955 | 1.042 | 0.05991 | 0.2845 |  |
| Nudt3 | nudix (nucleotide diphosphate | 0.07192 | 0.2864 | 0.7623 | -0.3916 | -1.9 | 0.6125 | 0.8465 | 1.091 | 0.1255 | 0.5145 | 0.1006 | 0.2846 | 0.7639 | -0.3886 | -1.721 | 4.89e-05 | 0.001021 | 0.4467 | -1.163 | -5.15 |  |
| Cd209a | CD209a antigen | 0.5488 | 0.7935 | 1.113 | 0.1549 | 0.6099 | 0.0328 | 0.2533 | 1.613 | 0.6895 | 2.293 | 0.001414 | 0.01334 | 0.4896 | -1.03 | -3.702 | 0.05267 | 0.183 | 0.6721 | -0.5733 | -2.06 |  |
| Lrrc32 | leucine rich repeat containing | 0.3681 | 0.663 | 1.157 | 0.2101 | 0.9209 | 0.2005 | 0.563 | 0.7806 | -0.3573 | -1.324 | 9.313e-06 | 0.0002771 | 2.773 | 1.471 | 5.888 | 0.3669 | 0.6023 | 1.173 | 0.2307 | 0.9233 |  |
| Nxpe2 | neurexophilin and PC-esterase | 0.1102 | 0.3599 | 1.392 | 0.4774 | 1.671 | 0.4906 | 0.7812 | 0.8483 | -0.2373 | -0.7024 | 0.0165 | 0.0819 | 1.764 | 0.8189 | 2.617 | 0.000406 | 0.00516 | 2.506 | 1.325 | 4.236 |  |
| Tmsb4x | thymosin, beta 4, X chromosome | 0.6311 | 0.8415 | 0.9048 | -0.1443 | -0.4876 | 0.03558 | 0.2655 | 0.5786 | -0.7895 | -2.254 | 0.01345 | 0.0707 | 1.839 | 0.8792 | 2.711 | 0.003947 | 0.02854 | 2.08 | 1.056 | 3.257 |  |
| Pitpnc1 | phosphatidylinositol transfer | 0.2059 | 0.5015 | 0.8506 | -0.2334 | -1.308 | 0.5002 | 0.7867 | 1.106 | 0.145 | 0.6867 | 0.3769 | 0.6283 | 1.13 | 0.1767 | 0.9037 | 1.584e-06 | 7.037e-05 | 0.4028 | -1.312 | -6.71 |  |
| 9130230L23Rik | RIKEN cDNA 9130230L23 gene | 0.998 | 0.9992 | 1 | 0.0005333 | 0.002552 | 0.04287 | 0.289 | 0.6903 | -0.5348 | -2.163 | 4.098e-05 | 0.0008591 | 2.292 | 1.197 | 5.228 | 0.08903 | 0.2581 | 1.328 | 0.4092 | 1.788 |  |
| Ifi47 | interferon gamma inducible pro | 0.7538 | 0.9019 | 1.08 | 0.1106 | 0.318 | 0.08235 | 0.3916 | 0.5935 | -0.7527 | -1.829 | 0.01619 | 0.08087 | 2.001 | 1.001 | 2.626 | 0.000472 | 0.005754 | 3.009 | 1.589 | 4.172 |  |
| Armcx4 | armadillo repeat containing, X | 0.8509 | 0.9441 | 0.9749 | -0.0367 | -0.1905 | 0.2562 | 0.616 | 1.203 | 0.2665 | 1.169 | 0.05081 | 0.1803 | 1.355 | 0.4386 | 2.078 | 3.142e-06 | 0.0001169 | 0.3928 | -1.348 | -6.387 |  |
| Rab4a | RAB4A, member RAS oncogene fam | 0.386 | 0.6789 | 0.8761 | -0.1908 | -0.8863 | 0.2112 | 0.5729 | 1.256 | 0.329 | 1.292 | 0.1627 | 0.3846 | 0.7891 | -0.3418 | -1.449 | 4.742e-05 | 0.0009966 | 0.43 | -1.218 | -5.164 |  |
| Rab3gap1 | RAB3 GTPase activating protein | 0.05884 | 0.2574 | 1.293 | 0.3708 | 2.004 | 0.6535 | 0.8669 | 0.9332 | -0.09976 | -0.4557 | 8.956e-06 | 0.0002689 | 2.293 | 1.197 | 5.906 | 0.3025 | 0.5439 | 1.16 | 0.2145 | 1.058 |  |
| Lypd6 | LY6/PLAUR domain containing 6 | 0.02693 | 0.165 | 0.7434 | -0.4278 | -2.388 | 0.8932 | 0.964 | 1.02 | 0.02883 | 0.136 | 0.07835 | 0.2413 | 0.7769 | -0.3641 | -1.855 | 5.177e-05 | 0.001064 | 0.498 | -1.006 | -5.125 |  |
| Rab15 | RAB15, member RAS oncogene fam | 0.01644 | 0.1236 | 1.565 | 0.6461 | 2.619 | 0.5783 | 0.828 | 1.121 | 0.165 | 0.5651 | 0.2791 | 0.533 | 1.232 | 0.3006 | 1.112 | 0.0004127 | 0.005224 | 2.208 | 1.143 | 4.229 |  |
| Sox9 | SRY (sex determining region Y) | 0.8674 | 0.9508 | 1.031 | 0.04446 | 0.1691 | 0.04249 | 0.288 | 1.596 | 0.6743 | 2.167 | 2.324e-06 | 9.31e-05 | 0.2716 | -1.881 | -6.529 | 0.9759 | 0.989 | 0.9939 | -0.008816 | -0.03061 |  |
| Fcna | ficolin A | 0.3092 | 0.6119 | 1.19 | 0.2508 | 1.043 | 0.2361 | 0.596 | 0.786 | -0.3474 | -1.222 | 0.0001234 | 0.002025 | 2.379 | 1.25 | 4.748 | 0.05182 | 0.1809 | 1.459 | 0.5445 | 2.068 |  |
| Hspb1 | heat shock protein 1 | 0.0169 | 0.1257 | 1.78 | 0.8318 | 2.606 | 0.6267 | 0.8538 | 1.138 | 0.1865 | 0.4939 | 0.2032 | 0.4406 | 1.376 | 0.46 | 1.316 | 0.00042 | 0.005291 | 2.781 | 1.476 | 4.222 |  |
| Gm19924 | NA | 0.2657 | 0.5716 | 0.7585 | -0.3987 | -1.145 | 0.1234 | 0.4617 | 0.6317 | -0.6627 | -1.609 | 0.0007821 | 0.008418 | 2.846 | 1.509 | 3.956 | 0.07486 | 0.2314 | 0.6084 | -0.7169 | -1.879 |  |
| Pfkl | phosphofructokinase, liver, B- | 0.0179 | 0.1298 | 0.7214 | -0.4711 | -2.58 | 0.9879 | 0.9965 | 1.002 | 0.003323 | 0.01538 | 4.019e-05 | 0.000848 | 0.4838 | -1.047 | -5.236 | 0.07491 | 0.2316 | 0.7706 | -0.3759 | -1.879 |  |
| Pdp1 | pyruvate dehyrogenase phosphat | 0.9435 | 0.9802 | 0.9884 | -0.01684 | -0.07172 | 0.1618 | 0.5172 | 1.323 | 0.4037 | 1.453 | 0.1567 | 0.3758 | 1.3 | 0.3786 | 1.471 | 3.223e-06 | 0.0001196 | 0.3208 | -1.64 | -6.375 |  |
| Rgcc | regulator of cell cycle | 0.4642 | 0.7374 | 1.117 | 0.1593 | 0.7462 | 0.1778 | 0.5381 | 0.7831 | -0.3528 | -1.397 | 0.03633 | 0.144 | 1.439 | 0.5247 | 2.244 | 0.0003292 | 0.004412 | 2.016 | 1.012 | 4.326 |  |
| Asph | aspartate-beta-hydroxylase | 0.3012 | 0.6046 | 1.111 | 0.1521 | 1.061 | 0.2492 | 0.6089 | 0.8698 | -0.2012 | -1.187 | 3.078e-06 | 0.0001167 | 2.006 | 1.004 | 6.397 | 0.7264 | 0.8667 | 1.039 | 0.0557 | 0.3548 |  |
| Lctl | lactase-like | 0.01364 | 0.1107 | 0.6039 | -0.7276 | -2.705 | 0.5021 | 0.7883 | 0.86 | -0.2176 | -0.6836 | 0.0001865 | 0.002792 | 0.3932 | -1.347 | -4.57 | 0.5403 | 0.7422 | 0.8805 | -0.1836 | -0.623 |  |
| Rpsa-ps2 | ribosomal protein SA, pseudoge | 0.3148 | 0.6164 | 0.7774 | -0.3633 | -1.031 | 0.0261 | 0.2228 | 0.4994 | -1.002 | -2.403 | 0.007024 | 0.04388 | 2.234 | 1.159 | 3.004 | 0.06428 | 0.209 | 1.689 | 0.7559 | 1.959 |  |
| Ttc30b | tetratricopeptide repeat domai | 0.267 | 0.5727 | 0.8366 | -0.2573 | -1.142 | 0.2636 | 0.621 | 1.237 | 0.3067 | 1.15 | 0.0003204 | 0.004253 | 0.4761 | -1.071 | -4.337 | 0.0205 | 0.09429 | 0.6501 | -0.6213 | -2.517 |  |
| Ttc23 | tetratricopeptide repeat domai | 0.09347 | 0.3298 | 0.8036 | -0.3154 | -1.761 | 0.5576 | 0.8176 | 1.092 | 0.1264 | 0.5963 | 0.2452 | 0.4942 | 0.8497 | -0.2349 | -1.197 | 1.938e-05 | 0.0004924 | 0.4696 | -1.09 | -5.559 |  |
| Nfil3 | nuclear factor, interleukin 3, | 0.0187 | 0.133 | 1.644 | 0.7171 | 2.559 | 0.9331 | 0.9783 | 1.02 | 0.02818 | 0.085 | 0.05512 | 0.1904 | 1.542 | 0.6252 | 2.037 | 0.0001388 | 0.002258 | 2.716 | 1.442 | 4.697 |  |
| mt-Tk | mitochondrially encoded tRNA l | 0.02513 | 0.1586 | 0.5681 | -0.8158 | -2.421 | 0.3338 | 0.6798 | 0.7606 | -0.3949 | -0.9904 | 0.0004437 | 0.005463 | 0.3416 | -1.55 | -4.198 | 0.4461 | 0.6699 | 1.22 | 0.2869 | 0.7773 |  |
| Bcar3 | breast cancer anti-estrogen re | 0.04012 | 0.2076 | 0.6734 | -0.5705 | -2.195 | 0.3355 | 0.6814 | 0.8103 | -0.3034 | -0.9868 | 0.0005972 | 0.006849 | 0.4479 | -1.159 | -4.071 | 0.2235 | 0.4541 | 1.281 | 0.3577 | 1.256 |  |
| Gm13623 | predicted gene 13623 | 0.406 | 0.6944 | 0.8363 | -0.2579 | -0.8488 | 0.0303 | 0.2417 | 0.5593 | -0.8383 | -2.332 | 0.005107 | 0.0346 | 2.066 | 1.047 | 3.145 | 0.04997 | 0.1761 | 1.618 | 0.6945 | 2.086 |  |
| Gm5265 | predicted pseudogene 5265 | 0.4456 | 0.7245 | 1.216 | 0.2818 | 0.7782 | 0.03804 | 0.2732 | 1.934 | 0.9517 | 2.221 | 0.2663 | 0.5191 | 0.7302 | -0.4536 | -1.144 | 0.0008429 | 0.00893 | 0.3401 | -1.556 | -3.923 |  |
| Camk1d | calcium/calmodulin-dependent p | 0.1197 | 0.3774 | 1.3 | 0.3785 | 1.625 | 0.4867 | 0.7789 | 0.8734 | -0.1952 | -0.7087 | 0.01237 | 0.06647 | 1.626 | 0.7013 | 2.749 | 0.0007074 | 0.007834 | 2.028 | 1.02 | 3.998 |  |
| Fam110b | family with sequence similarit | 0.07729 | 0.298 | 0.7946 | -0.3317 | -1.863 | 0.626 | 0.8535 | 1.075 | 0.1043 | 0.495 | 0.4131 | 0.6611 | 0.8931 | -0.163 | -0.8358 | 9.712e-06 | 0.0002868 | 0.4523 | -1.145 | -5.869 |  |
| Gm5763 | predicted pseudogene 5763 | 0.4275 | 0.711 | 1.124 | 0.1685 | 0.81 | 0.1848 | 0.5461 | 0.7911 | -0.3381 | -1.374 | 5.956e-05 | 0.001148 | 2.226 | 1.154 | 5.064 | 0.1242 | 0.3172 | 1.289 | 0.3658 | 1.605 |  |
| 1700040L02Rik | NA | 0.3757 | 0.6699 | 1.204 | 0.2675 | 0.906 | 0.02971 | 0.2395 | 1.763 | 0.8179 | 2.341 | 0.005601 | 0.03708 | 0.4987 | -1.004 | -3.104 | 0.05918 | 0.1977 | 0.6385 | -0.6472 | -2.001 |  |
| Tmem160 | transmembrane protein 160 | 0.0218 | 0.1457 | 0.6994 | -0.5159 | -2.488 | 0.9539 | 0.9858 | 1.01 | 0.01436 | 0.05853 | 4.366e-05 | 0.0009041 | 0.441 | -1.181 | -5.2 | 0.0865 | 0.2538 | 0.7529 | -0.4096 | -1.803 |  |
| Arhgap24 | Rho GTPase activating protein | 0.06487 | 0.2709 | 0.7631 | -0.39 | -1.954 | 0.7967 | 0.9282 | 1.044 | 0.06165 | 0.2611 | 1.36e-06 | 6.181e-05 | 0.3577 | -1.483 | -6.783 | 0.5645 | 0.7594 | 1.093 | 0.1281 | 0.5859 |  |
| St6gal1 | beta galactoside alpha 2,6 sia | 0.01799 | 0.1302 | 1.682 | 0.7502 | 2.578 | 0.7671 | 0.9156 | 1.074 | 0.1034 | 0.3003 | 0.02008 | 0.09458 | 1.748 | 0.8054 | 2.526 | 0.001967 | 0.01696 | 2.196 | 1.135 | 3.56 |  |
| Parvb | parvin, beta | 0.7751 | 0.911 | 1.039 | 0.05573 | 0.2897 | 0.6783 | 0.8788 | 0.9357 | -0.09582 | -0.4209 | 9.541e-06 | 0.0002821 | 2.36 | 1.239 | 5.877 | 0.001355 | 0.01275 | 0.5807 | -0.784 | -3.72 |  |
| Reep4 | receptor accessory protein 4 | 0.02685 | 0.1648 | 1.302 | 0.3805 | 2.389 | 0.9181 | 0.973 | 0.9865 | -0.01961 | -0.1041 | 1.054e-05 | 0.0003062 | 2.024 | 1.017 | 5.832 | 0.3068 | 0.5479 | 1.135 | 0.1829 | 1.049 |  |
| Snord82 | small nucleolar RNA, C/D box 8 | 0.3138 | 0.6156 | 0.7935 | -0.3337 | -1.033 | 0.0293 | 0.2378 | 0.537 | -0.8971 | -2.348 | 0.007332 | 0.04517 | 2.079 | 1.056 | 2.985 | 0.08806 | 0.2564 | 1.552 | 0.6344 | 1.793 |  |
| LOC102634333 | NA | 0.0139 | 0.1117 | 0.5409 | -0.8867 | -2.696 | 0.245 | 0.6051 | 0.7239 | -0.4661 | -1.198 | 0.9654 | 0.9868 | 0.9891 | -0.0158 | -0.04387 | 0.002487 | 0.02019 | 0.4216 | -1.246 | -3.458 |  |
| Kcna5 | potassium voltage-gated channe | 0.1598 | 0.4391 | 1.214 | 0.2797 | 1.46 | 0.4381 | 0.7513 | 0.8831 | -0.1794 | -0.7912 | 0.08835 | 0.2617 | 1.298 | 0.376 | 1.792 | 0.000117 | 0.001975 | 2.002 | 1.001 | 4.771 |  |
| Rasl12 | RAS-like, family 12 | 0.01598 | 0.1213 | 0.6637 | -0.5914 | -2.632 | 0.5485 | 0.8132 | 0.8936 | -0.1623 | -0.6104 | 0.0001325 | 0.002139 | 0.4472 | -1.161 | -4.717 | 0.6131 | 0.7937 | 0.9161 | -0.1264 | -0.5137 |  |
| Drd1 | dopamine receptor D1 | 0.06146 | 0.2631 | 1.323 | 0.4043 | 1.982 | 0.9848 | 0.9957 | 1.003 | 0.004652 | 0.01927 | 2.052e-06 | 8.447e-05 | 2.775 | 1.472 | 6.587 | 0.2356 | 0.4685 | 0.8274 | -0.2733 | -1.223 |  |
| Rpl12 | ribosomal protein L12 | 0.2425 | 0.5456 | 1.213 | 0.2784 | 1.204 | 0.3395 | 0.6839 | 0.8307 | -0.2676 | -0.9786 | 0.04594 | 0.1685 | 1.453 | 0.5389 | 2.128 | 0.0003179 | 0.004297 | 2.142 | 1.099 | 4.341 |  |
| Plek | pleckstrin | 0.01444 | 0.1138 | 1.9 | 0.9257 | 2.679 | 0.4103 | 0.7339 | 1.269 | 0.3438 | 0.8409 | 0.108 | 0.2978 | 1.555 | 0.637 | 1.683 | 0.008312 | 0.04912 | 2.156 | 1.109 | 2.929 |  |
| Gm40525 | NA | 0.908 | 0.9661 | 0.9751 | -0.03632 | -0.1171 | 0.05315 | 0.3182 | 0.5927 | -0.7545 | -2.055 | 0.02597 | 0.1138 | 1.762 | 0.8175 | 2.405 | 0.0008219 | 0.008801 | 2.526 | 1.337 | 3.934 |  |
| Gm41154 | NA | 0.908 | 0.9661 | 0.9751 | -0.03632 | -0.1171 | 0.05315 | 0.3182 | 0.5927 | -0.7545 | -2.055 | 0.02597 | 0.1138 | 1.762 | 0.8175 | 2.405 | 0.0008219 | 0.008801 | 2.526 | 1.337 | 3.934 |  |
| Gm40498 | NA | 0.908 | 0.9661 | 0.9751 | -0.03632 | -0.1171 | 0.05315 | 0.3182 | 0.5927 | -0.7545 | -2.055 | 0.02597 | 0.1138 | 1.762 | 0.8175 | 2.405 | 0.0008219 | 0.008801 | 2.526 | 1.337 | 3.934 |  |
| Axin2 | axin 2 | 0.044 | 0.2188 | 0.7222 | -0.4696 | -2.15 | 0.5693 | 0.8229 | 0.9015 | -0.1496 | -0.5787 | 6.254e-05 | 0.001191 | 0.4333 | -1.207 | -5.043 | 0.3152 | 0.556 | 1.186 | 0.2465 | 1.03 |  |
| Wbscr17 | NA | 0.1139 | 0.3662 | 0.8096 | -0.3047 | -1.653 | 0.5499 | 0.8139 | 1.096 | 0.1326 | 0.6083 | 0.3451 | 0.5997 | 0.8734 | -0.1952 | -0.967 | 2.058e-05 | 0.0005176 | 0.4611 | -1.117 | -5.532 |  |
| Mettl21c | methyltransferase like 21C | 0.038 | 0.2017 | 0.6484 | -0.6251 | -2.222 | 0.3582 | 0.6978 | 0.8049 | -0.3131 | -0.9406 | 0.4108 | 0.659 | 1.197 | 0.2589 | 0.8401 | 0.0009387 | 0.009698 | 0.4368 | -1.195 | -3.877 |  |
| Adamts5 | a disintegrin-like and metallo | 0.01475 | 0.1154 | 0.5462 | -0.8726 | -2.669 | 0.3573 | 0.6972 | 0.7767 | -0.3645 | -0.9423 | 0.0004604 | 0.005626 | 0.3541 | -1.498 | -4.182 | 0.9418 | 0.9733 | 0.9818 | -0.02649 | -0.07396 |  |
| Zc2hc1c | zinc finger, C2HC-type contain | 0.06371 | 0.2684 | 1.303 | 0.3822 | 1.963 | 0.8431 | 0.9475 | 1.033 | 0.04619 | 0.2005 | 0.2617 | 0.514 | 0.843 | -0.2463 | -1.155 | 9.45e-06 | 0.000281 | 2.386 | 1.254 | 5.881 |  |
| Mnda | NA | 0.3533 | 0.65 | 1.263 | 0.3365 | 0.9504 | 0.2336 | 0.595 | 0.7 | -0.5146 | -1.228 | 0.005119 | 0.03465 | 2.328 | 1.219 | 3.144 | 0.002599 | 0.02087 | 2.521 | 1.334 | 3.439 |  |
| Glipr1 | GLI pathogenesis-related 1 (gl | 0.01587 | 0.1209 | 2.095 | 1.067 | 2.635 | 0.3745 | 0.709 | 1.352 | 0.4353 | 0.9084 | 0.2525 | 0.5024 | 1.437 | 0.5227 | 1.178 | 0.005706 | 0.03731 | 2.591 | 1.373 | 3.096 |  |
| Caskin2 | CASK-interacting protein 2 | 0.386 | 0.6789 | 1.097 | 0.1341 | 0.8863 | 0.2275 | 0.5885 | 0.8568 | -0.2229 | -1.245 | 2.524e-06 | 0.0001002 | 2.108 | 1.076 | 6.49 | 0.9779 | 0.9899 | 0.9968 | -0.00465 | -0.02805 |  |
| Ms4a7 | membrane-spanning 4-domains, s | 0.03364 | 0.1881 | 1.466 | 0.5516 | 2.281 | 0.8496 | 0.9501 | 0.9626 | -0.05495 | -0.1921 | 0.0003278 | 0.004318 | 2.213 | 1.146 | 4.328 | 0.01941 | 0.09051 | 1.595 | 0.6733 | 2.542 |  |
| Hes1 | hes family bHLH transcription | 0.01838 | 0.1317 | 0.5644 | -0.8252 | -2.568 | 0.5151 | 0.7951 | 0.8397 | -0.252 | -0.6627 | 0.2219 | 0.4652 | 0.7351 | -0.4439 | -1.261 | 0.001696 | 0.01513 | 0.413 | -1.276 | -3.623 |  |
| Pkn3 | protein kinase N3 | 0.1511 | 0.4271 | 1.188 | 0.2486 | 1.493 | 0.4457 | 0.7563 | 0.8992 | -0.1533 | -0.778 | 1.412e-05 | 0.0003814 | 2.056 | 1.04 | 5.701 | 0.3745 | 0.6088 | 1.122 | 0.1657 | 0.9085 |  |
| 9530091C08Rik | NA | 0.03557 | 0.1941 | 0.6916 | -0.5321 | -2.254 | 0.8621 | 0.9537 | 1.035 | 0.04915 | 0.176 | 0.9526 | 0.9814 | 0.9893 | -0.01557 | -0.06023 | 1.675e-06 | 7.353e-05 | 0.3018 | -1.728 | -6.683 |  |
| Uba7 | ubiquitin-like modifier activa | 0.7762 | 0.9118 | 1.068 | 0.09543 | 0.2882 | 0.08747 | 0.4001 | 0.6138 | -0.7041 | -1.797 | 0.006838 | 0.04304 | 2.135 | 1.094 | 3.016 | 0.001806 | 0.01592 | 2.47 | 1.305 | 3.596 |  |
| Stxbp6 | syntaxin binding protein 6 (am | 0.05291 | 0.243 | 0.7389 | -0.4365 | -2.058 | 0.7886 | 0.9244 | 0.9538 | -0.0682 | -0.2717 | 1.231e-05 | 0.000346 | 0.3953 | -1.339 | -5.762 | 0.3258 | 0.5659 | 1.176 | 0.2341 | 1.007 |  |
| Inhbb | inhibin beta-B | 0.405 | 0.6941 | 0.8473 | -0.239 | -0.8507 | 0.2098 | 0.5723 | 1.348 | 0.4308 | 1.296 | 0.0007999 | 0.008544 | 0.4309 | -1.214 | -3.946 | 0.01778 | 0.08485 | 0.5764 | -0.7949 | -2.583 |  |
| Snord55 | small nucleolar RNA, C/D box 5 | 0.03168 | 0.1816 | 1.545 | 0.6276 | 2.31 | 0.8801 | 0.9595 | 0.9665 | -0.0491 | -0.1527 | 0.005729 | 0.03774 | 1.893 | 0.9207 | 3.094 | 0.00133 | 0.01255 | 2.158 | 1.109 | 3.728 |  |
| Aoc3 | amine oxidase, copper containi | 0.6926 | 0.8738 | 1.126 | 0.1708 | 0.4011 | 0.05084 | 0.3118 | 2.066 | 1.047 | 2.078 | 0.05707 | 0.1951 | 0.5205 | -0.9421 | -2.019 | 0.001731 | 0.01539 | 0.3107 | -1.686 | -3.615 |  |
| Scimp | SLP adaptor and CSK interactin | 0.0845 | 0.3112 | 1.371 | 0.4553 | 1.815 | 0.6339 | 0.8581 | 0.9053 | -0.1435 | -0.4836 | 0.0589 | 0.1994 | 1.464 | 0.5503 | 2.003 | 0.0002019 | 0.003007 | 2.372 | 1.246 | 4.536 |  |
| Bcat1 | branched chain aminotransferas | 0.2856 | 0.5902 | 0.8722 | -0.1973 | -1.097 | 0.299 | 0.6538 | 1.17 | 0.2269 | 1.066 | 7.34e-06 | 0.0002325 | 0.4409 | -1.181 | -5.996 | 0.648 | 0.816 | 0.9387 | -0.09132 | -0.4635 |  |
| Gm26226 | predicted gene, 26226 | 0.1313 | 0.3972 | 0.8357 | -0.2589 | -1.574 | 0.899 | 0.9661 | 1.018 | 0.02503 | 0.1286 | 0.1221 | 0.3214 | 1.224 | 0.2911 | 1.615 | 3.473e-06 | 0.0001263 | 0.4528 | -1.143 | -6.341 |  |
| Pex16 | peroxisomal biogenesis factor | 0.8641 | 0.9498 | 1.02 | 0.02916 | 0.1734 | 0.05955 | 0.3375 | 1.317 | 0.3977 | 1.998 | 6.364e-06 | 0.0002088 | 0.461 | -1.117 | -6.061 | 0.9506 | 0.9776 | 1.008 | 0.01156 | 0.06275 |  |
| Hsp25-ps1 | heat shock protein 25, pseudog | 0.02103 | 0.142 | 1.685 | 0.7523 | 2.505 | 0.5322 | 0.8035 | 1.17 | 0.2259 | 0.6358 | 0.4437 | 0.6862 | 1.195 | 0.2571 | 0.7815 | 0.0006759 | 0.007579 | 2.5 | 1.322 | 4.018 |  |
| Ctss | cathepsin S | 0.02111 | 0.1423 | 1.794 | 0.843 | 2.503 | 0.7506 | 0.9084 | 1.093 | 0.1284 | 0.3223 | 0.0502 | 0.1789 | 1.704 | 0.769 | 2.084 | 0.001336 | 0.0126 | 2.593 | 1.375 | 3.726 |  |
| Tnfrsf14 | tumor necrosis factor receptor | 0.3274 | 0.6272 | 1.197 | 0.2597 | 1.004 | 0.2619 | 0.6201 | 0.7828 | -0.3533 | -1.154 | 0.009668 | 0.05534 | 1.754 | 0.8106 | 2.861 | 0.001672 | 0.01496 | 2.04 | 1.028 | 3.63 |  |
| Atg13 | autophagy related 13 | 0.08693 | 0.3168 | 1.224 | 0.2917 | 1.8 | 0.628 | 0.8544 | 0.9367 | -0.09435 | -0.4921 | 0.8139 | 0.9179 | 1.03 | 0.04234 | 0.2385 | 3.904e-06 | 0.0001401 | 2.167 | 1.116 | 6.286 |  |
| Gm25615 | predicted gene, 25615 | 0.8056 | 0.9247 | 1.065 | 0.09138 | 0.2494 | 0.08642 | 0.3972 | 0.5817 | -0.7817 | -1.803 | 0.003323 | 0.02522 | 2.527 | 1.337 | 3.333 | 0.004559 | 0.03161 | 2.432 | 1.282 | 3.194 |  |
| Hcls1 | hematopoietic cell specific Ly | 0.01677 | 0.125 | 1.988 | 0.9916 | 2.61 | 0.4447 | 0.7559 | 1.275 | 0.3505 | 0.7797 | 0.1109 | 0.3027 | 1.618 | 0.6943 | 1.668 | 0.007863 | 0.04712 | 2.344 | 1.229 | 2.953 |  |
| Rpl36a-ps2 | ribosomal protein L36A, pseudo | 0.9688 | 0.9893 | 0.9947 | -0.007733 | -0.03964 | 0.05652 | 0.3284 | 0.7233 | -0.4673 | -2.024 | 0.000111 | 0.00187 | 2.034 | 1.024 | 4.794 | 0.1131 | 0.3001 | 1.278 | 0.3542 | 1.657 |  |
| Cd164l2 | CD164 sialomucin-like 2 | 0.8856 | 0.9571 | 1.016 | 0.02271 | 0.1457 | 0.1717 | 0.5299 | 0.8343 | -0.2614 | -1.418 | 6.153e-06 | 0.000204 | 2.052 | 1.037 | 6.077 | 0.2915 | 0.5326 | 0.8797 | -0.185 | -1.084 |  |
| Frmd8os | FERM domain containing 8, oppo | 0.6342 | 0.8431 | 0.9199 | -0.1204 | -0.4832 | 0.1359 | 0.4782 | 1.374 | 0.4583 | 1.554 | 0.01815 | 0.08799 | 0.6145 | -0.7026 | -2.574 | 0.001163 | 0.01139 | 0.4885 | -1.034 | -3.786 |  |
| Dok6 | docking protein 6 | 0.1243 | 0.3849 | 1.252 | 0.3248 | 1.605 | 0.528 | 0.8023 | 0.8989 | -0.1538 | -0.6423 | 5.552e-06 | 0.0001874 | 2.563 | 1.358 | 6.124 | 0.7288 | 0.8678 | 1.056 | 0.07797 | 0.3516 |  |
| Dennd3 | DENN/MADD domain containing 3 | 0.039 | 0.2042 | 1.49 | 0.5749 | 2.209 | 0.8362 | 0.945 | 0.9563 | -0.06449 | -0.2095 | 0.0006799 | 0.007558 | 2.211 | 1.145 | 4.015 | 0.01339 | 0.06939 | 1.709 | 0.7735 | 2.713 |  |
| Stac3 | SH3 and cysteine rich domain 3 | 0.02607 | 0.1619 | 0.742 | -0.4306 | -2.403 | 0.8666 | 0.9551 | 0.9753 | -0.03607 | -0.1701 | 0.554 | 0.7693 | 0.9214 | -0.1181 | -0.602 | 2.596e-05 | 0.0006161 | 0.4778 | -1.065 | -5.429 |  |
| Atg101 | autophagy related 101 | 0.7192 | 0.8862 | 0.9451 | -0.08154 | -0.3646 | 0.05203 | 0.3156 | 0.6846 | -0.5467 | -2.066 | 0.0001101 | 0.001861 | 2.258 | 1.175 | 4.797 | 0.3791 | 0.6132 | 1.165 | 0.2204 | 0.8996 |  |
| Tmeff1 | transmembrane protein with EGF | 0.02118 | 0.1426 | 1.55 | 0.6319 | 2.501 | 0.8691 | 0.9558 | 1.035 | 0.0499 | 0.1669 | 0.001372 | 0.01307 | 2.039 | 1.028 | 3.715 | 0.01733 | 0.0833 | 1.645 | 0.7181 | 2.595 |  |
| Pcna-ps2 | proliferating cell nuclear ant | 0.8784 | 0.9551 | 1.024 | 0.0347 | 0.1549 | 0.08723 | 0.3992 | 0.7187 | -0.4766 | -1.798 | 0.05966 | 0.2012 | 1.404 | 0.4899 | 1.997 | 0.0003882 | 0.004988 | 2.062 | 1.044 | 4.255 |  |
| Id1 | inhibitor of DNA binding 1, HL | 0.7619 | 0.9058 | 1.04 | 0.05618 | 0.3072 | 0.1051 | 0.4314 | 0.7752 | -0.3673 | -1.698 | 4.694e-06 | 0.0001641 | 2.366 | 1.242 | 6.201 | 0.8894 | 0.9505 | 1.02 | 0.02821 | 0.1408 |  |
| Gm5934 | predicted gene 5934 | 0.7339 | 0.8929 | 1.072 | 0.1006 | 0.3448 | 0.1042 | 0.4296 | 0.6653 | -0.588 | -1.703 | 0.005423 | 0.03621 | 1.996 | 0.997 | 3.118 | 0.003243 | 0.02461 | 2.098 | 1.069 | 3.343 |  |
| Gpam | glycerol-3-phosphate acyltrans | 0.7773 | 0.9123 | 0.9546 | -0.0671 | -0.2866 | 0.05205 | 0.3156 | 0.6726 | -0.5722 | -2.066 | 0.0003037 | 0.00407 | 2.171 | 1.118 | 4.36 | 0.1371 | 0.3377 | 1.317 | 0.3971 | 1.549 |  |
| C1qb | complement component 1, q subc | 0.1253 | 0.3865 | 1.323 | 0.4034 | 1.6 | 0.5383 | 0.8068 | 0.8785 | -0.1868 | -0.6262 | 0.03185 | 0.1311 | 1.555 | 0.6374 | 2.308 | 0.0005538 | 0.006525 | 2.194 | 1.133 | 4.103 |  |
| Gm12592 | predicted gene 12592 | 0.02692 | 0.165 | 1.373 | 0.4577 | 2.388 | 0.8809 | 0.9597 | 1.024 | 0.03441 | 0.1517 | 0.4803 | 0.7146 | 1.11 | 0.151 | 0.7192 | 3.252e-05 | 0.0007377 | 2.172 | 1.119 | 5.329 |  |
| Znrd1as | zinc ribbon domain containing | 0.8884 | 0.9578 | 0.9784 | -0.03153 | -0.1421 | 0.08573 | 0.3965 | 1.39 | 0.4746 | 1.808 | 0.08211 | 0.249 | 0.7346 | -0.4449 | -1.831 | 0.0002666 | 0.00375 | 0.4752 | -1.073 | -4.416 |  |
| Fxyd5 | FXYD domain-containing ion tra | 0.02093 | 0.1418 | 1.737 | 0.7964 | 2.507 | 0.6899 | 0.8837 | 1.111 | 0.1522 | 0.4049 | 0.03321 | 0.1353 | 1.736 | 0.796 | 2.288 | 0.003868 | 0.02813 | 2.199 | 1.137 | 3.266 |  |
| Snx21 | sorting nexin family member 21 | 0.9415 | 0.9796 | 1.009 | 0.01288 | 0.07428 | 0.07166 | 0.3666 | 0.763 | -0.3903 | -1.902 | 4.788e-06 | 0.0001669 | 2.26 | 1.176 | 6.192 | 0.8628 | 0.9373 | 1.023 | 0.03326 | 0.1751 |  |
| Rasgrf1 | RAS protein-specific guanine n | 0.432 | 0.7132 | 1.089 | 0.1226 | 0.802 | 0.3388 | 0.6836 | 0.8844 | -0.1772 | -0.98 | 5.141e-06 | 0.0001768 | 2.044 | 1.031 | 6.159 | 0.4404 | 0.6655 | 0.9127 | -0.1318 | -0.7873 |  |
| Gm10032 | predicted gene 10032 | 0.1844 | 0.4742 | 1.336 | 0.418 | 1.375 | 0.03862 | 0.275 | 1.737 | 0.7964 | 2.214 | 0.001908 | 0.01667 | 0.4384 | -1.19 | -3.573 | 0.8715 | 0.9418 | 1.039 | 0.05455 | 0.1638 |  |
| Cisd1 | CDGSH iron sulfur domain 1 | 0.8323 | 0.9365 | 1.027 | 0.03882 | 0.2145 | 0.1495 | 0.5005 | 0.8006 | -0.3209 | -1.499 | 5.516e-06 | 0.0001871 | 2.321 | 1.215 | 6.127 | 0.5028 | 0.7148 | 0.9105 | -0.1353 | -0.6825 |  |
| Hck | hemopoietic cell kinase | 0.02246 | 0.1479 | 1.647 | 0.7197 | 2.474 | 0.6671 | 0.8734 | 1.11 | 0.1503 | 0.4365 | 0.116 | 0.3113 | 1.438 | 0.5237 | 1.643 | 0.001363 | 0.01281 | 2.273 | 1.185 | 3.718 |  |
| Gm5423 | NA | 0.02646 | 0.1634 | 1.625 | 0.7008 | 2.396 | 0.3697 | 0.7064 | 1.246 | 0.3176 | 0.9178 | 0.7938 | 0.9075 | 0.9429 | -0.08486 | -0.2649 | 0.0009921 | 0.0101 | 2.353 | 1.235 | 3.854 |  |
| Zcchc24 | zinc finger, CCHC domain conta | 0.9519 | 0.9827 | 0.9929 | -0.01023 | -0.06113 | 0.0729 | 0.3693 | 0.7711 | -0.375 | -1.893 | 0.899 | 0.9584 | 0.9838 | -0.02358 | -0.1286 | 5.902e-06 | 0.0001966 | 2.17 | 1.118 | 6.096 |  |
| Rasl2-9 | RAS-like, family 2, locus 9 | 0.3564 | 0.6531 | 1.242 | 0.3121 | 0.944 | 0.04333 | 0.29 | 1.795 | 0.8439 | 2.157 | 0.1917 | 0.4254 | 0.7123 | -0.4894 | -1.351 | 0.006269 | 0.03985 | 0.4646 | -1.106 | -3.054 |  |
| Gbp11 | guanylate binding protein 11 | 0.5343 | 0.7843 | 0.887 | -0.173 | -0.6324 | 0.04792 | 0.3045 | 0.6232 | -0.6822 | -2.107 | 0.001516 | 0.0141 | 2.144 | 1.1 | 3.672 | 0.1696 | 0.3863 | 1.345 | 0.4271 | 1.425 |  |
| Hyls1 | HYLS1, centriolar and ciliogen | 0.2958 | 0.5998 | 1.158 | 0.2122 | 1.074 | 0.3043 | 0.6584 | 0.8429 | -0.2466 | -1.054 | 7.845e-05 | 0.001417 | 2.1 | 1.07 | 4.944 | 0.1732 | 0.391 | 1.236 | 0.3058 | 1.413 |  |
| Mrap | melanocortin 2 receptor access | 0.6372 | 0.8449 | 0.9019 | -0.149 | -0.4788 | 0.1361 | 0.4786 | 1.486 | 0.5717 | 1.553 | 0.008672 | 0.05116 | 0.5029 | -0.9916 | -2.91 | 0.00263 | 0.02105 | 0.4443 | -1.17 | -3.434 |  |
| Ppp1r3c | protein phosphatase 1, regulat | 0.3665 | 0.6613 | 1.191 | 0.2524 | 0.924 | 0.2613 | 0.6195 | 0.7719 | -0.3736 | -1.156 | 0.02129 | 0.09848 | 1.679 | 0.7477 | 2.499 | 0.001185 | 0.01157 | 2.189 | 1.13 | 3.778 |  |
| Gm609 | predicted gene 609 | 0.2777 | 0.5832 | 0.7857 | -0.3479 | -1.116 | 0.04459 | 0.2942 | 0.5781 | -0.7906 | -2.143 | 0.5182 | 0.7437 | 1.168 | 0.2247 | 0.6578 | 0.004196 | 0.02984 | 2.149 | 1.103 | 3.231 |  |
| Sdcbp2 | syndecan binding protein (synt | 0.274 | 0.5798 | 1.125 | 0.1696 | 1.125 | 0.3965 | 0.7244 | 0.8984 | -0.1546 | -0.8665 | 4.508e-06 | 0.0001591 | 2.038 | 1.027 | 6.22 | 0.7435 | 0.8756 | 0.9627 | -0.0548 | -0.3318 |  |
| Sdc3 | syndecan 3 | 0.5489 | 0.7935 | 1.077 | 0.1073 | 0.6098 | 0.1975 | 0.5606 | 0.8251 | -0.2774 | -1.333 | 0.4344 | 0.6786 | 1.112 | 0.1537 | 0.7978 | 4.448e-05 | 0.000948 | 2 | 1 | 5.192 |  |
| Tnfaip3 | tumor necrosis factor, alpha-i | 0.9186 | 0.9707 | 0.9813 | -0.0273 | -0.1036 | 0.0853 | 0.3961 | 1.479 | 0.5647 | 1.81 | 0.09917 | 0.2823 | 0.7074 | -0.4994 | -1.729 | 0.0002639 | 0.003722 | 0.4128 | -1.277 | -4.421 |  |
| H2-Ab1 | histocompatibility 2, class II | 0.1682 | 0.451 | 1.727 | 0.788 | 1.43 | 0.4639 | 0.7672 | 0.7135 | -0.4869 | -0.7468 | 0.0194 | 0.09238 | 2.898 | 1.535 | 2.542 | 0.001143 | 0.01127 | 4.89 | 2.29 | 3.793 |  |
| Srm | spermidine synthase | 0.5137 | 0.77 | 1.085 | 0.1179 | 0.665 | 0.4678 | 0.7686 | 0.898 | -0.1553 | -0.7403 | 0.1071 | 0.2963 | 0.7969 | -0.3276 | -1.687 | 8.933e-06 | 0.0002699 | 2.215 | 1.147 | 5.907 |  |
| Cd74 | CD74 antigen (invariant polype | 0.08623 | 0.3152 | 2.028 | 1.02 | 1.805 | 0.649 | 0.8648 | 0.8071 | -0.3091 | -0.4621 | 0.01546 | 0.07821 | 3.116 | 1.64 | 2.648 | 0.001145 | 0.01128 | 5.092 | 2.348 | 3.792 |  |
| BC035947 | cDNA sequence BC035947 | 0.8088 | 0.9268 | 0.9684 | -0.04633 | -0.2452 | 0.09665 | 0.416 | 1.31 | 0.3899 | 1.743 | 4.772e-05 | 0.0009701 | 0.4768 | -1.068 | -5.161 | 0.2598 | 0.4971 | 0.8467 | -0.2401 | -1.16 |  |
| Ehhadh | enoyl-Coenzyme A, hydratase/3- | 0.2904 | 0.5942 | 1.332 | 0.4131 | 1.086 | 0.03729 | 0.271 | 2.006 | 1.004 | 2.231 | 0.008114 | 0.0487 | 0.4278 | -1.225 | -2.939 | 0.2029 | 0.4292 | 0.6836 | -0.5487 | -1.317 |  |
| Slfn2 | schlafen 2 | 0.02045 | 0.1399 | 2.28 | 1.189 | 2.518 | 0.3787 | 0.7113 | 1.417 | 0.503 | 0.9004 | 0.3794 | 0.6303 | 1.38 | 0.465 | 0.899 | 0.005923 | 0.03828 | 3.016 | 1.593 | 3.079 |  |
| Terc | telomerase RNA component | 0.6513 | 0.8515 | 1.095 | 0.1313 | 0.4589 | 0.144 | 0.4917 | 0.6998 | -0.5149 | -1.521 | 0.02319 | 0.1048 | 1.706 | 0.7708 | 2.459 | 0.001338 | 0.01262 | 2.247 | 1.168 | 3.725 |  |
| 6430573F11Rik | NA | 0.6243 | 0.838 | 1.084 | 0.1165 | 0.4975 | 0.1478 | 0.4983 | 0.7488 | -0.4173 | -1.506 | 0.0001849 | 0.002776 | 2.256 | 1.173 | 4.573 | 0.111 | 0.2967 | 1.345 | 0.4279 | 1.668 |  |
| Nmt2 | N-myristoyltransferase 2 | 0.8432 | 0.941 | 1.032 | 0.0448 | 0.2004 | 0.3728 | 0.7082 | 0.846 | -0.2412 | -0.9117 | 1.237e-05 | 0.0003466 | 2.659 | 1.411 | 5.76 | 0.03537 | 0.1387 | 0.6817 | -0.5528 | -2.257 |  |
| Micu3 | mitochondrial calcium uptake f | 0.3831 | 0.6757 | 1.159 | 0.2124 | 0.8919 | 0.05286 | 0.3178 | 1.495 | 0.58 | 2.058 | 0.7859 | 0.9031 | 0.9514 | -0.07181 | -0.2753 | 0.0007898 | 0.008536 | 0.4894 | -1.031 | -3.951 |  |
| Dusp18 | dual specificity phosphatase 1 | 0.03256 | 0.185 | 1.315 | 0.3946 | 2.297 | 0.7429 | 0.9056 | 1.048 | 0.06761 | 0.3326 | 0.8544 | 0.9382 | 0.976 | -0.03498 | -0.1859 | 2.779e-05 | 0.0006491 | 2.022 | 1.016 | 5.399 |  |
| Cpped1 | calcineurin-like phosphoestera | 0.1098 | 0.3595 | 0.7449 | -0.4248 | -1.674 | 0.5645 | 0.8208 | 1.13 | 0.176 | 0.5859 | 0.001661 | 0.01509 | 0.4965 | -1.01 | -3.632 | 0.00992 | 0.05572 | 0.5774 | -0.7923 | -2.849 |  |
| Gm9801 | predicted gene 9801 | 0.07421 | 0.2912 | 0.7968 | -0.3278 | -1.884 | 0.6986 | 0.8874 | 1.058 | 0.08087 | 0.3929 | 3.517e-05 | 0.0007718 | 0.4969 | -1.009 | -5.295 | 0.2687 | 0.5075 | 0.8605 | -0.2168 | -1.138 |  |
| Pml | promyelocytic leukemia | 0.04247 | 0.2145 | 1.391 | 0.4764 | 2.167 | 0.904 | 0.9681 | 0.9782 | -0.03177 | -0.1222 | 0.2678 | 0.5205 | 1.21 | 0.2745 | 1.14 | 4.452e-05 | 0.000948 | 2.378 | 1.25 | 5.191 |  |
| Ift81 | intraflagellar transport 81 | 0.0266 | 0.1638 | 1.397 | 0.4826 | 2.394 | 0.8358 | 0.9449 | 1.035 | 0.0501 | 0.2101 | 1.568e-05 | 0.0004127 | 2.376 | 1.248 | 5.654 | 0.8267 | 0.9196 | 1.035 | 0.04898 | 0.2218 |  |
| Pcna | proliferating cell nuclear ant | 0.8264 | 0.9342 | 1.029 | 0.04123 | 0.2222 | 0.114 | 0.4467 | 0.7776 | -0.3629 | -1.653 | 0.3254 | 0.5805 | 1.153 | 0.205 | 1.008 | 7.64e-05 | 0.001439 | 2.01 | 1.007 | 4.955 |  |
| Gm2666 | NA | 0.02553 | 0.1599 | 1.592 | 0.6706 | 2.413 | 0.8235 | 0.9395 | 1.053 | 0.07432 | 0.226 | 0.01783 | 0.08693 | 1.724 | 0.7858 | 2.582 | 0.003619 | 0.02679 | 2.004 | 1.003 | 3.295 |  |
| Cbr2 | carbonyl reductase 2 | 0.5723 | 0.8085 | 0.8652 | -0.2089 | -0.5741 | 0.1744 | 0.5334 | 1.523 | 0.6065 | 1.408 | 0.0004868 | 0.005871 | 0.3169 | -1.658 | -4.158 | 0.06195 | 0.204 | 0.579 | -0.7884 | -1.977 |  |
| Ptprj | protein tyrosine phosphatase, | 0.02562 | 0.16 | 1.665 | 0.7358 | 2.412 | 0.5843 | 0.831 | 1.149 | 0.2008 | 0.5561 | 0.2939 | 0.5489 | 1.284 | 0.3602 | 1.078 | 0.00134 | 0.01263 | 2.37 | 1.245 | 3.725 |  |
| Slc5a3 | solute carrier family 5 (inosi | 0.02393 | 0.1535 | 0.6608 | -0.5977 | -2.444 | 0.6406 | 0.8605 | 0.9093 | -0.1372 | -0.474 | 5.989e-05 | 0.001151 | 0.3907 | -1.356 | -5.061 | 0.9247 | 0.9664 | 0.9824 | -0.02566 | -0.09577 |  |
| Nif3l1 | Ngg1 interacting factor 3-like | 0.04379 | 0.2182 | 0.7711 | -0.3751 | -2.152 | 0.8776 | 0.9588 | 1.023 | 0.03216 | 0.156 | 1.76e-05 | 0.0004509 | 0.4765 | -1.069 | -5.602 | 0.4344 | 0.6606 | 0.8998 | -0.1523 | -0.7977 |  |
| Zc3hav1 | zinc finger CCCH type, antivir | 0.05394 | 0.2455 | 1.394 | 0.4788 | 2.048 | 0.8604 | 0.9534 | 0.9664 | -0.04928 | -0.1781 | 0.06468 | 0.212 | 1.415 | 0.5008 | 1.955 | 0.0004113 | 0.005209 | 2.119 | 1.083 | 4.231 |  |
| Syt12 | synaptotagmin XII | 0.7098 | 0.8813 | 0.9263 | -0.1105 | -0.3775 | 0.2271 | 0.5884 | 1.349 | 0.4316 | 1.246 | 9.952e-06 | 0.0002916 | 0.272 | -1.878 | -5.858 | 0.4413 | 0.666 | 1.191 | 0.2519 | 0.7856 |  |
| Lrg1 | leucine-rich alpha-2-glycoprot | 0.5552 | 0.7977 | 0.8913 | -0.166 | -0.6 | 0.1955 | 0.5574 | 1.355 | 0.4385 | 1.339 | 1.293e-05 | 0.0003569 | 0.2995 | -1.74 | -5.74 | 0.8099 | 0.9114 | 0.9501 | -0.07388 | -0.2438 |  |
| Mir7082 | microRNA 7082 | 0.4836 | 0.7507 | 1.155 | 0.208 | 0.7138 | 0.06311 | 0.3468 | 1.601 | 0.6786 | 1.968 | 0.5816 | 0.7869 | 0.8834 | -0.1788 | -0.5602 | 0.000973 | 0.009951 | 0.4255 | -1.233 | -3.862 |  |
| Ifit3b | interferon-induced protein wit | 0.235 | 0.5363 | 0.7752 | -0.3673 | -1.224 | 0.3882 | 0.7177 | 1.242 | 0.3131 | 0.8821 | 0.001315 | 0.01267 | 0.4273 | -1.227 | -3.733 | 0.02222 | 0.09975 | 0.5686 | -0.8145 | -2.479 |  |
| Agpat2 | 1-acylglycerol-3-phosphate O-a | 0.2151 | 0.5136 | 0.7641 | -0.3881 | -1.28 | 0.4177 | 0.7388 | 1.228 | 0.2968 | 0.8275 | 0.0004561 | 0.005592 | 0.3815 | -1.39 | -4.186 | 0.05913 | 0.1976 | 0.6309 | -0.6645 | -2.001 |  |
| Dhx58 | DEXH (Asp-Glu-X-His) box polyp | 0.2797 | 0.5848 | 1.227 | 0.2952 | 1.111 | 0.04603 | 0.2985 | 1.59 | 0.6689 | 2.127 | 0.001585 | 0.01454 | 0.4786 | -1.063 | -3.653 | 0.8449 | 0.9291 | 0.9608 | -0.05767 | -0.1981 |  |
| Thpo | thrombopoietin | 0.7014 | 0.8778 | 0.9454 | -0.08098 | -0.3889 | 0.06476 | 0.3493 | 0.7162 | -0.4816 | -1.955 | 5.555e-05 | 0.001088 | 2.238 | 1.162 | 5.094 | 0.8632 | 0.9374 | 1.028 | 0.03982 | 0.1746 |  |
| Tppp | tubulin polymerization promoti | 0.3842 | 0.677 | 0.8434 | -0.2457 | -0.8898 | 0.4375 | 0.7509 | 0.8357 | -0.2589 | -0.7923 | 0.002176 | 0.01842 | 2.09 | 1.064 | 3.516 | 0.002942 | 0.02286 | 0.4917 | -1.024 | -3.385 |  |
| Gm22188 | NA | 0.9725 | 0.9901 | 0.9911 | -0.01292 | -0.03489 | 0.06833 | 0.3586 | 0.557 | -0.8442 | -1.927 | 0.003329 | 0.02524 | 2.551 | 1.351 | 3.332 | 0.01066 | 0.05885 | 2.208 | 1.142 | 2.817 |  |
| Ang | angiogenin, ribonuclease, RNas | 0.1708 | 0.4546 | 1.304 | 0.383 | 1.421 | 0.4829 | 0.7768 | 0.8538 | -0.228 | -0.7149 | 0.00158 | 0.01451 | 2.112 | 1.079 | 3.654 | 0.01891 | 0.08899 | 1.687 | 0.7541 | 2.554 |  |
| D3Ertd751e | DNA segment, Chr 3, ERATO Doi | 0.6219 | 0.8368 | 0.9067 | -0.1412 | -0.5009 | 0.1823 | 0.5428 | 1.377 | 0.461 | 1.382 | 0.06152 | 0.2054 | 0.6543 | -0.6119 | -1.981 | 0.0009585 | 0.009827 | 0.4368 | -1.195 | -3.868 |  |
| Cd9 | CD9 antigen | 0.9602 | 0.9861 | 1.01 | 0.01397 | 0.05052 | 0.07818 | 0.3829 | 0.6564 | -0.6073 | -1.857 | 0.001814 | 0.01607 | 2.127 | 1.089 | 3.595 | 0.01868 | 0.08819 | 1.712 | 0.7753 | 2.56 |  |
| Gm20883 | predicted gene, 20883 | 0.2593 | 0.5644 | 1.486 | 0.5711 | 1.161 | 0.3845 | 0.7155 | 0.6986 | -0.5175 | -0.8892 | 0.01396 | 0.07269 | 2.736 | 1.452 | 2.694 | 0.003072 | 0.0236 | 3.516 | 1.814 | 3.367 |  |
| Polr3gl | polymerase (RNA) III (DNA dire | 0.282 | 0.5867 | 1.14 | 0.1886 | 1.106 | 0.5288 | 0.8023 | 0.9143 | -0.1293 | -0.641 | 8.747e-06 | 0.0002644 | 2.151 | 1.105 | 5.916 | 0.4267 | 0.6533 | 0.9003 | -0.1516 | -0.8113 |  |
| Cxcl12 | chemokine (C-X-C motif) ligand | 0.4026 | 0.6922 | 1.15 | 0.2021 | 0.8551 | 0.6012 | 0.8405 | 1.108 | 0.1485 | 0.5311 | 0.0006954 | 0.007677 | 2.052 | 1.037 | 4.006 | 0.002945 | 0.02286 | 0.5447 | -0.8764 | -3.385 |  |
| Padi2 | peptidyl arginine deiminase, t | 0.1201 | 0.378 | 0.8045 | -0.3138 | -1.624 | 0.7874 | 0.9238 | 1.044 | 0.06251 | 0.2734 | 0.5942 | 0.7952 | 1.083 | 0.1146 | 0.5415 | 9.595e-06 | 0.000284 | 0.4223 | -1.244 | -5.875 |  |
| Olfr47 | olfactory receptor 47 | 0.7128 | 0.8829 | 1.085 | 0.1181 | 0.3733 | 0.06402 | 0.3478 | 1.663 | 0.7337 | 1.961 | 0.004963 | 0.03386 | 0.4685 | -1.094 | -3.157 | 0.04194 | 0.1564 | 0.5933 | -0.7531 | -2.174 |  |
| Slc9a3r2 | solute carrier family 9 (sodiu | 0.1342 | 0.4024 | 1.27 | 0.3447 | 1.561 | 0.8645 | 0.9543 | 0.9692 | -0.04515 | -0.1728 | 8.735e-06 | 0.0002644 | 2.697 | 1.431 | 5.917 | 0.2921 | 0.5332 | 0.8341 | -0.2617 | -1.082 |  |
| Tax1bp3 | Tax1 (human T cell leukemia vi | 0.06279 | 0.266 | 0.752 | -0.4112 | -1.971 | 0.8045 | 0.9315 | 1.044 | 0.06193 | 0.2508 | 0.0002832 | 0.003857 | 0.4988 | -1.004 | -4.39 | 0.08393 | 0.2489 | 0.7496 | -0.4158 | -1.819 |  |
| Serpina3c | serine (or cysteine) peptidase | 0.2139 | 0.5127 | 0.704 | -0.5064 | -1.284 | 0.4329 | 0.7485 | 1.296 | 0.3736 | 0.8005 | 0.001171 | 0.01152 | 0.322 | -1.635 | -3.783 | 0.03062 | 0.1253 | 0.4981 | -1.005 | -2.327 |  |
| Pamr1 | peptidase domain containing as | 0.03587 | 0.1951 | 1.359 | 0.4421 | 2.25 | 0.98 | 0.9942 | 0.9959 | -0.005902 | -0.02539 | 3.575e-05 | 0.0007792 | 2.201 | 1.138 | 5.288 | 0.3501 | 0.5884 | 1.153 | 0.2059 | 0.9568 |  |
| Ripk4 | receptor-interacting serine-th | 0.06388 | 0.2689 | 1.37 | 0.4541 | 1.962 | 0.9697 | 0.9902 | 1.007 | 0.01053 | 0.03844 | 7.984e-06 | 0.000248 | 2.849 | 1.511 | 5.958 | 0.4858 | 0.7009 | 0.8827 | -0.1801 | -0.7102 |  |
| LOC102640399 | NA | 0.7434 | 0.8975 | 1.084 | 0.1161 | 0.3319 | 0.126 | 0.4657 | 0.6326 | -0.6606 | -1.597 | 0.006721 | 0.04244 | 2.232 | 1.158 | 3.023 | 0.006193 | 0.03951 | 2.253 | 1.172 | 3.06 |  |
| Mad2l1bp | MAD2L1 binding protein | 0.5954 | 0.8217 | 1.117 | 0.1597 | 0.5397 | 0.177 | 0.5374 | 0.7121 | -0.4899 | -1.399 | 0.003689 | 0.02727 | 2.093 | 1.065 | 3.287 | 0.01304 | 0.06821 | 1.845 | 0.8834 | 2.725 |  |
| Slco2a1 | solute carrier organic anion t | 0.02754 | 0.1673 | 1.658 | 0.7294 | 2.377 | 0.523 | 0.7998 | 1.178 | 0.236 | 0.6502 | 0.2248 | 0.4684 | 1.339 | 0.421 | 1.253 | 0.005033 | 0.03397 | 2.084 | 1.059 | 3.151 |  |
| B330016D10Rik | RIKEN cDNA B330016D10 gene | 0.8439 | 0.9412 | 0.9712 | -0.04217 | -0.1995 | 0.1621 | 0.5177 | 0.7775 | -0.3631 | -1.452 | 4.046e-05 | 0.000852 | 2.316 | 1.212 | 5.233 | 0.2951 | 0.5359 | 0.8415 | -0.249 | -1.075 |  |
| Pld4 | phospholipase D family, member | 0.03445 | 0.1909 | 1.526 | 0.6101 | 2.27 | 0.7708 | 0.9167 | 1.067 | 0.09394 | 0.2954 | 0.2165 | 0.4581 | 1.298 | 0.3758 | 1.276 | 0.0007339 | 0.008058 | 2.254 | 1.173 | 3.983 |  |
| Efnb1 | ephrin B1 | 0.06404 | 0.2694 | 1.36 | 0.4438 | 1.96 | 0.8892 | 0.9623 | 1.027 | 0.03779 | 0.1411 | 1.507e-05 | 0.0004017 | 2.651 | 1.406 | 5.671 | 0.4565 | 0.6788 | 0.8776 | -0.1883 | -0.7594 |  |
| Prkcd | protein kinase C, delta | 0.3028 | 0.6059 | 1.139 | 0.188 | 1.058 | 0.3806 | 0.7124 | 0.8775 | -0.1886 | -0.8967 | 0.9931 | 0.9977 | 0.9988 | -0.001702 | -0.008742 | 1.104e-05 | 0.0003199 | 2.191 | 1.131 | 5.811 |  |
| Ret | ret proto-oncogene | 0.05104 | 0.2378 | 0.6862 | -0.5433 | -2.076 | 0.927 | 0.9767 | 1.02 | 0.02874 | 0.09282 | 0.7025 | 0.8594 | 0.9259 | -0.1111 | -0.3875 | 1.775e-05 | 0.0004607 | 0.3287 | -1.605 | -5.598 |  |
| Fermt3 | fermitin family member 3 | 0.02986 | 0.1752 | 1.649 | 0.7213 | 2.339 | 0.6901 | 0.8838 | 1.108 | 0.1476 | 0.4045 | 0.08151 | 0.248 | 1.537 | 0.6198 | 1.835 | 0.00393 | 0.02847 | 2.145 | 1.101 | 3.259 |  |
| Tmem47 | transmembrane protein 47 | 0.8395 | 0.939 | 0.9707 | -0.04291 | -0.2052 | 0.1311 | 0.472 | 1.31 | 0.3896 | 1.574 | 0.4526 | 0.6927 | 0.8855 | -0.1755 | -0.7661 | 8.727e-05 | 0.001584 | 0.4595 | -1.122 | -4.898 |  |
| Hnf4a | hepatic nuclear factor 4, alph | 0.9093 | 0.967 | 0.979 | -0.03058 | -0.1154 | 0.09472 | 0.4126 | 1.464 | 0.5503 | 1.754 | 0.0004432 | 0.00546 | 0.4294 | -1.219 | -4.199 | 0.08672 | 0.2542 | 0.6958 | -0.5233 | -1.802 |  |
| Spi1 | spleen focus forming virus (SF | 0.03118 | 0.1799 | 1.714 | 0.7772 | 2.318 | 0.5378 | 0.8066 | 1.188 | 0.2487 | 0.627 | 0.6048 | 0.8014 | 1.143 | 0.1932 | 0.5259 | 0.00115 | 0.01132 | 2.625 | 1.392 | 3.79 |  |
| Gm23460 | predicted gene, 23460 | 0.07179 | 0.2862 | 0.7639 | -0.3886 | -1.901 | 0.8234 | 0.9395 | 1.039 | 0.05469 | 0.2261 | 0.7928 | 0.9068 | 0.9595 | -0.05961 | -0.2662 | 1.5e-05 | 0.0004048 | 0.4146 | -1.27 | -5.673 |  |
| Gsap | gamma-secretase activating pro | 0.8015 | 0.9229 | 1.047 | 0.06569 | 0.2548 | 0.1324 | 0.4738 | 0.7177 | -0.4785 | -1.569 | 0.08439 | 0.2536 | 1.427 | 0.5129 | 1.816 | 0.0007658 | 0.008336 | 2.173 | 1.12 | 3.965 |  |
| Cldn1 | claudin 1 | 0.3426 | 0.6412 | 1.211 | 0.2765 | 0.9722 | 0.3135 | 0.6637 | 0.7857 | -0.3479 | -1.034 | 0.001946 | 0.01694 | 2.159 | 1.11 | 3.564 | 0.02371 | 0.1047 | 1.697 | 0.7627 | 2.448 |  |
| Snord32a | small nucleolar RNA, C/D box 3 | 0.6375 | 0.8449 | 0.85 | -0.2344 | -0.4785 | 0.0648 | 0.3494 | 0.456 | -1.133 | -1.954 | 0.005088 | 0.0345 | 3.223 | 1.688 | 3.146 | 0.07528 | 0.2321 | 2.01 | 1.007 | 1.876 |  |
| Vsir | V-set immunoregulatory recepto | 0.2495 | 0.5532 | 1.231 | 0.2999 | 1.186 | 0.4075 | 0.7316 | 0.8391 | -0.2531 | -0.8461 | 0.0007732 | 0.008336 | 2.139 | 1.097 | 3.96 | 0.05597 | 0.19 | 1.476 | 0.562 | 2.029 |  |
| Asf1b | anti-silencing function 1B his | 0.4402 | 0.7206 | 1.148 | 0.1995 | 0.7875 | 0.6841 | 0.8815 | 0.9178 | -0.1237 | -0.4128 | 0.05559 | 0.1915 | 0.6764 | -0.5641 | -2.033 | 2.081e-05 | 0.0005216 | 2.895 | 1.534 | 5.527 |  |
| Sorbs1 | sorbin and SH3 domain containi | 0.2546 | 0.5586 | 1.161 | 0.2154 | 1.173 | 0.6574 | 0.869 | 0.9344 | -0.09784 | -0.4502 | 0.3404 | 0.5963 | 0.8727 | -0.1965 | -0.9766 | 1.588e-05 | 0.0004232 | 2.198 | 1.136 | 5.648 |  |
| Ppargc1b | peroxisome proliferative activ | 0.9986 | 0.9993 | 0.9996 | -0.0006033 | -0.001723 | 0.1993 | 0.562 | 1.464 | 0.5502 | 1.327 | 0.2777 | 0.5318 | 1.346 | 0.4282 | 1.116 | 2.636e-05 | 0.0006228 | 0.2364 | -2.08 | -5.422 |  |
| Gm15372 | predicted gene 15372 | 0.3383 | 0.6373 | 1.433 | 0.5192 | 0.9811 | 0.0632 | 0.3468 | 2.348 | 1.232 | 1.967 | 0.8269 | 0.9243 | 0.9148 | -0.1285 | -0.2216 | 0.002443 | 0.01996 | 0.2484 | -2.009 | -3.466 |  |
| Gm10488 | predicted gene 10488 | 0.8433 | 0.9411 | 1.054 | 0.07591 | 0.2003 | 0.1121 | 0.4442 | 0.5965 | -0.7455 | -1.662 | 0.01059 | 0.05919 | 2.252 | 1.171 | 2.82 | 0.005467 | 0.03609 | 2.451 | 1.293 | 3.115 |  |
| Erbb3 | erb-b2 receptor tyrosine kinas | 0.03944 | 0.2055 | 1.335 | 0.4167 | 2.204 | 0.8701 | 0.9562 | 1.026 | 0.03706 | 0.1656 | 0.8398 | 0.9314 | 1.03 | 0.04242 | 0.2048 | 3.524e-05 | 0.0007882 | 2.139 | 1.097 | 5.294 |  |
| Gm5946 | predicted gene 5946 | 0.1918 | 0.484 | 0.7835 | -0.3519 | -1.351 | 0.5213 | 0.7987 | 1.15 | 0.2012 | 0.6529 | 0.09554 | 0.2755 | 0.7075 | -0.4992 | -1.75 | 0.0006045 | 0.006979 | 0.4475 | -1.16 | -4.066 |  |
| Gcgr | glucagon receptor | 0.5561 | 0.798 | 1.087 | 0.1207 | 0.5986 | 0.3278 | 0.6751 | 0.8472 | -0.2392 | -1.003 | 1.566e-05 | 0.0004127 | 2.376 | 1.248 | 5.654 | 0.4539 | 0.6767 | 0.8897 | -0.1687 | -0.7639 |  |
| Has2 | hyaluronan synthase 2 | 0.03484 | 0.1919 | 0.7007 | -0.5131 | -2.264 | 0.9647 | 0.9883 | 0.9917 | -0.01201 | -0.04479 | 0.0006167 | 0.007025 | 0.4975 | -1.007 | -4.057 | 0.05944 | 0.1982 | 0.709 | -0.4961 | -1.999 |  |
| Nmnat3 | nicotinamide nucleotide adenyl | 0.8205 | 0.9321 | 1.028 | 0.03983 | 0.2299 | 0.196 | 0.5585 | 0.8269 | -0.2742 | -1.338 | 1.845e-05 | 0.0004681 | 2.084 | 1.059 | 5.581 | 0.5445 | 0.7451 | 0.9221 | -0.117 | -0.6165 |  |
| Casq2 | calsequestrin 2 | 0.03376 | 0.1885 | 0.656 | -0.6082 | -2.279 | 0.8935 | 0.964 | 0.9708 | -0.04282 | -0.1356 | 0.0005196 | 0.006153 | 0.4331 | -1.207 | -4.131 | 0.11 | 0.295 | 0.7126 | -0.4889 | -1.673 |  |
| Tyrobp | TYRO protein tyrosine kinase b | 0.127 | 0.3893 | 1.392 | 0.4774 | 1.593 | 0.6281 | 0.8545 | 0.8861 | -0.1745 | -0.492 | 0.01825 | 0.08833 | 1.795 | 0.8443 | 2.571 | 0.002946 | 0.02286 | 2.161 | 1.112 | 3.385 |  |
| Mgl2 | macrophage galactose N-acetyl- | 0.03526 | 0.193 | 0.6324 | -0.6611 | -2.258 | 0.9334 | 0.9783 | 0.9799 | -0.02931 | -0.08464 | 0.00113 | 0.01122 | 0.4299 | -1.218 | -3.798 | 0.04739 | 0.1698 | 0.6252 | -0.6775 | -2.113 |  |
| Gm5460 | predicted gene 5460 | 0.4251 | 0.7092 | 1.241 | 0.3115 | 0.8143 | 0.2953 | 0.6506 | 0.7138 | -0.4864 | -1.075 | 3.614e-05 | 0.0007844 | 4.639 | 2.214 | 5.283 | 0.6721 | 0.8325 | 1.133 | 0.18 | 0.4295 |  |
| Nnat | neuronatin | 0.5204 | 0.7754 | 0.7276 | -0.4588 | -0.6543 | 0.2521 | 0.6119 | 1.97 | 0.9785 | 1.179 | 0.1137 | 0.3077 | 0.4145 | -1.271 | -1.654 | 0.0007757 | 0.008412 | 0.1215 | -3.041 | -3.959 |  |
| Arap2 | ArfGAP with RhoGAP domain, ank | 0.6479 | 0.8495 | 1.11 | 0.1499 | 0.4636 | 0.07139 | 0.3662 | 1.657 | 0.7285 | 1.904 | 0.00238 | 0.01967 | 0.4258 | -1.232 | -3.477 | 0.1701 | 0.3867 | 0.7051 | -0.5041 | -1.423 |  |
| Wnt2b | wingless-type MMTV integration | 0.03029 | 0.1765 | 1.525 | 0.6085 | 2.332 | 0.5471 | 0.8121 | 1.14 | 0.1891 | 0.6126 | 0.001294 | 0.0125 | 2.098 | 1.069 | 3.74 | 0.5159 | 0.7251 | 1.14 | 0.1891 | 0.6615 |  |
| Plekhf1 | pleckstrin homology domain con | 0.152 | 0.4285 | 1.35 | 0.4325 | 1.489 | 0.5943 | 0.8366 | 0.879 | -0.186 | -0.5413 | 2.564e-05 | 0.0006052 | 3.315 | 1.729 | 5.434 | 0.6946 | 0.8468 | 1.092 | 0.1267 | 0.3983 |  |
| Sorl1 | sortilin-related receptor, LDL | 0.03378 | 0.1885 | 0.6531 | -0.6145 | -2.279 | 0.7126 | 0.8932 | 0.9207 | -0.1192 | -0.3737 | 7.009e-05 | 0.001289 | 0.3598 | -1.475 | -4.993 | 0.97 | 0.9862 | 0.9922 | -0.01126 | -0.03814 |  |
| Gnai1 | guanine nucleotide binding pro | 0.7413 | 0.8965 | 1.137 | 0.1854 | 0.3348 | 0.08109 | 0.3892 | 2.303 | 1.204 | 1.837 | 0.07263 | 0.2291 | 0.4508 | -1.15 | -1.895 | 0.005142 | 0.03447 | 0.2669 | -1.906 | -3.142 |  |
| Gm5168 | predicted gene 5168 | 0.8084 | 0.9266 | 1.052 | 0.07288 | 0.2457 | 0.1336 | 0.4754 | 0.6836 | -0.5488 | -1.564 | 0.01702 | 0.08385 | 1.797 | 0.8458 | 2.603 | 0.005199 | 0.03477 | 2.027 | 1.019 | 3.137 |  |
| Dnajb3 | DnaJ heat shock protein family | 0.9667 | 0.9884 | 0.9929 | -0.0103 | -0.04233 | 0.09062 | 0.4055 | 0.7012 | -0.5121 | -1.778 | 0.0001682 | 0.002585 | 2.346 | 1.23 | 4.614 | 0.3146 | 0.5553 | 1.21 | 0.2751 | 1.032 |  |
| Itga3 | integrin alpha 3 | 0.3027 | 0.6058 | 1.182 | 0.2406 | 1.058 | 0.3876 | 0.7173 | 0.8481 | -0.2377 | -0.8834 | 4.745e-05 | 0.0009672 | 2.439 | 1.286 | 5.163 | 0.5576 | 0.7548 | 1.108 | 0.1486 | 0.5964 |  |
| Cbs | cystathionine beta-synthase | 0.07671 | 0.2973 | 1.4 | 0.4857 | 1.867 | 0.8082 | 0.9333 | 0.9489 | -0.07574 | -0.246 | 4.221e-05 | 0.0008806 | 2.802 | 1.486 | 5.215 | 0.4954 | 0.7084 | 1.147 | 0.198 | 0.6945 |  |
| Hist2h3b | NA | 0.8018 | 0.923 | 0.9621 | -0.0558 | -0.2543 | 0.1729 | 0.5307 | 0.7754 | -0.367 | -1.414 | 0.4424 | 0.6852 | 0.8776 | -0.1883 | -0.7837 | 0.0001485 | 0.002371 | 2.176 | 1.122 | 4.668 |  |
| Ifi204 | interferon activated gene 204 | 0.5083 | 0.7662 | 1.186 | 0.2458 | 0.6736 | 0.2564 | 0.616 | 0.7049 | -0.5045 | -1.168 | 0.03066 | 0.1277 | 1.905 | 0.9299 | 2.326 | 0.003541 | 0.02635 | 2.499 | 1.321 | 3.305 |  |
| Trav16 | T cell receptor alpha variable | 0.9304 | 0.9754 | 1.022 | 0.03075 | 0.08848 | 0.09497 | 0.4128 | 1.648 | 0.7208 | 1.753 | 0.06232 | 0.2072 | 0.5939 | -0.7517 | -1.974 | 0.00246 | 0.02003 | 0.401 | -1.318 | -3.463 |  |
| H2-Aa | histocompatibility 2, class II | 0.03975 | 0.2064 | 2.49 | 1.316 | 2.2 | 0.7966 | 0.9282 | 1.137 | 0.1849 | 0.2612 | 0.06245 | 0.2075 | 2.451 | 1.293 | 1.973 | 0.004451 | 0.03105 | 4.288 | 2.1 | 3.205 |  |
| Crip3 | cysteine-rich protein 3 | 0.6191 | 0.8353 | 1.133 | 0.1806 | 0.505 | 0.1295 | 0.4704 | 1.59 | 0.669 | 1.581 | 0.6785 | 0.846 | 1.121 | 0.1648 | 0.4207 | 0.0004835 | 0.00588 | 0.3231 | -1.63 | -4.161 |  |
| Selk | NA | 0.4501 | 0.7273 | 0.8729 | -0.1962 | -0.7704 | 0.07105 | 0.3654 | 0.6716 | -0.5744 | -1.907 | 0.001788 | 0.01588 | 2.006 | 1.004 | 3.601 | 0.6451 | 0.8143 | 1.095 | 0.1304 | 0.4676 |  |
| Dcpp3 | demilune cell and parotid prot | 0.6248 | 0.8382 | 0.9011 | -0.1503 | -0.4967 | 0.2166 | 0.5779 | 1.372 | 0.4567 | 1.276 | 0.0451 | 0.1664 | 0.612 | -0.7084 | -2.138 | 0.003141 | 0.02404 | 0.4625 | -1.113 | -3.357 |  |
| Pcdhgc3 | protocadherin gamma subfamily | 0.08249 | 0.3072 | 0.6696 | -0.5787 | -1.828 | 0.3819 | 0.7134 | 0.7928 | -0.3349 | -0.8942 | 0.004191 | 0.02997 | 0.46 | -1.12 | -3.231 | 0.2751 | 0.5145 | 1.31 | 0.389 | 1.122 |  |
| Bok | BCL2-related ovarian killer | 0.349 | 0.6467 | 0.8714 | -0.1986 | -0.959 | 0.3747 | 0.709 | 1.167 | 0.2225 | 0.908 | 2.301e-05 | 0.0005563 | 0.4223 | -1.244 | -5.482 | 0.9322 | 0.9698 | 0.9865 | -0.01956 | -0.08621 |  |
| Adrm1 | adhesion regulating molecule 1 | 0.3996 | 0.6895 | 1.11 | 0.1509 | 0.8607 | 0.5278 | 0.8023 | 0.9118 | -0.1333 | -0.6426 | 0.4099 | 0.6581 | 0.894 | -0.1616 | -0.8417 | 3.628e-05 | 0.0008066 | 2.02 | 1.014 | 5.281 |  |
| Ier5 | immediate early response 5 | 0.06303 | 0.2666 | 1.377 | 0.4616 | 1.969 | 0.8597 | 0.9534 | 1.035 | 0.04966 | 0.179 | 0.8054 | 0.9136 | 0.9565 | -0.06413 | -0.2497 | 5.996e-05 | 0.001189 | 2.462 | 1.3 | 5.061 |  |
| Ppm1k | protein phosphatase 1K (PP2C d | 0.8827 | 0.9558 | 1.028 | 0.03966 | 0.1495 | 0.2096 | 0.5722 | 1.326 | 0.407 | 1.296 | 0.3516 | 0.6056 | 1.212 | 0.2772 | 0.9539 | 0.0001241 | 0.002068 | 0.3845 | -1.379 | -4.745 |  |
| Entpd6 | ectonucleoside triphosphate di | 0.1231 | 0.3834 | 0.8165 | -0.2924 | -1.61 | 0.7884 | 0.9243 | 1.041 | 0.05846 | 0.272 | 1.709e-05 | 0.0004391 | 0.461 | -1.117 | -5.615 | 0.7494 | 0.8796 | 1.046 | 0.06443 | 0.3238 |  |
| Gtf3c4 | general transcription factor I | 0.05352 | 0.2445 | 1.319 | 0.3998 | 2.052 | 0.9714 | 0.9908 | 1.006 | 0.008385 | 0.03637 | 0.6337 | 0.8187 | 1.074 | 0.1033 | 0.4839 | 6.624e-05 | 0.001296 | 2.101 | 1.071 | 5.018 |  |
| Acy1 | aminoacylase 1 | 0.8487 | 0.943 | 1.025 | 0.0352 | 0.1933 | 0.1583 | 0.5116 | 0.8034 | -0.3157 | -1.466 | 0.9726 | 0.99 | 1.005 | 0.006932 | 0.03476 | 3.945e-05 | 0.0008571 | 2.065 | 1.046 | 5.244 |  |
| Gsk3b | glycogen synthase kinase 3 bet | 0.238 | 0.5396 | 1.163 | 0.2179 | 1.216 | 0.6554 | 0.8678 | 0.9356 | -0.09603 | -0.453 | 0.5861 | 0.7903 | 0.9275 | -0.1086 | -0.5535 | 3.084e-05 | 0.0007089 | 2.071 | 1.05 | 5.353 |  |
| Gbp6 | guanylate binding protein 6 | 0.551 | 0.7945 | 0.8805 | -0.1836 | -0.6065 | 0.09291 | 0.4105 | 0.6452 | -0.6321 | -1.765 | 0.7133 | 0.8661 | 1.089 | 0.1236 | 0.3727 | 0.001472 | 0.01359 | 2.332 | 1.222 | 3.684 |  |
| D16Ertd472e | DNA segment, Chr 16, ERATO Doi | 0.04956 | 0.2345 | 1.438 | 0.5242 | 2.091 | 0.4894 | 0.7806 | 1.156 | 0.209 | 0.7042 | 0.001194 | 0.0117 | 2.052 | 1.037 | 3.774 | 0.744 | 0.8759 | 0.9389 | -0.09098 | -0.3312 |  |
| Gm15085 | predicted gene 15085 | 0.5981 | 0.8238 | 1.108 | 0.1475 | 0.5357 | 0.238 | 0.5977 | 0.7597 | -0.3964 | -1.216 | 0.08364 | 0.2522 | 1.463 | 0.5494 | 1.821 | 0.001842 | 0.01614 | 2.118 | 1.083 | 3.588 |  |
| Lenep | lens epithelial protein | 0.9047 | 0.9645 | 0.9814 | -0.02706 | -0.1213 | 0.1417 | 0.4882 | 1.323 | 0.4039 | 1.53 | 0.2822 | 0.5367 | 0.8293 | -0.2701 | -1.105 | 0.0004574 | 0.005635 | 0.4922 | -1.023 | -4.185 |  |
| Pet117 | PET117 homolog | 0.569 | 0.8062 | 0.848 | -0.2378 | -0.579 | 0.2391 | 0.5988 | 1.505 | 0.5897 | 1.213 | 0.001176 | 0.01156 | 0.3075 | -1.701 | -3.781 | 0.09777 | 0.2737 | 0.5817 | -0.7816 | -1.737 |  |
| Gm21244 | predicted gene, 21244 | 0.4655 | 0.7382 | 1.223 | 0.2906 | 0.744 | 0.3008 | 0.6548 | 0.7116 | -0.4909 | -1.062 | 0.03533 | 0.141 | 1.953 | 0.9659 | 2.258 | 0.00428 | 0.03029 | 2.6 | 1.379 | 3.222 |  |
| Mup-ps7 | major urinary protein, pseudog | 0.2847 | 0.5896 | 1.28 | 0.3558 | 1.099 | 0.4363 | 0.7507 | 0.8099 | -0.3042 | -0.7943 | 0.02297 | 0.104 | 1.832 | 0.8734 | 2.463 | 0.005388 | 0.03565 | 2.153 | 1.107 | 3.121 |  |
| Tmem45b | transmembrane protein 45b | 0.05012 | 0.2356 | 0.5214 | -0.9395 | -2.085 | 0.9044 | 0.9682 | 0.9561 | -0.06483 | -0.1216 | 0.1064 | 0.2949 | 0.5607 | -0.8347 | -1.691 | 0.00169 | 0.01509 | 0.2893 | -1.789 | -3.625 |  |
| Dusp3 | dual specificity phosphatase 3 | 0.1707 | 0.4546 | 0.8351 | -0.26 | -1.421 | 0.863 | 0.9538 | 1.027 | 0.03785 | 0.1748 | 0.4286 | 0.6741 | 1.119 | 0.1619 | 0.808 | 3.751e-05 | 0.0008239 | 0.4811 | -1.055 | -5.266 |  |
| LOC105243233 | NA | 0.5052 | 0.7644 | 1.169 | 0.2251 | 0.6786 | 0.2767 | 0.6324 | 0.7377 | -0.4389 | -1.118 | 0.001011 | 0.01027 | 2.634 | 1.397 | 3.845 | 0.1173 | 0.3066 | 1.51 | 0.5948 | 1.637 |  |
| Musk | muscle, skeletal, receptor tyr | 0.9998 | 0.9998 | 1 | 6.667e-05 | 0.0002457 | 0.1148 | 0.4481 | 0.6929 | -0.5293 | -1.649 | 0.1076 | 0.2971 | 1.415 | 0.5007 | 1.685 | 0.001562 | 0.01419 | 2.125 | 1.087 | 3.659 |  |
| Cbx4 | chromobox 4 | 0.4882 | 0.753 | 1.132 | 0.1789 | 0.7062 | 0.1243 | 0.4632 | 1.396 | 0.4809 | 1.605 | 0.001197 | 0.01172 | 0.484 | -1.047 | -3.773 | 0.6173 | 0.7962 | 1.103 | 0.1408 | 0.5075 |  |
| Rufy4 | RUN and FYVE domain containing | 0.1956 | 0.4889 | 1.174 | 0.2318 | 1.339 | 0.7258 | 0.8982 | 0.9508 | -0.07285 | -0.3557 | 2.514e-05 | 0.0005977 | 2.045 | 1.032 | 5.443 | 0.5402 | 0.7421 | 0.9214 | -0.1182 | -0.6232 |  |
| Slc38a3 | solute carrier family 38, memb | 0.3031 | 0.6062 | 0.8232 | -0.2806 | -1.057 | 0.7961 | 0.928 | 0.9446 | -0.08228 | -0.2619 | 0.0002466 | 0.003456 | 0.4077 | -1.294 | -4.45 | 0.02605 | 0.1123 | 1.624 | 0.6992 | 2.404 |  |
| Csrp3 | cysteine and glycine-rich prot | 0.05123 | 0.2383 | 2.975 | 1.573 | 2.074 | 0.3752 | 0.7093 | 1.758 | 0.8138 | 0.907 | 0.7811 | 0.9006 | 0.8503 | -0.234 | -0.2817 | 0.006733 | 0.04215 | 5.699 | 2.511 | 3.022 |  |
| Dgat2 | diacylglycerol O-acyltransfera | 0.09902 | 0.3401 | 0.6224 | -0.6841 | -1.73 | 0.8675 | 0.9555 | 1.056 | 0.07905 | 0.169 | 2.087e-05 | 0.0005131 | 0.1903 | -2.393 | -5.526 | 0.8127 | 0.9125 | 1.075 | 0.104 | 0.2401 |  |
| Mir30c-2 | microRNA 30c-2 | 0.5322 | 0.783 | 1.107 | 0.1468 | 0.6357 | 0.3988 | 0.726 | 0.8494 | -0.2355 | -0.8622 | 3.811e-05 | 0.000815 | 2.514 | 1.33 | 5.259 | 0.4771 | 0.6951 | 0.8807 | -0.1832 | -0.7246 |  |
| Xylt1 | xylosyltransferase 1 | 0.9223 | 0.9719 | 1.016 | 0.02224 | 0.09873 | 0.217 | 0.5782 | 1.266 | 0.3398 | 1.275 | 0.4225 | 0.6693 | 1.15 | 0.2021 | 0.8189 | 0.0001445 | 0.002328 | 0.4491 | -1.155 | -4.68 |  |
| Mir16-1 | microRNA 16-1 | 0.769 | 0.909 | 0.955 | -0.0664 | -0.2977 | 0.2045 | 0.5677 | 0.7867 | -0.3462 | -1.312 | 0.0002524 | 0.003519 | 2.121 | 1.085 | 4.44 | 0.3271 | 0.5668 | 0.8435 | -0.2455 | -1.005 |  |
| Gm13251 | NA | 0.9392 | 0.9787 | 0.9828 | -0.0251 | -0.07719 | 0.1101 | 0.4409 | 0.6402 | -0.6434 | -1.672 | 0.04017 | 0.1543 | 1.719 | 0.7819 | 2.195 | 0.006899 | 0.04289 | 2.104 | 1.073 | 3.012 |  |
| BC117090 | NA | 0.7052 | 0.8793 | 0.9499 | -0.07423 | -0.3837 | 0.2314 | 0.5929 | 1.216 | 0.2825 | 1.234 | 0.8253 | 0.9237 | 0.9677 | -0.0474 | -0.2237 | 0.0001027 | 0.001787 | 0.4921 | -1.023 | -4.827 |  |
| Gm6969 | predicted pseudogene 6969 | 0.9127 | 0.9685 | 1.018 | 0.02547 | 0.111 | 0.1367 | 0.4793 | 0.7469 | -0.421 | -1.551 | 0.0001805 | 0.002731 | 2.223 | 1.152 | 4.584 | 0.4652 | 0.686 | 1.139 | 0.1871 | 0.7445 |  |
| Tbc1d24 | TBC1 domain family, member 24 | 0.353 | 0.6497 | 0.8676 | -0.205 | -0.9509 | 0.8571 | 0.9526 | 1.033 | 0.04652 | 0.1824 | 0.08855 | 0.262 | 1.341 | 0.4228 | 1.79 | 7.128e-05 | 0.001375 | 0.4422 | -1.177 | -4.986 |  |
| Fbln5 | fibulin 5 | 0.07057 | 0.2835 | 1.382 | 0.4666 | 1.91 | 0.9036 | 0.9679 | 0.9757 | -0.03544 | -0.1226 | 0.0005071 | 0.006056 | 2.155 | 1.108 | 4.141 | 0.1524 | 0.3617 | 1.318 | 0.3981 | 1.488 |  |
| Aqp11 | aquaporin 11 | 0.1963 | 0.4897 | 0.7647 | -0.3871 | -1.337 | 0.5806 | 0.8293 | 1.143 | 0.1924 | 0.5617 | 0.000908 | 0.009406 | 0.425 | -1.234 | -3.892 | 0.1276 | 0.3228 | 0.7051 | -0.5042 | -1.59 |  |
| 1700011I03Rik | NA | 0.1501 | 0.4256 | 1.268 | 0.3422 | 1.497 | 0.7005 | 0.888 | 1.076 | 0.1055 | 0.3902 | 0.2364 | 0.4832 | 0.8091 | -0.3057 | -1.221 | 0.000619 | 0.007089 | 2.022 | 1.016 | 4.056 |  |
| H2-Eb1 | histocompatibility 2, class II | 0.1602 | 0.4398 | 1.599 | 0.6773 | 1.459 | 0.6404 | 0.8605 | 0.8347 | -0.2606 | -0.4743 | 0.01859 | 0.08947 | 2.468 | 1.303 | 2.562 | 0.007584 | 0.04587 | 2.849 | 1.51 | 2.97 |  |
| Arhgap42 | Rho GTPase activating protein | 0.07941 | 0.3006 | 1.315 | 0.3947 | 1.848 | 0.9908 | 0.9974 | 0.9979 | -0.002965 | -0.01173 | 2.559e-05 | 0.0006052 | 2.414 | 1.272 | 5.435 | 0.7409 | 0.8742 | 0.9471 | -0.07844 | -0.3353 |  |
| Olfr1034 | olfactory receptor 1034 | 0.778 | 0.9128 | 1.074 | 0.1033 | 0.2858 | 0.18 | 0.5405 | 0.6625 | -0.5941 | -1.39 | 0.003764 | 0.02767 | 2.458 | 1.297 | 3.278 | 0.05846 | 0.196 | 1.734 | 0.7944 | 2.007 |  |
| Gm15770 | predicted gene 15770 | 0.04646 | 0.2263 | 0.5815 | -0.7821 | -2.123 | 0.7025 | 0.889 | 0.8895 | -0.1689 | -0.3875 | 0.001431 | 0.01347 | 0.3556 | -1.492 | -3.697 | 0.3993 | 0.631 | 0.7859 | -0.3476 | -0.8614 |  |
| Scarna13 | small Cajal body-specific RNA | 0.388 | 0.6805 | 1.365 | 0.4487 | 0.8826 | 0.3755 | 0.7094 | 0.6853 | -0.5452 | -0.9064 | 0.00317 | 0.02437 | 3.649 | 1.867 | 3.353 | 0.05916 | 0.1977 | 2.165 | 1.114 | 2.001 |  |
| Pparg | peroxisome proliferator activa | 0.2334 | 0.5346 | 1.314 | 0.394 | 1.229 | 0.5277 | 0.8023 | 0.8445 | -0.2438 | -0.6427 | 0.001762 | 0.01568 | 2.406 | 1.267 | 3.607 | 0.08051 | 0.2425 | 1.565 | 0.6466 | 1.841 |  |
| Adamtsl1 | ADAMTS-like 1 | 0.08187 | 0.3056 | 1.269 | 0.3437 | 1.832 | 0.8924 | 0.9637 | 0.9791 | -0.03041 | -0.137 | 3.006e-05 | 0.0006842 | 2.147 | 1.102 | 5.364 | 0.9231 | 0.9657 | 1.014 | 0.02008 | 0.09774 |  |
| Cd274 | CD274 antigen | 0.08691 | 0.3168 | 0.7257 | -0.4626 | -1.8 | 0.8662 | 0.9549 | 1.037 | 0.0519 | 0.1707 | 0.000236 | 0.00336 | 0.4182 | -1.258 | -4.469 | 0.3221 | 0.5625 | 0.8203 | -0.2858 | -1.015 |  |
| G0s2 | G0/G1 switch gene 2 | 0.05738 | 0.254 | 0.3779 | -1.404 | -2.017 | 0.9959 | 0.9991 | 1.003 | 0.00425 | 0.005159 | 0.001368 | 0.01306 | 0.1402 | -2.834 | -3.716 | 0.08321 | 0.2476 | 0.3814 | -1.391 | -1.824 |  |
| Fancb | Fanconi anemia, complementatio | 0.9447 | 0.9806 | 1.01 | 0.01404 | 0.07025 | 0.3054 | 0.6594 | 0.8416 | -0.2488 | -1.052 | 0.2501 | 0.5001 | 0.8355 | -0.2594 | -1.185 | 0.000113 | 0.001926 | 2.067 | 1.048 | 4.786 |  |
| Kazald1 | Kazal-type serine peptidase in | 0.231 | 0.5319 | 0.8155 | -0.2942 | -1.236 | 0.6423 | 0.8609 | 1.096 | 0.1329 | 0.4716 | 0.8281 | 0.9249 | 1.041 | 0.05737 | 0.22 | 5.098e-05 | 0.001054 | 0.3954 | -1.339 | -5.132 |  |
| Ptgfr | prostaglandin F receptor | 0.6753 | 0.8655 | 1.112 | 0.1529 | 0.4251 | 0.2309 | 0.5922 | 0.6946 | -0.5258 | -1.236 | 0.0005906 | 0.006798 | 3.043 | 1.606 | 4.076 | 0.2874 | 0.5282 | 1.348 | 0.4305 | 1.093 |  |
| Psat1 | phosphoserine aminotransferase | 0.8884 | 0.9578 | 0.9692 | -0.04507 | -0.1422 | 0.1492 | 0.5 | 1.477 | 0.5627 | 1.5 | 0.001565 | 0.01444 | 0.4146 | -1.27 | -3.658 | 0.1271 | 0.322 | 0.6817 | -0.5529 | -1.592 |  |
| A730049H05Rik | RIKEN cDNA A730049H05 gene | 0.7012 | 0.8777 | 1.06 | 0.08362 | 0.3893 | 0.2289 | 0.5899 | 0.8036 | -0.3155 | -1.241 | 0.0001131 | 0.001896 | 2.183 | 1.126 | 4.785 | 0.7655 | 0.8876 | 1.051 | 0.07116 | 0.3024 |  |
| Mir30a | microRNA 30a | 0.09638 | 0.3349 | 1.402 | 0.4877 | 1.745 | 0.8425 | 0.9474 | 0.9549 | -0.06657 | -0.2013 | 0.0001277 | 0.002081 | 2.731 | 1.449 | 4.733 | 0.4934 | 0.707 | 1.16 | 0.2137 | 0.6978 |  |
| H2-DMa | histocompatibility 2, class II | 0.05042 | 0.236 | 1.573 | 0.6533 | 2.082 | 0.5151 | 0.7951 | 1.186 | 0.246 | 0.6627 | 0.6626 | 0.8372 | 1.111 | 0.1522 | 0.4429 | 0.005508 | 0.03628 | 2.099 | 1.069 | 3.111 |  |
| Tmem88b | transmembrane protein 88B | 0.7314 | 0.892 | 0.947 | -0.07851 | -0.3481 | 0.2343 | 0.595 | 1.255 | 0.3273 | 1.226 | 0.7788 | 0.8999 | 0.9524 | -0.07035 | -0.2847 | 0.0001574 | 0.002473 | 0.4515 | -1.147 | -4.643 |  |
| Amd-ps1 | S-adenosylmethionine decarboxy | 0.06861 | 0.2795 | 0.5949 | -0.7492 | -1.925 | 0.8682 | 0.9557 | 0.9477 | -0.07744 | -0.1682 | 0.7393 | 0.8799 | 0.9051 | -0.1439 | -0.3374 | 0.0002841 | 0.003939 | 0.2733 | -1.871 | -4.389 |  |
| Gnao1 | guanine nucleotide binding pro | 0.9251 | 0.9734 | 1.021 | 0.02944 | 0.09522 | 0.118 | 0.4537 | 1.513 | 0.5978 | 1.634 | 0.001622 | 0.01481 | 0.4252 | -1.234 | -3.643 | 0.1796 | 0.3996 | 0.7214 | -0.471 | -1.391 |  |
| Tmem252 | transmembrane protein 252 | 0.05606 | 0.2507 | 1.338 | 0.4197 | 2.028 | 0.7777 | 0.9198 | 1.05 | 0.07005 | 0.2861 | 0.0002145 | 0.003119 | 2.031 | 1.022 | 4.51 | 0.9069 | 0.9581 | 1.019 | 0.02683 | 0.1184 |  |
| Prrx1 | paired related homeobox 1 | 0.3573 | 0.6539 | 0.8514 | -0.2321 | -0.9422 | 0.4494 | 0.758 | 1.169 | 0.2249 | 0.7715 | 0.2857 | 0.5404 | 0.8145 | -0.296 | -1.097 | 0.0007328 | 0.008058 | 0.4747 | -1.075 | -3.983 |  |
| Lep | leptin | 0.4077 | 0.6957 | 0.5968 | -0.7447 | -0.8457 | 0.3748 | 0.709 | 1.926 | 0.9459 | 0.9079 | 0.003896 | 0.02842 | 0.1128 | -3.148 | -3.263 | 0.0599 | 0.1992 | 0.2635 | -1.924 | -1.995 |  |
| LOC105242520 | NA | 0.3757 | 0.6699 | 0.7786 | -0.361 | -0.9061 | 0.1392 | 0.4837 | 0.6045 | -0.7261 | -1.54 | 0.004927 | 0.03369 | 2.601 | 1.379 | 3.16 | 0.5228 | 0.73 | 0.8214 | -0.2839 | -0.6505 |  |
| Apbb1ip | amyloid beta (A4) precursor pr | 0.07245 | 0.2876 | 1.517 | 0.6017 | 1.897 | 0.9155 | 0.972 | 0.9724 | -0.04032 | -0.1074 | 0.005032 | 0.03422 | 2.136 | 1.095 | 3.151 | 0.02677 | 0.1144 | 1.779 | 0.8309 | 2.391 |  |
| Esyt1 | extended synaptotagmin-like pr | 0.1156 | 0.3699 | 0.7961 | -0.329 | -1.645 | 0.8224 | 0.9393 | 1.038 | 0.05381 | 0.2274 | 3.648e-05 | 0.0007886 | 0.4486 | -1.156 | -5.279 | 0.9709 | 0.9867 | 1.006 | 0.00808 | 0.03688 |  |
| Apoc3 | apolipoprotein C-III | 0.2582 | 0.563 | 1.393 | 0.4783 | 1.164 | 0.5332 | 0.8042 | 0.8076 | -0.3084 | -0.6342 | 0.0001359 | 0.002177 | 4.343 | 2.119 | 4.706 | 0.5976 | 0.7829 | 1.182 | 0.2415 | 0.5364 |  |
| Zscan21 | zinc finger and SCAN domain co | 0.3411 | 0.6401 | 1.135 | 0.1833 | 0.9752 | 0.5575 | 0.8176 | 0.9121 | -0.1327 | -0.5966 | 0.7417 | 0.8808 | 0.9534 | -0.06883 | -0.3343 | 7.949e-05 | 0.001472 | 2.023 | 1.017 | 4.938 |  |
| A530053G22Rik | RIKEN cDNA A530053G22 gene | 0.9442 | 0.9803 | 1.021 | 0.03047 | 0.07087 | 0.1221 | 0.4601 | 1.767 | 0.8211 | 1.614 | 0.004132 | 0.02968 | 0.3476 | -1.525 | -3.237 | 0.08702 | 0.2547 | 0.5557 | -0.8475 | -1.8 |  |
| Gm23344 | predicted gene, 23344 | 0.2769 | 0.5826 | 1.292 | 0.3697 | 1.118 | 0.502 | 0.7882 | 0.8307 | -0.2675 | -0.6837 | 0.003469 | 0.02599 | 2.298 | 1.201 | 3.314 | 0.06374 | 0.2078 | 1.637 | 0.7111 | 1.963 |  |
| Csad | cysteine sulfinic acid decarbo | 0.3706 | 0.6654 | 1.152 | 0.2035 | 0.9159 | 0.4219 | 0.7418 | 0.8612 | -0.2156 | -0.82 | 0.0003883 | 0.004944 | 2.05 | 1.036 | 4.255 | 0.3735 | 0.608 | 1.166 | 0.2216 | 0.9103 |  |
| Mug4-ps | murinoglobulin 4, pseudogene | 0.4216 | 0.7066 | 1.362 | 0.4455 | 0.8205 | 0.4206 | 0.741 | 0.6934 | -0.5283 | -0.8224 | 0.3265 | 0.5816 | 1.514 | 0.5983 | 1.006 | 0.0008781 | 0.009219 | 5.004 | 2.323 | 3.906 |  |
| Ak4 | adenylate kinase 4 | 0.2513 | 0.5551 | 1.184 | 0.2441 | 1.181 | 0.758 | 0.9118 | 0.9484 | -0.07636 | -0.3124 | 6.323e-05 | 0.001197 | 2.204 | 1.14 | 5.038 | 0.4387 | 0.664 | 0.8834 | -0.1788 | -0.7901 |  |
| Sav1 | salvador family WW domain cont | 0.253 | 0.5569 | 1.183 | 0.2425 | 1.177 | 0.8685 | 0.9558 | 1.029 | 0.04089 | 0.1677 | 0.0002494 | 0.003489 | 2.004 | 1.003 | 4.445 | 0.09289 | 0.265 | 0.7587 | -0.3983 | -1.765 |  |
| Tfcp2l1 | transcription factor CP2-like | 0.1312 | 0.3971 | 1.276 | 0.3511 | 1.574 | 0.8399 | 0.9464 | 0.9633 | -0.054 | -0.2046 | 0.9122 | 0.9642 | 1.019 | 0.02727 | 0.1116 | 9.472e-05 | 0.001691 | 2.278 | 1.188 | 4.862 |  |
| Ccbl2 | NA | 0.2023 | 0.4974 | 1.327 | 0.4082 | 1.318 | 0.6353 | 0.8581 | 0.8849 | -0.1764 | -0.4816 | 0.000291 | 0.003936 | 2.8 | 1.485 | 4.379 | 0.4375 | 0.6629 | 1.205 | 0.2687 | 0.7923 |  |
| Gbp8 | guanylate-binding protein 8 | 0.08348 | 0.309 | 1.479 | 0.5642 | 1.822 | 0.9594 | 0.987 | 0.987 | -0.0189 | -0.05157 | 0.1173 | 0.3135 | 1.469 | 0.5553 | 1.637 | 0.002157 | 0.01819 | 2.288 | 1.194 | 3.52 |  |
| Hist1h3e | NA | 0.7198 | 0.8864 | 1.056 | 0.07927 | 0.3638 | 0.3334 | 0.6796 | 0.8377 | -0.2555 | -0.9913 | 0.6774 | 0.8454 | 0.9325 | -0.1008 | -0.4222 | 0.0001618 | 0.002523 | 2.151 | 1.105 | 4.631 |  |
| Itm2a | integral membrane protein 2A | 0.1245 | 0.3852 | 0.7761 | -0.3657 | -1.604 | 0.8645 | 0.9543 | 1.033 | 0.04665 | 0.1729 | 0.3735 | 0.6252 | 0.8542 | -0.2274 | -0.9103 | 0.0006396 | 0.007264 | 0.4966 | -1.01 | -4.042 |  |
| Serpina3b | serine (or cysteine) peptidase | 0.5489 | 0.7936 | 0.8965 | -0.1577 | -0.6097 | 0.3239 | 0.672 | 1.239 | 0.3095 | 1.011 | 0.0002812 | 0.003834 | 0.4221 | -1.244 | -4.393 | 0.5857 | 0.7749 | 0.8969 | -0.1569 | -0.554 |  |
| Uhrf1bp1 | UHRF1 (ICBP90) binding protein | 0.4793 | 0.7481 | 0.8694 | -0.2018 | -0.7209 | 0.1113 | 0.4431 | 0.6821 | -0.5519 | -1.666 | 0.003645 | 0.02703 | 2.014 | 1.01 | 3.292 | 0.8964 | 0.9533 | 1.028 | 0.04045 | 0.1319 |  |
| 4930533I22Rik | RIKEN cDNA 4930533I22 gene | 0.57 | 0.8068 | 1.213 | 0.279 | 0.5776 | 0.3075 | 0.6602 | 0.6604 | -0.5985 | -1.047 | 0.005256 | 0.03534 | 3.154 | 1.657 | 3.132 | 0.08014 | 0.2417 | 1.966 | 0.9754 | 1.844 |  |
| Pltp | phospholipid transfer protein | 0.9655 | 0.9881 | 1.009 | 0.01229 | 0.04381 | 0.1509 | 0.5032 | 0.7091 | -0.4959 | -1.493 | 0.00363 | 0.02697 | 2.017 | 1.013 | 3.294 | 0.1115 | 0.2976 | 1.426 | 0.5119 | 1.665 |  |
| Samd10 | sterile alpha motif domain con | 0.9332 | 0.9761 | 0.9838 | -0.0236 | -0.08485 | 0.1626 | 0.518 | 0.7184 | -0.4772 | -1.45 | 0.529 | 0.752 | 1.145 | 0.1952 | 0.6407 | 0.00106 | 0.01062 | 2.243 | 1.166 | 3.825 |  |
| Yipf5 | Yip1 domain family, member 5 | 0.3159 | 0.6173 | 0.8434 | -0.2457 | -1.029 | 0.5035 | 0.7888 | 1.143 | 0.1925 | 0.6813 | 0.0003606 | 0.004657 | 0.4596 | -1.121 | -4.287 | 0.469 | 0.6884 | 0.8747 | -0.1931 | -0.7382 |  |
| Atp1b4 | ATPase, (Na+)/K+ transporting, | 0.8574 | 0.9466 | 0.9705 | -0.04313 | -0.182 | 0.2078 | 0.57 | 0.7764 | -0.365 | -1.302 | 0.7172 | 0.8682 | 0.936 | -0.09539 | -0.3674 | 0.0005747 | 0.00671 | 2.087 | 1.061 | 4.087 |  |
| Adap1 | ArfGAP with dual PH domains 1 | 0.4463 | 0.7252 | 0.8872 | -0.1726 | -0.777 | 0.6716 | 0.8751 | 1.082 | 0.1131 | 0.4302 | 0.0001208 | 0.002 | 0.4482 | -1.158 | -4.757 | 0.2311 | 0.4634 | 1.232 | 0.3006 | 1.235 |  |
| Trp53i11 | transformation related protein | 0.5069 | 0.7653 | 1.176 | 0.2337 | 0.6758 | 0.1205 | 0.4569 | 1.584 | 0.6637 | 1.622 | 0.003417 | 0.02569 | 0.4181 | -1.258 | -3.32 | 0.9338 | 0.9702 | 0.9782 | -0.03186 | -0.08408 |  |
| Lonrf1 | LON peptidase N-terminal domai | 0.7271 | 0.8897 | 0.9355 | -0.09618 | -0.3539 | 0.1902 | 0.551 | 0.7391 | -0.4361 | -1.356 | 0.7459 | 0.8824 | 0.9344 | -0.09782 | -0.3286 | 0.001252 | 0.01204 | 2.17 | 1.118 | 3.754 |  |
| Ehd4 | EH-domain containing 4 | 0.08767 | 0.3184 | 1.367 | 0.451 | 1.796 | 0.904 | 0.9681 | 1.025 | 0.03628 | 0.1221 | 0.82 | 0.921 | 1.045 | 0.06345 | 0.2306 | 0.0003565 | 0.004679 | 2.267 | 1.181 | 4.292 |  |
| 1700113H08Rik | RIKEN cDNA 1700113H08 gene | 0.1895 | 0.481 | 0.8042 | -0.3143 | -1.358 | 0.7767 | 0.9195 | 1.056 | 0.07871 | 0.2875 | 0.7595 | 0.8895 | 0.9469 | -0.07868 | -0.3104 | 0.0003174 | 0.004293 | 0.4664 | -1.1 | -4.341 |  |
| Olfr1033 | olfactory receptor 1033 | 0.8889 | 0.958 | 0.9669 | -0.04859 | -0.1414 | 0.1532 | 0.5064 | 0.6581 | -0.6036 | -1.485 | 0.0007992 | 0.008544 | 2.799 | 1.485 | 3.946 | 0.6376 | 0.8092 | 1.133 | 0.18 | 0.4784 |  |
| Slpi | secretory leukocyte peptidase | 0.7357 | 0.8935 | 0.9194 | -0.1212 | -0.3423 | 0.1346 | 0.4762 | 0.6358 | -0.6533 | -1.56 | 0.008384 | 0.04991 | 2.195 | 1.134 | 2.925 | 0.2475 | 0.4825 | 1.377 | 0.462 | 1.191 |  |
| Eml1 | echinoderm microtubule associa | 0.3082 | 0.6109 | 0.8489 | -0.2363 | -1.046 | 0.6158 | 0.8484 | 1.099 | 0.1363 | 0.5098 | 0.917 | 0.9662 | 1.018 | 0.02614 | 0.1056 | 0.0001654 | 0.00257 | 0.4525 | -1.144 | -4.621 |  |
| Garem | NA | 0.2834 | 0.5881 | 1.166 | 0.2221 | 1.102 | 0.7521 | 0.9091 | 0.9485 | -0.07633 | -0.3202 | 0.6375 | 0.8207 | 0.9294 | -0.1056 | -0.4785 | 0.0002023 | 0.003011 | 2.001 | 1.001 | 4.535 |  |
| Pde7a | phosphodiesterase 7A | 0.7711 | 0.9095 | 1.046 | 0.0653 | 0.295 | 0.4058 | 0.7304 | 0.8571 | -0.2225 | -0.8493 | 0.4763 | 0.7117 | 0.8851 | -0.1761 | -0.7259 | 0.0003617 | 0.004725 | 2.055 | 1.039 | 4.286 |  |
| Slc4a11 | solute carrier family 4, sodiu | 0.3572 | 0.6538 | 1.154 | 0.207 | 0.9426 | 0.5078 | 0.7912 | 0.8856 | -0.1752 | -0.6744 | 0.0002173 | 0.003141 | 2.119 | 1.083 | 4.504 | 0.7956 | 0.9039 | 1.045 | 0.06316 | 0.2626 |  |
| Gm2005 | predicted pseudogene 2005 | 0.7736 | 0.9105 | 0.9151 | -0.128 | -0.2916 | 0.1602 | 0.5149 | 0.5913 | -0.758 | -1.459 | 0.3415 | 0.5971 | 1.384 | 0.4688 | 0.9745 | 0.00785 | 0.04708 | 2.678 | 1.421 | 2.954 |  |
| Hist1h3b | NA | 0.8736 | 0.9527 | 1.025 | 0.0362 | 0.1612 | 0.3146 | 0.6646 | 0.8269 | -0.2742 | -1.031 | 0.6407 | 0.8223 | 0.9223 | -0.1166 | -0.4739 | 0.000386 | 0.004965 | 2.067 | 1.048 | 4.258 |  |
| Map3k19 | mitogen-activated protein kina | 0.08445 | 0.3112 | 1.706 | 0.7709 | 1.816 | 0.9806 | 0.9944 | 1.009 | 0.01235 | 0.02458 | 0.001704 | 0.01537 | 3.214 | 1.684 | 3.621 | 0.2181 | 0.448 | 1.507 | 0.5914 | 1.272 |  |
| Hist1h3g | NA | 0.9561 | 0.9845 | 0.9913 | -0.0126 | -0.0558 | 0.25 | 0.6097 | 0.803 | -0.3165 | -1.185 | 0.6501 | 0.8286 | 0.9241 | -0.1139 | -0.4605 | 0.0005006 | 0.006031 | 2.036 | 1.026 | 4.146 |  |
| Pydc3 | NA | 0.08611 | 0.3152 | 1.946 | 0.9602 | 1.805 | 0.8173 | 0.937 | 1.107 | 0.1473 | 0.234 | 0.2539 | 0.5042 | 1.607 | 0.6844 | 1.175 | 0.006908 | 0.04293 | 3.373 | 1.754 | 3.011 |  |
| Cd28 | CD28 antigen | 0.4841 | 0.7509 | 0.8401 | -0.2514 | -0.713 | 0.5596 | 0.8185 | 1.187 | 0.2475 | 0.5933 | 0.6569 | 0.8337 | 1.128 | 0.1742 | 0.4509 | 0.0003291 | 0.004412 | 0.314 | -1.671 | -4.326 |  |
| Gm13768 | predicted gene 13768 | 0.07919 | 0.3004 | 1.532 | 0.6157 | 1.85 | 0.5837 | 0.8306 | 1.164 | 0.2194 | 0.5571 | 0.8383 | 0.9303 | 1.054 | 0.07539 | 0.2068 | 0.006271 | 0.03985 | 2.164 | 1.113 | 3.054 |  |
| Nr4a3 | nuclear receptor subfamily 4, | 0.3109 | 0.6133 | 1.328 | 0.4094 | 1.04 | 0.5587 | 0.8181 | 0.8253 | -0.2771 | -0.5947 | 0.001917 | 0.01674 | 2.908 | 1.54 | 3.571 | 0.2428 | 0.4768 | 1.433 | 0.5192 | 1.204 |  |
| Glis3 | GLIS family zinc finger 3 | 0.9755 | 0.9913 | 0.9953 | -0.00679 | -0.03111 | 0.3511 | 0.6928 | 1.186 | 0.2466 | 0.9548 | 0.0003067 | 0.004105 | 0.4858 | -1.042 | -4.356 | 0.253 | 0.4883 | 1.215 | 0.2814 | 1.177 |  |
| Pik3r3 | phosphoinositide-3-kinase regu | 0.7355 | 0.8934 | 1.051 | 0.07151 | 0.3426 | 0.5656 | 0.8211 | 0.9048 | -0.1443 | -0.5842 | 0.0002803 | 0.003823 | 2.007 | 1.005 | 4.395 | 0.1336 | 0.3324 | 0.7805 | -0.3575 | -1.563 |  |
| Gm6155 | predicted pseudogene 6155 | 0.7397 | 0.8959 | 1.096 | 0.1317 | 0.3369 | 0.1714 | 0.5297 | 1.576 | 0.6565 | 1.419 | 0.5153 | 0.7416 | 0.8214 | -0.2838 | -0.6624 | 0.00689 | 0.04286 | 0.4088 | -1.29 | -3.012 |  |
| Hist1h3h | NA | 0.9003 | 0.9628 | 1.019 | 0.02783 | 0.1269 | 0.2905 | 0.6461 | 0.8226 | -0.2817 | -1.086 | 0.7471 | 0.8831 | 0.947 | -0.07852 | -0.3269 | 0.0004492 | 0.005558 | 2.01 | 1.007 | 4.193 |  |
| Gm12864 | predicted gene 12864 | 0.9766 | 0.9917 | 1.006 | 0.008 | 0.02965 | 0.1897 | 0.5507 | 0.7405 | -0.4334 | -1.358 | 0.0004557 | 0.005592 | 2.358 | 1.237 | 4.187 | 0.7719 | 0.8913 | 1.062 | 0.08684 | 0.2938 |  |
| Hist1h3c | NA | 0.9981 | 0.9992 | 0.9996 | -0.0005333 | -0.002374 | 0.2833 | 0.6385 | 0.8162 | -0.293 | -1.103 | 0.5626 | 0.775 | 0.9044 | -0.1449 | -0.5888 | 0.0004907 | 0.005938 | 2.031 | 1.022 | 4.155 |  |
| Gstt1 | glutathione S-transferase, the | 0.2708 | 0.5762 | 0.832 | -0.2653 | -1.133 | 0.6256 | 0.8532 | 1.1 | 0.1374 | 0.4956 | 0.0007641 | 0.008247 | 0.4939 | -1.018 | -3.966 | 0.4668 | 0.6871 | 0.8764 | -0.1904 | -0.7419 |  |
| Raet1e | retinoic acid early transcript | 0.2815 | 0.5863 | 1.257 | 0.3296 | 1.107 | 0.6037 | 0.8421 | 0.8791 | -0.1858 | -0.5274 | 0.004692 | 0.0325 | 2.053 | 1.038 | 3.182 | 0.1348 | 0.3341 | 1.423 | 0.5084 | 1.559 |  |
| Ifit3 | interferon-induced protein wit | 0.2374 | 0.5388 | 0.7844 | -0.3504 | -1.218 | 0.6888 | 0.8835 | 1.101 | 0.1384 | 0.4064 | 0.0002357 | 0.003357 | 0.3767 | -1.409 | -4.469 | 0.8563 | 0.9342 | 0.9607 | -0.0578 | -0.1834 |  |
| Agbl1 | ATP/GTP binding protein-like 1 | 0.7905 | 0.9182 | 0.9202 | -0.1199 | -0.2692 | 0.2923 | 0.6483 | 1.485 | 0.5702 | 1.082 | 0.8936 | 0.9551 | 0.9552 | -0.06608 | -0.1354 | 0.0005491 | 0.006479 | 0.2493 | -2.004 | -4.107 |  |
| A530016L24Rik | RIKEN cDNA A530016L24 gene | 0.995 | 0.9983 | 1.002 | 0.003203 | 0.006336 | 0.1769 | 0.5374 | 1.787 | 0.8374 | 1.4 | 0.00582 | 0.03816 | 0.3057 | -1.71 | -3.087 | 0.1676 | 0.3834 | 0.5771 | -0.7931 | -1.432 |  |
| Bmp3 | bone morphogenetic protein 3 | 0.09 | 0.3232 | 0.6369 | -0.6507 | -1.782 | 0.8182 | 0.9373 | 0.9326 | -0.1007 | -0.2329 | 0.004579 | 0.03192 | 0.4126 | -1.277 | -3.192 | 0.3624 | 0.5986 | 0.7722 | -0.373 | -0.9322 |  |
| Tnc | tenascin C | 0.7954 | 0.9202 | 1.059 | 0.0832 | 0.2627 | 0.3659 | 0.7036 | 1.272 | 0.3466 | 0.9251 | 0.001685 | 0.01527 | 0.4182 | -1.258 | -3.626 | 0.1928 | 0.4165 | 1.383 | 0.4676 | 1.348 |  |
| Hist1h3i | NA | 0.6367 | 0.8448 | 1.079 | 0.1094 | 0.4796 | 0.4407 | 0.7531 | 0.8631 | -0.2124 | -0.7866 | 0.7712 | 0.8958 | 0.9502 | -0.0737 | -0.2948 | 0.0004881 | 0.005919 | 2.055 | 1.039 | 4.157 |  |
| Methig1 | methyltransferase hypoxia indu | 0.1362 | 0.4056 | 1.37 | 0.4537 | 1.553 | 0.9227 | 0.9746 | 0.9767 | -0.03397 | -0.09824 | 0.227 | 0.4714 | 1.319 | 0.3991 | 1.247 | 0.003392 | 0.0255 | 2.091 | 1.064 | 3.324 |  |
| 4930467E23Rik | RIKEN cDNA 4930467E23 gene | 0.1296 | 0.3942 | 1.45 | 0.5362 | 1.581 | 0.9077 | 0.9692 | 0.9679 | -0.0471 | -0.1174 | 0.003197 | 0.02452 | 2.369 | 1.244 | 3.349 | 0.1887 | 0.4108 | 1.42 | 0.5056 | 1.361 |  |
| Jun | jun proto-oncogene | 0.4406 | 0.7211 | 1.231 | 0.2995 | 0.7868 | 0.5254 | 0.8013 | 0.8172 | -0.2912 | -0.6464 | 0.6144 | 0.8066 | 1.159 | 0.2134 | 0.5118 | 0.001301 | 0.01234 | 2.946 | 1.559 | 3.737 |  |
| Mia2 | MIA SH3 domain ER export facto | 0.8154 | 0.9303 | 0.9548 | -0.0667 | -0.2365 | 0.2653 | 0.6228 | 1.304 | 0.3824 | 1.146 | 0.002647 | 0.02137 | 0.4796 | -1.06 | -3.431 | 0.3633 | 0.5993 | 0.8194 | -0.2874 | -0.9304 |  |
| Klhl29 | kelch-like 29 | 0.1309 | 0.3967 | 0.7649 | -0.3866 | -1.575 | 0.9952 | 0.999 | 1.001 | 0.001773 | 0.006104 | 0.8592 | 0.9402 | 0.9671 | -0.0483 | -0.1796 | 0.0004918 | 0.005948 | 0.4611 | -1.117 | -4.154 |  |
| Dgkb | diacylglycerol kinase, beta | 0.7351 | 0.8932 | 1.1 | 0.1378 | 0.3431 | 0.1835 | 0.5443 | 1.574 | 0.6545 | 1.378 | 0.005268 | 0.03539 | 0.3851 | -1.377 | -3.131 | 0.6978 | 0.8487 | 0.8868 | -0.1732 | -0.394 |  |
| Cd209d | CD209d antigen | 0.7545 | 0.902 | 1.075 | 0.1043 | 0.317 | 0.1806 | 0.5411 | 1.454 | 0.5402 | 1.387 | 0.007803 | 0.04734 | 0.4777 | -1.066 | -2.957 | 0.5102 | 0.7202 | 0.8457 | -0.2417 | -0.6705 |  |
| Traj18 | T cell receptor alpha joining | 0.1221 | 0.3818 | 2.454 | 1.295 | 1.615 | 0.8612 | 0.9534 | 1.124 | 0.1681 | 0.1771 | 0.5228 | 0.7471 | 1.486 | 0.5715 | 0.6504 | 0.00453 | 0.03148 | 7.01 | 2.809 | 3.197 |  |
| Slc39a8 | solute carrier family 39 (meta | 0.6597 | 0.8569 | 1.109 | 0.1492 | 0.4469 | 0.4025 | 0.7289 | 0.7912 | -0.3378 | -0.8554 | 0.0004168 | 0.005199 | 2.918 | 1.545 | 4.225 | 0.8212 | 0.9168 | 0.9436 | -0.08371 | -0.2289 |  |
| Ccdc3 | coiled-coil domain containing | 0.4584 | 0.7336 | 0.8198 | -0.2866 | -0.7562 | 0.5388 | 0.8069 | 1.215 | 0.2805 | 0.6254 | 0.0003505 | 0.004546 | 0.2901 | -1.785 | -4.299 | 0.8422 | 0.928 | 1.06 | 0.08376 | 0.2017 |  |
| Pde4d | phosphodiesterase 4D, cAMP spe | 0.256 | 0.5603 | 1.231 | 0.2993 | 1.169 | 0.7085 | 0.892 | 0.9235 | -0.1149 | -0.3793 | 0.0005404 | 0.006341 | 2.224 | 1.153 | 4.114 | 0.754 | 0.8822 | 1.064 | 0.08906 | 0.3177 |  |
| Ier3 | immediate early response 3 | 0.2473 | 0.5509 | 1.269 | 0.3432 | 1.192 | 0.8192 | 0.9378 | 0.9467 | -0.07895 | -0.2317 | 0.8885 | 0.953 | 0.9694 | -0.0448 | -0.142 | 0.0004891 | 0.005926 | 2.482 | 1.311 | 4.156 |  |
| Mir99a | microRNA 99a | 0.9503 | 0.9823 | 0.9896 | -0.01509 | -0.06317 | 0.209 | 0.5713 | 0.7754 | -0.367 | -1.298 | 0.0007117 | 0.007783 | 2.064 | 1.046 | 3.996 | 0.9699 | 0.9862 | 1.007 | 0.009996 | 0.0382 |  |
| Mob3a | MOB kinase activator 3A | 0.2207 | 0.5201 | 1.28 | 0.3562 | 1.264 | 0.9791 | 0.9938 | 0.9939 | -0.008855 | -0.02656 | 0.5895 | 0.7926 | 0.8893 | -0.1693 | -0.5484 | 0.0005619 | 0.00659 | 2.402 | 1.264 | 4.097 |  |
| Ptger3 | prostaglandin E receptor 3 (su | 0.1206 | 0.3788 | 0.651 | -0.6193 | -1.621 | 0.9869 | 0.9961 | 0.9948 | -0.007534 | -0.01667 | 0.000351 | 0.004551 | 0.2875 | -1.798 | -4.298 | 0.9311 | 0.9693 | 0.9749 | -0.03666 | -0.08762 |  |
| Flnc | filamin C, gamma | 0.2474 | 0.5509 | 1.205 | 0.2696 | 1.191 | 0.9111 | 0.9702 | 0.9792 | -0.03027 | -0.1131 | 0.6566 | 0.8337 | 0.9254 | -0.1119 | -0.4514 | 0.0005836 | 0.006796 | 2.016 | 1.012 | 4.081 |  |
| B4galnt2 | beta-1,4-N-acetyl-galactosamin | 0.4949 | 0.7574 | 0.8679 | -0.2045 | -0.6953 | 0.4675 | 0.7685 | 1.196 | 0.2578 | 0.7408 | 0.005395 | 0.03608 | 0.4982 | -1.005 | -3.12 | 0.2502 | 0.4853 | 0.7676 | -0.3815 | -1.184 |  |
| Scd3 | stearoyl-coenzyme A desaturase | 0.5653 | 0.8039 | 1.154 | 0.2068 | 0.5847 | 0.4359 | 0.7503 | 0.794 | -0.3327 | -0.795 | 0.004606 | 0.03206 | 2.355 | 1.236 | 3.19 | 0.3352 | 0.5737 | 1.304 | 0.3827 | 0.9876 |  |
| Egr2 | early growth response 2 | 0.6683 | 0.8616 | 0.9339 | -0.09863 | -0.4349 | 0.535 | 0.8051 | 1.125 | 0.1694 | 0.6313 | 0.0006586 | 0.007381 | 0.4997 | -1.001 | -4.029 | 0.4375 | 0.6629 | 1.146 | 0.1968 | 0.7923 |  |
| Zfp454 | zinc finger protein 454 | 0.1615 | 0.4413 | 0.7445 | -0.4256 | -1.454 | 0.8508 | 0.9504 | 0.9553 | -0.066 | -0.1905 | 0.8264 | 0.9243 | 1.051 | 0.07126 | 0.2222 | 0.001848 | 0.01615 | 0.4506 | -1.15 | -3.587 |  |
| Tspan5 | tetraspanin 5 | 0.7874 | 0.917 | 0.9492 | -0.07529 | -0.2733 | 0.4809 | 0.7756 | 1.176 | 0.2341 | 0.7182 | 0.0007453 | 0.008072 | 0.4354 | -1.2 | -3.976 | 0.3787 | 0.6128 | 1.207 | 0.2716 | 0.9002 |  |
| Smco4 | single-pass membrane protein w | 0.2068 | 0.5028 | 0.7711 | -0.375 | -1.305 | 0.8709 | 0.9564 | 1.04 | 0.05596 | 0.1646 | 0.516 | 0.7419 | 0.8656 | -0.2082 | -0.6613 | 0.002564 | 0.02067 | 0.4715 | -1.085 | -3.445 |  |
| Pygb | brain glycogen phosphorylase | 0.1632 | 0.4442 | 0.689 | -0.5374 | -1.448 | 0.9112 | 0.9702 | 1.035 | 0.04961 | 0.113 | 0.001697 | 0.01534 | 0.3601 | -1.473 | -3.623 | 0.4873 | 0.7021 | 0.8192 | -0.2878 | -0.7077 |  |
| Peg10 | paternally expressed 10 | 0.1833 | 0.4729 | 1.474 | 0.5596 | 1.378 | 0.8932 | 0.964 | 0.9557 | -0.06531 | -0.136 | 0.0004015 | 0.00505 | 3.696 | 1.886 | 4.241 | 0.9799 | 0.9907 | 0.9922 | -0.01136 | -0.02555 |  |
| Rmrp | RNA component of mitochondrial | 0.677 | 0.8663 | 1.111 | 0.1518 | 0.4227 | 0.3932 | 0.7223 | 0.7733 | -0.3708 | -0.8726 | 0.006588 | 0.04185 | 2.286 | 1.193 | 3.032 | 0.3518 | 0.5896 | 1.297 | 0.3751 | 0.9533 |  |
| BC026585 | NA | 0.8806 | 0.9555 | 0.9715 | -0.04178 | -0.1521 | 0.3023 | 0.6561 | 1.269 | 0.344 | 1.059 | 0.00318 | 0.02443 | 0.4972 | -1.008 | -3.352 | 0.6254 | 0.8014 | 0.9018 | -0.1492 | -0.4958 |  |
| Cldn15 | claudin 15 | 0.1349 | 0.4034 | 1.381 | 0.4655 | 1.558 | 0.8512 | 0.9504 | 1.048 | 0.06721 | 0.1901 | 0.005865 | 0.03839 | 2.013 | 1.009 | 3.084 | 0.5769 | 0.7685 | 1.137 | 0.1856 | 0.5671 |  |
| 4931406H21Rik | NA | 0.1477 | 0.4223 | 1.34 | 0.4227 | 1.506 | 0.9599 | 0.9871 | 1.012 | 0.01689 | 0.05085 | 0.001794 | 0.01591 | 2.153 | 1.107 | 3.599 | 0.722 | 0.8637 | 1.08 | 0.1109 | 0.3608 |  |
| Angptl7 | angiopoietin-like 7 | 0.2824 | 0.5872 | 1.392 | 0.4768 | 1.105 | 0.8333 | 0.9438 | 0.9273 | -0.1089 | -0.2133 | 0.9454 | 0.9787 | 0.9775 | -0.03276 | -0.06929 | 0.001163 | 0.01139 | 3.458 | 1.79 | 3.786 |  |
| Pln | phospholamban | 0.7686 | 0.9087 | 1.1 | 0.1372 | 0.2983 | 0.2631 | 0.6206 | 1.544 | 0.627 | 1.152 | 0.005558 | 0.03687 | 0.3377 | -1.566 | -3.107 | 0.8492 | 0.9309 | 1.07 | 0.09708 | 0.1926 |  |
| Sowahc | sosondowah ankyrin repeat doma | 0.6859 | 0.8714 | 1.101 | 0.1389 | 0.4104 | 0.4221 | 0.7419 | 0.7965 | -0.3283 | -0.8196 | 0.004511 | 0.03161 | 2.276 | 1.186 | 3.199 | 0.5681 | 0.7621 | 1.161 | 0.2153 | 0.5805 |  |
| Kcnk1 | potassium channel, subfamily K | 0.9899 | 0.9969 | 1.003 | 0.0039 | 0.0128 | 0.9205 | 0.9739 | 1.026 | 0.03645 | 0.1011 | 0.003586 | 0.02669 | 0.466 | -1.102 | -3.299 | 0.006974 | 0.04326 | 2.005 | 1.004 | 3.007 |  |
| Zbed3 | zinc finger, BED type containi | 0.5195 | 0.7747 | 0.8759 | -0.1911 | -0.6557 | 0.5828 | 0.8301 | 1.143 | 0.1926 | 0.5584 | 0.8793 | 0.9497 | 0.9665 | -0.04912 | -0.1538 | 0.002527 | 0.02046 | 0.4659 | -1.102 | -3.451 |  |
| Cidea | cell death-inducing DNA fragme | 0.8572 | 0.9466 | 0.9666 | -0.04902 | -0.1822 | 0.7939 | 0.927 | 1.06 | 0.08428 | 0.2648 | 0.002885 | 0.02273 | 0.5 | -1 | -3.394 | 0.05515 | 0.1882 | 1.516 | 0.6001 | 2.037 |  |
| Calml3 | calmodulin-like 3 | 0.1711 | 0.4552 | 0.7286 | -0.4568 | -1.42 | 0.9822 | 0.9949 | 0.9941 | -0.00859 | -0.02256 | 0.002977 | 0.02332 | 0.4378 | -1.192 | -3.38 | 0.6687 | 0.8301 | 0.8993 | -0.1531 | -0.4343 |  |
| Gm39875 | NA | 0.8152 | 0.9302 | 0.9487 | -0.07595 | -0.2368 | 0.4375 | 0.7509 | 1.232 | 0.3007 | 0.7923 | 0.8349 | 0.9286 | 1.053 | 0.07419 | 0.2111 | 0.002674 | 0.0213 | 0.434 | -1.204 | -3.427 |  |
| Dao | D-amino acid oxidase | 0.7948 | 0.9199 | 0.9329 | -0.1002 | -0.2635 | 0.3766 | 0.7106 | 1.326 | 0.407 | 0.9043 | 0.006276 | 0.04041 | 0.414 | -1.272 | -3.054 | 0.5564 | 0.7541 | 0.8413 | -0.2493 | -0.5982 |  |
| H2-DMb1 | histocompatibility 2, class II | 0.2864 | 0.591 | 1.422 | 0.508 | 1.095 | 0.9567 | 0.9863 | 1.021 | 0.03017 | 0.05499 | 0.613 | 0.8056 | 0.8345 | -0.2611 | -0.5139 | 0.003299 | 0.02494 | 3.236 | 1.694 | 3.336 |  |
| Slc30a2 | solute carrier family 30 (zinc | 0.9925 | 0.9976 | 1.002 | 0.003463 | 0.009577 | 0.5154 | 0.7953 | 0.8217 | -0.2834 | -0.6622 | 0.342 | 0.5974 | 0.7654 | -0.3856 | -0.9734 | 0.005095 | 0.03426 | 2.372 | 1.246 | 3.146 |  |
| Gm28645 | predicted gene 28645 | 0.734 | 0.8929 | 0.9173 | -0.1245 | -0.3445 | 0.44 | 0.7527 | 1.263 | 0.3369 | 0.7879 | 0.007155 | 0.04444 | 0.4395 | -1.186 | -2.995 | 0.6218 | 0.7994 | 0.8715 | -0.1984 | -0.5011 |  |
| mt-Ty | mitochondrially encoded tRNA t | 0.4461 | 0.725 | 1.31 | 0.3898 | 0.7772 | 0.6968 | 0.8869 | 0.8499 | -0.2346 | -0.3953 | 0.004061 | 0.02933 | 3.441 | 1.783 | 3.245 | 0.8711 | 0.9415 | 1.065 | 0.09032 | 0.1644 |  |
| 2010106E10Rik | RIKEN cDNA 2010106E10 gene | 0.6052 | 0.8285 | 1.137 | 0.1854 | 0.5252 | 0.6909 | 0.8841 | 0.8898 | -0.1685 | -0.4035 | 0.00427 | 0.03036 | 2.372 | 1.246 | 3.223 | 0.6382 | 0.8095 | 0.8799 | -0.1846 | -0.4774 |  |
| Cdhr3 | cadherin-related family member | 0.9425 | 0.98 | 0.983 | -0.02467 | -0.07305 | 0.5543 | 0.8167 | 1.181 | 0.2403 | 0.6015 | 0.007276 | 0.04492 | 0.4648 | -1.105 | -2.988 | 0.4115 | 0.6404 | 1.24 | 0.3103 | 0.8388 |  |
| Gm41338 | NA | 0.3617 | 0.6573 | 1.336 | 0.4178 | 0.9334 | 0.8965 | 0.965 | 0.9528 | -0.06977 | -0.1317 | 0.007485 | 0.04588 | 2.749 | 1.459 | 2.975 | 0.9938 | 0.997 | 1.003 | 0.003832 | 0.007815 |  |
