# Supplementary material for: GIANT: galaxy-based tool for interactive analysis of transcriptomic data
Source: Sci Rep. 2020 Nov 16;10:19835. doi: 10.1038/s41598-020-76769-w (PMC7670435; doi:10.1038/s41598-020-76769-w)
Supplement: Supplementary file 2 — Supplementary Information. [file 41598_2020_76769_MOESM2_ESM.zip › Tools_InputOutput_Parameters/MicroArray_workflow/Step4-LIMMA/Outputs/LIMMA_FastedVsFed_HTML.html_75.html]

### LIMMA statistics (p.val, FC)

LIMMA results

### P-val histograms

Histogram Diet\_Fasted:Tissue\_AdiposeTis

Histogram Diet\_Fasted:Tissue\_SkeletalMus

Histogram FastedVsFed

Histogram FastedVsFed-Liver

### Source of variation

F-ratio barplot
