# Supplementary material for: GIANT: galaxy-based tool for interactive analysis of transcriptomic data
Source: Sci Rep. 2020 Nov 16;10:19835. doi: 10.1038/s41598-020-76769-w (PMC7670435; doi:10.1038/s41598-020-76769-w)
Supplement: Supplementary file 2 — Supplementary Information. [file 41598_2020_76769_MOESM2_ESM.zip › Tools_InputOutput_Parameters/MicroArray_workflow/Step5-Volcano/Outputs/extra_files_path_78/output.html]

| Gene | Info | FastedVsFed | | | | FastedVsFed-Liver | | | |  |
| --- | --- | --- | --- | --- | --- | --- | --- | --- | --- | --- |
|  |  | p-val | Adjusted.p-val | FC | log2(FC) | p-val | Adjusted.p-val | FC | log2(FC) |  |
| Gene | Info | p-val | Adjusted.p-val | FC | log2(FC) | p-val | Adjusted.p-val | FC | log2(FC) |  |
| --- | --- | --- | --- | --- | --- | --- | --- | --- | --- | --- |
| Cdkn1a | na | 2.06e-13 | 4.899e-10 | 14.03 | 3.81 | 9.415e-15 | 1.298e-11 | 39.56 | 5.306 |  |
| --- | --- | --- | --- | --- | --- | --- | --- | --- | --- | --- |
| Per1 | na | 4.565e-12 | 5.428e-09 | 5.07 | 2.342 | 3.353e-11 | 8.859e-09 | 5.591 | 2.483 |  |
| Zbtb16 | na | 3.152e-10 | 7.495e-08 | 3.317 | 1.73 | 7.096e-13 | 3.21e-10 | 7.301 | 2.868 |  |
| Srebf1 | na | 7.111e-10 | 1.301e-07 | 0.1572 | -2.669 | 8.962e-11 | 2.131e-08 | 0.08514 | -3.554 |  |
| Fmo2 | na | 3.53e-10 | 7.631e-08 | 4.986 | 2.318 | 7.893e-10 | 8.938e-08 | 6.143 | 2.619 |  |
| Serpina7 | na | 1.968e-08 | 1.3e-06 | 0.2868 | -1.802 | 1.092e-14 | 1.298e-11 | 0.03678 | -4.765 |  |
| Mvk | na | 1.142e-08 | 9.052e-07 | 0.3681 | -1.442 | 1.385e-10 | 2.533e-08 | 0.2161 | -2.21 |  |
| Aldh4a1 | na | 2.401e-09 | 3.349e-07 | 0.4793 | -1.061 | 2.207e-09 | 1.802e-07 | 0.417 | -1.262 |  |
| Plin5 | na | 3.031e-09 | 3.604e-07 | 3.959 | 1.985 | 3.944e-09 | 2.68e-07 | 4.966 | 2.312 |  |
| Eif4ebp3 | na | 7.44e-09 | 7.517e-07 | 3.408 | 1.769 | 2.776e-09 | 2e-07 | 4.659 | 2.22 |  |
| Fkbp5 | na | 3.287e-09 | 3.722e-07 | 9.158 | 3.195 | 4.289e-09 | 2.833e-07 | 13.18 | 3.72 |  |
| Lss | na | 1.025e-07 | 4.514e-06 | 0.3934 | -1.346 | 2.245e-11 | 6.673e-09 | 0.1624 | -2.622 |  |
| Acot1 | na | 5.516e-08 | 2.852e-06 | 2.83 | 1.501 | 3.864e-10 | 5.121e-08 | 5.252 | 2.393 |  |
| Slc27a1 | na | 3.912e-08 | 2.163e-06 | 3.207 | 1.681 | 1.58e-09 | 1.566e-07 | 5.337 | 2.416 |  |
| Gm15998 | na | 4.259e-07 | 1.269e-05 | 0.3434 | -1.542 | 1.943e-14 | 1.54e-11 | 0.03533 | -4.823 |  |
| E230001N04Rik | na | 7.903e-09 | 7.517e-07 | 5.184 | 2.374 | 1.764e-08 | 7.452e-07 | 6.382 | 2.674 |  |
| Pcsk9 | na | 4.537e-07 | 1.3e-05 | 0.4025 | -1.313 | 6.793e-13 | 3.21e-10 | 0.09382 | -3.414 |  |
| Dhcr7 | na | 2.417e-08 | 1.474e-06 | 0.3164 | -1.66 | 1.137e-08 | 5.2e-07 | 0.2401 | -2.058 |  |
| Hsd3b5 | na | 8.454e-07 | 2.116e-05 | 0.1843 | -2.44 | 8.1e-13 | 3.21e-10 | 0.01062 | -6.557 |  |
| Lpin1 | na | 1.728e-07 | 6.523e-06 | 2.914 | 1.543 | 5.376e-09 | 3.455e-07 | 4.82 | 2.269 |  |
| Il6ra | na | 9.384e-11 | 3.359e-08 | 6.589 | 2.72 | 4.763e-07 | 1.02e-05 | 3.769 | 1.914 |  |
| Ddit4 | na | 2.516e-11 | 1.299e-08 | 41.38 | 5.371 | 6.18e-07 | 1.256e-05 | 10.92 | 3.449 |  |
| Ivns1abp | na | 9.872e-09 | 8.445e-07 | 2.637 | 1.399 | 2.992e-07 | 7.187e-06 | 2.519 | 1.333 |  |
| Fbxo31 | na | 9.944e-09 | 8.445e-07 | 4.161 | 2.057 | 3.589e-07 | 8.052e-06 | 3.829 | 1.937 |  |
| Gamt | na | 8.387e-10 | 1.425e-07 | 0.2621 | -1.932 | 6.598e-07 | 1.308e-05 | 0.3518 | -1.507 |  |
| Insig1 | na | 2.01e-06 | 4.137e-05 | 0.3964 | -1.335 | 2.101e-09 | 1.802e-07 | 0.1823 | -2.456 |  |
| Gm38481 | na | 1.29e-06 | 2.887e-05 | 0.3117 | -1.682 | 6.249e-09 | 3.81e-07 | 0.1427 | -2.809 |  |
| Idi1 | na | 3.843e-07 | 1.202e-05 | 0.2427 | -2.043 | 3.096e-08 | 1.083e-06 | 0.1392 | -2.845 |  |
| Fdps | na | 1.109e-06 | 2.585e-05 | 0.3213 | -1.638 | 1.084e-08 | 5.184e-07 | 0.163 | -2.617 |  |
| Sc5d | na | 1.02e-06 | 2.45e-05 | 0.4714 | -1.085 | 1.136e-08 | 5.2e-07 | 0.3038 | -1.719 |  |
| Hes6 | na | 2.853e-06 | 5.428e-05 | 0.4251 | -1.234 | 1.081e-08 | 5.184e-07 | 0.2318 | -2.109 |  |
| Retsat | na | 1.204e-06 | 2.753e-05 | 1.872 | 0.9048 | 5.595e-08 | 1.834e-06 | 2.485 | 1.313 |  |
| 1500012F01Rik | na | 5.741e-10 | 1.138e-07 | 2.68 | 1.422 | 3.466e-06 | 4.965e-05 | 1.951 | 0.9645 |  |
| Lgals4 | na | 4.268e-07 | 1.269e-05 | 3.169 | 1.664 | 2.823e-07 | 6.85e-06 | 4.067 | 2.024 |  |
| Cebpd | na | 2.624e-11 | 1.299e-08 | 6.165 | 2.624 | 5.737e-06 | 7.322e-05 | 2.713 | 1.44 |  |
| Acot2 | na | 7.465e-07 | 1.951e-05 | 3.959 | 1.985 | 2.659e-07 | 6.519e-06 | 5.728 | 2.518 |  |
| Fam214a | na | 9.395e-08 | 4.296e-06 | 3.112 | 1.638 | 1.624e-06 | 2.819e-05 | 3.029 | 1.599 |  |
| Cyp17a1 | na | 2.225e-05 | 0.0002544 | 3.265 | 1.707 | 9.986e-11 | 2.159e-08 | 22.49 | 4.491 |  |
| Nsdhl | na | 6.442e-06 | 0.0001008 | 0.4181 | -1.258 | 1.17e-08 | 5.25e-07 | 0.2069 | -2.273 |  |
| Gcat | na | 3.628e-06 | 6.487e-05 | 0.5957 | -0.7474 | 2.542e-08 | 9.3e-07 | 0.4251 | -1.234 |  |
| Fam107a | na | 1.931e-07 | 6.957e-06 | 5.07 | 2.342 | 1.386e-06 | 2.46e-05 | 5.367 | 2.424 |  |
| Usp2 | na | 4.326e-05 | 0.0004161 | 1.8 | 0.848 | 1.855e-11 | 6.302e-09 | 6.017 | 2.589 |  |
| Acss2 | na | 1.299e-06 | 2.887e-05 | 0.3373 | -1.568 | 4.073e-07 | 8.968e-06 | 0.2484 | -2.009 |  |
| Efna1 | na | 8.467e-06 | 0.0001235 | 0.515 | -0.9574 | 2.515e-08 | 9.3e-07 | 0.311 | -1.685 |  |
| Ttyh2 | na | 7.879e-09 | 7.517e-07 | 0.441 | -1.181 | 9.051e-06 | 0.0001008 | 0.547 | -0.8704 |  |
| Slc22a5 | na | 3.096e-05 | 0.0003243 | 1.82 | 0.8636 | 8.93e-09 | 4.925e-07 | 3.47 | 1.795 |  |
| Slc25a34 | na | 2.198e-07 | 7.687e-06 | 3.583 | 1.841 | 4.146e-06 | 5.699e-05 | 3.422 | 1.775 |  |
| Rgn | na | 8.306e-05 | 0.0006589 | 0.6653 | -0.588 | 1.336e-10 | 2.533e-08 | 0.3078 | -1.7 |  |
| Mlxipl | na | 1.344e-05 | 0.0001737 | 0.6024 | -0.7312 | 1.104e-07 | 3.163e-06 | 0.4311 | -1.214 |  |
| Mvd | na | 6.495e-05 | 0.000562 | 0.4423 | -1.177 | 2.273e-09 | 1.802e-07 | 0.1408 | -2.828 |  |
| Cyp2u1 | na | 3.141e-06 | 5.753e-05 | 0.4611 | -1.117 | 8.671e-07 | 1.65e-05 | 0.3665 | -1.448 |  |
| Gadd45b | na | 1.515e-05 | 0.0001886 | 3.091 | 1.628 | 9.237e-08 | 2.746e-06 | 6.783 | 2.762 |  |
| 1600002H07Rik | na | 2.029e-05 | 0.0002354 | 1.981 | 0.9863 | 8.526e-08 | 2.603e-06 | 3.297 | 1.721 |  |
| Rgs16 | na | 7.034e-05 | 0.0005849 | 4.202 | 2.071 | 2.907e-09 | 2.033e-07 | 30.68 | 4.939 |  |
| Mylip | na | 3.145e-06 | 5.753e-05 | 2.13 | 1.091 | 1.404e-06 | 2.473e-05 | 2.581 | 1.368 |  |
| Cd276 | na | 1.101e-05 | 0.0001505 | 0.5797 | -0.7867 | 3.443e-07 | 7.873e-06 | 0.4371 | -1.194 |  |
| Pmvk | na | 3.942e-05 | 0.0003842 | 0.4513 | -1.148 | 2.015e-08 | 7.855e-07 | 0.2007 | -2.317 |  |
| Dpy19l3 | na | 4.68e-05 | 0.0004416 | 0.4115 | -1.281 | 1.84e-08 | 7.452e-07 | 0.1608 | -2.637 |  |
| Adh4 | na | 7.392e-05 | 0.0006082 | 0.6141 | -0.7035 | 8.693e-09 | 4.922e-07 | 0.3352 | -1.577 |  |
| Slc16a5 | na | 0.0001427 | 0.000964 | 2.059 | 1.042 | 1.851e-10 | 2.934e-08 | 8.61 | 3.106 |  |
| Foxo1 | na | 5.92e-08 | 2.995e-06 | 2.912 | 1.542 | 1.531e-05 | 0.0001522 | 2.355 | 1.236 |  |
| Dclre1b | na | 1.781e-07 | 6.549e-06 | 2.309 | 1.207 | 1.039e-05 | 0.0001128 | 2.101 | 1.071 |  |
| Map3k6 | na | 2.731e-11 | 1.299e-08 | 3.792 | 1.923 | 3.777e-05 | 0.0003097 | 1.881 | 0.9113 |  |
| Car3 | na | 8.091e-05 | 0.0006485 | 0.5928 | -0.7543 | 9.584e-09 | 4.955e-07 | 0.3089 | -1.695 |  |
| Catsper2 | na | 2.355e-05 | 0.0002629 | 1.856 | 0.8925 | 2.21e-07 | 5.718e-06 | 2.791 | 1.481 |  |
| Adgrf1 | na | 0.0001222 | 0.00087 | 0.4463 | -1.164 | 2.503e-09 | 1.884e-07 | 0.1301 | -2.942 |  |
| Clec2h | na | 0.0001321 | 0.0009093 | 0.3489 | -1.519 | 1.869e-09 | 1.709e-07 | 0.06552 | -3.932 |  |
| Sqle | na | 5.211e-05 | 0.0004739 | 0.346 | -1.531 | 3.788e-08 | 1.287e-06 | 0.1214 | -3.042 |  |
| Bnip3 | na | 1.746e-06 | 3.72e-05 | 2.17 | 1.118 | 5.039e-06 | 6.584e-05 | 2.338 | 1.225 |  |
| Hsd3b4 | na | 0.0001735 | 0.001118 | 0.4414 | -1.18 | 4.839e-10 | 5.754e-08 | 0.09552 | -3.388 |  |
| Nr1d1 | na | 1.584e-07 | 6.102e-06 | 0.2852 | -1.81 | 1.536e-05 | 0.0001522 | 0.3427 | -1.545 |  |
| Slc20a1 | na | 4.418e-05 | 0.0004186 | 1.769 | 0.823 | 2.444e-07 | 6.249e-06 | 2.693 | 1.429 |  |
| Mfsd2a | na | 0.0002274 | 0.001362 | 2.551 | 1.351 | 3.438e-10 | 5.11e-08 | 16.69 | 4.061 |  |
| Hsdl2 | na | 6.648e-05 | 0.0005664 | 1.535 | 0.6178 | 1.071e-07 | 3.109e-06 | 2.255 | 1.173 |  |
| Hsd17b10 | na | 1.053e-05 | 0.000149 | 1.713 | 0.7763 | 2.519e-06 | 3.84e-05 | 2.031 | 1.022 |  |
| Prelid2 | na | 4.283e-06 | 7.275e-05 | 1.982 | 0.9872 | 4.984e-06 | 6.548e-05 | 2.227 | 1.155 |  |
| Apex2 | na | 0.0002739 | 0.001562 | 1.683 | 0.7514 | 3.876e-10 | 5.121e-08 | 4.87 | 2.284 |  |
| Pfkfb2 | na | 0.0002808 | 0.001579 | 1.485 | 0.5701 | 1.789e-10 | 2.934e-08 | 3.519 | 1.815 |  |
| Tnfaip2 | na | 3.192e-06 | 5.794e-05 | 0.3112 | -1.684 | 6.942e-06 | 8.337e-05 | 0.2717 | -1.88 |  |
| Rnpep | na | 8.1e-05 | 0.0006485 | 0.6746 | -0.568 | 1.767e-07 | 4.775e-06 | 0.479 | -1.062 |  |
| Tlcd2 | na | 8.551e-06 | 0.000124 | 0.5098 | -0.9721 | 4.408e-06 | 5.99e-05 | 0.4326 | -1.209 |  |
| Lrp2bp | na | 1.228e-05 | 0.000165 | 0.4184 | -1.257 | 3.243e-06 | 4.731e-05 | 0.3197 | -1.645 |  |
| Acsl3 | na | 3.631e-05 | 0.0003628 | 0.4579 | -1.127 | 7.721e-07 | 1.505e-05 | 0.291 | -1.781 |  |
| Igfals | na | 1.226e-07 | 5.206e-06 | 0.2692 | -1.893 | 3.575e-05 | 0.0002983 | 0.3572 | -1.485 |  |
| Mir704 | na | 6.054e-05 | 0.0005312 | 2.476 | 1.308 | 3.835e-07 | 8.523e-06 | 4.803 | 2.264 |  |
| Slc45a3 | na | 2.043e-05 | 0.0002358 | 2.176 | 1.122 | 2.046e-06 | 3.289e-05 | 2.99 | 1.58 |  |
| Nid2 | na | 3.524e-07 | 1.148e-05 | 0.3558 | -1.491 | 2.541e-05 | 0.000223 | 0.4092 | -1.289 |  |
| Aim1 | na | 1.765e-05 | 0.0002152 | 2.444 | 1.289 | 2.95e-06 | 4.33e-05 | 3.359 | 1.748 |  |
| Fam149a | na | 7.245e-05 | 0.0005982 | 0.6332 | -0.6592 | 3.579e-07 | 8.052e-06 | 0.446 | -1.165 |  |
| Cpt1a | na | 4.398e-05 | 0.0004186 | 1.809 | 0.855 | 1.272e-06 | 2.274e-05 | 2.509 | 1.327 |  |
| Mir185 | na | 1.061e-05 | 0.0001491 | 2.418 | 1.274 | 5.875e-06 | 7.392e-05 | 2.986 | 1.578 |  |
| Tsc22d3 | na | 5.487e-06 | 8.958e-05 | 2.493 | 1.318 | 8.694e-06 | 9.892e-05 | 2.84 | 1.506 |  |
| Rab27a | na | 4.342e-07 | 1.275e-05 | 2.225 | 1.154 | 3.083e-05 | 0.0002609 | 1.995 | 0.9962 |  |
| Slc10a6 | na | 1.108e-08 | 9.052e-07 | 3.832 | 1.938 | 8.55e-05 | 0.0005876 | 2.318 | 1.213 |  |
| Tor1a | na | 7.003e-05 | 0.0005849 | 1.518 | 0.6018 | 7.91e-07 | 1.529e-05 | 2.006 | 1.004 |  |
| Acot3 | na | 6.949e-05 | 0.0005839 | 2.518 | 1.332 | 1.112e-06 | 2.099e-05 | 4.5 | 2.17 |  |
| Abca8a | na | 0.0001889 | 0.001191 | 0.4547 | -1.137 | 8.539e-08 | 2.603e-06 | 0.1884 | -2.408 |  |
| Gabbr2 | na | 0.0002108 | 0.001292 | 0.6316 | -0.6629 | 5.475e-08 | 1.834e-06 | 0.3637 | -1.459 |  |
| Nudt7 | na | 0.0004181 | 0.002076 | 0.5145 | -0.9587 | 2.204e-09 | 1.802e-07 | 0.1491 | -2.746 |  |
| Galnt15 | na | 2.221e-10 | 6.602e-08 | 3.336 | 1.738 | 0.0001336 | 0.0008273 | 1.777 | 0.8298 |  |
| Cyp4a31 | na | 0.0005201 | 0.002438 | 1.9 | 0.9261 | 4.203e-10 | 5.26e-08 | 7.934 | 2.988 |  |
| St3gal5 | na | 0.000299 | 0.00165 | 1.804 | 0.8511 | 1.637e-08 | 7.078e-07 | 4.255 | 2.089 |  |
| Gstm3 | na | 0.0003867 | 0.001966 | 0.4389 | -1.188 | 6.581e-09 | 3.912e-07 | 0.1117 | -3.162 |  |
| 5830473C10Rik | na | 0.0002746 | 0.001562 | 0.6063 | -0.7218 | 2.905e-08 | 1.047e-06 | 0.3086 | -1.696 |  |
| Acss3 | na | 2.475e-05 | 0.0002725 | 2.173 | 1.12 | 5.439e-06 | 7.041e-05 | 2.811 | 1.491 |  |
| Nr4a1 | na | 9.736e-05 | 0.0007373 | 0.3832 | -1.384 | 1.147e-06 | 2.114e-05 | 0.2007 | -2.317 |  |
| Pgd | na | 1.418e-05 | 0.0001795 | 0.5175 | -0.9504 | 9.073e-06 | 0.0001008 | 0.4463 | -1.164 |  |
| Gstm6 | na | 8.483e-05 | 0.000668 | 0.5043 | -0.9877 | 1.84e-06 | 3.081e-05 | 0.3345 | -1.58 |  |
| Acot4 | na | 1.182e-05 | 0.0001606 | 2.774 | 1.472 | 1.291e-05 | 0.0001352 | 3.317 | 1.73 |  |
| Noct | na | 0.0001246 | 0.0008818 | 2.242 | 1.165 | 1.135e-06 | 2.109e-05 | 3.992 | 1.997 |  |
| Id3 | na | 0.0004675 | 0.002264 | 0.4813 | -1.055 | 1.09e-08 | 5.184e-07 | 0.146 | -2.776 |  |
| Pnpla2 | na | 5.5e-06 | 8.958e-05 | 1.982 | 0.9869 | 2.15e-05 | 0.0001966 | 2.071 | 1.05 |  |
| Sorbs3 | na | 2.543e-06 | 4.957e-05 | 2.579 | 1.367 | 3.665e-05 | 0.0003037 | 2.49 | 1.316 |  |
| Gm3571 | na | 0.0001929 | 0.001207 | 0.4585 | -1.125 | 4.907e-07 | 1.033e-05 | 0.2291 | -2.126 |  |
| Keg1 | na | 0.0005759 | 0.002614 | 0.4924 | -1.022 | 9.273e-09 | 4.925e-07 | 0.146 | -2.776 |  |
| Prkch | na | 1.081e-05 | 0.0001503 | 2.062 | 1.044 | 1.856e-05 | 0.0001765 | 2.27 | 1.183 |  |
| Cyb5b | na | 2.824e-07 | 9.458e-06 | 0.4386 | -1.189 | 9.224e-05 | 0.0006214 | 0.533 | -0.9078 |  |
| Nrep | na | 3.115e-08 | 1.807e-06 | 0.1992 | -2.328 | 0.0001463 | 0.000883 | 0.3587 | -1.479 |  |
| Chrna4 | na | 0.0004875 | 0.002323 | 0.4377 | -1.192 | 2.462e-08 | 9.293e-07 | 0.1253 | -2.997 |  |
| Tango2 | na | 7.519e-06 | 0.0001111 | 2.225 | 1.154 | 2.462e-05 | 0.0002193 | 2.367 | 1.243 |  |
| Fam134b | na | 1.082e-06 | 2.573e-05 | 2.61 | 1.384 | 7.251e-05 | 0.0005147 | 2.269 | 1.182 |  |
| Plin4 | na | 0.0002698 | 0.001553 | 2.085 | 1.06 | 6.429e-07 | 1.294e-05 | 4.09 | 2.032 |  |
| Wdr6 | na | 0.0001181 | 0.000851 | 0.6148 | -0.7018 | 3.68e-06 | 5.202e-05 | 0.4665 | -1.1 |  |
| C330021F23Rik | na | 0.0009011 | 0.003705 | 0.5104 | -0.9704 | 9.319e-09 | 4.925e-07 | 0.1472 | -2.764 |  |
| Msmo1 | na | 0.0002209 | 0.00134 | 0.4272 | -1.227 | 1.228e-06 | 2.212e-05 | 0.2169 | -2.205 |  |
| Hmgcr | na | 0.001405 | 0.005156 | 0.7108 | -0.4924 | 1.23e-09 | 1.33e-07 | 0.3156 | -1.664 |  |
| Cblb | na | 9.64e-08 | 4.325e-06 | 2.282 | 1.19 | 0.0002378 | 0.001331 | 1.712 | 0.776 |  |
| Hn1l | na | 5.144e-05 | 0.0004723 | 0.5159 | -0.9547 | 1.465e-05 | 0.0001489 | 0.4199 | -1.252 |  |
| Gm5510 | na | 1.752e-06 | 3.72e-05 | 0.4655 | -1.103 | 9.621e-05 | 0.0006445 | 0.5171 | -0.9515 |  |
| Gm9824 | na | 2.293e-05 | 0.0002597 | 0.5119 | -0.966 | 2.344e-05 | 0.0002103 | 0.4534 | -1.141 |  |
| Ip6k2 | na | 0.0002862 | 0.001601 | 1.542 | 0.6244 | 1.781e-06 | 3.004e-05 | 2.175 | 1.121 |  |
| Nqo2 | na | 3.924e-06 | 6.912e-05 | 0.475 | -1.074 | 9.223e-05 | 0.0006214 | 0.5051 | -0.9854 |  |
| Angptl2 | na | 3.395e-08 | 1.922e-06 | 0.284 | -1.816 | 0.0004451 | 0.002178 | 0.4857 | -1.042 |  |
| Ociad2 | na | 3.366e-05 | 0.0003406 | 0.488 | -1.035 | 2.696e-05 | 0.000234 | 0.4213 | -1.247 |  |
| As3mt | na | 0.001069 | 0.004206 | 1.571 | 0.6518 | 6.795e-08 | 2.126e-06 | 3.187 | 1.672 |  |
| Man2a2 | na | 0.0001266 | 0.0008876 | 0.6214 | -0.6864 | 1.026e-05 | 0.0001119 | 0.4993 | -1.002 |  |
| Aqp8 | na | 0.001541 | 0.005478 | 0.5067 | -0.9807 | 1.074e-08 | 5.184e-07 | 0.1299 | -2.944 |  |
| Gsta4 | na | 6.85e-06 | 0.0001042 | 0.4341 | -1.204 | 8.9e-05 | 0.000606 | 0.4488 | -1.156 |  |
| Tspan7 | na | 0.0001191 | 0.0008556 | 0.6111 | -0.7105 | 1.29e-05 | 0.0001352 | 0.4955 | -1.013 |  |
| Abhd2 | na | 9.641e-05 | 0.0007328 | 2.672 | 1.418 | 1.75e-05 | 0.0001692 | 3.829 | 1.937 |  |
| Hsd3b2 | na | 0.001013 | 0.004028 | 0.6036 | -0.7284 | 1.875e-07 | 5.01e-06 | 0.2994 | -1.74 |  |
| Gpc4 | na | 9.578e-07 | 2.348e-05 | 0.4444 | -1.17 | 0.0002402 | 0.001338 | 0.5401 | -0.8888 |  |
| Sort1 | na | 0.0007881 | 0.003353 | 0.6286 | -0.6698 | 4.198e-07 | 9.075e-06 | 0.3597 | -1.475 |  |
| Abcb10 | na | 0.0004283 | 0.002108 | 0.6655 | -0.5875 | 2.038e-06 | 3.289e-05 | 0.4707 | -1.087 |  |
| Cyp2c55 | na | 0.001619 | 0.005645 | 0.4466 | -1.163 | 3.078e-08 | 1.083e-06 | 0.1022 | -3.291 |  |
| Por | na | 0.0001897 | 0.001191 | 1.855 | 0.8918 | 1.13e-05 | 0.0001205 | 2.533 | 1.341 |  |
| Gm15441 | na | 0.002451 | 0.007501 | 1.89 | 0.9181 | 1.777e-09 | 1.69e-07 | 9.494 | 3.247 |  |
| Cyp39a1 | na | 0.001666 | 0.005758 | 1.847 | 0.8851 | 5.722e-08 | 1.839e-06 | 5.337 | 2.416 |  |
| Acsm3 | na | 1.508e-07 | 6.102e-06 | 3.499 | 1.807 | 0.0006716 | 0.003054 | 2.132 | 1.092 |  |
| Cyp4f14 | na | 0.001076 | 0.004212 | 0.6245 | -0.6792 | 5.894e-07 | 1.208e-05 | 0.3506 | -1.512 |  |
| Slc25a20 | na | 0.0003197 | 0.001732 | 1.561 | 0.6426 | 7.326e-06 | 8.667e-05 | 2.072 | 1.051 |  |
| Aifm2 | na | 0.0002123 | 0.001298 | 1.891 | 0.9192 | 1.494e-05 | 0.00015 | 2.579 | 1.367 |  |
| Thrsp | na | 0.0007918 | 0.003362 | 0.331 | -1.595 | 2.047e-06 | 3.289e-05 | 0.1128 | -3.148 |  |
| Defb1 | na | 0.001728 | 0.00593 | 1.714 | 0.7775 | 1.072e-07 | 3.109e-06 | 4.135 | 2.048 |  |
| 1810011O10Rik | na | 0.0005156 | 0.002428 | 2.304 | 1.204 | 4.53e-06 | 6.121e-05 | 4.414 | 2.142 |  |
| Uhrf1bp1l | na | 8.192e-07 | 2.072e-05 | 2.034 | 1.024 | 0.0005498 | 0.002594 | 1.634 | 0.7083 |  |
| Ergic1 | na | 5.663e-07 | 1.548e-05 | 0.4395 | -1.186 | 0.0006574 | 0.003012 | 0.5805 | -0.7846 |  |
| Gm14964 | na | 5.701e-06 | 9.16e-05 | 2.592 | 1.374 | 0.0002449 | 0.001345 | 2.272 | 1.184 |  |
| Mmp15 | na | 7.699e-05 | 0.0006249 | 0.5082 | -0.9766 | 4.709e-05 | 0.0003593 | 0.4335 | -1.206 |  |
| Cirbp | na | 9.809e-05 | 0.0007376 | 1.982 | 0.9867 | 4.064e-05 | 0.0003211 | 2.395 | 1.26 |  |
| Man2a1 | na | 5.835e-05 | 0.0005177 | 1.848 | 0.8859 | 7.17e-05 | 0.0005105 | 2.042 | 1.03 |  |
| Chrna2 | na | 0.00039 | 0.001973 | 2.565 | 1.359 | 8.901e-06 | 0.0001005 | 4.701 | 2.233 |  |
| Phlda2 | na | 0.0008547 | 0.003578 | 0.6553 | -0.6097 | 3.697e-06 | 5.202e-05 | 0.4469 | -1.162 |  |
| Abca2 | na | 0.001128 | 0.00434 | 0.612 | -0.7084 | 1.946e-06 | 3.214e-05 | 0.3637 | -1.459 |  |
| Slc25a32 | na | 0.0001848 | 0.001172 | 1.796 | 0.8448 | 2.733e-05 | 0.0002355 | 2.269 | 1.182 |  |
| Abca8b | na | 0.0001329 | 0.0009108 | 0.5247 | -0.9303 | 3.933e-05 | 0.0003166 | 0.4281 | -1.224 |  |
| Selenbp2 | na | 0.001932 | 0.006405 | 0.5892 | -0.7631 | 2.625e-07 | 6.502e-06 | 0.2641 | -1.921 |  |
| Abhd17c | na | 0.0002405 | 0.001423 | 0.6121 | -0.7081 | 2.203e-05 | 0.0001993 | 0.4887 | -1.033 |  |
| 1500011K16Rik | na | 9.896e-06 | 0.0001409 | 0.489 | -1.032 | 0.0002856 | 0.001513 | 0.5306 | -0.9143 |  |
| Me1 | na | 9.38e-06 | 0.0001352 | 0.4219 | -1.245 | 0.0003114 | 0.001631 | 0.4701 | -1.089 |  |
| Slc15a4 | na | 1.589e-06 | 3.467e-05 | 2.089 | 1.063 | 0.0007156 | 0.003229 | 1.681 | 0.7491 |  |
| 1810064F22Rik | na | 0.002001 | 0.006545 | 0.518 | -0.949 | 3.359e-07 | 7.831e-06 | 0.1948 | -2.36 |  |
| Serpina4-ps1 | na | 0.00206 | 0.006711 | 0.308 | -1.699 | 3.244e-07 | 7.638e-06 | 0.05256 | -4.25 |  |
| Cyp1a2 | na | 0.001895 | 0.00634 | 0.7217 | -0.4705 | 4.874e-07 | 1.033e-05 | 0.4557 | -1.134 |  |
| Prok1 | na | 0.002809 | 0.008381 | 0.716 | -0.482 | 3.43e-08 | 1.182e-06 | 0.3663 | -1.449 |  |
| Mfsd7c | na | 0.001399 | 0.005146 | 1.696 | 0.7621 | 2.628e-06 | 3.98e-05 | 2.977 | 1.574 |  |
| Prss8 | na | 0.00178 | 0.006082 | 0.5768 | -0.7938 | 1.126e-06 | 2.108e-05 | 0.2888 | -1.792 |  |
| Gm5785 | na | 0.0001058 | 0.0007765 | 1.937 | 0.9541 | 8.299e-05 | 0.0005737 | 2.225 | 1.154 |  |
| Mmab | na | 0.0004432 | 0.00216 | 0.488 | -1.035 | 1.687e-05 | 0.0001644 | 0.3209 | -1.64 |  |
| Rdh11 | na | 0.004053 | 0.01109 | 0.6246 | -0.679 | 6.818e-09 | 3.954e-07 | 0.1941 | -2.365 |  |
| Plxnd1 | na | 2.111e-08 | 1.335e-06 | 0.4098 | -1.287 | 0.004296 | 0.01423 | 0.6832 | -0.5497 |  |
| Abcb1b | na | 1.807e-08 | 1.228e-06 | 2.776 | 1.473 | 0.004437 | 0.01463 | 1.538 | 0.6207 |  |
| 2810416G20Rik | na | 0.003136 | 0.009173 | 1.689 | 0.7562 | 8.798e-08 | 2.648e-06 | 4.506 | 2.172 |  |
| Stk38l | na | 0.0007235 | 0.00314 | 0.6029 | -0.7301 | 1.455e-05 | 0.0001489 | 0.4257 | -1.232 |  |
| Aacs | na | 0.003645 | 0.01014 | 0.4724 | -1.082 | 2.175e-08 | 8.342e-07 | 0.09068 | -3.463 |  |
| Rnf186 | na | 0.001466 | 0.005306 | 0.6163 | -0.6982 | 4.044e-06 | 5.591e-05 | 0.3776 | -1.405 |  |
| Echdc1 | na | 0.0002437 | 0.001434 | 0.5091 | -0.9741 | 4.859e-05 | 0.0003657 | 0.3969 | -1.333 |  |
| Tmem150a | na | 0.0002101 | 0.001291 | 0.5556 | -0.8479 | 6.775e-05 | 0.0004897 | 0.4627 | -1.112 |  |
| Gse1 | na | 0.0004114 | 0.002051 | 0.5975 | -0.7431 | 2.848e-05 | 0.0002445 | 0.4601 | -1.12 |  |
| Fabp5 | na | 0.003314 | 0.009544 | 0.4806 | -1.057 | 1.401e-07 | 3.874e-06 | 0.128 | -2.966 |  |
| Col6a3 | na | 1.567e-07 | 6.102e-06 | 0.3768 | -1.408 | 0.004163 | 0.01392 | 0.6214 | -0.6864 |  |
| Col15a1 | na | 5.464e-09 | 5.906e-07 | 0.1909 | -2.389 | 0.009709 | 0.02829 | 0.5608 | -0.8344 |  |
| Tox | na | 0.001676 | 0.00578 | 1.952 | 0.9647 | 4.268e-06 | 5.833e-05 | 3.901 | 1.964 |  |
| Cep85l | na | 2.916e-06 | 5.503e-05 | 2.704 | 1.435 | 0.00153 | 0.006177 | 1.959 | 0.97 |  |
| Gm19680 | na | 0.001892 | 0.00634 | 0.6097 | -0.7138 | 2.772e-06 | 4.127e-05 | 0.3482 | -1.522 |  |
| Mbl1 | na | 0.002201 | 0.007025 | 0.692 | -0.5312 | 1.741e-06 | 2.957e-05 | 0.4374 | -1.193 |  |
| Asl | na | 0.009784 | 0.02308 | 1.231 | 0.2993 | 2.535e-09 | 1.884e-07 | 2.392 | 1.258 |  |
| Grn | na | 0.0007623 | 0.003266 | 1.839 | 0.8789 | 2.105e-05 | 0.000194 | 2.728 | 1.448 |  |
| Asic5 | na | 0.00694 | 0.01739 | 1.434 | 0.5203 | 1.983e-08 | 7.855e-07 | 3.56 | 1.832 |  |
| Slc22a28 | na | 0.003014 | 0.008881 | 0.6162 | -0.6985 | 5.293e-07 | 1.104e-05 | 0.2926 | -1.773 |  |
| Ttc39c | na | 0.001637 | 0.005673 | 0.6652 | -0.5881 | 6.431e-06 | 7.924e-05 | 0.4481 | -1.158 |  |
| Clec1b | na | 0.0001317 | 0.0009093 | 1.839 | 0.8792 | 0.0001373 | 0.0008437 | 2.051 | 1.036 |  |
| Arrdc2 | na | 9.104e-08 | 4.245e-06 | 3.926 | 1.973 | 0.006983 | 0.02143 | 1.819 | 0.8631 |  |
| Atp11a | na | 0.0007085 | 0.003097 | 0.5116 | -0.9669 | 2.521e-05 | 0.000222 | 0.3399 | -1.557 |  |
| F11 | na | 0.001506 | 0.005402 | 0.6455 | -0.6316 | 8.447e-06 | 9.657e-05 | 0.4335 | -1.206 |  |
| Afm | na | 0.003822 | 0.01053 | 0.7698 | -0.3775 | 2.212e-07 | 5.718e-06 | 0.4836 | -1.048 |  |
| Gm15453 | na | 0.002335 | 0.007287 | 1.63 | 0.7052 | 2.346e-06 | 3.67e-05 | 2.953 | 1.562 |  |
| Sec61g | na | 0.001845 | 0.006241 | 0.7012 | -0.512 | 5.789e-06 | 7.322e-05 | 0.4894 | -1.031 |  |
| Cyp51 | na | 0.003249 | 0.009434 | 0.4945 | -1.016 | 6.475e-07 | 1.294e-05 | 0.1685 | -2.569 |  |
| Acly | na | 8.869e-05 | 0.0006909 | 0.3558 | -1.491 | 0.0002587 | 0.001405 | 0.3303 | -1.598 |  |
| 6430571L13Rik | na | 2.75e-09 | 3.442e-07 | 0.2994 | -1.74 | 0.01782 | 0.04631 | 0.6941 | -0.5268 |  |
| Gale | na | 0.0002501 | 0.001464 | 0.5379 | -0.8945 | 0.0001029 | 0.0006797 | 0.4509 | -1.149 |  |
| Gm21839 | na | 0.0001292 | 0.0008957 | 2.008 | 1.006 | 0.0001988 | 0.001139 | 2.208 | 1.143 |  |
| Gm5499 | na | 6.173e-07 | 1.668e-05 | 0.4914 | -1.025 | 0.004822 | 0.01571 | 0.6894 | -0.5365 |  |
| Eepd1 | na | 0.0001844 | 0.001172 | 0.4734 | -1.079 | 0.0001406 | 0.0008551 | 0.4036 | -1.309 |  |
| Map3k13 | na | 0.002603 | 0.007874 | 0.6809 | -0.5544 | 2.181e-06 | 3.441e-05 | 0.4202 | -1.251 |  |
| Syde2 | na | 5.332e-07 | 1.509e-05 | 2.757 | 1.463 | 0.005352 | 0.01711 | 1.678 | 0.7471 |  |
| Scd2 | na | 4.638e-08 | 2.451e-06 | 0.1812 | -2.464 | 0.01194 | 0.03313 | 0.5177 | -0.9498 |  |
| Col6a2 | na | 9.431e-07 | 2.336e-05 | 0.3901 | -1.358 | 0.004463 | 0.01463 | 0.5989 | -0.7396 |  |
| Lipg | na | 0.001568 | 0.005532 | 2.006 | 1.004 | 1.243e-05 | 0.0001314 | 3.658 | 1.871 |  |
| Steap4 | na | 1.716e-05 | 0.0002111 | 0.3858 | -1.374 | 0.001261 | 0.005307 | 0.4711 | -1.086 |  |
| Vnn1 | na | 1.066e-05 | 0.0001491 | 4.448 | 2.153 | 0.001814 | 0.007083 | 2.971 | 1.571 |  |
| Sdsl | na | 0.021 | 0.04282 | 1.271 | 0.3461 | 1.502e-09 | 1.553e-07 | 3.269 | 1.709 |  |
| Enpep | na | 1.806e-05 | 0.000218 | 0.4016 | -1.316 | 0.001279 | 0.005364 | 0.4853 | -1.043 |  |
| Clec9a | na | 0.0001831 | 0.00117 | 2.478 | 1.309 | 0.0001881 | 0.001083 | 2.918 | 1.545 |  |
| Fam13a | na | 1.966e-05 | 0.0002325 | 3.242 | 1.697 | 0.001285 | 0.00538 | 2.554 | 1.353 |  |
| Cyp2c70 | na | 0.01031 | 0.02415 | 0.5894 | -0.7626 | 5.821e-08 | 1.846e-06 | 0.1576 | -2.666 |  |
| Acer2 | na | 6.867e-08 | 3.333e-06 | 2.728 | 1.448 | 0.01612 | 0.04231 | 1.458 | 0.5439 |  |
| Klk1b4 | na | 0.003509 | 0.009863 | 0.6167 | -0.6973 | 1.74e-06 | 2.957e-05 | 0.316 | -1.662 |  |
| Lyve1 | na | 0.0009892 | 0.003967 | 2.309 | 1.207 | 3.686e-05 | 0.0003044 | 3.872 | 1.953 |  |
| Aplnr | na | 9.887e-11 | 3.359e-08 | 0.3189 | -1.649 | 0.04666 | 0.103 | 1.264 | 0.3385 |  |
| Gas7 | na | 0.001089 | 0.004252 | 0.6166 | -0.6975 | 3.312e-05 | 0.0002783 | 0.45 | -1.152 |  |
| Rnf39 | na | 1.445e-07 | 6.028e-06 | 2.854 | 1.513 | 0.01589 | 0.04189 | 1.513 | 0.5978 |  |
| Tacc2 | na | 1.942e-06 | 4.051e-05 | 2.154 | 1.107 | 0.005693 | 0.01798 | 1.53 | 0.6132 |  |
| BC029214 | na | 0.01098 | 0.02542 | 0.6776 | -0.5616 | 1.376e-07 | 3.85e-06 | 0.2722 | -1.877 |  |
| Crat | na | 0.008839 | 0.0213 | 1.355 | 0.4381 | 3.215e-07 | 7.638e-06 | 2.528 | 1.338 |  |
| Glrx | na | 0.002814 | 0.008386 | 1.615 | 0.6916 | 4.941e-06 | 6.528e-05 | 2.799 | 1.485 |  |
| Acadm | na | 0.01128 | 0.02602 | 1.292 | 0.3694 | 1.633e-07 | 4.464e-06 | 2.339 | 1.226 |  |
| Taf1a | na | 0.000316 | 0.00172 | 1.819 | 0.8632 | 0.0001443 | 0.0008754 | 2.145 | 1.101 |  |
| Fam46a | na | 6.964e-06 | 0.0001048 | 2.628 | 1.394 | 0.003641 | 0.01242 | 1.869 | 0.9021 |  |
| Slc25a22 | na | 2.685e-05 | 0.0002915 | 2.043 | 1.031 | 0.001566 | 0.006301 | 1.77 | 0.824 |  |
| Cdip1 | na | 0.00809 | 0.01967 | 1.411 | 0.4965 | 4.14e-07 | 9.032e-06 | 2.772 | 1.471 |  |
| LOC105243827 | na | 4.382e-06 | 7.39e-05 | 3.915 | 1.969 | 0.004472 | 0.01463 | 2.293 | 1.197 |  |
| Hmgcs2 | na | 6.372e-07 | 1.703e-05 | 5.653 | 2.499 | 0.01253 | 0.03445 | 2.196 | 1.135 |  |
| Slc19a1 | na | 0.01099 | 0.02542 | 1.291 | 0.3686 | 2.479e-07 | 6.271e-06 | 2.274 | 1.185 |  |
| Snord1c | na | 0.0001454 | 0.0009685 | 2.014 | 1.01 | 0.0003928 | 0.001966 | 2.121 | 1.085 |  |
| Gm24253 | na | 0.0001454 | 0.0009685 | 2.014 | 1.01 | 0.0003928 | 0.001966 | 2.121 | 1.085 |  |
| Cyp21a2-ps | na | 0.00372 | 0.01029 | 0.6392 | -0.6457 | 2.453e-06 | 3.788e-05 | 0.3504 | -1.513 |  |
| Adap2 | na | 1.79e-07 | 6.549e-06 | 3.392 | 1.762 | 0.01866 | 0.04784 | 1.608 | 0.6857 |  |
| Lncpint | na | 1.21e-07 | 5.206e-06 | 2.975 | 1.573 | 0.02268 | 0.05606 | 1.49 | 0.5755 |  |
| Tnk2 | na | 2.221e-06 | 4.514e-05 | 0.4398 | -1.185 | 0.007232 | 0.02213 | 0.6416 | -0.6402 |  |
| Zim1 | na | 1.391e-09 | 2.205e-07 | 10.23 | 3.355 | 0.08041 | 0.1586 | 1.62 | 0.6961 |  |
| Arv1 | na | 0.001153 | 0.004422 | 1.554 | 0.6364 | 4.224e-05 | 0.0003315 | 2.051 | 1.036 |  |
| Thbs2 | na | 8.855e-09 | 8.099e-07 | 4.669 | 2.223 | 0.06087 | 0.1286 | 1.47 | 0.5554 |  |
| Tcp11l2 | na | 8.017e-07 | 2.072e-05 | 2.007 | 1.005 | 0.01435 | 0.03867 | 0.7305 | -0.453 |  |
| Serpinh1 | na | 6.746e-06 | 0.0001035 | 0.4078 | -1.294 | 0.004462 | 0.01463 | 0.5693 | -0.8127 |  |
| Cutal | na | 0.002025 | 0.006606 | 0.6815 | -0.5532 | 1.548e-05 | 0.0001527 | 0.485 | -1.044 |  |
| Cerk | na | 5.932e-06 | 9.342e-05 | 2.065 | 1.046 | 0.00483 | 0.01571 | 1.562 | 0.6435 |  |
| Gm40892 | na | 1.286e-05 | 0.0001702 | 2.432 | 1.282 | 0.003427 | 0.01183 | 1.837 | 0.877 |  |
| Klf15 | na | 2.719e-05 | 0.0002939 | 2.302 | 1.203 | 0.002117 | 0.008046 | 1.904 | 0.9288 |  |
| Mycl | na | 1.72e-05 | 0.0002111 | 2.297 | 1.2 | 0.002995 | 0.01056 | 1.808 | 0.8545 |  |
| Plxna1 | na | 1.774e-08 | 1.228e-06 | 0.476 | -1.071 | 0.05527 | 0.1184 | 0.8201 | -0.2862 |  |
| Fmo3 | na | 0.000337 | 0.001797 | 2.549 | 1.35 | 0.0002005 | 0.001146 | 3.204 | 1.68 |  |
| Rarb | na | 3.202e-05 | 0.0003311 | 0.4914 | -1.025 | 0.002056 | 0.00786 | 0.5724 | -0.8048 |  |
| Klhl38 | na | 8.108e-07 | 2.072e-05 | 2.004 | 1.003 | 0.01588 | 0.04189 | 1.361 | 0.4446 |  |
| AA986860 | na | 0.02102 | 0.04282 | 1.214 | 0.2793 | 5.631e-08 | 1.834e-06 | 2.152 | 1.106 |  |
| Gm10156 | na | 0.004125 | 0.01126 | 1.501 | 0.586 | 3.459e-06 | 4.965e-05 | 2.563 | 1.358 |  |
| Nudt2 | na | 0.0002935 | 0.001631 | 0.5537 | -0.8527 | 0.0002742 | 0.001479 | 0.4945 | -1.016 |  |
| Rasgef1b | na | 0.000429 | 0.002108 | 2.578 | 1.366 | 0.0001589 | 0.0009447 | 3.432 | 1.779 |  |
| Lgals1 | na | 0.001978 | 0.006479 | 0.5983 | -0.7411 | 2.086e-05 | 0.000193 | 0.389 | -1.362 |  |
| Pold2 | na | 0.01443 | 0.03171 | 0.7524 | -0.4105 | 3.408e-07 | 7.868e-06 | 0.3917 | -1.352 |  |
| Hsd3b3 | na | 0.009242 | 0.02202 | 0.7057 | -0.5029 | 1.194e-06 | 2.167e-05 | 0.3753 | -1.414 |  |
| Mt2 | na | 0.0002894 | 0.001615 | 4.611 | 2.205 | 0.0003033 | 0.001596 | 6.054 | 2.598 |  |
| Derl3 | na | 0.0003428 | 0.001824 | 2.85 | 1.511 | 0.0002518 | 0.001375 | 3.588 | 1.843 |  |
| Steap3 | na | 4.081e-06 | 7.084e-05 | 0.4793 | -1.061 | 0.007777 | 0.02336 | 0.6631 | -0.5928 |  |
| Trim25 | na | 3.297e-05 | 0.0003379 | 2.007 | 1.005 | 0.002329 | 0.0086 | 1.715 | 0.7786 |  |
| Npc1 | na | 2.294e-07 | 7.906e-06 | 2.838 | 1.505 | 0.0304 | 0.07172 | 1.456 | 0.542 |  |
| Akr1c6 | na | 0.007354 | 0.01818 | 0.7356 | -0.443 | 2.119e-06 | 3.382e-05 | 0.4491 | -1.155 |  |
| Atp4a | na | 0.004816 | 0.01282 | 0.7198 | -0.4744 | 3.745e-06 | 5.239e-05 | 0.4614 | -1.116 |  |
| Ccnd3 | na | 0.0008078 | 0.003418 | 1.879 | 0.9101 | 0.0001083 | 0.0007056 | 2.483 | 1.312 |  |
| Pdk4 | na | 1.749e-08 | 1.228e-06 | 3.787 | 1.921 | 0.09038 | 0.173 | 0.7329 | -0.4483 |  |
| Apol10b | na | 6.551e-07 | 1.731e-05 | 0.442 | -1.178 | 0.02252 | 0.05573 | 0.7155 | -0.483 |  |
| Gm26064 | na | 4.546e-06 | 7.613e-05 | 2.617 | 1.388 | 0.009874 | 0.02867 | 1.686 | 0.7535 |  |
| Ugt1a7c | na | 0.002386 | 0.007378 | 3.162 | 1.661 | 1.983e-05 | 0.0001871 | 8.797 | 3.137 |  |
| Slc4a1 | na | 0.0008673 | 0.003618 | 0.6148 | -0.7018 | 0.0001193 | 0.0007606 | 0.4962 | -1.011 |  |
| Mertk | na | 3.323e-05 | 0.0003391 | 2.056 | 1.04 | 0.002774 | 0.00986 | 1.728 | 0.7892 |  |
| Gm22645 | na | 0.0009781 | 0.003936 | 0.601 | -0.7345 | 0.0001059 | 0.0006918 | 0.4717 | -1.084 |  |
| Mir3064 | na | 0.0009157 | 0.003735 | 1.628 | 0.703 | 0.0001171 | 0.0007506 | 2.029 | 1.021 |  |
| Rpusd3 | na | 0.0007132 | 0.003106 | 0.5865 | -0.7699 | 0.0001526 | 0.0009164 | 0.479 | -1.062 |  |
| Osgin1 | na | 0.05023 | 0.08764 | 0.6936 | -0.5278 | 1.83e-08 | 7.452e-07 | 0.1543 | -2.696 |  |
| Igkv19-93 | na | 6.299e-05 | 0.0005507 | 7.722 | 2.949 | 0.002245 | 0.008381 | 5.37 | 2.425 |  |
| Setdb2 | na | 0.0014 | 0.005146 | 1.862 | 0.897 | 8.285e-05 | 0.0005737 | 2.656 | 1.409 |  |
| Col5a3 | na | 6.333e-05 | 0.0005516 | 0.4938 | -1.018 | 0.002313 | 0.008554 | 0.5607 | -0.8347 |  |
| Hmgcs1 | na | 0.005752 | 0.01497 | 0.463 | -1.111 | 6.283e-06 | 7.782e-05 | 0.1672 | -2.58 |  |
| Ctgf | na | 0.0007532 | 0.003239 | 3.08 | 1.623 | 0.0001667 | 0.0009788 | 4.701 | 2.233 |  |
| Gm25783 | na | 0.004017 | 0.01102 | 1.531 | 0.6143 | 8.342e-06 | 9.646e-05 | 2.511 | 1.328 |  |
| Cxcl14 | na | 6.646e-05 | 0.0005664 | 0.4853 | -1.043 | 0.002574 | 0.009331 | 0.556 | -0.8468 |  |
| Tec | na | 1.388e-05 | 0.0001784 | 2.027 | 1.019 | 0.007475 | 0.02264 | 1.546 | 0.6283 |  |
| Ms4a4a | na | 1.579e-08 | 1.173e-06 | 3.994 | 1.998 | 0.1827 | 0.3034 | 1.283 | 0.3595 |  |
| Fdft1 | na | 0.03351 | 0.0624 | 0.7511 | -0.4129 | 2.132e-07 | 5.633e-06 | 0.3195 | -1.646 |  |
| Snord14e | na | 0.001795 | 0.006115 | 1.775 | 0.8282 | 5.347e-05 | 0.0003986 | 2.624 | 1.392 |  |
| Slc46a1 | na | 2.006e-05 | 0.0002338 | 0.475 | -1.074 | 0.006425 | 0.01989 | 0.6166 | -0.6976 |  |
| Peg3 | na | 4.921e-05 | 0.0004553 | 8.988 | 3.168 | 0.003697 | 0.01256 | 5.252 | 2.393 |  |
| Mt1 | na | 0.0002273 | 0.001362 | 3.6 | 1.848 | 0.0007686 | 0.003429 | 3.816 | 1.932 |  |
| Slc25a19 | na | 9.828e-07 | 2.385e-05 | 0.4039 | -1.308 | 0.03906 | 0.08906 | 0.7107 | -0.4926 |  |
| Dpep1 | na | 0.0002318 | 0.001385 | 2.116 | 1.081 | 0.0007416 | 0.003315 | 2.199 | 1.137 |  |
| Lepr | na | 0.0004946 | 0.002343 | 2.99 | 1.58 | 0.0003174 | 0.001649 | 3.877 | 1.955 |  |
| 1700001C19Rik | na | 0.00959 | 0.02271 | 0.7613 | -0.3935 | 3.683e-06 | 5.202e-05 | 0.4911 | -1.026 |  |
| Trp53inp1 | na | 5.443e-07 | 1.523e-05 | 3.753 | 1.908 | 0.06593 | 0.1353 | 1.524 | 0.6077 |  |
| Chst15 | na | 0.0005964 | 0.002686 | 2.155 | 1.108 | 0.0002756 | 0.001483 | 2.672 | 1.418 |  |
| Insc | na | 0.0032 | 0.009337 | 0.6503 | -0.6208 | 2.025e-05 | 0.0001903 | 0.4308 | -1.215 |  |
| Gjc3 | na | 0.01366 | 0.03044 | 1.643 | 0.7162 | 2.434e-06 | 3.783e-05 | 4.11 | 2.039 |  |
| Gpr182 | na | 1.825e-06 | 3.841e-05 | 2.427 | 1.279 | 0.03215 | 0.07562 | 1.439 | 0.5247 |  |
| Cars | na | 0.02039 | 0.0418 | 0.7685 | -0.3798 | 1.171e-06 | 2.142e-05 | 0.4287 | -1.222 |  |
| Map3k5 | na | 0.00632 | 0.01609 | 1.448 | 0.5345 | 8.962e-06 | 0.0001005 | 2.336 | 1.224 |  |
| Enc1 | na | 1.493e-05 | 0.0001878 | 2.299 | 1.201 | 0.0106 | 0.03012 | 1.631 | 0.7059 |  |
| Ucp2 | na | 2.507e-06 | 4.937e-05 | 3.638 | 1.863 | 0.03501 | 0.0813 | 1.703 | 0.7681 |  |
| Fbxl21 | na | 0.002352 | 0.007321 | 1.479 | 0.5651 | 4.036e-05 | 0.0003202 | 2.007 | 1.005 |  |
| Asb12 | na | 8.386e-08 | 3.988e-06 | 0.3605 | -1.472 | 0.2069 | 0.3329 | 0.8248 | -0.2778 |  |
| Dsg1c | na | 0.002981 | 0.008795 | 0.626 | -0.6757 | 2.645e-05 | 0.0002304 | 0.4112 | -1.282 |  |
| Nes | na | 0.0001323 | 0.0009093 | 0.3363 | -1.572 | 0.002199 | 0.008261 | 0.3832 | -1.384 |  |
| Cxx1a | na | 0.001677 | 0.00578 | 0.6197 | -0.6904 | 0.0001018 | 0.0006756 | 0.4704 | -1.088 |  |
| Pdzd7 | na | 1.561e-07 | 6.102e-06 | 0.3826 | -1.386 | 0.1825 | 0.3033 | 0.8188 | -0.2884 |  |
| Cerkl | na | 0.005904 | 0.01533 | 1.545 | 0.6276 | 1.382e-05 | 0.0001429 | 2.595 | 1.376 |  |
| Sec61b | na | 0.0003103 | 0.001696 | 0.5174 | -0.9506 | 0.0008391 | 0.003695 | 0.4948 | -1.015 |  |
| Rab30 | na | 0.118 | 0.1783 | 1.275 | 0.3504 | 1.849e-08 | 7.452e-07 | 4.864 | 2.282 |  |
| Map2k6 | na | 0.001812 | 0.006156 | 0.6057 | -0.7233 | 9.592e-05 | 0.0006443 | 2.228 | 1.156 |  |
| Slc17a8 | na | 0.01205 | 0.02742 | 1.471 | 0.5566 | 5.448e-06 | 7.041e-05 | 2.757 | 1.463 |  |
| Gm16551 | na | 0.01822 | 0.0382 | 0.5725 | -0.8047 | 2.753e-06 | 4.127e-05 | 0.191 | -2.388 |  |
| 1810055G02Rik | na | 0.1381 | 0.2025 | 1.208 | 0.2726 | 1.571e-08 | 6.918e-07 | 3.722 | 1.896 |  |
| Itgb3 | na | 0.0009174 | 0.003736 | 1.702 | 0.7669 | 0.000282 | 0.001504 | 2.035 | 1.025 |  |
| Rbm3 | na | 0.006398 | 0.01627 | 1.442 | 0.5281 | 1.492e-05 | 0.00015 | 2.242 | 1.165 |  |
| Nek6 | na | 0.002924 | 0.008648 | 0.6107 | -0.7114 | 3.904e-05 | 0.0003166 | 0.405 | -1.304 |  |
| Nfkbia | na | 2.018e-06 | 4.137e-05 | 2.23 | 1.157 | 0.06998 | 0.1417 | 1.317 | 0.3974 |  |
| Gm9958 | na | 0.00118 | 0.004504 | 1.682 | 0.7505 | 0.0002414 | 0.001338 | 2.068 | 1.048 |  |
| Acacb | na | 0.009387 | 0.02234 | 1.559 | 0.6402 | 9.303e-06 | 0.0001029 | 0.341 | -1.552 |  |
| Fasn | na | 0.0002262 | 0.001362 | 0.2267 | -2.141 | 0.001935 | 0.007453 | 0.2478 | -2.013 |  |
| Gbp2b | na | 0.008946 | 0.02151 | 0.6655 | -0.5875 | 1.195e-05 | 0.0001269 | 0.3826 | -1.386 |  |
| B230340J04Rik | na | 0.002673 | 0.008036 | 1.71 | 0.7737 | 5.25e-05 | 0.0003926 | 2.579 | 1.367 |  |
| 8430408G22Rik | na | 2.368e-09 | 3.349e-07 | 14.6 | 3.868 | 0.6945 | 0.7805 | 0.8833 | -0.1791 |  |
| Eps8l2 | na | 0.02504 | 0.04923 | 0.7992 | -0.3233 | 2.499e-06 | 3.834e-05 | 0.4911 | -1.026 |  |
| Rorc | na | 4.007e-07 | 1.237e-05 | 2.108 | 1.076 | 0.2275 | 0.3571 | 1.161 | 0.2148 |  |
| Tmem180 | na | 0.002656 | 0.007995 | 0.6474 | -0.6273 | 6.147e-05 | 0.0004509 | 0.4688 | -1.093 |  |
| AI607873 | na | 3.278e-05 | 0.0003374 | 2.321 | 1.215 | 0.01355 | 0.03683 | 1.66 | 0.7308 |  |
| Ky | na | 2.134e-08 | 1.335e-06 | 0.3683 | -1.441 | 0.4507 | 0.5793 | 0.9031 | -0.1471 |  |
| Cxx1b | na | 0.001889 | 0.00634 | 0.6206 | -0.6882 | 0.0001234 | 0.0007784 | 0.4727 | -1.081 |  |
| Rrm2 | na | 0.007314 | 0.01812 | 1.44 | 0.5258 | 1.808e-05 | 0.0001729 | 2.242 | 1.165 |  |
| Cntrl | na | 0.001608 | 0.00563 | 1.694 | 0.7605 | 0.0001813 | 0.001046 | 2.19 | 1.131 |  |
| Gpt | na | 2.917e-08 | 1.734e-06 | 0.3434 | -1.542 | 0.4891 | 0.6157 | 1.107 | 0.1471 |  |
| Shank2 | na | 0.01172 | 0.0268 | 0.7146 | -0.4848 | 1.026e-05 | 0.0001119 | 0.4326 | -1.209 |  |
| Adra1b | na | 0.01966 | 0.04058 | 0.7249 | -0.4641 | 5.762e-06 | 7.322e-05 | 0.4 | -1.322 |  |
| Cyp2d22 | na | 3.7e-07 | 1.173e-05 | 2.25 | 1.17 | 0.2887 | 0.423 | 1.151 | 0.2034 |  |
| Sesn1 | na | 3.112e-10 | 7.495e-08 | 2.528 | 1.338 | 0.9027 | 0.9351 | 0.9882 | -0.0171 |  |
| Prnd | na | 2.612e-07 | 8.873e-06 | 0.2336 | -2.098 | 0.3428 | 0.4784 | 0.8023 | -0.3178 |  |
| Gabarapl1 | na | 2.535e-09 | 3.349e-07 | 2.657 | 1.41 | 0.8933 | 0.9278 | 1.016 | 0.02236 |  |
| Slc43a1 | na | 4.236e-08 | 2.289e-06 | 3.162 | 1.661 | 0.6241 | 0.7293 | 1.083 | 0.1146 |  |
| Fam71e1 | na | 0.003938 | 0.01083 | 1.446 | 0.5318 | 3.942e-05 | 0.0003166 | 2.018 | 1.013 |  |
| Slco1a1 | na | 0.007226 | 0.01797 | 0.6757 | -0.5655 | 2.324e-05 | 0.0002093 | 0.4278 | -1.225 |  |
| Igfbp5 | na | 1.895e-05 | 0.0002264 | 0.2092 | -2.257 | 0.02988 | 0.0707 | 0.4595 | -1.122 |  |
| Car4 | na | 3.598e-07 | 1.156e-05 | 2.36 | 1.239 | 0.3608 | 0.4977 | 1.137 | 0.1846 |  |
| C730034F03Rik | na | 0.002126 | 0.00686 | 1.599 | 0.6771 | 0.0001295 | 0.0008104 | 2.105 | 1.074 |  |
| Foxi1 | na | 0.008903 | 0.02143 | 1.494 | 0.5792 | 2.081e-05 | 0.000193 | 2.474 | 1.307 |  |
| Dgka | na | 0.0004772 | 0.002288 | 2.549 | 1.35 | 0.001451 | 0.005898 | 2.667 | 1.415 |  |
| Cpt1b | na | 0.05394 | 0.09295 | 1.255 | 0.3277 | 2.185e-06 | 3.441e-05 | 2.365 | 1.242 |  |
| Elovl6 | na | 7.018e-05 | 0.0005849 | 0.3605 | -1.472 | 0.01344 | 0.03657 | 0.519 | -0.9463 |  |
| Shq1 | na | 3e-04 | 0.001651 | 2.092 | 1.065 | 0.002458 | 0.008962 | 2 | 0.9997 |  |
| Cyp2a22 | na | 0.004775 | 0.01273 | 2.222 | 1.152 | 4.039e-05 | 0.0003202 | 4.75 | 2.248 |  |
| Cyp1b1 | na | 2.841e-05 | 0.0003015 | 4.566 | 2.191 | 0.02457 | 0.05968 | 2.25 | 1.17 |  |
| Agpat9 | na | 0.02846 | 0.05467 | 1.499 | 0.584 | 6.127e-06 | 7.628e-05 | 3.432 | 1.779 |  |
| Crcp | na | 0.04727 | 0.08302 | 1.305 | 0.3839 | 2.904e-06 | 4.289e-05 | 2.603 | 1.38 |  |
| Fcamr | na | 0.002274 | 0.007185 | 1.915 | 0.9376 | 0.0001348 | 0.0008326 | 2.817 | 1.494 |  |
| Gstm4 | na | 0.00107 | 0.004206 | 0.5501 | -0.8622 | 0.0006283 | 0.002906 | 0.4727 | -1.081 |  |
| Gm23462 | na | 0.001312 | 0.004875 | 2.239 | 1.163 | 0.0004762 | 0.002311 | 2.9 | 1.536 |  |
| Gstp1 | na | 0.004704 | 0.0126 | 0.5841 | -0.7758 | 4.515e-05 | 0.0003475 | 0.3545 | -1.496 |  |
| Dpyd | na | 1.222e-05 | 0.000165 | 2.028 | 1.02 | 0.0737 | 0.1478 | 1.315 | 0.3952 |  |
| Usp3 | na | 1.591e-07 | 6.102e-06 | 2.126 | 1.088 | 0.6833 | 0.7735 | 1.048 | 0.06794 |  |
| Pnpla7 | na | 1.502e-08 | 1.152e-06 | 2.635 | 1.398 | 0.9833 | 0.9891 | 1.003 | 0.003844 |  |
| Xkr9 | na | 0.01332 | 0.02983 | 0.7511 | -0.4129 | 1.772e-05 | 0.0001706 | 0.4976 | -1.007 |  |
| 1700039E15Rik | na | 0.1242 | 0.1863 | 1.148 | 0.1997 | 5.684e-07 | 1.175e-05 | 2.086 | 1.061 |  |
| Gm9992 | na | 4.986e-05 | 0.0004596 | 2.247 | 1.168 | 0.02036 | 0.05145 | 1.599 | 0.6771 |  |
| Serpina9 | na | 0.00675 | 0.01706 | 0.6944 | -0.5261 | 3.922e-05 | 0.0003166 | 0.4727 | -1.081 |  |
| Slc41a3 | na | 0.9058 | 0.9325 | 1.017 | 0.02456 | 5.854e-09 | 3.663e-07 | 5.056 | 2.338 |  |
| Prkag3 | na | 1.125e-06 | 2.597e-05 | 0.3394 | -1.559 | 0.338 | 0.4742 | 0.8331 | -0.2635 |  |
| Unc5a | na | 2.339e-05 | 0.0002624 | 0.4833 | -1.049 | 0.03956 | 0.08994 | 0.7075 | -0.4991 |  |
| Lrrc38 | na | 1.69e-06 | 3.653e-05 | 0.429 | -1.221 | 0.3056 | 0.4406 | 0.8541 | -0.2275 |  |
| Acnat2 | na | 0.03833 | 0.06985 | 1.438 | 0.5239 | 6.044e-06 | 7.565e-05 | 3.251 | 1.701 |  |
| Inmt | na | 1.92e-05 | 0.0002283 | 3.437 | 1.781 | 0.06744 | 0.1378 | 0.6018 | -0.7326 |  |
| Itgb6 | na | 2.776e-06 | 5.324e-05 | 0.4531 | -1.142 | 0.2567 | 0.3859 | 0.8439 | -0.2449 |  |
| Adgrg2 | na | 0.000363 | 0.001888 | 2.334 | 1.223 | 0.003128 | 0.01097 | 2.196 | 1.135 |  |
| Gm826 | na | 0.003323 | 0.009544 | 1.807 | 0.8538 | 0.000105 | 0.0006892 | 2.751 | 1.46 |  |
| Ptprs | na | 1.406e-06 | 3.096e-05 | 0.4547 | -1.137 | 0.3699 | 0.5076 | 0.8812 | -0.1824 |  |
| C77080 | na | 0.00701 | 0.01751 | 0.6953 | -0.5243 | 4.691e-05 | 0.0003593 | 0.4773 | -1.067 |  |
| Gm34985 | na | 0.004077 | 0.01114 | 2.123 | 1.086 | 7.495e-05 | 0.0005258 | 3.907 | 1.966 |  |
| Serpina6 | na | 0.03356 | 0.06245 | 0.7723 | -0.3727 | 8.382e-06 | 9.646e-05 | 0.4516 | -1.147 |  |
| Bhlhe40 | na | 2.322e-05 | 0.0002617 | 0.3972 | -1.332 | 0.07062 | 0.1428 | 0.6834 | -0.5493 |  |
| Tsku | na | 0.007315 | 0.01812 | 1.87 | 0.9028 | 4.737e-05 | 0.0003598 | 3.6 | 1.848 |  |
| Vwa3a | na | 3.038e-06 | 5.688e-05 | 2.283 | 1.191 | 0.3133 | 0.4491 | 1.171 | 0.2276 |  |
| Col3a1 | na | 0.0003547 | 0.001866 | 0.4469 | -1.162 | 0.00409 | 0.01376 | 0.487 | -1.038 |  |
| Dsg1a | na | 0.02095 | 0.0428 | 0.7687 | -0.3796 | 1.683e-05 | 0.0001644 | 0.4976 | -1.007 |  |
| AI464131 | na | 0.0002728 | 0.001559 | 0.4701 | -1.089 | 0.006136 | 0.0191 | 0.5374 | -0.8958 |  |
| Gdf1 | na | 1.419e-05 | 0.0001795 | 0.4711 | -1.086 | 0.1306 | 0.2319 | 0.7817 | -0.3554 |  |
| Gm12653 | na | 0.000997 | 0.003985 | 0.5281 | -0.921 | 0.001321 | 0.005501 | 0.481 | -1.056 |  |
| Rell1 | na | 0.003725 | 0.01029 | 0.6609 | -0.5974 | 0.000102 | 0.0006756 | 0.4863 | -1.04 |  |
| Serpina12 | na | 0.01353 | 0.03021 | 0.6447 | -0.6332 | 3.487e-05 | 0.000292 | 0.362 | -1.466 |  |
| Maff | na | 2.604e-05 | 0.0002841 | 2.118 | 1.083 | 0.08343 | 0.1621 | 0.7421 | -0.4303 |  |
| Tram2 | na | 0.0502 | 0.08764 | 1.298 | 0.3767 | 8.252e-06 | 9.619e-05 | 2.413 | 1.271 |  |
| Bhmt | na | 0.0395 | 0.07165 | 1.344 | 0.4261 | 9.898e-06 | 0.000109 | 2.533 | 1.341 |  |
| Baiap2 | na | 0.03115 | 0.05898 | 1.34 | 0.4222 | 1.357e-05 | 0.0001415 | 2.349 | 1.232 |  |
| Arrdc3 | na | 1.983e-07 | 7.038e-06 | 4.081 | 2.029 | 0.9917 | 0.9942 | 1.002 | 0.003273 |  |
| Csmd1 | na | 0.0005171 | 0.00243 | 2.011 | 1.008 | 0.003219 | 0.01121 | 1.953 | 0.9654 |  |
| Dusp10 | na | 0.0002757 | 0.001565 | 0.4272 | -1.227 | 0.007197 | 0.02205 | 0.5045 | -0.9871 |  |
| Slc1a4 | na | 0.02202 | 0.04441 | 0.7257 | -0.4625 | 2.054e-05 | 0.0001923 | 0.4296 | -1.219 |  |
| Sntg2 | na | 0.007945 | 0.01938 | 0.6986 | -0.5174 | 6.195e-05 | 0.0004519 | 0.4836 | -1.048 |  |
| Cyp2c29 | na | 0.01147 | 0.02635 | 0.6754 | -0.5662 | 4.38e-05 | 0.0003426 | 0.4202 | -1.251 |  |
| Erbb4 | na | 0.003717 | 0.01029 | 0.6159 | -0.6992 | 0.000123 | 0.0007784 | 0.4365 | -1.196 |  |
| Slc37a4 | na | 0.8012 | 0.8493 | 1.028 | 0.04009 | 1.182e-07 | 3.346e-06 | 2.803 | 1.487 |  |
| Rnf19b | na | 0.06395 | 0.1072 | 0.8241 | -0.2791 | 7.481e-06 | 8.807e-05 | 0.4972 | -1.008 |  |
| LOC105247125 | na | 8.346e-05 | 0.0006594 | 4.031 | 2.011 | 0.03595 | 0.08296 | 2.126 | 1.088 |  |
| Pptc7 | na | 0.3073 | 0.3922 | 1.142 | 0.1918 | 6.875e-07 | 1.351e-05 | 2.908 | 1.54 |  |
| Slc22a7 | na | 0.07459 | 0.1212 | 0.6991 | -0.5164 | 6.596e-06 | 8.06e-05 | 0.2565 | -1.963 |  |
| Spsb1 | na | 2.472e-06 | 4.937e-05 | 2.204 | 1.14 | 0.6527 | 0.7516 | 1.068 | 0.09483 |  |
| Il1r1 | na | 0.008165 | 0.01981 | 1.845 | 0.8836 | 7.329e-05 | 0.0005187 | 3.413 | 1.771 |  |
| Gm40902 | na | 0.0001016 | 0.0007527 | 2.646 | 1.404 | 0.02742 | 0.06532 | 1.763 | 0.818 |  |
| Anxa2 | na | 0.000277 | 0.001568 | 0.4087 | -1.291 | 0.009693 | 0.02829 | 0.5024 | -0.9932 |  |
| Zdhhc23 | na | 0.0003593 | 0.001874 | 2.595 | 1.376 | 0.006236 | 0.01938 | 2.235 | 1.16 |  |
| Meiob | na | 0.0137 | 0.0305 | 0.7112 | -0.4917 | 4.203e-05 | 0.000331 | 0.4591 | -1.123 |  |
| Abcb4 | na | 0.2019 | 0.2775 | 1.13 | 0.1761 | 1.938e-06 | 3.214e-05 | 2.063 | 1.045 |  |
| Stxbp4 | na | 5.799e-05 | 0.0005165 | 0.4887 | -1.033 | 0.0751 | 0.1503 | 0.7309 | -0.4523 |  |
| Lifr | na | 0.1542 | 0.2217 | 0.8498 | -0.2348 | 2.777e-06 | 4.127e-05 | 0.4326 | -1.209 |  |
| Gm16418 | na | 0.002268 | 0.007181 | 0.5016 | -0.9955 | 0.0004152 | 0.002057 | 0.3729 | -1.423 |  |
| Trabd2b | na | 3.521e-06 | 6.343e-05 | 0.3747 | -1.416 | 0.5376 | 0.6558 | 1.122 | 0.1658 |  |
| Nxpe3 | na | 1.975e-05 | 0.0002325 | 2.155 | 1.108 | 0.1876 | 0.3089 | 1.25 | 0.3223 |  |
| Fibin | na | 7.161e-06 | 0.0001071 | 2.497 | 1.32 | 0.3681 | 0.5057 | 1.181 | 0.2395 |  |
| Gm10309 | na | 3.072e-06 | 5.707e-05 | 2.449 | 1.292 | 0.6562 | 0.7538 | 1.078 | 0.108 |  |
| Baiap2l1 | na | 0.02228 | 0.04482 | 1.434 | 0.5199 | 2.72e-05 | 0.0002352 | 2.537 | 1.343 |  |
| Ciart | na | 0.08263 | 0.1319 | 1.388 | 0.4727 | 8.053e-06 | 9.434e-05 | 3.536 | 1.822 |  |
| Angptl1 | na | 9.25e-05 | 0.0007142 | 0.4178 | -1.259 | 0.05266 | 0.114 | 0.6462 | -0.63 |  |
| Stard4 | na | 0.01962 | 0.04054 | 0.647 | -0.6282 | 3.906e-05 | 0.0003166 | 0.3444 | -1.538 |  |
| Nr1i3 | na | 0.06644 | 0.1106 | 1.255 | 0.3279 | 1.047e-05 | 0.0001132 | 2.244 | 1.166 |  |
| Gngt1 | na | 0.01395 | 0.03095 | 0.7337 | -0.4467 | 5.246e-05 | 0.0003926 | 0.4986 | -1.004 |  |
| Slc18b1 | na | 1.242e-06 | 2.813e-05 | 0.4914 | -1.025 | 0.9765 | 0.9852 | 0.9963 | -0.005296 |  |
| Pld5 | na | 5.09e-06 | 8.464e-05 | 0.4972 | -1.008 | 0.6214 | 0.7276 | 0.9349 | -0.09707 |  |
| Slc17a9 | na | 0.001435 | 0.005217 | 1.848 | 0.8861 | 0.001634 | 0.006542 | 2.046 | 1.033 |  |
| Cntnap1 | na | 0.1801 | 0.253 | 1.196 | 0.2586 | 3.427e-06 | 4.965e-05 | 2.635 | 1.398 |  |
| Gata6 | na | 0.006012 | 0.01554 | 1.6 | 0.6779 | 0.0001333 | 0.0008273 | 2.347 | 1.231 |  |
| Lfng | na | 0.001788 | 0.0061 | 1.875 | 0.907 | 0.001037 | 0.004467 | 2.208 | 1.143 |  |
| Tirap | na | 0.001913 | 0.006362 | 0.5228 | -0.9357 | 0.0007847 | 0.003481 | 0.4275 | -1.226 |  |
| Tfpi2 | na | 0.01431 | 0.03151 | 0.7138 | -0.4864 | 5.423e-05 | 0.0004017 | 0.4681 | -1.095 |  |
| D230025D16Rik | na | 2.662e-06 | 5.147e-05 | 2.387 | 1.255 | 0.8495 | 0.9029 | 1.031 | 0.04413 |  |
| Sult1a1 | na | 0.0003525 | 0.001859 | 2.092 | 1.065 | 0.009964 | 0.02882 | 1.784 | 0.8351 |  |
| 3010026O09Rik | na | 0.003607 | 0.01007 | 0.5161 | -0.9544 | 0.000244 | 0.001343 | 0.3472 | -1.526 |  |
| Fam198a | na | 0.03521 | 0.06486 | 0.703 | -0.5084 | 2.49e-05 | 0.0002209 | 0.366 | -1.45 |  |
| Gm34595 | na | 0.0001934 | 0.001207 | 0.2819 | -1.827 | 0.0249 | 0.06024 | 0.4503 | -1.151 |  |
| Foxq1 | na | 0.04453 | 0.07899 | 0.5389 | -0.8918 | 2.204e-05 | 0.0001993 | 0.153 | -2.708 |  |
| 2310015D24Rik | na | 5.747e-05 | 0.0005138 | 0.361 | -1.47 | 0.1606 | 0.2742 | 0.7077 | -0.4988 |  |
| Clca3a1 | na | 0.0004721 | 0.002277 | 2.717 | 1.442 | 0.008368 | 0.02494 | 2.293 | 1.197 |  |
| BC005685 | na | 0.0001441 | 0.0009653 | 4.234 | 2.082 | 0.03964 | 0.08999 | 2.231 | 1.158 |  |
| Ccl2 | na | 0.0001949 | 0.001213 | 0.484 | -1.047 | 0.0287 | 0.06811 | 0.6409 | -0.6418 |  |
| Gk | na | 0.09778 | 0.1518 | 1.247 | 0.319 | 1.107e-05 | 0.0001191 | 2.398 | 1.262 |  |
| Slc15a2 | na | 0.0003586 | 0.001874 | 2.445 | 1.29 | 0.01359 | 0.03688 | 1.949 | 0.963 |  |
| Pgm3 | na | 0.01742 | 0.03682 | 1.423 | 0.5086 | 6.452e-05 | 0.0004678 | 2.245 | 1.167 |  |
| Cidec | na | 0.01683 | 0.03586 | 2.832 | 1.502 | 6.863e-05 | 0.0004931 | 10.61 | 3.407 |  |
| Gm6762 | na | 0.005542 | 0.01451 | 0.6534 | -0.614 | 0.000203 | 0.001158 | 0.48 | -1.059 |  |
| Olfml1 | na | 0.0004251 | 0.002102 | 0.4773 | -1.067 | 0.01086 | 0.03065 | 0.5582 | -0.8411 |  |
| Mid1ip1 | na | 0.01527 | 0.03313 | 0.5977 | -0.7425 | 8.042e-05 | 0.0005608 | 0.3224 | -1.633 |  |
| Gm7819 | na | 0.0008547 | 0.003578 | 2.164 | 1.114 | 0.005181 | 0.01667 | 2.079 | 1.056 |  |
| Spryd3 | na | 0.7362 | 0.7943 | 1.042 | 0.05964 | 1.43e-06 | 2.5e-05 | 2.632 | 1.396 |  |
| D930015E06Rik | na | 5.221e-05 | 0.0004739 | 2.049 | 1.035 | 0.2137 | 0.3411 | 1.237 | 0.3071 |  |
| Ptges | na | 7.459e-06 | 0.0001109 | 0.4341 | -1.204 | 0.8414 | 0.8976 | 1.034 | 0.04824 |  |
| Lce6a | na | 0.01344 | 0.03007 | 0.7067 | -0.5008 | 0.0001038 | 0.0006838 | 0.4816 | -1.054 |  |
| H2-Q10 | na | 0.04117 | 0.0739 | 0.7815 | -0.3557 | 3.622e-05 | 0.0003012 | 0.4938 | -1.018 |  |
| Nrg4 | na | 0.02245 | 0.04511 | 1.658 | 0.7293 | 6.163e-05 | 0.0004509 | 3.389 | 1.761 |  |
| Got1 | na | 0.003436 | 0.00975 | 1.641 | 0.715 | 0.0004685 | 0.002278 | 2.091 | 1.064 |  |
| Col14a1 | na | 0.002489 | 0.007598 | 0.5955 | -0.7478 | 0.0007848 | 0.003481 | 0.4959 | -1.012 |  |
| Plcd4 | na | 0.00014 | 0.0009485 | 0.3758 | -1.412 | 0.08081 | 0.1591 | 0.6352 | -0.6548 |  |
| Slc25a10 | na | 9.375e-05 | 0.0007207 | 0.3732 | -1.422 | 0.1444 | 0.251 | 1.439 | 0.5254 |  |
| Doc2b | na | 6.521e-06 | 0.0001014 | 2.369 | 1.244 | 0.9385 | 0.9579 | 1.013 | 0.019 |  |
| Bhlhe41 | na | 5.959e-05 | 0.0005248 | 0.3338 | -1.583 | 0.2354 | 0.3633 | 0.7308 | -0.4525 |  |
| 2900026A02Rik | na | 0.05262 | 0.09112 | 0.7939 | -0.3329 | 3.075e-05 | 0.0002609 | 0.4918 | -1.024 |  |
| Rhbdd2 | na | 0.2082 | 0.2841 | 1.143 | 0.1934 | 7.088e-06 | 8.428e-05 | 2.082 | 1.058 |  |
| Trpv2 | na | 9.781e-05 | 0.0007376 | 2.532 | 1.34 | 0.1394 | 0.2434 | 1.418 | 0.5035 |  |
| Slc7a2 | na | 0.0006687 | 0.00297 | 2.372 | 1.246 | 0.009812 | 0.02856 | 2.066 | 1.047 |  |
| Traj7 | na | 0.002712 | 0.008143 | 0.4272 | -1.227 | 0.0007292 | 0.003272 | 0.3095 | -1.692 |  |
| Arl4d | na | 0.0003481 | 0.001848 | 3.331 | 1.736 | 0.02039 | 0.05147 | 2.302 | 1.203 |  |
| Grtp1 | na | 9.815e-06 | 0.0001406 | 0.4665 | -1.1 | 0.8883 | 0.9253 | 0.9783 | -0.03158 |  |
| Gdap1 | na | 2.978e-05 | 0.0003133 | 0.4816 | -1.054 | 0.3851 | 0.5197 | 0.8667 | -0.2064 |  |
| Slc7a8 | na | 0.01973 | 0.04069 | 1.416 | 0.502 | 8.905e-05 | 0.000606 | 0.4519 | -1.146 |  |
| Fbxo36 | na | 0.2623 | 0.3451 | 1.168 | 0.2237 | 6.786e-06 | 8.233e-05 | 2.61 | 1.384 |  |
| Emp1 | na | 0.0001481 | 0.0009837 | 0.4084 | -1.292 | 0.08888 | 0.1703 | 0.6665 | -0.5854 |  |
| Ncf1 | na | 0.0003222 | 0.00174 | 2.12 | 1.084 | 0.02338 | 0.05726 | 1.655 | 0.7264 |  |
| Synpo2l | na | 0.0001568 | 0.00103 | 0.4948 | -1.015 | 0.08326 | 0.1621 | 0.7212 | -0.4716 |  |
| Txnip | na | 0.005159 | 0.01362 | 1.631 | 0.706 | 0.0003415 | 0.001758 | 2.215 | 1.147 |  |
| N4bp2l1 | na | 0.001871 | 0.006302 | 1.965 | 0.9742 | 0.002254 | 0.008401 | 2.184 | 1.127 |  |
| Stard5 | na | 0.0008183 | 0.00345 | 2.222 | 1.152 | 0.009695 | 0.02829 | 1.986 | 0.99 |  |
| Rasa3 | na | 5.175e-05 | 0.0004733 | 2.224 | 1.153 | 0.3525 | 0.4889 | 1.192 | 0.2533 |  |
| 5430403G16Rik | na | 0.003502 | 0.009863 | 0.5866 | -0.7696 | 0.0005954 | 0.002771 | 0.4601 | -1.12 |  |
| Cyp26a1 | na | 0.1289 | 0.1917 | 0.583 | -0.7784 | 1.81e-05 | 0.0001729 | 0.1051 | -3.25 |  |
| Lrrc39 | na | 1.097e-05 | 0.0001505 | 0.3787 | -1.401 | 0.9754 | 0.9845 | 1.006 | 0.008909 |  |
| Slc4a10 | na | 0.000418 | 0.002076 | 0.4444 | -1.17 | 0.02122 | 0.05306 | 0.5666 | -0.8196 |  |
| Cyp2c38 | na | 0.08622 | 0.1362 | 1.332 | 0.4133 | 2.423e-05 | 0.0002166 | 2.788 | 1.479 |  |
| Grem2 | na | 0.0002165 | 0.00132 | 0.4281 | -1.224 | 0.08026 | 0.1585 | 0.6633 | -0.5922 |  |
| Tbc1d8 | na | 0.02821 | 0.05436 | 1.471 | 0.5564 | 8.705e-05 | 0.0005966 | 2.572 | 1.363 |  |
| Aldoc | na | 0.01158 | 0.02653 | 0.6158 | -0.6994 | 0.0001769 | 0.001026 | 0.3877 | -1.367 |  |
| Sec14l5 | na | 1.237e-05 | 0.0001653 | 0.4538 | -1.14 | 0.9651 | 0.9766 | 1.007 | 0.01037 |  |
| Ly6a | na | 0.1909 | 0.2652 | 0.8577 | -0.2215 | 1.495e-05 | 0.00015 | 0.4672 | -1.098 |  |
| Acaca | na | 0.0007663 | 0.003277 | 0.4213 | -1.247 | 0.01456 | 0.03912 | 0.5016 | -0.9953 |  |
| BC023105 | na | 0.004952 | 0.01314 | 0.4948 | -1.015 | 0.0005213 | 0.002494 | 0.3368 | -1.57 |  |
| Gm9909 | na | 2.493e-05 | 0.0002732 | 0.4302 | -1.217 | 0.7824 | 0.8539 | 1.053 | 0.07403 |  |
| Gm4841 | na | 0.00111 | 0.004285 | 0.4404 | -1.183 | 0.007534 | 0.02279 | 0.4688 | -1.093 |  |
| Rgs2 | na | 3.118e-05 | 0.0003249 | 3.475 | 1.797 | 0.7021 | 0.7864 | 0.8986 | -0.1543 |  |
| Pglyrp1 | na | 0.0005621 | 0.002575 | 2.099 | 1.07 | 0.02015 | 0.05098 | 1.717 | 0.7803 |  |
| Pik3r5 | na | 0.0003869 | 0.001966 | 2.317 | 1.212 | 0.0372 | 0.08564 | 1.685 | 0.7524 |  |
| Gm5424 | na | 0.0003916 | 0.001977 | 2.175 | 1.121 | 0.03713 | 0.08556 | 1.621 | 0.6969 |  |
| Glns-ps1 | na | 0.000125 | 0.000882 | 2.066 | 1.047 | 0.2548 | 0.3845 | 1.237 | 0.3065 |  |
| Nceh1 | na | 0.002792 | 0.008341 | 1.803 | 0.8503 | 0.001554 | 0.006263 | 2.116 | 1.081 |  |
| Nipal1 | na | 0.04207 | 0.07533 | 1.597 | 0.6752 | 7.787e-05 | 0.0005446 | 3.531 | 1.82 |  |
| Marcks | na | 0.0005507 | 0.002538 | 0.49 | -1.029 | 0.02469 | 0.05991 | 0.6068 | -0.7206 |  |
| Fstl1 | na | 0.002081 | 0.006751 | 0.5151 | -0.9571 | 0.002873 | 0.01015 | 0.4704 | -1.088 |  |
| Bcl6b | na | 0.0001399 | 0.0009485 | 0.4717 | -1.084 | 0.2306 | 0.3603 | 1.264 | 0.3378 |  |
| Ighv14-2 | na | 0.001421 | 0.005199 | 7.392 | 2.886 | 0.007337 | 0.0224 | 6.746 | 2.754 |  |
| Slc25a37 | na | 0.2282 | 0.3069 | 0.8395 | -0.2524 | 1.735e-05 | 0.0001684 | 0.3931 | -1.347 |  |
| Pex11a | na | 0.7854 | 0.8353 | 1.029 | 0.04057 | 6.609e-06 | 8.06e-05 | 2.072 | 1.051 |  |
| Abi3 | na | 0.0002026 | 0.001251 | 2.135 | 1.094 | 0.156 | 0.268 | 1.339 | 0.4208 |  |
| Tmem140 | na | 0.0002026 | 0.001251 | 2.163 | 1.113 | 0.1695 | 0.2864 | 0.7506 | -0.4139 |  |
| Adam23 | na | 0.02898 | 0.05549 | 1.584 | 0.6636 | 0.000123 | 0.0007784 | 3 | 1.585 |  |
| Gm379 | na | 0.01081 | 0.02518 | 0.6513 | -0.6185 | 0.0003496 | 0.001792 | 0.4601 | -1.12 |  |
| LOC100043921 | na | 0.001244 | 0.004711 | 5.594 | 2.484 | 0.01088 | 0.03065 | 4.582 | 2.196 |  |
| mt-Tf | na | 0.0009099 | 0.003718 | 0.4453 | -1.167 | 0.01795 | 0.0466 | 0.5302 | -0.9153 |  |
| Mir1a-1 | na | 0.0001104 | 0.0008053 | 0.4826 | -1.051 | 0.3629 | 0.4997 | 0.8459 | -0.2414 |  |
| Cml5 | na | 0.02946 | 0.05627 | 0.5962 | -0.7462 | 0.000129 | 0.0008094 | 0.2912 | -1.78 |  |
| Slc25a47 | na | 0.2108 | 0.2869 | 1.184 | 0.244 | 2.143e-05 | 0.0001966 | 2.347 | 1.231 |  |
| Cyp4a14 | na | 0.08592 | 0.1361 | 1.401 | 0.4862 | 4.751e-05 | 0.0003598 | 3.125 | 1.644 |  |
| Cxcr4 | na | 0.001099 | 0.004263 | 3.145 | 1.653 | 0.01486 | 0.0397 | 2.581 | 1.368 |  |
| Treh | na | 0.2644 | 0.3472 | 1.132 | 0.1783 | 1.981e-05 | 0.0001871 | 2.028 | 1.02 |  |
| Adh6-ps1 | na | 0.03131 | 0.05917 | 0.6134 | -0.7051 | 0.0001233 | 0.0007784 | 0.3054 | -1.711 |  |
| 2010003K11Rik | na | 0.948 | 0.9618 | 0.9898 | -0.01481 | 6.996e-06 | 8.36e-05 | 3.023 | 1.596 |  |
| Med4 | na | 0.007401 | 0.01828 | 1.549 | 0.6318 | 0.0005722 | 0.002684 | 2.036 | 1.026 |  |
| Cxcl13 | na | 0.0007169 | 0.003117 | 9.6 | 3.263 | 0.02652 | 0.06364 | 4.979 | 2.316 |  |
| Apol9b | na | 0.01942 | 0.04016 | 0.7076 | -0.4989 | 0.0002293 | 0.001287 | 0.486 | -1.041 |  |
| Gm5558 | na | 0.001298 | 0.004846 | 0.4918 | -1.024 | 0.0136 | 0.03688 | 0.5444 | -0.8773 |  |
| Sult1d1 | na | 0.04121 | 0.0739 | 1.559 | 0.6407 | 0.0001123 | 0.0007243 | 3.169 | 1.664 |  |
| Slamf7 | na | 0.0006614 | 0.002951 | 2.41 | 1.269 | 0.03738 | 0.08587 | 1.779 | 0.8313 |  |
| Pfkfb1 | na | 0.2913 | 0.3746 | 1.13 | 0.176 | 2.074e-05 | 0.000193 | 2.088 | 1.062 |  |
| Nmrk2 | na | 0.0001805 | 0.001157 | 0.1878 | -2.413 | 0.3133 | 0.4491 | 0.6398 | -0.6443 |  |
| Dbp | na | 9.832e-05 | 0.0007376 | 0.2269 | -2.14 | 0.5558 | 0.6709 | 0.8049 | -0.3131 |  |
| Hsd17b6 | na | 0.03056 | 0.05804 | 0.7211 | -0.4717 | 0.0001577 | 0.0009399 | 0.4623 | -1.113 |  |
| Ms4a6c | na | 0.0001786 | 0.001148 | 2.894 | 1.533 | 0.3401 | 0.4752 | 1.307 | 0.3863 |  |
| Atl2 | na | 0.09158 | 0.1432 | 1.248 | 0.3197 | 6.245e-05 | 0.0004541 | 2.108 | 1.076 |  |
| Omd | na | 0.006276 | 0.01601 | 1.831 | 0.8723 | 0.001097 | 0.0047 | 2.442 | 1.288 |  |
| 1810010H24Rik | na | 0.6553 | 0.7266 | 0.945 | -0.08168 | 1.531e-05 | 0.0001522 | 2.31 | 1.208 |  |
| Hspa12a | na | 0.0001541 | 0.001021 | 0.462 | -1.114 | 0.3725 | 0.5097 | 1.196 | 0.2586 |  |
| Pmf1 | na | 0.01652 | 0.0353 | 0.6634 | -0.592 | 0.0003708 | 0.001888 | 0.4525 | -1.144 |  |
| Trbv20 | na | 0.001433 | 0.005217 | 8.067 | 3.012 | 0.01537 | 0.04075 | 5.877 | 2.555 |  |
| Erc2 | na | 0.06199 | 0.1048 | 0.7832 | -0.3526 | 0.0001114 | 0.0007238 | 0.4962 | -1.011 |  |
| Slc13a2 | na | 0.02405 | 0.04762 | 0.663 | -0.5929 | 0.0002644 | 0.001429 | 0.4147 | -1.27 |  |
| Hamp2 | na | 0.01407 | 0.0311 | 0.5972 | -0.7438 | 0.0004815 | 0.002332 | 0.389 | -1.362 |  |
| Stap1 | na | 0.003452 | 0.009772 | 4.15 | 2.053 | 0.00243 | 0.008904 | 5.816 | 2.54 |  |
| Gm23264 | na | 0.001427 | 0.005212 | 2.068 | 1.048 | 0.01825 | 0.04728 | 1.818 | 0.8625 |  |
| Slc41a2 | na | 0.1595 | 0.2282 | 0.7507 | -0.4136 | 4.826e-05 | 0.0003643 | 0.3021 | -1.727 |  |
| Fzd7 | na | 0.007748 | 0.01899 | 0.5883 | -0.7653 | 0.0009871 | 0.004276 | 0.4414 | -1.18 |  |
| Prox1 | na | 0.004212 | 0.01148 | 2.898 | 1.535 | 0.001818 | 0.007087 | 4.059 | 2.021 |  |
| Fgfr3 | na | 0.07454 | 0.1212 | 1.262 | 0.3352 | 0.0001052 | 0.0006892 | 0.4948 | -1.015 |  |
| Tppp3 | na | 0.0005435 | 0.00251 | 0.4969 | -1.009 | 0.1061 | 0.1968 | 0.7114 | -0.4913 |  |
| Gm25357 | na | 0.001437 | 0.005217 | 0.4979 | -1.006 | 0.01986 | 0.05035 | 0.5681 | -0.8158 |  |
| Irf4 | na | 0.0001005 | 0.0007492 | 4.42 | 2.144 | 0.8746 | 0.9155 | 1.06 | 0.08383 |  |
| Cyp4a32 | na | 0.05869 | 0.0999 | 1.391 | 0.4761 | 0.0001381 | 0.0008464 | 2.497 | 1.32 |  |
| Zfand4 | na | 0.6831 | 0.7527 | 0.9497 | -0.07448 | 2.187e-05 | 0.0001993 | 0.4441 | -1.171 |  |
| Ces2c | na | 0.07629 | 0.1237 | 0.6861 | -0.5435 | 0.0001175 | 0.0007511 | 0.3209 | -1.64 |  |
| Dapp1 | na | 0.001547 | 0.005491 | 2.495 | 1.319 | 0.01954 | 0.0497 | 2.117 | 1.082 |  |
| Gm22887 | na | 0.0186 | 0.0387 | 1.539 | 0.6224 | 0.0005222 | 0.002494 | 2.277 | 1.187 |  |
| Gfra1 | na | 0.4361 | 0.5235 | 1.109 | 0.1491 | 3.777e-05 | 0.0003097 | 2.249 | 1.169 |  |
| Slc35g1 | na | 0.27 | 0.353 | 1.141 | 0.1902 | 4.465e-05 | 0.0003452 | 2.041 | 1.029 |  |
| Fgf1 | na | 0.02828 | 0.05441 | 1.389 | 0.4742 | 0.000373 | 0.001895 | 2.02 | 1.014 |  |
| Foxp1 | na | 0.0006108 | 0.002746 | 2.152 | 1.106 | 0.141 | 0.246 | 1.408 | 0.4939 |  |
| Ighv5-12 | na | 0.001774 | 0.00607 | 6.182 | 2.628 | 0.01838 | 0.04735 | 4.643 | 2.215 |  |
| Kcnb1 | na | 0.0004719 | 0.002277 | 0.45 | -1.152 | 0.2204 | 0.349 | 0.7508 | -0.4135 |  |
| Gm4184 | na | 0.03394 | 0.06291 | 0.7084 | -0.4974 | 0.0003173 | 0.001649 | 0.4595 | -1.122 |  |
| Angptl4 | na | 0.002847 | 0.008442 | 2.187 | 1.129 | 0.005759 | 0.01809 | 2.321 | 1.215 |  |
| Ms4a6b | na | 0.0008893 | 0.003684 | 3.681 | 1.88 | 0.09313 | 0.1772 | 2.008 | 1.006 |  |
| March1 | na | 0.001271 | 0.004782 | 2.028 | 1.02 | 0.04569 | 0.1013 | 1.609 | 0.6865 |  |
| Sfxn2 | na | 0.5234 | 0.6068 | 1.09 | 0.1246 | 3.956e-05 | 0.0003166 | 2.282 | 1.19 |  |
| Gm12407 | na | 0.001952 | 0.006411 | 5.487 | 2.456 | 0.01503 | 0.03998 | 4.5 | 2.17 |  |
| Gm2457 | na | 0.001952 | 0.006411 | 5.487 | 2.456 | 0.01503 | 0.03998 | 4.5 | 2.17 |  |
| Slc22a30 | na | 0.05481 | 0.09431 | 0.6949 | -0.5251 | 0.0002437 | 0.001343 | 0.3904 | -1.357 |  |
| Tubb4b-ps1 | na | 0.1511 | 0.218 | 0.8036 | -0.3155 | 9.783e-05 | 0.0006535 | 0.4317 | -1.212 |  |
| Lox | na | 0.0002982 | 0.001649 | 2.305 | 1.205 | 0.418 | 0.5513 | 1.206 | 0.27 |  |
| Loxl4 | na | 0.04042 | 0.07287 | 0.7226 | -0.4688 | 0.0003357 | 0.001735 | 0.4688 | -1.093 |  |
| Mir107 | na | 0.02476 | 0.04882 | 0.6618 | -0.5956 | 0.0005255 | 0.002504 | 0.4362 | -1.197 |  |
| Dpy19l1 | na | 0.07296 | 0.1193 | 1.288 | 0.3648 | 0.000219 | 0.001237 | 2.036 | 1.026 |  |
| A530099J19Rik | na | 0.001628 | 0.00566 | 2.357 | 1.237 | 0.03434 | 0.08006 | 1.883 | 0.9129 |  |
| Ccl19 | na | 0.002125 | 0.00686 | 5.315 | 2.41 | 0.01562 | 0.04132 | 4.398 | 2.137 |  |
| Gm6781 | na | 0.57 | 0.6517 | 0.9079 | -0.1394 | 4.44e-05 | 0.000345 | 0.3577 | -1.483 |  |
| Bach2 | na | 0.0002698 | 0.001553 | 2.521 | 1.334 | 0.69 | 0.7778 | 0.9045 | -0.1448 |  |
| Gm3579 | na | 0.09134 | 0.1429 | 1.709 | 0.773 | 0.0001718 | 0.001004 | 5.187 | 2.375 |  |
| Abcg1 | na | 0.0003114 | 0.001698 | 4.672 | 2.224 | 0.4796 | 0.6061 | 1.353 | 0.436 |  |
| Acat2 | na | 0.02418 | 0.0478 | 0.631 | -0.6644 | 0.000648 | 0.002975 | 0.4058 | -1.301 |  |
| Rassf2 | na | 0.001477 | 0.005338 | 2.86 | 1.516 | 0.04935 | 0.108 | 2.027 | 1.019 |  |
| Gpnmb | na | 0.001042 | 0.004123 | 2.417 | 1.273 | 0.1032 | 0.1925 | 1.593 | 0.6713 |  |
| Alas2 | na | 0.1059 | 0.1621 | 0.7158 | -0.4823 | 0.0001603 | 0.0009506 | 0.3387 | -1.562 |  |
| Atp2a3 | na | 0.0007321 | 0.003165 | 2.415 | 1.272 | 0.2073 | 0.3331 | 1.407 | 0.4922 |  |
| Atp10d | na | 0.002535 | 0.007699 | 2.483 | 1.312 | 0.01115 | 0.03134 | 2.393 | 1.259 |  |
| Fst | na | 0.4264 | 0.5158 | 0.8701 | -0.2007 | 7.115e-05 | 0.0005081 | 0.364 | -1.458 |  |
| Slc25a51 | na | 0.1452 | 0.2111 | 1.242 | 0.3122 | 0.0001326 | 0.0008255 | 2.218 | 1.149 |  |
| Adam11 | na | 0.4881 | 0.5747 | 0.8974 | -0.1561 | 5.411e-05 | 0.0004017 | 0.3964 | -1.335 |  |
| Gm16412 | na | 0.04631 | 0.08157 | 0.7104 | -0.4933 | 0.0003992 | 0.001994 | 0.4457 | -1.166 |  |
| Hbb-bt | na | 0.03016 | 0.05738 | 0.6923 | -0.5306 | 0.000589 | 0.002752 | 0.4675 | -1.097 |  |
| Hba-a1 | na | 0.03922 | 0.07125 | 0.649 | -0.6238 | 0.000491 | 0.002364 | 0.3816 | -1.39 |  |
| Cyp2d40 | na | 0.03226 | 0.06064 | 0.7005 | -0.5135 | 0.0005546 | 0.002612 | 0.472 | -1.083 |  |
| Inpp5d | na | 0.001247 | 0.004714 | 2.599 | 1.378 | 0.09153 | 0.175 | 1.705 | 0.7695 |  |
| Baz1a | na | 0.001601 | 0.005624 | 2.27 | 1.183 | 0.05466 | 0.1173 | 1.721 | 0.783 |  |
| Gm15401 | na | 0.2366 | 0.3163 | 1.236 | 0.3058 | 9.992e-05 | 0.0006656 | 2.704 | 1.435 |  |
| Tubb4b | na | 0.2781 | 0.3612 | 0.8383 | -0.2545 | 9.113e-05 | 0.0006174 | 0.4011 | -1.318 |  |
| Lin9 | na | 0.02298 | 0.04588 | 0.6448 | -0.6331 | 0.0008108 | 0.003577 | 0.4359 | -1.198 |  |
| Ralgps2 | na | 0.002523 | 0.007672 | 3.22 | 1.687 | 0.01488 | 0.03971 | 2.91 | 1.541 |  |
| Banp | na | 0.01414 | 0.03119 | 1.666 | 0.7361 | 0.00172 | 0.006772 | 2.253 | 1.172 |  |
| Gm32819 | na | 0.001012 | 0.004028 | 2.283 | 1.191 | 0.1649 | 0.2795 | 1.442 | 0.5281 |  |
| Cdh1 | na | 0.0578 | 0.0986 | 0.7159 | -0.4822 | 0.0004019 | 0.002 | 0.4347 | -1.202 |  |
| Vcan | na | 0.001 | 0.00399 | 0.4887 | -1.033 | 0.1706 | 0.2875 | 0.7315 | -0.451 |  |
| Gck | na | 0.09674 | 0.1506 | 0.6071 | -0.72 | 0.0002511 | 0.001375 | 0.2221 | -2.171 |  |
| Nr0b2 | na | 0.01477 | 0.03228 | 0.5655 | -0.8225 | 0.001821 | 0.007087 | 0.4033 | -1.31 |  |
| Bst1 | na | 0.002466 | 0.007537 | 2.926 | 1.549 | 0.01867 | 0.04784 | 2.558 | 1.355 |  |
| Mgll | na | 0.5332 | 0.6167 | 1.113 | 0.1551 | 8.419e-05 | 0.0005803 | 2.68 | 1.422 |  |
| Tfrc | na | 0.0005308 | 0.002465 | 0.429 | -1.221 | 0.4374 | 0.5688 | 1.212 | 0.2779 |  |
| Pcdhga7 | na | 0.01482 | 0.03236 | 0.6286 | -0.6699 | 0.002012 | 0.007705 | 0.4813 | -1.055 |  |
| Camk2b | na | 0.1923 | 0.2666 | 1.289 | 0.3663 | 0.0001573 | 0.0009398 | 2.811 | 1.491 |  |
| Utp14b | na | 0.01298 | 0.02917 | 0.6237 | -0.6811 | 0.002528 | 0.009178 | 0.4931 | -1.02 |  |
| Gm17167 | na | 0.01203 | 0.02742 | 1.922 | 0.9423 | 0.002596 | 0.009396 | 2.617 | 1.388 |  |
| Tmc7 | na | 0.1099 | 0.1674 | 0.7963 | -0.3287 | 0.0002836 | 0.001509 | 2.028 | 1.02 |  |
| Ifi30 | na | 0.00176 | 0.006031 | 2.511 | 1.328 | 0.07899 | 0.1568 | 1.748 | 0.806 |  |
| Hba-a2 | na | 0.04677 | 0.0822 | 0.6434 | -0.6363 | 0.0006635 | 0.003028 | 0.3711 | -1.43 |  |
| Car1 | na | 0.06228 | 0.105 | 0.6292 | -0.6685 | 0.0004872 | 0.002355 | 0.3151 | -1.666 |  |
| Atp5s | na | 0.1797 | 0.2527 | 1.247 | 0.3189 | 0.0001808 | 0.001046 | 2.369 | 1.244 |  |
| Ighv1-54 | na | 0.03833 | 0.06985 | 2.291 | 1.196 | 0.0008587 | 0.003774 | 5.649 | 2.498 |  |
| Gm42368 | na | 0.06937 | 0.1148 | 1.695 | 0.761 | 0.0005114 | 0.002457 | 3.84 | 1.941 |  |
| Paqr9 | na | 0.0005706 | 0.002604 | 0.4575 | -1.128 | 0.549 | 0.6657 | 0.8712 | -0.199 |  |
| Lsr | na | 0.122 | 0.1835 | 0.8016 | -0.3191 | 0.0003016 | 0.00159 | 0.4931 | -1.02 |  |
| Trim24 | na | 0.08295 | 0.1321 | 1.319 | 0.3995 | 0.0004444 | 0.002178 | 2.124 | 1.087 |  |
| Htra1 | na | 0.009208 | 0.02198 | 0.5869 | -0.7689 | 0.004335 | 0.01434 | 0.4948 | -1.015 |  |
| n-R5s193 | na | 0.02977 | 0.05677 | 1.566 | 0.6467 | 0.001397 | 0.005738 | 2.317 | 1.212 |  |
| Cited4 | na | 0.000694 | 0.003062 | 0.4284 | -1.223 | 0.6013 | 0.7128 | 0.8755 | -0.1918 |  |
| Lvrn | na | 0.0009834 | 0.00395 | 2.024 | 1.017 | 0.3575 | 0.4946 | 0.8157 | -0.2939 |  |
| Vcam1 | na | 0.003096 | 0.009112 | 2.608 | 1.383 | 0.02128 | 0.05316 | 2.323 | 1.216 |  |
| Igkv1-117 | na | 0.002606 | 0.007874 | 10.5 | 3.392 | 0.03396 | 0.07933 | 6.307 | 2.657 |  |
| Icam1 | na | 0.001901 | 0.00634 | 2.104 | 1.073 | 0.1001 | 0.1876 | 1.529 | 0.6124 |  |
| Gcnt1 | na | 0.001831 | 0.006211 | 2.049 | 1.035 | 0.1198 | 0.2178 | 1.468 | 0.5542 |  |
| P2ry10 | na | 0.002245 | 0.007128 | 2.387 | 1.255 | 0.06088 | 0.1286 | 1.792 | 0.8419 |  |
| Tmtc2 | na | 0.3174 | 0.4023 | 1.192 | 0.2531 | 0.0001736 | 0.001012 | 2.537 | 1.343 |  |
| Gadd45g | na | 0.003374 | 0.00962 | 2.562 | 1.357 | 0.01963 | 0.04987 | 2.338 | 1.225 |  |
| Slc25a53 | na | 0.003532 | 0.009905 | 2.065 | 1.046 | 0.01752 | 0.04568 | 1.958 | 0.9697 |  |
| Hbb-bs | na | 0.05584 | 0.09567 | 0.6828 | -0.5504 | 0.0008811 | 0.003845 | 0.4199 | -1.252 |  |
| Gm24707 | na | 0.0009213 | 0.003739 | 2.425 | 1.278 | 0.4222 | 0.5547 | 1.247 | 0.3188 |  |
| Sgpl1 | na | 0.000577 | 0.002614 | 2.035 | 1.025 | 0.8236 | 0.883 | 1.048 | 0.06706 |  |
| Gpr65 | na | 0.001304 | 0.004853 | 2.5 | 1.322 | 0.3046 | 0.4401 | 1.358 | 0.441 |  |
| Plcg2 | na | 0.002426 | 0.007456 | 2.612 | 1.385 | 0.05085 | 0.1107 | 1.974 | 0.9814 |  |
| Pole4 | na | 0.1738 | 0.2453 | 1.219 | 0.2857 | 0.0003166 | 0.001649 | 2.058 | 1.041 |  |
| Slc40a1 | na | 0.0008866 | 0.003679 | 2.036 | 1.026 | 0.5548 | 0.6704 | 1.138 | 0.1868 |  |
| Fam105a | na | 0.003129 | 0.009173 | 2.041 | 1.029 | 0.02539 | 0.0613 | 1.835 | 0.8761 |  |
| Cyp2c39 | na | 0.1034 | 0.1587 | 1.392 | 0.477 | 0.0005397 | 0.002557 | 2.569 | 1.361 |  |
| Plxnc1 | na | 0.002348 | 0.007318 | 2.355 | 1.236 | 0.06301 | 0.1318 | 1.774 | 0.8267 |  |
| Mest | na | 0.00214 | 0.006877 | 0.4014 | -1.317 | 0.08225 | 0.161 | 0.5708 | -0.809 |  |
| Asns | na | 0.06072 | 0.103 | 1.886 | 0.9152 | 0.001007 | 0.004354 | 4.275 | 2.096 |  |
| Aff3 | na | 0.002756 | 0.008254 | 2.283 | 1.191 | 0.04145 | 0.09361 | 1.866 | 0.9 |  |
| Uxt | na | 0.01989 | 0.04097 | 1.56 | 0.6412 | 0.003136 | 0.01098 | 2.01 | 1.007 |  |
| Nt5dc2 | na | 0.1686 | 0.2391 | 1.345 | 0.4274 | 0.0003874 | 0.001948 | 2.842 | 1.507 |  |
| Apol11b | na | 0.03181 | 0.05989 | 0.6472 | -0.6277 | 0.002002 | 0.007679 | 0.4528 | -1.143 |  |
| Ptbp3 | na | 0.003513 | 0.009863 | 2.18 | 1.124 | 0.02323 | 0.05707 | 1.984 | 0.9883 |  |
| Bdh2 | na | 0.4818 | 0.5686 | 0.8877 | -0.1719 | 0.0001969 | 0.001131 | 0.409 | -1.29 |  |
| mt-Tm | na | 0.06098 | 0.1033 | 0.462 | -1.114 | 0.001165 | 0.004929 | 0.1752 | -2.513 |  |
| Mir122 | na | 0.05158 | 0.08953 | 0.6085 | -0.7167 | 0.001442 | 0.005898 | 0.3504 | -1.513 |  |
| Il22ra2 | na | 0.00212 | 0.006859 | 2.83 | 1.501 | 0.125 | 0.2245 | 1.749 | 0.8062 |  |
| Creld2 | na | 0.2141 | 0.2908 | 1.227 | 0.2947 | 0.0003763 | 0.0019 | 2.235 | 1.16 |  |
| Ppip5k2 | na | 0.1211 | 0.1824 | 1.275 | 0.3508 | 0.0006685 | 0.003045 | 2.043 | 1.031 |  |
| Tlr7 | na | 0.001947 | 0.006411 | 2.423 | 1.277 | 0.167 | 0.2827 | 1.524 | 0.6083 |  |
| Coa6 | na | 0.1411 | 0.2062 | 1.39 | 0.475 | 0.0006385 | 0.00294 | 2.795 | 1.483 |  |
| Dok3 | na | 0.00344 | 0.00975 | 2.521 | 1.334 | 0.03911 | 0.08908 | 2.072 | 1.051 |  |
| Dexi | na | 0.02875 | 0.05509 | 0.6355 | -0.654 | 0.003156 | 0.01104 | 0.4659 | -1.102 |  |
| Igsf6 | na | 0.002165 | 0.006939 | 2.12 | 1.084 | 0.153 | 0.2634 | 1.455 | 0.5414 |  |
| Slco1a4 | na | 0.07279 | 0.1192 | 1.482 | 0.568 | 0.001434 | 0.005879 | 2.481 | 1.311 |  |
| Atf5 | na | 0.2259 | 0.3042 | 1.22 | 0.2871 | 0.0004529 | 0.002211 | 2.202 | 1.139 |  |
| Cd86 | na | 0.001877 | 0.006313 | 2.422 | 1.276 | 0.266 | 0.3951 | 1.398 | 0.4829 |  |
| Golt1a | na | 0.339 | 0.4252 | 1.167 | 0.2227 | 0.0003822 | 0.001926 | 2.215 | 1.147 |  |
| Pim1 | na | 0.07284 | 0.1192 | 0.7075 | -0.4991 | 0.001653 | 0.006584 | 0.456 | -1.133 |  |
| Hcar1 | na | 0.001107 | 0.00428 | 0.4538 | -1.14 | 0.7463 | 0.8243 | 1.084 | 0.1163 |  |
| C4bp-ps1 | na | 0.1361 | 0.2003 | 1.306 | 0.3851 | 0.0009726 | 0.004236 | 2.193 | 1.133 |  |
| Gm9870 | na | 0.07765 | 0.1254 | 0.7118 | -0.4904 | 0.001751 | 0.006871 | 0.4582 | -1.126 |  |
| AB124611 | na | 0.002954 | 0.008726 | 2.656 | 1.409 | 0.1016 | 0.1898 | 1.797 | 0.8456 |  |
| Stc1 | na | 0.001721 | 0.005914 | 2.066 | 1.047 | 0.4012 | 0.5348 | 1.226 | 0.2938 |  |
| Btg2 | na | 0.002493 | 0.0076 | 2.098 | 1.069 | 0.164 | 0.2786 | 0.6931 | -0.5289 |  |
| Ostn | na | 0.002377 | 0.00737 | 0.4931 | -1.02 | 0.1848 | 0.3058 | 0.7186 | -0.4768 |  |
| Syk | na | 0.002275 | 0.007185 | 2.725 | 1.446 | 0.2219 | 0.3508 | 1.534 | 0.6169 |  |
| Vtcn1 | na | 0.2341 | 0.3136 | 0.8351 | -0.2599 | 0.0006629 | 0.003028 | 0.4969 | -1.009 |  |
| Tubb5 | na | 0.8687 | 0.9055 | 1.027 | 0.0381 | 0.0002904 | 0.001535 | 0.4417 | -1.179 |  |
| Gm42035 | na | 0.1384 | 0.2028 | 1.443 | 0.5292 | 0.001269 | 0.005332 | 2.87 | 1.521 |  |
| Crlf3 | na | 0.003382 | 0.009632 | 2.101 | 1.071 | 0.1008 | 0.1887 | 1.576 | 0.6559 |  |
| Mir1a-2 | na | 0.001417 | 0.005192 | 0.4544 | -1.138 | 0.864 | 0.9113 | 0.9572 | -0.06312 |  |
| Sult1e1 | na | 0.1559 | 0.2237 | 1.556 | 0.6376 | 0.001246 | 0.005254 | 3.789 | 1.922 |  |
| Serpine1 | na | 0.001519 | 0.00544 | 2.9 | 1.536 | 0.7658 | 0.8419 | 1.109 | 0.1495 |  |
| Coro2a | na | 0.003493 | 0.009853 | 2.163 | 1.113 | 0.1095 | 0.2025 | 1.587 | 0.666 |  |
| Cd163 | na | 0.002308 | 0.00725 | 2.101 | 1.071 | 0.3238 | 0.4598 | 1.29 | 0.3671 |  |
| Mettl11b | na | 0.002791 | 0.008341 | 0.3653 | -1.453 | 0.2288 | 0.3586 | 0.6478 | -0.6263 |  |
| Olfr1160 | na | 0.07863 | 0.1266 | 0.6488 | -0.6241 | 0.002679 | 0.009609 | 0.3882 | -1.365 |  |
| Apcs | na | 0.05795 | 0.09879 | 0.6636 | -0.5916 | 0.003905 | 0.01321 | 0.4553 | -1.135 |  |
| Comt | na | 0.08576 | 0.136 | 0.6977 | -0.5193 | 0.00261 | 0.009418 | 0.4447 | -1.169 |  |
| Zfp9 | na | 0.4272 | 0.5165 | 1.143 | 0.1932 | 0.0007227 | 0.003249 | 2.181 | 1.125 |  |
| Gm15432 | na | 0.05223 | 0.09053 | 0.6381 | -0.6481 | 0.004453 | 0.01463 | 0.438 | -1.191 |  |
| Sucnr1 | na | 0.07443 | 0.1211 | 0.6897 | -0.5359 | 0.00341 | 0.01182 | 0.4604 | -1.119 |  |
| Sbk1 | na | 0.1061 | 0.1623 | 1.491 | 0.5767 | 0.002338 | 0.00862 | 2.648 | 1.405 |  |
| Cyp7a1 | na | 0.4701 | 0.5564 | 1.145 | 0.1957 | 0.0007138 | 0.003228 | 2.388 | 1.256 |  |
| Myc | na | 0.001701 | 0.005854 | 2.253 | 1.172 | 0.8809 | 0.9188 | 0.9605 | -0.05809 |  |
| Mtmr11 | na | 0.2554 | 0.3375 | 0.8022 | -0.318 | 0.001327 | 0.005517 | 0.4359 | -1.198 |  |
| Gvin1 | na | 0.05682 | 0.09714 | 0.6394 | -0.6451 | 0.005244 | 0.01685 | 0.4404 | -1.183 |  |
| Psmb10 | na | 0.3328 | 0.4183 | 0.8497 | -0.2349 | 0.001159 | 0.004921 | 0.4793 | -1.061 |  |
| Slc16a7 | na | 0.4667 | 0.5535 | 1.155 | 0.2084 | 0.0008638 | 0.003788 | 2.462 | 1.3 |  |
| Onecut1 | na | 0.1836 | 0.257 | 0.5366 | -0.898 | 0.001897 | 0.007358 | 0.1478 | -2.758 |  |
| Ttpa | na | 0.08692 | 0.1368 | 1.448 | 0.534 | 0.003508 | 0.01204 | 2.236 | 1.161 |  |
| Ppargc1a | na | 0.4237 | 0.5133 | 1.185 | 0.2454 | 0.001161 | 0.004921 | 2.542 | 1.346 |  |
| Cdsn | na | 0.001939 | 0.006405 | 0.4371 | -1.194 | 0.8159 | 0.8803 | 1.067 | 0.09345 |  |
| Gm8989 | na | 0.1641 | 0.2337 | 0.7548 | -0.4059 | 0.00215 | 0.008128 | 0.4441 | -1.171 |  |
| Igfbp3 | na | 0.002095 | 0.006787 | 2.108 | 1.076 | 0.7207 | 0.8006 | 0.9135 | -0.1306 |  |
| Gm9847 | na | 0.1171 | 0.1771 | 0.6532 | -0.6144 | 0.003423 | 0.01183 | 0.36 | -1.474 |  |
| Haao | na | 0.003505 | 0.009863 | 2.261 | 1.177 | 0.2418 | 0.3698 | 1.422 | 0.5075 |  |
| BC021614 | na | 0.8156 | 0.8609 | 0.9652 | -0.05117 | 0.000714 | 0.003228 | 0.4921 | -1.023 |  |
| Lin7a | na | 0.413 | 0.5013 | 0.8613 | -0.2154 | 0.001448 | 0.005898 | 0.4585 | -1.125 |  |
| Slc10a2 | na | 0.1346 | 0.1986 | 0.7404 | -0.4336 | 0.003439 | 0.01185 | 0.4691 | -1.092 |  |
| Cyp2b10 | na | 0.007452 | 0.01834 | 3.872 | 1.953 | 0.1266 | 0.2264 | 2.357 | 1.237 |  |
| Sulf2 | na | 0.8876 | 0.9185 | 1.027 | 0.03849 | 0.000865 | 0.003788 | 2.369 | 1.244 |  |
| Tmem254c | na | 0.7613 | 0.817 | 1.054 | 0.07541 | 0.001124 | 0.00479 | 2.145 | 1.101 |  |
| Selenbp1 | na | 0.2079 | 0.2838 | 0.7775 | -0.363 | 0.002692 | 0.009641 | 0.4569 | -1.13 |  |
| Fam129b | na | 0.148 | 0.2145 | 1.34 | 0.4222 | 0.004241 | 0.01407 | 0.476 | -1.071 |  |
| Otub2 | na | 0.009474 | 0.02248 | 0.4857 | -1.042 | 0.123 | 0.2218 | 0.619 | -0.692 |  |
| Alpl | na | 0.4667 | 0.5535 | 0.8489 | -0.2364 | 0.001992 | 0.007653 | 2.532 | 1.34 |  |
| Postn | na | 0.0102 | 0.02392 | 2.022 | 1.016 | 0.1317 | 0.2334 | 1.587 | 0.6664 |  |
| Pnpla3 | na | 0.003644 | 0.01014 | 0.3983 | -1.328 | 0.3834 | 0.5183 | 1.343 | 0.4254 |  |
| Rasd1 | na | 0.006272 | 0.01601 | 0.4447 | -1.169 | 0.2833 | 0.4159 | 1.413 | 0.4992 |  |
| Rpsa | na | 0.1025 | 0.1576 | 0.7103 | -0.4935 | 0.00764 | 0.02306 | 0.4959 | -1.012 |  |
| Mir365-2 | na | 0.006854 | 0.01728 | 0.4993 | -1.002 | 0.3035 | 0.4393 | 1.334 | 0.4154 |  |
| Apoa4 | na | 0.6171 | 0.6955 | 1.204 | 0.2676 | 0.00214 | 0.008116 | 4.582 | 2.196 |  |
| mt-Tg | na | 0.1498 | 0.2167 | 0.612 | -0.7083 | 0.005733 | 0.01808 | 0.3012 | -1.731 |  |
| Adrb2 | na | 0.003297 | 0.009521 | 2.253 | 1.172 | 0.7206 | 0.8006 | 1.11 | 0.1508 |  |
| Wfdc1 | na | 0.1251 | 0.187 | 0.7283 | -0.4573 | 0.007365 | 0.0224 | 0.4972 | -1.008 |  |
| Plk3 | na | 0.1914 | 0.2657 | 0.7567 | -0.4022 | 0.004738 | 0.01548 | 0.4607 | -1.118 |  |
| Dhrs9 | na | 0.8306 | 0.8729 | 0.9589 | -0.06058 | 0.001916 | 0.007397 | 0.441 | -1.181 |  |
| Mpeg1 | na | 0.003299 | 0.009521 | 3.072 | 1.619 | 0.8427 | 0.8978 | 1.083 | 0.1154 |  |
| Cyp2b9 | na | 0.009126 | 0.02185 | 2.089 | 1.063 | 0.2714 | 0.4014 | 1.407 | 0.4928 |  |
| Btg1 | na | 0.003606 | 0.01007 | 2.395 | 1.26 | 0.7156 | 0.7967 | 1.123 | 0.1671 |  |
| Npas2 | na | 0.43 | 0.5191 | 1.191 | 0.252 | 0.003879 | 0.01314 | 0.4326 | -1.209 |  |
| Tubb2a | na | 0.7309 | 0.7911 | 0.9351 | -0.09685 | 0.00261 | 0.009418 | 0.4572 | -1.129 |  |
| Cabyr | na | 0.4374 | 0.5248 | 0.7676 | -0.3816 | 0.004103 | 0.01376 | 0.2782 | -1.846 |  |
| Itgb2 | na | 0.01061 | 0.02478 | 2.038 | 1.027 | 0.2915 | 0.4261 | 1.382 | 0.4669 |  |
| Prg4 | na | 0.01919 | 0.03979 | 2.289 | 1.195 | 0.1463 | 0.2541 | 0.5591 | -0.8387 |  |
| Angpt1 | na | 0.004967 | 0.01317 | 0.4955 | -1.013 | 0.688 | 0.7772 | 0.8983 | -0.1547 |  |
| Stk17b | na | 0.007428 | 0.01831 | 2.667 | 1.415 | 0.4469 | 0.5763 | 1.353 | 0.4362 |  |
| Rsad2 | na | 0.1668 | 0.2372 | 1.361 | 0.4451 | 0.01087 | 0.03065 | 2.043 | 1.031 |  |
| Cyp4a10 | na | 0.2736 | 0.3567 | 1.333 | 0.4148 | 0.007593 | 0.02294 | 2.452 | 1.294 |  |
| Slc22a3 | na | 0.6207 | 0.6989 | 1.106 | 0.1447 | 0.004799 | 0.01565 | 2.116 | 1.081 |  |
| Irf5 | na | 0.9373 | 0.9554 | 0.9828 | -0.02504 | 0.004179 | 0.01394 | 0.4344 | -1.203 |  |
| Lmod2 | na | 0.01741 | 0.03682 | 2.088 | 1.062 | 0.289 | 0.4232 | 1.442 | 0.5278 |  |
| Gm4297 | na | 0.5111 | 0.597 | 0.8502 | -0.2342 | 0.00656 | 0.02023 | 0.4184 | -1.257 |  |
| Gbp10 | na | 0.6691 | 0.7401 | 0.9164 | -0.126 | 0.005635 | 0.01782 | 0.4776 | -1.066 |  |
| Parm1 | na | 0.006465 | 0.01639 | 0.4544 | -1.138 | 0.8805 | 0.9188 | 0.9543 | -0.06742 |  |
| Ugt1a8 | na | 0.6109 | 0.6891 | 1.236 | 0.3052 | 0.006515 | 0.02015 | 4.353 | 2.122 |  |
| Cd68 | na | 0.007282 | 0.01808 | 2.006 | 1.004 | 0.8453 | 0.8998 | 1.056 | 0.07859 |  |
| Glipr1 | na | 0.01587 | 0.03418 | 2.095 | 1.067 | 0.3745 | 0.5113 | 1.352 | 0.4353 |  |
| Cntfr | na | 0.7748 | 0.8265 | 0.9394 | -0.09021 | 0.006858 | 0.0211 | 2.157 | 1.109 |  |
| n-R5s88 | na | 0.5485 | 0.6318 | 0.8599 | -0.2177 | 0.008449 | 0.02515 | 0.4254 | -1.233 |  |
| Acta1 | na | 0.01675 | 0.03576 | 2.035 | 1.025 | 0.4424 | 0.5727 | 1.287 | 0.3641 |  |
| n-R5s136 | na | 0.2342 | 0.3136 | 0.6784 | -0.5598 | 0.01847 | 0.04748 | 0.3829 | -1.385 |  |
| Tmem100 | na | 0.94 | 0.9573 | 1.016 | 0.02303 | 0.006703 | 0.02065 | 2.116 | 1.081 |  |
| Slfn2 | na | 0.02045 | 0.04189 | 2.28 | 1.189 | 0.3787 | 0.5143 | 1.417 | 0.503 |  |
| Lbp | na | 0.7518 | 0.8079 | 0.93 | -0.1047 | 0.009838 | 0.0286 | 2.148 | 1.103 |  |
| Grb14 | na | 0.0199 | 0.04097 | 0.4753 | -1.073 | 0.6816 | 0.7729 | 1.156 | 0.2088 |  |
