# Supplementary material for: GIANT: galaxy-based tool for interactive analysis of transcriptomic data
Source: Sci Rep. 2020 Nov 16;10:19835. doi: 10.1038/s41598-020-76769-w (PMC7670435; doi:10.1038/s41598-020-76769-w)
Supplement: Supplementary file 2 — Supplementary Information. [file 41598_2020_76769_MOESM2_ESM.zip › Tools_InputOutput_Parameters/MicroArray_workflow/Step5-Volcano/Outputs/Volcano_FastedVsFed_HTML.html_78.html]

### Statistics (p.val, adjusted p.val, FC, log2FC)

Access to statistics

### Volcanos

Volcano FastedVsFed

Volcano FastedVsFed-Liver
