# Supplementary material for: GIANT: galaxy-based tool for interactive analysis of transcriptomic data
Source: Sci Rep. 2020 Nov 16;10:19835. doi: 10.1038/s41598-020-76769-w (PMC7670435; doi:10.1038/s41598-020-76769-w)
Supplement: Supplementary file 2 — Supplementary Information. [file 41598_2020_76769_MOESM2_ESM.zip › Tools_InputOutput_Parameters/MicroArray_workflow/Step6-Heatmap/Outputs/extra_files_path_81/outputClustering.html]

| Gene | Info | Cluster | FastedVsFed-Liver | | | | |  |
| --- | --- | --- | --- | --- | --- | --- | --- | --- |
|  |  |  | p-val | FDR.p-val | FC | log2(FC) | t-stat |  |
| Gene | Info | Cluster | p-val | FDR.p-val | FC | log2(FC) | t-stat |  |
| --- | --- | --- | --- | --- | --- | --- | --- | --- |
| Serpina7 | serine (or cysteine) peptidase | 1 | 1.092e-14 | 1.404e-10 | 0.03679 | -4.765 | -20.02 |  |
| --- | --- | --- | --- | --- | --- | --- | --- | --- |
| Gm15998 | predicted gene 15998 | 1 | 1.943e-14 | 1.666e-10 | 0.03532 | -4.823 | -19.42 |  |
| Pcsk9 | proprotein convertase subtilis | 1 | 6.793e-13 | 3.472e-09 | 0.09384 | -3.414 | -16.08 |  |
| Hsd3b5 | hydroxy-delta-5-steroid dehydr | 2 | 8.1e-13 | 3.472e-09 | 0.01062 | -6.557 | -15.93 |  |
| Zbtb16 | zinc finger and BTB domain con | 3 | 7.096e-13 | 3.472e-09 | 7.299 | 2.868 | 16.04 |  |
| Usp2 | ubiquitin specific peptidase 2 | 4 | 1.855e-11 | 6.816e-08 | 6.015 | 2.589 | 13.43 |  |
| Cyp17a1 | cytochrome P450, family 17, su | 1 | 9.986e-11 | 2.335e-07 | 22.49 | 4.491 | 12.22 |  |
| Rgn | regucalcin | 2 | 1.336e-10 | 2.741e-07 | 0.3077 | -1.7 | -12.02 |  |
| Slc16a5 | solute carrier family 16 (mono | 1 | 1.851e-10 | 3.174e-07 | 8.613 | 3.106 | 11.8 |  |
| Pfkfb2 | 6-phosphofructo-2-kinase/fruct | 5 | 1.789e-10 | 3.174e-07 | 3.519 | 1.815 | 11.82 |  |
| Mfsd2a | major facilitator superfamily | 2 | 3.438e-10 | 5.527e-07 | 16.69 | 4.061 | 11.39 |  |
| Hsd3b4 | hydroxy-delta-5-steroid dehydr | 2 | 4.839e-10 | 6.224e-07 | 0.09551 | -3.388 | -11.17 |  |
| Apex2 | apurinic/apyrimidinic endonucl | 4 | 3.876e-10 | 5.538e-07 | 4.869 | 2.284 | 11.31 |  |
| Clec2h | C-type lectin domain family 2, | 1 | 1.869e-09 | 1.849e-06 | 0.06554 | -3.932 | -10.32 |  |
| Cyp4a31 | cytochrome P450, family 4, sub | 2 | 4.203e-10 | 5.69e-07 | 7.932 | 2.988 | 11.26 |  |
| Adgrf1 | adhesion G protein-coupled rec | 1 | 2.503e-09 | 2.038e-06 | 0.1301 | -2.942 | -10.15 |  |
| Rgs16 | regulator of G-protein signali | 1 | 2.907e-09 | 2.199e-06 | 30.67 | 4.939 | 10.06 |  |
| Nudt7 | nudix (nucleoside diphosphate | 5 | 2.204e-09 | 1.949e-06 | 0.1491 | -2.746 | -10.22 |  |
| Cdkn1a | cyclin-dependent kinase inhibi | 4 | 9.415e-15 | 1.404e-10 | 39.55 | 5.306 | 20.17 |  |
| Mvd | mevalonate (diphospho) decarbo | 1 | 2.273e-09 | 1.949e-06 | 0.1409 | -2.828 | -10.21 |  |
| Hmgcr | 3-hydroxy-3-methylglutaryl-Coe | 5 | 1.23e-09 | 1.439e-06 | 0.3155 | -1.664 | -10.58 |  |
| Gstm3 | glutathione S-transferase, mu | 2 | 6.581e-09 | 4.232e-06 | 0.1117 | -3.162 | -9.577 |  |
| Adh4 | alcohol dehydrogenase 4 (class | 2 | 8.693e-09 | 5.324e-06 | 0.3351 | -1.577 | -9.417 |  |
| Keg1 | kidney expressed gene 1 | 2 | 9.273e-09 | 5.327e-06 | 0.146 | -2.776 | -9.38 |  |
| Lss | lanosterol synthase | 5 | 2.245e-11 | 7.219e-08 | 0.1624 | -2.622 | -13.29 |  |
| C330021F23Rik | RIKEN cDNA C330021F23 gene | 1 | 9.319e-09 | 5.327e-06 | 0.1472 | -2.764 | -9.378 |  |
| Car3 | carbonic anhydrase 3 | 3 | 9.584e-09 | 5.359e-06 | 0.3089 | -1.695 | -9.362 |  |
| Gm15441 | predicted gene 15441 | 1 | 1.777e-09 | 1.828e-06 | 9.491 | 3.247 | 10.35 |  |
| Aqp8 | aquaporin 8 | 2 | 1.074e-08 | 5.61e-06 | 0.13 | -2.944 | -9.297 |  |
| Id3 | inhibitor of DNA binding 3 | 5 | 1.09e-08 | 5.61e-06 | 0.146 | -2.776 | -9.288 |  |
| St3gal5 | ST3 beta-galactoside alpha-2,3 | 4 | 1.637e-08 | 7.655e-06 | 4.255 | 2.089 | 9.06 |  |
| Chrna4 | cholinergic receptor, nicotini | 1 | 2.462e-08 | 1.005e-05 | 0.1253 | -2.997 | -8.834 |  |
| 5830473C10Rik | NA | 2 | 2.905e-08 | 1.132e-05 | 0.3087 | -1.696 | -8.743 |  |
| Rdh11 | retinol dehydrogenase 11 | 2 | 6.818e-09 | 4.277e-06 | 0.1941 | -2.365 | -9.557 |  |
| Cyp2c55 | cytochrome P450, family 2, sub | 1 | 3.078e-08 | 1.171e-05 | 0.1022 | -3.291 | -8.712 |  |
| Map2k6 | mitogen-activated protein kina | 4 | 9.592e-05 | 0.005765 | 2.229 | 1.156 | 4.857 |  |
| Asl | argininosuccinate lyase | 2 | 2.535e-09 | 2.038e-06 | 2.391 | 1.258 | 10.14 |  |
| Dpy19l3 | dpy-19-like 3 (C. elegans) | 5 | 1.84e-08 | 8.061e-06 | 0.1608 | -2.637 | -8.995 |  |
| Pmvk | phosphomevalonate kinase | 5 | 2.015e-08 | 8.497e-06 | 0.2007 | -2.317 | -8.944 |  |
| Aacs | acetoacetyl-CoA synthetase | 5 | 2.175e-08 | 9.026e-06 | 0.09067 | -3.463 | -8.902 |  |
| Prok1 | prokineticin 1 | 1 | 3.43e-08 | 1.279e-05 | 0.3663 | -1.449 | -8.653 |  |
| Asic5 | acid-sensing (proton-gated) io | 1 | 1.983e-08 | 8.497e-06 | 3.56 | 1.832 | 8.953 |  |
| Sdsl | serine dehydratase-like | 1 | 1.502e-09 | 1.68e-06 | 3.269 | 1.709 | 10.46 |  |
| Gabbr2 | gamma-aminobutyric acid (GABA) | 2 | 5.475e-08 | 1.984e-05 | 0.3638 | -1.459 | -8.402 |  |
| Tnfaip2 | tumor necrosis factor, alpha-i | 5 | 6.942e-06 | 0.0008544 | 0.2717 | -1.88 | -6.022 |  |
| As3mt | arsenic (+3 oxidation state) m | 4 | 6.795e-08 | 2.3e-05 | 3.187 | 1.672 | 8.287 |  |
| Slc22a5 | solute carrier family 22 (orga | 5 | 8.93e-09 | 5.327e-06 | 3.471 | 1.795 | 9.402 |  |
| Acacb | acetyl-Coenzyme A carboxylase | 3 | 9.303e-06 | 0.00104 | 0.3411 | -1.552 | -5.889 |  |
| Defb1 | defensin beta 1 | 1 | 1.072e-07 | 3.362e-05 | 4.135 | 2.048 | 8.048 |  |
| Cyp2c70 | cytochrome P450, family 2, sub | 2 | 5.821e-08 | 1.996e-05 | 0.1575 | -2.666 | -8.369 |  |
| 2810416G20Rik | NA | 4 | 8.798e-08 | 2.865e-05 | 4.507 | 2.172 | 8.151 |  |
| Abca8a | ATP-binding cassette, sub-fami | 3 | 8.539e-08 | 2.816e-05 | 0.1885 | -2.408 | -8.167 |  |
| Cyp39a1 | cytochrome P450, family 39, su | 4 | 5.722e-08 | 1.989e-05 | 5.338 | 2.416 | 8.378 |  |
| Sqle | squalene epoxidase | 2 | 3.788e-08 | 1.392e-05 | 0.1214 | -3.042 | -8.599 |  |
| Hes6 | hairy and enhancer of split 6 | 2 | 1.081e-08 | 5.61e-06 | 0.2319 | -2.109 | -9.293 |  |
| Mvk | mevalonate kinase | 5 | 1.385e-10 | 2.741e-07 | 0.2161 | -2.21 | -12 |  |
| BC029214 | NA | 5 | 1.376e-07 | 4.165e-05 | 0.2723 | -1.877 | -7.919 |  |
| Hsd3b2 | hydroxy-delta-5-steroid dehydr | 2 | 1.875e-07 | 5.42e-05 | 0.2995 | -1.74 | -7.76 |  |
| Efna1 | ephrin A1 | 5 | 2.515e-08 | 1.006e-05 | 0.311 | -1.685 | -8.822 |  |
| Ddit4 | DNA-damage-inducible transcrip | 4 | 6.18e-07 | 0.0001359 | 10.92 | 3.449 | 7.164 |  |
| Fabp5 | fatty acid binding protein 5, | 5 | 1.401e-07 | 4.191e-05 | 0.128 | -2.966 | -7.909 |  |
| Afm | afamin | 2 | 2.212e-07 | 6.184e-05 | 0.4836 | -1.048 | -7.676 |  |
| Gm38481 | NA | 5 | 6.249e-09 | 4.122e-06 | 0.1427 | -2.809 | -9.607 |  |
| Selenbp2 | selenium binding protein 2 | 5 | 2.625e-07 | 7.033e-05 | 0.2641 | -1.921 | -7.59 |  |
| Acadm | acyl-Coenzyme A dehydrogenase, | 3 | 1.633e-07 | 4.828e-05 | 2.339 | 1.226 | 7.831 |  |
| Nsdhl | NAD(P) dependent steroid dehyd | 5 | 1.17e-08 | 5.679e-06 | 0.2069 | -2.273 | -9.248 |  |
| Cebpd | CCAAT/enhancer binding protein | 4 | 5.737e-06 | 0.0007521 | 2.714 | 1.44 | 6.109 |  |
| Slc7a8 | solute carrier family 7 (catio | 3 | 8.905e-05 | 0.005449 | 0.452 | -1.146 | -4.889 |  |
| Serpina4-ps1 | serine (or cysteine) peptidase | 2 | 3.244e-07 | 8.262e-05 | 0.05254 | -4.25 | -7.483 |  |
| Hsdl2 | hydroxysteroid dehydrogenase l | 3 | 1.071e-07 | 3.362e-05 | 2.255 | 1.173 | 8.048 |  |
| Acot2 | acyl-CoA thioesterase 2 | 4 | 2.659e-07 | 7.051e-05 | 5.729 | 2.518 | 7.583 |  |
| Crat | carnitine acetyltransferase | 3 | 3.215e-07 | 8.262e-05 | 2.528 | 1.338 | 7.488 |  |
| AA986860 | expressed sequence AA986860 | 1 | 5.631e-08 | 1.984e-05 | 2.153 | 1.106 | 8.387 |  |
| 1810064F22Rik | RIKEN cDNA 1810064F22 gene | 1 | 3.359e-07 | 8.472e-05 | 0.1948 | -2.36 | -7.466 |  |
| Rab30 | RAB30, member RAS oncogene fam | 5 | 1.849e-08 | 8.061e-06 | 4.865 | 2.282 | 8.992 |  |
| 1810055G02Rik | RIKEN cDNA 1810055G02 gene | 5 | 1.571e-08 | 7.482e-06 | 3.723 | 1.896 | 9.083 |  |
| Fdft1 | farnesyl diphosphate farnesyl | 3 | 2.132e-07 | 6.095e-05 | 0.3195 | -1.646 | -7.695 |  |
| Gcat | glycine C-acetyltransferase (2 | 1 | 2.542e-08 | 1.006e-05 | 0.4253 | -1.234 | -8.816 |  |
| Cyp1a2 | cytochrome P450, family 1, sub | 2 | 4.874e-07 | 0.0001117 | 0.4558 | -1.134 | -7.281 |  |
| Slc22a28 | solute carrier family 22, memb | 2 | 5.293e-07 | 0.0001194 | 0.2926 | -1.773 | -7.24 |  |
| Slc19a1 | solute carrier family 19 (fola | 5 | 2.479e-07 | 6.783e-05 | 2.274 | 1.185 | 7.618 |  |
| Rnpep | arginyl aminopeptidase (aminop | 5 | 1.767e-07 | 5.165e-05 | 0.479 | -1.062 | -7.79 |  |
| Cyp51 | cytochrome P450, family 51 | 5 | 6.475e-07 | 0.00014 | 0.1685 | -2.569 | -7.141 |  |
| Pold2 | polymerase (DNA directed), del | 4 | 3.408e-07 | 8.511e-05 | 0.3917 | -1.352 | -7.459 |  |
| Sort1 | sortilin 1 | 3 | 4.198e-07 | 9.818e-05 | 0.3598 | -1.475 | -7.355 |  |
| Sc5d | sterol-C5-desaturase | 3 | 1.136e-08 | 5.624e-06 | 0.3038 | -1.719 | -9.265 |  |
| Cars | cysteinyl-tRNA synthetase | 4 | 1.171e-06 | 0.0002299 | 0.4285 | -1.222 | -6.854 |  |
| Hsd3b3 | hydroxy-delta-5-steroid dehydr | 2 | 1.194e-06 | 0.0002326 | 0.3753 | -1.414 | -6.845 |  |
| Cyp4f14 | cytochrome P450, family 4, sub | 2 | 5.894e-07 | 0.0001307 | 0.3505 | -1.512 | -7.187 |  |
| 1700039E15Rik | NA | 1 | 5.684e-07 | 0.0001271 | 2.086 | 1.061 | 7.205 |  |
| 1600002H07Rik | NA | 2 | 8.526e-08 | 2.816e-05 | 3.296 | 1.721 | 8.167 |  |
| Lpin1 | lipin 1 | 3 | 5.376e-09 | 3.738e-06 | 4.818 | 2.269 | 9.695 |  |
| Fdps | farnesyl diphosphate synthetas | 5 | 1.084e-08 | 5.61e-06 | 0.163 | -2.617 | -9.291 |  |
| Insig1 | insulin induced gene 1 | 3 | 2.101e-09 | 1.949e-06 | 0.1823 | -2.456 | -10.25 |  |
| Mlxipl | MLX interacting protein-like | 3 | 1.104e-07 | 3.421e-05 | 0.4309 | -1.214 | -8.033 |  |
| Cdip1 | cell death inducing Trp53 targ | 4 | 4.14e-07 | 9.77e-05 | 2.771 | 1.471 | 7.362 |  |
| Srebf1 | sterol regulatory element bind | 5 | 8.962e-11 | 2.305e-07 | 0.08513 | -3.554 | -12.3 |  |
| Slc27a1 | solute carrier family 27 (fatt | 3 | 1.58e-09 | 1.693e-06 | 5.338 | 2.416 | 10.43 |  |
| Osgin1 | oxidative stress induced growt | 5 | 1.83e-08 | 8.061e-06 | 0.1543 | -2.696 | -8.998 |  |
| Prss8 | protease, serine 8 (prostasin) | 1 | 1.126e-06 | 0.0002263 | 0.2888 | -1.792 | -6.873 |  |
| Gm3571 | predicted gene 3571 | 2 | 4.907e-07 | 0.0001117 | 0.2291 | -2.126 | -7.278 |  |
| Fam149a | family with sequence similarit | 2 | 3.579e-07 | 8.709e-05 | 0.4461 | -1.165 | -7.434 |  |
| Il6ra | interleukin 6 receptor, alpha | 4 | 4.763e-07 | 0.0001104 | 3.768 | 1.914 | 7.292 |  |
| Cpt1b | carnitine palmitoyltransferase | 4 | 2.185e-06 | 0.0003698 | 2.365 | 1.242 | 6.558 |  |
| Gjc3 | gap junction protein, gamma 3 | 1 | 2.434e-06 | 0.0004046 | 4.11 | 2.039 | 6.507 |  |
| Gamt | guanidinoacetate methyltransfe | 5 | 6.598e-07 | 0.0001414 | 0.3519 | -1.507 | -7.132 |  |
| Akr1c6 | aldo-keto reductase family 1, | 2 | 2.119e-06 | 0.0003633 | 0.4491 | -1.155 | -6.572 |  |
| Plin4 | perilipin 4 | 3 | 6.429e-07 | 0.00014 | 4.091 | 2.032 | 7.145 |  |
| Klk1b4 | kallikrein 1-related pepidase | 1 | 1.74e-06 | 0.0003176 | 0.316 | -1.662 | -6.665 |  |
| Foxo1 | forkhead box O1 | 3 | 1.531e-05 | 0.001485 | 2.355 | 1.236 | 5.664 |  |
| Mbl1 | mannose-binding lectin (protei | 2 | 1.741e-06 | 0.0003176 | 0.4374 | -1.193 | -6.665 |  |
| Gm16551 | predicted gene 16551 | 1 | 2.753e-06 | 0.0004383 | 0.1911 | -2.388 | -6.449 |  |
| Crcp | calcitonin gene-related peptid | 5 | 2.904e-06 | 0.0004547 | 2.603 | 1.38 | 6.424 |  |
| Acot1 | acyl-CoA thioesterase 1 | 2 | 3.864e-10 | 5.538e-07 | 5.253 | 2.393 | 11.32 |  |
| Mir704 | microRNA 704 | 1 | 3.835e-07 | 9.22e-05 | 4.805 | 2.264 | 7.4 |  |
| Catsper2 | cation channel, sperm associat | 1 | 2.21e-07 | 6.184e-05 | 2.792 | 1.481 | 7.676 |  |
| Gadd45b | growth arrest and DNA-damage-i | 4 | 9.237e-08 | 2.97e-05 | 6.782 | 2.762 | 8.126 |  |
| Eps8l2 | EPS8-like 2 | 2 | 2.499e-06 | 0.0004094 | 0.4912 | -1.026 | -6.494 |  |
| Pgd | phosphogluconate dehydrogenase | 3 | 9.073e-06 | 0.001019 | 0.4462 | -1.164 | -5.9 |  |
| Slc41a3 | solute carrier family 41, memb | 4 | 5.854e-09 | 3.962e-06 | 5.058 | 2.338 | 9.645 |  |
| Cntnap1 | contactin associated protein-l | 1 | 3.427e-06 | 0.0005214 | 2.636 | 1.398 | 6.347 |  |
| Cyp21a2-ps | cytochrome P450, family 21, su | 1 | 2.453e-06 | 0.0004046 | 0.3503 | -1.513 | -6.503 |  |
| Map3k13 | mitogen-activated protein kina | 2 | 2.181e-06 | 0.0003698 | 0.4201 | -1.251 | -6.559 |  |
| Igfals | insulin-like growth factor bin | 2 | 3.575e-05 | 0.002821 | 0.3573 | -1.485 | -5.288 |  |
| Slc37a4 | solute carrier family 37 (gluc | 4 | 1.182e-07 | 3.619e-05 | 2.802 | 1.487 | 7.997 |  |
| 1700001C19Rik | RIKEN cDNA 1700001C19 gene | 1 | 3.683e-06 | 0.0005466 | 0.4911 | -1.026 | -6.314 |  |
| Abcb4 | ATP-binding cassette, sub-fami | 4 | 1.938e-06 | 0.0003453 | 2.063 | 1.045 | 6.614 |  |
| Pptc7 | PTC7 protein phosphatase homol | 4 | 6.875e-07 | 0.0001462 | 2.909 | 1.54 | 7.112 |  |
| Slc10a6 | solute carrier family 10 (sodi | 1 | 8.55e-05 | 0.005304 | 2.318 | 1.213 | 4.907 |  |
| Gm15453 | NA | 3 | 2.346e-06 | 0.0003944 | 2.952 | 1.562 | 6.524 |  |
| Gm19680 | predicted gene, 19680 | 5 | 2.772e-06 | 0.0004383 | 0.3482 | -1.522 | -6.446 |  |
| Fbxo31 | F-box protein 31 | 3 | 3.589e-07 | 8.709e-05 | 3.828 | 1.937 | 7.433 |  |
| Atp4a | ATPase, H+/K+ exchanging, gast | 1 | 3.745e-06 | 0.0005505 | 0.4612 | -1.116 | -6.306 |  |
| Thrsp | thyroid hormone responsive | 3 | 2.047e-06 | 0.0003534 | 0.1128 | -3.148 | -6.588 |  |
| Fgfr3 | fibroblast growth factor recep | 2 | 0.0001052 | 0.006142 | 0.4947 | -1.015 | -4.817 |  |
| Slc22a7 | solute carrier family 22 (orga | 1 | 6.596e-06 | 0.0008253 | 0.2564 | -1.963 | -6.045 |  |
| Lifr | LIF receptor alpha | 3 | 2.777e-06 | 0.0004383 | 0.4326 | -1.209 | -6.445 |  |
| Gm10156 | predicted gene 10156 | 3 | 3.459e-06 | 0.0005214 | 2.564 | 1.358 | 6.343 |  |
| Adra1b | adrenergic receptor, alpha 1b | 2 | 5.762e-06 | 0.0007521 | 0.3999 | -1.322 | -6.107 |  |
| Slc17a8 | solute carrier family 17 (sodi | 1 | 5.448e-06 | 0.0007262 | 2.756 | 1.463 | 6.133 |  |
| Hsd17b10 | hydroxysteroid (17-beta) dehyd | 3 | 2.519e-06 | 0.0004101 | 2.031 | 1.022 | 6.491 |  |
| Acnat2 | acyl-coenzyme A amino acid N-a | 1 | 6.044e-06 | 0.0007773 | 3.251 | 1.701 | 6.085 |  |
| Msmo1 | methylsterol monoxygenase 1 | 5 | 1.228e-06 | 0.0002375 | 0.2169 | -2.205 | -6.832 |  |
| Fbxo36 | F-box protein 36 | 5 | 6.786e-06 | 0.0008432 | 2.61 | 1.384 | 6.032 |  |
| Spryd3 | SPRY domain containing 3 | 5 | 1.43e-06 | 0.0002685 | 2.632 | 1.396 | 6.759 |  |
| Ciart | circadian associated repressor | 1 | 8.053e-06 | 0.0009547 | 3.537 | 1.822 | 5.954 |  |
| Retsat | retinol saturase (all trans re | 3 | 5.595e-08 | 1.984e-05 | 2.485 | 1.313 | 8.39 |  |
| Tor1a | torsin family 1, member A (tor | 5 | 7.91e-07 | 0.0001654 | 2.006 | 1.004 | 7.044 |  |
| Rnf19b | ring finger protein 19B | 4 | 7.481e-06 | 0.0009034 | 0.4972 | -1.008 | -5.988 |  |
| Ip6k2 | inositol hexaphosphate kinase | 5 | 1.781e-06 | 0.0003226 | 2.175 | 1.121 | 6.654 |  |
| Abcb10 | ATP-binding cassette, sub-fami | 5 | 2.038e-06 | 0.0003534 | 0.4706 | -1.087 | -6.591 |  |
| Mylip | myosin regulatory light chain | 4 | 1.404e-06 | 0.0002656 | 2.58 | 1.368 | 6.767 |  |
| Slc20a1 | solute carrier family 20, memb | 4 | 2.444e-07 | 6.759e-05 | 2.693 | 1.429 | 7.626 |  |
| Tram2 | translocating chain-associatin | 5 | 8.252e-06 | 0.0009713 | 2.413 | 1.271 | 5.943 |  |
| Agpat9 | NA | 5 | 6.127e-06 | 0.0007841 | 3.431 | 1.779 | 6.079 |  |
| Idi1 | isopentenyl-diphosphate delta | 5 | 3.096e-08 | 1.171e-05 | 0.1392 | -2.845 | -8.709 |  |
| Angptl2 | angiopoietin-like 2 | 3 | 0.0004451 | 0.01704 | 0.4858 | -1.042 | -4.197 |  |
| Rnf186 | ring finger protein 186 | 1 | 4.044e-06 | 0.0005844 | 0.3776 | -1.405 | -6.27 |  |
| Noct | nocturnin | 5 | 1.135e-06 | 0.0002264 | 3.991 | 1.997 | 6.869 |  |
| Bhmt | betaine-homocysteine methyltra | 1 | 9.898e-06 | 0.001093 | 2.534 | 1.341 | 5.86 |  |
| Abca2 | ATP-binding cassette, sub-fami | 5 | 1.946e-06 | 0.0003453 | 0.3638 | -1.459 | -6.612 |  |
| Tox | thymocyte selection-associated | 1 | 4.268e-06 | 0.0006099 | 3.903 | 1.964 | 6.245 |  |
| Hmgcs1 | 3-hydroxy-3-methylglutaryl-Coe | 3 | 6.283e-06 | 0.0007962 | 0.1672 | -2.58 | -6.067 |  |
| Serpina6 | serine (or cysteine) peptidase | 2 | 8.382e-06 | 0.000973 | 0.4516 | -1.147 | -5.936 |  |
| Nr4a1 | nuclear receptor subfamily 4, | 4 | 1.147e-06 | 0.0002269 | 0.2007 | -2.317 | -6.864 |  |
| Phlda2 | pleckstrin homology like domai | 1 | 3.697e-06 | 0.0005466 | 0.4468 | -1.162 | -6.312 |  |
| Cd276 | CD276 antigen | 5 | 3.443e-07 | 8.515e-05 | 0.4372 | -1.194 | -7.453 |  |
| Nr1i3 | nuclear receptor subfamily 1, | 2 | 1.047e-05 | 0.001122 | 2.244 | 1.166 | 5.835 |  |
| Tlcd2 | TLC domain containing 2 | 2 | 4.408e-06 | 0.0006264 | 0.4325 | -1.209 | -6.23 |  |
| Eif4ebp3 | eukaryotic translation initiat | 4 | 2.776e-09 | 2.164e-06 | 4.661 | 2.22 | 10.09 |  |
| Tmc7 | transmembrane channel-like gen | 4 | 0.0002836 | 0.01234 | 2.028 | 1.02 | 4.39 |  |
| Nrep | neuronal regeneration related | 4 | 0.0001463 | 0.007649 | 0.3587 | -1.479 | -4.674 |  |
| Ly6a | lymphocyte antigen 6 complex, | 3 | 1.495e-05 | 0.001467 | 0.467 | -1.098 | -5.675 |  |
| Mfsd7c | NA | 1 | 2.628e-06 | 0.0004251 | 2.978 | 1.574 | 6.471 |  |
| Acsl3 | acyl-CoA synthetase long-chain | 5 | 7.721e-07 | 0.0001628 | 0.2909 | -1.781 | -7.055 |  |
| Acot3 | acyl-CoA thioesterase 3 | 1 | 1.112e-06 | 0.0002253 | 4.5 | 2.17 | 6.879 |  |
| Pex11a | peroxisomal biogenesis factor | 3 | 6.609e-06 | 0.0008253 | 2.072 | 1.051 | 6.044 |  |
| Sec61g | SEC61, gamma subunit | 3 | 5.789e-06 | 0.0007521 | 0.4893 | -1.031 | -6.105 |  |
| Baiap2 | brain-specific angiogenesis in | 5 | 1.357e-05 | 0.001385 | 2.349 | 1.232 | 5.718 |  |
| Rhbdd2 | rhomboid domain containing 2 | 5 | 7.088e-06 | 0.0008641 | 2.082 | 1.058 | 6.012 |  |
| Cyp26a1 | cytochrome P450, family 26, su | 1 | 1.81e-05 | 0.001687 | 0.1051 | -3.25 | -5.589 |  |
| Acss2 | acyl-CoA synthetase short-chai | 3 | 4.073e-07 | 9.7e-05 | 0.2484 | -2.009 | -7.37 |  |
| 2010003K11Rik | RIKEN cDNA 2010003K11 gene | 1 | 6.996e-06 | 0.0008569 | 3.022 | 1.596 | 6.018 |  |
| Shank2 | SH3 and multiple ankyrin repea | 1 | 1.026e-05 | 0.001117 | 0.4327 | -1.209 | -5.844 |  |
| Ttc39c | tetratricopeptide repeat domai | 2 | 6.431e-06 | 0.0008109 | 0.4481 | -1.158 | -6.057 |  |
| Treh | trehalase (brush-border membra | 1 | 1.981e-05 | 0.001822 | 2.027 | 1.02 | 5.549 |  |
| Gm25783 | predicted gene, 25783 | 1 | 8.342e-06 | 0.000973 | 2.511 | 1.328 | 5.938 |  |
| Glrx | glutaredoxin | 5 | 4.941e-06 | 0.0006761 | 2.799 | 1.485 | 6.178 |  |
| 1810010H24Rik | RIKEN cDNA 1810010H24 gene | 5 | 1.531e-05 | 0.001485 | 2.31 | 1.208 | 5.664 |  |
| Prelid2 | PRELI domain containing 2 | 1 | 4.984e-06 | 0.0006783 | 2.227 | 1.155 | 6.174 |  |
| Gbp2b | guanylate binding protein 2b | 1 | 1.195e-05 | 0.00125 | 0.3825 | -1.386 | -5.775 |  |
| Zfand4 | zinc finger, AN1-type domain 4 | 2 | 2.187e-05 | 0.001927 | 0.4441 | -1.171 | -5.505 |  |
| Dsg1a | desmoglein 1 alpha | 1 | 1.683e-05 | 0.001607 | 0.4974 | -1.007 | -5.622 |  |
| Gstm6 | glutathione S-transferase, mu | 2 | 1.84e-06 | 0.000331 | 0.3344 | -1.58 | -6.639 |  |
| Plin5 | perilipin 5 | 2 | 3.944e-09 | 2.898e-06 | 4.967 | 2.312 | 9.876 |  |
| Cyp2c38 | cytochrome P450, family 2, sub | 2 | 2.423e-05 | 0.002099 | 2.788 | 1.479 | 5.459 |  |
| 1810011O10Rik | NA | 5 | 4.53e-06 | 0.0006368 | 4.414 | 2.142 | 6.218 |  |
| Foxq1 | forkhead box Q1 | 1 | 2.204e-05 | 0.001928 | 0.1531 | -2.708 | -5.502 |  |
| Cpt1a | carnitine palmitoyltransferase | 3 | 1.272e-06 | 0.0002442 | 2.508 | 1.327 | 6.814 |  |
| Slc1a4 | solute carrier family 1 (gluta | 1 | 2.054e-05 | 0.001873 | 0.4295 | -1.219 | -5.533 |  |
| Xkr9 | X-linked Kx blood group relate | 2 | 1.772e-05 | 0.001664 | 0.4975 | -1.007 | -5.599 |  |
| F11 | coagulation factor XI | 2 | 8.447e-06 | 0.0009743 | 0.4333 | -1.206 | -5.932 |  |
| Gfra1 | glial cell line derived neurot | 2 | 3.777e-05 | 0.002927 | 2.248 | 1.169 | 5.263 |  |
| Cerkl | ceramide kinase-like | 1 | 1.382e-05 | 0.001399 | 2.596 | 1.376 | 5.71 |  |
| Fam198a | NA | 1 | 2.49e-05 | 0.002142 | 0.3659 | -1.45 | -5.447 |  |
| Sfxn2 | sideroflexin 2 | 5 | 3.956e-05 | 0.002984 | 2.282 | 1.19 | 5.243 |  |
| Acsm3 | acyl-CoA synthetase medium-cha | 5 | 0.0006716 | 0.02291 | 2.132 | 1.092 | 4.021 |  |
| Rbm3 | RNA binding motif (RNP1, RRM) | 3 | 1.492e-05 | 0.001467 | 2.242 | 1.165 | 5.676 |  |
| Gk | glycerol kinase | 2 | 1.107e-05 | 0.001177 | 2.399 | 1.262 | 5.81 |  |
| Baiap2l1 | BAI1-associated protein 2-like | 2 | 2.72e-05 | 0.002279 | 2.537 | 1.343 | 5.408 |  |
| Acot4 | acyl-CoA thioesterase 4 | 2 | 1.291e-05 | 0.001328 | 3.316 | 1.73 | 5.741 |  |
| Wdr6 | WD repeat domain 6 | 1 | 3.68e-06 | 0.0005466 | 0.4665 | -1.1 | -6.314 |  |
| H2-Q10 | histocompatibility 2, Q region | 2 | 3.622e-05 | 0.002849 | 0.4937 | -1.018 | -5.282 |  |
| Slc41a2 | solute carrier family 41, memb | 2 | 4.826e-05 | 0.003412 | 0.3022 | -1.727 | -5.156 |  |
| Foxi1 | forkhead box I1 | 1 | 2.081e-05 | 0.001876 | 2.475 | 1.307 | 5.527 |  |
| Slc25a47 | solute carrier family 25, memb | 2 | 2.143e-05 | 0.001901 | 2.348 | 1.231 | 5.514 |  |
| Gm6781 | predicted gene 6781 | 3 | 4.44e-05 | 0.003245 | 0.3577 | -1.483 | -5.192 |  |
| Lipg | lipase, endothelial | 1 | 1.243e-05 | 0.001289 | 3.657 | 1.871 | 5.758 |  |
| Cyp4a14 | cytochrome P450, family 4, sub | 2 | 4.751e-05 | 0.003376 | 3.125 | 1.644 | 5.163 |  |
| Slc25a37 | solute carrier family 25, memb | 4 | 1.735e-05 | 0.001646 | 0.393 | -1.347 | -5.608 |  |
| Adam11 | a disintegrin and metallopepti | 1 | 5.411e-05 | 0.00374 | 0.3964 | -1.335 | -5.106 |  |
| Acly | ATP citrate lyase | 3 | 0.0002587 | 0.01154 | 0.3303 | -1.598 | -4.429 |  |
| Aldh4a1 | aldehyde dehydrogenase 4 famil | 4 | 2.207e-09 | 1.949e-06 | 0.4169 | -1.262 | -10.22 |  |
| Map3k5 | mitogen-activated protein kina | 5 | 8.962e-06 | 0.001016 | 2.336 | 1.224 | 5.905 |  |
| Pfkfb1 | 6-phosphofructo-2-kinase/fruct | 3 | 2.074e-05 | 0.001876 | 2.088 | 1.062 | 5.529 |  |
| Slc35g1 | solute carrier family 35, memb | 5 | 4.465e-05 | 0.003249 | 2.041 | 1.029 | 5.19 |  |
| Cutal | cutA divalent cation tolerance | 1 | 1.548e-05 | 0.001491 | 0.4849 | -1.044 | -5.659 |  |
| Slco1a1 | solute carrier organic anion t | 2 | 2.324e-05 | 0.002026 | 0.4277 | -1.225 | -5.478 |  |
| Fst | follistatin | 5 | 7.115e-05 | 0.004645 | 0.3639 | -1.458 | -4.986 |  |
| Dhcr7 | 7-dehydrocholesterol reductase | 2 | 1.137e-08 | 5.624e-06 | 0.2402 | -2.058 | -9.265 |  |
| Slc25a20 | solute carrier family 25 (mito | 3 | 7.326e-06 | 0.0008889 | 2.072 | 1.051 | 5.997 |  |
| Insc | INSC spindle orientation adapt | 1 | 2.025e-05 | 0.001854 | 0.4308 | -1.215 | -5.539 |  |
| 2900026A02Rik | RIKEN cDNA 2900026A02 gene | 5 | 3.075e-05 | 0.002501 | 0.4919 | -1.024 | -5.354 |  |
| Mgll | monoglyceride lipase | 3 | 8.419e-05 | 0.005248 | 2.679 | 1.422 | 4.913 |  |
| Serpina12 | serine (or cysteine) peptidase | 1 | 3.487e-05 | 0.002777 | 0.3621 | -1.466 | -5.299 |  |
| Chrna2 | cholinergic receptor, nicotini | 1 | 8.901e-06 | 0.001015 | 4.702 | 2.233 | 5.909 |  |
| E230001N04Rik | RIKEN cDNA E230001N04 gene | 1 | 1.764e-08 | 8.061e-06 | 6.383 | 2.674 | 9.018 |  |
| Stard4 | StAR-related lipid transfer (S | 2 | 3.906e-05 | 0.002982 | 0.3444 | -1.538 | -5.249 |  |
| Ugt1a7c | UDP glucuronosyltransferase 1 | 5 | 1.983e-05 | 0.001822 | 8.796 | 3.137 | 5.549 |  |
| Atl2 | atlastin GTPase 2 | 3 | 6.245e-05 | 0.004205 | 2.109 | 1.076 | 5.043 |  |
| Meiob | meiosis specific with OB domai | 2 | 4.203e-05 | 0.003122 | 0.4592 | -1.123 | -5.216 |  |
| Stk38l | serine/threonine kinase 38 lik | 5 | 1.455e-05 | 0.001455 | 0.4259 | -1.232 | -5.687 |  |
| Slc45a3 | solute carrier family 45, memb | 2 | 2.046e-06 | 0.0003534 | 2.989 | 1.58 | 6.589 |  |
| Lgals1 | lectin, galactose binding, sol | 3 | 2.086e-05 | 0.001876 | 0.389 | -1.362 | -5.526 |  |
| Ivns1abp | influenza virus NS1A binding p | 3 | 2.992e-07 | 7.774e-05 | 2.519 | 1.333 | 7.524 |  |
| Rrm2 | ribonucleotide reductase M2 | 1 | 1.808e-05 | 0.001687 | 2.242 | 1.165 | 5.59 |  |
| Dsg1c | desmoglein 1 gamma | 1 | 2.645e-05 | 0.002238 | 0.4111 | -1.282 | -5.421 |  |
| Cyp2c29 | cytochrome P450, family 2, sub | 2 | 4.38e-05 | 0.003228 | 0.4201 | -1.251 | -5.198 |  |
| Aim1 | NA | 2 | 2.95e-06 | 0.0004571 | 3.359 | 1.748 | 6.417 |  |
| Serpina9 | serine (or cysteine) peptidase | 1 | 3.922e-05 | 0.002982 | 0.4727 | -1.081 | -5.247 |  |
| Tfpi2 | tissue factor pathway inhibito | 2 | 5.423e-05 | 0.00374 | 0.4681 | -1.095 | -5.105 |  |
| Me1 | malic enzyme 1, NADP(+)-depend | 3 | 0.0003114 | 0.01322 | 0.4702 | -1.089 | -4.35 |  |
| Gngt1 | guanine nucleotide binding pro | 1 | 5.246e-05 | 0.00365 | 0.4986 | -1.004 | -5.119 |  |
| Gm15401 | predicted gene 15401 | 1 | 9.992e-05 | 0.005963 | 2.703 | 1.435 | 4.839 |  |
| Acss3 | acyl-CoA synthetase short-chai | 3 | 5.439e-06 | 0.0007262 | 2.812 | 1.491 | 6.133 |  |
| Nipal1 | NIPA-like domain containing 1 | 1 | 7.787e-05 | 0.004958 | 3.53 | 1.82 | 4.947 |  |
| Cyp2u1 | cytochrome P450, family 2, sub | 5 | 8.671e-07 | 0.0001784 | 0.3664 | -1.448 | -6.999 |  |
| C77080 | expressed sequence C77080 | 2 | 4.691e-05 | 0.003375 | 0.4773 | -1.067 | -5.168 |  |
| Tubb4b | tubulin, beta 4B class IVB | 3 | 9.113e-05 | 0.005555 | 0.4011 | -1.318 | -4.879 |  |
| Cyp2a22 | cytochrome P450, family 2, sub | 1 | 4.039e-05 | 0.00302 | 4.751 | 2.248 | 5.234 |  |
| Cidec | cell death-inducing DFFA-like | 3 | 6.863e-05 | 0.004527 | 10.61 | 3.407 | 5.002 |  |
| Tsku | tsukushi, small leucine rich p | 1 | 4.737e-05 | 0.003375 | 3.599 | 1.848 | 5.164 |  |
| Grn | granulin | 3 | 2.105e-05 | 0.001887 | 2.728 | 1.448 | 5.522 |  |
| Aifm2 | apoptosis-inducing factor, mit | 5 | 1.494e-05 | 0.001467 | 2.58 | 1.367 | 5.675 |  |
| Mmab | methylmalonic aciduria (cobala | 4 | 1.687e-05 | 0.001607 | 0.3209 | -1.64 | -5.621 |  |
| Pgm3 | phosphoglucomutase 3 | 5 | 6.452e-05 | 0.004334 | 2.246 | 1.167 | 5.029 |  |
| Fkbp5 | FK506 binding protein 5 | 4 | 4.289e-09 | 3.065e-06 | 13.18 | 3.72 | 9.827 |  |
| Por | P450 (cytochrome) oxidoreducta | 3 | 1.13e-05 | 0.001191 | 2.534 | 1.341 | 5.801 |  |
| Lgals4 | lectin, galactose binding, sol | 5 | 2.823e-07 | 7.409e-05 | 4.067 | 2.024 | 7.553 |  |
| Erc2 | ELKS/RAB6-interacting/CAST fam | 1 | 0.0001114 | 0.006424 | 0.4961 | -1.011 | -4.792 |  |
| Bdh2 | 3-hydroxybutyrate dehydrogenas | 2 | 0.0001969 | 0.009483 | 0.409 | -1.29 | -4.546 |  |
| Tmtc2 | transmembrane and tetratricope | 1 | 0.0001736 | 0.008606 | 2.537 | 1.343 | 4.6 |  |
| Fam71e1 | family with sequence similarit | 1 | 3.942e-05 | 0.002982 | 2.018 | 1.013 | 5.245 |  |
| Camk2b | calcium/calmodulin-dependent p | 4 | 0.0001573 | 0.008078 | 2.811 | 1.491 | 4.643 |  |
| Gstp1 | glutathione S-transferase, pi | 3 | 4.515e-05 | 0.003271 | 0.3544 | -1.496 | -5.185 |  |
| Man2a2 | mannosidase 2, alpha 2 | 3 | 1.026e-05 | 0.001117 | 0.4993 | -1.002 | -5.844 |  |
| Ces2c | carboxylesterase 2C | 1 | 0.0001175 | 0.006645 | 0.3208 | -1.64 | -4.769 |  |
| Tubb5 | tubulin, beta 5 class I | 3 | 0.0002904 | 0.01253 | 0.4417 | -1.179 | -4.38 |  |
| Nek6 | NIMA (never in mitosis gene a) | 5 | 3.904e-05 | 0.002982 | 0.405 | -1.304 | -5.249 |  |
| Nrg4 | neuregulin 4 | 5 | 6.163e-05 | 0.004172 | 3.389 | 1.761 | 5.049 |  |
| Slc25a34 | solute carrier family 25, memb | 1 | 4.146e-06 | 0.0005958 | 3.423 | 1.775 | 6.259 |  |
| Fmo2 | flavin containing monooxygenas | 3 | 7.893e-10 | 9.668e-07 | 6.142 | 2.619 | 10.86 |  |
| Gas7 | growth arrest specific 7 | 5 | 3.312e-05 | 0.002654 | 0.4501 | -1.152 | -5.321 |  |
| Tbc1d8 | TBC1 domain family, member 8 | 5 | 8.705e-05 | 0.00537 | 2.572 | 1.363 | 4.899 |  |
| Tubb4b-ps1 | tubulin, beta 4B class IVB, ps | 3 | 9.783e-05 | 0.005853 | 0.4315 | -1.212 | -4.848 |  |
| Alas2 | aminolevulinic acid synthase 2 | 5 | 0.0001603 | 0.008176 | 0.3388 | -1.562 | -4.635 |  |
| Lrp2bp | Lrp2 binding protein | 1 | 3.243e-06 | 0.0004995 | 0.3198 | -1.645 | -6.373 |  |
| Sult1d1 | sulfotransferase family 1D, me | 2 | 0.0001123 | 0.006428 | 3.168 | 1.664 | 4.789 |  |
| Sntg2 | syntrophin, gamma 2 | 1 | 6.195e-05 | 0.004182 | 0.4837 | -1.048 | -5.047 |  |
| Atp11a | ATPase, class VI, type 11A | 5 | 2.521e-05 | 0.002154 | 0.3398 | -1.557 | -5.442 |  |
| Atp5s | NA | 4 | 0.0001808 | 0.008841 | 2.369 | 1.244 | 4.583 |  |
| Cyp4a32 | cytochrome P450, family 4, sub | 2 | 0.0001381 | 0.007388 | 2.497 | 1.32 | 4.699 |  |
| Fbxl21 | F-box and leucine-rich repeat | 1 | 4.036e-05 | 0.00302 | 2.007 | 1.005 | 5.234 |  |
| Lyve1 | lymphatic vessel endothelial h | 5 | 3.686e-05 | 0.002873 | 3.872 | 1.953 | 5.274 |  |
| Gse1 | genetic suppressor element 1, | 5 | 2.848e-05 | 0.002363 | 0.4602 | -1.12 | -5.388 |  |
| Alpl | alkaline phosphatase, liver/bo | 5 | 0.001992 | 0.04842 | 2.531 | 1.34 | 3.554 |  |
| Adh6-ps1 | alcohol dehydrogenase 6 (class | 1 | 0.0001233 | 0.006873 | 0.3055 | -1.711 | -4.748 |  |
| Tspan7 | tetraspanin 7 | 3 | 1.29e-05 | 0.001328 | 0.4954 | -1.013 | -5.741 |  |
| Eepd1 | endonuclease/exonuclease/phosp | 3 | 0.0001406 | 0.007475 | 0.4035 | -1.309 | -4.691 |  |
| Il1r1 | interleukin 1 receptor, type I | 5 | 7.329e-05 | 0.004737 | 3.412 | 1.771 | 4.974 |  |
| Adam23 | a disintegrin and metallopepti | 5 | 0.000123 | 0.006873 | 3 | 1.585 | 4.749 |  |
| Gm21839 | predicted gene, 21839 | 1 | 0.0001988 | 0.00956 | 2.209 | 1.143 | 4.542 |  |
| Lce6a | late cornified envelope 6A | 1 | 0.0001038 | 0.006095 | 0.4817 | -1.054 | -4.823 |  |
| Cml5 | NA | 2 | 0.000129 | 0.007122 | 0.2911 | -1.78 | -4.728 |  |
| Mid1ip1 | Mid1 interacting protein 1 (ga | 5 | 8.042e-05 | 0.005095 | 0.3223 | -1.633 | -4.933 |  |
| Tmem180 | NA | 1 | 6.147e-05 | 0.004172 | 0.4688 | -1.093 | -5.05 |  |
| B230340J04Rik | NA | 1 | 5.25e-05 | 0.00365 | 2.58 | 1.367 | 5.119 |  |
| Slc25a32 | solute carrier family 25, memb | 4 | 2.733e-05 | 0.002281 | 2.268 | 1.182 | 5.406 |  |
| Arv1 | ARV1 homolog, fatty acid homeo | 1 | 4.224e-05 | 0.003122 | 2.051 | 1.036 | 5.214 |  |
| Gm34985 | NA | 1 | 7.495e-05 | 0.0048 | 3.908 | 1.966 | 4.964 |  |
| Abhd17c | abhydrolase domain containing | 5 | 2.203e-05 | 0.001928 | 0.4886 | -1.033 | -5.502 |  |
| Snord14e | small nucleolar RNA, C/D box 1 | 3 | 5.347e-05 | 0.003707 | 2.624 | 1.392 | 5.111 |  |
| Abhd2 | abhydrolase domain containing | 5 | 1.75e-05 | 0.001655 | 3.829 | 1.937 | 5.604 |  |
| Fmo3 | flavin containing monooxygenas | 1 | 0.0002005 | 0.009604 | 3.204 | 1.68 | 4.539 |  |
| Slc25a51 | solute carrier family 25, memb | 4 | 0.0001326 | 0.007225 | 2.218 | 1.149 | 4.717 |  |
| Hsd17b6 | hydroxysteroid (17-beta) dehyd | 2 | 0.0001577 | 0.008081 | 0.4623 | -1.113 | -4.642 |  |
| Hn1l | NA | 5 | 1.465e-05 | 0.001455 | 0.4198 | -1.252 | -5.684 |  |
| Tango2 | transport and golgi organizati | 3 | 2.462e-05 | 0.002125 | 2.367 | 1.243 | 5.452 |  |
| Dpy19l1 | dpy-19-like 1 (C. elegans) | 5 | 0.000219 | 0.0103 | 2.037 | 1.026 | 4.501 |  |
| Golt1a | golgi transport 1A | 1 | 0.0003822 | 0.01522 | 2.214 | 1.147 | 4.262 |  |
| Lsr | lipolysis stimulated lipoprote | 2 | 0.0003016 | 0.01291 | 0.4931 | -1.02 | -4.363 |  |
| Gm3579 | NA | 3 | 0.0001718 | 0.008561 | 5.186 | 2.375 | 4.605 |  |
| Gck | glucokinase | 2 | 0.0002511 | 0.01134 | 0.222 | -2.171 | -4.442 |  |
| Creld2 | cysteine-rich with EGF-like do | 5 | 0.0003763 | 0.01503 | 2.234 | 1.16 | 4.269 |  |
| Setdb2 | SET domain, bifurcated 2 | 5 | 8.285e-05 | 0.005194 | 2.656 | 1.409 | 4.92 |  |
| Vnn1 | vanin 1 | 5 | 0.001814 | 0.04543 | 2.972 | 1.571 | 3.595 |  |
| Fam214a | family with sequence similarit | 3 | 1.624e-06 | 0.0003027 | 3.029 | 1.599 | 6.698 |  |
| Slc22a30 | solute carrier family 22, memb | 2 | 0.0002437 | 0.01109 | 0.3904 | -1.357 | -4.455 |  |
| Mir185 | microRNA 185 | 5 | 5.875e-06 | 0.0007594 | 2.985 | 1.578 | 6.098 |  |
| BC021614 | NA | 2 | 0.000714 | 0.02401 | 0.492 | -1.023 | -3.994 |  |
| Per1 | period circadian clock 1 | 4 | 3.353e-11 | 9.583e-08 | 5.591 | 2.483 | 12.99 |  |
| Atf5 | activating transcription facto | 5 | 0.0004529 | 0.01731 | 2.202 | 1.139 | 4.189 |  |
| Nt5dc2 | 5'-nucleotidase domain contain | 5 | 0.0003874 | 0.0154 | 2.842 | 1.507 | 4.256 |  |
| Pole4 | polymerase (DNA-directed), eps | 3 | 0.0003166 | 0.01333 | 2.057 | 1.041 | 4.343 |  |
| Steap4 | STEAP family member 4 | 3 | 0.001261 | 0.03632 | 0.471 | -1.086 | -3.751 |  |
| Sulf2 | sulfatase 2 | 3 | 0.000865 | 0.02761 | 2.369 | 1.244 | 3.912 |  |
| Nr1d1 | nuclear receptor subfamily 1, | 3 | 1.536e-05 | 0.001485 | 0.3426 | -1.545 | -5.663 |  |
| Nid2 | nidogen 2 | 5 | 2.541e-05 | 0.002164 | 0.4093 | -1.289 | -5.438 |  |
| Rell1 | RELT-like 1 | 5 | 0.000102 | 0.006022 | 0.4863 | -1.04 | -4.83 |  |
| Aldoc | aldolase C, fructose-bisphosph | 1 | 0.0001769 | 0.008686 | 0.3876 | -1.367 | -4.592 |  |
| Gm826 | predicted gene 826 | 1 | 0.000105 | 0.006142 | 2.752 | 1.46 | 4.817 |  |
| Gata6 | GATA binding protein 6 | 5 | 0.0001333 | 0.007234 | 2.347 | 1.231 | 4.714 |  |
| Erbb4 | erb-b2 receptor tyrosine kinas | 1 | 0.000123 | 0.006873 | 0.4363 | -1.196 | -4.749 |  |
| Pnpla2 | patatin-like phospholipase dom | 3 | 2.15e-05 | 0.001901 | 2.071 | 1.05 | 5.512 |  |
| Cyp7a1 | cytochrome P450, family 7, sub | 2 | 0.0007138 | 0.02401 | 2.388 | 1.256 | 3.995 |  |
| Fcamr | Fc receptor, IgA, IgM, high af | 1 | 0.0001348 | 0.007282 | 2.817 | 1.494 | 4.71 |  |
| Fam13a | family with sequence similarit | 3 | 0.001285 | 0.03672 | 2.555 | 1.353 | 3.743 |  |
| Echdc1 | enoyl Coenzyme A hydratase dom | 3 | 4.859e-05 | 0.003425 | 0.3969 | -1.333 | -5.153 |  |
| Apol9b | apolipoprotein L 9b | 1 | 0.0002293 | 0.01067 | 0.4861 | -1.041 | -4.481 |  |
| Loxl4 | lysyl oxidase-like 4 | 1 | 0.0003357 | 0.014 | 0.4689 | -1.093 | -4.317 |  |
| Cxx1a | NA | 5 | 0.0001018 | 0.006022 | 0.4705 | -1.088 | -4.831 |  |
| Slc13a2 | solute carrier family 13 (sodi | 1 | 0.0002644 | 0.01171 | 0.4147 | -1.27 | -4.42 |  |
| Slc16a7 | solute carrier family 16 (mono | 5 | 0.0008638 | 0.02761 | 2.463 | 1.3 | 3.913 |  |
| Abca8b | ATP-binding cassette, sub-fami | 5 | 3.933e-05 | 0.002982 | 0.428 | -1.224 | -5.246 |  |
| Vtcn1 | V-set domain containing T cell | 1 | 0.0006629 | 0.02273 | 0.4969 | -1.009 | -4.026 |  |
| Zfp9 | zinc finger protein 9 | 5 | 0.0007227 | 0.02408 | 2.182 | 1.125 | 3.989 |  |
| Trim24 | tripartite motif-containing 24 | 4 | 0.0004444 | 0.01704 | 2.124 | 1.087 | 4.197 |  |
| Gm4184 | NA | 3 | 0.0003173 | 0.01333 | 0.4594 | -1.122 | -4.342 |  |
| C730034F03Rik | RIKEN cDNA C730034F03 gene | 1 | 0.0001295 | 0.007127 | 2.105 | 1.074 | 4.727 |  |
| Cdh1 | cadherin 1 | 2 | 0.0004019 | 0.01584 | 0.4347 | -1.202 | -4.24 |  |
| Cxx1b | NA | 5 | 0.0001234 | 0.006873 | 0.4728 | -1.081 | -4.748 |  |
| Cyp2c39 | cytochrome P450, family 2, sub | 1 | 0.0005397 | 0.01964 | 2.568 | 1.361 | 4.114 |  |
| Cirbp | cold inducible RNA binding pro | 4 | 4.064e-05 | 0.00303 | 2.395 | 1.26 | 5.231 |  |
| Dclre1b | DNA cross-link repair 1B | 5 | 1.039e-05 | 0.001119 | 2.101 | 1.071 | 5.838 |  |
| Tmem150a | transmembrane protein 150A | 4 | 6.775e-05 | 0.004492 | 0.4627 | -1.112 | -5.008 |  |
| Gm16412 | predicted pseudogene 16412 | 3 | 0.0003992 | 0.0158 | 0.4457 | -1.166 | -4.243 |  |
| Coa6 | cytochrome c oxidase assembly | 1 | 0.0006385 | 0.02219 | 2.795 | 1.483 | 4.042 |  |
| Gm22645 | predicted gene, 22645 | 1 | 0.0001059 | 0.006165 | 0.4718 | -1.084 | -4.814 |  |
| Enpep | glutamyl aminopeptidase | 3 | 0.001279 | 0.03663 | 0.4853 | -1.043 | -3.745 |  |
| Mt2 | metallothionein 2 | 3 | 0.0003033 | 0.01294 | 6.053 | 2.598 | 4.361 |  |
| Ccnd3 | cyclin D3 | 5 | 0.0001083 | 0.006288 | 2.484 | 1.312 | 4.804 |  |
| Mir3064 | microRNA 3064 | 3 | 0.0001171 | 0.006634 | 2.029 | 1.021 | 4.77 |  |
| Fam134b | NA | 4 | 7.251e-05 | 0.004698 | 2.27 | 1.182 | 4.978 |  |
| Car1 | carbonic anhydrase 1 | 5 | 0.0004872 | 0.01834 | 0.3152 | -1.666 | -4.158 |  |
| Tsc22d3 | TSC22 domain family, member 3 | 4 | 8.694e-06 | 0.0009984 | 2.84 | 1.506 | 5.919 |  |
| Gm6762 | predicted pseudogene 6762 | 1 | 0.000203 | 0.009678 | 0.4798 | -1.059 | -4.533 |  |
| Lfng | LFNG O-fucosylpeptide 3-beta-N | 1 | 0.001037 | 0.03182 | 2.208 | 1.143 | 3.835 |  |
| Bnip3 | BCL2/adenovirus E1B interactin | 3 | 5.039e-06 | 0.0006822 | 2.337 | 1.225 | 6.169 |  |
| Sorbs3 | sorbin and SH3 domain containi | 4 | 3.665e-05 | 0.002865 | 2.49 | 1.316 | 5.277 |  |
| Gm42368 | NA | 3 | 0.0005114 | 0.01901 | 3.84 | 1.941 | 4.137 |  |
| 3010026O09Rik | NA | 5 | 0.000244 | 0.01109 | 0.3473 | -1.526 | -4.454 |  |
| Fgf1 | fibroblast growth factor 1 | 5 | 0.000373 | 0.01502 | 2.019 | 1.014 | 4.272 |  |
| Slc4a1 | solute carrier family 4 (anion | 1 | 0.0001193 | 0.006732 | 0.4962 | -1.011 | -4.762 |  |
| Ppip5k2 | diphosphoinositol pentakisphos | 5 | 0.0006685 | 0.02284 | 2.044 | 1.031 | 4.023 |  |
| Gm9824 | predicted pseudogene 9824 | 3 | 2.344e-05 | 0.002037 | 0.4535 | -1.141 | -5.474 |  |
| Hba-a1 | hemoglobin alpha, adult chain | 3 | 0.000491 | 0.01841 | 0.3816 | -1.39 | -4.155 |  |
| Ociad2 | OCIA domain containing 2 | 4 | 2.696e-05 | 0.002266 | 0.4214 | -1.247 | -5.412 |  |
| Mmp15 | matrix metallopeptidase 15 | 5 | 4.709e-05 | 0.003375 | 0.4336 | -1.206 | -5.167 |  |
| Pmf1 | polyamine-modulated factor 1 | 5 | 0.0003708 | 0.01497 | 0.4524 | -1.144 | -4.275 |  |
| Psmb10 | proteasome (prosome, macropain | 5 | 0.001159 | 0.03432 | 0.4794 | -1.061 | -3.787 |  |
| Gale | galactose-4-epimerase, UDP | 1 | 0.0001029 | 0.006057 | 0.4508 | -1.149 | -4.826 |  |
| Dhrs9 | dehydrogenase/reductase (SDR f | 1 | 0.001916 | 0.04706 | 0.4411 | -1.181 | -3.571 |  |
| Lin7a | lin-7 homolog A (C. elegans) | 5 | 0.001448 | 0.03946 | 0.4585 | -1.125 | -3.692 |  |
| Cntrl | centriolin | 5 | 0.0001813 | 0.008847 | 2.19 | 1.131 | 4.582 |  |
| Prkch | protein kinase C, eta | 5 | 1.856e-05 | 0.001723 | 2.271 | 1.183 | 5.578 |  |
| Gm22887 | predicted gene, 22887 | 1 | 0.0005222 | 0.01924 | 2.276 | 1.187 | 4.128 |  |
| Rpusd3 | RNA pseudouridylate synthase d | 5 | 0.0001526 | 0.0079 | 0.4791 | -1.062 | -4.656 |  |
| Gm379 | predicted gene 379 | 1 | 0.0003496 | 0.01428 | 0.4602 | -1.12 | -4.3 |  |
| C4bp-ps1 | complement component 4 binding | 1 | 0.0009726 | 0.03033 | 2.193 | 1.133 | 3.862 |  |
| Hba-a2 | hemoglobin alpha, adult chain | 3 | 0.0006635 | 0.02273 | 0.3711 | -1.43 | -4.026 |  |
| Mtmr11 | myotubularin related protein 1 | 1 | 0.001327 | 0.03726 | 0.4358 | -1.198 | -3.729 |  |
| Mir107 | microRNA 107 | 1 | 0.0005255 | 0.01931 | 0.4361 | -1.197 | -4.126 |  |
| Cyp2d40 | cytochrome P450, family 2, sub | 2 | 0.0005546 | 0.02005 | 0.472 | -1.083 | -4.103 |  |
| Ctgf | NA | 5 | 0.0001667 | 0.00838 | 4.702 | 2.233 | 4.618 |  |
| Ppargc1a | peroxisome proliferative activ | 4 | 0.001161 | 0.03432 | 2.542 | 1.346 | 3.786 |  |
| Hbb-bt | hemoglobin, beta adult t chain | 3 | 0.000589 | 0.02101 | 0.4676 | -1.097 | -4.077 |  |
| Txnip | thioredoxin interacting protei | 3 | 0.0003415 | 0.0141 | 2.214 | 1.147 | 4.31 |  |
| Acat2 | acetyl-Coenzyme A acetyltransf | 5 | 0.000648 | 0.02236 | 0.4059 | -1.301 | -4.036 |  |
| Fam107a | family with sequence similarit | 1 | 1.386e-06 | 0.000264 | 5.366 | 2.424 | 6.774 |  |
| Sult1e1 | sulfotransferase family 1E, me | 5 | 0.001246 | 0.03609 | 3.788 | 1.922 | 3.756 |  |
| Gm5785 | NA | 4 | 8.299e-05 | 0.005194 | 2.225 | 1.154 | 4.919 |  |
| Rasgef1b | RasGEF domain family, member 1 | 2 | 0.0001589 | 0.008128 | 3.432 | 1.779 | 4.639 |  |
| Hamp2 | hepcidin antimicrobial peptide | 5 | 0.0004815 | 0.01822 | 0.3891 | -1.362 | -4.163 |  |
| Gm9958 | predicted gene 9958 | 5 | 0.0002414 | 0.01103 | 2.067 | 1.048 | 4.459 |  |
| Taf1a | TATA-box binding protein assoc | 4 | 0.0001443 | 0.007607 | 2.144 | 1.101 | 4.68 |  |
| Asns | asparagine synthetase | 5 | 0.001007 | 0.03118 | 4.275 | 2.096 | 3.847 |  |
| Gm14964 | predicted gene 14964 | 1 | 0.0002449 | 0.01111 | 2.273 | 1.184 | 4.453 |  |
| Man2a1 | mannosidase 2, alpha 1 | 3 | 7.17e-05 | 0.004669 | 2.041 | 1.03 | 4.983 |  |
| Clec9a | C-type lectin domain family 9, | 1 | 0.0001881 | 0.009131 | 2.918 | 1.545 | 4.566 |  |
| Hbb-bs | hemoglobin, beta adult s chain | 3 | 0.0008811 | 0.02798 | 0.4197 | -1.252 | -3.905 |  |
| Gm42035 | NA | 3 | 0.001269 | 0.03648 | 2.869 | 1.521 | 3.748 |  |
| Itgb3 | integrin beta 3 | 1 | 0.000282 | 0.0123 | 2.035 | 1.025 | 4.392 |  |
| BC023105 | cDNA sequence BC023105 | 4 | 0.0005213 | 0.01924 | 0.3369 | -1.57 | -4.129 |  |
| Ighv1-54 | immunoglobulin heavy variable | 1 | 0.0008587 | 0.02751 | 5.647 | 2.498 | 3.916 |  |
| Got1 | glutamic-oxaloacetic transamin | 4 | 0.0004685 | 0.0178 | 2.091 | 1.064 | 4.175 |  |
| Med4 | mediator complex subunit 4 | 4 | 0.0005722 | 0.02053 | 2.036 | 1.026 | 4.089 |  |
| Derl3 | Der1-like domain family, membe | 1 | 0.0002518 | 0.01134 | 3.587 | 1.843 | 4.441 |  |
| Gm16418 | predicted pseudogene 16418 | 3 | 0.0004152 | 0.01625 | 0.3729 | -1.423 | -4.227 |  |
| Chst15 | carbohydrate sulfotransferase | 5 | 0.0002756 | 0.01214 | 2.671 | 1.418 | 4.402 |  |
| Clec1b | C-type lectin domain family 1, | 2 | 0.0001373 | 0.007357 | 2.051 | 1.036 | 4.702 |  |
| mt-Tm | mitochondrially encoded tRNA m | 3 | 0.001165 | 0.0344 | 0.1752 | -2.513 | -3.785 |  |
| Onecut1 | one cut domain, family member | 1 | 0.001897 | 0.04683 | 0.1478 | -2.758 | -3.575 |  |
| Lin9 | lin-9 homolog (C. elegans) | 5 | 0.0008108 | 0.0262 | 0.4358 | -1.198 | -3.94 |  |
| Slco1a4 | solute carrier organic anion t | 2 | 0.001434 | 0.03923 | 2.482 | 1.311 | 3.696 |  |
| Gm23462 | predicted gene, 23462 | 5 | 0.0004762 | 0.01804 | 2.9 | 1.536 | 4.168 |  |
| Pim1 | proviral integration site 1 | 4 | 0.001653 | 0.04312 | 0.4558 | -1.133 | -3.635 |  |
| Lepr | leptin receptor | 1 | 0.0003174 | 0.01333 | 3.877 | 1.955 | 4.341 |  |
| 5430403G16Rik | RIKEN cDNA 5430403G16 gene | 1 | 0.0005954 | 0.0211 | 0.46 | -1.12 | -4.072 |  |
| Nudt2 | nudix (nucleoside diphosphate | 4 | 0.0002742 | 0.0121 | 0.4946 | -1.016 | -4.404 |  |
| Mir122 | microRNA 122 | 2 | 0.001442 | 0.03939 | 0.3505 | -1.513 | -3.693 |  |
| Tmem254c | transmembrane protein 254c | 1 | 0.001124 | 0.03367 | 2.145 | 1.101 | 3.8 |  |
| Gsta4 | glutathione S-transferase, alp | 5 | 8.9e-05 | 0.005449 | 0.4488 | -1.156 | -4.889 |  |
| Gm9870 | predicted gene 9870 | 1 | 0.001751 | 0.04451 | 0.4582 | -1.126 | -3.61 |  |
| Traj7 | T cell receptor alpha joining | 1 | 0.0007292 | 0.0242 | 0.3095 | -1.692 | -3.985 |  |
| Fzd7 | frizzled class receptor 7 | 4 | 0.0009871 | 0.03063 | 0.4415 | -1.18 | -3.856 |  |
| n-R5s193 | nuclear encoded rRNA 5S 193 | 5 | 0.001397 | 0.0384 | 2.317 | 1.212 | 3.707 |  |
| Gstm4 | glutathione S-transferase, mu | 3 | 0.0006283 | 0.02193 | 0.4727 | -1.081 | -4.049 |  |
| Omd | osteomodulin | 1 | 0.001097 | 0.03336 | 2.442 | 1.288 | 3.811 |  |
| Banp | BTG3 associated nuclear protei | 5 | 0.00172 | 0.04406 | 2.253 | 1.172 | 3.617 |  |
| Col14a1 | collagen, type XIV, alpha 1 | 5 | 0.0007848 | 0.02562 | 0.4959 | -1.012 | -3.954 |  |
| Tirap | toll-interleukin 1 receptor (T | 4 | 0.0007847 | 0.02562 | 0.4276 | -1.226 | -3.954 |  |
| Dpep1 | dipeptidase 1 | 3 | 0.0007416 | 0.02455 | 2.199 | 1.137 | 3.978 |  |
| Apol11b | apolipoprotein L 11b | 1 | 0.002002 | 0.04862 | 0.4529 | -1.143 | -3.552 |  |
| Snord1c | small nucleolar RNA, C/D box 1 | 1 | 0.0003928 | 0.01557 | 2.122 | 1.085 | 4.25 |  |
| Gm24253 | predicted gene, 24253 | 1 | 0.0003928 | 0.01557 | 2.122 | 1.085 | 4.25 |  |
| Nr0b2 | nuclear receptor subfamily 0, | 2 | 0.001821 | 0.04551 | 0.4032 | -1.31 | -3.593 |  |
| Pcdhga7 | protocadherin gamma subfamily | 1 | 0.002012 | 0.04878 | 0.4813 | -1.055 | -3.55 |  |
| Sec61b | Sec61 beta subunit | 1 | 0.0008391 | 0.02701 | 0.4949 | -1.015 | -3.925 |  |
| Nceh1 | neutral cholesterol ester hydr | 4 | 0.001554 | 0.04155 | 2.115 | 1.081 | 3.661 |  |
| Mt1 | metallothionein 1 | 3 | 0.0007686 | 0.02525 | 3.815 | 1.932 | 3.963 |  |
| Gm12653 | predicted gene 12653 | 1 | 0.001321 | 0.03721 | 0.4811 | -1.056 | -3.731 |  |
| Prox1 | prospero homeobox 1 | 2 | 0.001818 | 0.04549 | 4.059 | 2.021 | 3.594 |  |
| Slc17a9 | solute carrier family 17, memb | 1 | 0.001634 | 0.04288 | 2.046 | 1.033 | 3.64 |  |
| Fasn | fatty acid synthase | 3 | 0.001935 | 0.04731 | 0.2477 | -2.013 | -3.567 |  |
| Dgka | diacylglycerol kinase, alpha | 4 | 0.001451 | 0.03946 | 2.667 | 1.415 | 3.691 |  |
