# Supplementary material for: GIANT: galaxy-based tool for interactive analysis of transcriptomic data
Source: Sci Rep. 2020 Nov 16;10:19835. doi: 10.1038/s41598-020-76769-w (PMC7670435; doi:10.1038/s41598-020-76769-w)
Supplement: Supplementary file 2 — Supplementary Information. [file 41598_2020_76769_MOESM2_ESM.zip › Tools_InputOutput_Parameters/MicroArray_workflow/Step6-Heatmap/Outputs/Heatmap_FastedVsFed-Liver_HTML.html_81.html]

### Clustering tabular

Clustering results

### Heatmap plot

Heatmap

### Scree plot

Scree plot

### Circular plot

Circular plot
