# Supplementary material for: GIANT: galaxy-based tool for interactive analysis of transcriptomic data
Source: Sci Rep. 2020 Nov 16;10:19835. doi: 10.1038/s41598-020-76769-w (PMC7670435; doi:10.1038/s41598-020-76769-w)
Supplement: Supplementary file 2 — Supplementary Information. [file 41598_2020_76769_MOESM2_ESM.zip › Tools_InputOutput_Parameters/RNA-seq_workflow/Step1-Normalization_Differential/Outputs/limma_on_data_2_and_data_1__Report_5.html]

### Limma Analysis Output:

Links to PDF copies of plots are in 'Plots' section below   


#### Differential Expression Counts:

|  |  |  |  |
| --- | --- | --- | --- |
|  | Up | Flat | Down |
| CDAA-CSAA | 1860 | 10540 | 576 |

#### Plots:

CpmPlots.pdf  
DensityPlots.pdf  
BoxPlots.pdf  
MDSPlot\_Diet.pdf  
MDSPlot\_extra.pdf  
MDPlots\_Samples.pdf  
VoomPlot.pdf  
SAPlot.pdf  
MDPlot\_CDAA-CSAA.pdf  
VolcanoPlot\_CDAA-CSAA.pdf  
Heatmap\_CDAA-CSAA.pdf  
Stripcharts\_CDAA-CSAA.pdf  

#### Tables:

limma-voom\_filtcounts.tsv  
limma-voom\_normcounts.tsv  
limma-voom\_CDAA-CSAA.tsv  

#### Glimma Interactive Results:

Glimma\_MDSPlot.html  

Alt-click links to download file.

Click floppy disc icon associated history item to download all files.

.tsv files can be viewed in Excel or any spreadsheet program.

#### Additional Information

- Genes without more than 1 CPM in at least 5 samples are insignificant and filtered out.
- 19686 of 32662 (60.27%) genes were filtered out for low expression.
- TMM was the method used to normalise library sizes.
- The limma-voom method was used.
- Weights were not applied to samples.
- Testing significance relative to a fold-change threshold (TREAT) was performed using a threshold of log2 = 1 at FDR of 0.05 .
- TREAT was used with robust settings (robust=TRUE).
- MD Plot highlighted genes are significant at FDR of 0.05 and exhibit log2-fold-change of at least 1.

#### Summary of experimental data:

\*CHECK THAT SAMPLES ARE ASSOCIATED WITH CORRECT GROUP(S)\*

| SampleID | Diet (Primary Factor) | Time |
| --- | --- | --- |
| 528\_1\_CSAA\_week\_4 | CSAA | X4weeks |
| 528\_2\_CSAA\_week\_4 | CSAA | X4weeks |
| 528\_3\_CSAA\_week\_4 | CSAA | X4weeks |
| 528\_4\_CSAA\_week\_4 | CSAA | X4weeks |
| 528\_5\_CSAA\_week\_4 | CSAA | X4weeks |
| 528\_6\_CSAA\_week\_4 | CSAA | X4weeks |
| 528\_7\_CSAA\_week\_4 | CSAA | X4weeks |
| 528\_8\_CSAA\_week\_4 | CSAA | X4weeks |
| 528\_17\_CDDA\_Chol\_1pc\_week\_4 | CDAA | X4weeks |
| 528\_18\_CDDA\_Chol\_1pc\_week\_4 | CDAA | X4weeks |
| 528\_19\_CDDA\_Chol\_1pc\_week\_4 | CDAA | X4weeks |
| 528\_20\_CDDA\_Chol\_1pc\_week\_4 | CDAA | X4weeks |
| 528\_21\_CDDA\_Chol\_1pc\_week\_4 | CDAA | X4weeks |
| 528\_22\_CDDA\_Chol\_1pc\_week\_4 | CDAA | X4weeks |
| 528\_23\_CDDA\_Chol\_1pc\_week\_4 | CDAA | X4weeks |
| 528\_24\_CDDA\_Chol\_1pc\_week\_4 | CDAA | X4weeks |
| 528\_33\_CSAA\_week\_8 | CSAA | X8weeks |
| 528\_34\_CSAA\_week\_8 | CSAA | X8weeks |
| 528\_35\_CSAA\_week\_8 | CSAA | X8weeks |
| 528\_36\_CSAA\_week\_8 | CSAA | X8weeks |
| 528\_37\_CSAA\_week\_8 | CSAA | X8weeks |
| 528\_38\_CSAA\_week\_8 | CSAA | X8weeks |
| 528\_39\_CSAA\_week\_8 | CSAA | X8weeks |
| 528\_40\_CSAA\_week\_8 | CSAA | X8weeks |
| 528\_49\_CDDA\_Chol\_1pc\_week\_8 | CDAA | X8weeks |
| 528\_50\_CDDA\_Chol\_1pc\_week\_8 | CDAA | X8weeks |
| 528\_51\_CDDA\_Chol\_1pc\_week\_8 | CDAA | X8weeks |
| 528\_52\_CDDA\_Chol\_1pc\_week\_8 | CDAA | X8weeks |
| 528\_53\_CDDA\_Chol\_1pc\_week\_8 | CDAA | X8weeks |
| 528\_54\_CDDA\_Chol\_1pc\_week\_8 | CDAA | X8weeks |
| 528\_55\_CDDA\_Chol\_1pc\_week\_8 | CDAA | X8weeks |
| 528\_56\_CDDA\_Chol\_1pc\_week\_8 | CDAA | X8weeks |
| 528\_65\_CSAA\_week\_12 | CSAA | X12weeks |
| 528\_66\_CSAA\_week\_12 | CSAA | X12weeks |
| 528\_67\_CSAA\_week\_12 | CSAA | X12weeks |
| 528\_68\_CSAA\_week\_12 | CSAA | X12weeks |
| 528\_69\_CSAA\_week\_12 | CSAA | X12weeks |
| 528\_70\_CSAA\_week\_12 | CSAA | X12weeks |
| 528\_71\_CSAA\_week\_12 | CSAA | X12weeks |
| 528\_72\_CSAA\_week\_12 | CSAA | X12weeks |
| 528\_81\_CDDA\_Chol\_1pc\_week\_12 | CDAA | X12weeks |
| 528\_82\_CDDA\_Chol\_1pc\_week\_12 | CDAA | X12weeks |
| 528\_83\_CDDA\_Chol\_1pc\_week\_12 | CDAA | X12weeks |
| 528\_84\_CDDA\_Chol\_1pc\_week\_12 | CDAA | X12weeks |
| 528\_85\_CDDA\_Chol\_1pc\_week\_12 | CDAA | X12weeks |
| 528\_86\_CDDA\_Chol\_1pc\_week\_12 | CDAA | X12weeks |
| 528\_87\_CDDA\_Chol\_1pc\_week\_12 | CDAA | X12weeks |
| 528\_88\_CDDA\_Chol\_1pc\_week\_12 | CDAA | X12weeks |

### Citations

Please cite the following paper for this tool:
  
Liu R, Holik AZ, Su S, Jansz N, Chen K, Leong HS, Blewitt ME, Asselin-Labat ML, Smyth GK, Ritchie ME (2015). Why weight? Modelling sample and observational level variability improves power in RNA-seq analyses. Nucleic Acids Research, 43(15), e97.

#### limma

Please cite the paper below for the limma software itself. Please also try to cite the appropriate methodology articles that describe the statistical methods implemented in limma, depending on which limma functions you are using. The methodology articles are listed in Section 2.1 of the limma User's Guide. Cite no. 3 only if sample weights were used.

1. Smyth GK (2005). Limma: linear models for microarray data. In: 'Bioinformatics and Computational Biology Solutions using R and Bioconductor'. R. Gentleman, V. Carey, S. doit,. Irizarry, W. Huber (eds), Springer, New York, pages 397-420.
2. Law CW, Chen Y, Shi W, and Smyth GK (2014). Voom: precision weights unlock linear model analysis tools for RNA-seq read counts. Genome Biology 15, R29.
3. Ritchie ME, Diyagama D, Neilson J, van Laar R, Dobrovic A, Holloway A and Smyth GK (2006). Empirical array quality weights for microarray data. BMC Bioinformatics 7, Article 261.

#### edgeR

Please cite the first paper for the software itself and the other papers for the various original statistical methods implemented in edgeR. See Section 1.2 in the edgeR User's Guide for more detail.

1. Robinson MD, McCarthy DJ and Smyth GK (2010). edgeR: a Bioconductor package for differential expression analysis of digital gene expression data. Bioinformatics 26, 139-140
2. Robinson MD and Smyth GK (2007). Moderated statistical tests for assessing differences in tag abundance. Bioinformatics 23, 2881-2887
3. Robinson MD and Smyth GK (2008). Small-sample estimation of negative binomial dispersion, with applications to SAGE data. Biostatistics, 9, 321-332
4. McCarthy DJ, Chen Y and Smyth GK (2012). Differential expression analysis of multifactor RNA-Seq experiments with respect to biological variation. Nucleic Acids Research 40, 4288-4297

Please report problems or suggestions to: su.s@wehi.edu.au

Session Info
