# Supplementary material for: GIANT: galaxy-based tool for interactive analysis of transcriptomic data
Source: Sci Rep. 2020 Nov 16;10:19835. doi: 10.1038/s41598-020-76769-w (PMC7670435; doi:10.1038/s41598-020-76769-w)
Supplement: Supplementary file 2 — Supplementary Information. [file 41598_2020_76769_MOESM2_ESM.zip › Tools_InputOutput_Parameters/RNA-seq_workflow/Step1-Normalization_Differential/Outputs/extra_files_path_5/cpmplots.pdf]

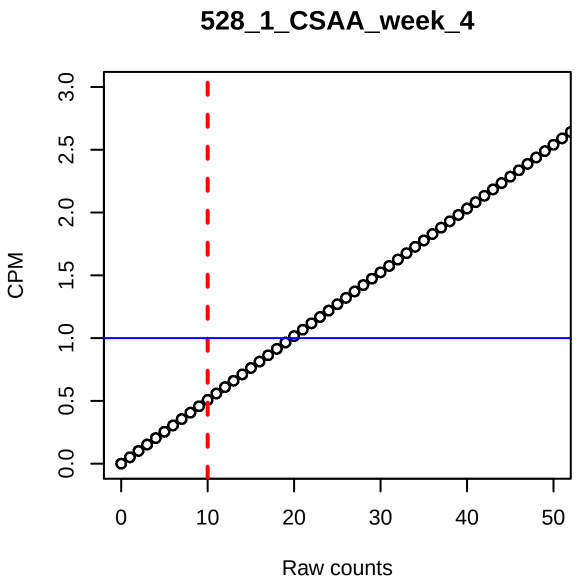
528_1_CSAA_week_4
CPM
0.0
0.5
1.0
1.5
2.0
2.5
3.0
0
10
20
30
40
50
Raw counts

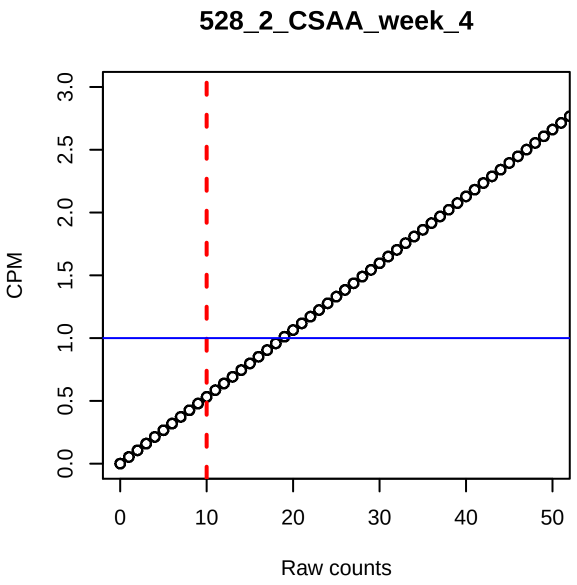
528_2_CSAA_week_4
CPM
0.0
0.5
1.0
1.5
2.0
2.5
3.0
0
10
20
30
40
50
Raw counts

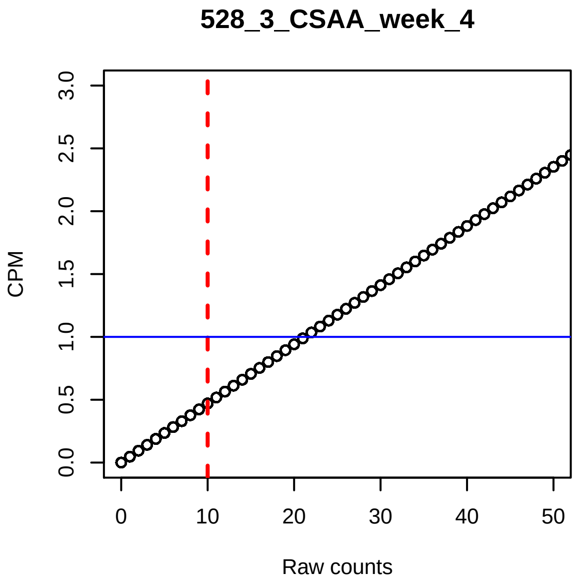
528_3_CSAA_week_4
CPM
0.0
0.5
1.0
1.5
2.0
2.5
3.0
0
10
20
30
40
50
Raw counts

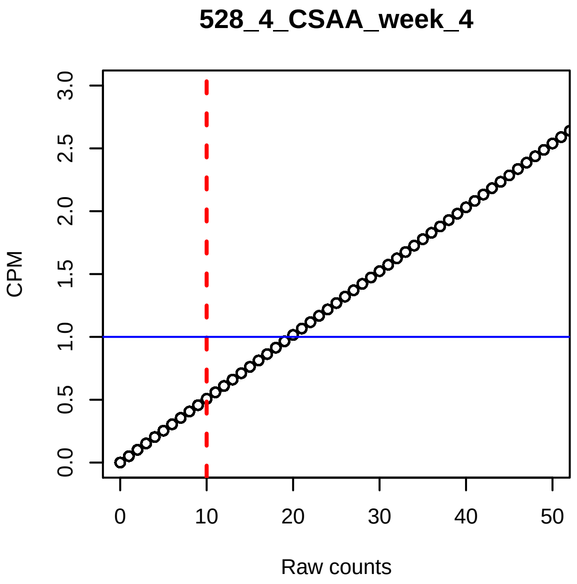
528_4_CSAA_week_4
CPM
0.0
0.5
1.0
1.5
2.0
2.5
3.0
0
10
20
30
40
50
Raw counts

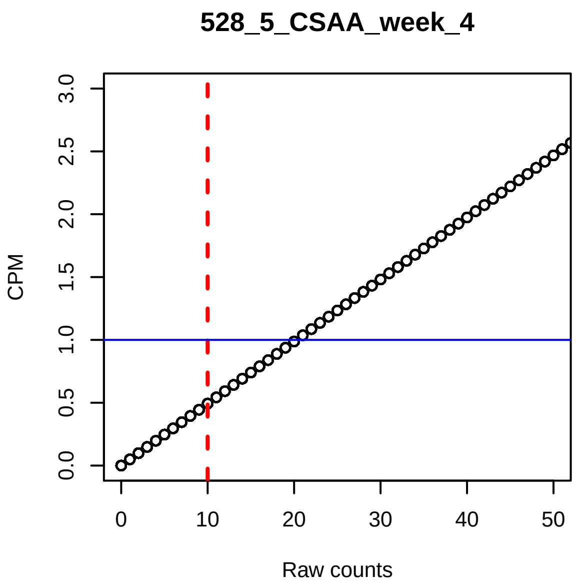
528_5_CSAA_week_4
CPM
0.0
0.5
1.0
1.5
2.0
2.5
3.0
0
10
20
30
40
50
Raw counts

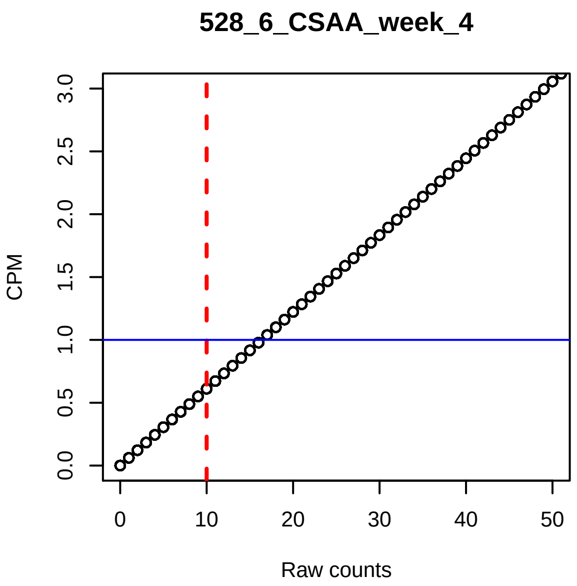
528_6_CSAA_week_4
CPM
0.0
0.5
1.0
1.5
2.0
2.5
3.0
0
10
20
30
40
50
Raw counts

528_7_CSAA_week_4

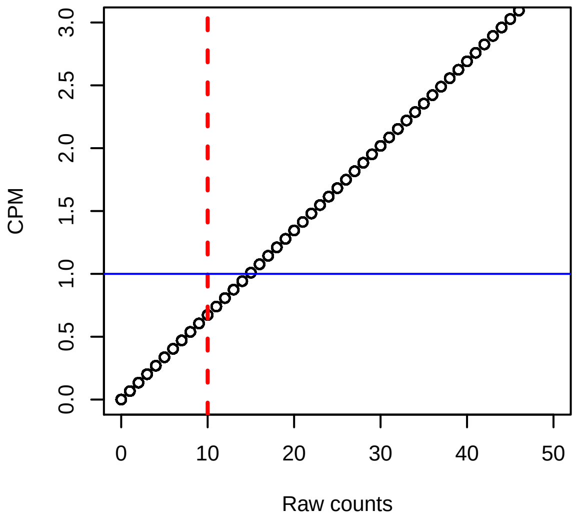

528_8_CSAA_week_4

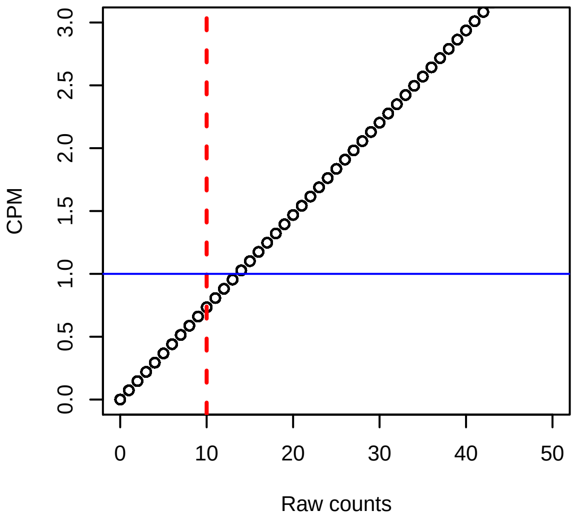

528_17_CDDA_Chol_1pc_week_4

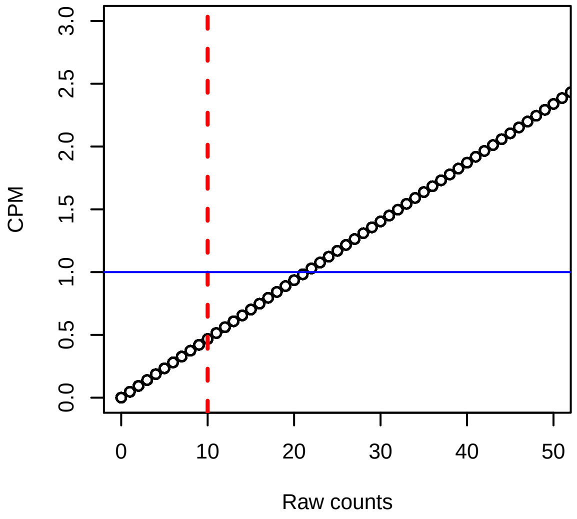

528_18_CDDA_Chol_1pc_week_4

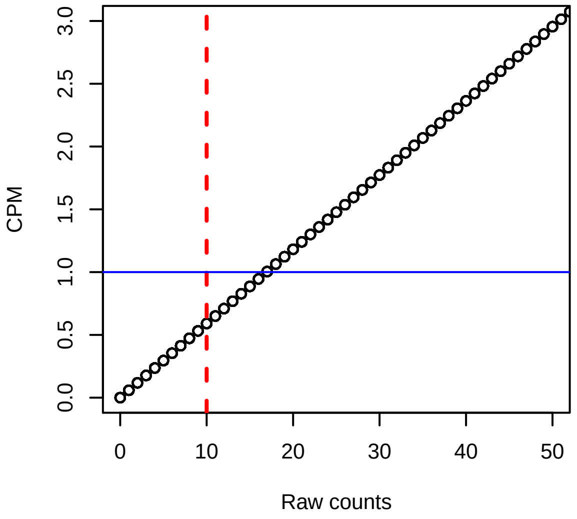

528_19_CDDA_Chol_1pc_week_4

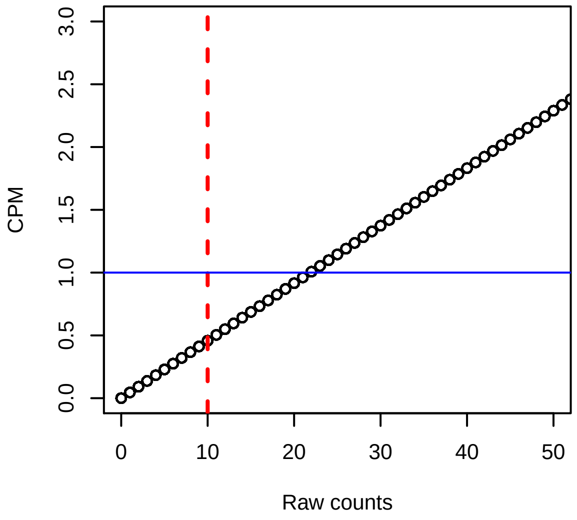

528_20_CDDA_Chol_1pc_week_4

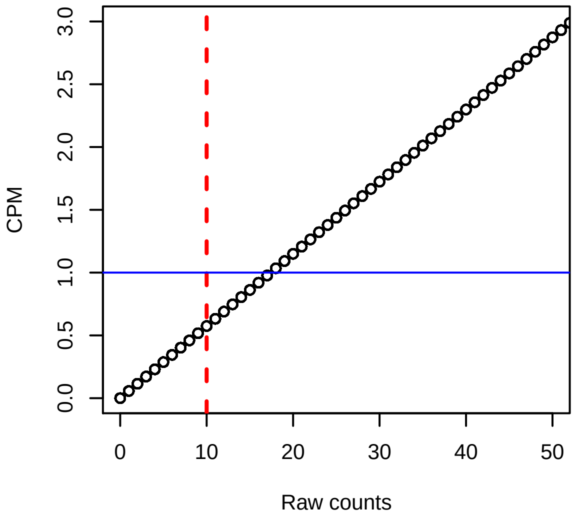

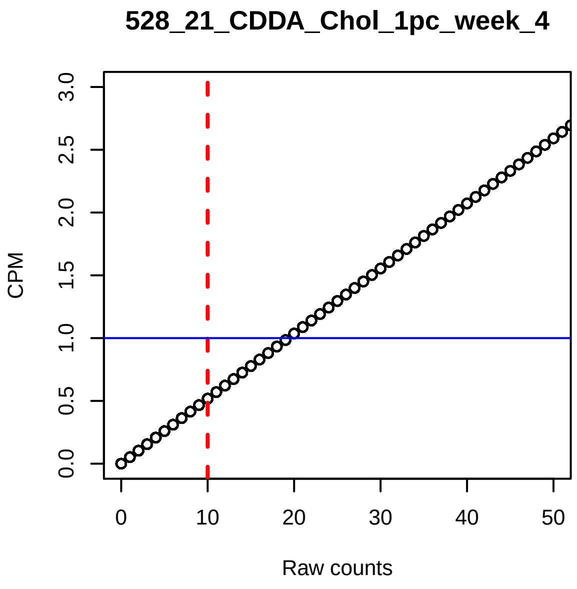
528_21_CDDA_Chol_1pc_week_4
CPM
0.0 0.5 1.0 1.5 2.0 2.5 3.0
0 10 20 30 40 50
Raw counts

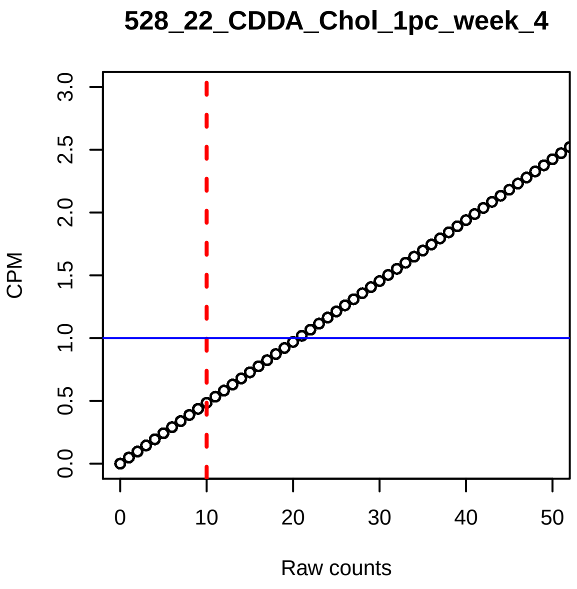
528_22_CDDA_Chol_1pc_week_4
CPM
0.0 0.5 1.0 1.5 2.0 2.5 3.0
0 10 20 30 40 50
Raw counts

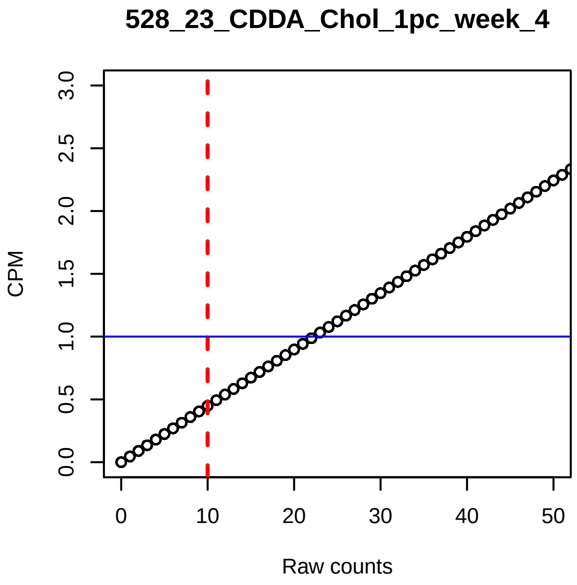
528_23_CDDA_Chol_1pc_week_4
CPM
0.0 0.5 1.0 1.5 2.0 2.5 3.0
0 10 20 30 40 50
Raw counts

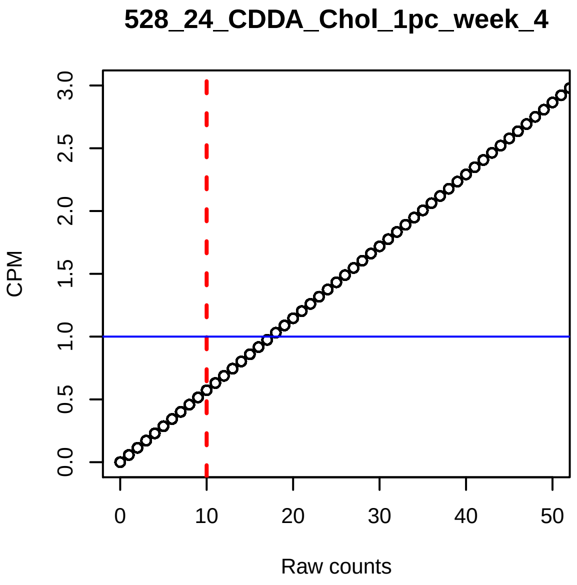
528_24_CDDA_Chol_1pc_week_4
CPM
0.0 0.5 1.0 1.5 2.0 2.5 3.0
0 10 20 30 40 50
Raw counts

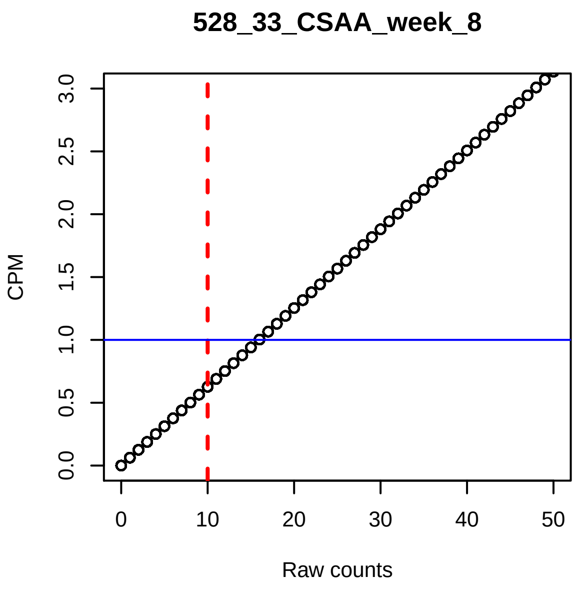
528_33_CSAA_week_8
CPM
0.0 0.5 1.0 1.5 2.0 2.5 3.0
0 10 20 30 40 50
Raw counts

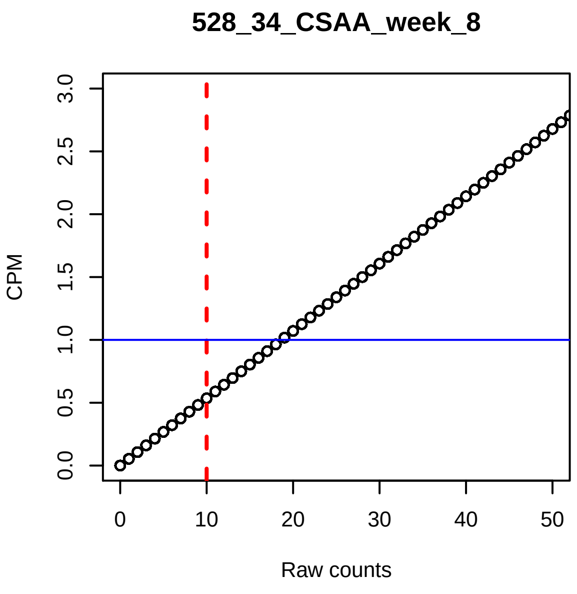
528_34_CSAA_week_8
CPM
0.0 0.5 1.0 1.5 2.0 2.5 3.0
0 10 20 30 40 50
Raw counts

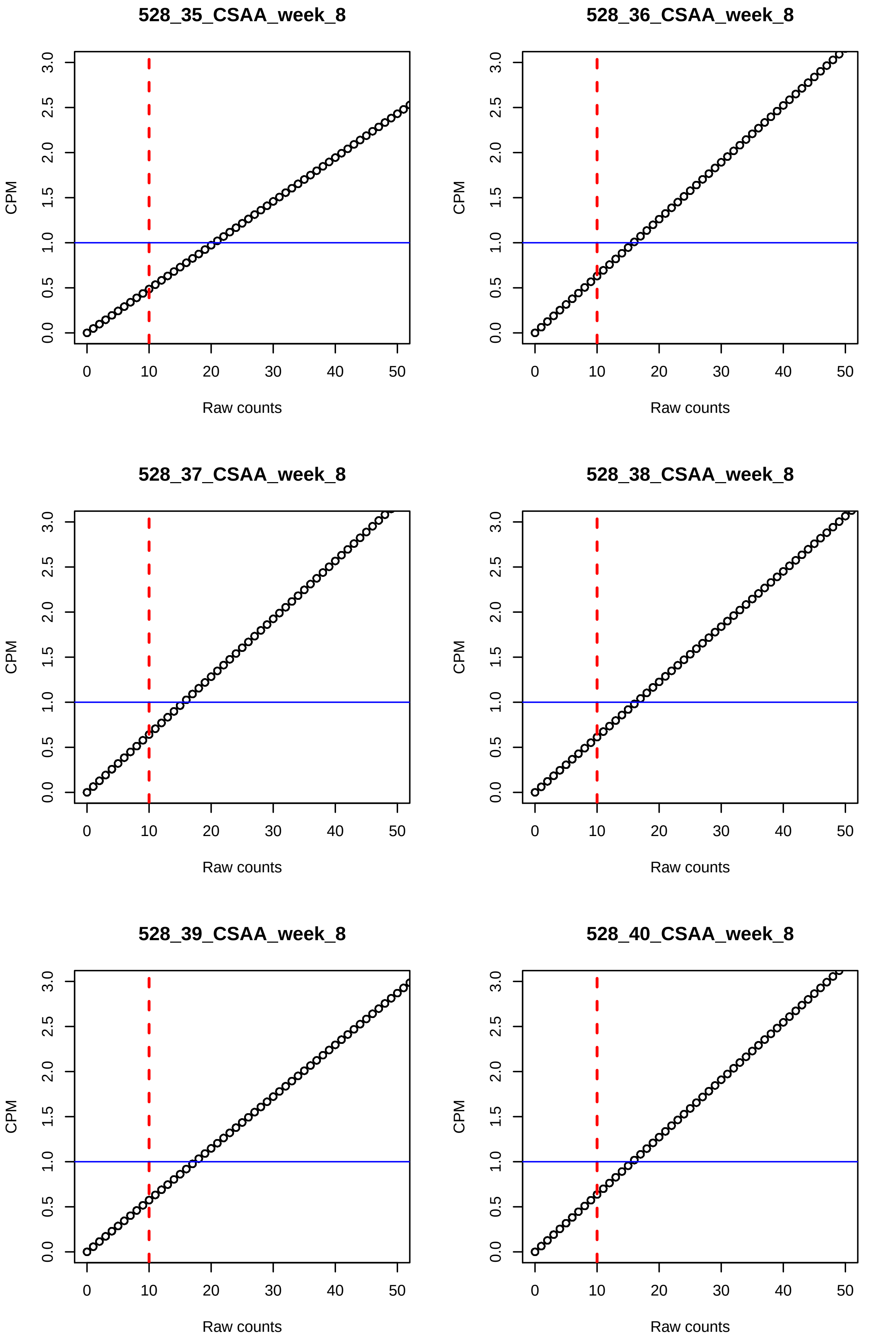
528_35_CSAA_week_8
528_36_CSAA_week_8
528_37_CSAA_week_8
528_38_CSAA_week_8
528_39_CSAA_week_8
528_40_CSAA_week_8
CPM
Raw counts
0.0 0.5 1.0 1.5 2.0 2.5 3.0
0 10 20 30 40 50

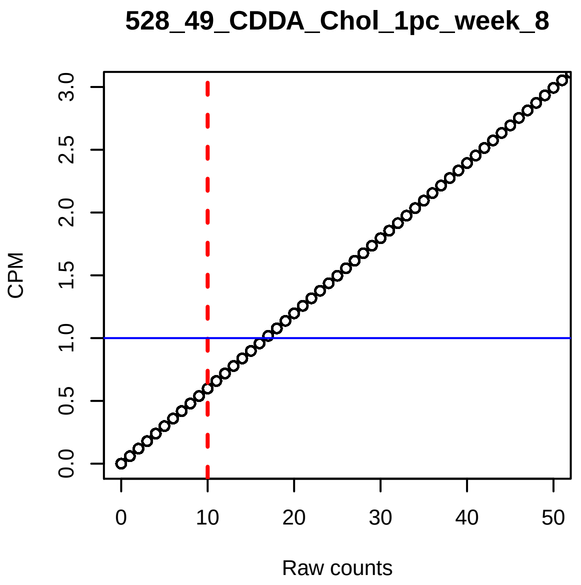
528_49_CDDA_Chol_1pc_week_8
CPM
0.0 0.5 1.0 1.5 2.0 2.5 3.0
0 10 20 30 40 50
Raw counts

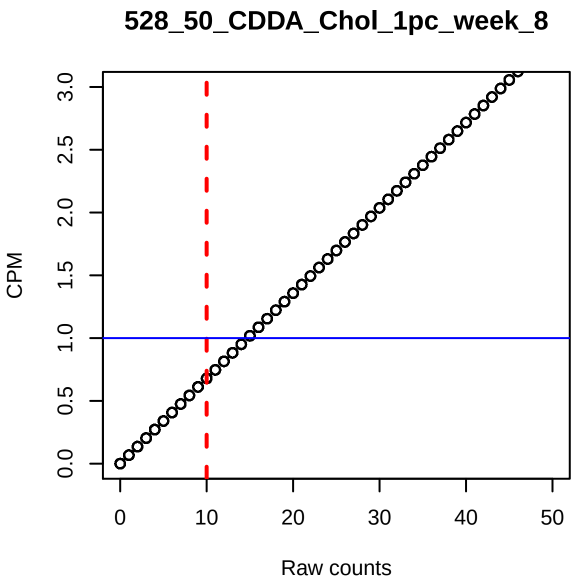
528_50_CDDA_Chol_1pc_week_8
CPM
0.0 0.5 1.0 1.5 2.0 2.5 3.0
0 10 20 30 40 50
Raw counts

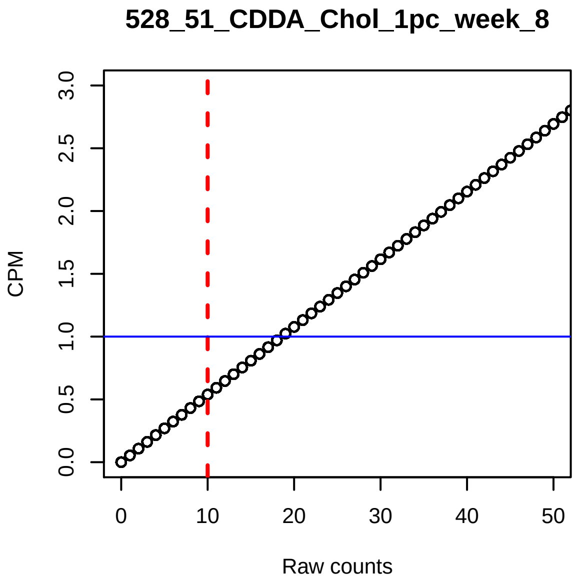
528_51_CDDA_Chol_1pc_week_8
CPM
0.0 0.5 1.0 1.5 2.0 2.5 3.0
0 10 20 30 40 50
Raw counts

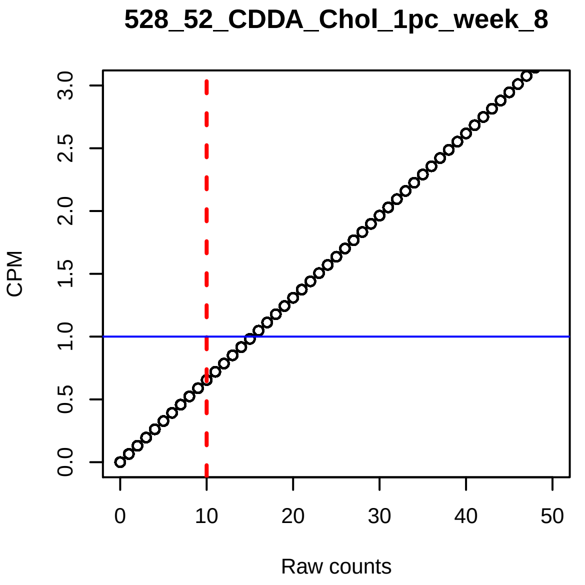
528_52_CDDA_Chol_1pc_week_8
CPM
0.0 0.5 1.0 1.5 2.0 2.5 3.0
0 10 20 30 40 50
Raw counts

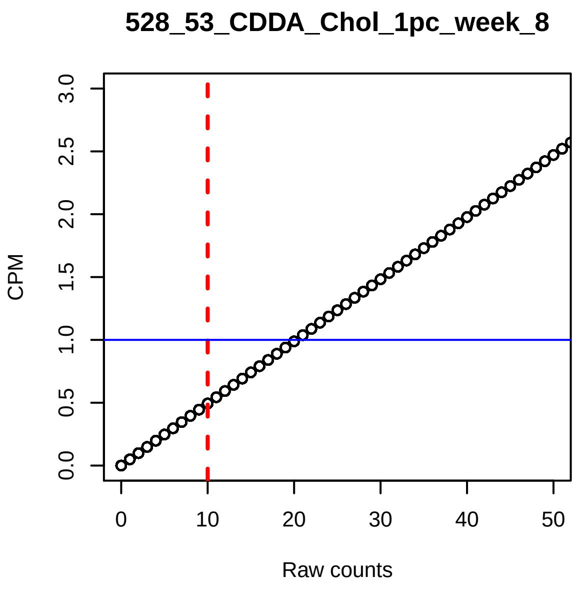
528_53_CDDA_Chol_1pc_week_8
CPM
0.0 0.5 1.0 1.5 2.0 2.5 3.0
0 10 20 30 40 50
Raw counts

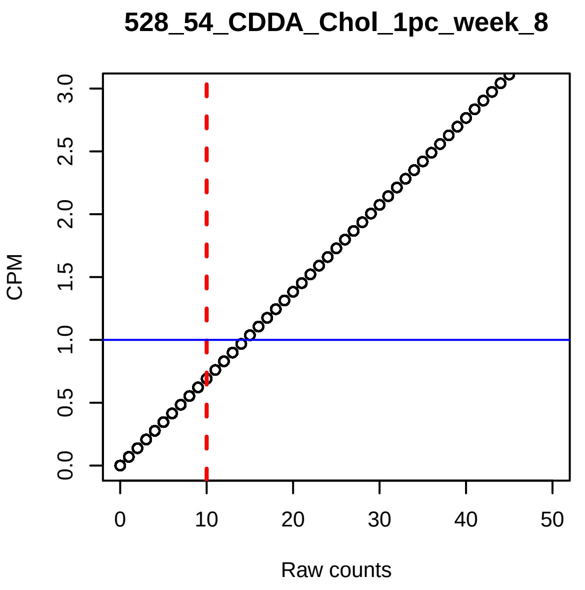
528_54_CDDA_Chol_1pc_week_8
CPM
0.0 0.5 1.0 1.5 2.0 2.5 3.0
0 10 20 30 40 50
Raw counts

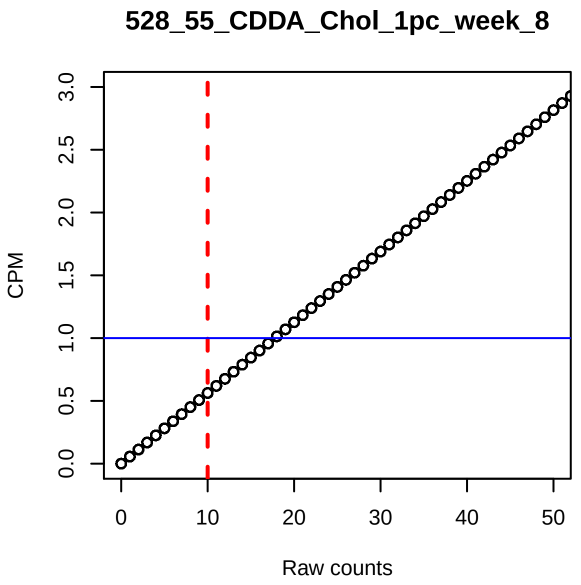
528_55_CDDA_Chol_1pc_week_8
CPM
Raw counts
0.0 0.5 1.0 1.5 2.0 2.5 3.0
0 10 20 30 40 50

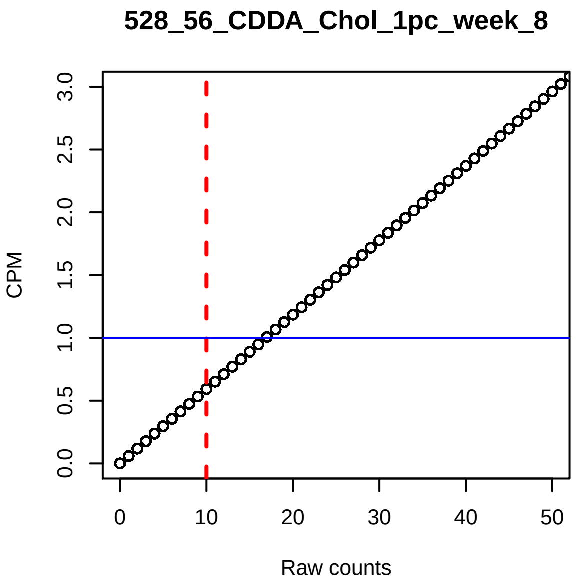
528_56_CDDA_Chol_1pc_week_8
CPM
Raw counts
0.0 0.5 1.0 1.5 2.0 2.5 3.0
0 10 20 30 40 50

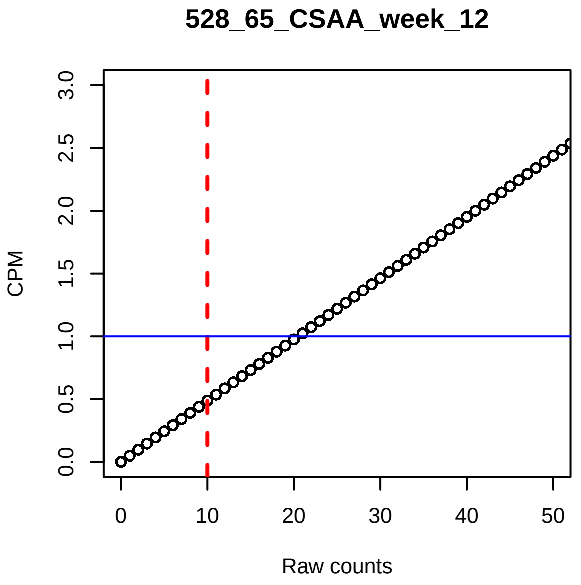
528_65_CSAA_week_12
CPM
Raw counts
0.0 0.5 1.0 1.5 2.0 2.5 3.0
0 10 20 30 40 50

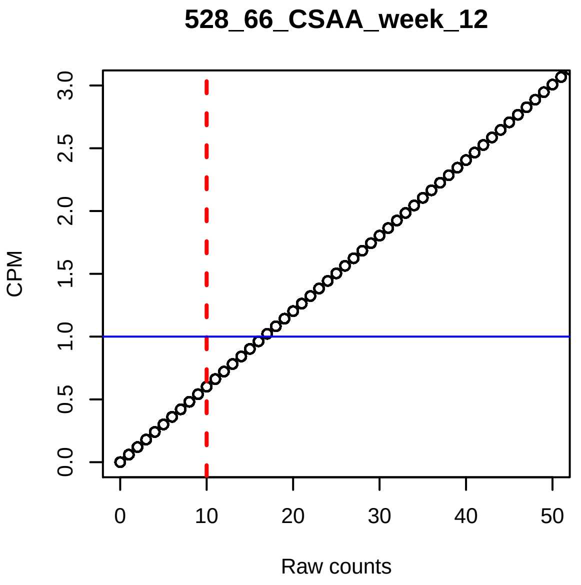
528_66_CSAA_week_12
CPM
Raw counts
0.0 0.5 1.0 1.5 2.0 2.5 3.0
0 10 20 30 40 50

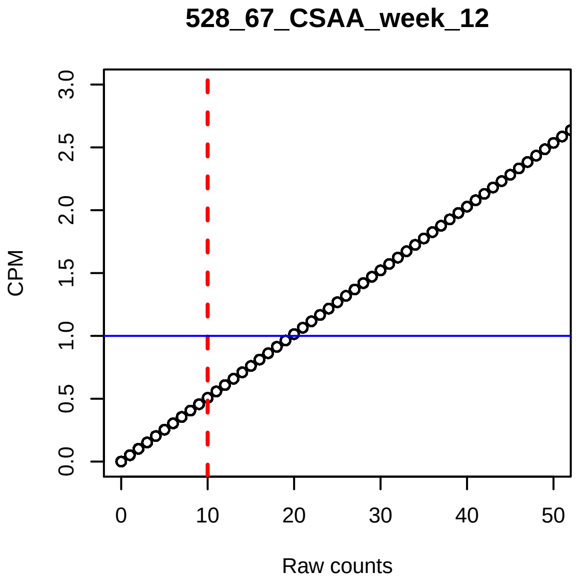
528_67_CSAA_week_12
CPM
Raw counts
0.0 0.5 1.0 1.5 2.0 2.5 3.0
0 10 20 30 40 50

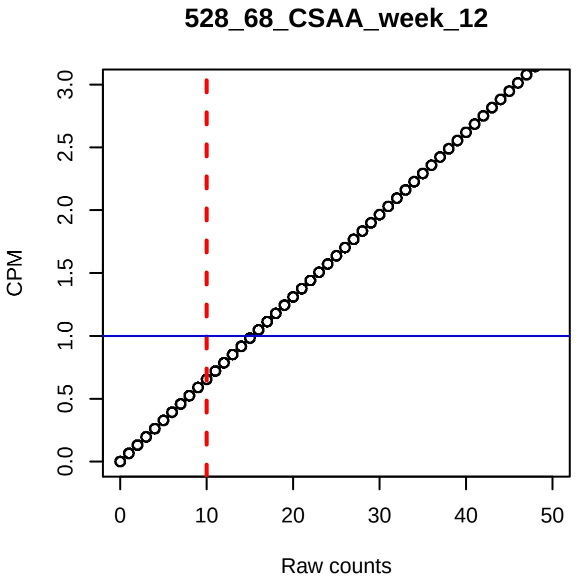
528_68_CSAA_week_12
CPM
Raw counts
0.0 0.5 1.0 1.5 2.0 2.5 3.0
0 10 20 30 40 50

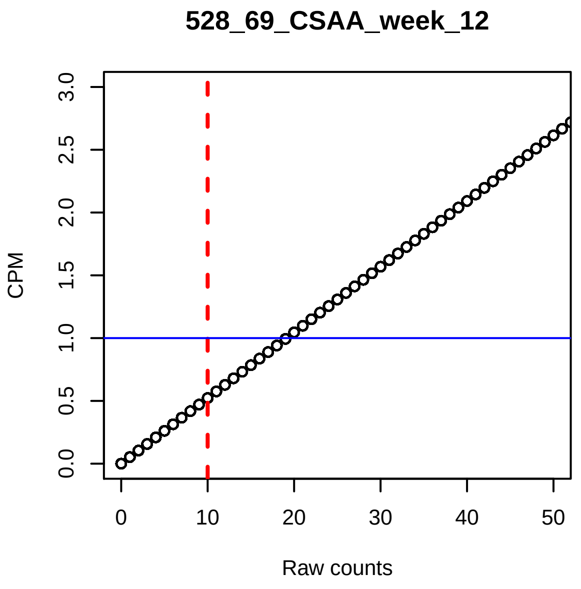
528_69_CSAA_week_12
CPM
0.0 0.5 1.0 1.5 2.0 2.5 3.0
0 10 20 30 40 50
Raw counts

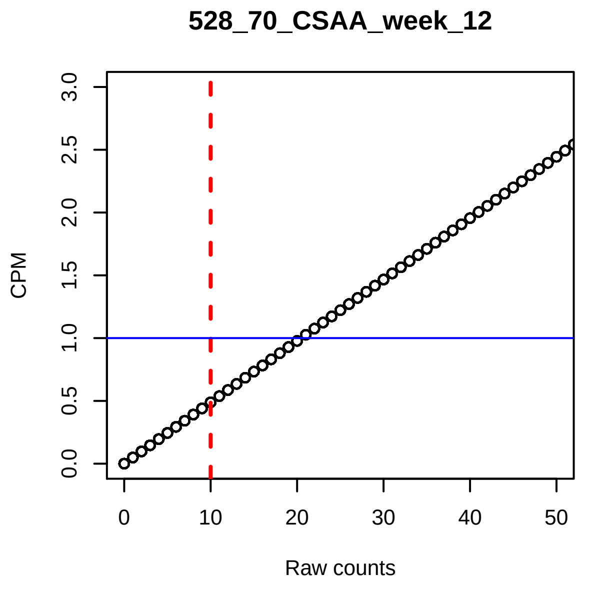
528_70_CSAA_week_12
CPM
0.0 0.5 1.0 1.5 2.0 2.5 3.0
0 10 20 30 40 50
Raw counts

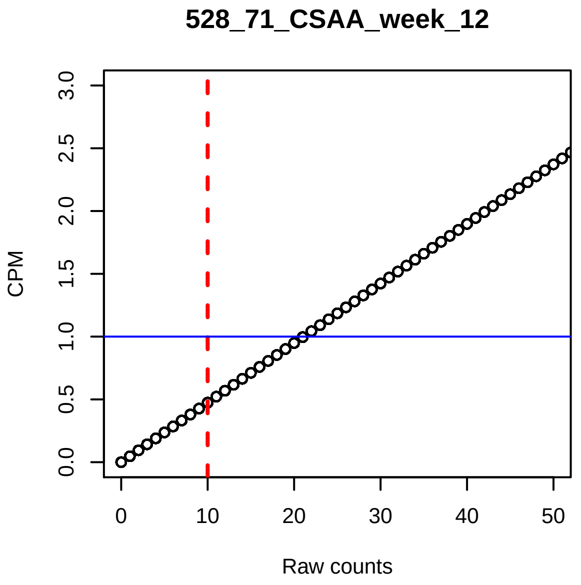
528_71_CSAA_week_12
CPM
0.0 0.5 1.0 1.5 2.0 2.5 3.0
0 10 20 30 40 50
Raw counts

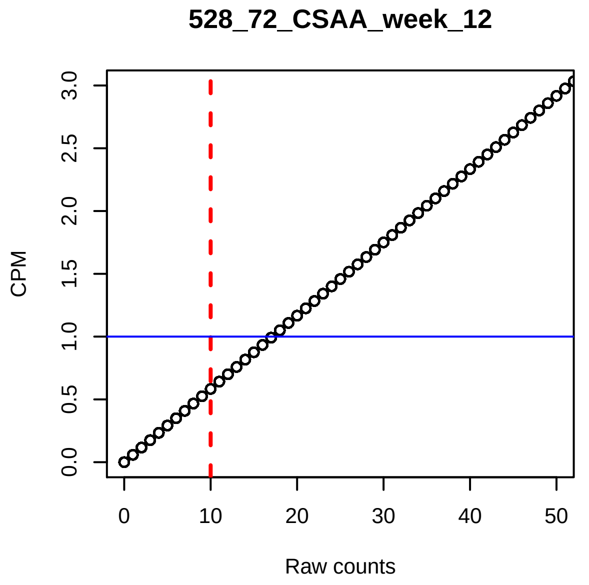
528_72_CSAA_week_12
CPM
0.0 0.5 1.0 1.5 2.0 2.5 3.0
0 10 20 30 40 50
Raw counts

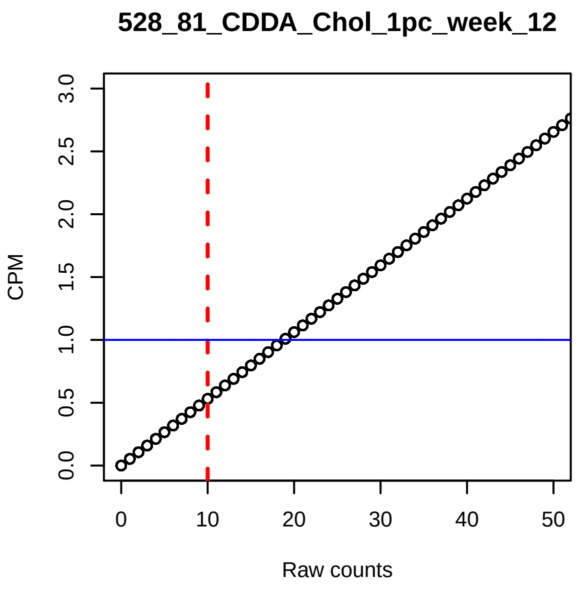
528_81_CDDA_Chol_1pc_week_12
CPM
0.0 0.5 1.0 1.5 2.0 2.5 3.0
0 10 20 30 40 50
Raw counts

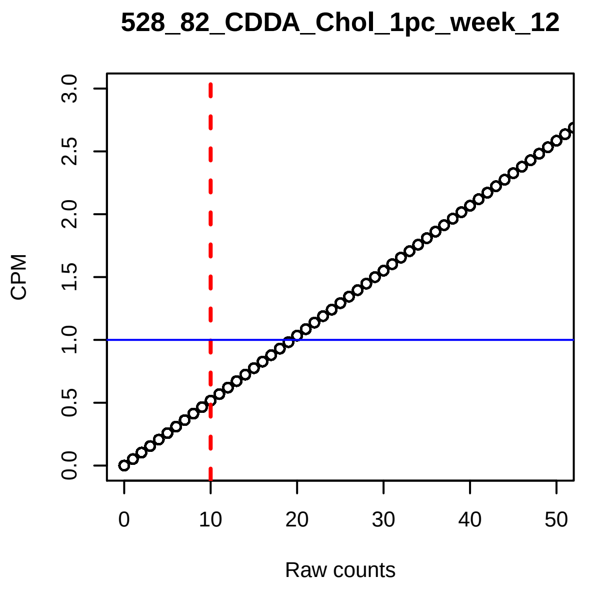
528_82_CDDA_Chol_1pc_week_12
CPM
0.0 0.5 1.0 1.5 2.0 2.5 3.0
0 10 20 30 40 50
Raw counts

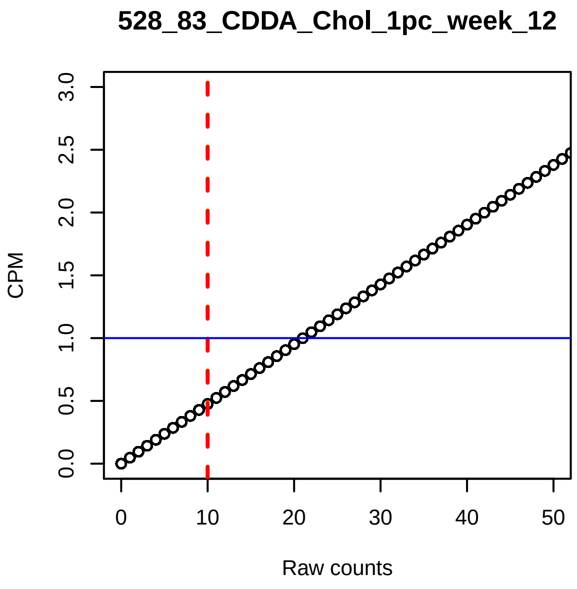
528_83_CDDA_Chol_1pc_week_12
CPM
Raw counts
0.0
0.5
1.0
1.5
2.0
2.5
3.0
0
10
20
30
40
50

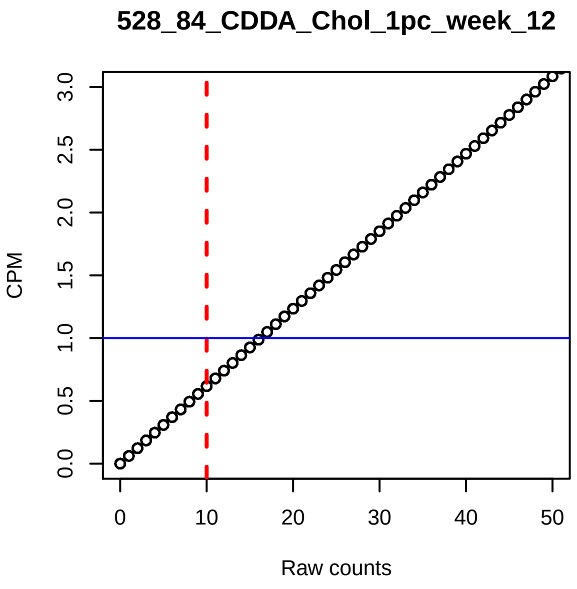
528_84_CDDA_Chol_1pc_week_12
CPM
Raw counts
0.0
0.5
1.0
1.5
2.0
2.5
3.0
0
10
20
30
40
50

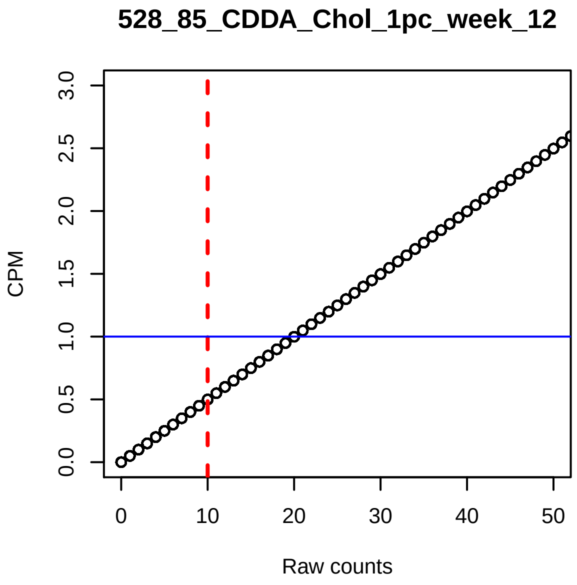
528_85_CDDA_Chol_1pc_week_12
CPM
Raw counts
0.0
0.5
1.0
1.5
2.0
2.5
3.0
0
10
20
30
40
50

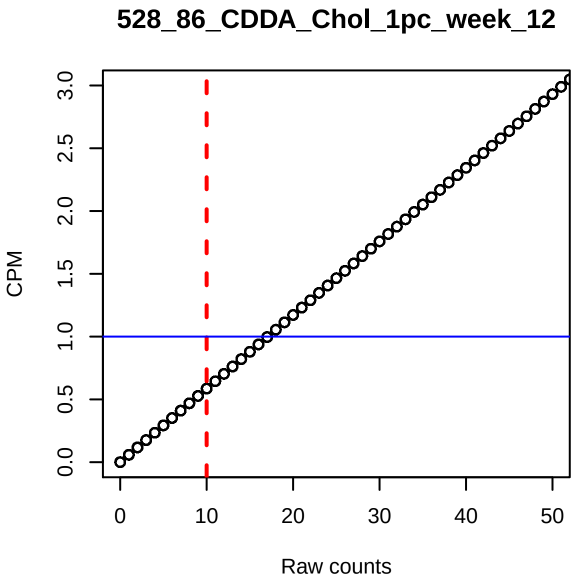
528_86_CDDA_Chol_1pc_week_12
CPM
Raw counts
0.0
0.5
1.0
1.5
2.0
2.5
3.0
0
10
20
30
40
50

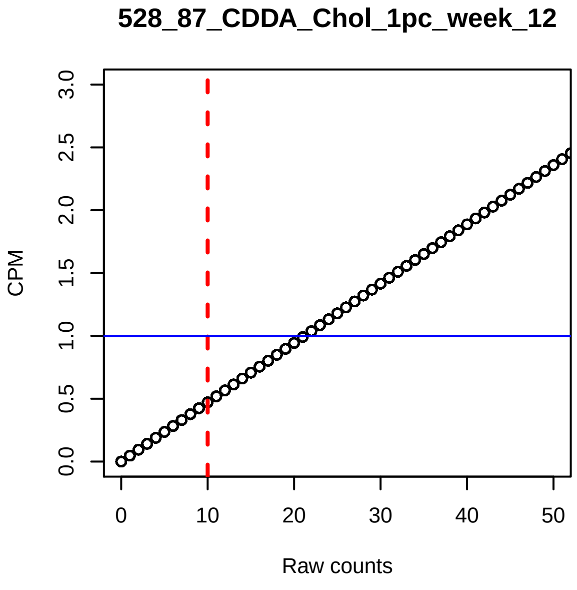
528_87_CDDA_Chol_1pc_week_12
CPM
Raw counts
0.0
0.5
1.0
1.5
2.0
2.5
3.0
0
10
20
30
40
50

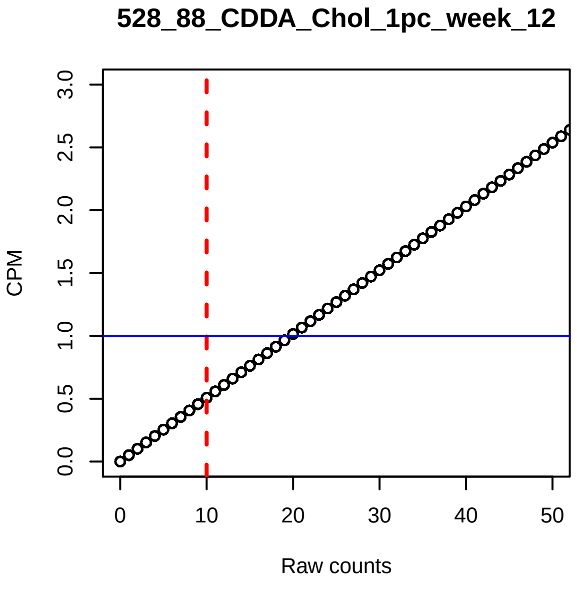
528_88_CDDA_Chol_1pc_week_12
CPM
Raw counts
0.0
0.5
1.0
1.5
2.0
2.5
3.0
0
10
20
30
40
50
