# Supplementary material for: GIANT: galaxy-based tool for interactive analysis of transcriptomic data
Source: Sci Rep. 2020 Nov 16;10:19835. doi: 10.1038/s41598-020-76769-w (PMC7670435; doi:10.1038/s41598-020-76769-w)
Supplement: Supplementary file 2 — Supplementary Information. [file 41598_2020_76769_MOESM2_ESM.zip › Tools_InputOutput_Parameters/RNA-seq_workflow/Step1-Normalization_Differential/Outputs/extra_files_path_5/heatmap_CDAA-CSAA.pdf]

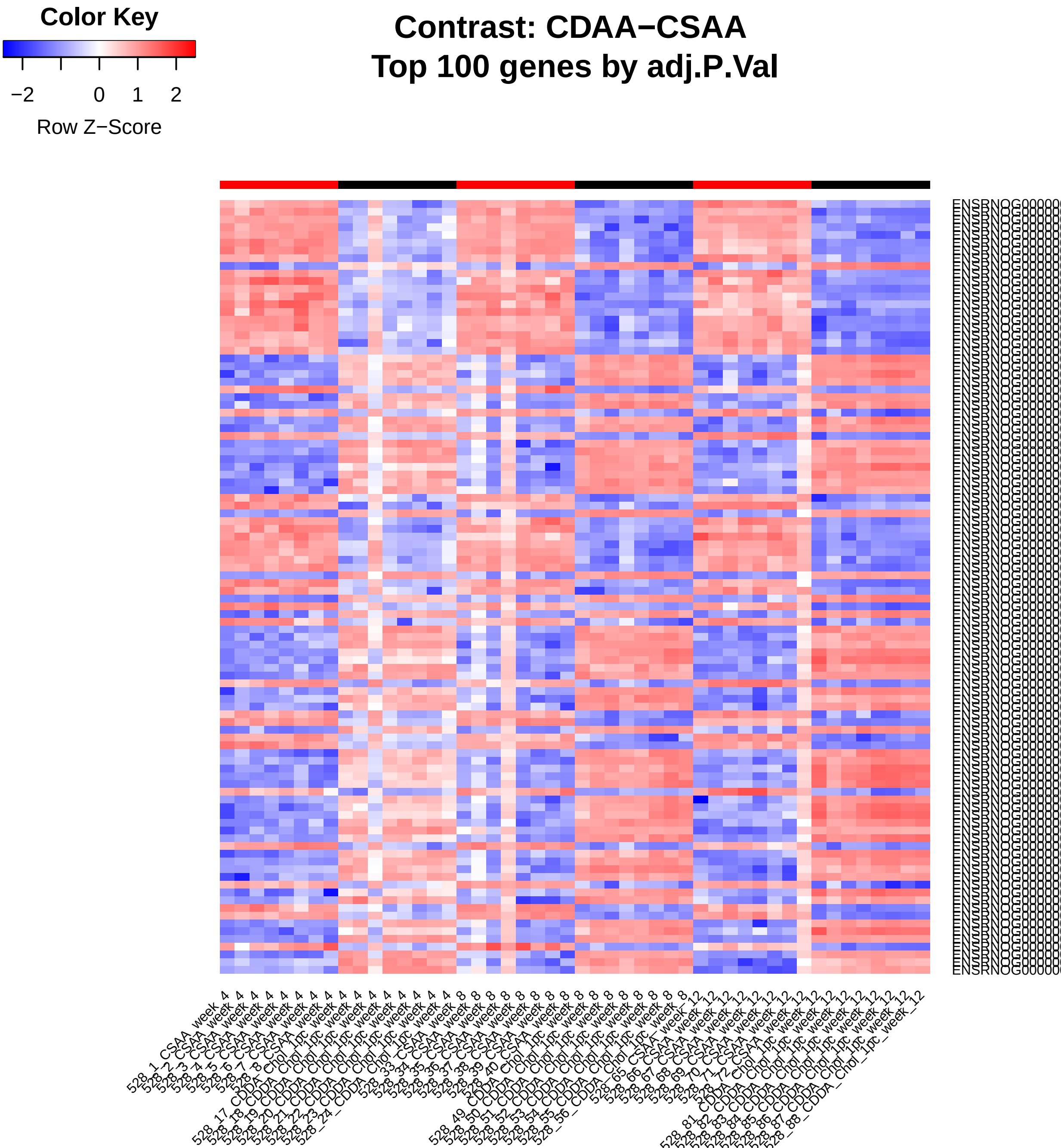
Color Key
−2
0
1
2
Row Z−Score
Contrast: CDAA−CSAA
Top 100 genes by adj.P.Val
