# Supplementary material for: GIANT: galaxy-based tool for interactive analysis of transcriptomic data
Source: Sci Rep. 2020 Nov 16;10:19835. doi: 10.1038/s41598-020-76769-w (PMC7670435; doi:10.1038/s41598-020-76769-w)
Supplement: Supplementary file 2 — Supplementary Information. [file 41598_2020_76769_MOESM2_ESM.zip › Tools_InputOutput_Parameters/RNA-seq_workflow/Step1-Normalization_Differential/Outputs/extra_files_path_5/densityplots.pdf]

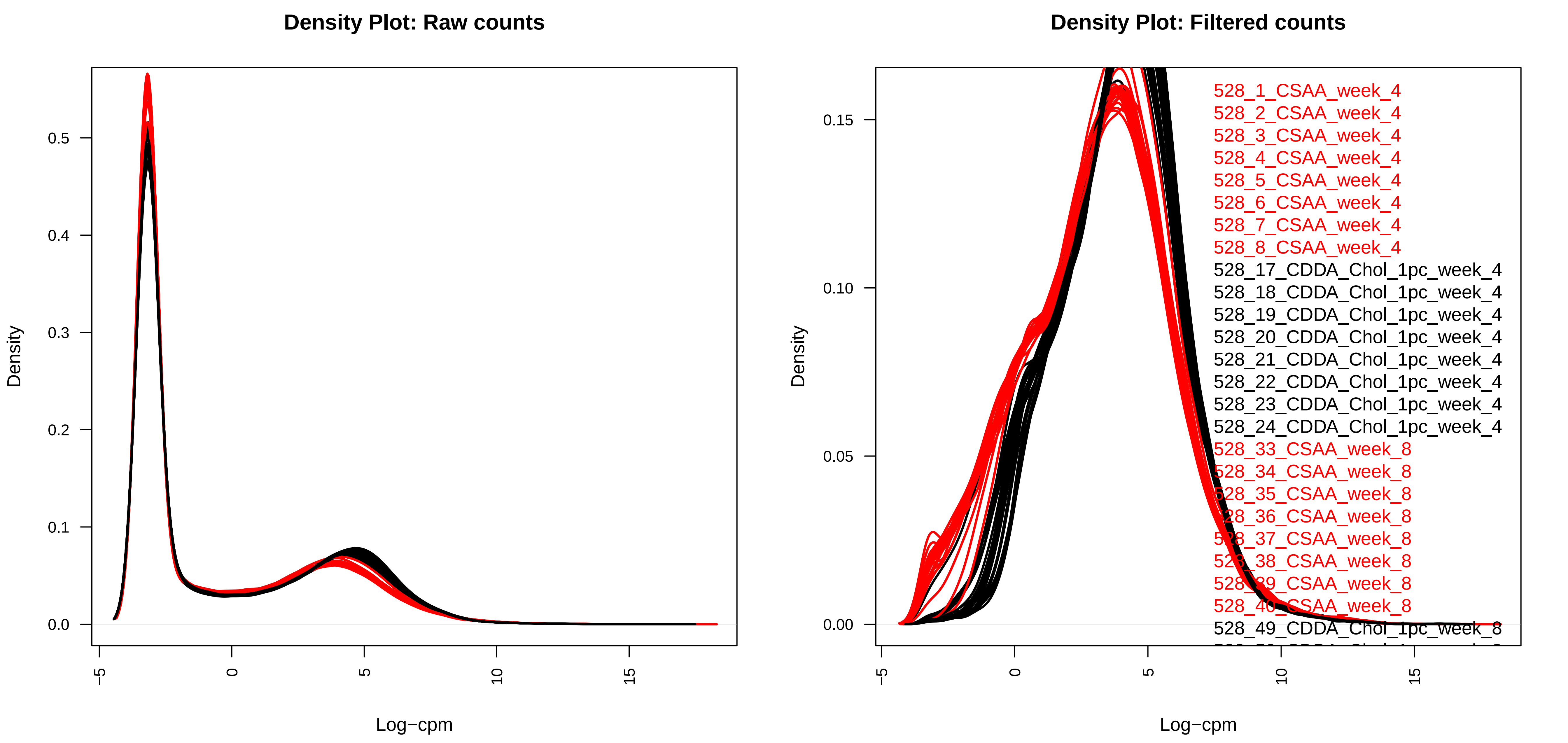
Density Plot: Raw counts
Density
0.0
0.1
0.2
0.3
0.4
0.5
-5
0
5
10
15
Log−cpm
Density Plot: Filtered counts
Density
0.00
0.05
0.10
0.15
-5
0
5
10
15
Log−cpm
528_1_CSAA_week_4
528_2_CSAA_week_4
528_3_CSAA_week_4
528_4_CSAA_week_4
528_5_CSAA_week_4
528_6_CSAA_week_4
528_7_CSAA_week_4
528_8_CSAA_week_4
528_17_CDDA_Chol_1pc_week_4
528_18_CDDA_Chol_1pc_week_4
528_19_CDDA_Chol_1pc_week_4
528_20_CDDA_Chol_1pc_week_4
528_21_CDDA_Chol_1pc_week_4
528_22_CDDA_Chol_1pc_week_4
528_23_CDDA_Chol_1pc_week_4
528_24_CDDA_Chol_1pc_week_4
528_33_CSAA_week_8
528_34_CSAA_week_8
528_35_CSAA_week_8
528_36_CSAA_week_8
528_37_CSAA_week_8
528_38_CSAA_week_8
528_39_CSAA_week_8
528_40_CSAA_week_8
528_49_CDDA_Chol_1pc_week_8
