# Supplementary material for: GIANT: galaxy-based tool for interactive analysis of transcriptomic data
Source: Sci Rep. 2020 Nov 16;10:19835. doi: 10.1038/s41598-020-76769-w (PMC7670435; doi:10.1038/s41598-020-76769-w)
Supplement: Supplementary file 2 — Supplementary Information. [file 41598_2020_76769_MOESM2_ESM.zip › Tools_InputOutput_Parameters/RNA-seq_workflow/Step1-Normalization_Differential/Outputs/extra_files_path_5/stripcharts_CDAA-CSAA.pdf]

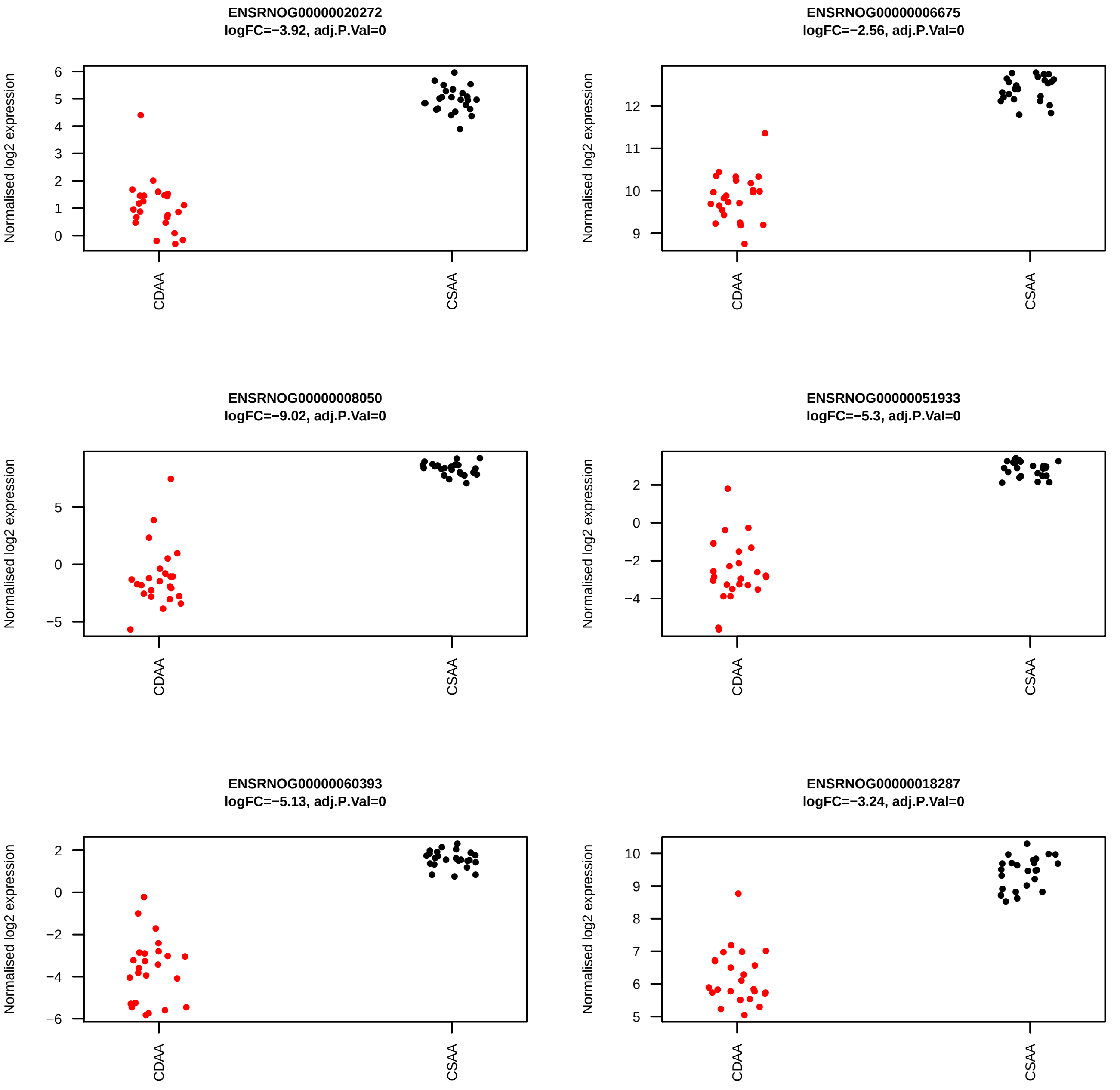
ENSRNOG00000020272
logFC=−3.92, adj.P.Val=0
ENSRNOG00000006675
logFC=−2.56, adj.P.Val=0
ENSRNOG00000008050
logFC=−9.02, adj.P.Val=0
ENSRNOG00000051933
logFC=−5.3, adj.P.Val=0
ENSRNOG00000060393
logFC=−5.13, adj.P.Val=0
ENSRNOG00000018287
logFC=−3.24, adj.P.Val=0
Normalised log2 expression
CDAA
CSAA

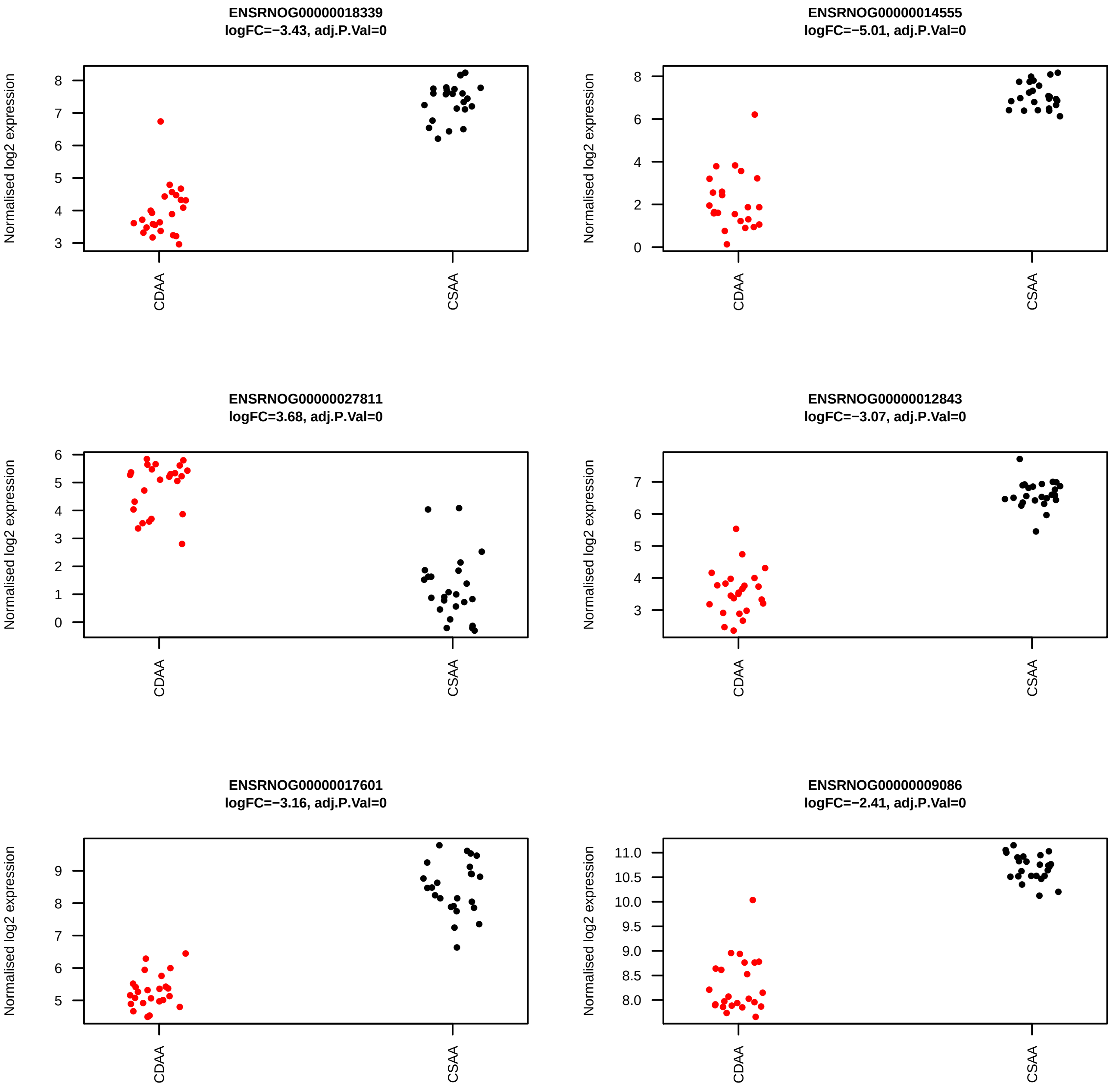
ENSRNOG00000018339
logFC=−3.43, adj.P.Val=0
Normalised log2 expression
CDAA
CSAA
ENSRNOG00000014555
logFC=−5.01, adj.P.Val=0
Normalised log2 expression
CDAA
CSAA
ENSRNOG00000027811
logFC=3.68, adj.P.Val=0
Normalised log2 expression
CDAA
CSAA
ENSRNOG00000012843
logFC=−3.07, adj.P.Val=0
Normalised log2 expression
CDAA
CSAA
ENSRNOG00000017601
logFC=−3.16, adj.P.Val=0
Normalised log2 expression
CDAA
CSAA
ENSRNOG00000009086
logFC=−2.41, adj.P.Val=0
Normalised log2 expression
CDAA
CSAA

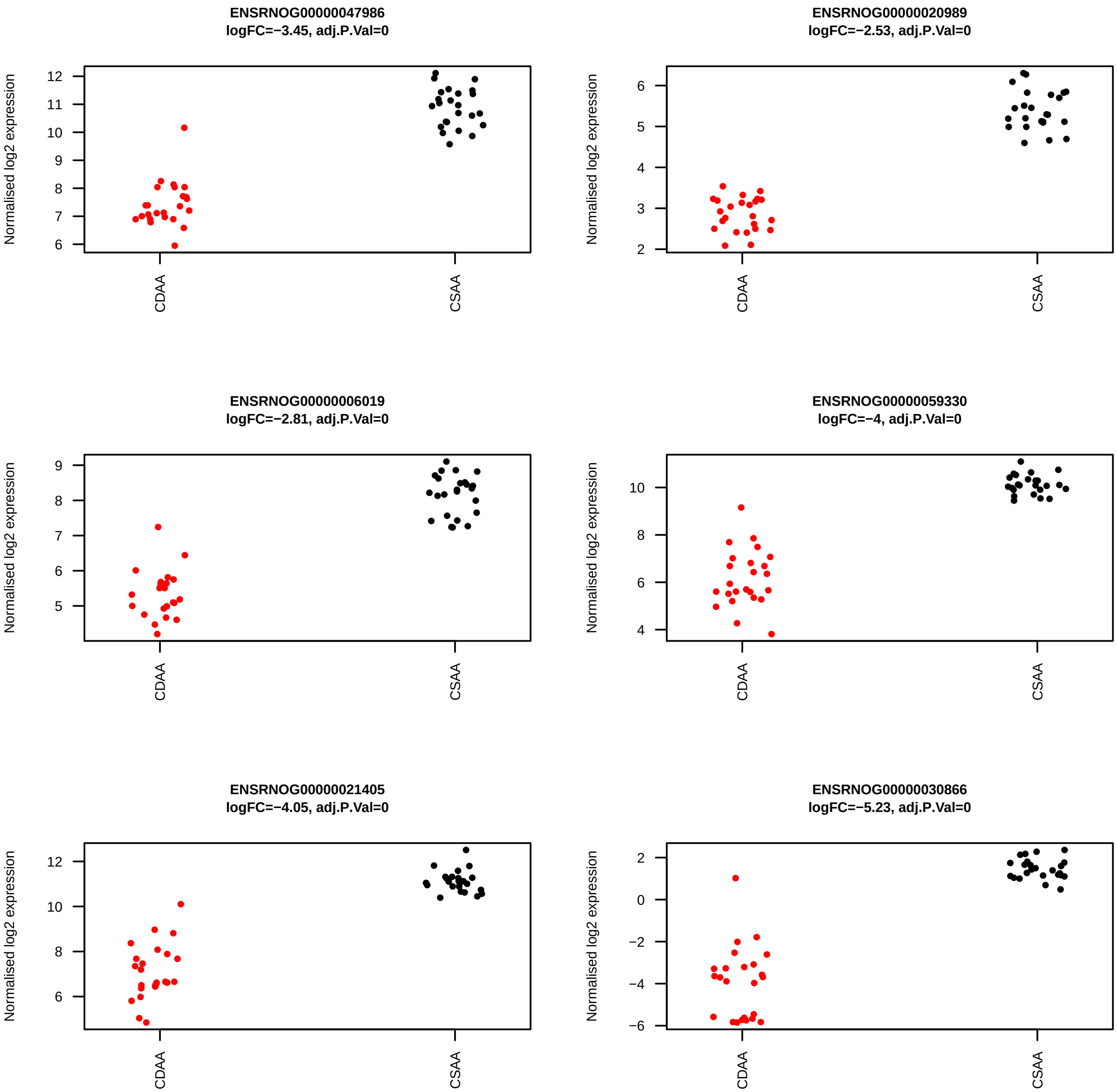
ENSRNOG00000047986
logFC=−3.45, adj.P.Val=0
Normalised log2 expression
12
11
10
9
8
7
6
CDAA
CSAA
ENSRNOG00000020989
logFC=−2.53, adj.P.Val=0
Normalised log2 expression
6
5
4
3
2
CDAA
CSAA
ENSRNOG00000006019
logFC=−2.81, adj.P.Val=0
Normalised log2 expression
9
8
7
6
5
CDAA
CSAA
ENSRNOG00000059330
logFC=−4, adj.P.Val=0
Normalised log2 expression
10
8
6
4
CDAA
CSAA
ENSRNOG00000021405
logFC=−4.05, adj.P.Val=0
Normalised log2 expression
12
10
8
6
CDAA
CSAA
ENSRNOG00000030866
logFC=−5.23, adj.P.Val=0
Normalised log2 expression
2
0
−2
−4
−6
CDAA
CSAA

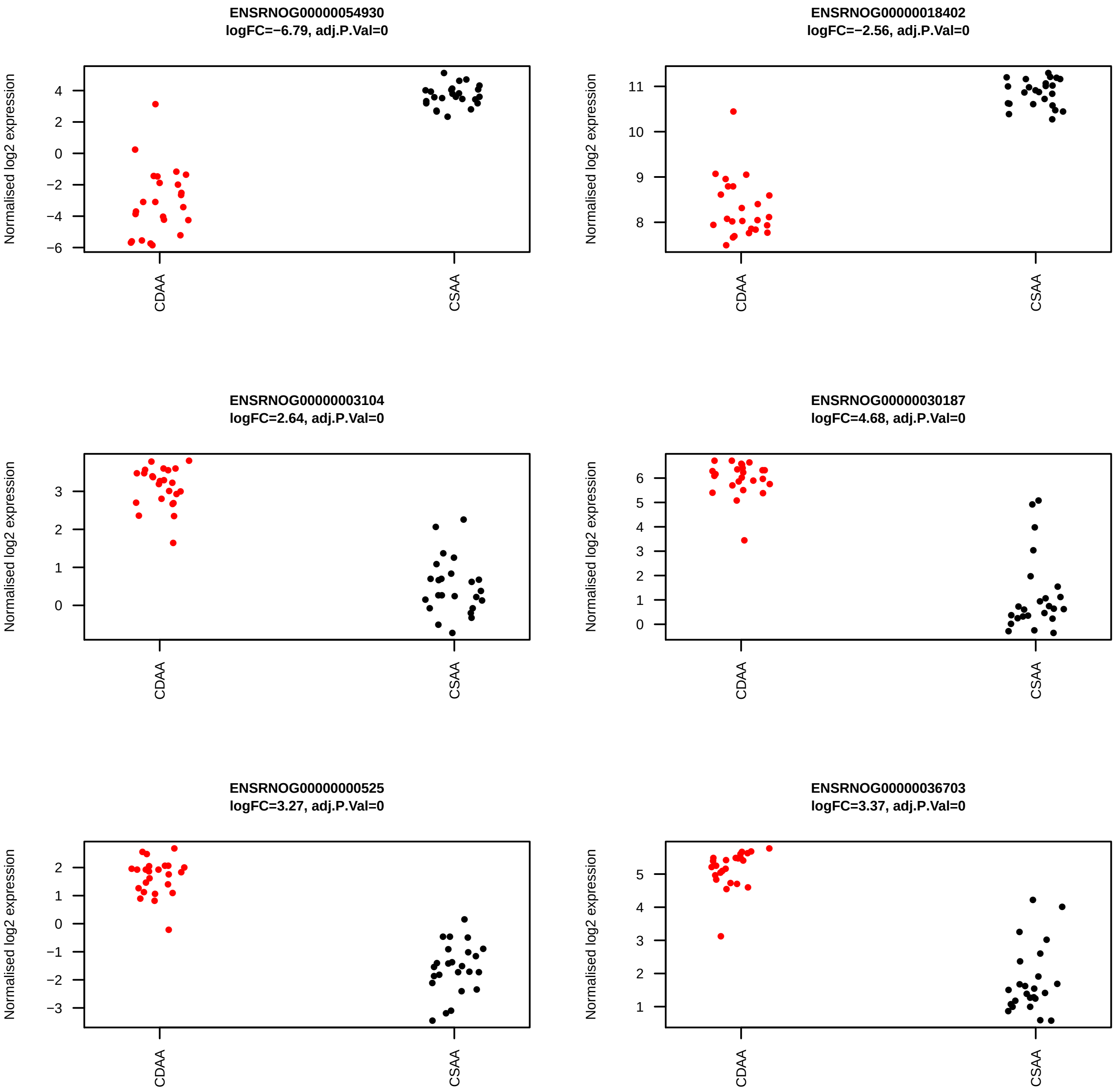
ENSRNOG00000054930
logFC=−6.79, adj.P.Val=0
ENSRNOG00000018402
logFC=−2.56, adj.P.Val=0
ENSRNOG00000003104
logFC=2.64, adj.P.Val=0
ENSRNOG00000030187
logFC=4.68, adj.P.Val=0
ENSRNOG00000000525
logFC=3.27, adj.P.Val=0
ENSRNOG00000036703
logFC=3.37, adj.P.Val=0
Normalised log2 expression
CDAA
CSAA

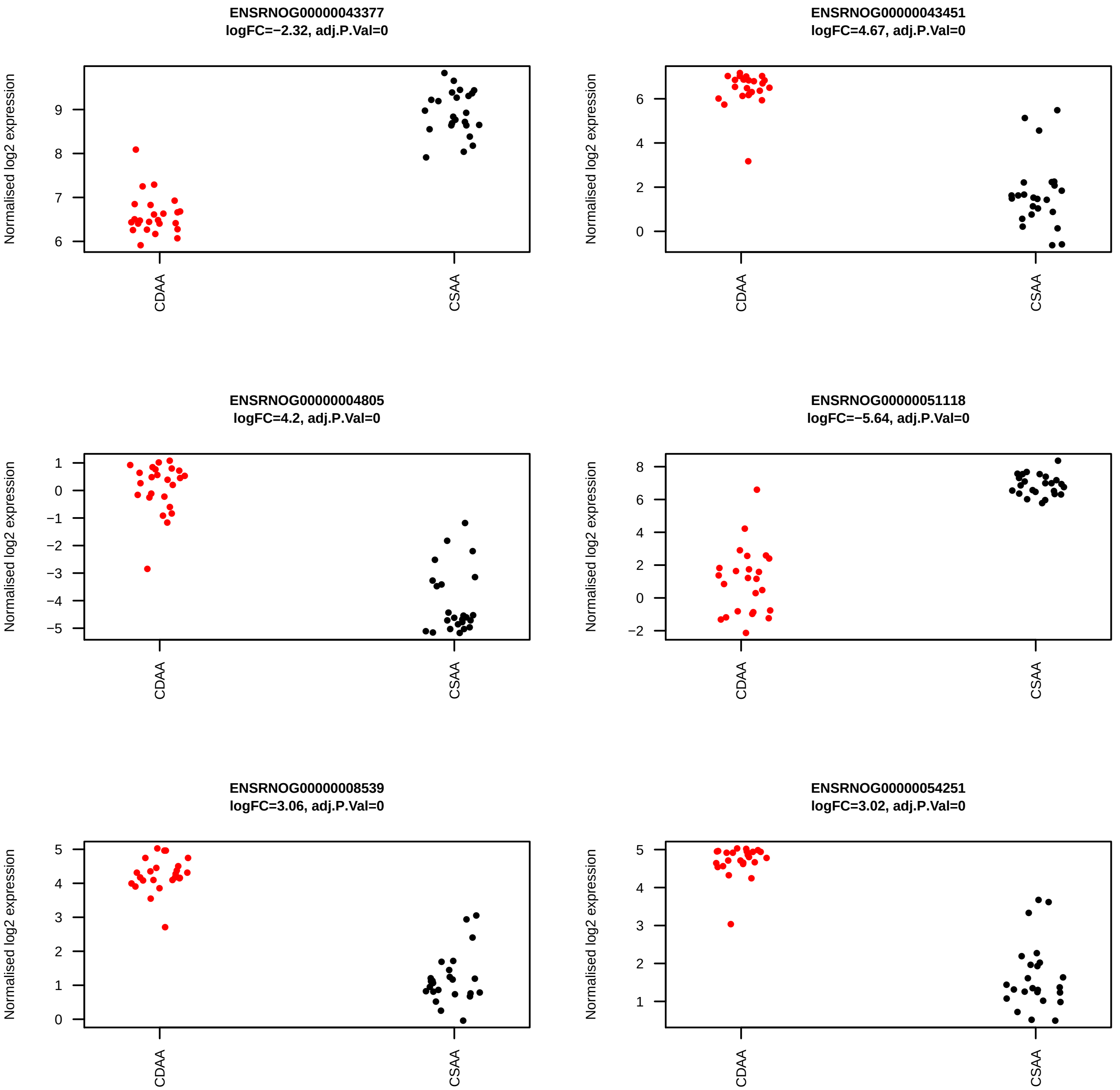
ENSRNOG00000043377
logFC=−2.32, adj.P.Val=0
ENSRNOG00000043451
logFC=4.67, adj.P.Val=0
ENSRNOG00000004805
logFC=4.2, adj.P.Val=0
ENSRNOG00000051118
logFC=−5.64, adj.P.Val=0
ENSRNOG00000008539
logFC=3.06, adj.P.Val=0
ENSRNOG00000054251
logFC=3.02, adj.P.Val=0
Normalised log2 expression
CDAA
CSAA

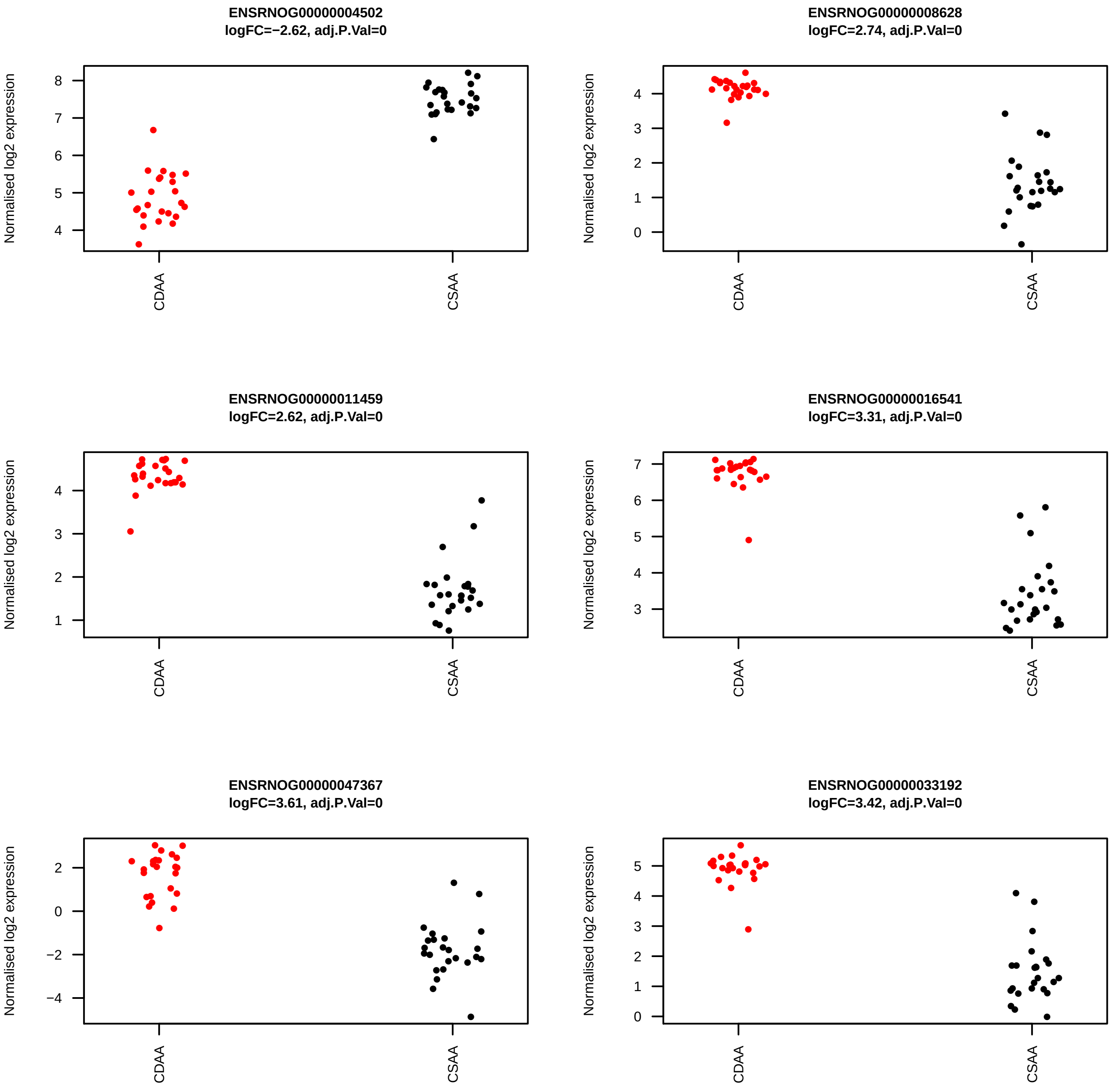
ENSRNOG00000004502
logFC=−2.62, adj.P.Val=0
ENSRNOG00000008628
logFC=2.74, adj.P.Val=0
ENSRNOG00000011459
logFC=2.62, adj.P.Val=0
ENSRNOG00000016541
logFC=3.31, adj.P.Val=0
ENSRNOG00000047367
logFC=3.61, adj.P.Val=0
ENSRNOG00000033192
logFC=3.42, adj.P.Val=0
Normalised log2 expression
CDAA
CSAA

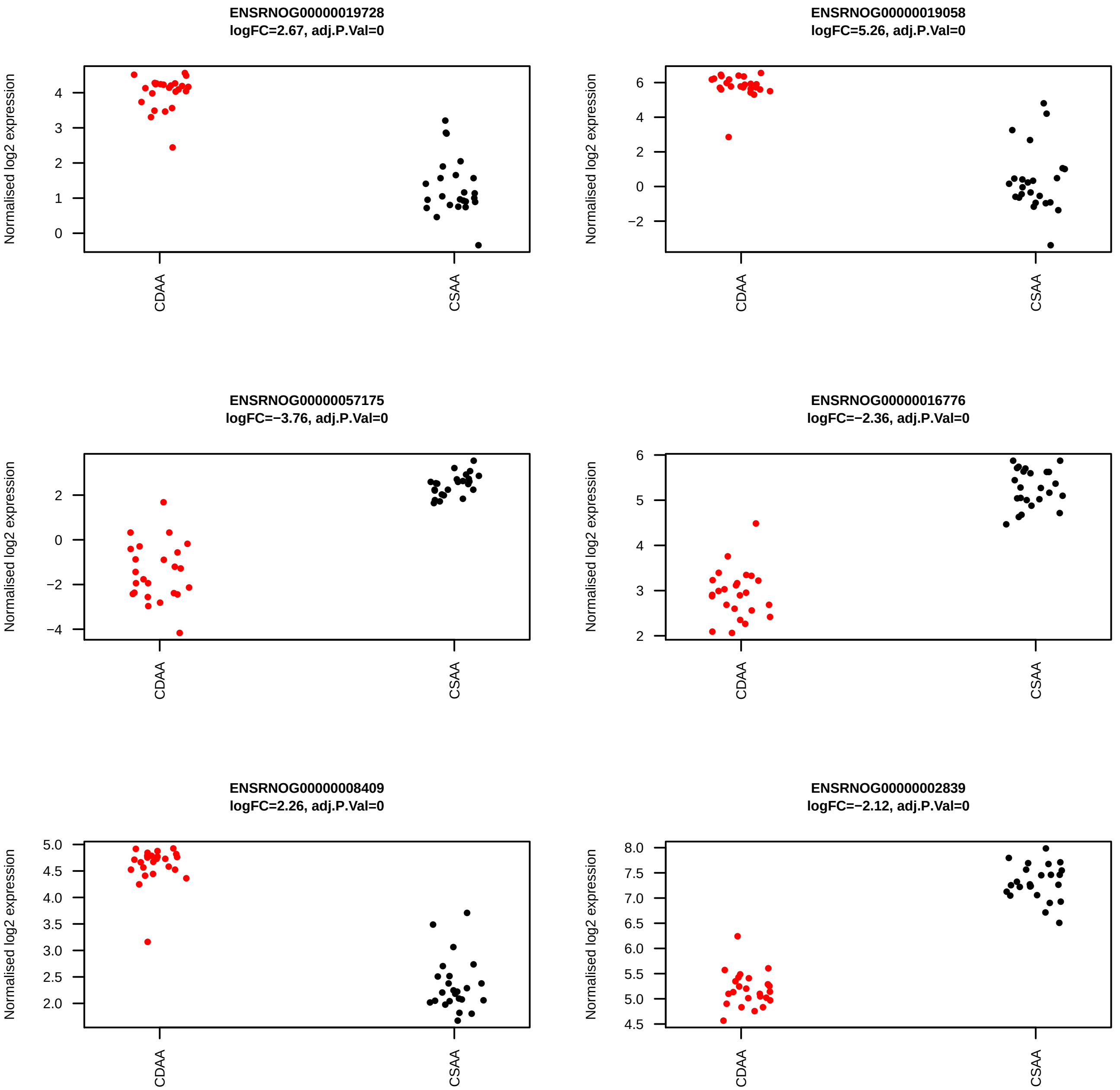
ENSRNOG00000019728
logFC=2.67, adj.P.Val=0
ENSRNOG00000019058
logFC=5.26, adj.P.Val=0
ENSRNOG00000057175
logFC=−3.76, adj.P.Val=0
ENSRNOG00000016776
logFC=−2.36, adj.P.Val=0
ENSRNOG00000008409
logFC=2.26, adj.P.Val=0
ENSRNOG00000002839
logFC=−2.12, adj.P.Val=0
Normalised log2 expression
CDAA
CSAA

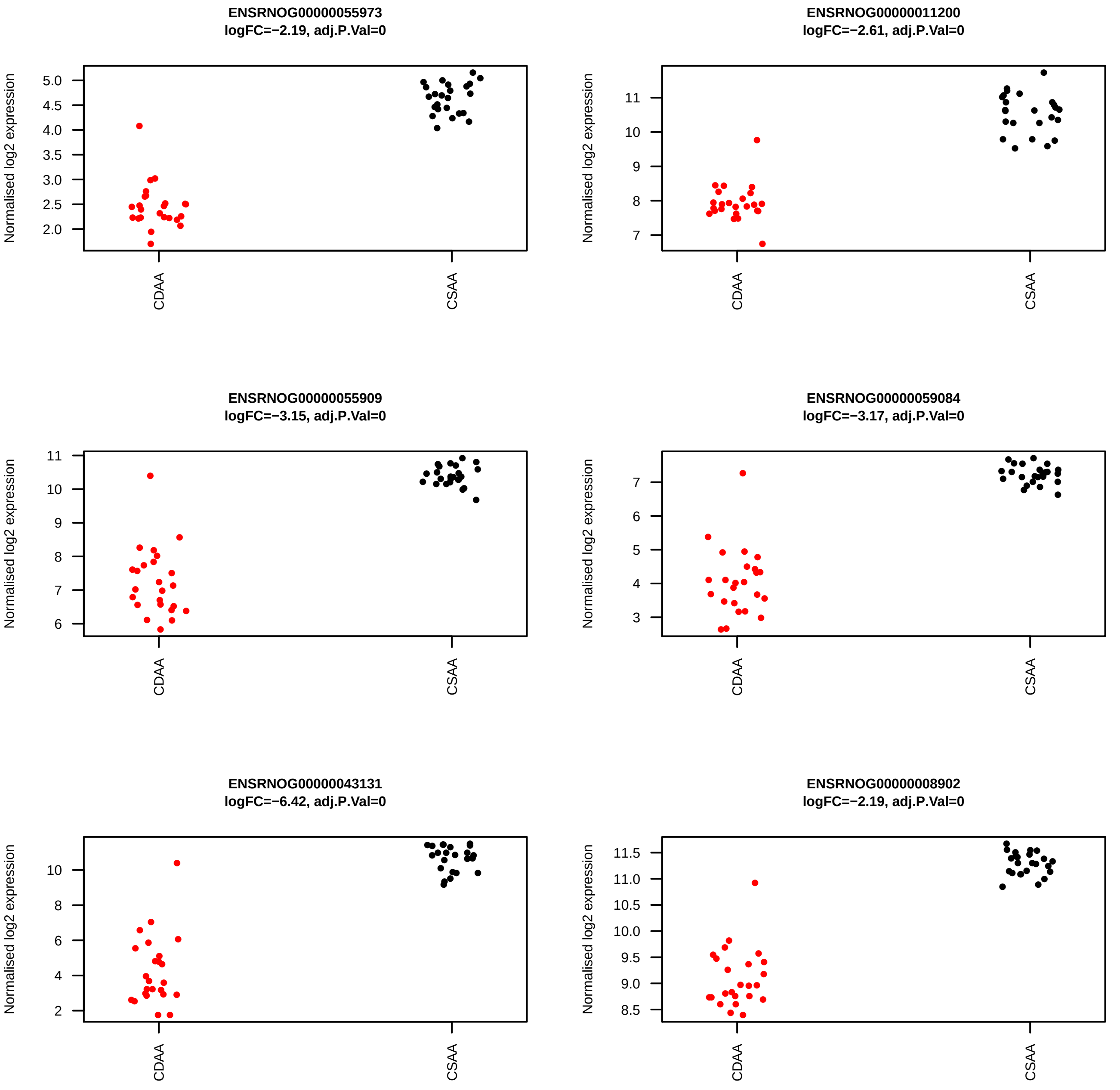
ENSRNOG00000055973
logFC=−2.19, adj.P.Val=0
Normalised log2 expression
2.0
2.5
3.0
3.5
4.0
4.5
5.0
CDAA
CSAA
ENSRNOG00000011200
logFC=−2.61, adj.P.Val=0
Normalised log2 expression
7
8
9
10
11
CDAA
CSAA
ENSRNOG00000055909
logFC=−3.15, adj.P.Val=0
Normalised log2 expression
6
7
8
9
10
11
CDAA
CSAA
ENSRNOG00000059084
logFC=−3.17, adj.P.Val=0
Normalised log2 expression
3
4
5
6
7
CDAA
CSAA
ENSRNOG00000043131
logFC=−6.42, adj.P.Val=0
Normalised log2 expression
2
4
6
8
10
CDAA
CSAA
ENSRNOG00000008902
logFC=−2.19, adj.P.Val=0
Normalised log2 expression
8.5
9.0
9.5
10.0
10.5
11.0
11.5
CDAA
CSAA

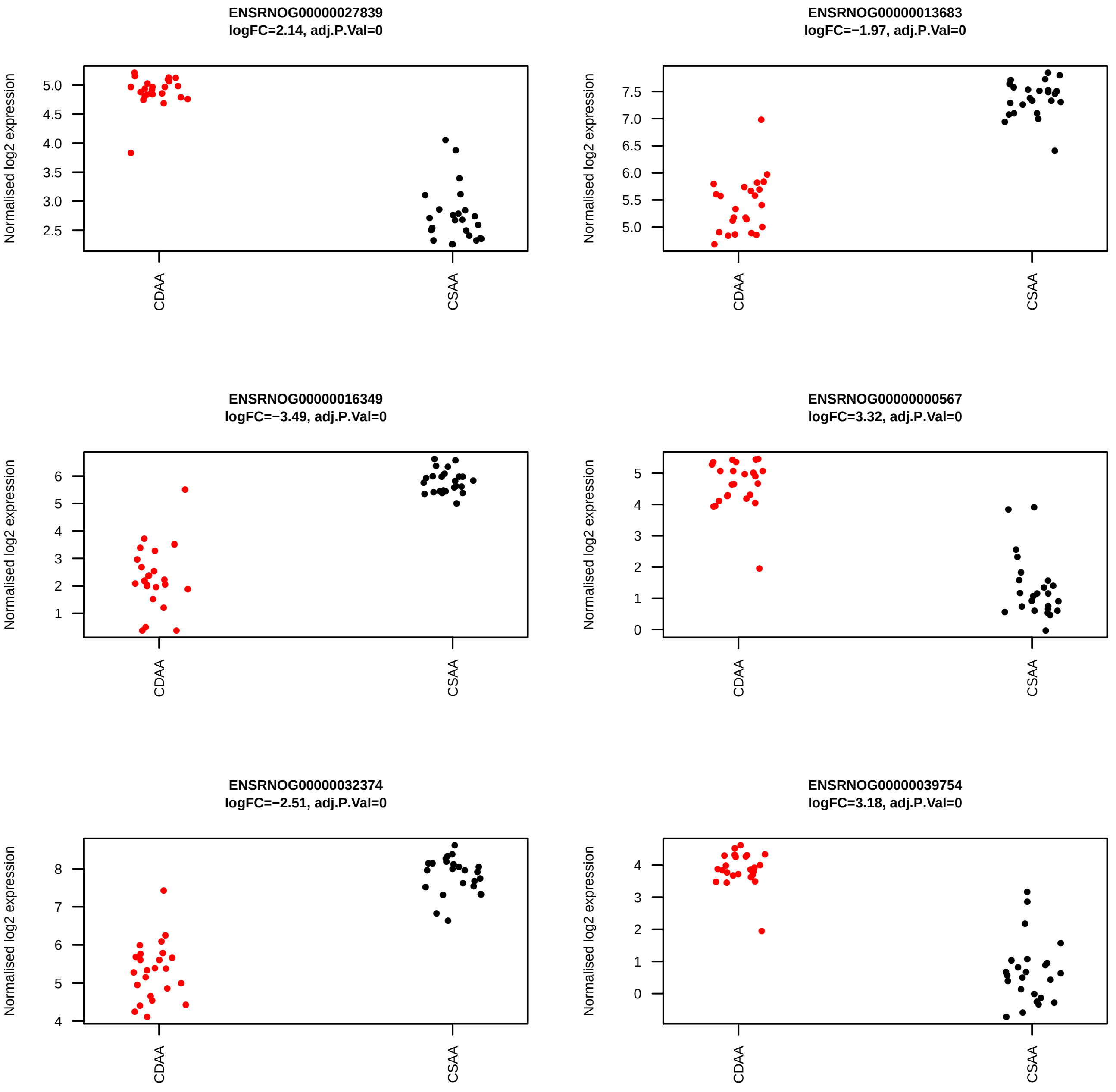
ENSRNOG00000027839
logFC=2.14, adj.P.Val=0
Normalised log2 expression
5.0
4.5
4.0
3.5
3.0
2.5
CDAA
CSAA
ENSRNOG00000013683
logFC=−1.97, adj.P.Val=0
Normalised log2 expression
7.5
7.0
6.5
6.0
5.5
5.0
CDAA
CSAA
ENSRNOG00000016349
logFC=−3.49, adj.P.Val=0
Normalised log2 expression
6
5
4
3
2
1
CDAA
CSAA
ENSRNOG00000000567
logFC=3.32, adj.P.Val=0
Normalised log2 expression
5
4
3
2
1
0
CDAA
CSAA
ENSRNOG00000032374
logFC=−2.51, adj.P.Val=0
Normalised log2 expression
8
7
6
5
4
CDAA
CSAA
ENSRNOG00000039754
logFC=3.18, adj.P.Val=0
Normalised log2 expression
4
3
2
1
0
CDAA
CSAA

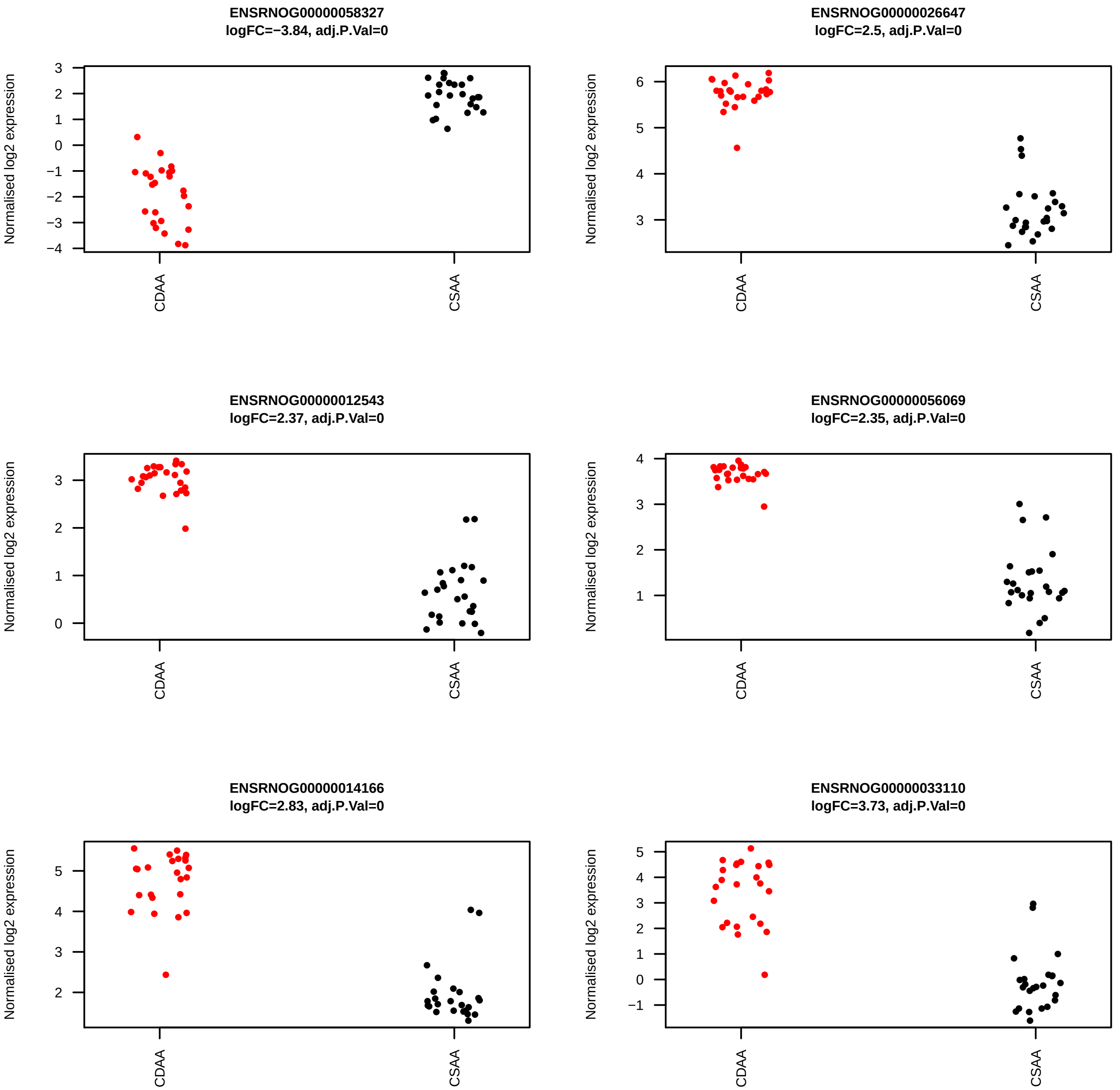
ENSRNOG00000058327
logFC=−3.84, adj.P.Val=0
ENSRNOG00000026647
logFC=2.5, adj.P.Val=0
ENSRNOG00000012543
logFC=2.37, adj.P.Val=0
ENSRNOG00000056069
logFC=2.35, adj.P.Val=0
ENSRNOG00000014166
logFC=2.83, adj.P.Val=0
ENSRNOG00000033110
logFC=3.73, adj.P.Val=0
Normalised log2 expression
CDAA
CSAA

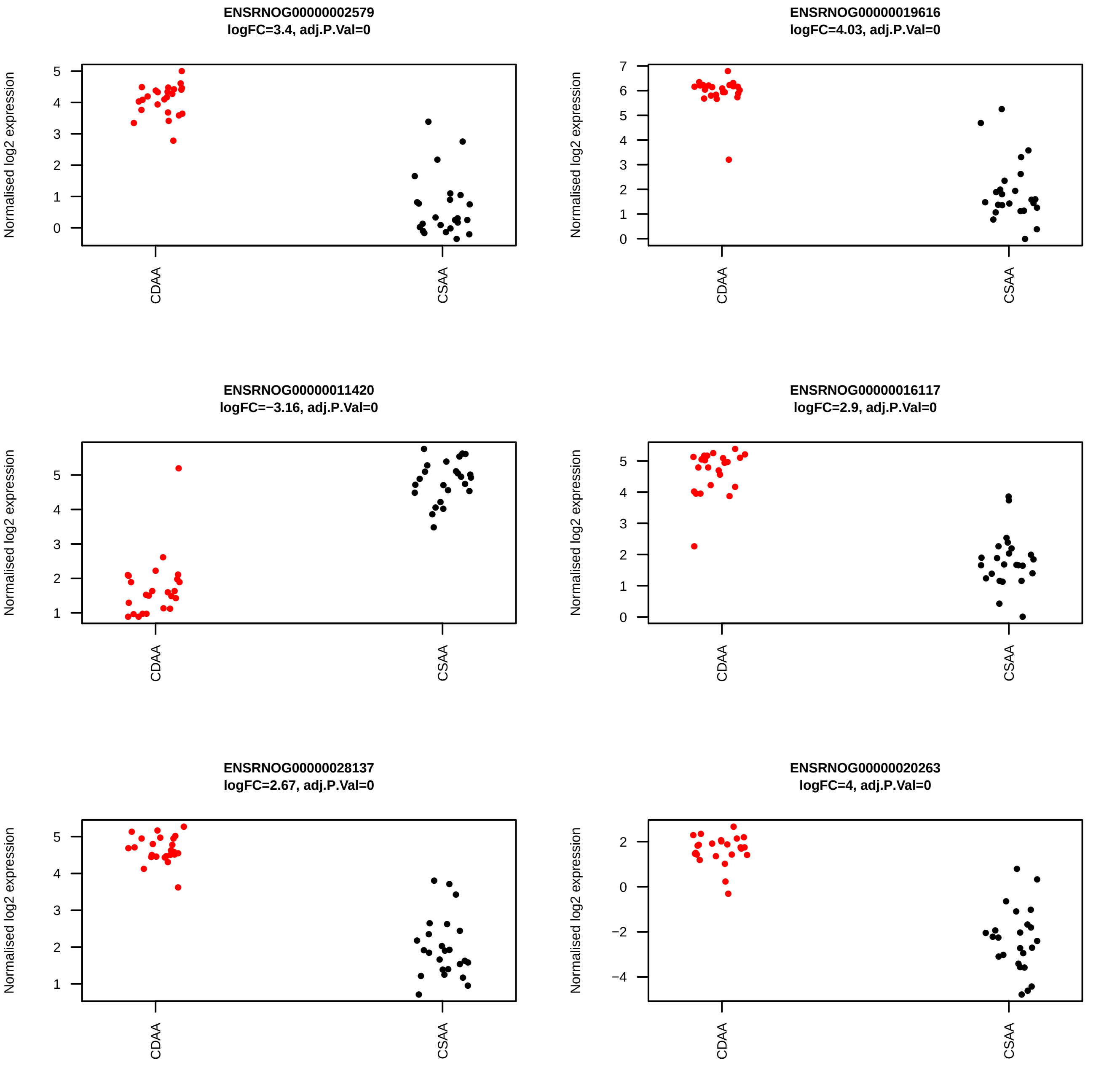
ENSRNOG00000002579
logFC=3.4, adj.P.Val=0
ENSRNOG00000019616
logFC=4.03, adj.P.Val=0
ENSRNOG00000011420
logFC=−3.16, adj.P.Val=0
ENSRNOG00000016117
logFC=2.9, adj.P.Val=0
ENSRNOG00000028137
logFC=2.67, adj.P.Val=0
ENSRNOG00000020263
logFC=4, adj.P.Val=0
Normalised log2 expression
CDAA
CSAA

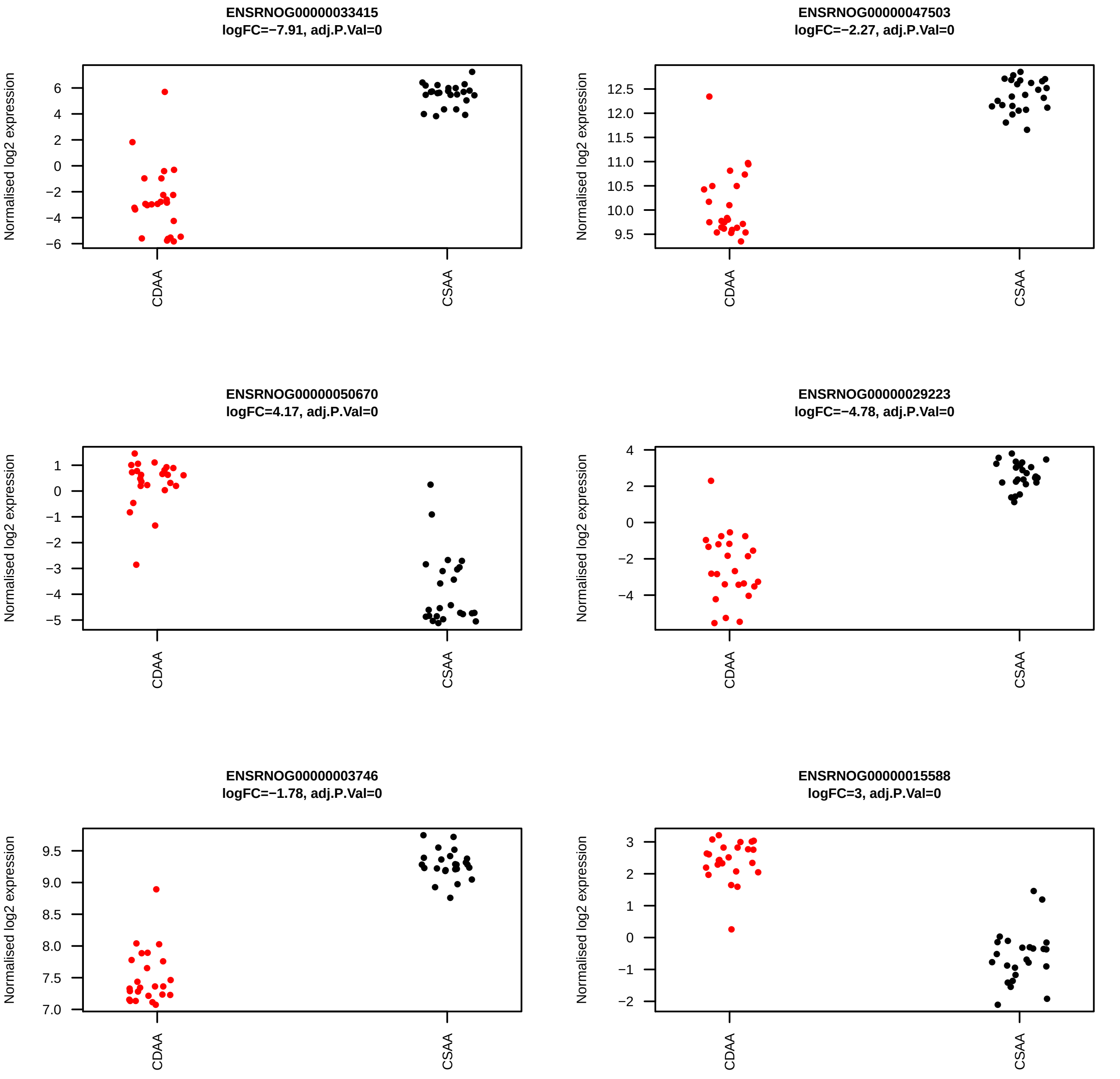
ENSRNOG00000033415
logFC=−7.91, adj.P.Val=0
Normalised log2 expression
6
4
2
0
−2
−4
−6
CDAA
CSAA
ENSRNOG00000047503
logFC=−2.27, adj.P.Val=0
Normalised log2 expression
12.5
12.0
11.5
11.0
10.5
10.0
9.5
CDAA
CSAA
ENSRNOG00000050670
logFC=4.17, adj.P.Val=0
Normalised log2 expression
1
0
−1
−2
−3
−4
−5
CDAA
CSAA
ENSRNOG00000029223
logFC=−4.78, adj.P.Val=0
Normalised log2 expression
4
2
0
−2
−4
CDAA
CSAA
ENSRNOG00000003746
logFC=−1.78, adj.P.Val=0
Normalised log2 expression
9.5
9.0
8.5
8.0
7.5
7.0
CDAA
CSAA
ENSRNOG00000015588
logFC=3, adj.P.Val=0
Normalised log2 expression
3
2
1
0
−1
−2
CDAA
CSAA

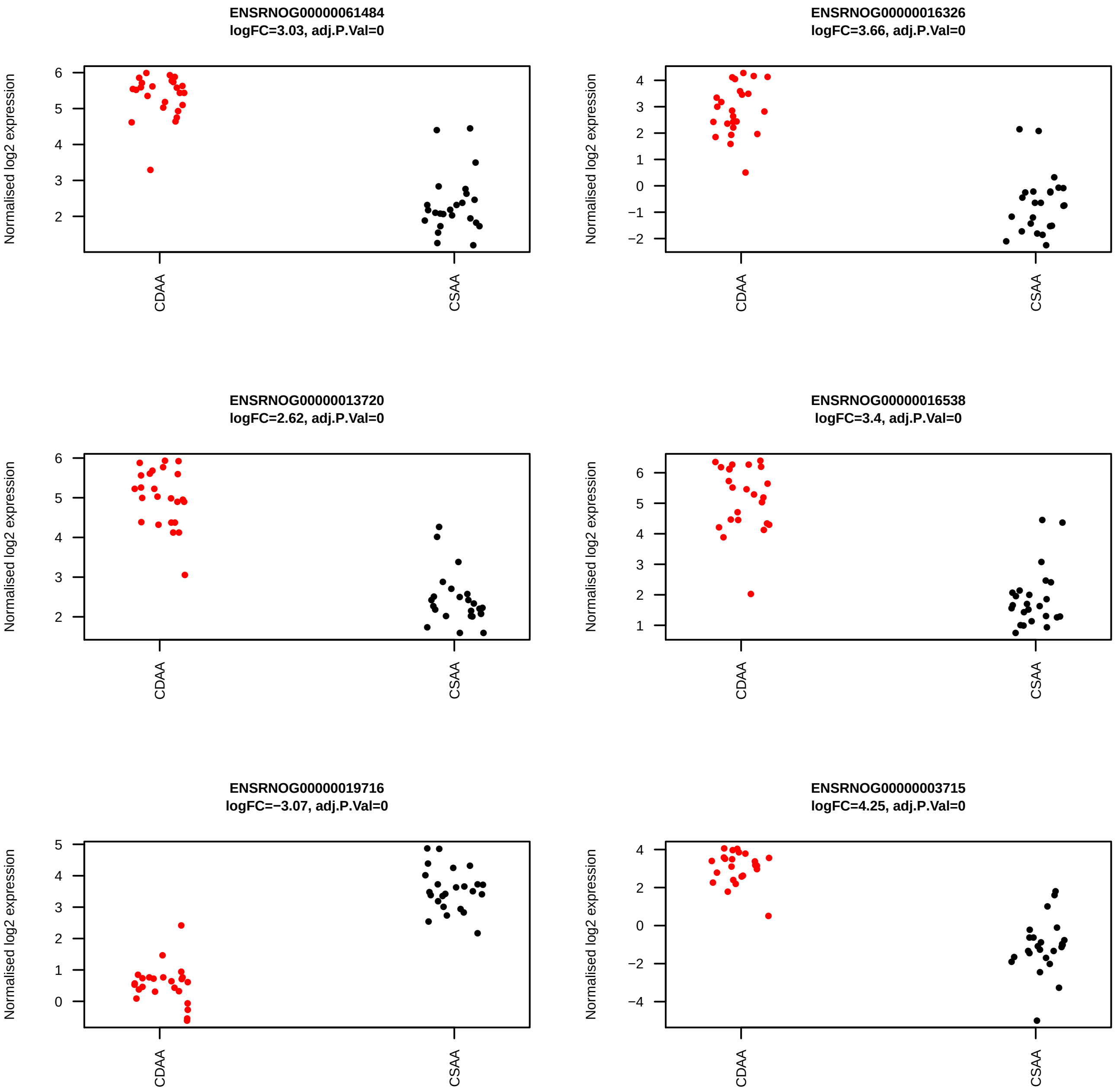
ENSRNOG00000061484
logFC=3.03, adj.P.Val=0
ENSRNOG00000016326
logFC=3.66, adj.P.Val=0
ENSRNOG00000013720
logFC=2.62, adj.P.Val=0
ENSRNOG00000016538
logFC=3.4, adj.P.Val=0
ENSRNOG00000019716
logFC=−3.07, adj.P.Val=0
ENSRNOG00000003715
logFC=4.25, adj.P.Val=0
Normalised log2 expression
CDAA
CSAA

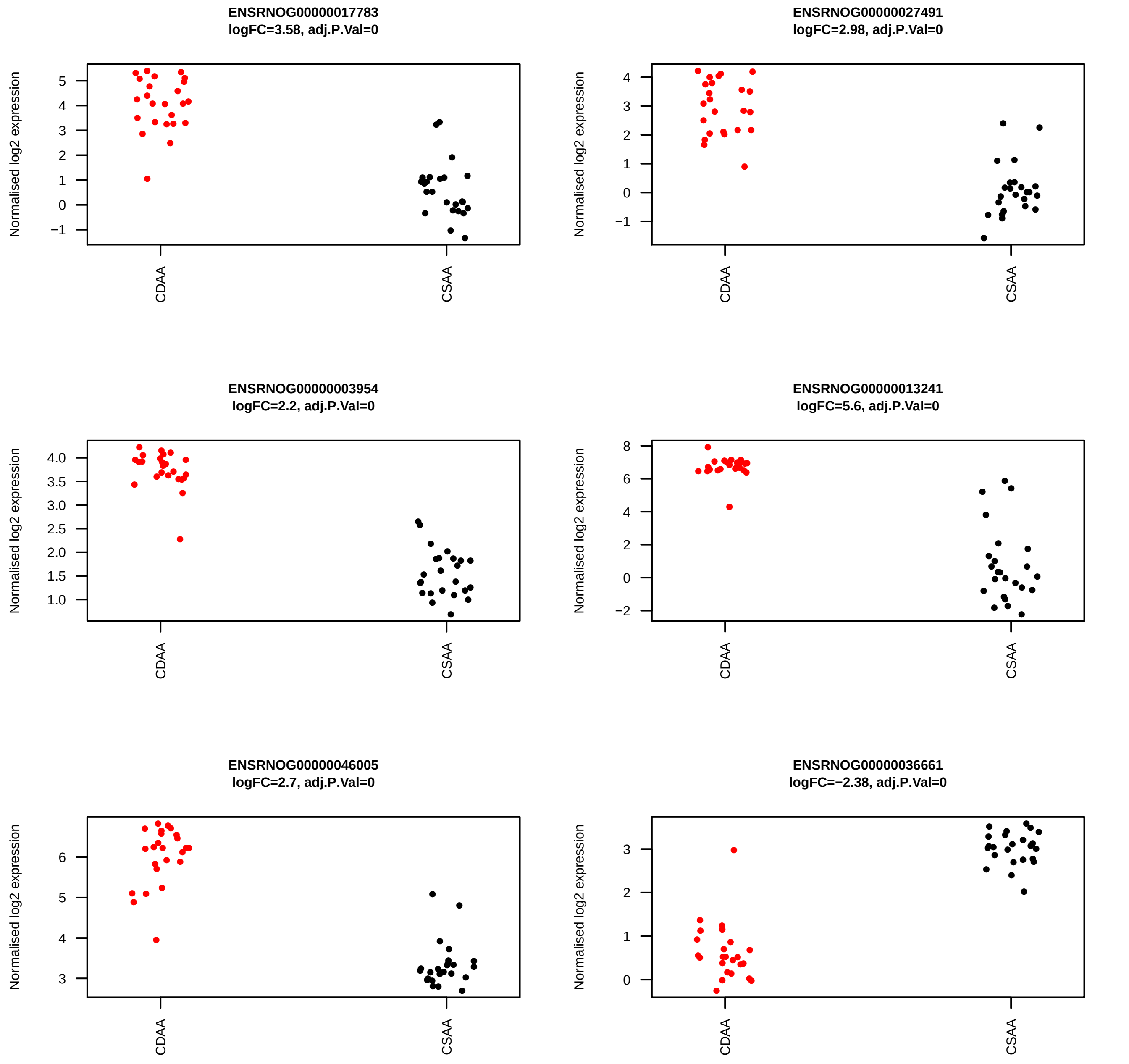
ENSRNOG00000017783
logFC=3.58, adj.P.Val=0
ENSRNOG00000027491
logFC=2.98, adj.P.Val=0
ENSRNOG00000003954
logFC=2.2, adj.P.Val=0
ENSRNOG00000013241
logFC=5.6, adj.P.Val=0
ENSRNOG00000046005
logFC=2.7, adj.P.Val=0
ENSRNOG00000036661
logFC=−2.38, adj.P.Val=0
Normalised log2 expression
CDAA
CSAA

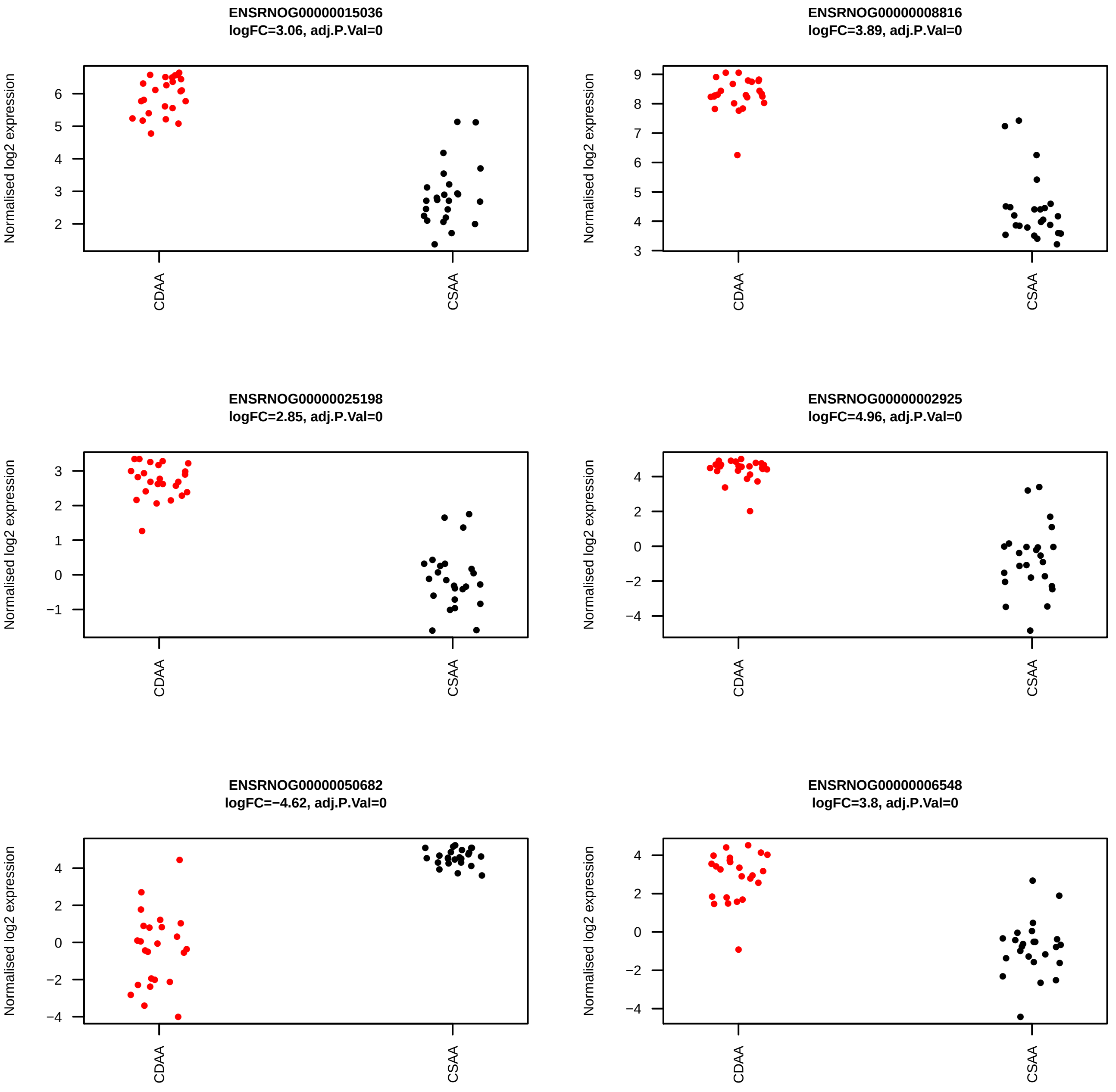
ENSRNOG00000015036
logFC=3.06, adj.P.Val=0
ENSRNOG00000008816
logFC=3.89, adj.P.Val=0
ENSRNOG00000025198
logFC=2.85, adj.P.Val=0
ENSRNOG00000002925
logFC=4.96, adj.P.Val=0
ENSRNOG00000050682
logFC=−4.62, adj.P.Val=0
ENSRNOG00000006548
logFC=3.8, adj.P.Val=0
Normalised log2 expression
CDAA
CSAA

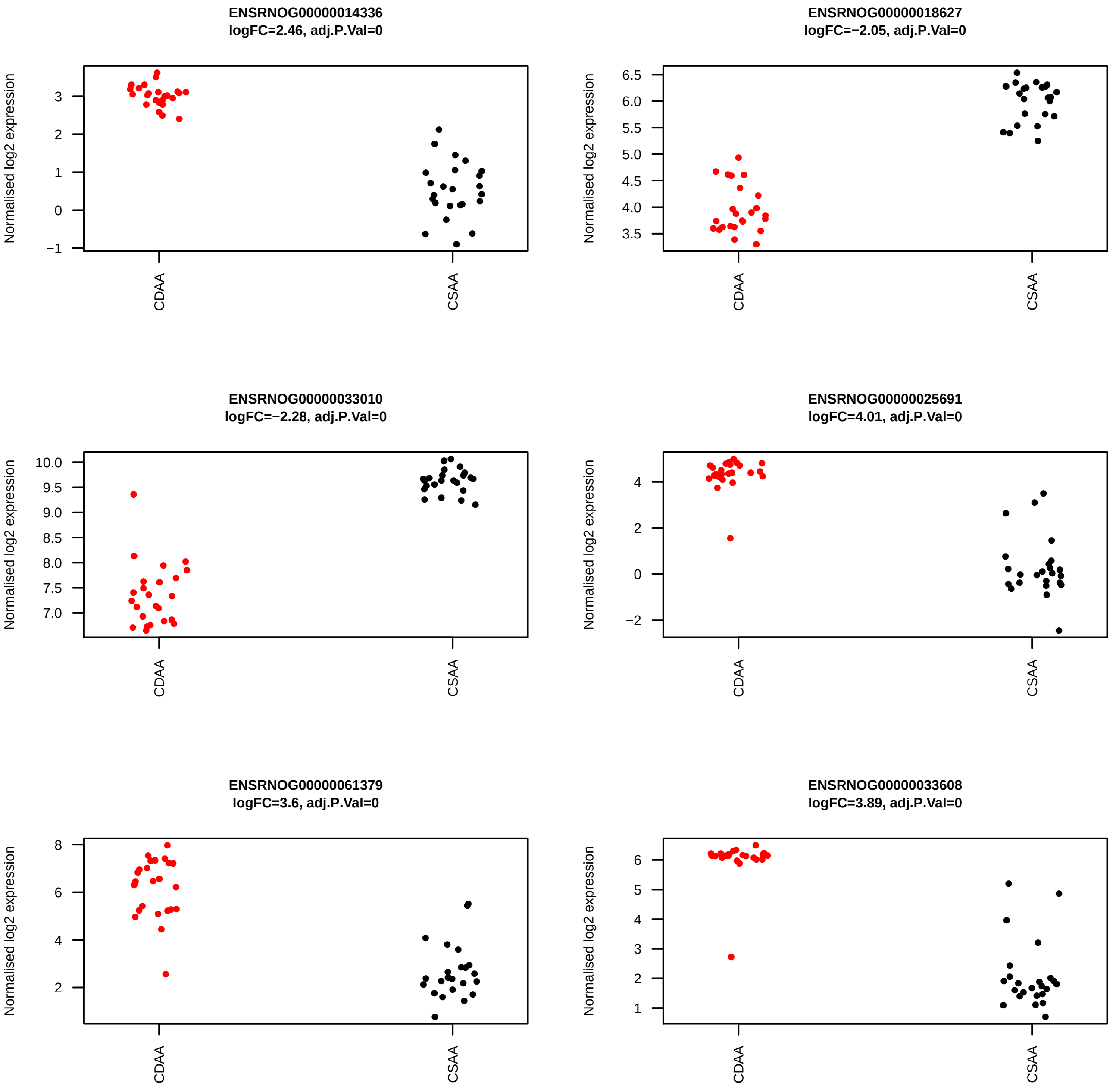
ENSRNOG00000014336
logFC=2.46, adj.P.Val=0
Normalised log2 expression
−1
0
1
2
3
CDAA
CSAA
ENSRNOG00000018627
logFC=−2.05, adj.P.Val=0
Normalised log2 expression
3.5
4.0
4.5
5.0
5.5
6.0
6.5
CDAA
CSAA
ENSRNOG00000033010
logFC=−2.28, adj.P.Val=0
Normalised log2 expression
7.0
7.5
8.0
8.5
9.0
9.5
10.0
CDAA
CSAA
ENSRNOG00000025691
logFC=4.01, adj.P.Val=0
Normalised log2 expression
−2
0
2
4
CDAA
CSAA
ENSRNOG00000061379
logFC=3.6, adj.P.Val=0
Normalised log2 expression
2
4
6
8
CDAA
CSAA
ENSRNOG00000033608
logFC=3.89, adj.P.Val=0
Normalised log2 expression
1
2
3
4
5
6
CDAA
CSAA

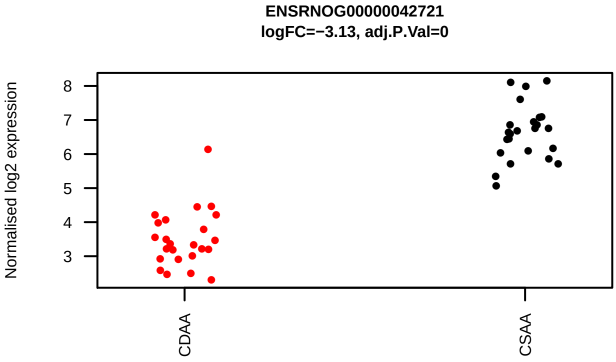
ENSRNOG00000042721
logFC=−3.13, adj.P.Val=0
Normalised log2 expression
8
7
6
5
4
3
CDAA
CSAA

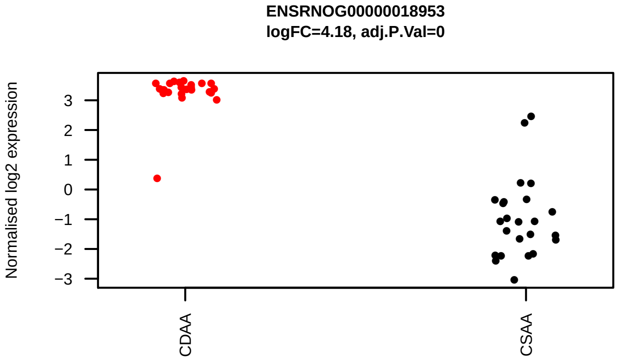
ENSRNOG00000018953
logFC=4.18, adj.P.Val=0
Normalised log2 expression
3
2
1
0
−1
−2
−3
CDAA
CSAA

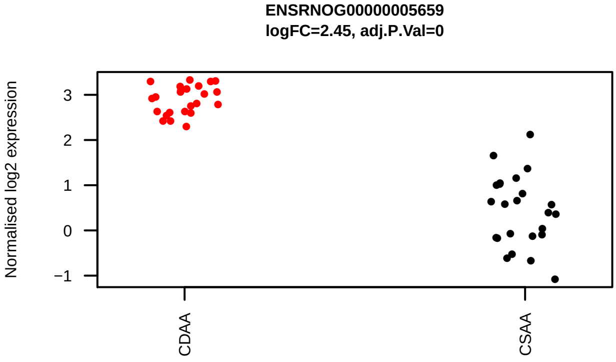
ENSRNOG00000005659
logFC=2.45, adj.P.Val=0
Normalised log2 expression
3
2
1
0
−1
CDAA
CSAA

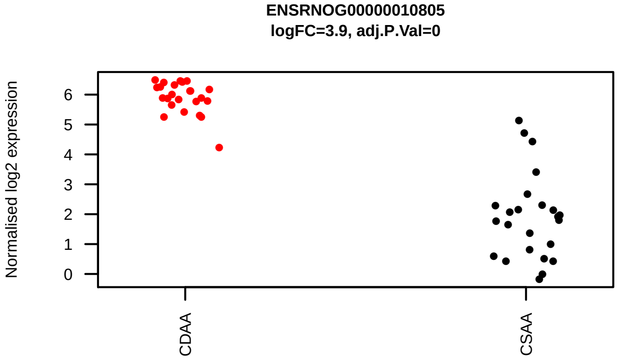
ENSRNOG00000010805
logFC=3.9, adj.P.Val=0
Normalised log2 expression
6
5
4
3
2
1
0
CDAA
CSAA
