# Supplementary material for: GIANT: galaxy-based tool for interactive analysis of transcriptomic data
Source: Sci Rep. 2020 Nov 16;10:19835. doi: 10.1038/s41598-020-76769-w (PMC7670435; doi:10.1038/s41598-020-76769-w)
Supplement: Supplementary file 2 — Supplementary Information. [file 41598_2020_76769_MOESM2_ESM.zip › Tools_InputOutput_Parameters/RNA-seq_workflow/Step1-Normalization_Differential/Outputs/extra_files_path_5/mdplots_samples.pdf]

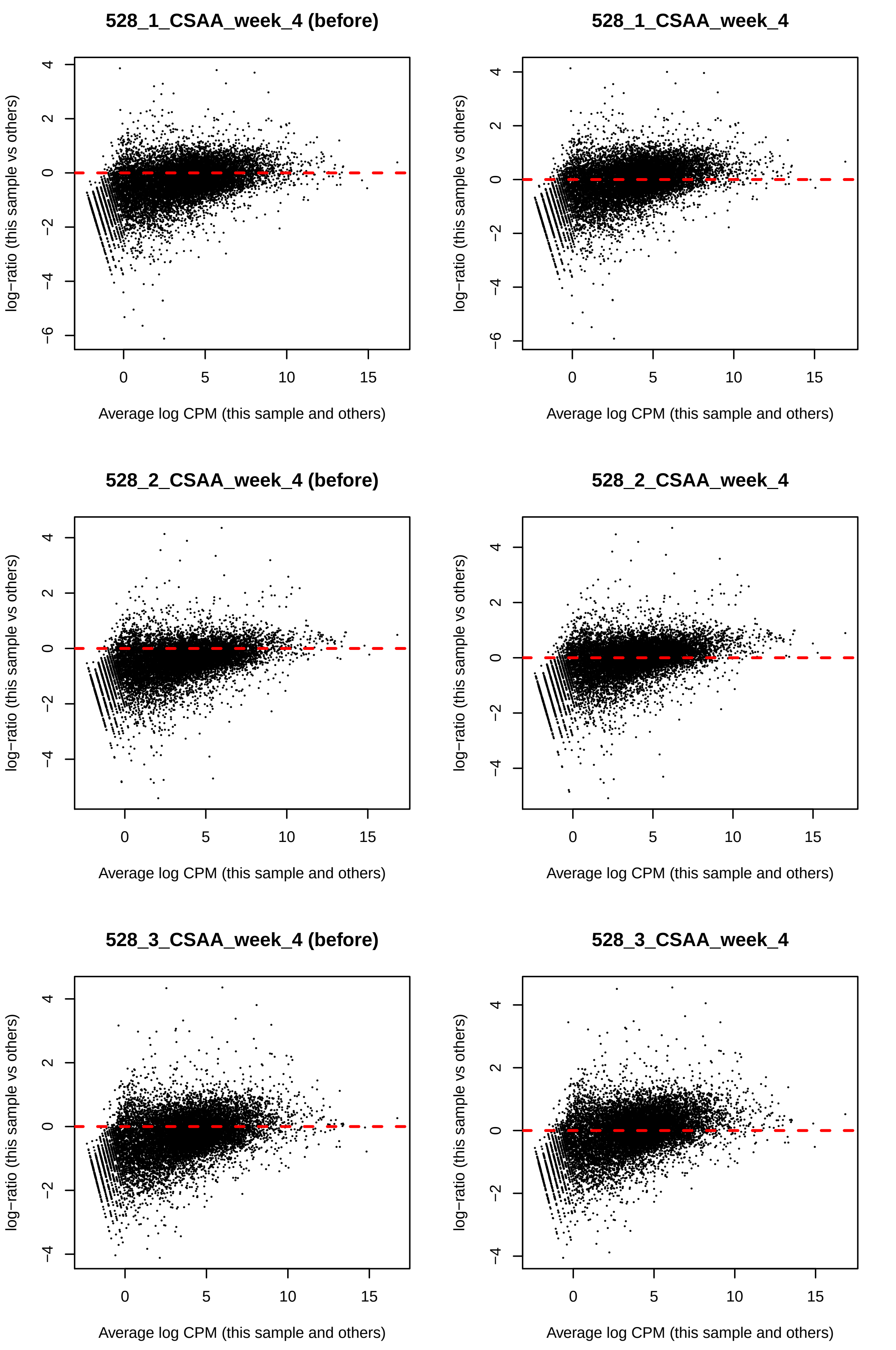
528_1_CSAA_week_4 (before)
528_1_CSAA_week_4
528_2_CSAA_week_4 (before)
528_2_CSAA_week_4
528_3_CSAA_week_4 (before)
528_3_CSAA_week_4
log−ratio (this sample vs others)
Average log CPM (this sample and others)

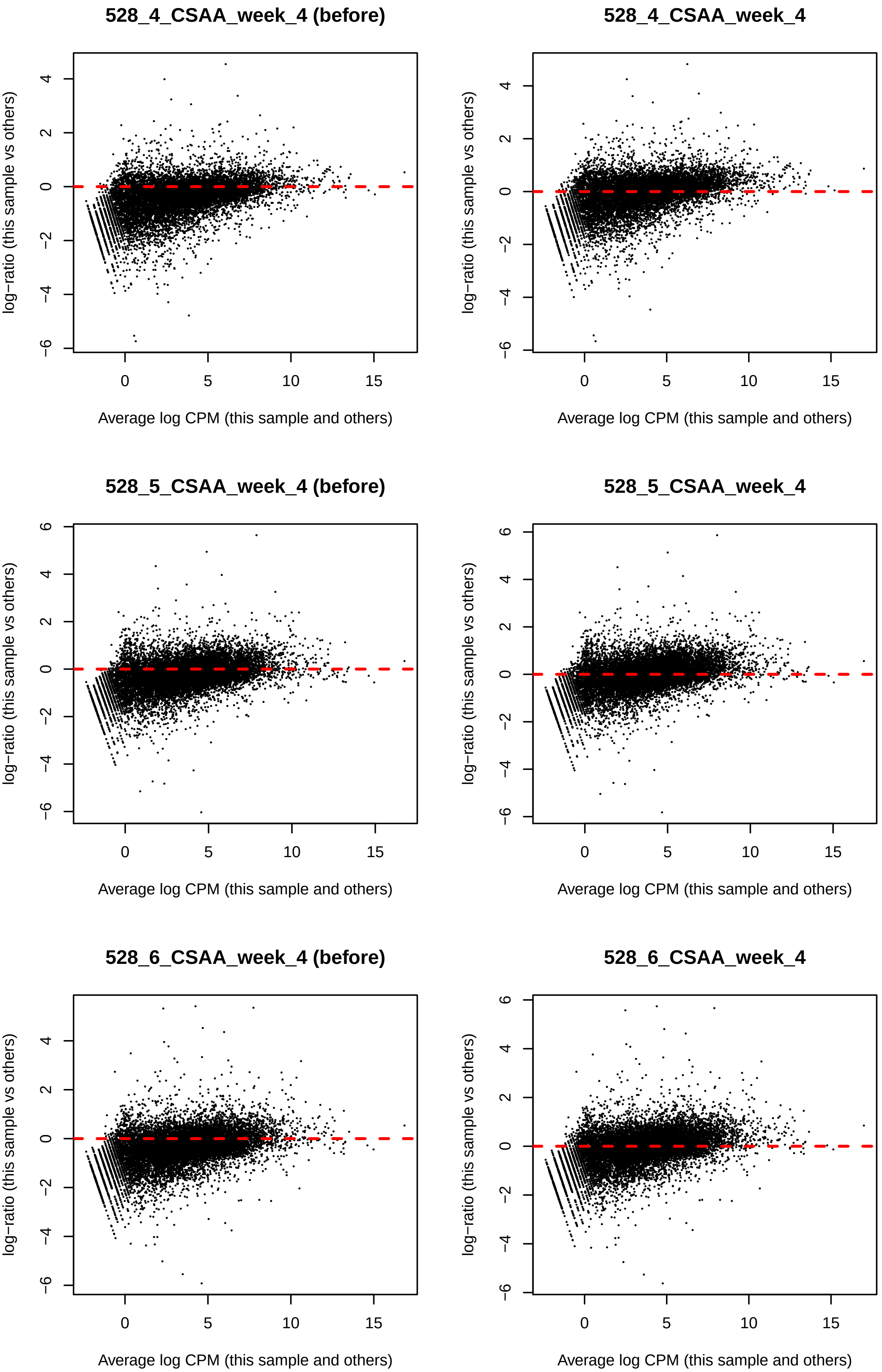
528_4_CSAA_week_4 (before)
528_4_CSAA_week_4
528_5_CSAA_week_4 (before)
528_5_CSAA_week_4
528_6_CSAA_week_4 (before)
528_6_CSAA_week_4
log−ratio (this sample vs others)
Average log CPM (this sample and others)

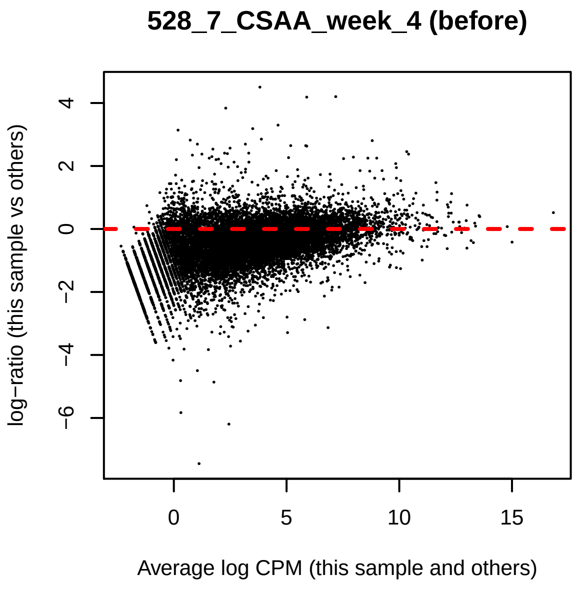
528_7_CSAA_week_4 (before)
log−ratio (this sample vs others)
Average log CPM (this sample and others)

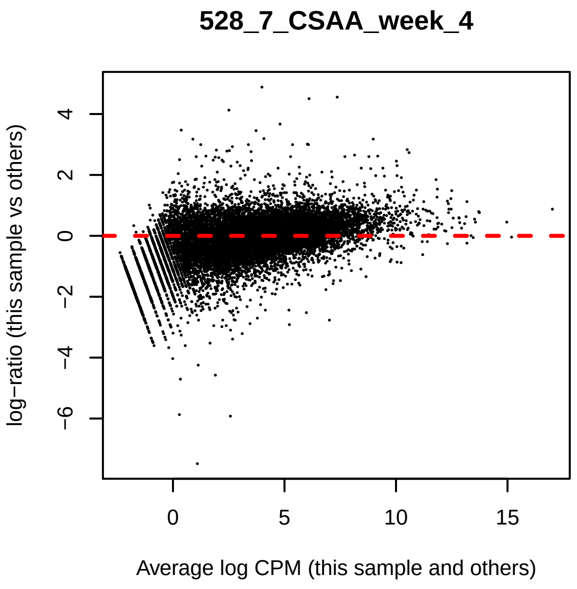
528_7_CSAA_week_4
log−ratio (this sample vs others)
Average log CPM (this sample and others)

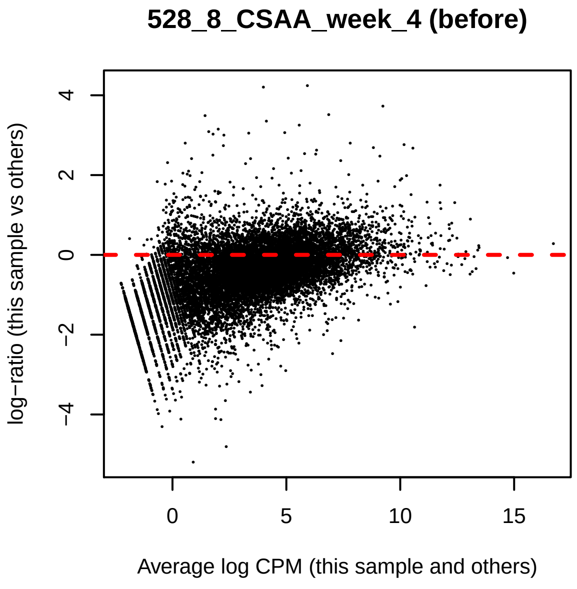
528_8_CSAA_week_4 (before)
log−ratio (this sample vs others)
Average log CPM (this sample and others)

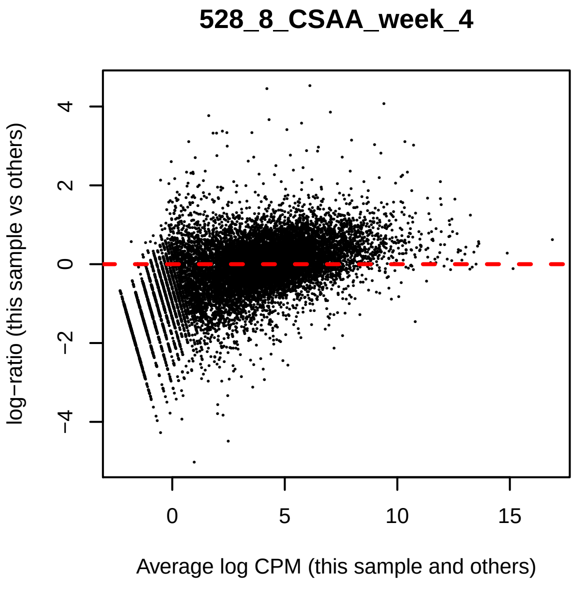
528_8_CSAA_week_4
log−ratio (this sample vs others)
Average log CPM (this sample and others)

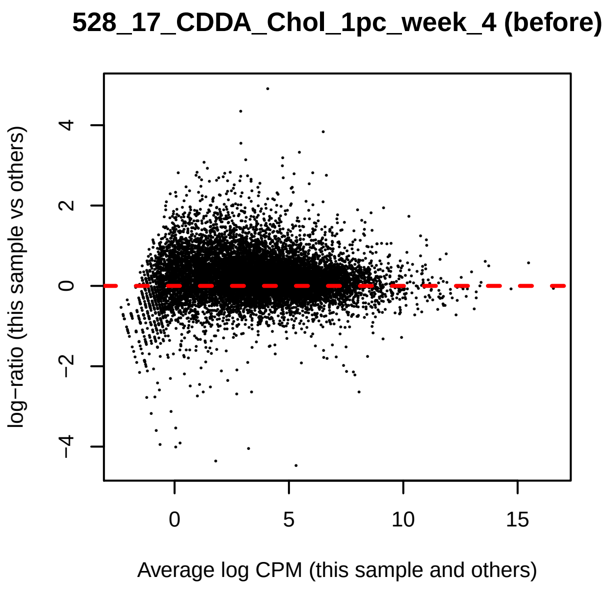
528_17_CDDA_Chol_1pc_week_4 (before)
log−ratio (this sample vs others)
Average log CPM (this sample and others)

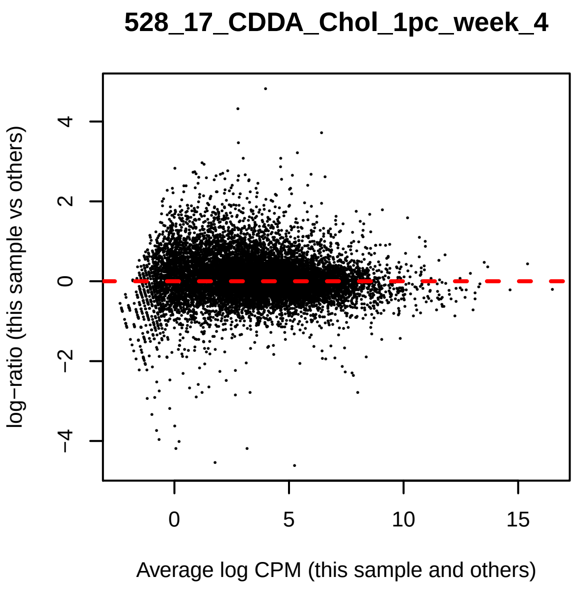
528_17_CDDA_Chol_1pc_week_4
log−ratio (this sample vs others)
Average log CPM (this sample and others)

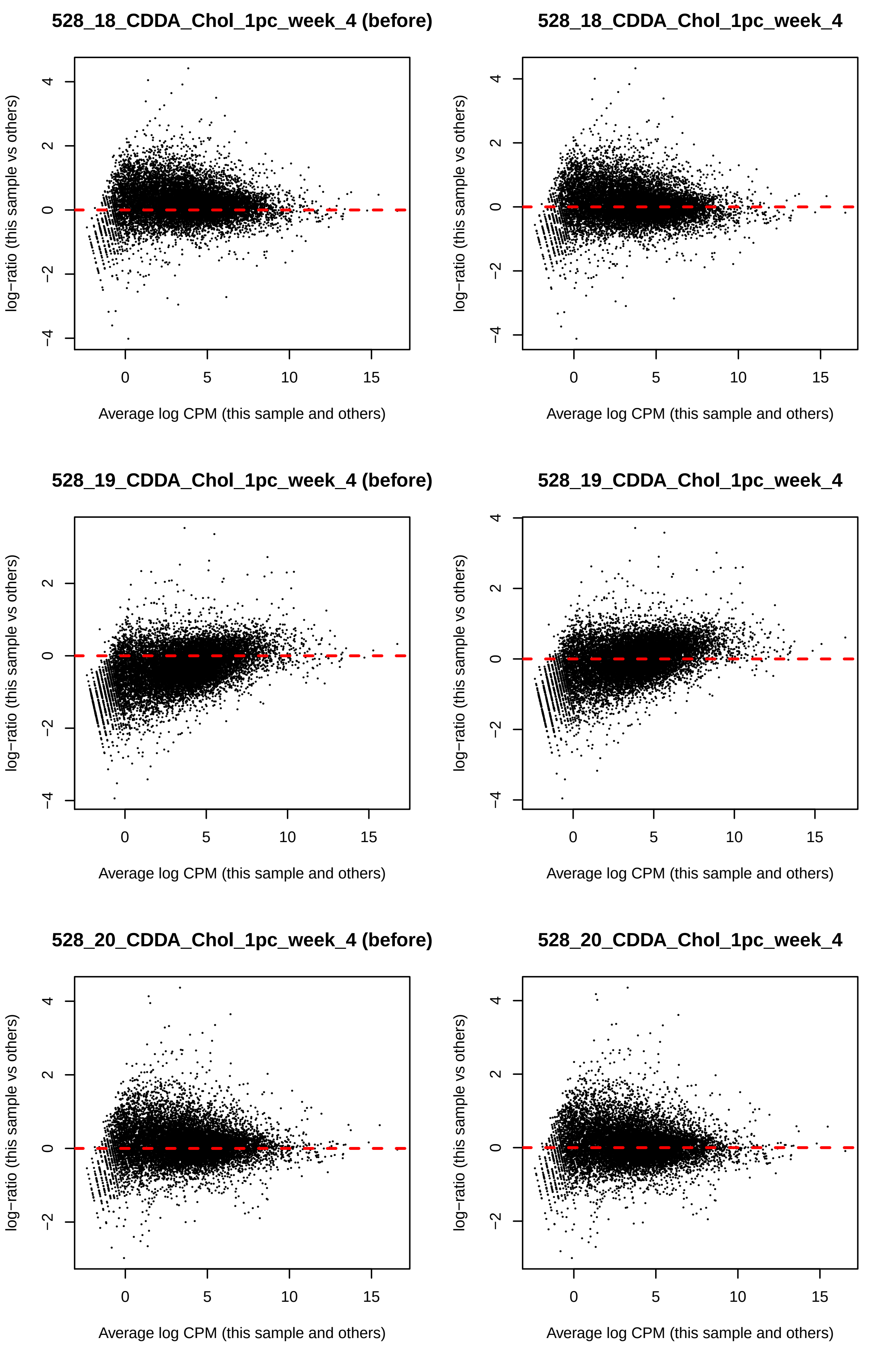
528_18_CDDA_Chol_1pc_week_4 (before)
528_18_CDDA_Chol_1pc_week_4
528_19_CDDA_Chol_1pc_week_4 (before)
528_19_CDDA_Chol_1pc_week_4
528_20_CDDA_Chol_1pc_week_4 (before)
528_20_CDDA_Chol_1pc_week_4
log−ratio (this sample vs others)
Average log CPM (this sample and others)
−4
−2
0
2
4
0
5
10
15

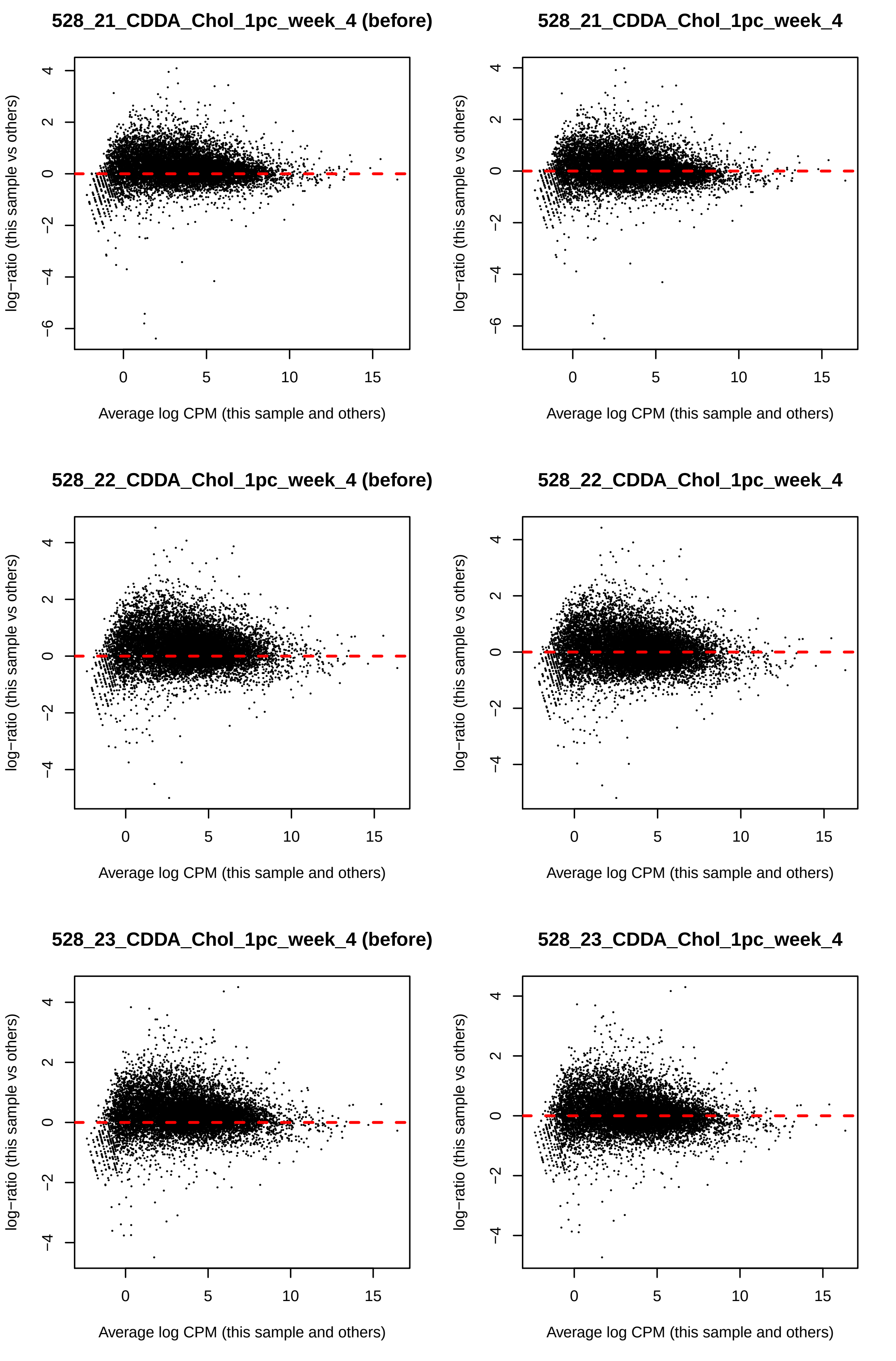

528_21_CDDA_Chol_1pc_week_4 (before)
528_21_CDDA_Chol_1pc_week_4
528_22_CDDA_Chol_1pc_week_4 (before)
528_22_CDDA_Chol_1pc_week_4
528_23_CDDA_Chol_1pc_week_4 (before)
528_23_CDDA_Chol_1pc_week_4
log−ratio (this sample vs others)
Average log CPM (this sample and others)

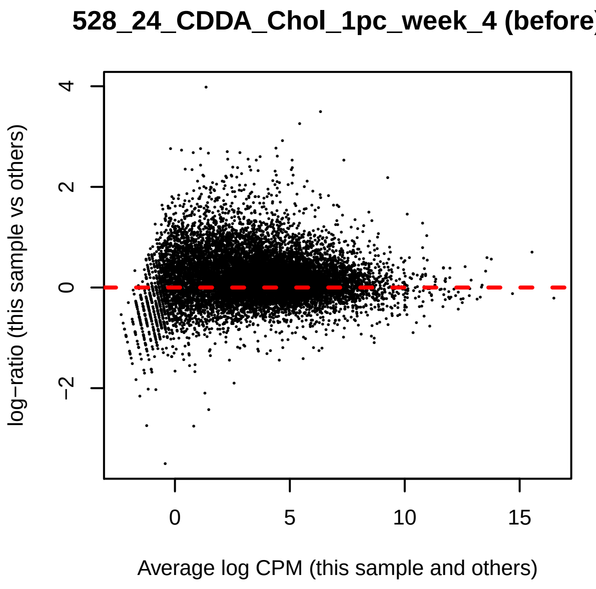
528_24_CDDA_Chol_1pc_week_4 (before)
log−ratio (this sample vs others)
Average log CPM (this sample and others)
4
2
0
−2
0
5
10
15

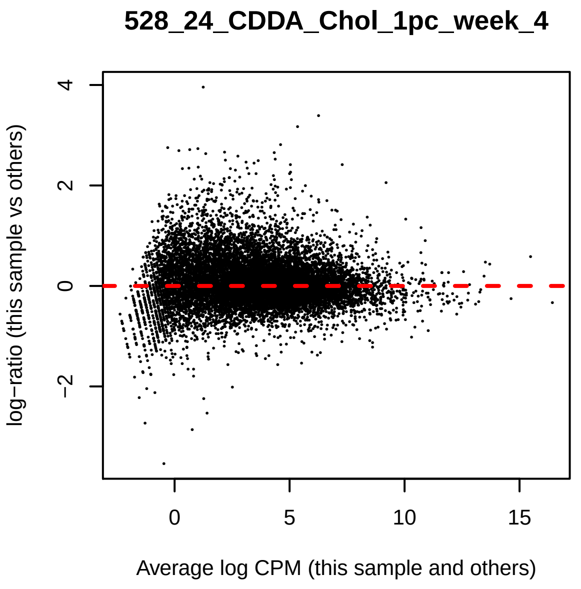
528_24_CDDA_Chol_1pc_week_4
log−ratio (this sample vs others)
Average log CPM (this sample and others)
4
2
0
−2
0
5
10
15

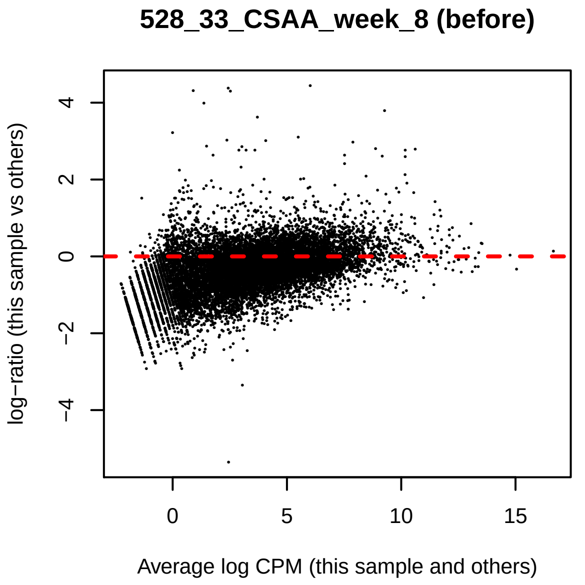
528_33_CSAA_week_8 (before)
log−ratio (this sample vs others)
Average log CPM (this sample and others)
4
2
0
−2
−4
0
5
10
15

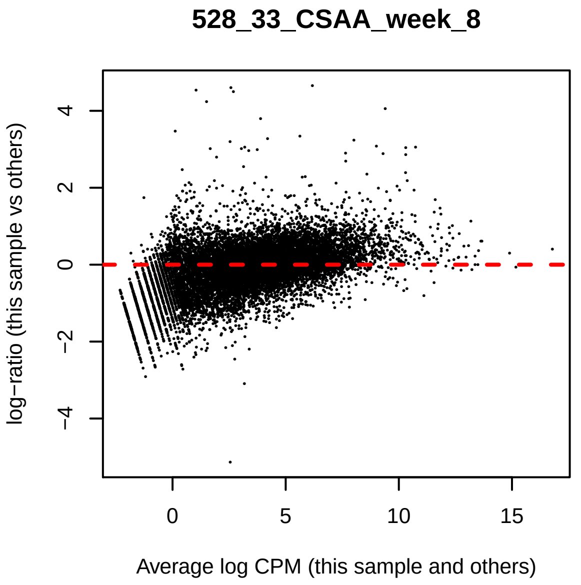
528_33_CSAA_week_8
log−ratio (this sample vs others)
Average log CPM (this sample and others)
4
2
0
−2
−4
0
5
10
15

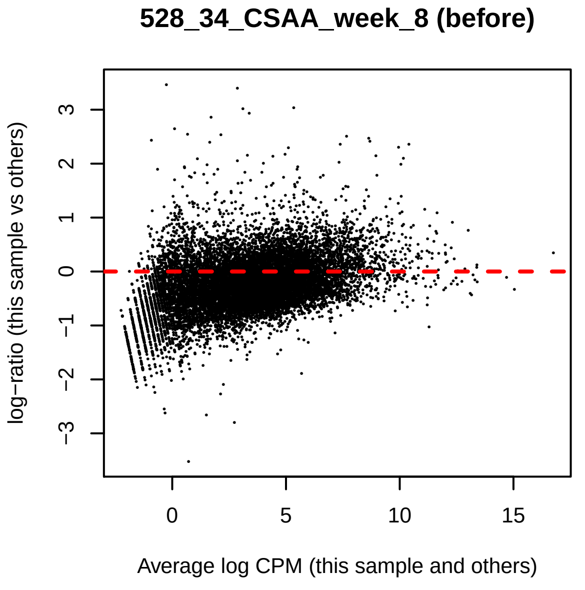
528_34_CSAA_week_8 (before)
log−ratio (this sample vs others)
Average log CPM (this sample and others)
3
2
1
0
−1
−2
−3
0
5
10
15

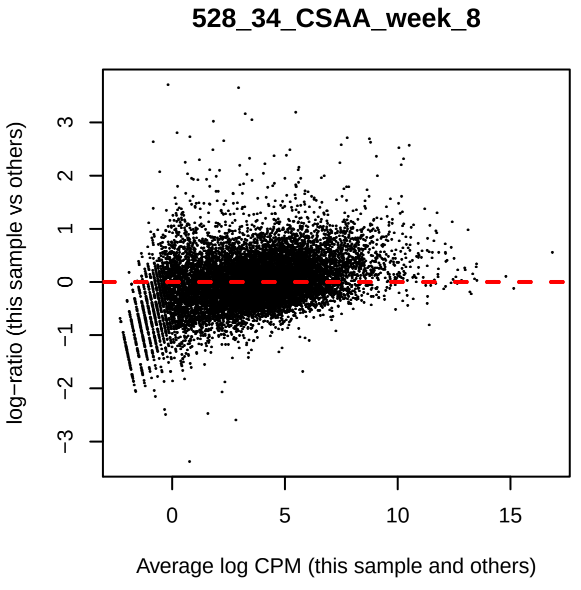
528_34_CSAA_week_8
log−ratio (this sample vs others)
Average log CPM (this sample and others)
3
2
1
0
−1
−2
−3
0
5
10
15

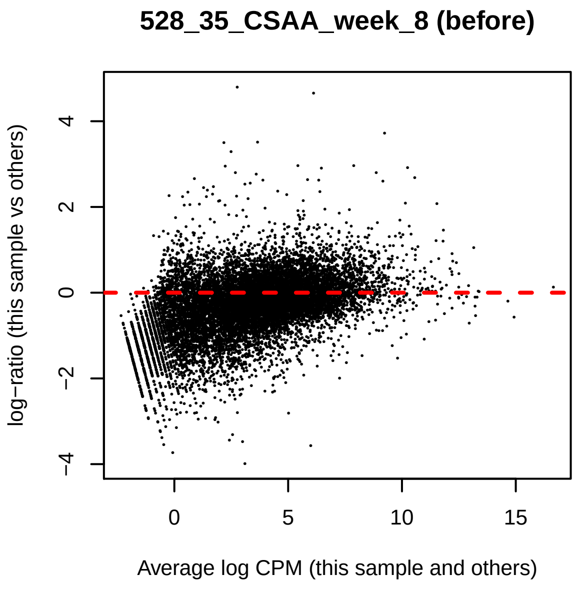
528_35_CSAA_week_8 (before)
log−ratio (this sample vs others)
Average log CPM (this sample and others)

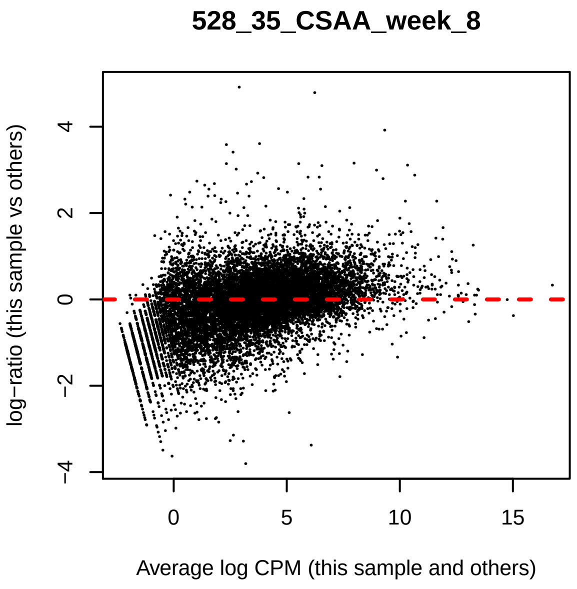
528_35_CSAA_week_8
log−ratio (this sample vs others)
Average log CPM (this sample and others)

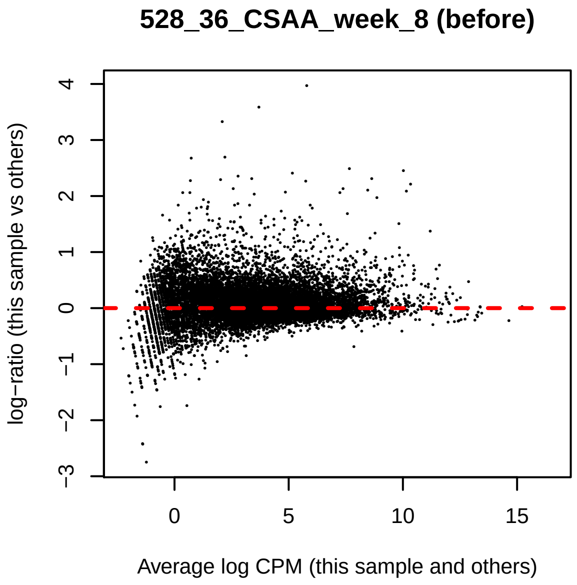
528_36_CSAA_week_8 (before)
log−ratio (this sample vs others)
Average log CPM (this sample and others)

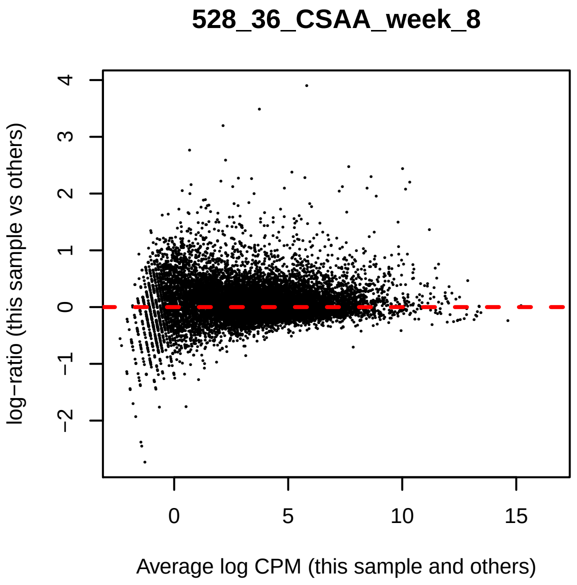
528_36_CSAA_week_8
log−ratio (this sample vs others)
Average log CPM (this sample and others)

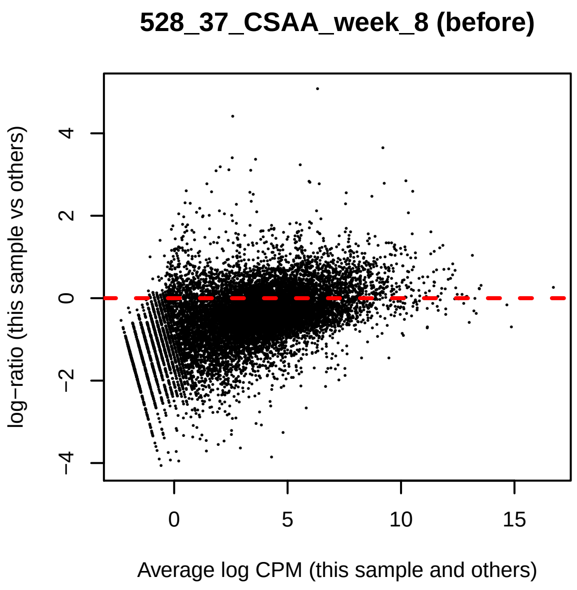
528_37_CSAA_week_8 (before)
log−ratio (this sample vs others)
Average log CPM (this sample and others)

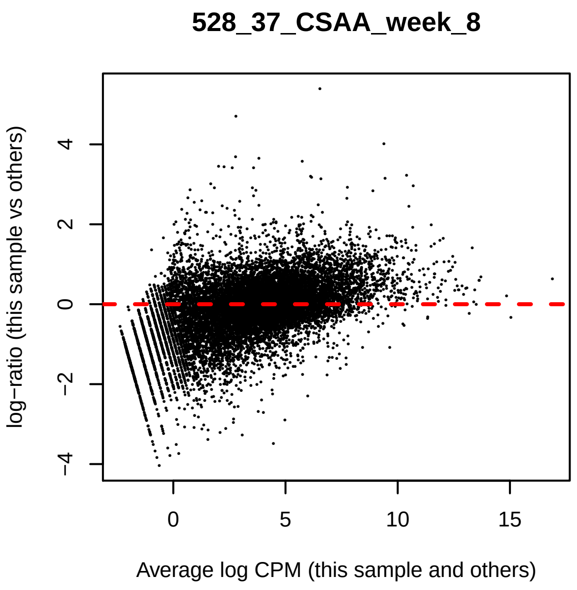
528_37_CSAA_week_8
log−ratio (this sample vs others)
Average log CPM (this sample and others)

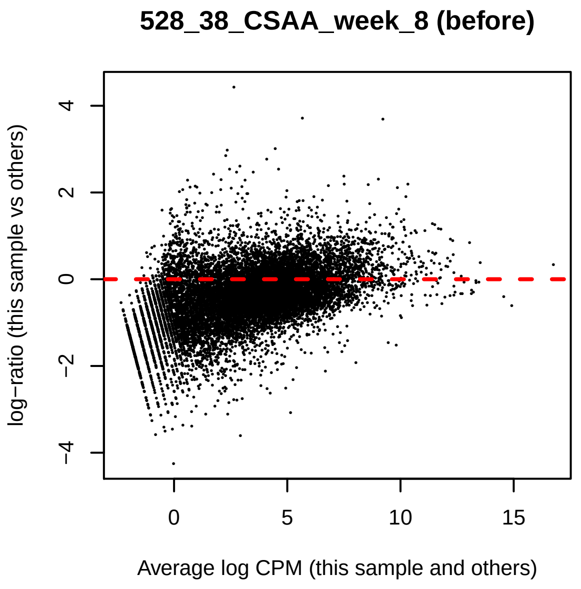
528_38_CSAA_week_8 (before)
log−ratio (this sample vs others)
Average log CPM (this sample and others)

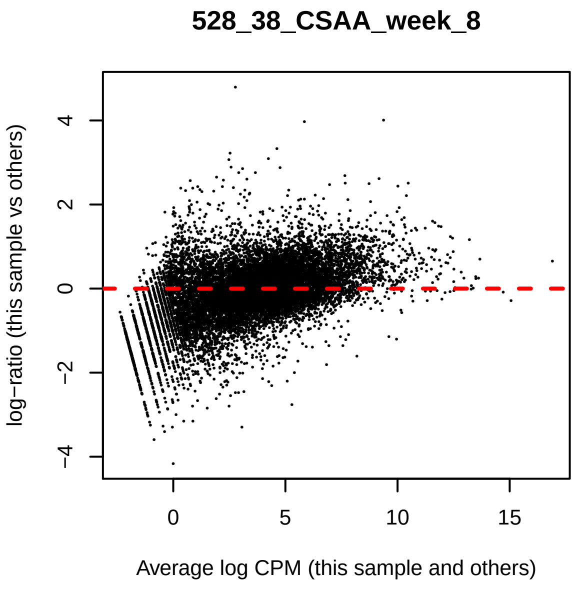
528_38_CSAA_week_8
log−ratio (this sample vs others)
Average log CPM (this sample and others)

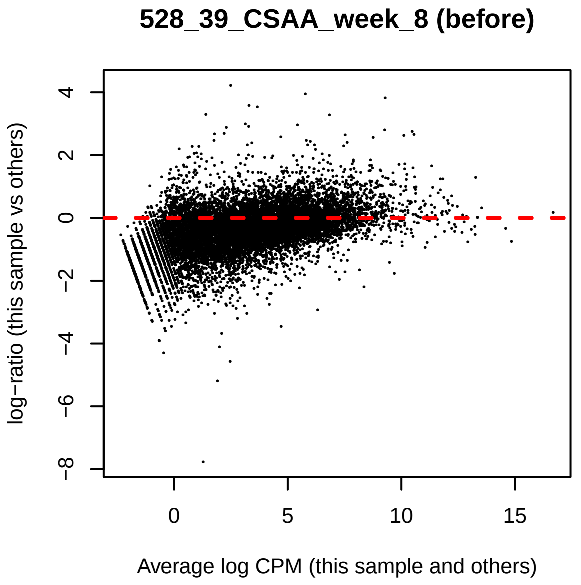
528_39_CSAA_week_8 (before)
log−ratio (this sample vs others)
Average log CPM (this sample and others)

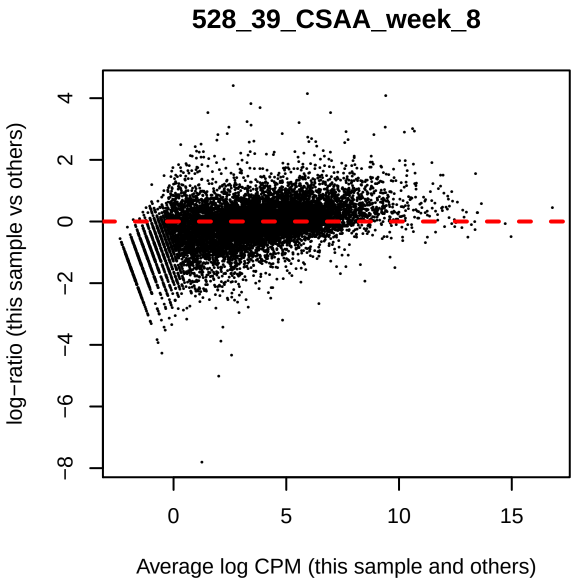
528_39_CSAA_week_8
log−ratio (this sample vs others)
Average log CPM (this sample and others)

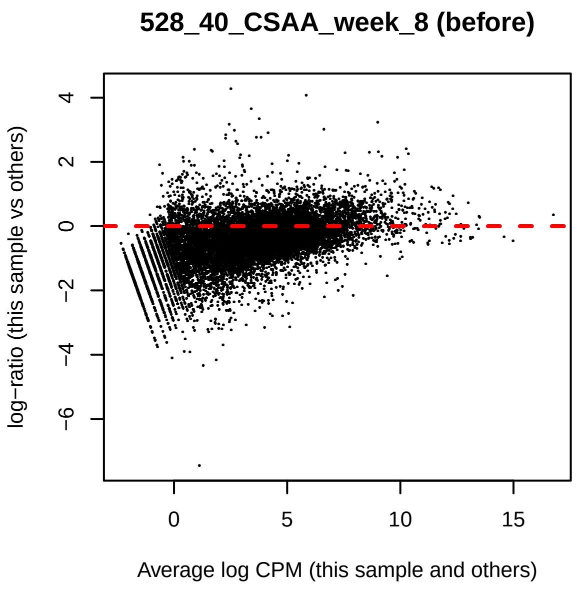
528_40_CSAA_week_8 (before)
log−ratio (this sample vs others)
Average log CPM (this sample and others)

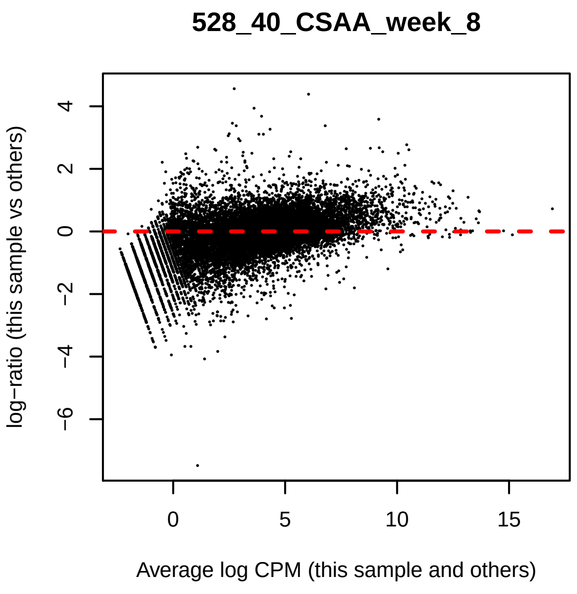
528_40_CSAA_week_8
log−ratio (this sample vs others)
Average log CPM (this sample and others)

528_49_CDDA_Chol_1pc_week_8 (before)

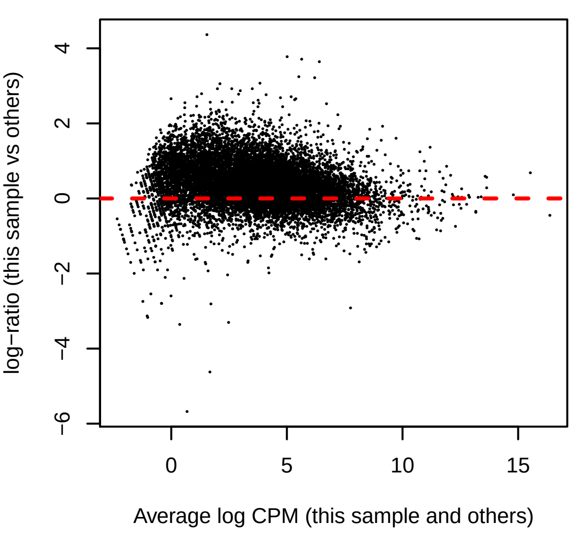

528_49_CDDA_Chol_1pc_week_8

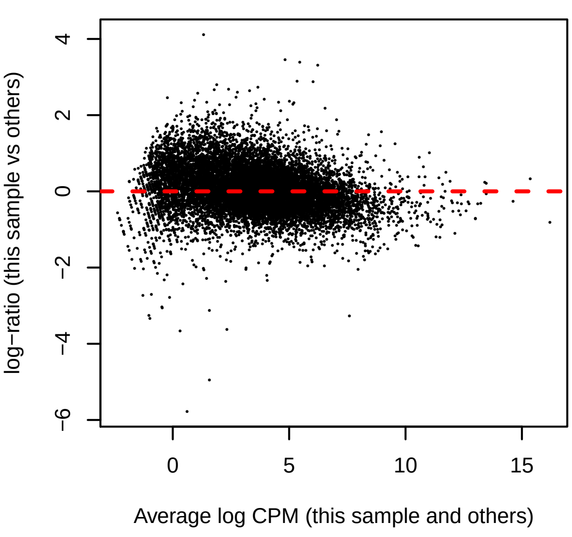

528_50_CDDA_Chol_1pc_week_8 (before)

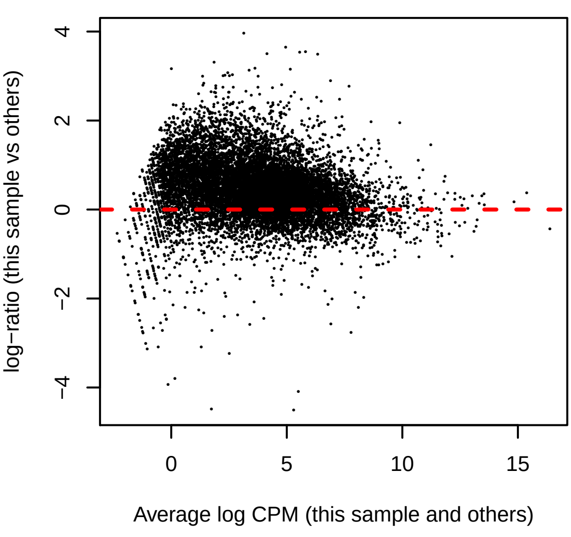

528_50_CDDA_Chol_1pc_week_8

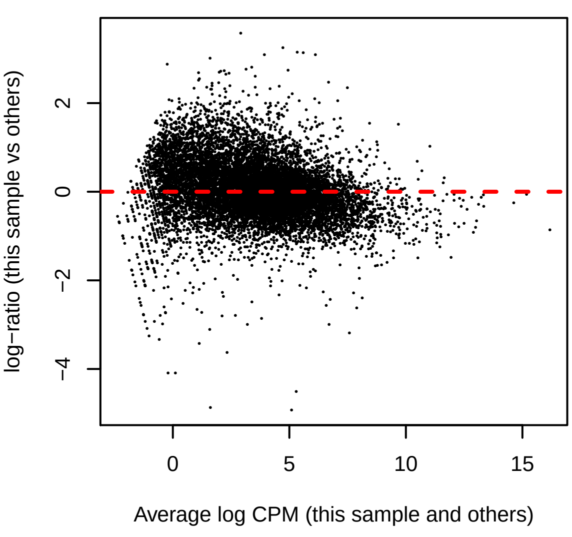

528_51_CDDA_Chol_1pc_week_8 (before)

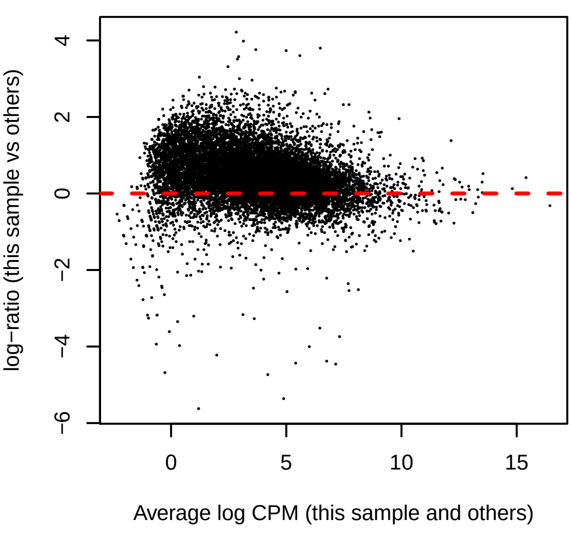

528_51_CDDA_Chol_1pc_week_8

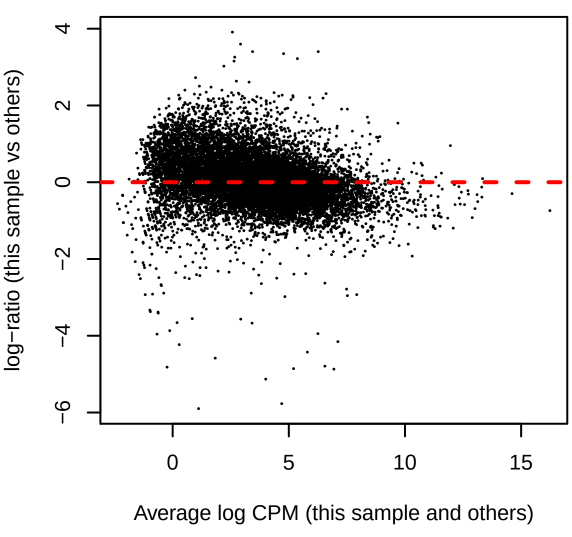

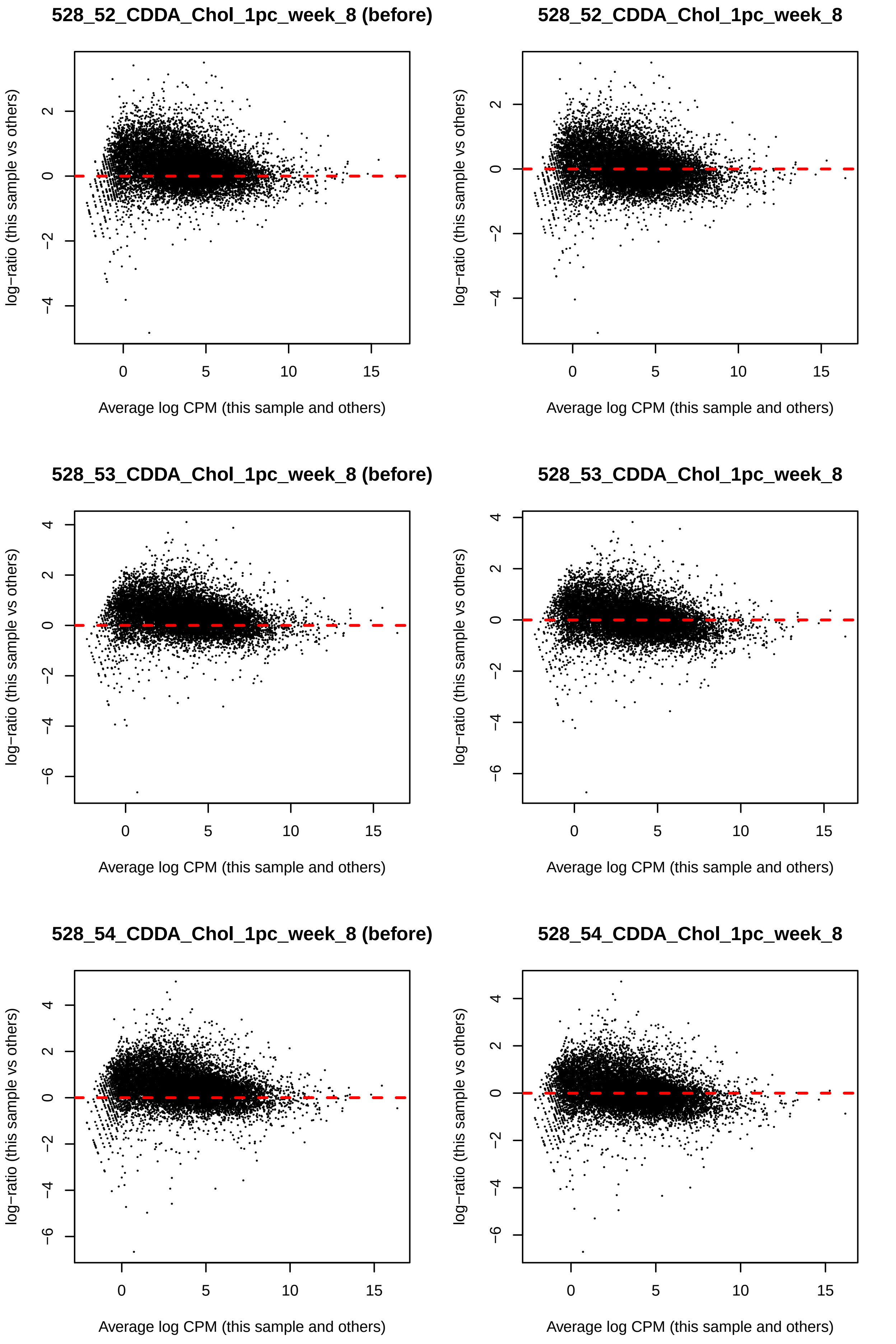
528_52_CDDA_Chol_1pc_week_8 (before)
528_52_CDDA_Chol_1pc_week_8
528_53_CDDA_Chol_1pc_week_8 (before)
528_53_CDDA_Chol_1pc_week_8
528_54_CDDA_Chol_1pc_week_8 (before)
528_54_CDDA_Chol_1pc_week_8
log−ratio (this sample vs others)
Average log CPM (this sample and others)
0
5
10
15
−6
−4
−2
0
2
4

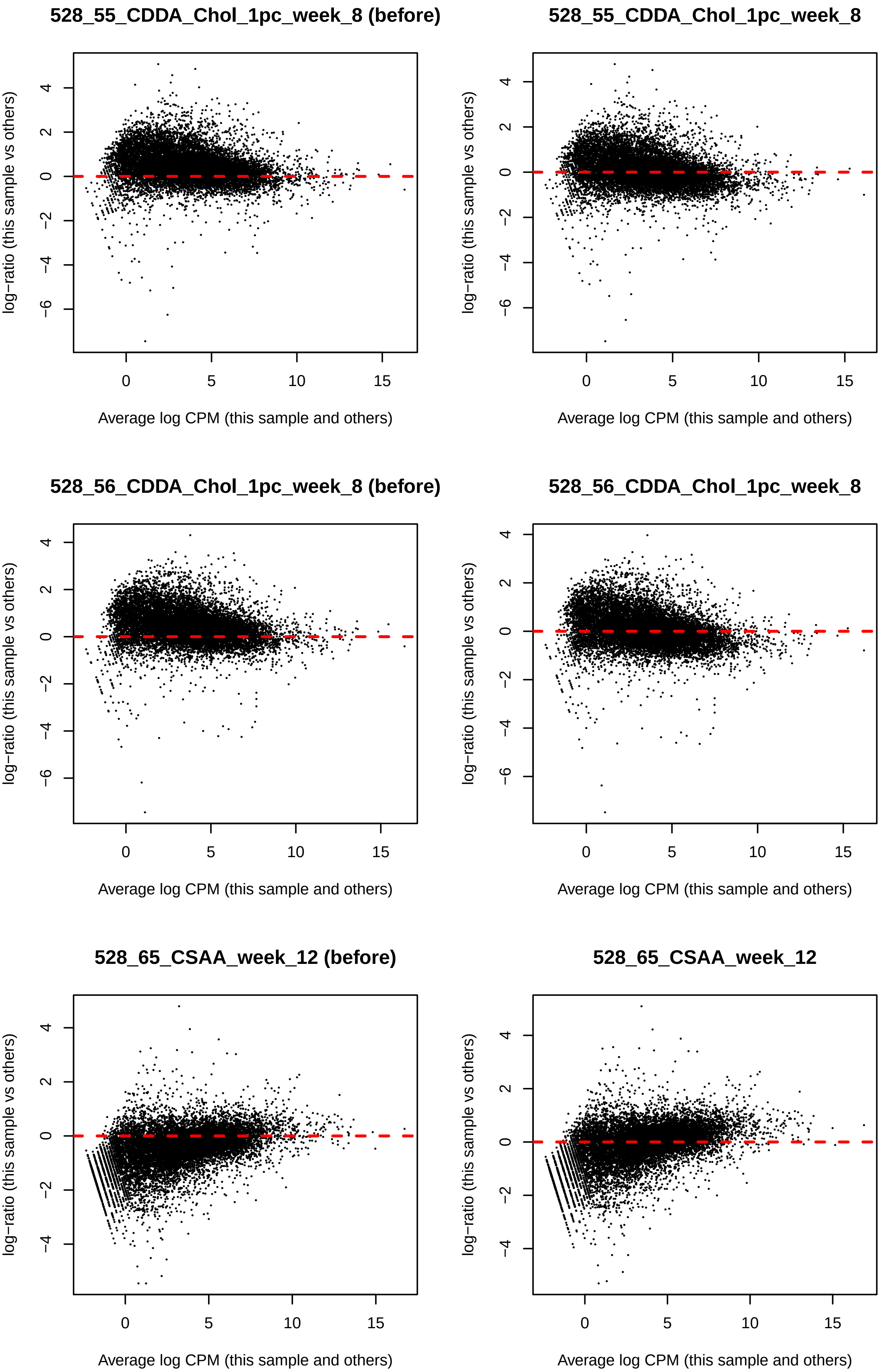
528_55_CDDA_Chol_1pc_week_8 (before)
528_55_CDDA_Chol_1pc_week_8
528_56_CDDA_Chol_1pc_week_8 (before)
528_56_CDDA_Chol_1pc_week_8
528_65_CSAA_week_12 (before)
528_65_CSAA_week_12
log−ratio (this sample vs others)
Average log CPM (this sample and others)
0
5
10
15
4
2
0
−2
−4
−6

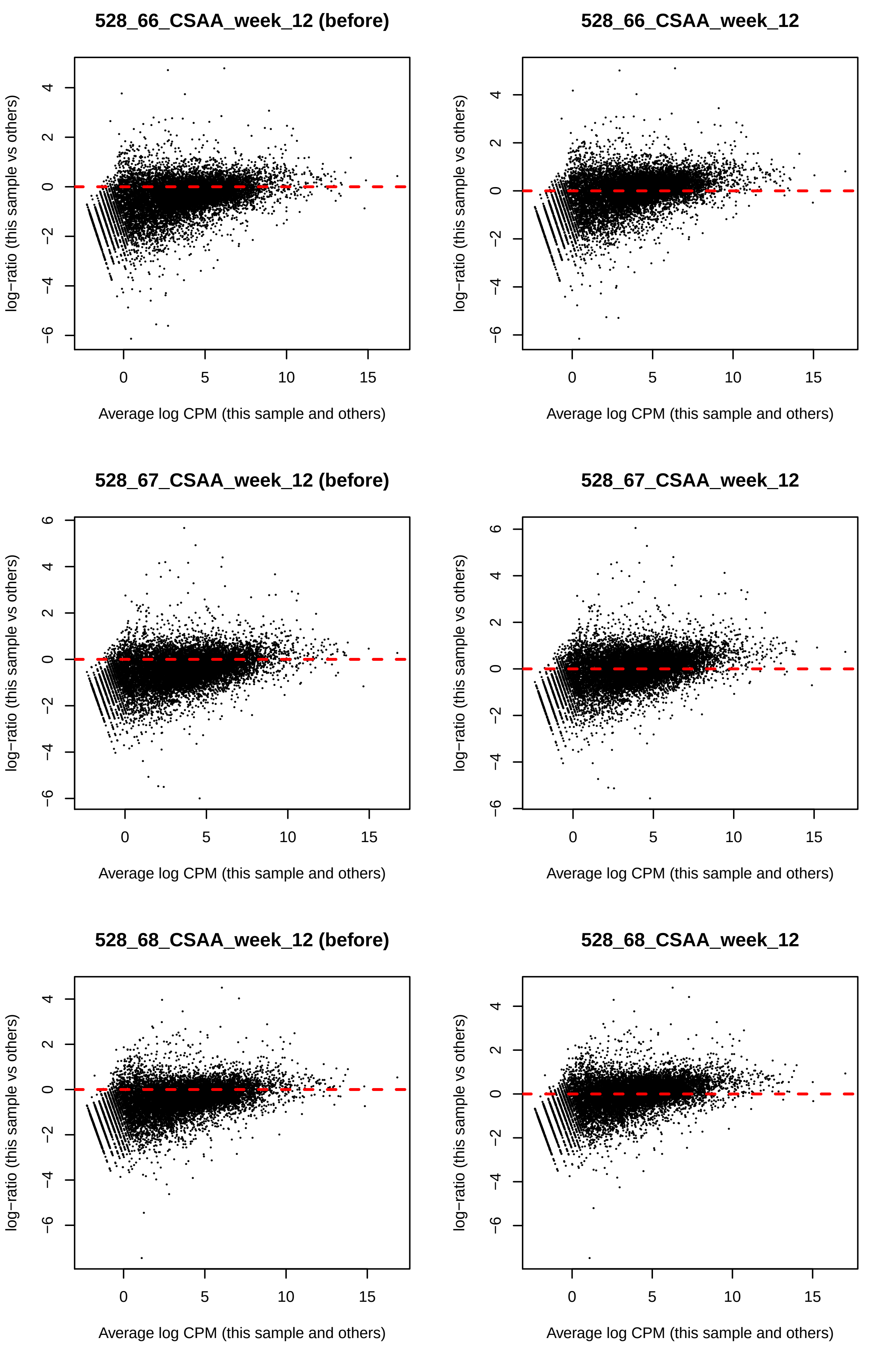
528_66_CSAA_week_12 (before)
528_66_CSAA_week_12
528_67_CSAA_week_12 (before)
528_67_CSAA_week_12
528_68_CSAA_week_12 (before)
528_68_CSAA_week_12
log−ratio (this sample vs others)
Average log CPM (this sample and others)

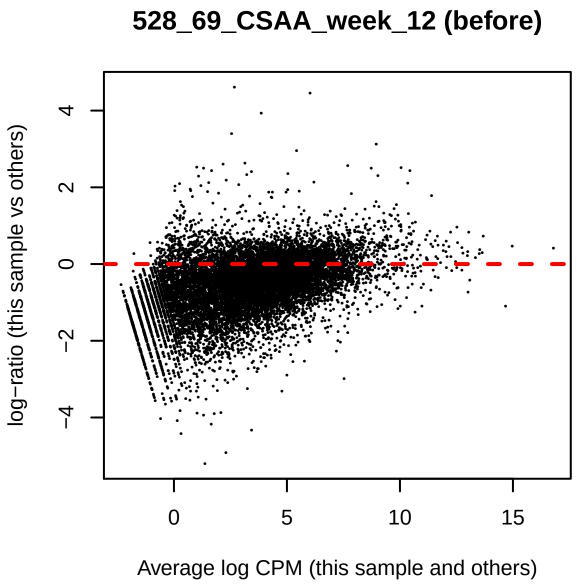
528_69_CSAA_week_12 (before)
log−ratio (this sample vs others)
Average log CPM (this sample and others)
4
2
0
−2
−4
0
5
10
15

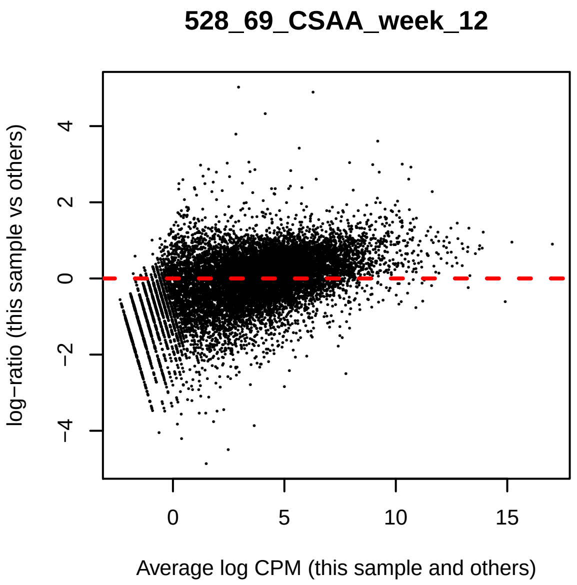
528_69_CSAA_week_12
log−ratio (this sample vs others)
Average log CPM (this sample and others)
4
2
0
−2
−4
0
5
10
15

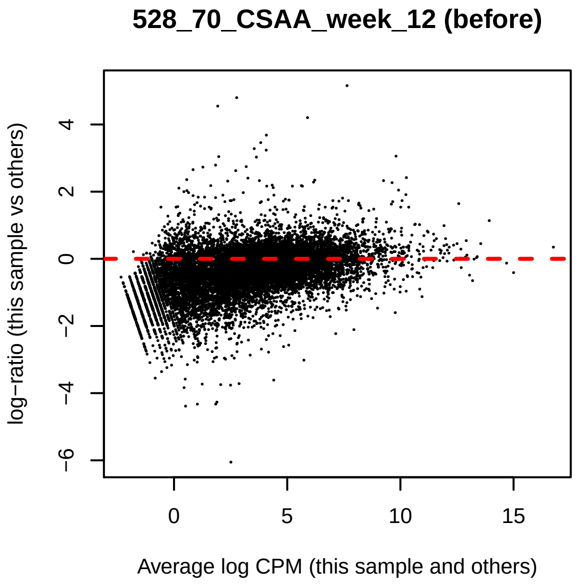
528_70_CSAA_week_12 (before)
log−ratio (this sample vs others)
Average log CPM (this sample and others)
4
2
0
−2
−4
−6
0
5
10
15

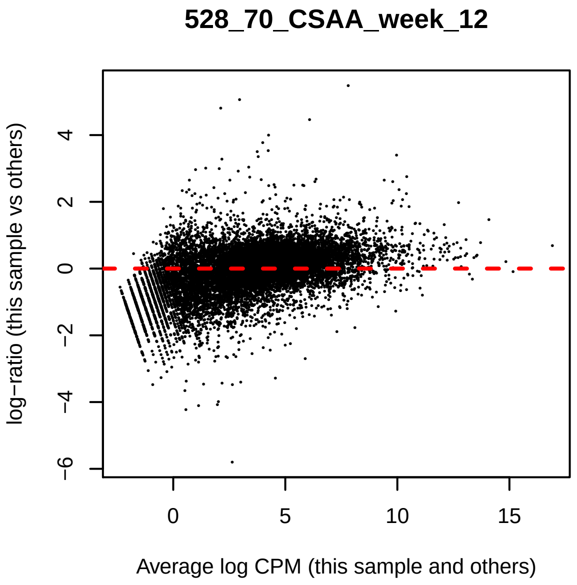
528_70_CSAA_week_12
log−ratio (this sample vs others)
Average log CPM (this sample and others)
4
2
0
−2
−4
−6
0
5
10
15

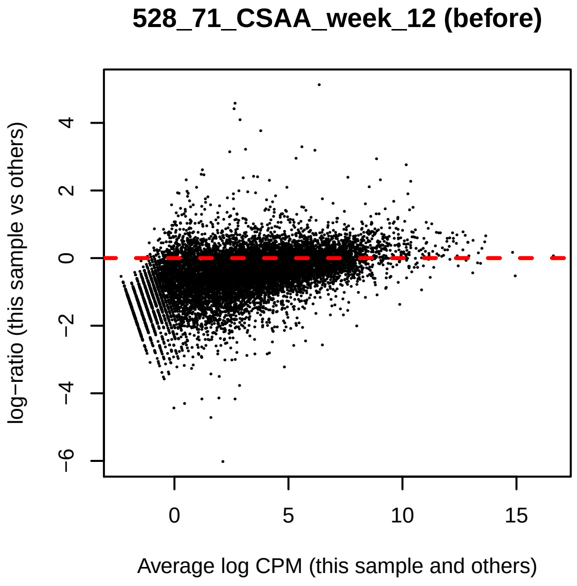
528_71_CSAA_week_12 (before)
log−ratio (this sample vs others)
Average log CPM (this sample and others)
4
2
0
−2
−4
−6
0
5
10
15

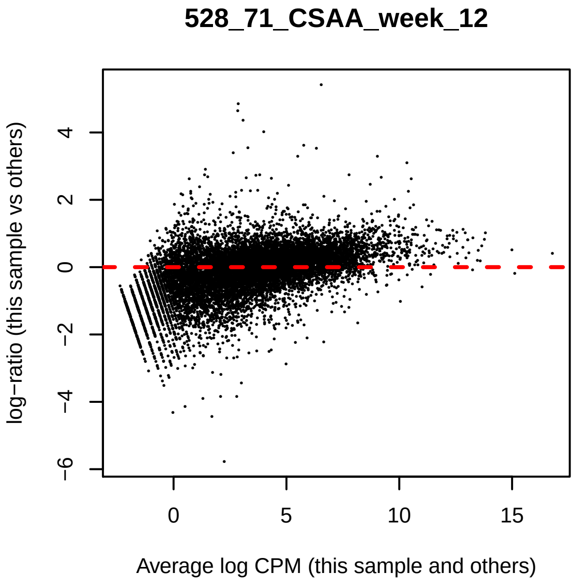
528_71_CSAA_week_12
log−ratio (this sample vs others)
Average log CPM (this sample and others)
4
2
0
−2
−4
−6
0
5
10
15

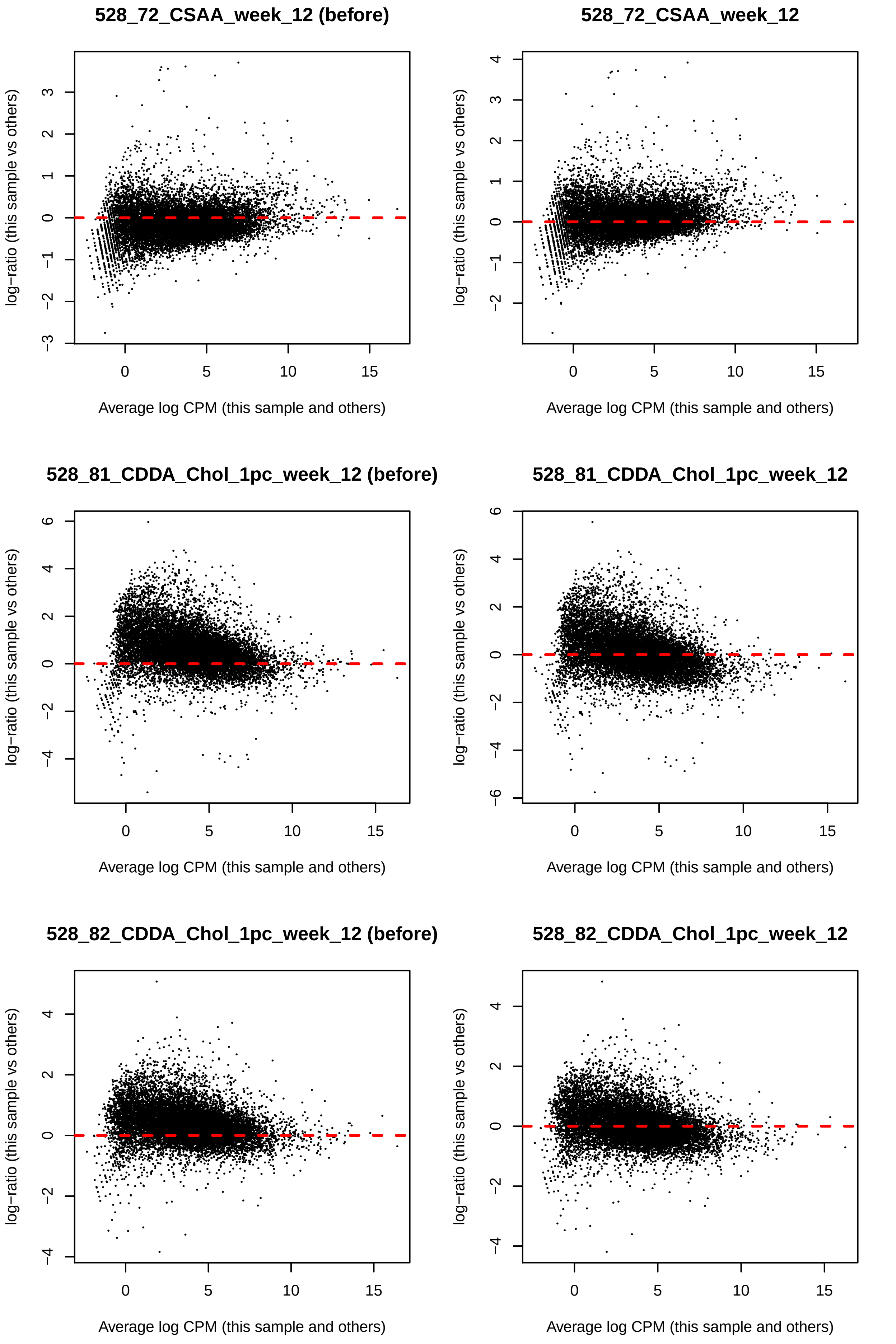

528_72_CSAA_week_12 (before)
528_72_CSAA_week_12
528_81_CDDA_Chol_1pc_week_12 (before)
528_81_CDDA_Chol_1pc_week_12
528_82_CDDA_Chol_1pc_week_12 (before)
528_82_CDDA_Chol_1pc_week_12
log−ratio (this sample vs others)
Average log CPM (this sample and others)

528_83_CDDA_Chol_1pc_week_12 (before)

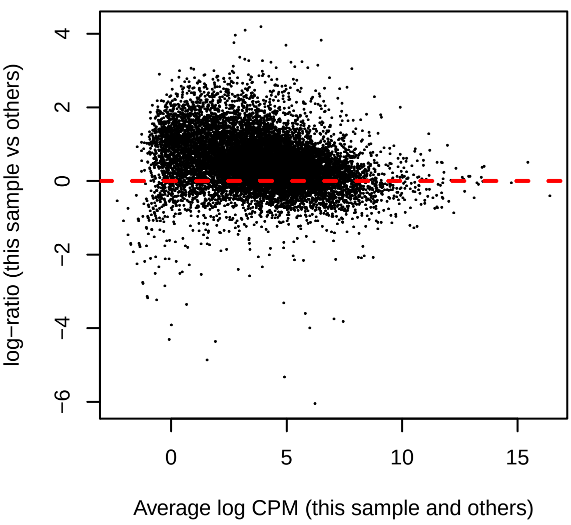

528_83_CDDA_Chol_1pc_week_12

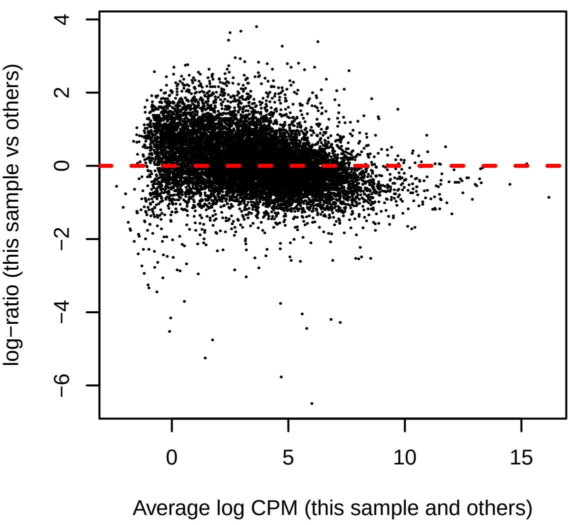

528_84_CDDA_Chol_1pc_week_12 (before)

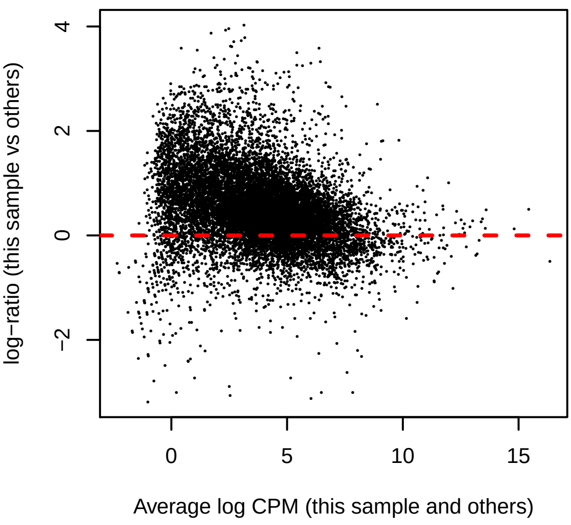

528_84_CDDA_Chol_1pc_week_12

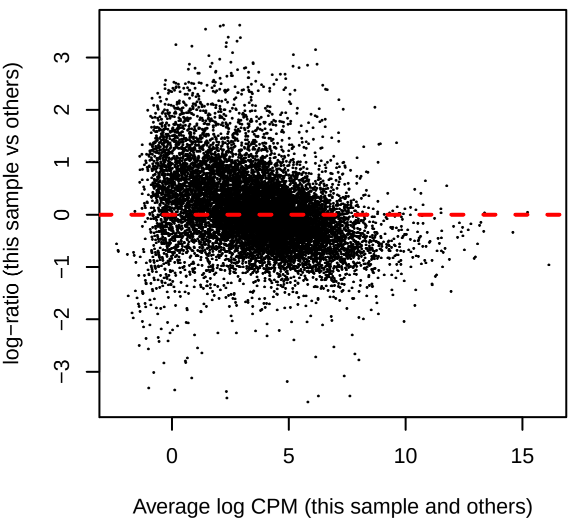

528_85_CDDA_Chol_1pc_week_12 (before)

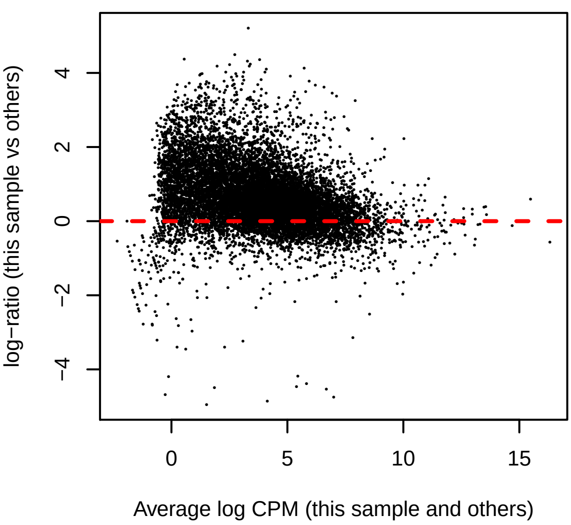

528_85_CDDA_Chol_1pc_week_12

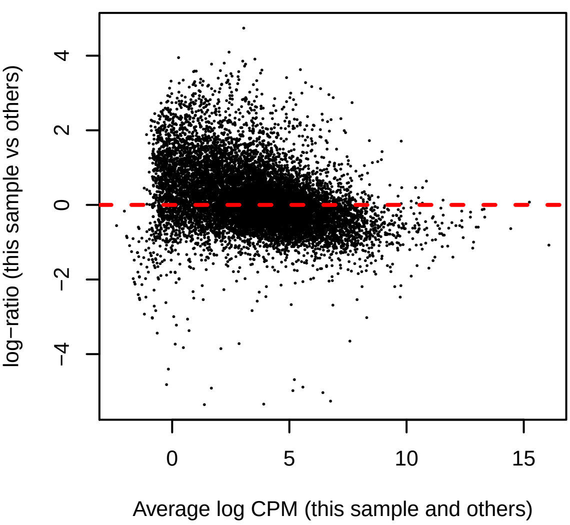

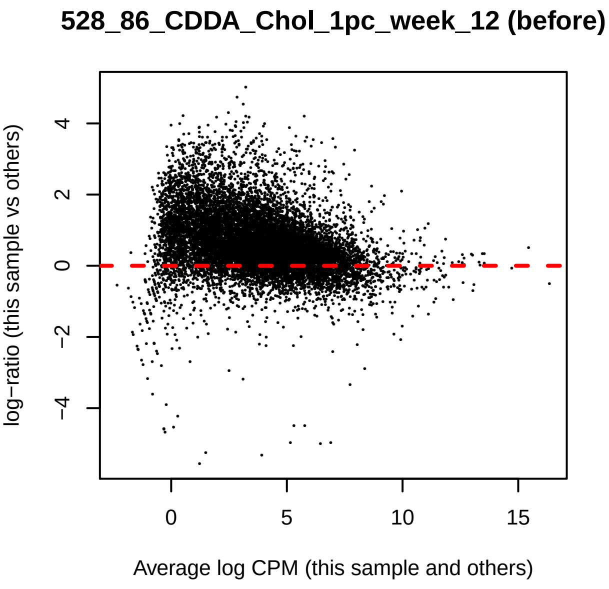
528_86_CDDA_Chol_1pc_week_12 (before)
log−ratio (this sample vs others)
Average log CPM (this sample and others)

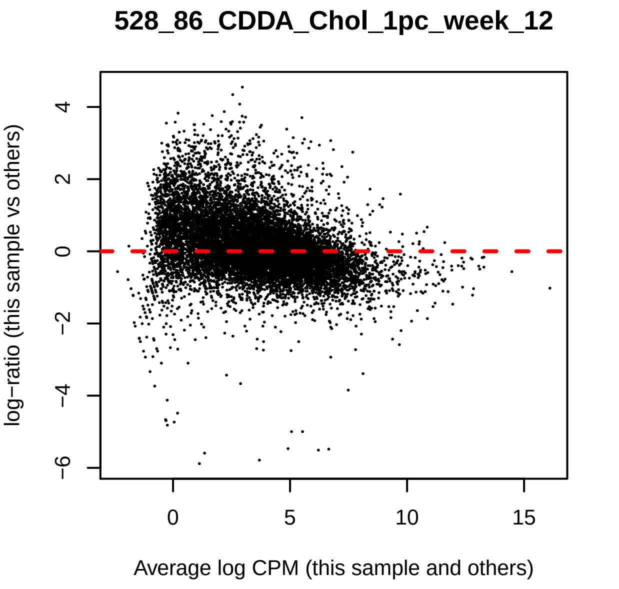
528_86_CDDA_Chol_1pc_week_12
log−ratio (this sample vs others)
Average log CPM (this sample and others)

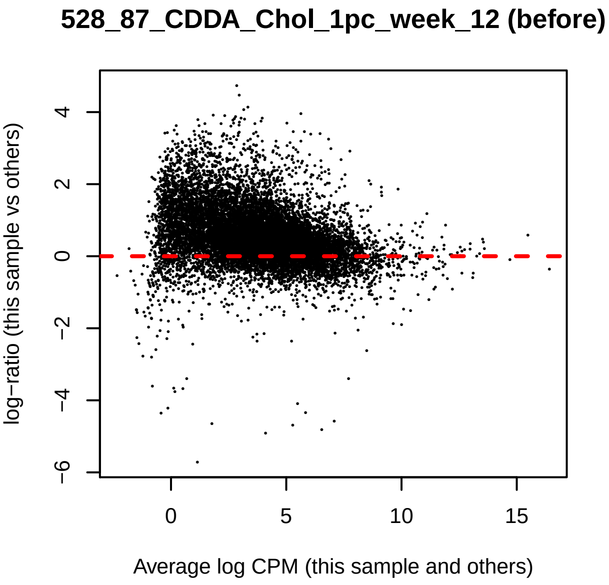
528_87_CDDA_Chol_1pc_week_12 (before)
log−ratio (this sample vs others)
Average log CPM (this sample and others)

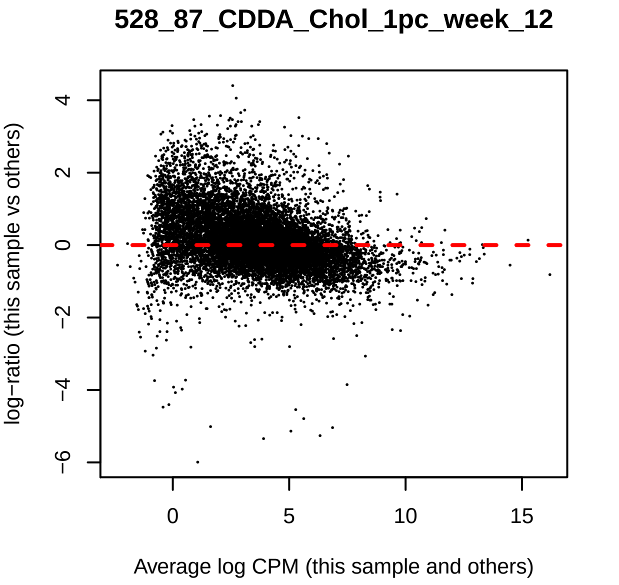
528_87_CDDA_Chol_1pc_week_12
log−ratio (this sample vs others)
Average log CPM (this sample and others)

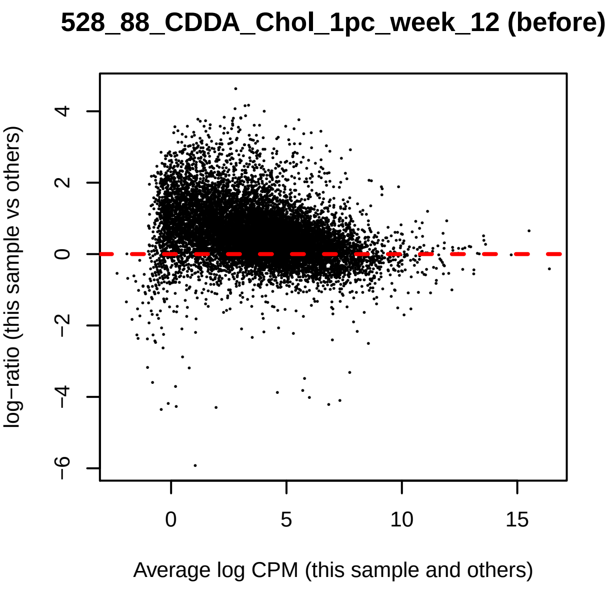
528_88_CDDA_Chol_1pc_week_12 (before)
log−ratio (this sample vs others)
Average log CPM (this sample and others)

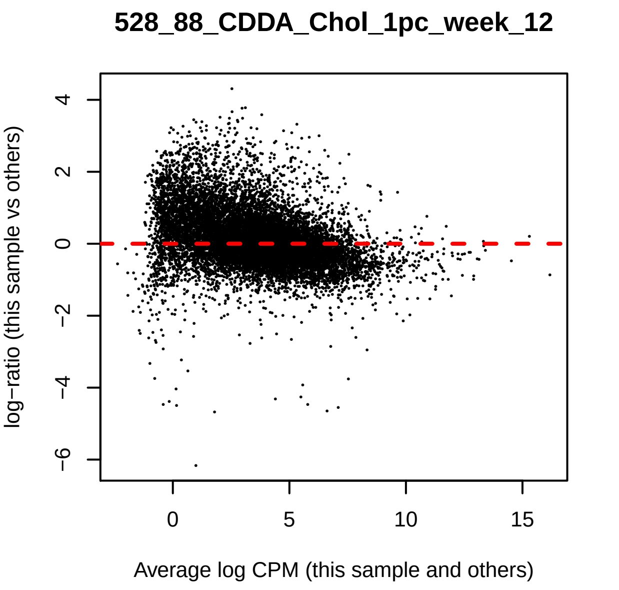
528_88_CDDA_Chol_1pc_week_12
log−ratio (this sample vs others)
Average log CPM (this sample and others)
