# Supplementary material for: GIANT: galaxy-based tool for interactive analysis of transcriptomic data
Source: Sci Rep. 2020 Nov 16;10:19835. doi: 10.1038/s41598-020-76769-w (PMC7670435; doi:10.1038/s41598-020-76769-w)
Supplement: Supplementary file 2 — Supplementary Information. [file 41598_2020_76769_MOESM2_ESM.zip › Tools_InputOutput_Parameters/RNA-seq_workflow/Step1-Normalization_Differential/Outputs/extra_files_path_5/mdsscree.pdf]

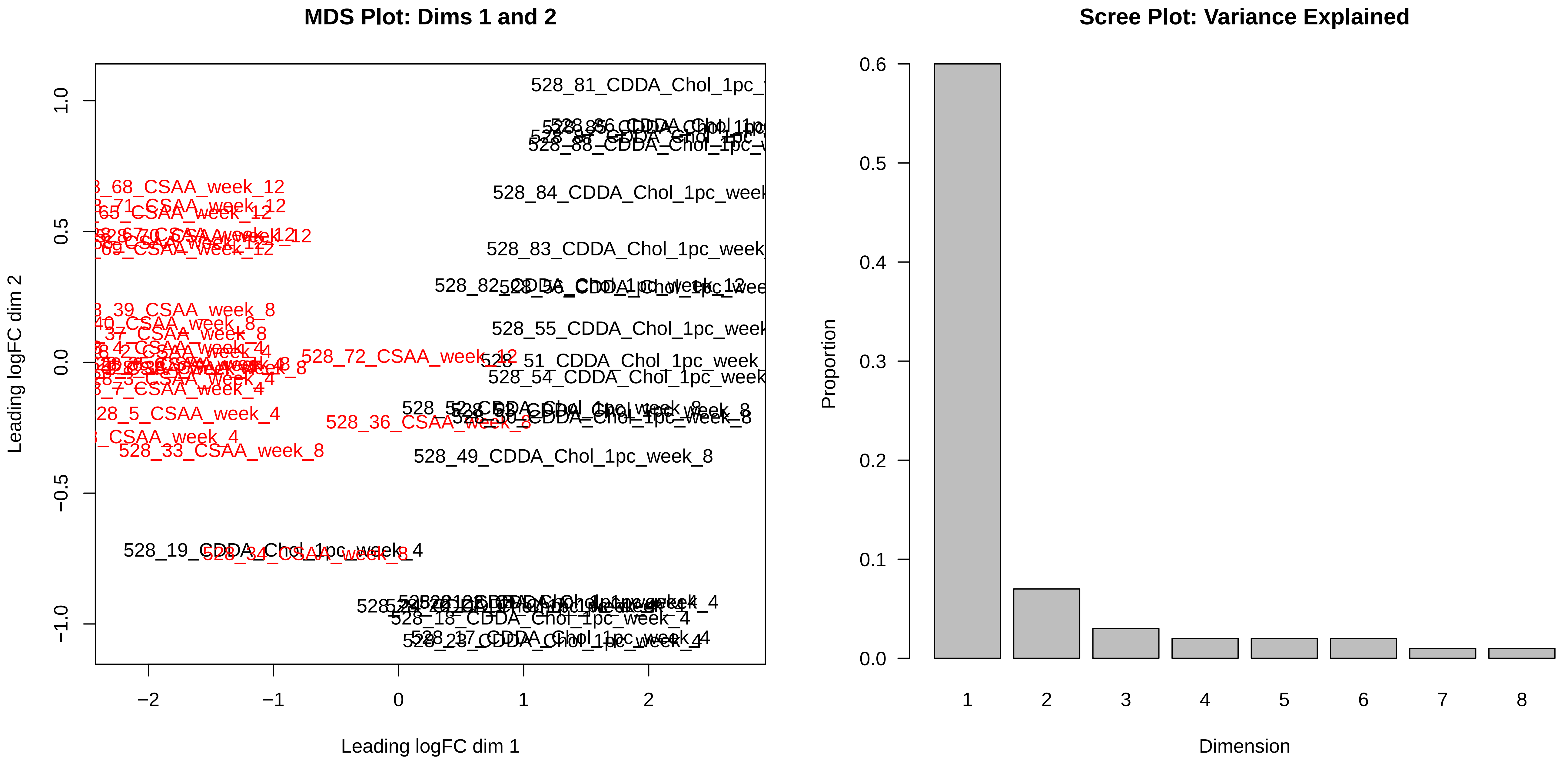
MDS Plot: Dims 1 and 2
Leading logFC dim 2
Leading logFC dim 1
1.0
0.5
0.0
−0.5
−1.0
−2
−1
0
1
2
528_84_CDDA_Chol_1pc_week
528_83_CDDA_Chol_1pc_week
528_55_CDDA_Chol_1pc_week
528_51_CDDA_Chol_1pc_week
528_54_CDDA_Chol_1pc_week
528_49_CDDA_Chol_1pc_week_8
528_72_CSAA_week_12
528_36_CSAA_week_8
528_5_CSAA_week_4
528_33_CSAA_week_8
528_19_CDDA_Chol_1pc_week_4
528_34_CSAA_week_8
528_18_CDDA_Chol_1pc_week_4
Scree Plot: Variance Explained
Proportion
Dimension
0.0
0.1
0.2
0.3
0.4
0.5
0.6
1
2
3
4
5
6
7
8
