# Supplementary material for: GIANT: galaxy-based tool for interactive analysis of transcriptomic data
Source: Sci Rep. 2020 Nov 16;10:19835. doi: 10.1038/s41598-020-76769-w (PMC7670435; doi:10.1038/s41598-020-76769-w)
Supplement: Supplementary file 2 — Supplementary Information. [file 41598_2020_76769_MOESM2_ESM.zip › Tools_InputOutput_Parameters/RNA-seq_workflow/Step1-Normalization_Differential/Outputs/extra_files_path_5/mdsplot_extra.pdf]

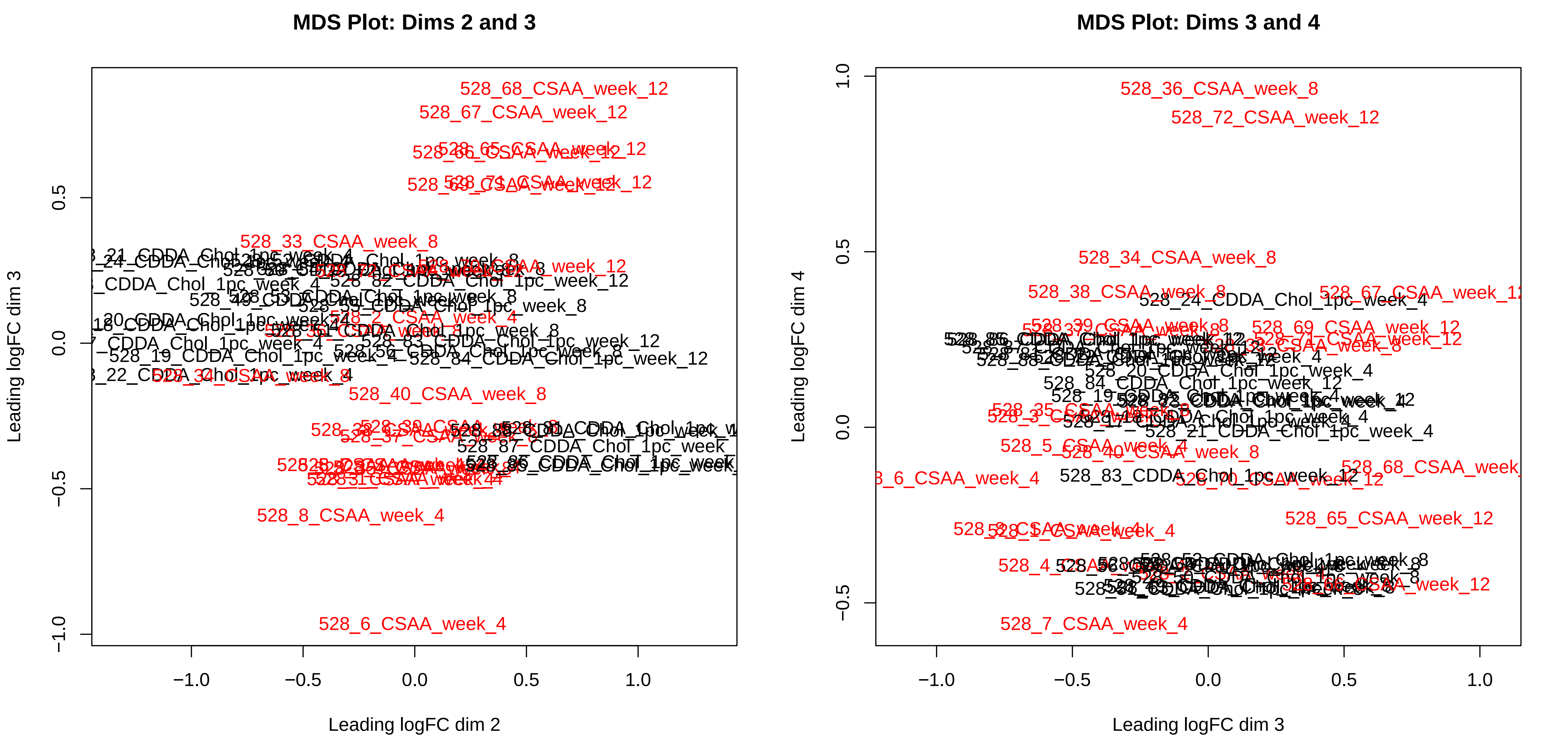
MDS Plot: Dims 2 and 3
Leading logFC dim 2
Leading logFC dim 3
−1.0
−0.5
0.0
0.5
1.0
528_68_CSAA_week_12
528_67_CSAA_week_12
528_33_CSAA_week_8
528_82_CDDA_Chol_1pc_week_12
528_2_CSAA_week_4
528_83_CDDA_Chol_1pc_week_12
528_84_CDDA_Chol_1pc_week_12
528_40_CSAA_week_8
528_8_CSAA_week_4
528_6_CSAA_week_4
MDS Plot: Dims 3 and 4
Leading logFC dim 3
Leading logFC dim 4
528_36_CSAA_week_8
528_72_CSAA_week_12
528_34_CSAA_week_8
528_38_CSAA_week_8
528_24_CDDA_Chol_1pc_week_4
528_69_CSAA_week_12
528_20_CDDA_Chol_1pc_week_4
528_84_CDDA_Chol_1pc_week_12
528_21_CDDA_Chol_1pc_week_4
528_40_CSAA_week_8
528_83_CDDA_Chol_1pc_week_12
528_65_CSAA_week_12
528_7_CSAA_week_4
