# Supplementary material for: GIANT: galaxy-based tool for interactive analysis of transcriptomic data
Source: Sci Rep. 2020 Nov 16;10:19835. doi: 10.1038/s41598-020-76769-w (PMC7670435; doi:10.1038/s41598-020-76769-w)
Supplement: Supplementary file 2 — Supplementary Information. [file 41598_2020_76769_MOESM2_ESM.zip › Tools_InputOutput_Parameters/RNA-seq_workflow/Step1-Normalization_Differential/Outputs/extra_files_path_5/saplot.pdf]

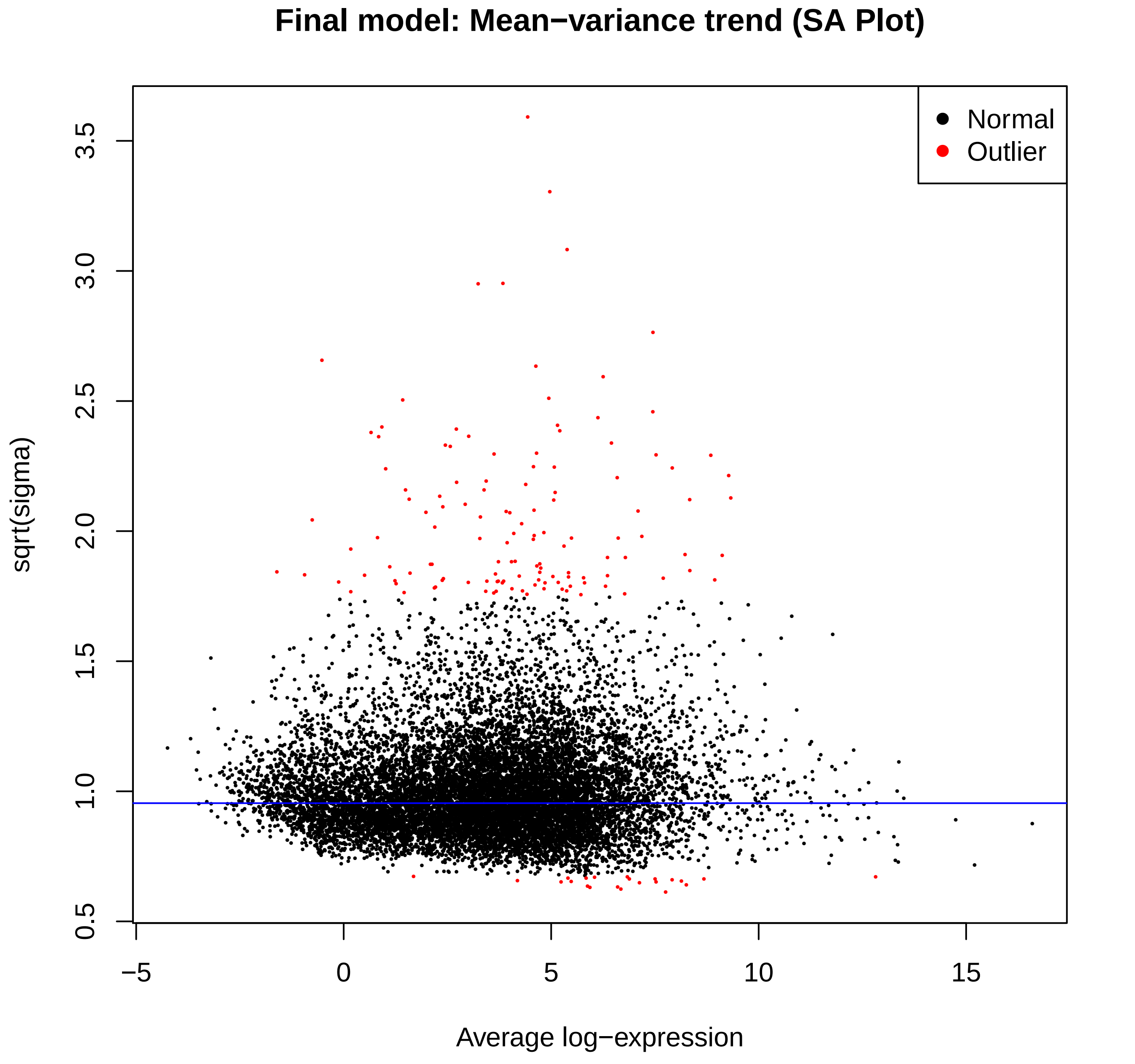
Final model: Mean−variance trend (SA Plot)
Normal
Outlier
sqrt(sigma)
0.5
1.0
1.5
2.0
2.5
3.0
3.5
−5
0
5
10
15
Average log−expression
