# Supplementary material for: GIANT: galaxy-based tool for interactive analysis of transcriptomic data
Source: Sci Rep. 2020 Nov 16;10:19835. doi: 10.1038/s41598-020-76769-w (PMC7670435; doi:10.1038/s41598-020-76769-w)
Supplement: Supplementary file 2 — Supplementary Information. [file 41598_2020_76769_MOESM2_ESM.zip › Tools_InputOutput_Parameters/RNA-seq_workflow/Step3-Heatmap/Outputs/extra_files_path_29/outputClustering.html]

| Gene | Cluster | logFC | adj.P.Val |  |
| --- | --- | --- | --- | --- |
| Gene | Cluster | logFC | adj.P.Val |  |
| --- | --- | --- | --- | --- |
| ENSRNOG00000027811 | 1 | 3.676 | 4.093e-16 |  |
| --- | --- | --- | --- | --- |
| ENSRNOG00000003104 | 2 | 2.641 | 2.102e-14 |  |
| ENSRNOG00000030187 | 1 | 4.684 | 3.095e-14 |  |
| ENSRNOG00000000525 | 3 | 3.275 | 3.882e-14 |  |
| ENSRNOG00000036703 | 1 | 3.373 | 4.396e-14 |  |
| ENSRNOG00000043451 | 4 | 4.668 | 5.848e-14 |  |
| ENSRNOG00000004805 | 5 | 4.197 | 6.408e-14 |  |
| ENSRNOG00000008539 | 1 | 3.063 | 7.748e-14 |  |
| ENSRNOG00000054251 | 1 | 3.016 | 7.783e-14 |  |
| ENSRNOG00000008628 | 2 | 2.738 | 1.038e-13 |  |
| ENSRNOG00000011459 | 1 | 2.619 | 1.17e-13 |  |
| ENSRNOG00000016541 | 4 | 3.314 | 1.246e-13 |  |
| ENSRNOG00000047367 | 3 | 3.605 | 1.246e-13 |  |
| ENSRNOG00000033192 | 1 | 3.422 | 1.25e-13 |  |
| ENSRNOG00000019728 | 2 | 2.666 | 1.33e-13 |  |
| ENSRNOG00000019058 | 1 | 5.256 | 1.33e-13 |  |
| ENSRNOG00000008409 | 1 | 2.261 | 1.367e-13 |  |
| ENSRNOG00000027839 | 1 | 2.139 | 1.753e-13 |  |
| ENSRNOG00000000567 | 1 | 3.315 | 2.065e-13 |  |
| ENSRNOG00000039754 | 2 | 3.181 | 2.548e-13 |  |
| ENSRNOG00000026647 | 1 | 2.498 | 2.797e-13 |  |
| ENSRNOG00000012543 | 2 | 2.375 | 2.973e-13 |  |
| ENSRNOG00000056069 | 2 | 2.351 | 2.999e-13 |  |
| ENSRNOG00000014166 | 1 | 2.831 | 2.999e-13 |  |
| ENSRNOG00000033110 | 2 | 3.73 | 3.11e-13 |  |
| ENSRNOG00000002579 | 2 | 3.397 | 3.183e-13 |  |
| ENSRNOG00000019616 | 1 | 4.03 | 3.264e-13 |  |
| ENSRNOG00000016117 | 1 | 2.904 | 3.972e-13 |  |
| ENSRNOG00000028137 | 1 | 2.674 | 4.247e-13 |  |
| ENSRNOG00000020263 | 3 | 3.999 | 4.266e-13 |  |
| ENSRNOG00000050670 | 5 | 4.167 | 5.365e-13 |  |
| ENSRNOG00000015588 | 3 | 2.998 | 5.488e-13 |  |
| ENSRNOG00000061484 | 1 | 3.028 | 5.974e-13 |  |
| ENSRNOG00000016326 | 3 | 3.657 | 6.438e-13 |  |
| ENSRNOG00000013720 | 1 | 2.622 | 7.536e-13 |  |
| ENSRNOG00000016538 | 1 | 3.4 | 8.027e-13 |  |
| ENSRNOG00000003715 | 3 | 4.247 | 8.617e-13 |  |
| ENSRNOG00000017783 | 2 | 3.578 | 8.617e-13 |  |
| ENSRNOG00000027491 | 3 | 2.98 | 8.617e-13 |  |
| ENSRNOG00000003954 | 2 | 2.199 | 8.617e-13 |  |
| ENSRNOG00000013241 | 4 | 5.603 | 1.128e-12 |  |
| ENSRNOG00000046005 | 1 | 2.702 | 1.188e-12 |  |
| ENSRNOG00000015036 | 1 | 3.057 | 1.227e-12 |  |
| ENSRNOG00000008816 | 4 | 3.894 | 1.363e-12 |  |
| ENSRNOG00000025198 | 3 | 2.848 | 1.394e-12 |  |
| ENSRNOG00000002925 | 1 | 4.962 | 1.394e-12 |  |
| ENSRNOG00000006548 | 3 | 3.804 | 1.503e-12 |  |
| ENSRNOG00000014336 | 2 | 2.457 | 1.585e-12 |  |
| ENSRNOG00000025691 | 1 | 4.012 | 1.782e-12 |  |
| ENSRNOG00000061379 | 1 | 3.603 | 1.805e-12 |  |
| ENSRNOG00000033608 | 1 | 3.888 | 2.138e-12 |  |
| ENSRNOG00000018953 | 2 | 4.179 | 2.138e-12 |  |
| ENSRNOG00000005659 | 2 | 2.447 | 2.138e-12 |  |
| ENSRNOG00000010805 | 1 | 3.904 | 2.142e-12 |  |
| ENSRNOG00000024428 | 2 | 2.45 | 2.155e-12 |  |
| ENSRNOG00000020281 | 2 | 2.817 | 2.155e-12 |  |
| ENSRNOG00000014034 | 2 | 2.806 | 2.317e-12 |  |
| ENSRNOG00000014361 | 3 | 3.752 | 2.52e-12 |  |
| ENSRNOG00000010645 | 4 | 2.783 | 2.668e-12 |  |
| ENSRNOG00000002525 | 3 | 4.02 | 2.782e-12 |  |
| ENSRNOG00000036711 | 2 | 2.166 | 2.878e-12 |  |
| ENSRNOG00000027894 | 3 | 2.799 | 3.128e-12 |  |
| ENSRNOG00000027742 | 1 | 3.529 | 3.128e-12 |  |
| ENSRNOG00000012094 | 1 | 3.85 | 3.234e-12 |  |
| ENSRNOG00000002667 | 1 | 3.114 | 3.276e-12 |  |
| ENSRNOG00000060412 | 5 | 3.989 | 3.972e-12 |  |
| ENSRNOG00000057153 | 1 | 2.914 | 4.071e-12 |  |
| ENSRNOG00000048411 | 3 | 2.613 | 4.071e-12 |  |
| ENSRNOG00000015420 | 2 | 2.351 | 4.992e-12 |  |
| ENSRNOG00000008115 | 2 | 2.279 | 5.178e-12 |  |
| ENSRNOG00000012181 | 4 | 3.174 | 5.275e-12 |  |
| ENSRNOG00000014453 | 4 | 2.704 | 5.474e-12 |  |
| ENSRNOG00000026306 | 5 | 4.243 | 5.507e-12 |  |
| ENSRNOG00000026295 | 5 | 4.208 | 5.571e-12 |  |
| ENSRNOG00000014532 | 4 | 2.731 | 5.631e-12 |  |
| ENSRNOG00000002730 | 1 | 2.311 | 5.636e-12 |  |
| ENSRNOG00000009687 | 2 | 2.389 | 6.038e-12 |  |
| ENSRNOG00000016866 | 2 | 2.933 | 6.075e-12 |  |
| ENSRNOG00000038916 | 2 | 2.149 | 6.292e-12 |  |
| ENSRNOG00000036728 | 3 | 3.837 | 6.393e-12 |  |
| ENSRNOG00000006631 | 5 | 3.503 | 7.143e-12 |  |
| ENSRNOG00000053272 | 3 | 2.701 | 7.196e-12 |  |
| ENSRNOG00000001466 | 4 | 5.606 | 7.322e-12 |  |
| ENSRNOG00000061316 | 3 | 2.415 | 7.322e-12 |  |
| ENSRNOG00000005960 | 3 | 3.244 | 7.459e-12 |  |
| ENSRNOG00000021062 | 1 | 2.258 | 8.97e-12 |  |
| ENSRNOG00000005574 | 3 | 3.904 | 1.049e-11 |  |
| ENSRNOG00000003703 | 2 | 2.015 | 1.049e-11 |  |
| ENSRNOG00000010362 | 4 | 2.917 | 1.12e-11 |  |
| ENSRNOG00000011977 | 3 | 2.524 | 1.17e-11 |  |
| ENSRNOG00000000142 | 2 | 3.051 | 1.201e-11 |  |
| ENSRNOG00000003897 | 4 | 4.068 | 1.212e-11 |  |
| ENSRNOG00000010997 | 1 | 2.852 | 1.212e-11 |  |
| ENSRNOG00000048961 | 6 | 3.628 | 1.212e-11 |  |
| ENSRNOG00000010721 | 3 | 2.47 | 1.212e-11 |  |
| ENSRNOG00000001158 | 1 | 1.981 | 1.223e-11 |  |
| ENSRNOG00000006094 | 1 | 3.144 | 1.249e-11 |  |
| ENSRNOG00000002659 | 2 | 2.017 | 1.249e-11 |  |
| ENSRNOG00000012083 | 3 | 3.121 | 1.382e-11 |  |
| ENSRNOG00000013895 | 1 | 2.53 | 1.469e-11 |  |
| ENSRNOG00000015941 | 2 | 2.797 | 1.477e-11 |  |
| ENSRNOG00000058039 | 1 | 2.639 | 1.651e-11 |  |
| ENSRNOG00000042950 | 3 | 4.973 | 1.726e-11 |  |
| ENSRNOG00000001270 | 3 | 2.481 | 1.801e-11 |  |
| ENSRNOG00000019202 | 1 | 2.839 | 1.965e-11 |  |
| ENSRNOG00000001527 | 5 | 2.868 | 1.976e-11 |  |
| ENSRNOG00000019138 | 2 | 3.009 | 1.98e-11 |  |
| ENSRNOG00000021176 | 3 | 2.131 | 2e-11 |  |
| ENSRNOG00000020310 | 3 | 3.533 | 2e-11 |  |
| ENSRNOG00000007302 | 4 | 2.692 | 2.001e-11 |  |
| ENSRNOG00000008947 | 1 | 3.144 | 2.089e-11 |  |
| ENSRNOG00000007805 | 5 | 2.909 | 2.104e-11 |  |
| ENSRNOG00000023428 | 2 | 2.412 | 2.104e-11 |  |
| ENSRNOG00000021896 | 3 | 5.21 | 2.104e-11 |  |
| ENSRNOG00000009339 | 3 | 2.787 | 2.128e-11 |  |
| ENSRNOG00000016695 | 1 | 2.771 | 2.337e-11 |  |
| ENSRNOG00000053047 | 1 | 2.554 | 2.37e-11 |  |
| ENSRNOG00000049361 | 3 | 3.247 | 2.459e-11 |  |
| ENSRNOG00000053691 | 2 | 3.8 | 2.5e-11 |  |
| ENSRNOG00000019100 | 3 | 2.664 | 2.634e-11 |  |
| ENSRNOG00000016810 | 1 | 2.069 | 2.701e-11 |  |
| ENSRNOG00000013967 | 2 | 3.052 | 2.902e-11 |  |
| ENSRNOG00000023628 | 1 | 2.104 | 2.95e-11 |  |
| ENSRNOG00000011541 | 1 | 2.965 | 2.95e-11 |  |
| ENSRNOG00000002843 | 3 | 3.958 | 3.214e-11 |  |
| ENSRNOG00000024728 | 3 | 2.57 | 3.284e-11 |  |
| ENSRNOG00000007377 | 3 | 3.48 | 3.357e-11 |  |
| ENSRNOG00000013578 | 3 | 4.821 | 3.444e-11 |  |
| ENSRNOG00000003832 | 6 | 3.447 | 3.754e-11 |  |
| ENSRNOG00000011774 | 2 | 3.98 | 3.927e-11 |  |
| ENSRNOG00000049033 | 3 | 3.298 | 3.968e-11 |  |
| ENSRNOG00000038035 | 3 | 2.634 | 4.011e-11 |  |
| ENSRNOG00000003895 | 2 | 3.269 | 4.068e-11 |  |
| ENSRNOG00000037931 | 5 | 2.895 | 4.173e-11 |  |
| ENSRNOG00000010841 | 3 | 2.619 | 4.28e-11 |  |
| ENSRNOG00000015514 | 2 | 4.324 | 4.513e-11 |  |
| ENSRNOG00000014776 | 3 | 2.255 | 4.733e-11 |  |
| ENSRNOG00000004516 | 2 | 2.614 | 4.953e-11 |  |
| ENSRNOG00000018371 | 1 | 2.976 | 5.142e-11 |  |
| ENSRNOG00000020587 | 2 | 2.601 | 5.419e-11 |  |
| ENSRNOG00000002948 | 1 | 3.21 | 5.453e-11 |  |
| ENSRNOG00000023851 | 5 | 3.773 | 5.463e-11 |  |
| ENSRNOG00000003866 | 2 | 2.267 | 5.758e-11 |  |
| ENSRNOG00000024365 | 2 | 2.407 | 5.823e-11 |  |
| ENSRNOG00000017932 | 2 | 2.276 | 5.823e-11 |  |
| ENSRNOG00000019477 | 3 | 2.954 | 6.166e-11 |  |
| ENSRNOG00000005126 | 2 | 2.522 | 6.291e-11 |  |
| ENSRNOG00000020792 | 6 | 4.074 | 6.291e-11 |  |
| ENSRNOG00000032922 | 2 | 2.999 | 6.31e-11 |  |
| ENSRNOG00000008676 | 1 | 2.498 | 6.452e-11 |  |
| ENSRNOG00000018651 | 2 | 2.312 | 6.477e-11 |  |
| ENSRNOG00000003587 | 2 | 2.954 | 7.06e-11 |  |
| ENSRNOG00000006956 | 5 | 3.163 | 7.139e-11 |  |
| ENSRNOG00000010208 | 1 | 2.475 | 7.166e-11 |  |
| ENSRNOG00000002216 | 2 | 2.108 | 7.329e-11 |  |
| ENSRNOG00000025001 | 1 | 2.561 | 7.445e-11 |  |
| ENSRNOG00000014137 | 2 | 3.597 | 7.445e-11 |  |
| ENSRNOG00000025094 | 3 | 2.849 | 7.593e-11 |  |
| ENSRNOG00000006076 | 3 | 2.956 | 7.608e-11 |  |
| ENSRNOG00000011517 | 1 | 2.801 | 7.733e-11 |  |
| ENSRNOG00000011427 | 5 | 2.966 | 7.879e-11 |  |
| ENSRNOG00000024945 | 3 | 1.949 | 8.063e-11 |  |
| ENSRNOG00000012051 | 3 | 2.836 | 8.088e-11 |  |
| ENSRNOG00000014398 | 3 | 2.695 | 8.173e-11 |  |
| ENSRNOG00000024650 | 3 | 2.996 | 8.352e-11 |  |
| ENSRNOG00000030183 | 1 | 3.398 | 8.359e-11 |  |
| ENSRNOG00000033680 | 2 | 3.532 | 8.359e-11 |  |
| ENSRNOG00000012442 | 2 | 3.219 | 8.533e-11 |  |
| ENSRNOG00000054033 | 5 | 2.861 | 8.667e-11 |  |
| ENSRNOG00000015902 | 3 | 3.917 | 8.685e-11 |  |
| ENSRNOG00000058003 | 3 | 2.416 | 8.878e-11 |  |
| ENSRNOG00000043093 | 5 | 3.487 | 8.932e-11 |  |
| ENSRNOG00000010017 | 2 | 2.03 | 8.932e-11 |  |
| ENSRNOG00000019648 | 4 | 2.763 | 8.932e-11 |  |
| ENSRNOG00000006997 | 4 | 2.124 | 8.996e-11 |  |
| ENSRNOG00000005670 | 2 | 2.498 | 9.288e-11 |  |
| ENSRNOG00000015366 | 1 | 3.69 | 9.306e-11 |  |
| ENSRNOG00000007080 | 2 | 3.608 | 9.306e-11 |  |
| ENSRNOG00000002403 | 2 | 3.091 | 9.306e-11 |  |
| ENSRNOG00000010450 | 6 | 3.495 | 9.6e-11 |  |
| ENSRNOG00000047800 | 2 | 2.15 | 9.822e-11 |  |
| ENSRNOG00000060179 | 2 | 2.881 | 9.899e-11 |  |
| ENSRNOG00000015880 | 2 | 2.275 | 9.899e-11 |  |
| ENSRNOG00000007906 | 3 | 2.565 | 1.009e-10 |  |
| ENSRNOG00000016687 | 3 | 4.111 | 1.014e-10 |  |
| ENSRNOG00000000614 | 1 | 3.321 | 1.017e-10 |  |
| ENSRNOG00000014746 | 3 | 2.876 | 1.026e-10 |  |
| ENSRNOG00000042445 | 3 | 5.643 | 1.055e-10 |  |
| ENSRNOG00000012640 | 1 | 2.086 | 1.072e-10 |  |
| ENSRNOG00000016366 | 2 | 2.318 | 1.087e-10 |  |
| ENSRNOG00000014080 | 3 | 2.55 | 1.118e-10 |  |
| ENSRNOG00000016758 | 2 | 2.21 | 1.133e-10 |  |
| ENSRNOG00000006557 | 1 | 2.852 | 1.144e-10 |  |
| ENSRNOG00000020032 | 2 | 2.532 | 1.182e-10 |  |
| ENSRNOG00000018214 | 1 | 3.952 | 1.246e-10 |  |
| ENSRNOG00000013166 | 2 | 2.863 | 1.305e-10 |  |
| ENSRNOG00000013598 | 5 | 2.627 | 1.365e-10 |  |
| ENSRNOG00000033665 | 5 | 3.594 | 1.396e-10 |  |
| ENSRNOG00000013057 | 2 | 2.84 | 1.446e-10 |  |
| ENSRNOG00000008323 | 3 | 3.177 | 1.456e-10 |  |
| ENSRNOG00000005479 | 2 | 3.016 | 1.472e-10 |  |
| ENSRNOG00000002886 | 1 | 3.021 | 1.473e-10 |  |
| ENSRNOG00000020129 | 2 | 3.789 | 1.473e-10 |  |
| ENSRNOG00000026848 | 3 | 4.073 | 1.473e-10 |  |
| ENSRNOG00000022764 | 3 | 2.252 | 1.51e-10 |  |
| ENSRNOG00000000433 | 3 | 2.753 | 1.559e-10 |  |
| ENSRNOG00000033235 | 2 | 2.523 | 1.63e-10 |  |
| ENSRNOG00000005956 | 3 | 3.071 | 1.632e-10 |  |
| ENSRNOG00000011329 | 1 | 2.223 | 1.658e-10 |  |
| ENSRNOG00000055103 | 3 | 4.025 | 1.695e-10 |  |
| ENSRNOG00000008680 | 2 | 3.78 | 1.75e-10 |  |
| ENSRNOG00000028016 | 3 | 2.101 | 1.75e-10 |  |
| ENSRNOG00000030012 | 3 | 2.321 | 1.789e-10 |  |
| ENSRNOG00000013668 | 1 | 2.171 | 1.926e-10 |  |
| ENSRNOG00000002041 | 3 | 3.173 | 1.927e-10 |  |
| ENSRNOG00000016408 | 2 | 2.222 | 1.992e-10 |  |
| ENSRNOG00000027976 | 6 | 4.099 | 2.022e-10 |  |
| ENSRNOG00000056219 | 1 | 4.262 | 2.04e-10 |  |
| ENSRNOG00000026059 | 5 | 3.326 | 2.043e-10 |  |
| ENSRNOG00000003388 | 3 | 2.65 | 2.067e-10 |  |
| ENSRNOG00000033090 | 1 | 2.347 | 2.104e-10 |  |
| ENSRNOG00000036726 | 3 | 3.197 | 2.175e-10 |  |
| ENSRNOG00000031890 | 3 | 4.45 | 2.175e-10 |  |
| ENSRNOG00000002461 | 4 | 2.162 | 2.239e-10 |  |
| ENSRNOG00000042330 | 3 | 5.175 | 2.278e-10 |  |
| ENSRNOG00000007300 | 3 | 2.582 | 2.368e-10 |  |
| ENSRNOG00000004111 | 2 | 2.024 | 2.393e-10 |  |
| ENSRNOG00000034290 | 1 | 3.408 | 2.395e-10 |  |
| ENSRNOG00000014783 | 2 | 2.093 | 2.438e-10 |  |
| ENSRNOG00000000632 | 2 | 2.426 | 2.445e-10 |  |
| ENSRNOG00000005580 | 2 | 2.879 | 2.491e-10 |  |
| ENSRNOG00000018735 | 4 | 1.999 | 2.562e-10 |  |
| ENSRNOG00000003357 | 4 | 2.473 | 2.562e-10 |  |
| ENSRNOG00000012109 | 2 | 2.02 | 2.588e-10 |  |
| ENSRNOG00000023148 | 5 | 4.178 | 2.594e-10 |  |
| ENSRNOG00000006591 | 7 | 3.519 | 2.654e-10 |  |
| ENSRNOG00000046803 | 5 | 3.784 | 2.793e-10 |  |
| ENSRNOG00000034038 | 1 | 3.252 | 2.836e-10 |  |
| ENSRNOG00000007484 | 3 | 3.313 | 2.86e-10 |  |
| ENSRNOG00000016623 | 5 | 3.147 | 2.9e-10 |  |
| ENSRNOG00000005903 | 2 | 2.433 | 2.928e-10 |  |
| ENSRNOG00000008182 | 3 | 3.566 | 2.981e-10 |  |
| ENSRNOG00000009037 | 2 | 3.258 | 3.044e-10 |  |
| ENSRNOG00000020539 | 3 | 2.88 | 3.077e-10 |  |
| ENSRNOG00000007467 | 3 | 3.061 | 3.225e-10 |  |
| ENSRNOG00000053384 | 3 | 2.715 | 3.234e-10 |  |
| ENSRNOG00000049766 | 3 | 3.368 | 3.234e-10 |  |
| ENSRNOG00000013102 | 3 | 2.332 | 3.234e-10 |  |
| ENSRNOG00000015160 | 2 | 3.708 | 3.234e-10 |  |
| ENSRNOG00000016512 | 5 | 3.84 | 3.282e-10 |  |
| ENSRNOG00000001254 | 4 | 2.503 | 3.554e-10 |  |
| ENSRNOG00000011777 | 3 | 2.576 | 3.556e-10 |  |
| ENSRNOG00000003736 | 1 | 2.097 | 3.563e-10 |  |
| ENSRNOG00000004063 | 5 | 2.861 | 3.567e-10 |  |
| ENSRNOG00000015430 | 3 | 2.397 | 3.663e-10 |  |
| ENSRNOG00000005277 | 6 | 3.154 | 3.72e-10 |  |
| ENSRNOG00000009629 | 4 | 3.175 | 3.756e-10 |  |
| ENSRNOG00000006030 | 6 | 3.446 | 3.818e-10 |  |
| ENSRNOG00000002881 | 3 | 2.797 | 3.849e-10 |  |
| ENSRNOG00000002930 | 2 | 3.355 | 3.91e-10 |  |
| ENSRNOG00000019141 | 5 | 3.299 | 3.988e-10 |  |
| ENSRNOG00000000515 | 6 | 3.237 | 4.205e-10 |  |
| ENSRNOG00000029598 | 2 | 2.051 | 4.517e-10 |  |
| ENSRNOG00000061403 | 2 | 1.964 | 4.952e-10 |  |
| ENSRNOG00000042137 | 3 | 2.041 | 5.29e-10 |  |
| ENSRNOG00000017511 | 2 | 3.379 | 5.399e-10 |  |
| ENSRNOG00000017212 | 3 | 2.528 | 5.623e-10 |  |
| ENSRNOG00000028541 | 6 | 3.783 | 5.664e-10 |  |
| ENSRNOG00000016281 | 4 | 2.55 | 5.664e-10 |  |
| ENSRNOG00000003098 | 3 | 3.278 | 5.671e-10 |  |
| ENSRNOG00000057620 | 2 | 2.069 | 5.824e-10 |  |
| ENSRNOG00000017416 | 2 | 2.608 | 5.857e-10 |  |
| ENSRNOG00000008237 | 1 | 2.461 | 5.934e-10 |  |
| ENSRNOG00000003657 | 3 | 2.143 | 5.971e-10 |  |
| ENSRNOG00000014170 | 3 | 2.317 | 6.136e-10 |  |
| ENSRNOG00000017428 | 3 | 2.902 | 6.192e-10 |  |
| ENSRNOG00000001480 | 1 | 1.997 | 6.246e-10 |  |
| ENSRNOG00000009334 | 3 | 2.706 | 6.33e-10 |  |
| ENSRNOG00000014424 | 3 | 3.527 | 6.493e-10 |  |
| ENSRNOG00000011181 | 3 | 2.468 | 6.541e-10 |  |
| ENSRNOG00000013917 | 1 | 2.22 | 6.737e-10 |  |
| ENSRNOG00000020560 | 1 | 2.882 | 6.877e-10 |  |
| ENSRNOG00000012972 | 5 | 2.964 | 6.937e-10 |  |
| ENSRNOG00000033734 | 5 | 3.722 | 6.997e-10 |  |
| ENSRNOG00000017434 | 5 | 2.667 | 7.022e-10 |  |
| ENSRNOG00000005678 | 1 | 2.387 | 7.087e-10 |  |
| ENSRNOG00000016926 | 3 | 2.431 | 7.207e-10 |  |
| ENSRNOG00000016177 | 6 | 3.474 | 7.31e-10 |  |
| ENSRNOG00000008986 | 3 | 3.539 | 7.31e-10 |  |
| ENSRNOG00000002607 | 5 | 3.383 | 7.387e-10 |  |
| ENSRNOG00000010507 | 5 | 3.702 | 7.572e-10 |  |
| ENSRNOG00000003648 | 5 | 4.046 | 7.745e-10 |  |
| ENSRNOG00000032472 | 3 | 4.327 | 7.923e-10 |  |
| ENSRNOG00000020132 | 5 | 3.052 | 8.089e-10 |  |
| ENSRNOG00000039496 | 2 | 1.712 | 8.187e-10 |  |
| ENSRNOG00000016846 | 1 | 1.71 | 8.353e-10 |  |
| ENSRNOG00000009204 | 5 | 2.531 | 8.477e-10 |  |
| ENSRNOG00000020300 | 1 | 1.787 | 8.477e-10 |  |
| ENSRNOG00000029148 | 3 | 2.38 | 8.764e-10 |  |
| ENSRNOG00000026919 | 5 | 3.716 | 8.986e-10 |  |
| ENSRNOG00000012830 | 3 | 2.772 | 9.126e-10 |  |
| ENSRNOG00000029911 | 3 | 3.709 | 9.177e-10 |  |
| ENSRNOG00000060448 | 5 | 2.229 | 9.31e-10 |  |
| ENSRNOG00000009031 | 3 | 6.063 | 9.348e-10 |  |
| ENSRNOG00000057760 | 5 | 2.745 | 1.008e-09 |  |
| ENSRNOG00000003207 | 2 | 1.967 | 1.019e-09 |  |
| ENSRNOG00000059140 | 2 | 2.09 | 1.056e-09 |  |
| ENSRNOG00000051548 | 3 | 2.364 | 1.078e-09 |  |
| ENSRNOG00000010105 | 2 | 2.569 | 1.096e-09 |  |
| ENSRNOG00000026203 | 5 | 2.706 | 1.155e-09 |  |
| ENSRNOG00000042152 | 5 | 3.629 | 1.155e-09 |  |
| ENSRNOG00000013074 | 3 | 2.952 | 1.172e-09 |  |
| ENSRNOG00000012160 | 1 | 1.835 | 1.172e-09 |  |
| ENSRNOG00000020822 | 2 | 1.921 | 1.18e-09 |  |
| ENSRNOG00000010626 | 5 | 3.537 | 1.18e-09 |  |
| ENSRNOG00000007033 | 3 | 2.716 | 1.18e-09 |  |
| ENSRNOG00000003018 | 2 | 2.036 | 1.214e-09 |  |
| ENSRNOG00000060100 | 5 | 2.318 | 1.227e-09 |  |
| ENSRNOG00000010524 | 1 | 2.547 | 1.264e-09 |  |
| ENSRNOG00000019022 | 1 | 2.369 | 1.264e-09 |  |
| ENSRNOG00000003785 | 5 | 3.92 | 1.283e-09 |  |
| ENSRNOG00000002097 | 3 | 4.626 | 1.36e-09 |  |
| ENSRNOG00000018715 | 3 | 2.381 | 1.366e-09 |  |
| ENSRNOG00000039976 | 3 | 2.908 | 1.371e-09 |  |
| ENSRNOG00000012835 | 3 | 2.712 | 1.38e-09 |  |
| ENSRNOG00000026235 | 2 | 1.939 | 1.387e-09 |  |
| ENSRNOG00000020431 | 3 | 2.21 | 1.399e-09 |  |
| ENSRNOG00000000321 | 3 | 2.707 | 1.416e-09 |  |
| ENSRNOG00000055111 | 3 | 2.341 | 1.422e-09 |  |
| ENSRNOG00000015423 | 2 | 2.429 | 1.443e-09 |  |
| ENSRNOG00000053494 | 5 | 4.342 | 1.448e-09 |  |
| ENSRNOG00000005825 | 4 | 2.51 | 1.467e-09 |  |
| ENSRNOG00000028077 | 5 | 2.997 | 1.481e-09 |  |
| ENSRNOG00000052564 | 4 | 2.603 | 1.481e-09 |  |
| ENSRNOG00000027747 | 5 | 2.557 | 1.523e-09 |  |
| ENSRNOG00000023972 | 4 | 2.511 | 1.552e-09 |  |
| ENSRNOG00000033389 | 3 | 2.258 | 1.601e-09 |  |
| ENSRNOG00000028650 | 2 | 2.198 | 1.605e-09 |  |
| ENSRNOG00000016343 | 2 | 2.52 | 1.623e-09 |  |
| ENSRNOG00000019018 | 1 | 2.684 | 1.659e-09 |  |
| ENSRNOG00000003338 | 2 | 2.177 | 1.659e-09 |  |
| ENSRNOG00000012019 | 3 | 2.937 | 1.678e-09 |  |
| ENSRNOG00000017114 | 2 | 2.132 | 1.695e-09 |  |
| ENSRNOG00000003464 | 1 | 2.471 | 1.732e-09 |  |
| ENSRNOG00000016166 | 4 | 1.776 | 1.762e-09 |  |
| ENSRNOG00000000406 | 7 | 3.667 | 1.785e-09 |  |
| ENSRNOG00000017163 | 3 | 2.23 | 1.787e-09 |  |
| ENSRNOG00000039902 | 3 | 2.148 | 1.788e-09 |  |
| ENSRNOG00000014371 | 3 | 2.616 | 1.804e-09 |  |
| ENSRNOG00000059827 | 5 | 3.376 | 1.915e-09 |  |
| ENSRNOG00000058539 | 2 | 2.425 | 1.924e-09 |  |
| ENSRNOG00000021713 | 3 | 2.918 | 1.94e-09 |  |
| ENSRNOG00000019810 | 2 | 1.967 | 1.975e-09 |  |
| ENSRNOG00000024986 | 3 | 3.623 | 1.991e-09 |  |
| ENSRNOG00000020714 | 3 | 2.212 | 2.033e-09 |  |
| ENSRNOG00000056847 | 2 | 3.494 | 2.067e-09 |  |
| ENSRNOG00000008678 | 3 | 2.254 | 2.123e-09 |  |
| ENSRNOG00000002711 | 3 | 2.335 | 2.153e-09 |  |
| ENSRNOG00000005053 | 2 | 2.722 | 2.175e-09 |  |
| ENSRNOG00000057125 | 3 | 2.8 | 2.187e-09 |  |
| ENSRNOG00000010529 | 1 | 2.161 | 2.23e-09 |  |
| ENSRNOG00000017671 | 1 | 1.829 | 2.23e-09 |  |
| ENSRNOG00000021220 | 3 | 2.74 | 2.362e-09 |  |
| ENSRNOG00000005971 | 3 | 2.895 | 2.368e-09 |  |
| ENSRNOG00000032240 | 1 | 1.835 | 2.43e-09 |  |
| ENSRNOG00000024277 | 3 | 2.193 | 2.435e-09 |  |
| ENSRNOG00000054474 | 3 | 2.314 | 2.435e-09 |  |
| ENSRNOG00000028856 | 3 | 2.545 | 2.458e-09 |  |
| ENSRNOG00000005772 | 3 | 2.506 | 2.473e-09 |  |
| ENSRNOG00000052064 | 2 | 1.753 | 2.522e-09 |  |
| ENSRNOG00000033658 | 3 | 2.25 | 2.646e-09 |  |
| ENSRNOG00000016688 | 3 | 2.334 | 2.646e-09 |  |
| ENSRNOG00000058891 | 5 | 2.847 | 2.662e-09 |  |
| ENSRNOG00000001469 | 1 | 2.561 | 2.685e-09 |  |
| ENSRNOG00000016412 | 1 | 1.908 | 2.703e-09 |  |
| ENSRNOG00000026985 | 2 | 2.302 | 2.845e-09 |  |
| ENSRNOG00000014021 | 6 | 3.579 | 2.896e-09 |  |
| ENSRNOG00000000869 | 3 | 1.733 | 2.914e-09 |  |
| ENSRNOG00000006735 | 3 | 2.296 | 2.942e-09 |  |
| ENSRNOG00000001757 | 3 | 3.748 | 2.947e-09 |  |
| ENSRNOG00000032844 | 4 | 1.927 | 3.055e-09 |  |
| ENSRNOG00000059894 | 3 | 2.478 | 3.085e-09 |  |
| ENSRNOG00000020138 | 6 | 3.182 | 3.187e-09 |  |
| ENSRNOG00000017209 | 5 | 2.908 | 3.237e-09 |  |
| ENSRNOG00000020625 | 3 | 2.49 | 3.258e-09 |  |
| ENSRNOG00000012658 | 5 | 2.978 | 3.309e-09 |  |
| ENSRNOG00000006116 | 3 | 2.115 | 3.325e-09 |  |
| ENSRNOG00000022305 | 3 | 2.079 | 3.379e-09 |  |
| ENSRNOG00000060614 | 1 | 2.117 | 3.477e-09 |  |
| ENSRNOG00000010319 | 4 | 1.955 | 3.524e-09 |  |
| ENSRNOG00000029141 | 7 | 3.178 | 3.636e-09 |  |
| ENSRNOG00000013452 | 1 | 1.887 | 3.77e-09 |  |
| ENSRNOG00000039544 | 6 | 2.846 | 3.777e-09 |  |
| ENSRNOG00000001249 | 4 | 2.402 | 3.787e-09 |  |
| ENSRNOG00000001414 | 1 | 3.195 | 3.891e-09 |  |
| ENSRNOG00000008005 | 2 | 1.932 | 4.126e-09 |  |
| ENSRNOG00000002414 | 2 | 1.938 | 4.192e-09 |  |
| ENSRNOG00000042978 | 2 | 2.528 | 4.192e-09 |  |
| ENSRNOG00000007332 | 3 | 2.832 | 4.248e-09 |  |
| ENSRNOG00000007387 | 1 | 2.058 | 4.307e-09 |  |
| ENSRNOG00000007583 | 2 | 1.775 | 4.346e-09 |  |
| ENSRNOG00000021274 | 6 | 3.413 | 4.459e-09 |  |
| ENSRNOG00000052003 | 3 | 2.288 | 4.512e-09 |  |
| ENSRNOG00000014963 | 3 | 3.356 | 4.542e-09 |  |
| ENSRNOG00000019244 | 1 | 2.144 | 4.664e-09 |  |
| ENSRNOG00000032339 | 3 | 3.307 | 4.718e-09 |  |
| ENSRNOG00000028460 | 5 | 2.978 | 4.799e-09 |  |
| ENSRNOG00000040287 | 3 | 4.16 | 4.825e-09 |  |
| ENSRNOG00000030210 | 3 | 4.13 | 4.944e-09 |  |
| ENSRNOG00000012294 | 3 | 2.263 | 4.944e-09 |  |
| ENSRNOG00000015810 | 5 | 2.129 | 5.031e-09 |  |
| ENSRNOG00000013194 | 3 | 2.41 | 5.164e-09 |  |
| ENSRNOG00000001476 | 5 | 3.579 | 5.206e-09 |  |
| ENSRNOG00000008622 | 6 | 3.298 | 5.321e-09 |  |
| ENSRNOG00000015308 | 3 | 2.309 | 5.373e-09 |  |
| ENSRNOG00000012630 | 1 | 2.061 | 5.39e-09 |  |
| ENSRNOG00000001224 | 1 | 1.825 | 5.39e-09 |  |
| ENSRNOG00000058866 | 2 | 1.898 | 5.42e-09 |  |
| ENSRNOG00000018087 | 4 | 2.12 | 5.559e-09 |  |
| ENSRNOG00000008346 | 3 | 3.129 | 5.592e-09 |  |
| ENSRNOG00000021669 | 1 | 2.939 | 5.841e-09 |  |
| ENSRNOG00000017477 | 3 | 2.503 | 5.907e-09 |  |
| ENSRNOG00000007733 | 3 | 2.084 | 6.009e-09 |  |
| ENSRNOG00000029055 | 3 | 2.44 | 6.303e-09 |  |
| ENSRNOG00000010718 | 5 | 2.57 | 6.423e-09 |  |
| ENSRNOG00000005935 | 3 | 3.168 | 6.441e-09 |  |
| ENSRNOG00000004276 | 3 | 2.234 | 6.473e-09 |  |
| ENSRNOG00000013240 | 5 | 2.82 | 6.488e-09 |  |
| ENSRNOG00000019412 | 1 | 2.758 | 6.544e-09 |  |
| ENSRNOG00000033496 | 5 | 3.723 | 6.544e-09 |  |
| ENSRNOG00000000307 | 3 | 2.047 | 6.544e-09 |  |
| ENSRNOG00000056955 | 3 | 2.614 | 6.567e-09 |  |
| ENSRNOG00000010701 | 6 | 2.809 | 6.626e-09 |  |
| ENSRNOG00000011944 | 2 | 2.2 | 6.704e-09 |  |
| ENSRNOG00000007650 | 4 | 2.003 | 6.717e-09 |  |
| ENSRNOG00000021390 | 5 | 1.958 | 6.832e-09 |  |
| ENSRNOG00000002244 | 1 | 1.896 | 6.832e-09 |  |
| ENSRNOG00000005695 | 4 | 2.744 | 6.832e-09 |  |
| ENSRNOG00000051952 | 1 | 2.477 | 6.84e-09 |  |
| ENSRNOG00000030266 | 2 | 1.954 | 6.842e-09 |  |
| ENSRNOG00000004695 | 3 | 5.89 | 6.862e-09 |  |
| ENSRNOG00000014867 | 3 | 2.383 | 6.99e-09 |  |
| ENSRNOG00000008936 | 3 | 2.867 | 7.032e-09 |  |
| ENSRNOG00000008165 | 2 | 2.047 | 7.339e-09 |  |
| ENSRNOG00000059705 | 3 | 1.852 | 7.418e-09 |  |
| ENSRNOG00000007980 | 2 | 3.141 | 7.446e-09 |  |
| ENSRNOG00000017767 | 2 | 2.116 | 7.672e-09 |  |
| ENSRNOG00000052150 | 3 | 3.346 | 7.703e-09 |  |
| ENSRNOG00000018859 | 3 | 2.172 | 7.889e-09 |  |
| ENSRNOG00000007090 | 5 | 2.839 | 8.031e-09 |  |
| ENSRNOG00000011453 | 5 | 2.604 | 8.189e-09 |  |
| ENSRNOG00000019943 | 3 | 2.151 | 8.203e-09 |  |
| ENSRNOG00000017510 | 4 | 1.867 | 8.233e-09 |  |
| ENSRNOG00000003732 | 3 | 2.873 | 8.298e-09 |  |
| ENSRNOG00000006324 | 5 | 2.952 | 8.483e-09 |  |
| ENSRNOG00000032656 | 5 | 3.911 | 8.642e-09 |  |
| ENSRNOG00000028238 | 5 | 3.318 | 8.684e-09 |  |
| ENSRNOG00000027125 | 2 | 1.917 | 8.753e-09 |  |
| ENSRNOG00000006426 | 3 | 4.341 | 8.782e-09 |  |
| ENSRNOG00000014837 | 3 | 2.71 | 9.024e-09 |  |
| ENSRNOG00000017628 | 1 | 2.022 | 9.029e-09 |  |
| ENSRNOG00000016483 | 6 | 3.83 | 9.086e-09 |  |
| ENSRNOG00000015845 | 1 | 1.895 | 9.255e-09 |  |
| ENSRNOG00000015667 | 1 | 2.415 | 9.27e-09 |  |
| ENSRNOG00000010378 | 6 | 3.267 | 9.353e-09 |  |
| ENSRNOG00000020813 | 2 | 2.065 | 9.364e-09 |  |
| ENSRNOG00000000451 | 4 | 1.751 | 9.378e-09 |  |
| ENSRNOG00000011841 | 3 | 2.426 | 9.441e-09 |  |
| ENSRNOG00000002660 | 3 | 2.741 | 9.512e-09 |  |
| ENSRNOG00000050404 | 3 | 2.417 | 9.658e-09 |  |
| ENSRNOG00000009014 | 6 | 3.313 | 9.811e-09 |  |
| ENSRNOG00000012960 | 2 | 1.725 | 9.813e-09 |  |
| ENSRNOG00000011654 | 3 | 2.053 | 9.898e-09 |  |
| ENSRNOG00000016885 | 1 | 2.447 | 9.981e-09 |  |
| ENSRNOG00000005807 | 3 | 1.904 | 1.034e-08 |  |
| ENSRNOG00000022884 | 3 | 2.256 | 1.036e-08 |  |
| ENSRNOG00000013334 | 6 | 3.574 | 1.04e-08 |  |
| ENSRNOG00000017897 | 2 | 3.923 | 1.04e-08 |  |
| ENSRNOG00000024178 | 5 | 2.709 | 1.073e-08 |  |
| ENSRNOG00000012840 | 4 | 1.692 | 1.088e-08 |  |
| ENSRNOG00000033984 | 3 | 3.065 | 1.096e-08 |  |
| ENSRNOG00000004821 | 2 | 2.085 | 1.097e-08 |  |
| ENSRNOG00000013656 | 5 | 2.8 | 1.107e-08 |  |
| ENSRNOG00000013222 | 3 | 4.402 | 1.113e-08 |  |
| ENSRNOG00000016225 | 2 | 1.864 | 1.156e-08 |  |
| ENSRNOG00000038328 | 5 | 2.578 | 1.179e-08 |  |
| ENSRNOG00000012811 | 2 | 3.882 | 1.191e-08 |  |
| ENSRNOG00000014293 | 3 | 2.017 | 1.197e-08 |  |
| ENSRNOG00000014479 | 1 | 1.649 | 1.21e-08 |  |
| ENSRNOG00000042741 | 6 | 2.768 | 1.212e-08 |  |
| ENSRNOG00000008257 | 5 | 2.651 | 1.232e-08 |  |
| ENSRNOG00000022710 | 3 | 2.954 | 1.232e-08 |  |
| ENSRNOG00000012566 | 6 | 2.926 | 1.236e-08 |  |
| ENSRNOG00000056228 | 3 | 4.008 | 1.237e-08 |  |
| ENSRNOG00000023640 | 5 | 2.935 | 1.257e-08 |  |
| ENSRNOG00000018929 | 3 | 2.078 | 1.271e-08 |  |
| ENSRNOG00000061376 | 5 | 3.224 | 1.276e-08 |  |
| ENSRNOG00000001645 | 1 | 1.836 | 1.319e-08 |  |
| ENSRNOG00000028415 | 2 | 2.205 | 1.325e-08 |  |
| ENSRNOG00000058470 | 1 | 2.272 | 1.33e-08 |  |
| ENSRNOG00000043219 | 5 | 3.482 | 1.335e-08 |  |
| ENSRNOG00000020525 | 5 | 2.351 | 1.379e-08 |  |
| ENSRNOG00000011925 | 6 | 3.255 | 1.398e-08 |  |
| ENSRNOG00000017172 | 2 | 1.992 | 1.399e-08 |  |
| ENSRNOG00000028545 | 5 | 2.954 | 1.411e-08 |  |
| ENSRNOG00000013727 | 3 | 2.336 | 1.433e-08 |  |
| ENSRNOG00000015449 | 5 | 3.999 | 1.446e-08 |  |
| ENSRNOG00000019965 | 2 | 2.015 | 1.471e-08 |  |
| ENSRNOG00000060665 | 3 | 1.937 | 1.471e-08 |  |
| ENSRNOG00000002014 | 5 | 2.173 | 1.531e-08 |  |
| ENSRNOG00000022537 | 5 | 2.732 | 1.554e-08 |  |
| ENSRNOG00000010872 | 1 | 2.047 | 1.563e-08 |  |
| ENSRNOG00000059857 | 1 | 2.57 | 1.579e-08 |  |
| ENSRNOG00000024757 | 5 | 2.177 | 1.618e-08 |  |
| ENSRNOG00000020470 | 2 | 1.84 | 1.66e-08 |  |
| ENSRNOG00000019582 | 3 | 2.586 | 1.688e-08 |  |
| ENSRNOG00000018752 | 3 | 1.825 | 1.709e-08 |  |
| ENSRNOG00000002013 | 3 | 4.281 | 1.718e-08 |  |
| ENSRNOG00000016831 | 1 | 1.876 | 1.748e-08 |  |
| ENSRNOG00000018770 | 3 | 2.159 | 1.768e-08 |  |
| ENSRNOG00000039284 | 2 | 1.808 | 1.769e-08 |  |
| ENSRNOG00000046428 | 3 | 2.115 | 1.794e-08 |  |
| ENSRNOG00000007564 | 3 | 2.028 | 1.799e-08 |  |
| ENSRNOG00000017328 | 1 | 1.923 | 1.799e-08 |  |
| ENSRNOG00000007057 | 5 | 3.38 | 1.81e-08 |  |
| ENSRNOG00000056209 | 6 | 2.804 | 1.81e-08 |  |
| ENSRNOG00000015465 | 5 | 2.596 | 1.825e-08 |  |
| ENSRNOG00000014768 | 6 | 3.165 | 1.826e-08 |  |
| ENSRNOG00000024264 | 5 | 2.71 | 1.83e-08 |  |
| ENSRNOG00000009488 | 4 | 2.793 | 1.854e-08 |  |
| ENSRNOG00000050794 | 3 | 2.501 | 1.863e-08 |  |
| ENSRNOG00000018350 | 1 | 1.561 | 1.932e-08 |  |
| ENSRNOG00000033835 | 5 | 2.72 | 1.934e-08 |  |
| ENSRNOG00000049517 | 1 | 2.166 | 1.966e-08 |  |
| ENSRNOG00000022309 | 3 | 2.573 | 1.993e-08 |  |
| ENSRNOG00000059903 | 5 | 2.471 | 1.997e-08 |  |
| ENSRNOG00000011015 | 2 | 2.003 | 2.022e-08 |  |
| ENSRNOG00000027592 | 2 | 1.889 | 2.03e-08 |  |
| ENSRNOG00000038722 | 2 | 2.078 | 2.052e-08 |  |
| ENSRNOG00000019316 | 1 | 2.032 | 2.143e-08 |  |
| ENSRNOG00000053450 | 4 | 1.909 | 2.222e-08 |  |
| ENSRNOG00000008203 | 6 | 3.411 | 2.236e-08 |  |
| ENSRNOG00000018815 | 3 | 2.529 | 2.251e-08 |  |
| ENSRNOG00000043007 | 2 | 1.983 | 2.278e-08 |  |
| ENSRNOG00000018509 | 5 | 2.428 | 2.288e-08 |  |
| ENSRNOG00000017485 | 3 | 2.134 | 2.359e-08 |  |
| ENSRNOG00000054515 | 2 | 1.981 | 2.392e-08 |  |
| ENSRNOG00000018524 | 1 | 1.971 | 2.475e-08 |  |
| ENSRNOG00000021285 | 5 | 3.044 | 2.492e-08 |  |
| ENSRNOG00000026604 | 3 | 2.208 | 2.505e-08 |  |
| ENSRNOG00000060464 | 3 | 2.712 | 2.506e-08 |  |
| ENSRNOG00000016684 | 6 | 3.517 | 2.554e-08 |  |
| ENSRNOG00000017803 | 2 | 1.757 | 2.555e-08 |  |
| ENSRNOG00000054860 | 3 | 2.284 | 2.555e-08 |  |
| ENSRNOG00000038738 | 6 | 2.514 | 2.555e-08 |  |
| ENSRNOG00000009625 | 2 | 1.659 | 2.581e-08 |  |
| ENSRNOG00000027204 | 5 | 2.505 | 2.617e-08 |  |
| ENSRNOG00000017206 | 1 | 2.974 | 2.617e-08 |  |
| ENSRNOG00000004482 | 3 | 2.315 | 2.721e-08 |  |
| ENSRNOG00000021560 | 5 | 2.641 | 2.727e-08 |  |
| ENSRNOG00000004743 | 5 | 3.533 | 2.727e-08 |  |
| ENSRNOG00000017637 | 5 | 3.203 | 2.727e-08 |  |
| ENSRNOG00000047314 | 1 | 1.606 | 2.798e-08 |  |
| ENSRNOG00000009892 | 5 | 2.881 | 2.877e-08 |  |
| ENSRNOG00000021104 | 2 | 1.945 | 2.877e-08 |  |
| ENSRNOG00000042041 | 6 | 3.222 | 3.065e-08 |  |
| ENSRNOG00000036877 | 7 | 3.239 | 3.128e-08 |  |
| ENSRNOG00000008759 | 3 | 2.042 | 3.138e-08 |  |
| ENSRNOG00000012862 | 2 | 4.854 | 3.142e-08 |  |
| ENSRNOG00000002934 | 4 | 2.117 | 3.156e-08 |  |
| ENSRNOG00000031665 | 3 | 2.221 | 3.177e-08 |  |
| ENSRNOG00000013034 | 5 | 4.935 | 3.182e-08 |  |
| ENSRNOG00000011723 | 3 | 2.255 | 3.204e-08 |  |
| ENSRNOG00000055962 | 4 | 1.866 | 3.273e-08 |  |
| ENSRNOG00000014960 | 5 | 2.457 | 3.302e-08 |  |
| ENSRNOG00000060773 | 5 | 2.349 | 3.302e-08 |  |
| ENSRNOG00000053945 | 5 | 2.269 | 3.309e-08 |  |
| ENSRNOG00000016377 | 3 | 2.574 | 3.451e-08 |  |
| ENSRNOG00000006715 | 5 | 2.65 | 3.481e-08 |  |
| ENSRNOG00000052376 | 5 | 2.718 | 3.529e-08 |  |
| ENSRNOG00000020579 | 6 | 3.54 | 3.667e-08 |  |
| ENSRNOG00000046891 | 2 | 1.813 | 3.736e-08 |  |
| ENSRNOG00000011631 | 6 | 2.996 | 3.775e-08 |  |
| ENSRNOG00000053766 | 6 | 3.072 | 3.903e-08 |  |
| ENSRNOG00000006995 | 1 | 1.592 | 3.924e-08 |  |
| ENSRNOG00000006767 | 3 | 2.025 | 3.94e-08 |  |
| ENSRNOG00000014231 | 5 | 3.031 | 3.94e-08 |  |
| ENSRNOG00000009503 | 5 | 2.246 | 3.98e-08 |  |
| ENSRNOG00000004921 | 3 | 2.395 | 4.071e-08 |  |
| ENSRNOG00000020803 | 7 | 3.253 | 4.072e-08 |  |
| ENSRNOG00000019311 | 1 | 1.769 | 4.094e-08 |  |
| ENSRNOG00000007258 | 5 | 2.671 | 4.135e-08 |  |
| ENSRNOG00000016544 | 6 | 3.09 | 4.135e-08 |  |
| ENSRNOG00000037302 | 5 | 2.684 | 4.141e-08 |  |
| ENSRNOG00000003687 | 2 | 2.062 | 4.395e-08 |  |
| ENSRNOG00000020279 | 3 | 1.858 | 4.49e-08 |  |
| ENSRNOG00000039582 | 3 | 2.489 | 4.501e-08 |  |
| ENSRNOG00000005810 | 3 | 1.931 | 4.563e-08 |  |
| ENSRNOG00000004650 | 7 | 3.171 | 4.576e-08 |  |
| ENSRNOG00000030486 | 5 | 2.93 | 4.709e-08 |  |
| ENSRNOG00000002728 | 2 | 2.87 | 4.884e-08 |  |
| ENSRNOG00000015794 | 5 | 2.514 | 4.888e-08 |  |
| ENSRNOG00000032778 | 2 | 1.772 | 5.079e-08 |  |
| ENSRNOG00000000700 | 3 | 2.401 | 5.117e-08 |  |
| ENSRNOG00000007923 | 3 | 2.498 | 5.207e-08 |  |
| ENSRNOG00000002273 | 2 | 1.641 | 5.283e-08 |  |
| ENSRNOG00000007600 | 5 | 3.034 | 5.456e-08 |  |
| ENSRNOG00000039390 | 3 | 1.941 | 5.481e-08 |  |
| ENSRNOG00000024435 | 7 | 2.905 | 5.536e-08 |  |
| ENSRNOG00000008016 | 1 | 2.432 | 5.539e-08 |  |
| ENSRNOG00000016782 | 6 | 2.696 | 5.676e-08 |  |
| ENSRNOG00000011589 | 1 | 1.651 | 5.709e-08 |  |
| ENSRNOG00000004540 | 5 | 3.214 | 5.883e-08 |  |
| ENSRNOG00000002664 | 2 | 1.927 | 6.085e-08 |  |
| ENSRNOG00000015346 | 5 | 2.506 | 6.101e-08 |  |
| ENSRNOG00000013577 | 2 | 2.458 | 6.255e-08 |  |
| ENSRNOG00000007025 | 6 | 2.853 | 6.273e-08 |  |
| ENSRNOG00000003782 | 2 | 1.633 | 6.343e-08 |  |
| ENSRNOG00000004393 | 7 | 2.994 | 6.521e-08 |  |
| ENSRNOG00000029938 | 2 | 1.966 | 6.554e-08 |  |
| ENSRNOG00000042478 | 6 | 2.746 | 6.673e-08 |  |
| ENSRNOG00000016164 | 3 | 4.733 | 6.735e-08 |  |
| ENSRNOG00000022838 | 6 | 2.901 | 6.898e-08 |  |
| ENSRNOG00000000655 | 1 | 1.555 | 7.038e-08 |  |
| ENSRNOG00000014087 | 1 | 2.179 | 7.236e-08 |  |
| ENSRNOG00000006236 | 3 | 1.805 | 7.414e-08 |  |
| ENSRNOG00000018611 | 5 | 3.287 | 7.415e-08 |  |
| ENSRNOG00000019689 | 1 | 2.054 | 7.417e-08 |  |
| ENSRNOG00000007089 | 4 | 1.613 | 7.953e-08 |  |
| ENSRNOG00000019440 | 3 | 2.466 | 7.964e-08 |  |
| ENSRNOG00000060141 | 3 | 5.191 | 7.978e-08 |  |
| ENSRNOG00000026941 | 3 | 2.504 | 8.089e-08 |  |
| ENSRNOG00000006957 | 5 | 2.589 | 8.136e-08 |  |
| ENSRNOG00000000805 | 1 | 1.789 | 8.199e-08 |  |
| ENSRNOG00000008356 | 5 | 2.783 | 8.238e-08 |  |
| ENSRNOG00000033136 | 6 | 3.42 | 8.249e-08 |  |
| ENSRNOG00000015529 | 3 | 2.302 | 8.49e-08 |  |
| ENSRNOG00000015408 | 5 | 3.341 | 8.594e-08 |  |
| ENSRNOG00000001379 | 4 | 1.994 | 9.001e-08 |  |
| ENSRNOG00000004766 | 3 | 2.131 | 9.102e-08 |  |
| ENSRNOG00000011346 | 3 | 1.868 | 9.102e-08 |  |
| ENSRNOG00000045913 | 5 | 2.908 | 9.102e-08 |  |
| ENSRNOG00000029178 | 1 | 1.958 | 9.143e-08 |  |
| ENSRNOG00000014973 | 7 | 2.867 | 9.164e-08 |  |
| ENSRNOG00000042189 | 1 | 1.682 | 9.301e-08 |  |
| ENSRNOG00000015382 | 3 | 2.378 | 9.403e-08 |  |
| ENSRNOG00000012002 | 1 | 1.616 | 9.403e-08 |  |
| ENSRNOG00000005190 | 1 | 1.869 | 9.508e-08 |  |
| ENSRNOG00000002192 | 1 | 1.949 | 9.519e-08 |  |
| ENSRNOG00000024651 | 6 | 3.36 | 9.538e-08 |  |
| ENSRNOG00000015397 | 3 | 1.796 | 9.637e-08 |  |
| ENSRNOG00000043286 | 3 | 2.018 | 9.696e-08 |  |
| ENSRNOG00000014010 | 5 | 3.482 | 9.736e-08 |  |
| ENSRNOG00000045821 | 5 | 2.627 | 9.87e-08 |  |
| ENSRNOG00000004613 | 3 | 1.932 | 9.972e-08 |  |
| ENSRNOG00000009867 | 2 | 1.972 | 1.078e-07 |  |
| ENSRNOG00000002810 | 3 | 2.004 | 1.145e-07 |  |
| ENSRNOG00000047300 | 6 | 3.67 | 1.174e-07 |  |
| ENSRNOG00000005954 | 6 | 3.061 | 1.176e-07 |  |
| ENSRNOG00000052070 | 1 | 1.872 | 1.183e-07 |  |
| ENSRNOG00000015093 | 1 | 1.787 | 1.222e-07 |  |
| ENSRNOG00000015948 | 3 | 2.373 | 1.242e-07 |  |
| ENSRNOG00000007584 | 1 | 1.533 | 1.267e-07 |  |
| ENSRNOG00000012989 | 5 | 3.788 | 1.292e-07 |  |
| ENSRNOG00000003544 | 7 | 3.385 | 1.314e-07 |  |
| ENSRNOG00000031475 | 3 | 2.257 | 1.321e-07 |  |
| ENSRNOG00000012628 | 6 | 2.114 | 1.324e-07 |  |
| ENSRNOG00000019556 | 1 | 2.028 | 1.368e-07 |  |
| ENSRNOG00000005153 | 3 | 1.826 | 1.446e-07 |  |
| ENSRNOG00000009884 | 1 | 2.29 | 1.452e-07 |  |
| ENSRNOG00000036802 | 7 | 3.258 | 1.492e-07 |  |
| ENSRNOG00000013148 | 5 | 2.033 | 1.517e-07 |  |
| ENSRNOG00000016257 | 1 | 1.805 | 1.562e-07 |  |
| ENSRNOG00000011750 | 3 | 1.714 | 1.585e-07 |  |
| ENSRNOG00000010516 | 2 | 1.742 | 1.606e-07 |  |
| ENSRNOG00000021161 | 1 | 1.725 | 1.642e-07 |  |
| ENSRNOG00000053026 | 3 | 2.233 | 1.673e-07 |  |
| ENSRNOG00000017260 | 2 | 1.88 | 1.694e-07 |  |
| ENSRNOG00000004195 | 6 | 2.609 | 1.703e-07 |  |
| ENSRNOG00000016689 | 3 | 1.811 | 1.741e-07 |  |
| ENSRNOG00000025384 | 6 | 2.895 | 1.797e-07 |  |
| ENSRNOG00000009919 | 5 | 3.066 | 1.842e-07 |  |
| ENSRNOG00000017155 | 3 | 2.161 | 1.845e-07 |  |
| ENSRNOG00000055672 | 2 | 4.818 | 1.845e-07 |  |
| ENSRNOG00000014896 | 6 | 3.466 | 1.869e-07 |  |
| ENSRNOG00000056714 | 2 | 1.559 | 1.869e-07 |  |
| ENSRNOG00000005291 | 5 | 2.228 | 1.884e-07 |  |
| ENSRNOG00000046660 | 5 | 2.352 | 1.91e-07 |  |
| ENSRNOG00000011253 | 5 | 3.359 | 1.924e-07 |  |
| ENSRNOG00000015401 | 6 | 3.034 | 1.96e-07 |  |
| ENSRNOG00000016041 | 3 | 1.948 | 1.988e-07 |  |
| ENSRNOG00000053577 | 3 | 2.09 | 2.011e-07 |  |
| ENSRNOG00000003453 | 2 | 1.874 | 2.031e-07 |  |
| ENSRNOG00000009785 | 3 | 2.028 | 2.043e-07 |  |
| ENSRNOG00000029212 | 3 | 2.62 | 2.201e-07 |  |
| ENSRNOG00000023340 | 7 | 2.99 | 2.208e-07 |  |
| ENSRNOG00000020027 | 1 | 1.742 | 2.23e-07 |  |
| ENSRNOG00000033722 | 6 | 2.665 | 2.26e-07 |  |
| ENSRNOG00000032150 | 6 | 3.618 | 2.266e-07 |  |
| ENSRNOG00000011054 | 1 | 1.507 | 2.362e-07 |  |
| ENSRNOG00000016294 | 1 | 1.563 | 2.376e-07 |  |
| ENSRNOG00000014156 | 5 | 2.325 | 2.403e-07 |  |
| ENSRNOG00000006674 | 5 | 2.579 | 2.436e-07 |  |
| ENSRNOG00000058289 | 6 | 2.623 | 2.45e-07 |  |
| ENSRNOG00000016423 | 1 | 1.581 | 2.477e-07 |  |
| ENSRNOG00000056297 | 2 | 1.625 | 2.477e-07 |  |
| ENSRNOG00000024594 | 5 | 2.289 | 2.477e-07 |  |
| ENSRNOG00000021381 | 3 | 2.755 | 2.487e-07 |  |
| ENSRNOG00000025120 | 7 | 3.152 | 2.518e-07 |  |
| ENSRNOG00000002105 | 5 | 2.431 | 2.542e-07 |  |
| ENSRNOG00000029394 | 3 | 2.859 | 2.575e-07 |  |
| ENSRNOG00000056786 | 2 | 1.837 | 2.579e-07 |  |
| ENSRNOG00000021586 | 3 | 1.792 | 2.681e-07 |  |
| ENSRNOG00000024711 | 5 | 2.226 | 2.714e-07 |  |
| ENSRNOG00000020505 | 2 | 1.597 | 2.727e-07 |  |
| ENSRNOG00000017156 | 6 | 2.707 | 2.801e-07 |  |
| ENSRNOG00000015520 | 5 | 2.7 | 2.825e-07 |  |
| ENSRNOG00000038286 | 5 | 2.404 | 2.831e-07 |  |
| ENSRNOG00000005034 | 3 | 1.967 | 2.843e-07 |  |
| ENSRNOG00000030759 | 7 | 2.637 | 2.843e-07 |  |
| ENSRNOG00000016910 | 2 | 1.683 | 2.843e-07 |  |
| ENSRNOG00000009144 | 5 | 2.571 | 2.894e-07 |  |
| ENSRNOG00000009311 | 5 | 2.654 | 2.905e-07 |  |
| ENSRNOG00000014513 | 3 | 1.896 | 2.972e-07 |  |
| ENSRNOG00000011644 | 5 | 2.584 | 2.986e-07 |  |
| ENSRNOG00000010183 | 1 | 1.709 | 2.99e-07 |  |
| ENSRNOG00000027936 | 5 | 2.978 | 3.06e-07 |  |
| ENSRNOG00000046050 | 3 | 1.695 | 3.147e-07 |  |
| ENSRNOG00000059540 | 5 | 2.509 | 3.194e-07 |  |
| ENSRNOG00000060524 | 7 | 2.894 | 3.195e-07 |  |
| ENSRNOG00000013679 | 3 | 1.804 | 3.23e-07 |  |
| ENSRNOG00000008045 | 2 | 2.159 | 3.239e-07 |  |
| ENSRNOG00000057569 | 1 | 2.238 | 3.239e-07 |  |
| ENSRNOG00000008749 | 1 | 1.972 | 3.258e-07 |  |
| ENSRNOG00000003217 | 4 | 1.688 | 3.283e-07 |  |
| ENSRNOG00000011313 | 6 | 2.81 | 3.299e-07 |  |
| ENSRNOG00000002418 | 3 | 1.856 | 3.299e-07 |  |
| ENSRNOG00000031801 | 5 | 2.669 | 3.442e-07 |  |
| ENSRNOG00000004341 | 5 | 3.18 | 3.463e-07 |  |
| ENSRNOG00000060241 | 5 | 2.216 | 3.507e-07 |  |
| ENSRNOG00000001312 | 2 | 1.924 | 3.513e-07 |  |
| ENSRNOG00000026787 | 6 | 2.817 | 3.646e-07 |  |
| ENSRNOG00000019365 | 1 | 1.981 | 3.707e-07 |  |
| ENSRNOG00000017438 | 1 | 2.225 | 3.72e-07 |  |
| ENSRNOG00000016308 | 3 | 1.733 | 3.736e-07 |  |
| ENSRNOG00000012991 | 3 | 1.858 | 3.736e-07 |  |
| ENSRNOG00000003802 | 3 | 2.213 | 3.742e-07 |  |
| ENSRNOG00000016525 | 5 | 2.597 | 3.762e-07 |  |
| ENSRNOG00000004487 | 5 | 2.62 | 3.769e-07 |  |
| ENSRNOG00000008040 | 6 | 2.669 | 3.808e-07 |  |
| ENSRNOG00000007597 | 3 | 2.661 | 3.831e-07 |  |
| ENSRNOG00000007129 | 5 | 2.658 | 3.854e-07 |  |
| ENSRNOG00000000842 | 3 | 2.133 | 3.894e-07 |  |
| ENSRNOG00000010891 | 3 | 1.869 | 4.068e-07 |  |
| ENSRNOG00000028774 | 7 | 2.574 | 4.079e-07 |  |
| ENSRNOG00000021962 | 7 | 2.763 | 4.092e-07 |  |
| ENSRNOG00000012531 | 5 | 2.651 | 4.15e-07 |  |
| ENSRNOG00000021782 | 7 | 3.335 | 4.155e-07 |  |
| ENSRNOG00000019604 | 3 | 1.816 | 4.207e-07 |  |
| ENSRNOG00000011332 | 6 | 2.553 | 4.221e-07 |  |
| ENSRNOG00000027942 | 7 | 2.735 | 4.256e-07 |  |
| ENSRNOG00000012950 | 6 | 2.769 | 4.457e-07 |  |
| ENSRNOG00000026143 | 5 | 2.467 | 4.482e-07 |  |
| ENSRNOG00000025184 | 6 | 3.378 | 4.508e-07 |  |
| ENSRNOG00000042596 | 7 | 3.345 | 4.508e-07 |  |
| ENSRNOG00000021663 | 1 | 2.766 | 4.564e-07 |  |
| ENSRNOG00000013825 | 3 | 2.504 | 4.674e-07 |  |
| ENSRNOG00000033215 | 4 | 1.676 | 4.688e-07 |  |
| ENSRNOG00000000479 | 3 | 2.316 | 4.755e-07 |  |
| ENSRNOG00000019181 | 1 | 1.781 | 4.773e-07 |  |
| ENSRNOG00000005038 | 5 | 2.637 | 4.792e-07 |  |
| ENSRNOG00000016670 | 2 | 1.537 | 4.926e-07 |  |
| ENSRNOG00000016708 | 3 | 1.773 | 5.003e-07 |  |
| ENSRNOG00000010607 | 7 | 2.858 | 5.022e-07 |  |
| ENSRNOG00000010514 | 5 | 1.978 | 5.027e-07 |  |
| ENSRNOG00000005151 | 5 | 2.333 | 5.304e-07 |  |
| ENSRNOG00000018262 | 5 | 2.043 | 5.566e-07 |  |
| ENSRNOG00000052256 | 5 | 2.055 | 5.636e-07 |  |
| ENSRNOG00000017496 | 1 | 1.717 | 5.664e-07 |  |
| ENSRNOG00000013282 | 7 | 2.582 | 5.685e-07 |  |
| ENSRNOG00000002773 | 2 | 3.35 | 5.706e-07 |  |
| ENSRNOG00000009341 | 3 | 1.889 | 5.734e-07 |  |
| ENSRNOG00000058586 | 2 | 1.707 | 5.849e-07 |  |
| ENSRNOG00000016219 | 4 | 2.142 | 5.861e-07 |  |
| ENSRNOG00000022499 | 5 | 2.352 | 5.917e-07 |  |
| ENSRNOG00000002746 | 1 | 1.611 | 5.954e-07 |  |
| ENSRNOG00000045997 | 3 | 1.973 | 6.155e-07 |  |
| ENSRNOG00000060703 | 5 | 2.548 | 6.204e-07 |  |
| ENSRNOG00000003972 | 6 | 3.263 | 6.465e-07 |  |
| ENSRNOG00000051216 | 3 | 1.784 | 6.465e-07 |  |
| ENSRNOG00000018681 | 5 | 2.365 | 6.517e-07 |  |
| ENSRNOG00000012058 | 6 | 2.27 | 6.542e-07 |  |
| ENSRNOG00000059741 | 6 | 2.731 | 6.592e-07 |  |
| ENSRNOG00000019000 | 1 | 1.523 | 6.602e-07 |  |
| ENSRNOG00000008970 | 5 | 3.224 | 6.613e-07 |  |
| ENSRNOG00000012481 | 6 | 2.738 | 6.746e-07 |  |
| ENSRNOG00000032729 | 5 | 3.177 | 6.817e-07 |  |
| ENSRNOG00000013313 | 1 | 2.037 | 6.817e-07 |  |
| ENSRNOG00000020193 | 3 | 1.989 | 6.895e-07 |  |
| ENSRNOG00000000187 | 1 | 1.797 | 6.922e-07 |  |
| ENSRNOG00000006646 | 2 | 1.592 | 6.984e-07 |  |
| ENSRNOG00000020277 | 5 | 2.015 | 7.067e-07 |  |
| ENSRNOG00000040208 | 3 | 5.031 | 7.211e-07 |  |
| ENSRNOG00000018321 | 5 | 2.654 | 7.283e-07 |  |
| ENSRNOG00000023473 | 3 | 1.928 | 7.474e-07 |  |
| ENSRNOG00000047213 | 2 | 1.49 | 7.598e-07 |  |
| ENSRNOG00000016368 | 6 | 2.601 | 7.617e-07 |  |
| ENSRNOG00000019577 | 5 | 2.699 | 7.629e-07 |  |
| ENSRNOG00000016043 | 3 | 4.113 | 7.903e-07 |  |
| ENSRNOG00000027035 | 5 | 2.404 | 7.939e-07 |  |
| ENSRNOG00000007462 | 2 | 1.741 | 7.939e-07 |  |
| ENSRNOG00000008301 | 4 | 1.596 | 8.162e-07 |  |
| ENSRNOG00000011407 | 5 | 2.222 | 8.262e-07 |  |
| ENSRNOG00000051970 | 3 | 2.144 | 8.55e-07 |  |
| ENSRNOG00000042960 | 7 | 2.532 | 8.611e-07 |  |
| ENSRNOG00000006952 | 2 | 1.666 | 8.611e-07 |  |
| ENSRNOG00000051922 | 1 | 1.969 | 8.719e-07 |  |
| ENSRNOG00000017020 | 1 | 1.496 | 8.827e-07 |  |
| ENSRNOG00000001908 | 3 | 1.66 | 9.075e-07 |  |
| ENSRNOG00000017854 | 1 | 1.719 | 9.323e-07 |  |
| ENSRNOG00000009495 | 2 | 1.873 | 9.507e-07 |  |
| ENSRNOG00000014653 | 2 | 1.975 | 9.562e-07 |  |
| ENSRNOG00000014443 | 3 | 1.812 | 9.562e-07 |  |
| ENSRNOG00000010515 | 3 | 2.048 | 9.585e-07 |  |
| ENSRNOG00000013018 | 6 | 3.348 | 9.657e-07 |  |
| ENSRNOG00000014258 | 1 | 1.53 | 9.721e-07 |  |
| ENSRNOG00000037462 | 6 | 2.193 | 9.738e-07 |  |
| ENSRNOG00000020985 | 6 | 2.982 | 9.892e-07 |  |
| ENSRNOG00000028822 | 2 | 1.924 | 9.914e-07 |  |
| ENSRNOG00000054890 | 4 | 2.043 | 9.987e-07 |  |
| ENSRNOG00000008575 | 3 | 3.933 | 1.009e-06 |  |
| ENSRNOG00000039891 | 3 | 1.9 | 1.04e-06 |  |
| ENSRNOG00000020713 | 3 | 1.949 | 1.059e-06 |  |
| ENSRNOG00000004192 | 2 | 1.479 | 1.063e-06 |  |
| ENSRNOG00000014343 | 5 | 2.586 | 1.064e-06 |  |
| ENSRNOG00000022337 | 6 | 2.6 | 1.077e-06 |  |
| ENSRNOG00000012886 | 2 | 2.449 | 1.083e-06 |  |
| ENSRNOG00000007574 | 5 | 1.985 | 1.083e-06 |  |
| ENSRNOG00000002258 | 1 | 1.671 | 1.087e-06 |  |
| ENSRNOG00000003546 | 1 | 2.12 | 1.102e-06 |  |
| ENSRNOG00000019643 | 5 | 2.284 | 1.102e-06 |  |
| ENSRNOG00000048433 | 3 | 2.196 | 1.103e-06 |  |
| ENSRNOG00000005621 | 7 | 2.76 | 1.152e-06 |  |
| ENSRNOG00000010262 | 4 | 2.649 | 1.159e-06 |  |
| ENSRNOG00000002146 | 2 | 1.795 | 1.186e-06 |  |
| ENSRNOG00000018003 | 7 | 2.765 | 1.2e-06 |  |
| ENSRNOG00000011074 | 7 | 3.133 | 1.21e-06 |  |
| ENSRNOG00000007254 | 5 | 2.847 | 1.216e-06 |  |
| ENSRNOG00000007359 | 3 | 1.843 | 1.224e-06 |  |
| ENSRNOG00000036829 | 1 | 1.521 | 1.225e-06 |  |
| ENSRNOG00000020309 | 2 | 1.925 | 1.237e-06 |  |
| ENSRNOG00000012660 | 3 | 4.752 | 1.269e-06 |  |
| ENSRNOG00000004585 | 5 | 2.295 | 1.295e-06 |  |
| ENSRNOG00000015131 | 3 | 2.247 | 1.3e-06 |  |
| ENSRNOG00000028247 | 5 | 2.112 | 1.347e-06 |  |
| ENSRNOG00000015461 | 1 | 1.811 | 1.359e-06 |  |
| ENSRNOG00000001375 | 6 | 2.418 | 1.359e-06 |  |
| ENSRNOG00000013231 | 1 | 1.655 | 1.362e-06 |  |
| ENSRNOG00000017911 | 3 | 1.834 | 1.394e-06 |  |
| ENSRNOG00000018526 | 3 | 1.985 | 1.395e-06 |  |
| ENSRNOG00000060103 | 5 | 3.662 | 1.405e-06 |  |
| ENSRNOG00000021013 | 2 | 1.596 | 1.411e-06 |  |
| ENSRNOG00000013069 | 6 | 2.735 | 1.411e-06 |  |
| ENSRNOG00000042286 | 5 | 1.719 | 1.416e-06 |  |
| ENSRNOG00000042029 | 6 | 2.79 | 1.424e-06 |  |
| ENSRNOG00000042499 | 4 | 1.991 | 1.425e-06 |  |
| ENSRNOG00000002753 | 2 | 2.205 | 1.452e-06 |  |
| ENSRNOG00000007136 | 1 | 1.634 | 1.454e-06 |  |
| ENSRNOG00000006963 | 3 | 1.634 | 1.454e-06 |  |
| ENSRNOG00000011076 | 6 | 2.599 | 1.497e-06 |  |
| ENSRNOG00000025408 | 5 | 2.361 | 1.5e-06 |  |
| ENSRNOG00000004322 | 3 | 1.698 | 1.524e-06 |  |
| ENSRNOG00000057096 | 3 | 2.224 | 1.544e-06 |  |
| ENSRNOG00000003960 | 7 | 2.811 | 1.582e-06 |  |
| ENSRNOG00000016156 | 6 | 2.397 | 1.595e-06 |  |
| ENSRNOG00000000875 | 2 | 1.632 | 1.629e-06 |  |
| ENSRNOG00000037695 | 5 | 2.535 | 1.659e-06 |  |
| ENSRNOG00000020935 | 1 | 1.563 | 1.659e-06 |  |
| ENSRNOG00000012495 | 1 | 1.558 | 1.723e-06 |  |
| ENSRNOG00000006198 | 5 | 2.546 | 1.733e-06 |  |
| ENSRNOG00000000860 | 7 | 2.647 | 1.756e-06 |  |
| ENSRNOG00000009342 | 3 | 2.073 | 1.764e-06 |  |
| ENSRNOG00000011582 | 2 | 1.589 | 1.775e-06 |  |
| ENSRNOG00000012802 | 5 | 2.114 | 1.784e-06 |  |
| ENSRNOG00000029510 | 2 | 1.835 | 1.806e-06 |  |
| ENSRNOG00000021745 | 6 | 2.244 | 1.907e-06 |  |
| ENSRNOG00000008054 | 5 | 2.5 | 1.924e-06 |  |
| ENSRNOG00000026857 | 5 | 2.048 | 1.973e-06 |  |
| ENSRNOG00000009787 | 7 | 2.785 | 1.999e-06 |  |
| ENSRNOG00000024923 | 3 | 2.319 | 2.059e-06 |  |
| ENSRNOG00000019854 | 2 | 1.63 | 2.124e-06 |  |
| ENSRNOG00000054286 | 3 | 2.293 | 2.13e-06 |  |
| ENSRNOG00000007110 | 6 | 2.5 | 2.13e-06 |  |
| ENSRNOG00000062002 | 6 | 2.46 | 2.13e-06 |  |
| ENSRNOG00000009449 | 1 | 1.561 | 2.132e-06 |  |
| ENSRNOG00000017539 | 3 | 2.201 | 2.138e-06 |  |
| ENSRNOG00000017775 | 5 | 2.18 | 2.146e-06 |  |
| ENSRNOG00000025527 | 6 | 2.873 | 2.152e-06 |  |
| ENSRNOG00000007393 | 1 | 1.821 | 2.178e-06 |  |
| ENSRNOG00000048782 | 7 | 2.814 | 2.198e-06 |  |
| ENSRNOG00000004882 | 6 | 2.941 | 2.209e-06 |  |
| ENSRNOG00000009347 | 2 | 1.479 | 2.229e-06 |  |
| ENSRNOG00000006857 | 6 | 2.28 | 2.25e-06 |  |
| ENSRNOG00000053075 | 6 | 2.769 | 2.253e-06 |  |
| ENSRNOG00000024346 | 5 | 2.504 | 2.307e-06 |  |
| ENSRNOG00000050792 | 5 | 2.809 | 2.341e-06 |  |
| ENSRNOG00000015787 | 5 | 2.474 | 2.346e-06 |  |
| ENSRNOG00000012512 | 5 | 1.951 | 2.348e-06 |  |
| ENSRNOG00000020482 | 5 | 1.944 | 2.353e-06 |  |
| ENSRNOG00000001767 | 6 | 2.084 | 2.356e-06 |  |
| ENSRNOG00000032590 | 2 | 1.564 | 2.364e-06 |  |
| ENSRNOG00000004578 | 3 | 5.15 | 2.404e-06 |  |
| ENSRNOG00000016346 | 1 | 1.416 | 2.564e-06 |  |
| ENSRNOG00000008184 | 7 | 2.878 | 2.589e-06 |  |
| ENSRNOG00000018659 | 1 | 1.617 | 2.589e-06 |  |
| ENSRNOG00000055966 | 6 | 2.174 | 2.621e-06 |  |
| ENSRNOG00000039668 | 2 | 1.717 | 2.675e-06 |  |
| ENSRNOG00000005985 | 7 | 2.941 | 2.697e-06 |  |
| ENSRNOG00000048043 | 2 | 1.621 | 2.71e-06 |  |
| ENSRNOG00000045558 | 3 | 1.846 | 2.711e-06 |  |
| ENSRNOG00000043094 | 2 | 1.621 | 2.711e-06 |  |
| ENSRNOG00000017762 | 3 | 5.356 | 2.711e-06 |  |
| ENSRNOG00000005964 | 5 | 2.257 | 2.75e-06 |  |
| ENSRNOG00000033531 | 5 | 2.321 | 2.767e-06 |  |
| ENSRNOG00000003622 | 4 | 1.606 | 2.778e-06 |  |
| ENSRNOG00000018461 | 1 | 1.923 | 2.848e-06 |  |
| ENSRNOG00000042230 | 3 | 1.879 | 2.88e-06 |  |
| ENSRNOG00000043182 | 3 | 1.614 | 2.922e-06 |  |
| ENSRNOG00000010646 | 2 | 1.547 | 2.94e-06 |  |
| ENSRNOG00000017078 | 2 | 1.712 | 2.982e-06 |  |
| ENSRNOG00000029662 | 1 | 1.49 | 3.019e-06 |  |
| ENSRNOG00000026432 | 7 | 2.38 | 3.028e-06 |  |
| ENSRNOG00000023679 | 5 | 2.235 | 3.091e-06 |  |
| ENSRNOG00000032798 | 3 | 1.824 | 3.106e-06 |  |
| ENSRNOG00000022975 | 1 | 1.501 | 3.115e-06 |  |
| ENSRNOG00000017857 | 2 | 1.533 | 3.13e-06 |  |
| ENSRNOG00000051307 | 2 | 2.043 | 3.134e-06 |  |
| ENSRNOG00000008841 | 6 | 2.431 | 3.149e-06 |  |
| ENSRNOG00000009686 | 3 | 2.949 | 3.173e-06 |  |
| ENSRNOG00000039698 | 7 | 2.977 | 3.193e-06 |  |
| ENSRNOG00000008159 | 5 | 2.153 | 3.199e-06 |  |
| ENSRNOG00000053200 | 1 | 1.483 | 3.217e-06 |  |
| ENSRNOG00000017250 | 5 | 1.996 | 3.249e-06 |  |
| ENSRNOG00000018915 | 3 | 2.089 | 3.387e-06 |  |
| ENSRNOG00000023937 | 5 | 1.881 | 3.403e-06 |  |
| ENSRNOG00000033335 | 5 | 2.131 | 3.421e-06 |  |
| ENSRNOG00000040108 | 1 | 1.821 | 3.475e-06 |  |
| ENSRNOG00000025302 | 3 | 2.1 | 3.486e-06 |  |
| ENSRNOG00000013215 | 3 | 2.041 | 3.591e-06 |  |
| ENSRNOG00000001427 | 3 | 1.709 | 3.62e-06 |  |
| ENSRNOG00000022521 | 5 | 2.021 | 3.63e-06 |  |
| ENSRNOG00000008161 | 5 | 2.69 | 3.677e-06 |  |
| ENSRNOG00000014233 | 3 | 2.378 | 3.705e-06 |  |
| ENSRNOG00000004048 | 3 | 1.608 | 3.832e-06 |  |
| ENSRNOG00000014230 | 5 | 2.808 | 3.86e-06 |  |
| ENSRNOG00000015618 | 3 | 2.292 | 3.938e-06 |  |
| ENSRNOG00000004317 | 5 | 2.645 | 3.955e-06 |  |
| ENSRNOG00000031058 | 3 | 1.607 | 3.988e-06 |  |
| ENSRNOG00000001514 | 3 | 1.722 | 3.995e-06 |  |
| ENSRNOG00000007541 | 3 | 1.891 | 4.01e-06 |  |
| ENSRNOG00000016357 | 3 | 1.881 | 4.058e-06 |  |
| ENSRNOG00000015596 | 3 | 2.013 | 4.08e-06 |  |
| ENSRNOG00000019445 | 1 | 3.636 | 4.083e-06 |  |
| ENSRNOG00000012860 | 3 | 1.816 | 4.083e-06 |  |
| ENSRNOG00000058742 | 3 | 2.501 | 4.103e-06 |  |
| ENSRNOG00000033059 | 3 | 1.941 | 4.161e-06 |  |
| ENSRNOG00000038572 | 3 | 1.979 | 4.199e-06 |  |
| ENSRNOG00000039183 | 5 | 2.08 | 4.367e-06 |  |
| ENSRNOG00000001807 | 6 | 1.968 | 4.398e-06 |  |
| ENSRNOG00000018666 | 3 | 1.552 | 4.469e-06 |  |
| ENSRNOG00000017065 | 6 | 2.263 | 4.473e-06 |  |
| ENSRNOG00000005347 | 7 | 2.345 | 4.473e-06 |  |
| ENSRNOG00000000704 | 3 | 1.708 | 4.664e-06 |  |
| ENSRNOG00000057250 | 7 | 2.69 | 4.713e-06 |  |
| ENSRNOG00000032350 | 7 | 3.106 | 4.755e-06 |  |
| ENSRNOG00000015318 | 3 | 1.91 | 4.792e-06 |  |
| ENSRNOG00000037331 | 5 | 1.913 | 4.841e-06 |  |
| ENSRNOG00000022067 | 6 | 2.347 | 4.935e-06 |  |
| ENSRNOG00000008943 | 5 | 3.057 | 4.991e-06 |  |
| ENSRNOG00000007437 | 2 | 1.48 | 5.016e-06 |  |
| ENSRNOG00000054404 | 5 | 2.599 | 5.055e-06 |  |
| ENSRNOG00000021611 | 2 | 1.931 | 5.083e-06 |  |
| ENSRNOG00000053735 | 3 | 2.485 | 5.148e-06 |  |
| ENSRNOG00000047606 | 1 | 1.693 | 5.199e-06 |  |
| ENSRNOG00000003208 | 5 | 2.083 | 5.228e-06 |  |
| ENSRNOG00000020030 | 7 | 3.445 | 5.244e-06 |  |
| ENSRNOG00000014603 | 6 | 2.952 | 5.266e-06 |  |
| ENSRNOG00000000341 | 2 | 1.631 | 5.343e-06 |  |
| ENSRNOG00000047697 | 5 | 2.415 | 5.449e-06 |  |
| ENSRNOG00000013653 | 2 | 1.705 | 5.492e-06 |  |
| ENSRNOG00000053430 | 6 | 2.307 | 5.692e-06 |  |
| ENSRNOG00000018159 | 4 | 1.48 | 5.729e-06 |  |
| ENSRNOG00000002180 | 1 | 1.432 | 5.838e-06 |  |
| ENSRNOG00000013380 | 6 | 2.29 | 5.902e-06 |  |
| ENSRNOG00000020038 | 3 | 1.946 | 6.163e-06 |  |
| ENSRNOG00000025624 | 5 | 2.151 | 6.163e-06 |  |
| ENSRNOG00000001854 | 5 | 2.042 | 6.202e-06 |  |
| ENSRNOG00000014265 | 5 | 3.678 | 6.219e-06 |  |
| ENSRNOG00000047712 | 5 | 2.315 | 6.598e-06 |  |
| ENSRNOG00000001715 | 6 | 2.307 | 6.599e-06 |  |
| ENSRNOG00000017917 | 6 | 2.117 | 6.754e-06 |  |
| ENSRNOG00000021017 | 6 | 2.188 | 6.786e-06 |  |
| ENSRNOG00000027463 | 6 | 2.32 | 6.852e-06 |  |
| ENSRNOG00000018615 | 5 | 2.325 | 6.87e-06 |  |
| ENSRNOG00000014496 | 5 | 2.017 | 6.991e-06 |  |
| ENSRNOG00000008048 | 2 | 1.563 | 7.021e-06 |  |
| ENSRNOG00000022598 | 5 | 1.969 | 7.178e-06 |  |
| ENSRNOG00000020936 | 5 | 2.594 | 7.185e-06 |  |
| ENSRNOG00000017680 | 2 | 1.953 | 7.254e-06 |  |
| ENSRNOG00000019907 | 2 | 1.818 | 7.254e-06 |  |
| ENSRNOG00000015873 | 6 | 2.307 | 7.259e-06 |  |
| ENSRNOG00000050968 | 5 | 2.032 | 7.259e-06 |  |
| ENSRNOG00000016700 | 2 | 1.589 | 7.326e-06 |  |
| ENSRNOG00000015445 | 3 | 1.735 | 7.414e-06 |  |
| ENSRNOG00000012346 | 1 | 1.394 | 7.518e-06 |  |
| ENSRNOG00000000563 | 5 | 2.008 | 7.578e-06 |  |
| ENSRNOG00000003148 | 1 | 1.679 | 7.599e-06 |  |
| ENSRNOG00000056756 | 1 | 1.432 | 7.614e-06 |  |
| ENSRNOG00000043167 | 2 | 1.528 | 7.92e-06 |  |
| ENSRNOG00000021691 | 5 | 2.359 | 7.983e-06 |  |
| ENSRNOG00000033844 | 6 | 3.98 | 8.082e-06 |  |
| ENSRNOG00000018330 | 7 | 2.61 | 8.119e-06 |  |
| ENSRNOG00000027139 | 5 | 2.152 | 8.421e-06 |  |
| ENSRNOG00000006731 | 3 | 1.855 | 8.608e-06 |  |
| ENSRNOG00000001216 | 1 | 1.623 | 8.61e-06 |  |
| ENSRNOG00000009227 | 5 | 3.413 | 8.817e-06 |  |
| ENSRNOG00000002050 | 4 | 1.458 | 9.127e-06 |  |
| ENSRNOG00000011800 | 3 | 1.947 | 9.215e-06 |  |
| ENSRNOG00000005299 | 5 | 1.842 | 9.27e-06 |  |
| ENSRNOG00000000156 | 5 | 2.275 | 9.302e-06 |  |
| ENSRNOG00000008425 | 3 | 1.769 | 9.305e-06 |  |
| ENSRNOG00000023007 | 3 | 1.713 | 9.305e-06 |  |
| ENSRNOG00000011882 | 3 | 1.727 | 9.382e-06 |  |
| ENSRNOG00000003720 | 7 | 2.224 | 9.62e-06 |  |
| ENSRNOG00000007012 | 5 | 2.415 | 9.62e-06 |  |
| ENSRNOG00000028636 | 6 | 2.263 | 9.658e-06 |  |
| ENSRNOG00000024874 | 2 | 1.674 | 9.735e-06 |  |
| ENSRNOG00000004554 | 1 | 1.674 | 9.736e-06 |  |
| ENSRNOG00000017874 | 1 | 1.501 | 9.791e-06 |  |
| ENSRNOG00000038365 | 2 | 1.509 | 9.819e-06 |  |
| ENSRNOG00000001252 | 3 | 1.946 | 9.881e-06 |  |
| ENSRNOG00000029211 | 6 | 3.176 | 1.015e-05 |  |
| ENSRNOG00000060137 | 6 | 2.234 | 1.016e-05 |  |
| ENSRNOG00000013867 | 1 | 1.637 | 1.023e-05 |  |
| ENSRNOG00000004165 | 5 | 2.057 | 1.026e-05 |  |
| ENSRNOG00000057867 | 6 | 2.442 | 1.033e-05 |  |
| ENSRNOG00000054420 | 6 | 2.649 | 1.033e-05 |  |
| ENSRNOG00000036859 | 3 | 1.991 | 1.033e-05 |  |
| ENSRNOG00000021924 | 4 | 1.576 | 1.038e-05 |  |
| ENSRNOG00000015027 | 5 | 1.925 | 1.041e-05 |  |
| ENSRNOG00000052113 | 1 | 1.515 | 1.046e-05 |  |
| ENSRNOG00000018122 | 6 | 2.346 | 1.08e-05 |  |
| ENSRNOG00000007989 | 5 | 1.979 | 1.08e-05 |  |
| ENSRNOG00000004783 | 3 | 1.901 | 1.086e-05 |  |
| ENSRNOG00000021255 | 3 | 1.649 | 1.096e-05 |  |
| ENSRNOG00000013076 | 1 | 1.405 | 1.097e-05 |  |
| ENSRNOG00000006783 | 5 | 2.065 | 1.114e-05 |  |
| ENSRNOG00000020695 | 3 | 1.615 | 1.124e-05 |  |
| ENSRNOG00000020901 | 6 | 2.581 | 1.133e-05 |  |
| ENSRNOG00000029465 | 6 | 2.627 | 1.135e-05 |  |
| ENSRNOG00000012482 | 5 | 1.908 | 1.135e-05 |  |
| ENSRNOG00000007568 | 3 | 1.624 | 1.164e-05 |  |
| ENSRNOG00000019536 | 3 | 1.632 | 1.179e-05 |  |
| ENSRNOG00000049437 | 6 | 2.556 | 1.188e-05 |  |
| ENSRNOG00000017930 | 2 | 1.507 | 1.191e-05 |  |
| ENSRNOG00000012561 | 3 | 1.646 | 1.196e-05 |  |
| ENSRNOG00000026248 | 3 | 1.761 | 1.223e-05 |  |
| ENSRNOG00000016632 | 6 | 2.328 | 1.224e-05 |  |
| ENSRNOG00000007047 | 3 | 1.531 | 1.246e-05 |  |
| ENSRNOG00000000768 | 4 | 1.963 | 1.258e-05 |  |
| ENSRNOG00000023993 | 7 | 2.848 | 1.288e-05 |  |
| ENSRNOG00000021891 | 3 | 1.842 | 1.291e-05 |  |
| ENSRNOG00000002141 | 3 | 1.723 | 1.311e-05 |  |
| ENSRNOG00000011683 | 2 | 1.497 | 1.311e-05 |  |
| ENSRNOG00000050539 | 1 | 1.726 | 1.395e-05 |  |
| ENSRNOG00000012095 | 2 | 2.031 | 1.4e-05 |  |
| ENSRNOG00000016551 | 1 | 1.692 | 1.421e-05 |  |
| ENSRNOG00000020078 | 3 | 1.785 | 1.427e-05 |  |
| ENSRNOG00000024931 | 3 | 1.601 | 1.452e-05 |  |
| ENSRNOG00000008134 | 5 | 1.773 | 1.463e-05 |  |
| ENSRNOG00000013064 | 5 | 1.714 | 1.467e-05 |  |
| ENSRNOG00000001692 | 5 | 2.071 | 1.481e-05 |  |
| ENSRNOG00000000239 | 5 | 2.661 | 1.495e-05 |  |
| ENSRNOG00000057794 | 6 | 2.191 | 1.509e-05 |  |
| ENSRNOG00000057797 | 6 | 2.6 | 1.514e-05 |  |
| ENSRNOG00000007100 | 7 | 2.36 | 1.531e-05 |  |
| ENSRNOG00000007679 | 1 | 1.445 | 1.535e-05 |  |
| ENSRNOG00000003807 | 3 | 1.855 | 1.543e-05 |  |
| ENSRNOG00000046001 | 7 | 2.614 | 1.564e-05 |  |
| ENSRNOG00000051986 | 3 | 1.419 | 1.582e-05 |  |
| ENSRNOG00000001065 | 2 | 1.563 | 1.585e-05 |  |
| ENSRNOG00000015655 | 1 | 1.46 | 1.594e-05 |  |
| ENSRNOG00000015717 | 3 | 1.752 | 1.599e-05 |  |
| ENSRNOG00000004003 | 3 | 1.934 | 1.603e-05 |  |
| ENSRNOG00000000288 | 3 | 1.835 | 1.611e-05 |  |
| ENSRNOG00000002318 | 3 | 1.697 | 1.618e-05 |  |
| ENSRNOG00000021569 | 5 | 2.216 | 1.634e-05 |  |
| ENSRNOG00000008224 | 3 | 1.782 | 1.635e-05 |  |
| ENSRNOG00000058645 | 3 | 3.578 | 1.649e-05 |  |
| ENSRNOG00000003870 | 3 | 1.769 | 1.672e-05 |  |
| ENSRNOG00000016731 | 2 | 1.606 | 1.675e-05 |  |
| ENSRNOG00000016488 | 1 | 1.521 | 1.705e-05 |  |
| ENSRNOG00000001297 | 2 | 1.628 | 1.711e-05 |  |
| ENSRNOG00000010830 | 1 | 1.675 | 1.761e-05 |  |
| ENSRNOG00000018567 | 4 | 1.781 | 1.765e-05 |  |
| ENSRNOG00000061394 | 6 | 2.16 | 1.771e-05 |  |
| ENSRNOG00000017022 | 1 | 1.44 | 1.774e-05 |  |
| ENSRNOG00000003457 | 5 | 2.237 | 1.799e-05 |  |
| ENSRNOG00000008099 | 6 | 2.231 | 1.827e-05 |  |
| ENSRNOG00000000699 | 2 | 1.629 | 1.846e-05 |  |
| ENSRNOG00000017325 | 3 | 3.704 | 1.877e-05 |  |
| ENSRNOG00000032973 | 5 | 1.814 | 1.879e-05 |  |
| ENSRNOG00000010266 | 2 | 1.56 | 1.893e-05 |  |
| ENSRNOG00000023226 | 1 | 1.889 | 1.913e-05 |  |
| ENSRNOG00000020563 | 6 | 2.69 | 1.915e-05 |  |
| ENSRNOG00000016071 | 2 | 1.552 | 1.923e-05 |  |
| ENSRNOG00000024998 | 5 | 1.898 | 1.929e-05 |  |
| ENSRNOG00000016413 | 3 | 1.654 | 1.976e-05 |  |
| ENSRNOG00000027240 | 7 | 2.755 | 1.979e-05 |  |
| ENSRNOG00000003806 | 5 | 1.863 | 1.996e-05 |  |
| ENSRNOG00000017980 | 1 | 1.562 | 2.066e-05 |  |
| ENSRNOG00000013970 | 5 | 1.824 | 2.073e-05 |  |
| ENSRNOG00000008113 | 1 | 1.655 | 2.127e-05 |  |
| ENSRNOG00000015279 | 7 | 2.864 | 2.165e-05 |  |
| ENSRNOG00000016576 | 3 | 1.662 | 2.203e-05 |  |
| ENSRNOG00000036682 | 3 | 1.663 | 2.223e-05 |  |
| ENSRNOG00000012037 | 3 | 1.559 | 2.245e-05 |  |
| ENSRNOG00000009211 | 3 | 1.682 | 2.272e-05 |  |
| ENSRNOG00000021682 | 1 | 1.559 | 2.307e-05 |  |
| ENSRNOG00000061085 | 3 | 1.714 | 2.337e-05 |  |
| ENSRNOG00000013862 | 5 | 2.065 | 2.342e-05 |  |
| ENSRNOG00000031515 | 3 | 1.778 | 2.418e-05 |  |
| ENSRNOG00000015396 | 4 | 1.453 | 2.432e-05 |  |
| ENSRNOG00000013954 | 2 | 1.534 | 2.51e-05 |  |
| ENSRNOG00000017073 | 6 | 2.272 | 2.533e-05 |  |
| ENSRNOG00000037563 | 1 | 1.44 | 2.563e-05 |  |
| ENSRNOG00000020182 | 4 | 1.446 | 2.583e-05 |  |
| ENSRNOG00000008761 | 6 | 2.465 | 2.59e-05 |  |
| ENSRNOG00000004084 | 7 | 2.53 | 2.59e-05 |  |
| ENSRNOG00000020699 | 3 | 1.576 | 2.714e-05 |  |
| ENSRNOG00000052695 | 5 | 1.812 | 2.724e-05 |  |
| ENSRNOG00000056837 | 2 | 1.508 | 2.762e-05 |  |
| ENSRNOG00000025111 | 5 | 1.936 | 2.801e-05 |  |
| ENSRNOG00000049614 | 6 | 2.298 | 2.826e-05 |  |
| ENSRNOG00000006930 | 6 | 1.976 | 2.837e-05 |  |
| ENSRNOG00000000596 | 2 | 1.421 | 2.851e-05 |  |
| ENSRNOG00000007515 | 3 | 1.505 | 2.879e-05 |  |
| ENSRNOG00000042139 | 1 | 1.456 | 2.899e-05 |  |
| ENSRNOG00000058111 | 7 | 2.337 | 2.913e-05 |  |
| ENSRNOG00000016978 | 3 | 1.714 | 3.016e-05 |  |
| ENSRNOG00000025581 | 7 | 2.584 | 3.046e-05 |  |
| ENSRNOG00000046996 | 1 | 1.382 | 3.127e-05 |  |
| ENSRNOG00000054524 | 5 | 2.451 | 3.127e-05 |  |
| ENSRNOG00000003654 | 7 | 2.478 | 3.131e-05 |  |
| ENSRNOG00000014164 | 6 | 2.136 | 3.265e-05 |  |
| ENSRNOG00000004303 | 1 | 1.502 | 3.306e-05 |  |
| ENSRNOG00000007561 | 3 | 1.82 | 3.363e-05 |  |
| ENSRNOG00000004621 | 4 | 1.694 | 3.363e-05 |  |
| ENSRNOG00000042980 | 2 | 1.546 | 3.481e-05 |  |
| ENSRNOG00000006519 | 6 | 2.111 | 3.563e-05 |  |
| ENSRNOG00000056658 | 6 | 2.467 | 3.566e-05 |  |
| ENSRNOG00000025695 | 4 | 1.523 | 3.715e-05 |  |
| ENSRNOG00000004409 | 2 | 1.412 | 3.823e-05 |  |
| ENSRNOG00000015191 | 2 | 1.544 | 3.866e-05 |  |
| ENSRNOG00000017003 | 2 | 1.511 | 3.903e-05 |  |
| ENSRNOG00000045829 | 1 | 2.128 | 4.001e-05 |  |
| ENSRNOG00000002159 | 3 | 2.395 | 4.048e-05 |  |
| ENSRNOG00000005705 | 5 | 2.239 | 4.149e-05 |  |
| ENSRNOG00000057702 | 7 | 2.799 | 4.269e-05 |  |
| ENSRNOG00000014153 | 1 | 1.425 | 4.299e-05 |  |
| ENSRNOG00000030983 | 6 | 2.436 | 4.328e-05 |  |
| ENSRNOG00000016013 | 6 | 2.331 | 4.341e-05 |  |
| ENSRNOG00000050521 | 6 | 2.415 | 4.35e-05 |  |
| ENSRNOG00000014797 | 2 | 1.694 | 4.405e-05 |  |
| ENSRNOG00000002555 | 6 | 2.72 | 4.441e-05 |  |
| ENSRNOG00000052119 | 5 | 3.677 | 4.454e-05 |  |
| ENSRNOG00000012471 | 3 | 2.902 | 4.487e-05 |  |
| ENSRNOG00000030118 | 4 | 1.351 | 4.558e-05 |  |
| ENSRNOG00000020652 | 1 | 1.524 | 4.568e-05 |  |
| ENSRNOG00000014786 | 3 | 1.656 | 4.586e-05 |  |
| ENSRNOG00000047783 | 5 | 2.622 | 4.761e-05 |  |
| ENSRNOG00000003209 | 2 | 2.875 | 4.892e-05 |  |
| ENSRNOG00000014936 | 1 | 1.48 | 4.903e-05 |  |
| ENSRNOG00000042915 | 6 | 2.356 | 4.903e-05 |  |
| ENSRNOG00000026914 | 5 | 1.767 | 4.908e-05 |  |
| ENSRNOG00000032929 | 3 | 1.657 | 5.053e-05 |  |
| ENSRNOG00000020594 | 2 | 1.535 | 5.061e-05 |  |
| ENSRNOG00000008901 | 6 | 2.224 | 5.088e-05 |  |
| ENSRNOG00000029071 | 5 | 2.083 | 5.137e-05 |  |
| ENSRNOG00000017248 | 6 | 2.171 | 5.255e-05 |  |
| ENSRNOG00000059456 | 5 | 1.712 | 5.259e-05 |  |
| ENSRNOG00000012730 | 1 | 1.361 | 5.276e-05 |  |
| ENSRNOG00000019211 | 2 | 1.414 | 5.405e-05 |  |
| ENSRNOG00000025843 | 5 | 1.744 | 5.918e-05 |  |
| ENSRNOG00000002187 | 5 | 2.3 | 5.962e-05 |  |
| ENSRNOG00000013914 | 6 | 1.864 | 6.154e-05 |  |
| ENSRNOG00000030101 | 6 | 2.151 | 6.297e-05 |  |
| ENSRNOG00000009822 | 2 | 1.557 | 6.363e-05 |  |
| ENSRNOG00000012494 | 5 | 1.977 | 6.442e-05 |  |
| ENSRNOG00000052758 | 1 | 1.372 | 6.603e-05 |  |
| ENSRNOG00000052357 | 5 | 2.104 | 6.608e-05 |  |
| ENSRNOG00000057616 | 7 | 2.631 | 6.612e-05 |  |
| ENSRNOG00000010392 | 2 | 1.687 | 6.617e-05 |  |
| ENSRNOG00000047505 | 7 | 2.397 | 6.641e-05 |  |
| ENSRNOG00000029912 | 5 | 2.034 | 6.718e-05 |  |
| ENSRNOG00000052802 | 4 | 1.437 | 6.734e-05 |  |
| ENSRNOG00000051620 | 1 | 1.403 | 6.802e-05 |  |
| ENSRNOG00000024620 | 3 | 1.426 | 6.852e-05 |  |
| ENSRNOG00000004861 | 3 | 1.626 | 6.871e-05 |  |
| ENSRNOG00000003510 | 3 | 2.083 | 6.946e-05 |  |
| ENSRNOG00000021526 | 7 | 2.535 | 7.158e-05 |  |
| ENSRNOG00000011967 | 6 | 2.138 | 7.229e-05 |  |
| ENSRNOG00000000640 | 3 | 2.331 | 7.298e-05 |  |
| ENSRNOG00000003134 | 1 | 1.516 | 7.449e-05 |  |
| ENSRNOG00000039640 | 6 | 2.968 | 7.449e-05 |  |
| ENSRNOG00000027540 | 3 | 1.698 | 7.492e-05 |  |
| ENSRNOG00000031469 | 7 | 2.388 | 7.663e-05 |  |
| ENSRNOG00000008785 | 7 | 2.561 | 7.702e-05 |  |
| ENSRNOG00000011134 | 2 | 1.524 | 7.745e-05 |  |
| ENSRNOG00000002791 | 6 | 2.078 | 7.775e-05 |  |
| ENSRNOG00000008030 | 5 | 1.847 | 7.789e-05 |  |
| ENSRNOG00000011984 | 3 | 1.723 | 7.789e-05 |  |
| ENSRNOG00000009878 | 2 | 1.351 | 7.856e-05 |  |
| ENSRNOG00000009734 | 3 | 2.788 | 8.152e-05 |  |
| ENSRNOG00000030639 | 6 | 2.279 | 8.309e-05 |  |
| ENSRNOG00000004217 | 2 | 1.411 | 8.31e-05 |  |
| ENSRNOG00000030027 | 3 | 1.446 | 8.32e-05 |  |
| ENSRNOG00000008479 | 3 | 1.558 | 8.328e-05 |  |
| ENSRNOG00000010018 | 2 | 1.48 | 8.356e-05 |  |
| ENSRNOG00000043410 | 6 | 2.13 | 8.545e-05 |  |
| ENSRNOG00000019305 | 5 | 1.963 | 8.696e-05 |  |
| ENSRNOG00000013426 | 5 | 2.077 | 8.711e-05 |  |
| ENSRNOG00000006628 | 1 | 1.629 | 8.805e-05 |  |
| ENSRNOG00000002449 | 1 | 1.367 | 8.911e-05 |  |
| ENSRNOG00000009531 | 7 | 2.641 | 9.057e-05 |  |
| ENSRNOG00000010888 | 3 | 1.878 | 9.062e-05 |  |
| ENSRNOG00000003603 | 6 | 1.86 | 9.278e-05 |  |
| ENSRNOG00000032364 | 2 | 1.577 | 9.378e-05 |  |
| ENSRNOG00000015830 | 6 | 2.194 | 9.447e-05 |  |
| ENSRNOG00000005888 | 5 | 2.085 | 9.515e-05 |  |
| ENSRNOG00000055221 | 3 | 4.964 | 9.706e-05 |  |
| ENSRNOG00000022636 | 1 | 1.654 | 9.852e-05 |  |
| ENSRNOG00000015616 | 5 | 1.823 | 9.937e-05 |  |
| ENSRNOG00000032048 | 3 | 1.836 | 0.0001008 |  |
| ENSRNOG00000021856 | 5 | 1.599 | 0.0001019 |  |
| ENSRNOG00000017699 | 7 | 2.8 | 0.0001041 |  |
| ENSRNOG00000051470 | 5 | 1.705 | 0.0001051 |  |
| ENSRNOG00000019937 | 6 | 1.902 | 0.0001063 |  |
| ENSRNOG00000037211 | 6 | 2.005 | 0.0001069 |  |
| ENSRNOG00000006723 | 7 | 2.277 | 0.0001073 |  |
| ENSRNOG00000058337 | 3 | 1.48 | 0.0001088 |  |
| ENSRNOG00000002652 | 3 | 1.483 | 0.0001107 |  |
| ENSRNOG00000034134 | 3 | 1.631 | 0.0001118 |  |
| ENSRNOG00000061216 | 4 | 1.406 | 0.0001174 |  |
| ENSRNOG00000011205 | 3 | 1.95 | 0.0001177 |  |
| ENSRNOG00000026762 | 5 | 1.586 | 0.000118 |  |
| ENSRNOG00000055025 | 5 | 4.139 | 0.0001184 |  |
| ENSRNOG00000045595 | 6 | 2.178 | 0.0001198 |  |
| ENSRNOG00000054274 | 3 | 1.896 | 0.0001205 |  |
| ENSRNOG00000007256 | 5 | 1.703 | 0.0001208 |  |
| ENSRNOG00000026607 | 6 | 2.661 | 0.0001236 |  |
| ENSRNOG00000021095 | 5 | 2.399 | 0.000124 |  |
| ENSRNOG00000006450 | 7 | 2.398 | 0.000124 |  |
| ENSRNOG00000013564 | 5 | 1.729 | 0.000124 |  |
| ENSRNOG00000050233 | 2 | 1.486 | 0.0001243 |  |
| ENSRNOG00000049410 | 7 | 2.158 | 0.0001277 |  |
| ENSRNOG00000008057 | 7 | 2.477 | 0.0001286 |  |
| ENSRNOG00000017899 | 1 | 1.752 | 0.0001292 |  |
| ENSRNOG00000058775 | 6 | 2.039 | 0.0001298 |  |
| ENSRNOG00000055650 | 5 | 1.672 | 0.0001313 |  |
| ENSRNOG00000014476 | 2 | 1.466 | 0.0001343 |  |
| ENSRNOG00000005936 | 3 | 1.524 | 0.0001345 |  |
| ENSRNOG00000005410 | 6 | 2.141 | 0.0001353 |  |
| ENSRNOG00000051383 | 5 | 1.701 | 0.0001421 |  |
| ENSRNOG00000009385 | 2 | 1.438 | 0.0001428 |  |
| ENSRNOG00000011646 | 3 | 1.756 | 0.0001436 |  |
| ENSRNOG00000016957 | 3 | 2.51 | 0.0001473 |  |
| ENSRNOG00000001555 | 2 | 1.847 | 0.0001476 |  |
| ENSRNOG00000036677 | 5 | 2.212 | 0.0001503 |  |
| ENSRNOG00000003407 | 6 | 2.036 | 0.0001531 |  |
| ENSRNOG00000046601 | 1 | 1.605 | 0.0001583 |  |
| ENSRNOG00000053895 | 6 | 2.068 | 0.0001587 |  |
| ENSRNOG00000007687 | 6 | 2.064 | 0.0001588 |  |
| ENSRNOG00000029342 | 6 | 2.046 | 0.0001649 |  |
| ENSRNOG00000002436 | 3 | 1.473 | 0.0001703 |  |
| ENSRNOG00000013017 | 7 | 2.359 | 0.0001706 |  |
| ENSRNOG00000013922 | 2 | 1.444 | 0.0001706 |  |
| ENSRNOG00000006367 | 7 | 3.159 | 0.0001725 |  |
| ENSRNOG00000050857 | 3 | 1.533 | 0.0001774 |  |
| ENSRNOG00000042888 | 5 | 1.98 | 0.0001849 |  |
| ENSRNOG00000004072 | 1 | 1.368 | 0.0001865 |  |
| ENSRNOG00000010473 | 6 | 2.324 | 0.0001869 |  |
| ENSRNOG00000027787 | 5 | 1.964 | 0.0001869 |  |
| ENSRNOG00000042944 | 5 | 2.09 | 0.0001926 |  |
| ENSRNOG00000002480 | 3 | 1.469 | 0.0001941 |  |
| ENSRNOG00000007613 | 3 | 1.577 | 0.0001943 |  |
| ENSRNOG00000008517 | 1 | 1.357 | 0.0001947 |  |
| ENSRNOG00000007925 | 7 | 2.192 | 0.0001949 |  |
| ENSRNOG00000018911 | 2 | 1.537 | 0.0001951 |  |
| ENSRNOG00000004708 | 6 | 2.648 | 0.000196 |  |
| ENSRNOG00000020867 | 5 | 2.427 | 0.0002007 |  |
| ENSRNOG00000000485 | 2 | 1.465 | 0.0002036 |  |
| ENSRNOG00000002365 | 3 | 1.944 | 0.0002065 |  |
| ENSRNOG00000015442 | 2 | 1.494 | 0.0002065 |  |
| ENSRNOG00000048771 | 2 | 1.745 | 0.0002069 |  |
| ENSRNOG00000005352 | 3 | 1.647 | 0.0002069 |  |
| ENSRNOG00000003649 | 1 | 1.537 | 0.0002071 |  |
| ENSRNOG00000056038 | 1 | 1.416 | 0.0002118 |  |
| ENSRNOG00000013694 | 5 | 1.769 | 0.0002152 |  |
| ENSRNOG00000015275 | 5 | 1.989 | 0.0002166 |  |
| ENSRNOG00000019217 | 3 | 1.636 | 0.0002167 |  |
| ENSRNOG00000025042 | 3 | 1.564 | 0.0002171 |  |
| ENSRNOG00000023309 | 6 | 2.047 | 0.0002171 |  |
| ENSRNOG00000028015 | 5 | 1.758 | 0.0002187 |  |
| ENSRNOG00000048915 | 3 | 1.438 | 0.0002238 |  |
| ENSRNOG00000033693 | 3 | 1.495 | 0.0002268 |  |
| ENSRNOG00000026238 | 5 | 1.763 | 0.0002328 |  |
| ENSRNOG00000014928 | 6 | 1.816 | 0.0002354 |  |
| ENSRNOG00000017469 | 1 | 1.556 | 0.0002358 |  |
| ENSRNOG00000017259 | 2 | 1.415 | 0.0002364 |  |
| ENSRNOG00000002932 | 3 | 1.392 | 0.0002431 |  |
| ENSRNOG00000013938 | 5 | 2.051 | 0.0002444 |  |
| ENSRNOG00000008415 | 2 | 1.601 | 0.0002455 |  |
| ENSRNOG00000054795 | 5 | 1.801 | 0.0002482 |  |
| ENSRNOG00000002524 | 3 | 1.726 | 0.0002499 |  |
| ENSRNOG00000008471 | 5 | 1.624 | 0.0002538 |  |
| ENSRNOG00000015496 | 1 | 1.365 | 0.0002554 |  |
| ENSRNOG00000005115 | 5 | 1.769 | 0.0002577 |  |
| ENSRNOG00000015024 | 6 | 1.89 | 0.0002584 |  |
| ENSRNOG00000059997 | 5 | 1.68 | 0.0002641 |  |
| ENSRNOG00000016724 | 3 | 1.633 | 0.000271 |  |
| ENSRNOG00000015562 | 4 | 2.836 | 0.0002743 |  |
| ENSRNOG00000010224 | 1 | 1.806 | 0.0002757 |  |
| ENSRNOG00000014202 | 3 | 1.498 | 0.0002763 |  |
| ENSRNOG00000054765 | 3 | 1.434 | 0.0002815 |  |
| ENSRNOG00000053239 | 5 | 1.684 | 0.0002827 |  |
| ENSRNOG00000005679 | 6 | 2.456 | 0.0002945 |  |
| ENSRNOG00000048166 | 3 | 1.582 | 0.0002961 |  |
| ENSRNOG00000045779 | 5 | 1.824 | 0.0002996 |  |
| ENSRNOG00000020993 | 5 | 1.975 | 0.0003034 |  |
| ENSRNOG00000020167 | 4 | 1.377 | 0.0003043 |  |
| ENSRNOG00000048039 | 6 | 2.276 | 0.0003044 |  |
| ENSRNOG00000001388 | 1 | 2.502 | 0.0003059 |  |
| ENSRNOG00000012874 | 6 | 2.157 | 0.0003069 |  |
| ENSRNOG00000037203 | 5 | 3.596 | 0.0003079 |  |
| ENSRNOG00000058034 | 5 | 2.68 | 0.0003079 |  |
| ENSRNOG00000049880 | 7 | 2.153 | 0.0003193 |  |
| ENSRNOG00000007367 | 1 | 1.405 | 0.0003219 |  |
| ENSRNOG00000017512 | 5 | 1.652 | 0.0003244 |  |
| ENSRNOG00000048161 | 6 | 1.905 | 0.000327 |  |
| ENSRNOG00000007281 | 6 | 2.237 | 0.000329 |  |
| ENSRNOG00000018598 | 5 | 1.714 | 0.000329 |  |
| ENSRNOG00000018851 | 5 | 1.848 | 0.0003386 |  |
| ENSRNOG00000008168 | 6 | 2.024 | 0.00034 |  |
| ENSRNOG00000053598 | 6 | 2.093 | 0.0003413 |  |
| ENSRNOG00000012318 | 2 | 1.477 | 0.0003419 |  |
| ENSRNOG00000006231 | 2 | 1.41 | 0.0003466 |  |
| ENSRNOG00000000483 | 3 | 1.699 | 0.0003467 |  |
| ENSRNOG00000006932 | 3 | 1.571 | 0.0003476 |  |
| ENSRNOG00000018366 | 7 | 2.492 | 0.0003482 |  |
| ENSRNOG00000013045 | 5 | 1.644 | 0.0003508 |  |
| ENSRNOG00000019662 | 2 | 1.356 | 0.0003568 |  |
| ENSRNOG00000025460 | 6 | 1.856 | 0.0003581 |  |
| ENSRNOG00000009157 | 3 | 1.485 | 0.0003581 |  |
| ENSRNOG00000027115 | 6 | 2.234 | 0.0003582 |  |
| ENSRNOG00000001874 | 5 | 2.108 | 0.0003606 |  |
| ENSRNOG00000027859 | 5 | 1.784 | 0.0003673 |  |
| ENSRNOG00000023257 | 2 | 1.445 | 0.0003699 |  |
| ENSRNOG00000031431 | 3 | 1.525 | 0.0003754 |  |
| ENSRNOG00000036816 | 1 | 1.345 | 0.0003772 |  |
| ENSRNOG00000006204 | 3 | 2.027 | 0.0003783 |  |
| ENSRNOG00000007159 | 2 | 1.934 | 0.0003801 |  |
| ENSRNOG00000033064 | 2 | 2.375 | 0.0003826 |  |
| ENSRNOG00000001470 | 3 | 1.289 | 0.0003829 |  |
| ENSRNOG00000013906 | 5 | 1.672 | 0.000384 |  |
| ENSRNOG00000008529 | 6 | 2.1 | 0.0003843 |  |
| ENSRNOG00000058006 | 5 | 2.583 | 0.0003847 |  |
| ENSRNOG00000049056 | 6 | 2.079 | 0.0003915 |  |
| ENSRNOG00000002950 | 5 | 1.613 | 0.000397 |  |
| ENSRNOG00000014311 | 2 | 1.534 | 0.0004015 |  |
| ENSRNOG00000057470 | 1 | 1.705 | 0.0004033 |  |
| ENSRNOG00000029399 | 2 | 1.404 | 0.0004144 |  |
| ENSRNOG00000007189 | 7 | 2.392 | 0.0004159 |  |
| ENSRNOG00000014013 | 1 | 1.478 | 0.0004298 |  |
| ENSRNOG00000018441 | 6 | 1.693 | 0.0004372 |  |
| ENSRNOG00000006604 | 6 | 1.928 | 0.0004401 |  |
| ENSRNOG00000019174 | 5 | 1.661 | 0.0004407 |  |
| ENSRNOG00000026302 | 6 | 2.008 | 0.0004434 |  |
| ENSRNOG00000055281 | 3 | 1.496 | 0.0004454 |  |
| ENSRNOG00000036649 | 5 | 1.661 | 0.0004521 |  |
| ENSRNOG00000017197 | 5 | 1.923 | 0.0004523 |  |
| ENSRNOG00000022640 | 3 | 1.668 | 0.0004625 |  |
| ENSRNOG00000042326 | 5 | 1.864 | 0.0004634 |  |
| ENSRNOG00000061070 | 5 | 1.637 | 0.000472 |  |
| ENSRNOG00000005620 | 2 | 1.31 | 0.0004739 |  |
| ENSRNOG00000010098 | 3 | 1.488 | 0.0004898 |  |
| ENSRNOG00000043416 | 1 | 1.566 | 0.0004902 |  |
| ENSRNOG00000038835 | 2 | 1.455 | 0.0004935 |  |
| ENSRNOG00000013820 | 3 | 1.667 | 0.0004954 |  |
| ENSRNOG00000010812 | 5 | 1.59 | 0.0005161 |  |
| ENSRNOG00000010666 | 6 | 2.151 | 0.000538 |  |
| ENSRNOG00000016196 | 5 | 1.887 | 0.0005385 |  |
| ENSRNOG00000010549 | 2 | 1.539 | 0.0005489 |  |
| ENSRNOG00000046955 | 3 | 1.475 | 0.0005516 |  |
| ENSRNOG00000015913 | 3 | 1.533 | 0.0005516 |  |
| ENSRNOG00000009751 | 3 | 1.582 | 0.0005561 |  |
| ENSRNOG00000043186 | 6 | 1.938 | 0.0005583 |  |
| ENSRNOG00000026053 | 6 | 2.238 | 0.0005592 |  |
| ENSRNOG00000049162 | 7 | 2.252 | 0.0005641 |  |
| ENSRNOG00000018267 | 5 | 1.544 | 0.0005775 |  |
| ENSRNOG00000019190 | 6 | 2.166 | 0.0005809 |  |
| ENSRNOG00000002792 | 6 | 2.393 | 0.0005984 |  |
| ENSRNOG00000011452 | 6 | 1.847 | 0.0006215 |  |
| ENSRNOG00000025254 | 7 | 2.28 | 0.0006228 |  |
| ENSRNOG00000059660 | 7 | 2.572 | 0.0006233 |  |
| ENSRNOG00000020142 | 6 | 1.956 | 0.0006345 |  |
| ENSRNOG00000003522 | 6 | 1.947 | 0.0006345 |  |
| ENSRNOG00000009372 | 6 | 2.274 | 0.0006346 |  |
| ENSRNOG00000004411 | 3 | 1.824 | 0.0006349 |  |
| ENSRNOG00000017605 | 5 | 1.714 | 0.0006349 |  |
| ENSRNOG00000003254 | 6 | 2.034 | 0.0006358 |  |
| ENSRNOG00000005348 | 1 | 1.585 | 0.0006513 |  |
| ENSRNOG00000042449 | 1 | 1.302 | 0.0006564 |  |
| ENSRNOG00000011208 | 7 | 2.307 | 0.0006686 |  |
| ENSRNOG00000009912 | 1 | 1.379 | 0.0006693 |  |
| ENSRNOG00000004242 | 5 | 1.664 | 0.0006778 |  |
| ENSRNOG00000006025 | 3 | 1.632 | 0.0006834 |  |
| ENSRNOG00000019740 | 5 | 1.641 | 0.0006839 |  |
| ENSRNOG00000012932 | 2 | 2.058 | 0.0006839 |  |
| ENSRNOG00000034015 | 1 | 1.344 | 0.0006932 |  |
| ENSRNOG00000021247 | 5 | 1.7 | 0.0006971 |  |
| ENSRNOG00000003713 | 6 | 2.719 | 0.0006979 |  |
| ENSRNOG00000022859 | 6 | 2.316 | 0.0007075 |  |
| ENSRNOG00000050430 | 2 | 1.404 | 0.0007088 |  |
| ENSRNOG00000036571 | 3 | 3.627 | 0.0007149 |  |
| ENSRNOG00000012698 | 2 | 1.391 | 0.0007423 |  |
| ENSRNOG00000007682 | 2 | 1.797 | 0.0007569 |  |
| ENSRNOG00000025587 | 7 | 2.306 | 0.000758 |  |
| ENSRNOG00000002506 | 3 | 1.473 | 0.0007616 |  |
| ENSRNOG00000039350 | 7 | 2.464 | 0.0007645 |  |
| ENSRNOG00000019129 | 6 | 1.965 | 0.0007859 |  |
| ENSRNOG00000021412 | 1 | 1.61 | 0.0007916 |  |
| ENSRNOG00000036960 | 1 | 1.346 | 0.0007999 |  |
| ENSRNOG00000042289 | 2 | 1.569 | 0.0008142 |  |
| ENSRNOG00000001374 | 7 | 2.312 | 0.0008169 |  |
| ENSRNOG00000000606 | 6 | 2.077 | 0.0008399 |  |
| ENSRNOG00000008012 | 4 | 1.448 | 0.0008561 |  |
| ENSRNOG00000051813 | 6 | 2.042 | 0.0008616 |  |
| ENSRNOG00000013152 | 3 | 1.627 | 0.0008831 |  |
| ENSRNOG00000016469 | 3 | 1.717 | 0.0008845 |  |
| ENSRNOG00000012534 | 3 | 1.464 | 0.0009175 |  |
| ENSRNOG00000007454 | 6 | 1.891 | 0.0009191 |  |
| ENSRNOG00000059203 | 5 | 1.623 | 0.0009235 |  |
| ENSRNOG00000000105 | 5 | 1.581 | 0.0009414 |  |
| ENSRNOG00000005386 | 2 | 1.52 | 0.0009576 |  |
| ENSRNOG00000011394 | 5 | 1.844 | 0.0009624 |  |
| ENSRNOG00000031700 | 2 | 1.397 | 0.0009679 |  |
| ENSRNOG00000016643 | 2 | 1.37 | 0.0009833 |  |
| ENSRNOG00000019822 | 2 | 1.689 | 0.0009858 |  |
| ENSRNOG00000009433 | 6 | 2.19 | 0.000996 |  |
| ENSRNOG00000014125 | 2 | 1.4 | 0.0009977 |  |
| ENSRNOG00000009079 | 5 | 1.698 | 0.0009978 |  |
| ENSRNOG00000028404 | 6 | 2.03 | 0.001042 |  |
| ENSRNOG00000022533 | 3 | 1.633 | 0.001063 |  |
| ENSRNOG00000018390 | 1 | 1.397 | 0.001065 |  |
| ENSRNOG00000002820 | 3 | 1.47 | 0.001101 |  |
| ENSRNOG00000001198 | 6 | 1.978 | 0.001132 |  |
| ENSRNOG00000012172 | 2 | 1.392 | 0.001137 |  |
| ENSRNOG00000011435 | 6 | 1.686 | 0.001145 |  |
| ENSRNOG00000061895 | 2 | 1.458 | 0.001153 |  |
| ENSRNOG00000010663 | 6 | 1.883 | 0.001168 |  |
| ENSRNOG00000007810 | 6 | 2.13 | 0.001191 |  |
| ENSRNOG00000021041 | 5 | 1.545 | 0.001192 |  |
| ENSRNOG00000009779 | 4 | 1.471 | 0.001196 |  |
| ENSRNOG00000052354 | 2 | 1.529 | 0.001207 |  |
| ENSRNOG00000047867 | 6 | 1.636 | 0.00122 |  |
| ENSRNOG00000008697 | 5 | 1.722 | 0.001233 |  |
| ENSRNOG00000004109 | 3 | 1.504 | 0.001263 |  |
| ENSRNOG00000038877 | 3 | 1.49 | 0.001263 |  |
| ENSRNOG00000012603 | 2 | 1.426 | 0.001263 |  |
| ENSRNOG00000022921 | 3 | 1.589 | 0.001283 |  |
| ENSRNOG00000003120 | 4 | 1.387 | 0.001311 |  |
| ENSRNOG00000000894 | 3 | 1.498 | 0.001319 |  |
| ENSRNOG00000020325 | 3 | 1.34 | 0.001322 |  |
| ENSRNOG00000046202 | 3 | 1.53 | 0.001363 |  |
| ENSRNOG00000002289 | 5 | 2.054 | 0.001388 |  |
| ENSRNOG00000053753 | 6 | 1.716 | 0.001392 |  |
| ENSRNOG00000024008 | 3 | 1.374 | 0.001418 |  |
| ENSRNOG00000038616 | 6 | 2.004 | 0.001431 |  |
| ENSRNOG00000024349 | 7 | 2.163 | 0.001433 |  |
| ENSRNOG00000009331 | 1 | 1.316 | 0.001436 |  |
| ENSRNOG00000005565 | 5 | 1.501 | 0.00146 |  |
| ENSRNOG00000050647 | 5 | 2.271 | 0.001495 |  |
| ENSRNOG00000014698 | 3 | 1.363 | 0.001497 |  |
| ENSRNOG00000045992 | 2 | 1.417 | 0.001537 |  |
| ENSRNOG00000036959 | 6 | 2.263 | 0.001589 |  |
| ENSRNOG00000021644 | 2 | 1.324 | 0.001589 |  |
| ENSRNOG00000010011 | 1 | 1.446 | 0.001589 |  |
| ENSRNOG00000025881 | 6 | 1.599 | 0.00162 |  |
| ENSRNOG00000050869 | 3 | 1.701 | 0.001633 |  |
| ENSRNOG00000002396 | 2 | 1.435 | 0.001697 |  |
| ENSRNOG00000008450 | 5 | 1.801 | 0.001705 |  |
| ENSRNOG00000003927 | 6 | 1.984 | 0.001712 |  |
| ENSRNOG00000011439 | 2 | 1.321 | 0.001721 |  |
| ENSRNOG00000021031 | 4 | 1.294 | 0.001737 |  |
| ENSRNOG00000025584 | 5 | 1.523 | 0.001766 |  |
| ENSRNOG00000007102 | 3 | 1.411 | 0.001772 |  |
| ENSRNOG00000017720 | 1 | 1.477 | 0.001773 |  |
| ENSRNOG00000006740 | 5 | 1.517 | 0.001795 |  |
| ENSRNOG00000017731 | 3 | 1.399 | 0.001816 |  |
| ENSRNOG00000016701 | 5 | 1.529 | 0.001892 |  |
| ENSRNOG00000007970 | 3 | 1.291 | 0.001912 |  |
| ENSRNOG00000003479 | 5 | 1.466 | 0.001951 |  |
| ENSRNOG00000055371 | 7 | 2.112 | 0.001989 |  |
| ENSRNOG00000022565 | 2 | 1.367 | 0.001991 |  |
| ENSRNOG00000012721 | 2 | 1.831 | 0.002024 |  |
| ENSRNOG00000015975 | 6 | 1.816 | 0.002027 |  |
| ENSRNOG00000019885 | 1 | 1.38 | 0.00203 |  |
| ENSRNOG00000059510 | 6 | 2.097 | 0.002034 |  |
| ENSRNOG00000015075 | 6 | 1.764 | 0.002057 |  |
| ENSRNOG00000015895 | 2 | 1.394 | 0.002063 |  |
| ENSRNOG00000001704 | 6 | 1.838 | 0.002071 |  |
| ENSRNOG00000025297 | 5 | 1.596 | 0.002151 |  |
| ENSRNOG00000054764 | 3 | 1.383 | 0.002163 |  |
| ENSRNOG00000030719 | 2 | 1.877 | 0.002174 |  |
| ENSRNOG00000015773 | 3 | 1.468 | 0.002191 |  |
| ENSRNOG00000016535 | 5 | 1.583 | 0.002235 |  |
| ENSRNOG00000002428 | 6 | 1.884 | 0.002268 |  |
| ENSRNOG00000018500 | 5 | 1.595 | 0.002268 |  |
| ENSRNOG00000016163 | 3 | 1.509 | 0.002278 |  |
| ENSRNOG00000021155 | 3 | 1.382 | 0.002282 |  |
| ENSRNOG00000001518 | 1 | 1.476 | 0.002335 |  |
| ENSRNOG00000017283 | 6 | 1.652 | 0.002348 |  |
| ENSRNOG00000037845 | 6 | 1.633 | 0.002352 |  |
| ENSRNOG00000003033 | 5 | 1.684 | 0.002362 |  |
| ENSRNOG00000061230 | 6 | 1.877 | 0.002401 |  |
| ENSRNOG00000000693 | 7 | 2.107 | 0.002405 |  |
| ENSRNOG00000014464 | 3 | 1.395 | 0.002413 |  |
| ENSRNOG00000058568 | 5 | 2.32 | 0.002417 |  |
| ENSRNOG00000002122 | 2 | 1.243 | 0.002442 |  |
| ENSRNOG00000014703 | 5 | 1.786 | 0.00247 |  |
| ENSRNOG00000014251 | 2 | 1.358 | 0.002491 |  |
| ENSRNOG00000012513 | 3 | 1.381 | 0.002499 |  |
| ENSRNOG00000009343 | 7 | 2.303 | 0.002499 |  |
| ENSRNOG00000025245 | 3 | 1.511 | 0.002576 |  |
| ENSRNOG00000009907 | 6 | 1.959 | 0.002689 |  |
| ENSRNOG00000014199 | 2 | 1.358 | 0.002691 |  |
| ENSRNOG00000019118 | 1 | 1.61 | 0.002733 |  |
| ENSRNOG00000021795 | 7 | 2.132 | 0.002748 |  |
| ENSRNOG00000033744 | 5 | 1.499 | 0.002748 |  |
| ENSRNOG00000005907 | 3 | 1.44 | 0.002775 |  |
| ENSRNOG00000017403 | 3 | 1.406 | 0.00279 |  |
| ENSRNOG00000058847 | 3 | 1.929 | 0.0028 |  |
| ENSRNOG00000011502 | 7 | 2.299 | 0.002834 |  |
| ENSRNOG00000001571 | 7 | 2.138 | 0.002838 |  |
| ENSRNOG00000020653 | 1 | 1.483 | 0.002851 |  |
| ENSRNOG00000018760 | 5 | 1.542 | 0.002863 |  |
| ENSRNOG00000008736 | 2 | 1.466 | 0.002875 |  |
| ENSRNOG00000013572 | 5 | 1.43 | 0.002876 |  |
| ENSRNOG00000054217 | 7 | 1.973 | 0.002912 |  |
| ENSRNOG00000024808 | 5 | 1.5 | 0.002913 |  |
| ENSRNOG00000016692 | 1 | 1.333 | 0.00294 |  |
| ENSRNOG00000037339 | 5 | 1.858 | 0.00298 |  |
| ENSRNOG00000027030 | 3 | 1.494 | 0.00309 |  |
| ENSRNOG00000020908 | 6 | 1.6 | 0.003098 |  |
| ENSRNOG00000032238 | 3 | 1.381 | 0.003119 |  |
| ENSRNOG00000038881 | 1 | 1.308 | 0.003151 |  |
| ENSRNOG00000024181 | 7 | 2.407 | 0.003197 |  |
| ENSRNOG00000005970 | 5 | 1.621 | 0.003231 |  |
| ENSRNOG00000022704 | 7 | 2.215 | 0.00326 |  |
| ENSRNOG00000007567 | 5 | 1.586 | 0.003282 |  |
| ENSRNOG00000003835 | 1 | 1.26 | 0.003293 |  |
| ENSRNOG00000020881 | 2 | 1.292 | 0.00335 |  |
| ENSRNOG00000002052 | 4 | 1.298 | 0.003391 |  |
| ENSRNOG00000003515 | 4 | 1.359 | 0.003407 |  |
| ENSRNOG00000051196 | 1 | 1.316 | 0.003448 |  |
| ENSRNOG00000054218 | 5 | 1.557 | 0.00345 |  |
| ENSRNOG00000019430 | 1 | 1.338 | 0.003506 |  |
| ENSRNOG00000048881 | 5 | 1.592 | 0.00355 |  |
| ENSRNOG00000008332 | 3 | 1.565 | 0.0036 |  |
| ENSRNOG00000002057 | 6 | 1.72 | 0.003606 |  |
| ENSRNOG00000012580 | 2 | 1.478 | 0.003653 |  |
| ENSRNOG00000001448 | 1 | 1.356 | 0.003741 |  |
| ENSRNOG00000050251 | 3 | 1.411 | 0.003896 |  |
| ENSRNOG00000053366 | 7 | 1.7 | 0.003897 |  |
| ENSRNOG00000026415 | 4 | 1.349 | 0.003903 |  |
| ENSRNOG00000014461 | 2 | 1.34 | 0.003912 |  |
| ENSRNOG00000036921 | 5 | 1.992 | 0.003918 |  |
| ENSRNOG00000048834 | 1 | 1.416 | 0.003929 |  |
| ENSRNOG00000003772 | 1 | 1.456 | 0.003939 |  |
| ENSRNOG00000010777 | 6 | 1.839 | 0.003963 |  |
| ENSRNOG00000014278 | 3 | 1.393 | 0.004001 |  |
| ENSRNOG00000028982 | 7 | 1.959 | 0.004003 |  |
| ENSRNOG00000017703 | 1 | 1.297 | 0.004004 |  |
| ENSRNOG00000046245 | 6 | 1.584 | 0.004027 |  |
| ENSRNOG00000005007 | 7 | 2.118 | 0.004031 |  |
| ENSRNOG00000023261 | 6 | 1.832 | 0.004133 |  |
| ENSRNOG00000021437 | 3 | 1.639 | 0.004261 |  |
| ENSRNOG00000014124 | 5 | 1.389 | 0.004309 |  |
| ENSRNOG00000001425 | 3 | 1.499 | 0.004315 |  |
| ENSRNOG00000010941 | 2 | 1.346 | 0.004397 |  |
| ENSRNOG00000058288 | 3 | 1.431 | 0.004461 |  |
| ENSRNOG00000018812 | 5 | 1.78 | 0.004495 |  |
| ENSRNOG00000036762 | 6 | 1.72 | 0.004495 |  |
| ENSRNOG00000020518 | 5 | 1.403 | 0.00453 |  |
| ENSRNOG00000021261 | 3 | 1.328 | 0.0046 |  |
| ENSRNOG00000014902 | 6 | 1.628 | 0.00464 |  |
| ENSRNOG00000052940 | 7 | 2.507 | 0.004704 |  |
| ENSRNOG00000050819 | 3 | 1.48 | 0.004705 |  |
| ENSRNOG00000006632 | 1 | 1.255 | 0.004705 |  |
| ENSRNOG00000061231 | 3 | 1.543 | 0.004756 |  |
| ENSRNOG00000032708 | 4 | 1.592 | 0.00478 |  |
| ENSRNOG00000020332 | 6 | 1.83 | 0.005012 |  |
| ENSRNOG00000017087 | 2 | 1.298 | 0.005256 |  |
| ENSRNOG00000003908 | 3 | 1.331 | 0.005369 |  |
| ENSRNOG00000015157 | 3 | 1.55 | 0.005377 |  |
| ENSRNOG00000008025 | 7 | 1.816 | 0.005387 |  |
| ENSRNOG00000014863 | 2 | 1.303 | 0.005442 |  |
| ENSRNOG00000029768 | 6 | 2.061 | 0.005463 |  |
| ENSRNOG00000006569 | 7 | 1.793 | 0.005491 |  |
| ENSRNOG00000022911 | 3 | 1.492 | 0.005548 |  |
| ENSRNOG00000016995 | 6 | 1.538 | 0.005648 |  |
| ENSRNOG00000021839 | 6 | 1.821 | 0.005809 |  |
| ENSRNOG00000011649 | 5 | 1.574 | 0.005973 |  |
| ENSRNOG00000014187 | 6 | 1.735 | 0.006096 |  |
| ENSRNOG00000011321 | 3 | 1.396 | 0.006118 |  |
| ENSRNOG00000019609 | 5 | 1.554 | 0.006143 |  |
| ENSRNOG00000020891 | 6 | 1.626 | 0.006207 |  |
| ENSRNOG00000014182 | 1 | 1.32 | 0.006226 |  |
| ENSRNOG00000019584 | 7 | 2.213 | 0.006257 |  |
| ENSRNOG00000018971 | 2 | 1.268 | 0.006501 |  |
| ENSRNOG00000021100 | 2 | 1.246 | 0.006553 |  |
| ENSRNOG00000001431 | 3 | 1.304 | 0.00674 |  |
| ENSRNOG00000004610 | 3 | 1.636 | 0.006765 |  |
| ENSRNOG00000021137 | 6 | 1.666 | 0.006774 |  |
| ENSRNOG00000021966 | 6 | 1.667 | 0.006809 |  |
| ENSRNOG00000017828 | 6 | 1.965 | 0.006832 |  |
| ENSRNOG00000056772 | 7 | 1.935 | 0.006867 |  |
| ENSRNOG00000005492 | 2 | 1.273 | 0.006883 |  |
| ENSRNOG00000009030 | 5 | 1.557 | 0.006891 |  |
| ENSRNOG00000015816 | 3 | 1.325 | 0.007007 |  |
| ENSRNOG00000016299 | 3 | 1.492 | 0.007043 |  |
| ENSRNOG00000001187 | 2 | 1.389 | 0.00706 |  |
| ENSRNOG00000026217 | 6 | 1.62 | 0.007148 |  |
| ENSRNOG00000007329 | 2 | 1.309 | 0.007333 |  |
| ENSRNOG00000010753 | 3 | 1.655 | 0.007447 |  |
| ENSRNOG00000016050 | 2 | 1.351 | 0.007447 |  |
| ENSRNOG00000022136 | 7 | 1.632 | 0.007447 |  |
| ENSRNOG00000000528 | 3 | 1.285 | 0.007479 |  |
| ENSRNOG00000008600 | 7 | 1.626 | 0.007492 |  |
| ENSRNOG00000023546 | 3 | 1.598 | 0.007543 |  |
| ENSRNOG00000026577 | 3 | 1.744 | 0.007547 |  |
| ENSRNOG00000004680 | 6 | 1.621 | 0.007553 |  |
| ENSRNOG00000061373 | 5 | 1.454 | 0.007577 |  |
| ENSRNOG00000033087 | 7 | 1.943 | 0.007675 |  |
| ENSRNOG00000027096 | 5 | 1.616 | 0.007735 |  |
| ENSRNOG00000005309 | 5 | 1.721 | 0.007767 |  |
| ENSRNOG00000013793 | 7 | 2.144 | 0.007822 |  |
| ENSRNOG00000012208 | 5 | 1.398 | 0.008149 |  |
| ENSRNOG00000043044 | 3 | 1.303 | 0.008149 |  |
| ENSRNOG00000014378 | 6 | 2 | 0.008175 |  |
| ENSRNOG00000021847 | 5 | 1.373 | 0.008175 |  |
| ENSRNOG00000056562 | 2 | 1.31 | 0.00823 |  |
| ENSRNOG00000021793 | 5 | 1.552 | 0.008253 |  |
| ENSRNOG00000015582 | 2 | 1.281 | 0.00826 |  |
| ENSRNOG00000002139 | 6 | 1.928 | 0.00826 |  |
| ENSRNOG00000004794 | 5 | 1.416 | 0.008263 |  |
| ENSRNOG00000019778 | 1 | 1.287 | 0.008435 |  |
| ENSRNOG00000016291 | 3 | 1.378 | 0.008538 |  |
| ENSRNOG00000003346 | 5 | 1.403 | 0.008546 |  |
| ENSRNOG00000046313 | 6 | 1.812 | 0.00865 |  |
| ENSRNOG00000049096 | 5 | 1.651 | 0.008792 |  |
| ENSRNOG00000015076 | 6 | 1.833 | 0.008974 |  |
| ENSRNOG00000054964 | 5 | 1.427 | 0.009173 |  |
| ENSRNOG00000015448 | 7 | 2.125 | 0.009179 |  |
| ENSRNOG00000013000 | 3 | 1.448 | 0.009266 |  |
| ENSRNOG00000033017 | 6 | 1.774 | 0.009277 |  |
| ENSRNOG00000049380 | 5 | 1.384 | 0.009306 |  |
| ENSRNOG00000025705 | 3 | 1.365 | 0.009316 |  |
| ENSRNOG00000010045 | 3 | 1.633 | 0.009317 |  |
| ENSRNOG00000054954 | 5 | 1.636 | 0.009601 |  |
| ENSRNOG00000016316 | 2 | 1.254 | 0.009722 |  |
| ENSRNOG00000028768 | 3 | 1.386 | 0.009753 |  |
| ENSRNOG00000001769 | 2 | 1.359 | 0.009771 |  |
| ENSRNOG00000037299 | 7 | 1.644 | 0.009795 |  |
| ENSRNOG00000014961 | 6 | 1.979 | 0.009833 |  |
| ENSRNOG00000016608 | 6 | 1.862 | 0.009924 |  |
| ENSRNOG00000020865 | 3 | 1.443 | 0.009931 |  |
| ENSRNOG00000013061 | 2 | 1.318 | 0.009982 |  |
